# Combining flavin photocatalysis with parallel synthesis: a general platform to optimize peptides with non-proteinogenic amino acids†

**DOI:** 10.1039/d1sc02562g

**Published:** 2021-06-30

**Authors:** Jacob R. Immel, Maheshwerreddy Chilamari, Steven Bloom

**Affiliations:** Department of Medicinal Chemistry, The University of Kansas Integrated Science Building Lawrence KS 66045 USA spbloom@ku.edu

## Abstract

Most peptide drugs contain non-proteinogenic amino acids (NPAAs), born out through extensive structure–activity relationship (SAR) studies using solid-phase peptide synthesis (SPPS). Synthetically laborious and expensive to manufacture, NPAAs also can have poor coupling efficiencies allowing only a small fraction to be sampled by conventional SPPS. To gain general access to NPAA-containing peptides, we developed a first-generation platform that merges contemporary flavin photocatalysis with parallel synthesis to simultaneously make, purify, quantify, and even test up to 96 single-NPAA peptide variants *via* the unique combination of boronic acids and a dehydroalanine residue in a peptide. We showcase the power of our newly minted platform to introduce NPAAs of diverse chemotypes-aliphatic, aromatic, heteroaromatic-directly into peptides, including 15 entirely new residues, and to evolve a simple proteinogenic peptide into an unnatural inhibitor of thrombin by non-classical peptide SAR.

## Abstract 2

We report a non-classical approach to interrogate peptides with non-proteinogenic amino acids *via* flavin photocatalysis. We establish a new platform to make, purify, quantify, and biochemically test up to 96 peptide variants in batch.

## Graphical abstract

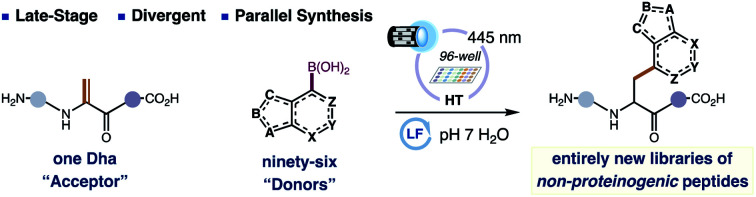

## Introduction

Replacing the endogenous amino acids of ordinary peptides with non-proteinogenic amino acids (NPAAs) can greatly enhance the utility of peptides as medicines, materials, and as synthetic probes for chemical biology.^1^ In the context of peptide drug discovery, NPAAs expand upon the limited functionality of traditional amino acids (AA) by taking advantage of non-covalent interactions (*e.g.* hydrogen bonding, electrostatic interactions, and cation–π interactions) that are often unavailable to the side chains offered by the endogenous AAs.^2^ The incorporation of NPAAs in place of standard AAs can increase the metabolic stability,^3^ half-life,^4^ cell penetrance,^5,6^ and the overall affinity of the peptide for its cognate receptor.^7^ To optimize a peptide with NPAAs, solid-phase peptide synthesis (SPPS) is primarily used to replace each position with a chemically distinct NPAA which is then evaluated for its biochemical activity, a.k.a. contemporary structure–activity relationship (SAR).^8^ Unfortunately, SPPS is a less than ideal platform for exploring peptide SAR with NPAAs. Fmoc protected NPAAs (required for standard SPPS) are inordinately expensive, have low commercial availability, are difficult to synthesize, and couple less efficiently than traditional AAs.^9^ A means to rapidly and efficiently interrogate the AAs of ordinary peptides with established and fundamentally new NPAAs is vital for the continued emergence of next generation peptide biopharmaceuticals.

**Fig. 1:**
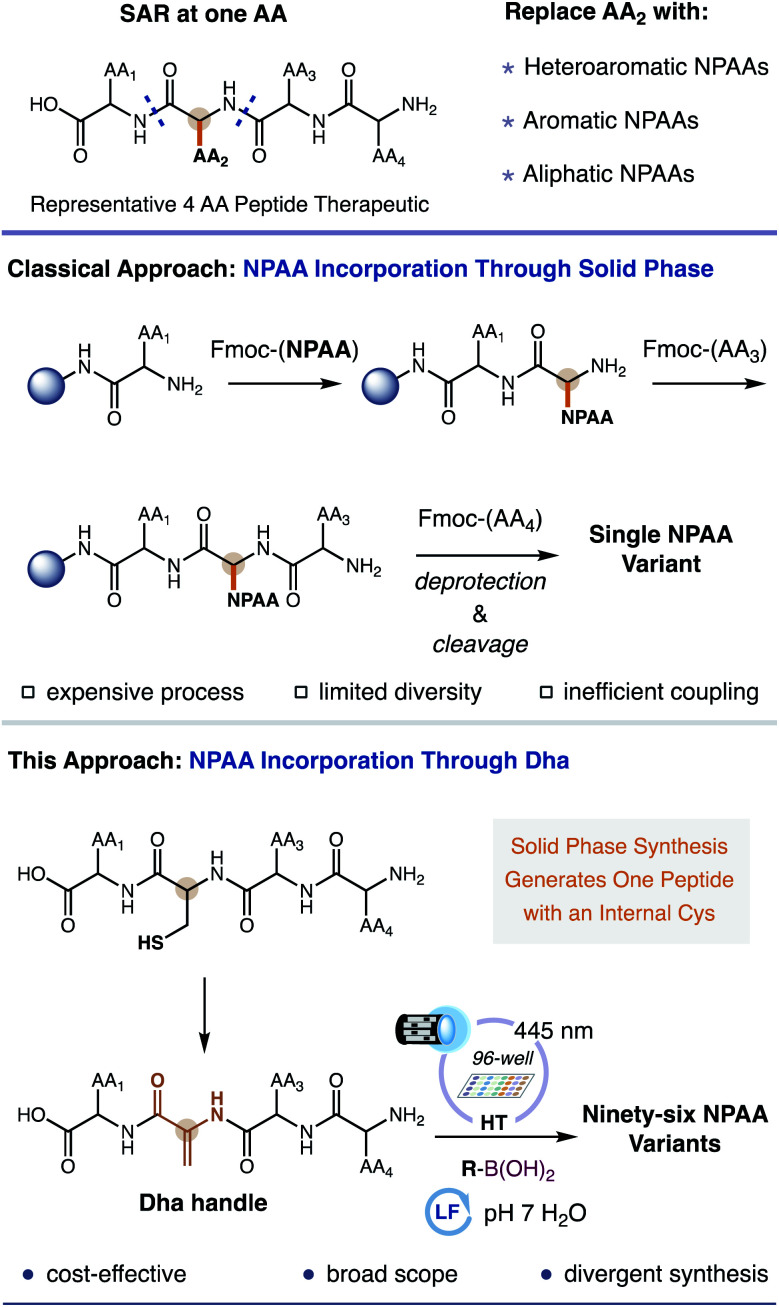
Classical peptide SAR with NPAAs occurs through sequential or parallel synthesis *via* solid phase. Our approach requires one internal Dha peptide to generate numerous variants from abundant boronic acids.

As an alternative to the direct use of SPPS to incorporate NPAAs, methods have been examined that insert NPAAs at a defined location in the peptide *via* late-stage fragment coupling. This conceptionally new approach to NPAA peptides breaks apart the NPAA into its constituent amide backbone and its side chain. The amide backbone is derived from a dehydroalanine (Dha) acceptor residue,^10^ readily accessible from a cysteine residue,^11^ and the side chain is delivered by various functional donors including carbon and heteroatom nucleophiles,^12^ organometallic reagents or metal-based complexes (*i.e.* palladium, rhodium, copper, and cobalt complexes),^13^ and *C*-centered radicals.^14^ Of all these, *C*-centered radicals are particularly advantageous due to their heightened reactivity, chemical accessibility through various functional groups, and innate aqueous compatibility.^15,16^ Unfortunately, current radical-based approaches for Dha-containing peptides are severely limited in scope, reliant on alkyl or α-heteroatom-based radicals, forging only a fraction of potential side chains.^17^ Aromatic and heteroaromatic side chains are elusive for radical based approaches. Furthermore, available methods have not been demonstrated for synthesizing comprehensive NPAA peptide libraries that can be biochemically evaluated in tandem. The inability to access a variety of side chain chemotypes in parallel renders current open-shell approaches impractical for peptide SAR with NPAAs. Our group recently reported a novel method for generating disparate heteroaromatic, aromatic, and aliphatic radicals under biocompatible conditions from commercially abundant boronic acids using an organic flavin photocatalyst.^18^ We hypothesized that our method to access disparate radicals could be combined with a Dha-containing peptide in a high-throughput (HT) system (*i.e.*, 96-well) to facilitate rapid peptide SAR (HT-*p*SAR), simultaneously making and evaluating entire libraries of peptide analogs including those with previously inaccessible NPAAs (Fig. 1).

## Results and discussion

**Fig. 2:**
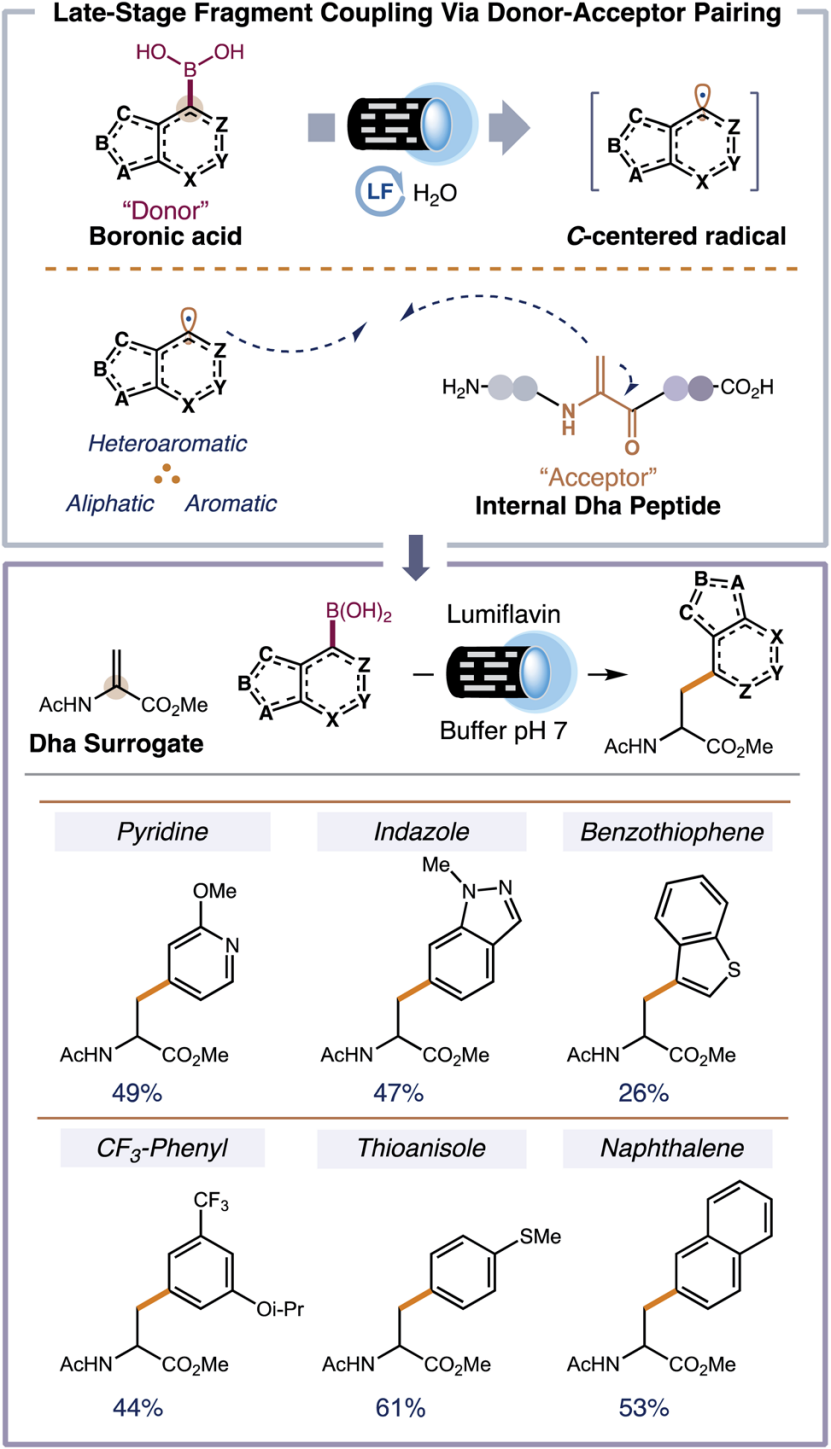
Late-stage coupling between *C*-centered radical donors with electrophilic dehydroalanine acceptors incorporates NPAAs directly into peptides. All amino acid reactions were performed using 0.35 mmol AcHN-Dha-CO_2_Me, 1.05 mmol of boronic acid, and 0.035 mmol of lumiflavin in a phosphate buffer solution at pH 7 [10 mM]. Reactions were degassed with N_2_ and irradiated for 15 h.

To evaluate flavin photocatalysis for Dha fragment coupling, we selected 2-methoxypyridine-4-boronic acid **1A** as a prototypical heterocycle and the commercial methyl 2-acetamidoacrylate (AcHN-**Dha**-CO_2_Me) as a surrogate for an internal Dha residue in a peptide. The combination of 5 mol% lumiflavin and 1.2 equiv. of boronic acid in an 85 : 15 buffered solution of water and DMF (10 mM overall concentration) afforded 8% ^1^H NMR yield of the heterocyclic NPAA under blue light irradiation (440 nm). Other photocatalysts were surveyed (*e.g.* 9-mesityl-10-methylacridinium tetrafluoroborate, (Ir[dF(CF_3_)ppy]_2_(dtbpy))PF_6_, and Ru(bpy)_3_Cl_2_·6H_2_O) but gave unsatisfactory results (<1% ^1^H NMR yield). Only flavin-based photocatalysts furnished the desired NPAA in appreciable quantities; of which, lumiflavin afforded the best result. After thorough optimization of the reaction conditions—cosolvent, buffer, concentration, and catalyst loading—, we prepared the desired pyridine-containing NPAA in 49% isolated yield (see ESI† for full optimization). Applying our optimized conditions to the synthesis of other NPAAs gave moderate yields on a 50 mg scale. Some representative NPAAs that can be accessed using our methodology are shown in Fig. 2.

**Fig. 3:**
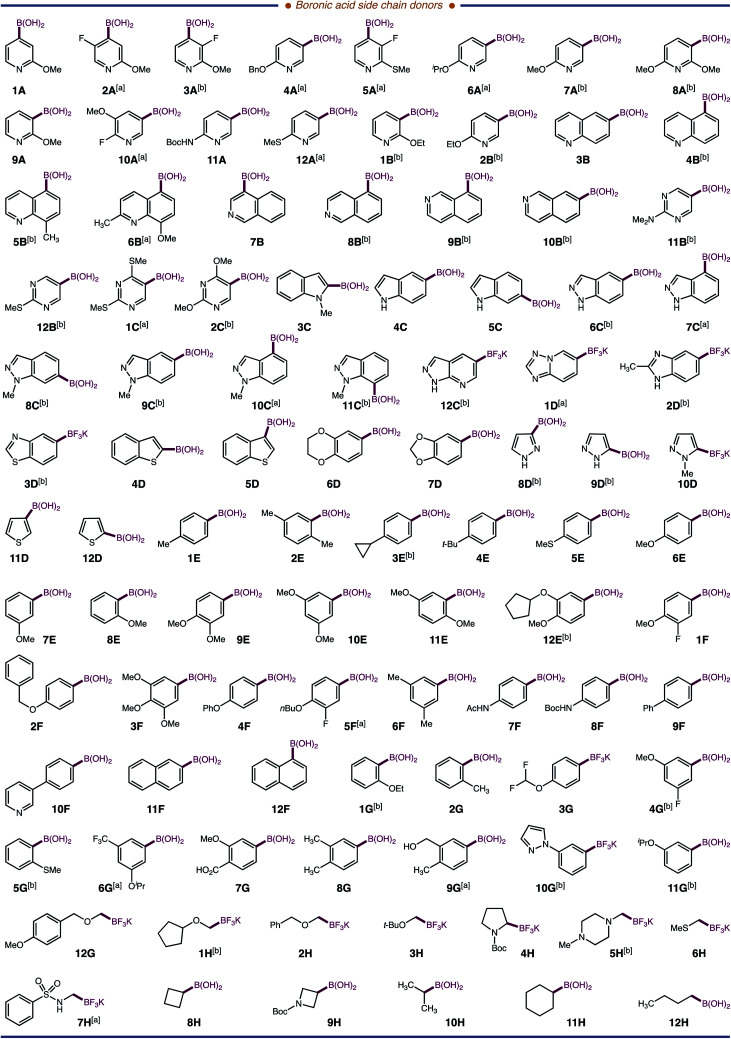
Ninenty-six boronic acids and potassium trifluoroborate salts as precursors for peptide side chains. [a] Forges an entirely new side chain. [b] The amino acid variant has no reported synthetic route to our knowledge.

Next, we examined the application of our system for incorporating NPAAs into a Dha-containing peptide. Our lab recently identified a peptide, Ac-G-P-F-F-NH_2_, that inhibits thrombin, albeit poorly (7.1% inhibition at 80 μM). We reasoned that Ac-G-P-F-F-NH_2_ might be a suitable vehicle to evaluate the proclivity of our system for library generation and for hit-to-lead optimization of peptides with NPAAs, thereby, establishing our proposed HT-*p*SAR platform. A four amino acid peptide, Ac-G-P-**Dha**-F-NH_2_, was prepared as a standard peptide, and quinoline-5-boronic acid **4B** was used as a standard side chain donor. Gratifyingly, the modified peptide Ac-G-P-[**5-Qin**]-F-NH_2_**4B′** was formed in 33% conversion using 5 mol% lumiflavin photocatalyst in aqueous solvent. Performing the reaction with 10 mol% lumiflavin, 5 equiv. of boronic acid, and 10 mM phosphate buffer gave an optimal 75% conversion after 6 hours of irradiation at 3 mM concentration. With this result, we turned our attention to adopting our method for HT applications. To accommodate a HT-workflow, we examined the commercial Lumidox® II 96-well array with 445 nm blue LEDs (28.3 W). This newly engineered system provides comparative results to the irradiation from two 40 W Kessil lamps in our standard reaction. With this system, 96 photochemical reactions can be performed in parallel, which combined with our side chain addition methodology, can transform a single Dha peptide into 96-single NPAA peptide variants. Parallel side chain diversification was first applied to our test peptide Ac-G-P-**Dha**-F-NH_2_. We selected 96 chemically distinct boronic acids (48 heteroaromatic, 35 aromatic, and 13 aliphatic; Fig. 3). All three classes of boronic acids worked well yielding non-proteinogenic and proteinogenic (Met – **6H′** and Leu - **10H′**) amino acid-containing peptides; of which, 15 have entirely new side chains and in the case of 31 other peptides, the NPAA lacks a synthetic route (Fig. 3). Additionally, we observed that the yield of our reaction depended largely on the nucleophilicity of the radicals generated, less nucleophilic radicals resulting in lower conversions of the starting Dha-peptide to modified products.^18^ Our results are distinct from previous reports where only aliphatic systems (Csp^3^-radicals) are shown to be efficient side chain donors into peptides. Our method provides the first example of a Dha-platform that is conducive to incorporating Csp^2^-radicals (aromatic and heteroaromatic side chains) into peptides.

**Scheme 1:**
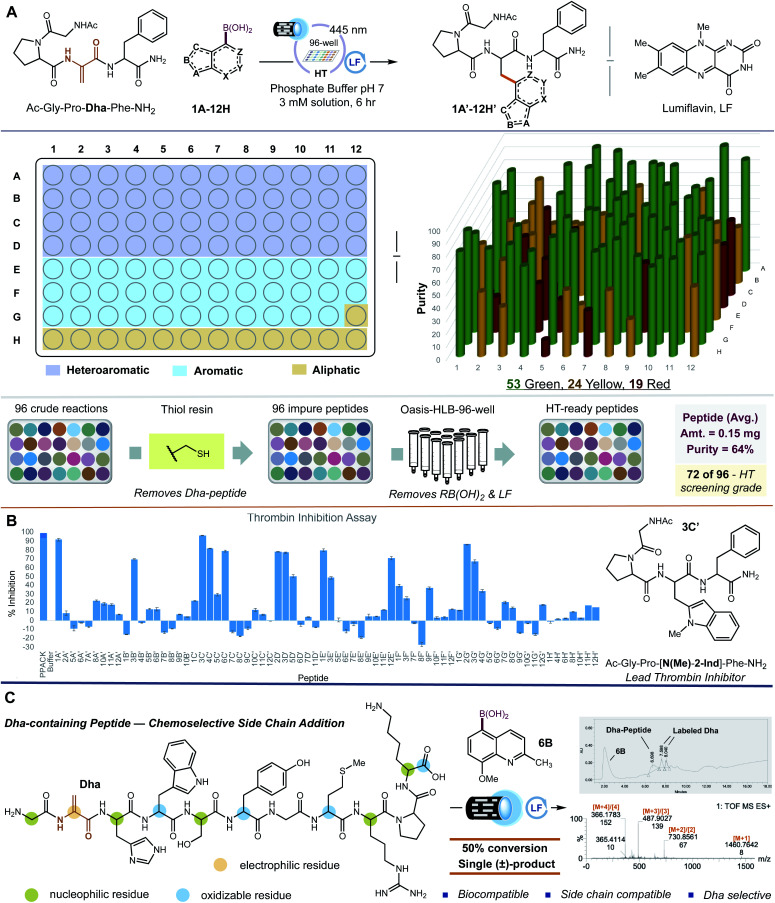
High-throughput peptide structure–activity relationship (HT-*p*SAR). (A) Each well contained 0.0023 mmol of Ac-G-P-**Dha**-F-NH_2_, 0.012 mmol of boronic acid or potassium trifluoroborate salt, 10 mol% lumiflavin, and a 10 mM solution of phosphate buffer at pH 7 [3 mM overall concentration]. The reactions were set up under N_2_ and irradiated with 445 nm blue LED lights for 6 hours. Upon completion, thiol resin was added to each well, stirred overnight, and the mixture was filtered. The solutions were then passed through Waters® Oasis HLB sorbent and analysed by LC-MS/MS. The final products were evaluated based on the criteria of ≥65% purity and ≥0.05 mg. Green = passed both criterion, yellow = passed one criteria, and red = passed neither. See ESI† for full experimental details. (B) The 72 screening grade peptides were tested in a thrombin inhibition assay where 80 μM was administered to the enzyme and its activity measured by fluorescence. Each peptide was run in duplicate, and its standard deviation is represented as error bars. (C) The selectivity of **6B** was assessed using the eleven amino acid peptide, H_2_N-G-**Dha**-H-W-S-Y-G-M-R-P-K-CO_2_H. Only the electrophilic Dha residue was modified.

For practical applications to peptide SAR, we reasoned that the isolation and quantification of our newly derived NPAA-peptides must also be completed in a rapid and highly parallel manner. As a goal, we sought to avoid the use of high-performance liquid chromatography (HPLC), a common but time-intensive technique, for peptide purification. We strived to obtain NPAA-derived peptides in yields ≥ 0.05 mg and purities exceeding 65% – defined as the total amount of correctly modified peptide (as a mixture of diastereomers) in comparison to all other materials at 214 nm – without HPLC purification. Peptides in purities of 50–70% are directly amenable for use in HT-screening experiments, including mutation analysis, hit-to-lead peptide sequence optimization, and protein–protein and receptor–ligand interaction studies.^19^ Following HT-side chain diversification, our 96 peptide samples are quenched with a polymer-bound 2-mercaptoethylamine resin.^20^ The thiol resin sequesters any unreacted Dha-peptide *via* thiol-conjugate addition allowing for simple batch-wise filtration to remove excess Dha-peptide from the samples.^21^ To remove exogenous boronic acid and lumiflavin, we developed a parallel elution technique for peptides based on solid-phase extraction (SPE). The samples are first loaded onto a 96-well plate with each well containing 60 mg of a hydrophilic–lipophilic balanced sorbent (Waters® Oasis HLB). Each well is then washed with 5% NaOH (aq.) to remove unreacted boronic acid. Lumiflavin is removed with 25% ^*i*^PrOH (aq.). Finally, the NPAA-derived peptide is eluted with 50% TFE (aq.) as a mixture of diastereomers. In some cases, the boronic acid coelutes with the NPAA-derived peptide. Liquid-phase extraction of the purified sample with Et_2_O can remove the residual boronic acid in such instances. To quantify our peptide variants, we sought to determine the amount of modified peptide directly from the LC chromatogram of the purified material (the aqueous TFE fraction in our case). Pioneered by Kuipers and Gruppen, the molar extinction coefficients from individual amino acids at 214 nm can be summed to obtain the net extinction coefficient of the intact peptide (relative standard deviation of ∼3%).^22^ Using the net extinction coefficient of the peptide, the LC chromatogram, and a variant of Beer–Lambert's law; the amount of each peptide can be readily deduced.^22^ To implement this quantification protocol for NPAA-containing peptides, we first measured the extinction coefficients for a variety of NPAAs (not previously reported) and proteinogenic amino acids, particularly those found in our test peptide, by UV-Vis spectroscopy (Fig. S6 and S7†). Our experimental values were in good agreement with those previously reported for proteinogenic amino acids. Extinction coefficients for non-proteinogenic residues are, therefore, reported in high confidence. Unfortunately, not all 96 NPAAs were available or accessible for UV-Vis determination. For a series of phenylalanine (Phe) derivatives – *p*-OMe, *p*-^*t*^Bu, *p*-Me, and *p*-F – we found that the average extinction coefficient of the NPAAs matched well with the unmodified Phe. Thus, we used the extinction coefficients of the parent amino acids (*i.e.*, 3-pyridyl-, 2-quinoyl-, *N*-Me-7-indazolyl-, 3-benzothienyl-, 2-thienyl-, 2-naphthyl-, and 3-cyclohexylalanine) at 214 nm to estimate corresponding NPAA analogs. Applying our isolation and quantification protocols to our peptides, we determined that fifty-three of the 96 peptides were obtained in yields ≥ 0.05 mg and in purities ≥ 65% (Scheme 1A; green colored substrates). Twenty-four of the 96 peptides lacked either sufficient purity or yield according to our predetermined goals (Scheme 1A; yellow colored substrates). Nineteen of the 96 peptides failed to meet either of our criterion (Scheme 1A; red colored substrates). On average, peptides were isolated in yields of 0.15 mg (11.7%) and purities of 64% without HPLC purification. While 55% of the peptides satisfied our criterion, peptides of 50–70% purity are generally sufficient for high-throughput experiments.^19^ Therefore, 72 of our 96 peptides (75%) are screening grade.

To complete the assessment of our HT-*p*SAR platform, we examined the ability of our peptide variants (those considered screening grade; ≥50% purity) to inhibit thrombin, a key protein necessary for blood coagulation.^23^ (It is important to note that using mixtures of peptide diastereomers is a well-established approach when mining peptide libraries for bioactive leads in a HT-assay. Therefore, our peptide libraries are suitable for early-stage peptide (1) lead generation, (2) structural refinement, and (3) biochemical assessment).^24^ A venerable target to control bleeding,^25^ inhibiting thrombin has also become a popular strategy to address a myriad of health care associated diseases, including Alzheimer's disease,^26^ Necrotizing enterocolitis (NEC) in premature infants,^27^ non-small cell lung cancer (preventing vasculogenic mimicry formation) and other cancers (counteracting immune evasion),^28,29^ and COVID-19 (controlling coagulopathy).^30^ In a standard thrombin inhibition assay (purchased from Sigma-Aldrich), we found that twelve of our peptides (administered at 80 μM and performed in duplicate) reduced the cleavage of a fluorogenic peptide ligand of thrombin (PPACK) by more than 50% (Scheme 1B). Peptides having indole-like NPAAs were the most effective at inhibiting thrombin **3C′**, **4C′**, **6C′**, **2D′**, and **3D′** (77–97%); of which, **3C′** was the optimal inhibitor (97% inhibition). Interestingly, these peptides were effective despite their lack of a cationic functional group (*i.e.*, a guanidine residue), a hallmark of many thrombin inhibitors that better enables the drug to engage the active site of thrombin.^31^ To verify the considerable effect of replacing residue F3 in Ac-G-P-**F**-F-NH_2_ with indole-like NPAAs to inhibit thrombin, we resynthesized **3C′**, separated the diastereomers by HPLC (>95% purity), and measured the percent thrombin inhibition for each diastereomer. Both (l)-*N*-methyl-2-indole 3C′ and (d)-*N*-methyl-2-indole 3C′ were effective, 67% and 30% respectively (note: (d)-*N*-methyl-2-indole 3C′ was grossly insoluble in water and DMSO). The ability of each diastereomer to inhibit thrombin is consistent with the results found in our 96-well screen. Thus, our completely parallel HT-*p*SAR platform can identify trustworthy NPAA replacements for traditional amino acids that improve upon the activity of ordinary peptides.

Exploring the utility of our side chain diversification method for other peptides beyond Ac-G-P-**Dha**-F-NH_2_, we examined H_2_N-G-**Dha**-H-W-S-Y-G-M-R-P-K-CO_2_H, a Dha-containing peptide which also contains common amino acids found in peptides and proteins that would most likely interfere with our photochemical transformation (Scheme 1C). Flavin photocatalysts are known to oxidatively modify *C*-terminal amino acids *via* decarboxylation,^32^ as well as directly oxidize tyrosine (Y),^33^ tryptophan (W),^33,34^ histidine (H),^34^ and methionine (M)^35^ residues. Moreover, radicals are known to modify histidine, tyrosine, and tryptophan amino acids.^36^ And finally, nucleophilic residues including histidine,^37,38^ serine (S),^39^ and lysine (K)^40,41^ are known to coordinate to boronic acids. Subjecting the 11-mer peptide to our optimized reaction conditions afforded 50% of a mono-labeled product in the presence of (8-methoxy-2-methylquinolin-5-yl)boronic acid **6B** (Scheme 1C). LC-MS/MS analysis revealed that the electrophilic Dha residue was the site for modification. No methionine or aromatic (H, Y, W) oxidation was found, and *C*-terminal decarboxylation was likewise not observed. The chemoselectivity of our reaction is remarkable given the propensity for flavin photocatalysts and *C*-centered radicals to modify these amino acids. Thus, our methodology is applicable to unprotected peptides containing common amino acid side chains.

## Conclusions

We have developed a brand new platform (HT-*p*SAR) that permits up to 96 single-amino acid NPAA peptide variants to be prepared, purified, and tested for bioactivity in parallel, from a single Dha-containing peptide precursor. We demonstrate that our platform can incorporate established and, heretofore, unknown NPAAs (heteroaromatic, aromatic, and aliphatic) and is highly specific for Dha residues. Finally, we showcase the utility of HT-*p*SAR to accelerate structure–activity relationship studies of peptides and to transform ordinary peptides like Ac-G-P-F-F-NH_2_ into lead therapeutic candidates.

## Data availability

The ESI† includes experimental optimizations and procedures, extinctions coefficients, thrombin inhibition data, characterization data, NMR data, and LC-MS/MS data of peptides.

## Author contributions

J. R. I. optimized the system and performed the 96-well plate reactions, purifications, and analyses. M. C. performed and analyzed the reaction on the 11-mer chemoselectivity peptide. J. R. I. and M. C. isolated the amino acid products. J. R. I., M. C., and S. B. wrote and revised the manuscript.

## Conflicts of interest

There are no conflicts to declare.

## Supplementary Material

SC-012-D1SC02562G-s001
