# Supplementary material for: Combining flavin photocatalysis with parallel synthesis: a general platform to optimize peptides with non-proteinogenic amino acids
Source: Chem Sci. 2021 Jun 30;12(29):10083–91. doi: 10.1039/d1sc02562g (PMC8317666; doi:10.1039/d1sc02562g)
Supplement: SC-012-D1SC02562G-s001 [file SC-012-D1SC02562G-s001.pdf]

Electronic Supplementary Material (ESI) for Chemical Science.
This journal is © The Royal Society of Chemistry 2021

Supporting Information

Combining Flavin Photocatalysis with Parallel Synthesis: A General Platform to Optimize Peptides with Non-Proteinogenic Amino Acids

Jacob R. Immel, Maheshwerreddy Chilamari, and Steven Bloom*

Table of Contents

General Information 2
Reaction Optimization 3
- **Effect of photocatalyst identity on the amino acid reaction** 3
- **Effect of cosolvent on the amino acid reaction** 4
- **Effect of buffer identity on the amino acid reaction** 6
- **Effect of concentration, lumiflavin loading, and boronic acid equivalents on the amino acid reaction** 6
- **Reaction optimization for the peptide** 8

Experimental Procedures 9
- **Picture of reaction setups** 9
- **General procedure for amino acid synthesis** 9
- **General procedure for setting up the 96-well reactions** 9
- **General procedure for purifying the 96-well reactions** 10
- **General procedure for addition into the selectivity peptide** 10
- **Synthesis of starting materials and other compounds** 10

Extinction Coefficients 16
- **Measured extinction coefficients of proteinogenic amino acids** 16
- **Measured extinction coefficients of non-proteinogenic amino acids** 16
- **Measured extinction coefficients of substituted phenylalanine derivatives** 17
- **Assumptions to determine yields** 17
- **Example calculation to determine yield** 17

Thrombin Inhibition Studies 19
Analytical Data of Products 21
- **Amino Acids** 21

Peptides 23
3C' Isomers 24
NMR Spectra 27
Peptide Selectivity 38
Plate Data 41
References 236

General Information

Unless stated otherwise, all reactions were performed under an atmosphere of N_2 with magnetic stirring. Materials received from commercial suppliers were used directly without further purification. Boronic acids and potassium trifluoroborates were purchased from Sigma-Aldrich, Alfa Aesar, Matrix Scientific, Frontier Scientific, A.K. Scientific, Combi-Blocks, and Oakwood Chemicals. Methyl 2-Acetamidoacrylate was purchased from Combi-Blocks and Oakwood Chemicals. Fmoc-protected amino acids were purchased from Chem-Impex. The organic solvents (reagent grade) used for reaction optimization and purification were purchased from Sigma Aldrich and Fisher Scientific. Aqueous buffers were freshly prepared using Millipore Grade I water (Resistivity > 5 MΩ cm, Conductivity < 0.2 μS/cm, TOC <30 ppb) and the pH adjusted using a Mettler Toledo FiveEasy pH meter. Thin-layer chromatography (TLC) was performed on silica gel coated aluminum TLC plates (Merck, TLC Silica gel 60 F_{254}) and visualized using a UV lamp (254 nm) in combination with KMnO4, Iodine, and Curcumin stains. The amino acid reactions were performed with Blue LED lights from Kessil (PR160L, 440 nm, 40 W). The peptide reactions were performed with the Lumidox® Generation II Controller and the 96-well LED array (445 nm, 295 mW per well) with a Lens Mat Active Cooling Base from Analytical Sales and Services Inc. The amino acid reactions were stirred on a Corning PC-620D stir plate at 1150 rpm. The plate reactions were stirred with a VP 710-C5 from V&P Scientific Inc. The products were purified by flash column chromatography on an automated Buchi Reveleris^{R} X2 instrument or on a Isolera 1 Biotage® instrument. ^{1}H NMR, ^{13}C NMR, and ^{19}F NMR spectra were recorded on a Bruker Ascend^{TM} 400 NMR spectrometer. ^{13}C NMR spectra were also recorded on a Bruker Ultrashield^{TM} Plus500 NMR spectrometer for some compounds. ^{1}H NMR and ^{13}C NMR spectra were referenced to $CDCl_3$ (7.26 ppm and 77.16 ppm) or D_2O (4.90 ppm). Peak multiplicities are designated by the following abbreviations: s, singlet; d, doublet; t, triplet; p, pentet, q, quartet; hept, heptet m, multiplet; dd, doublet of doublet; dt, doublet of triplet; and ddd, doublet of doublet of doublet. NMR Spectra were recorded at 298 K. Electrospray ionization spectra were acquired on a LCT Premier (Waters Corp.) time of flight mass spectrometer (HRMS). UV spectrum for peptides were determined on a Waters Acquity UPLC H-Class, and LC-MS/MS data was acquired on a Quatro Ultima (Waters Corp.) triple quadrupole mass analyzer with an electrospray ion source. Extinction coefficients were determined on Varian Cary 100 Bio UV-Vis Spectrophotometer.

Reaction Optimization

The reaction was optimized using 2-methoxypyridine-4-boronic acid **1A** at physiological conditions (pH 6-8). Methyl 2-acetamidoacrylate was used as a surrogate for an internal dehydroalanine. Our optimization primarily focused on finding a suitable photocatalyst, organic cosolvent, buffer, and solution concentration. A summary of our optimization results is presented in Supplementary Tables 1-5 below.

Effect of photocatalyst identity on the amino acid reaction

Procedure: To a 1-dram vial charged with a stir bar, methyl 2-acetamidoacrylate (5 mg, 0.035 mmol, 1 equiv.), 2-methoxypyridine-4-boronic acid **1A** (6.42 mg, 0.042 mmol, 1.2 equiv.), photocatalyst (0.0017 mmol, 0.05 equiv.), 525 µL of *N*,*N*-dimethylformamide (N_2 sparged for 5 minutes), 350 µL of a 0.1 M solution of phosphate buffer pH 7 (N_2 sparged for 5 minutes), and 2.625 mL ddH_2O (N_2 sparged for 5 minutes) were added. Then, the resulting solution was degassed by sparging with nitrogen for 5 min, parafilmed, and placed 5 cm away from two 40 W blue LED lamps. The sample was irradiated for 15 hours with stirring at 1150 rpm and fan cooling. After 15 hours, the sample was removed and extracted using ethyl acetate and washed with sodium bicarbonate (saturated aq.) and brine (saturated aq.) solutions. The organic layers were combined and concentrated by rotary evaporation followed by high vacuum. A crude ^{1}H NMR yield was obtained by the addition of dibromomethane as an internal standard (1 equiv., 0.035 mmol, 4.93 ppm).

Table S1. Effect of photocatalyst identity on the reaction

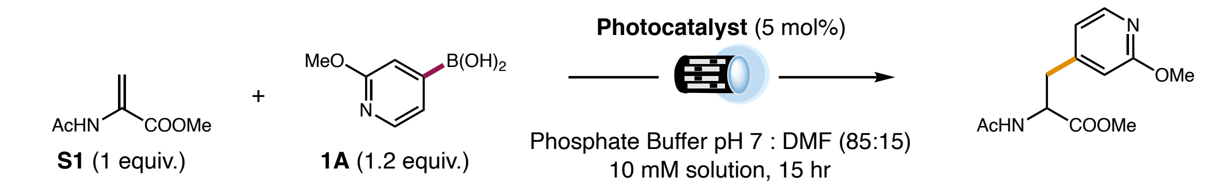

Entry	Photocatalyst identity	% Yield[a]
1	Eosin Y	0%
2	Tris(2,2′-bipyridyl)dichlororuthenium(II) hexahydrate	0%
3	(Ir[dF(CF_3)ppy]$_2$(dtbpy)) hexafluorophosphate	0%
4	9-Mesityl-10-methylacridinium tetrafluoroborate	<1%
5	Riboflavin	1%
6	Lumichrome	2%
7	Riboflavin 5'-monophosphate sodium salt	3%
8	Riboflavin tetrabutyrate	4%
**9**	**Lumiflavin**	**8%**

[a] % Yield determined by ^{1}H NMR spectra using dibromomethane (1 equiv.) as an internal standard.

Effect of cosolvent on the amino acid reaction

Procedure: To a 1-dram vial charged with a stir bar, methyl 2-acetamidoacrylate (5 mg, 0.035 mmol, 1 equiv.), 2-methoxypyridine-4-boronic acid **1A** (6.42 mg, 0.042 mmol, 1.2 equiv.), lumiflavin (0.45 mg, 0.0017 mmol, 0.05 equiv.), 525 μL of cosolvent (N_2 sparged for 5 minutes), 350 μL of a 0.1 M solution of phosphate buffer pH 7 (N_2 sparged for 5 minutes), and 2.625 mL ddH_2O (N_2 sparged for 5 minutes) were added. Then, the resulting solution was degassed by sparging with nitrogen for 5 min, parafilmed, and placed 5 cm away from two 40 W blue LED lamps. The sample was irradiated for 15 hours with stirring at 1150 rpm and fan cooling. After 15 hours, the sample was removed and extracted using ethyl acetate and washed with sodium bicarbonate (saturated aq.) and brine (saturated aq.) solutions. The organic layers were combined and concentrated by rotary evaporation followed by high vacuum. A crude ^{1}H NMR yield was obtained by the addition of dibromomethane as an internal standard (1 equiv., 0.035 mmol, 4.93 ppm).

Table S2. Effect of cosolvent on the reaction

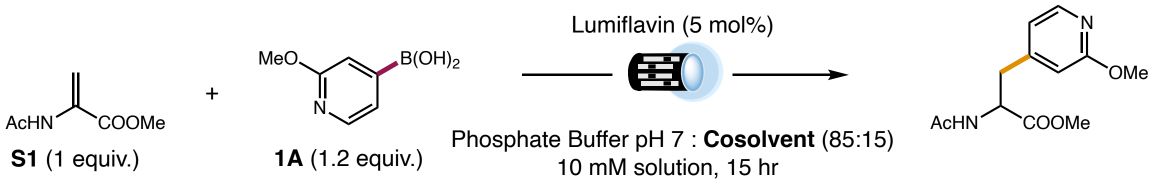

Entry	Cosolvent Identity	% Yield[a]
1	Cyclohexyl-*N*-methylpyrrolidine	<1%
2	Pyridine	2%
3	*N*-Methyl-2-Pyrrolidone	2%
4	Tetrahydrofuran	2%
5	Isopropanol	4%
6	*N*,*N*-Dimethylacetamide	5%
7	1,2-Dimethoxyethane	5%
8	Ethanol	8%
9	Diethylene glycol dimethyl ether	8%
10	Dimethylsulfoxide	9%
11	Acetonitrile	9%
12	Acetone	10%
13	Dioxane	10%
14	Sulfolane	10%
15	Propylene carbonate	10%
16	Methyl Ethyl Ketone	11%
17	Methanol	11%
18	*tert*-Butanol	12%
19	Methyl Acetate	12%
20	Glycerol	16%
21	Hexafluoro-2-propanol	17%
22	2,2,2-Trifluoroethanol	20%
**23**	**Water (no cosolvent)**	**28%**

[a] % Yield determined by ^{1}H NMR spectra using dibromomethane (1 equiv.) as an internal standard.

Effect of buffer identity on the amino acid reaction

Procedure: To a 1-dram vial charged with a stir bar, methyl 2-acetamidoacrylate (5 mg, 0.035 mmol, 1 equiv.), 2-methoxypyridine-4-boronic acid **1A** (6.42 mg, 0.042 mmol, 1.2 equiv.), lumiflavin (0.45 mg, 0.0017 mmol, 0.05 equiv.), buffer solution (N_2 sparged for 5 minutes), and 3.15 mL ddH_2O (N_2 sparged for 5 minutes) were added. Then, the resulting solution was degassed by sparging with nitrogen for 5 min, parafilmed, and placed 5 cm away from two 40 W blue LED lamps. The sample was irradiated for 15 hours with stirring at 1150 rpm and fan cooling. After 15 hours, the sample was removed and extracted using ethyl acetate and washed with sodium bicarbonate (saturated aq.) and brine (saturated aq.) solutions. The organic layers were combined and concentrated by rotary evaporation followed by high vacuum. A crude ^{1}H NMR yield was obtained by the addition of dibromomethane as an internal standard (1 equiv., 0.035 mmol, 4.93 ppm).

Table S3. Effect of buffer identity on the reaction

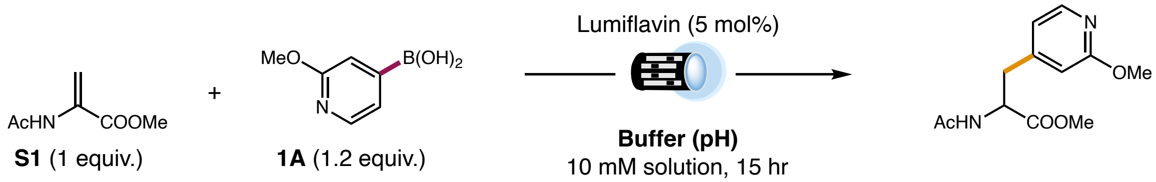

Entry	Buffer Identity (pH)	Buffer concentration	% Yield[a]
1	Ammonium carbonate (8.4)	10 mM	8%
2	Sodium bicarbonate (8.2)	10 mM	10%
3	MES (6.5)	10 mM	11%
4	HEPES (7)	10 mM	26%
5	Phosphate (8)	10 mM	13%
**6**	**Phosphate (7)**	**10 mM**	**28%**
7	Phosphate (7)	25 mM	21%
8	Phosphate (7)	50 mM	15%
9	Phosphate (7)	100 mM	19%

[a] % Yield determined by ^{1}H NMR spectra using dibromomethane (1 equiv.) as an internal standard.

Effect of concentration, lumiflavin loading, and boronic acid equivalents on the amino acid reaction

Procedure: To a 1-dram vial charged with a stir bar, methyl 2-acetamidoacrylate (5 mg, 0.035 mmol, 1 equiv.), 2-methoxypyridine-4-boronic acid **1A**, lumiflavin, phosphate buffer pH 7 (N_2 sparged for 5 minutes), and ddH_2O (N_2 sparged for 5 minutes) were added. Then, the resulting solution was degassed by sparging with nitrogen for 5 min, parafilmed, and placed 5 cm away from two 40 W blue LED lamps. The sample was irradiated for 15 hours with stirring at 1150 rpm

and fan cooling. After 15 hours, the sample was removed and extracted using ethyl acetate and washed with sodium bicarbonate (saturated aq.) and brine (saturated aq.) solutions. The organic layers were combined and concentrated by rotary evaporation followed by high vacuum. A crude ^{1}H NMR yield was obtained by the addition of dibromomethane as an internal standard (1 equiv., 0.035 mmol, 4.93 ppm).

Table S4. Effect of concentration, lumiflavin loading, and boronic acid equivalents on the reaction

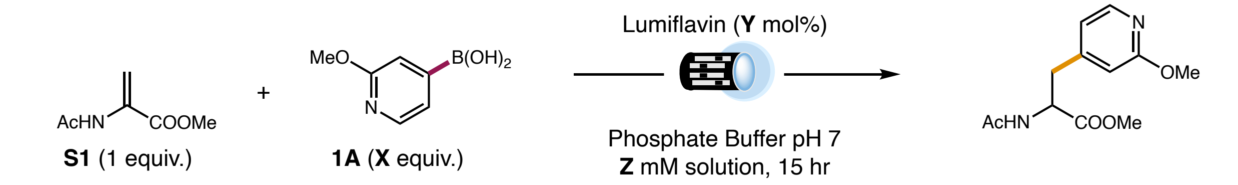

Entry	Boronic acid	mol% Lumiflavin	Concentration of solution	% Yield[a]
1	1.2 equiv.	5	1 mM	2%
2	1.2 equiv.	5	5 mM	27%
3	1.2 equiv.	5	10 mM	29%
4	1.2 equiv.	5	15 mM	15%
5	3 equiv.	5	10 mM	37%
6	3 equiv.	10	10 mM	39%
**7**	**3 equiv.**	**10**	**10 mM**	**[b]55% ([c]49%)**

[a] % Yield determined by ^{1}H NMR spectra using dibromomethane (1 equiv.) as an internal standard. [b] 50 mg scale of the acrylate. [c] Isolated yield.

Procedure for 50 mg scale: To a 100-mL round-bottom flask charged with a stir bar, methyl 2-acetamidoacrylate (50 mg, 0.35 mmol, 1 equiv.), 2-methoxypyridine-4-boronic acid **1A** (160.6 mg, 1.05 mmol, 3 equiv.), lumiflavin (8.97 mg, 0.035 mmol, 0.10 equiv.), 3.5 mL of a 0.1 M solution of phosphate buffer pH 7 (N_2 sparged for 5 minutes), and 31.5 mL ddH_2O (N_2 sparged for 5 minutes) were added. Then, the resulting solution was degassed by sparging with nitrogen for 5 min, parafilmed, and placed 5 cm away from two 40 W blue LED lamps. The sample was irradiated for 15 hours with stirring at 550 rpm and fan cooling. After 15 hours, 10 equiv. of L-Cysteine and 20 mL of sodium bicarbonate (saturated aq.) were added to the stirring solution at room temperature. After being stirred for 2 hours, the sample was extracted using ethyl acetate and washed with brine (saturated aq.). The organic layers were combined and concentrated by rotary evaporation followed by high vacuum. A crude ^{1}H NMR yield was obtained by the addition of nitromethane as an internal standard (1 equiv., 0.35 mmol, 4.33 ppm). The product was then purified by reverse phase flash chromatography using acetonitrile:water to yield 43.4 mg (49.2% isolated yield) of pure product.

Reaction optimization for the peptide

Procedure: To a 1 mL clear glass shell vial (purchased from Analytical Sales & Services Inc.) charged with a small stir bar, Ac-Gly-Pro-**Dha**-Phe-NH_2 (1 mg, 2.33 μmol, 1 equiv.), quinoline-5-boronic acid **4B**, lumiflavin, phosphate buffer pH 7 (N_2 sparged for 5 minutes), and ddH_2O (N_2 sparged for 5 minutes) were added. The vial was purged under N_2 for 3 minutes, parafilmed, and irradiated with blue light with stirring at 1150 rpm on the Corning PC-620D stir plate or at ~700 rpm on the VP 710-C5 for the specified amount of time. After that time, the sample was filtered and subjected to UPLC/MS analysis.

Table S5. Optimization of the peptide reaction

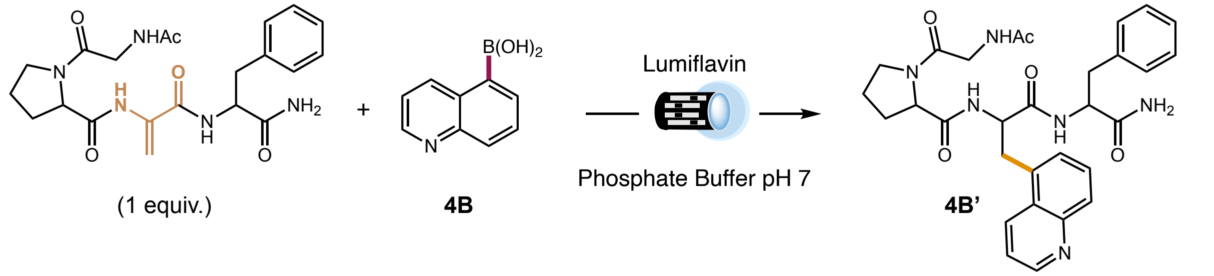

Entry	Lumiflavin	Light source	Boronic acid	Conc.	Time	% Conversion.[a]
1	5 mol%	Kessil	1 equiv.	10 mM	15 hr	19%
2	5 mol%	Kessil	3 equiv.	10 mM	15 hr	33%
3	5 mol%	Kessil	5 equiv.	10 mM	15 hr	37%
4	5 mol%	Kessil	10 equiv.	10 mM	15 hr	40%
5	5 mol%	Kessil	5 equiv.	5 mM	15 hr	36%
6	5 mol%	Kessil	5 equiv.	3 mM	15 hr	60%
7	5 mol%	Lumidox® Gen. II	5 equiv.	10 mM	15 hr	49%
8	5 mol%	Lumidox® Gen. II	5 equiv.	3 mM	8 hr	56%
9	10 mol%	Lumidox® Gen. II	5 equiv.	3 mM	8 hr	70%
**10**	**10 mol%**	**Lumidox® Gen. II**	**5 equiv.**	**3 mM**	**6 hr**	**75%**

[a]% Product determined by integrating the product peaks on LC as compared to all other peptide-related peaks.

Experimental Procedures

Picture of reaction setups

(A) (B)

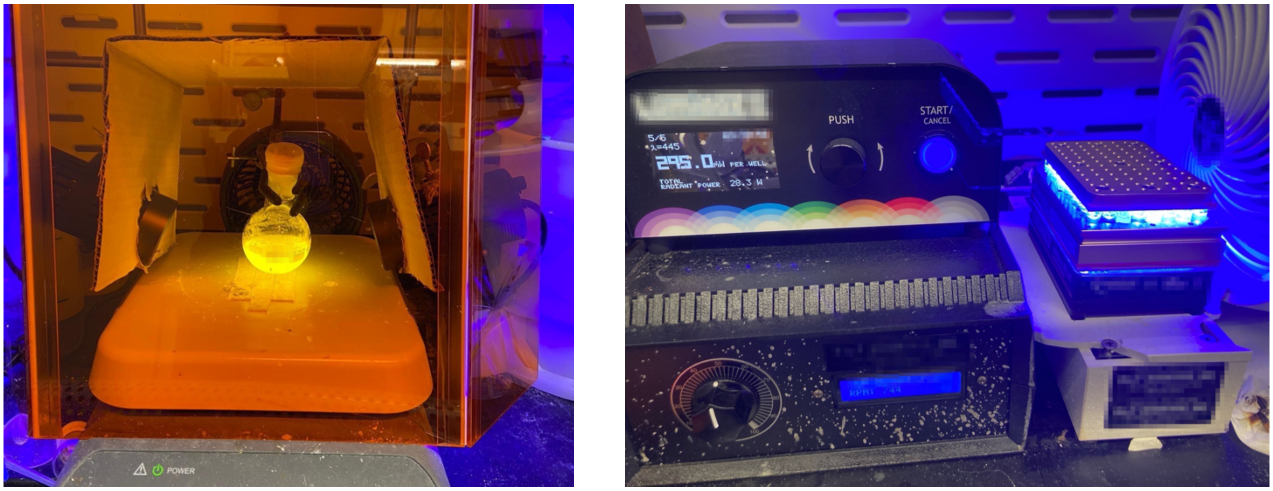

Figure S1. Reaction setup photographs. (A) Amino acid reactions. (B) 96-well plate reactions.

General procedure for amino acid synthesis

To a 100-mL round-bottom flask charged with a stir bar, methyl 2-acetamidoacrylate (50 mg, 0.35 mmol, 1 equiv.), boronic acid (1.05 mmol, 3 equiv.), lumiflavin (8.97 mg, 0.035 mmol, 0.10 equiv.), 3.5 mL of a 0.1 M solution of phosphate buffer pH 7 (N_2 sparged for 5 minutes), and 31.5 mL ddH_2O (N_2 sparged for 5 minutes) were added. Then, the resulting solution was degassed by sparging with nitrogen for 5 min, parafilmed, and placed 5 cm away from two 40 W blue LED lamps. The sample was irradiated for 15 hours with stirring at 550 rpm and fan cooling. After 15 hours, 10 equiv. of L-Cysteine and 20 mL of sodium bicarbonate (saturated aq.) were added to the stirring solution at room temperature. After being stirred for 2 hours, the sample was extracted using ethyl acetate and washed with brine (saturated aq.). The organic layers were combined and concentrated by rotary evaporation followed by high vacuum. A crude ^{1}H NMR yield was obtained by the addition of nitromethane as an internal standard (1 equiv., 0.35 mmol, 4.33 ppm). The product was then purified by reverse phase flash chromatography using acetonitrile:water.

General procedure for setting up the 96-well reactions

To 96 individual 1 mL clear glass shell vials (purchased from Analytical Sales & Services Inc.) each charged with a small stir bar, 96 different boronic acids or potassium trifluoroborates (11.65 µmol, 5 equiv.) were added. Stock solutions of peptide (100 mg of Ac-G-P-**Dha**-F-NH_2 in 50 mL of ddH_2O), phosphate buffer pH 7 (10 mL of a 0.1 M solution), and lumiflavin (9 mg in 30 mL of ddH_2O) were purged under N_2 for 5 minutes each. The 96 vials containing boronic acids and potassium trifluoroborates, the stock solutions, 100-1000 µL and 20-200 µL channel micropipettes (can use multichannel), and a Philips head screwdriver were placed in a N_2 glove bag (purchased from Cole-Palmer). The glove bag was filled once with N_2, emptied, refilled, and sealed. To each of the 96 vials was added 500 µL of the peptide solution, 77 µL of the buffered solution, and 200 µL of the lumiflavin solution. The screwdriver was used to screw down the cap onto the vials. The

vials were then removed from the glove bag and placed on the Lumidox® 96-well LED array (445 nm) and illuminated with stirring at ~700 rpm for 6 hours.

General procedure for purifying the 96-well reactions

Upon completion of the reaction, the cap was removed, and the stirring was stopped. From a 0.24 mM stock solution of TCEP in ddH$_2$O, 100 µL were added to each vial. 10 equiv. of 2-mercaptoethylamine, polymer-bound resin was also added to each vial and the mixture stirred at ~700 rpm overnight at room temperature to remove any unreacted Dha-containing peptide. The vials were then filtered using a 96-well filter plate. Utilizing Waters Oasis HLB 96-well Plate (60 mg sorbent per well), the sorbent was first washed and filtered with 1 mL of methanol and 1 mL of water. The sample solutions were then loaded onto the sorbent and filtered. The boronic acid and potassium trifluoroborates were removed by filtering 1 mL of a 5% NaOH solution through each well. The lumiflavin was removed by carefully filtering (small amount of vacuum) 800 µL of a 25% isopropanol solution in water. Finally, the products were eluted with 1 mL of a 50% trifluoroethanol solution in water. The products were then submitted to a UPLC for analysis of purity and yield.

In instances where exogenous boronic acid remained in the eluents, diethyl ether extraction was performed. The samples of interest were first lyophilized to powder. Then, 500 µL of diethyl ether was added to each sample followed by 500 µL of ddH$_2$O. The aqueous solution was extracted in a glass pipette. The products were then diluted with another 500 µL of ddH$_2$O and submitted to a UPLC for analysis of purity and yield.

General procedure for addition into the selectivity peptide

To a 1 mL clear glass shell vial (purchased from Analytical Sales & Services Inc.) charged with a small stir bar, H$_2$N-Gly-**Dha**-His-Trp-Ser-Tyr-Gly-Met-Arg-Pro-Lys-CO$_2$H (1 mg, 0.77 µmol, 1 equiv.), 2-methyl-8-methoxyquinoline-5-boronic acid **6B** (0.5 mg, 2.31 µmol 3 equiv.), lumiflavin (0.020 mg, 0.077 µmol, 0.1 equiv.), 25.6 µL 0.1 M phosphate buffer pH 7 (N$_2$ sparged for 5 minutes), and 230.4 µL ddH$_2$O (N$_2$ sparged for 5 minutes) were added. The vial was purged under N$_2$ for 3 minutes, parafilmed, and irradiated with blue light with stirring at ~700 rpm on the VP 710-C5 for 6 hours. After 6 hours, the sample was filtered and subjected to UPLC/MS analysis.

Synthesis of starting materials and other compounds

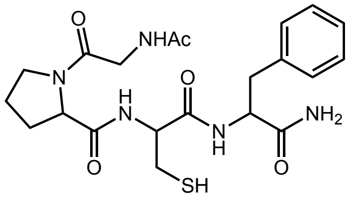

Ac-Gly-Pro-Cys-Phe-NH$_2$ was synthesized by solid phase peptide synthesis using Fmoc-protected amino acids on rink amid resin, cleaved with 95:2.5:2.5 TFA:TIPS:H$_2$O solution, and purified by reverse phase flash chromatography.

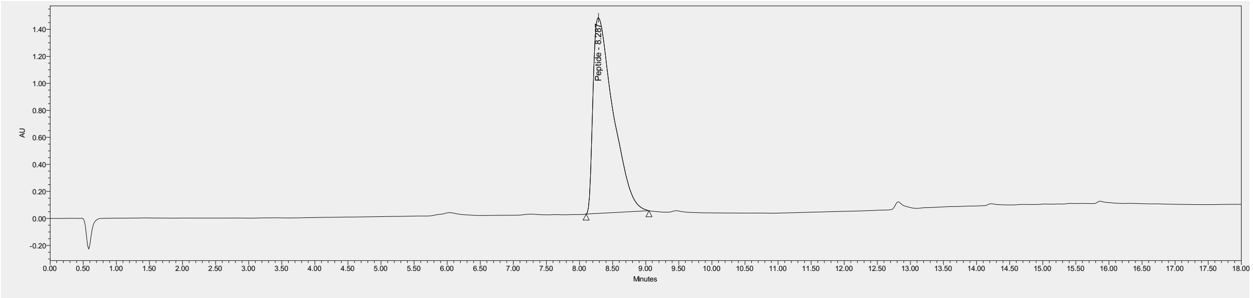

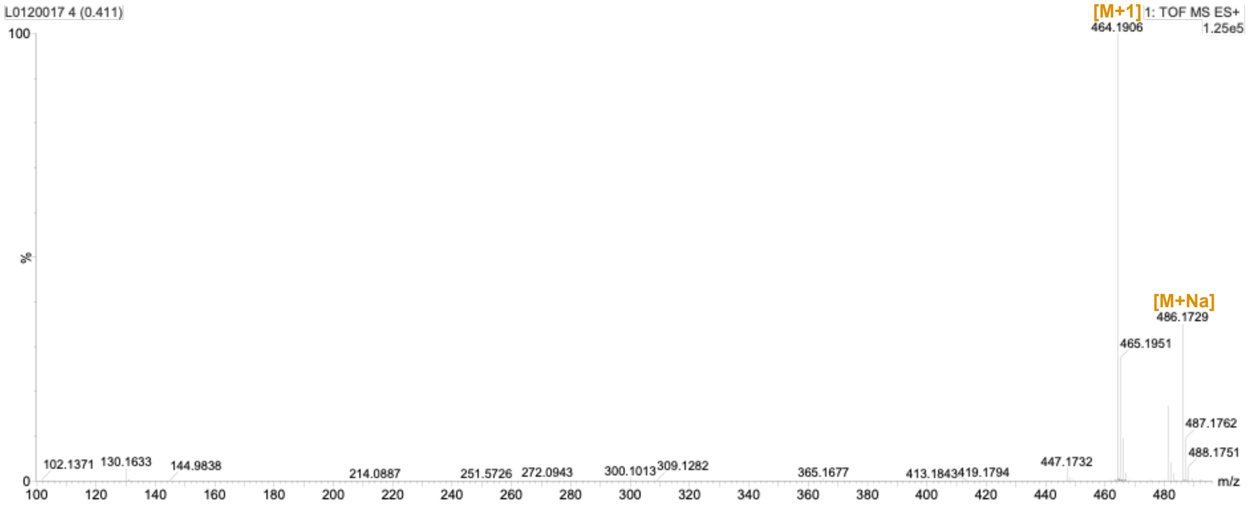

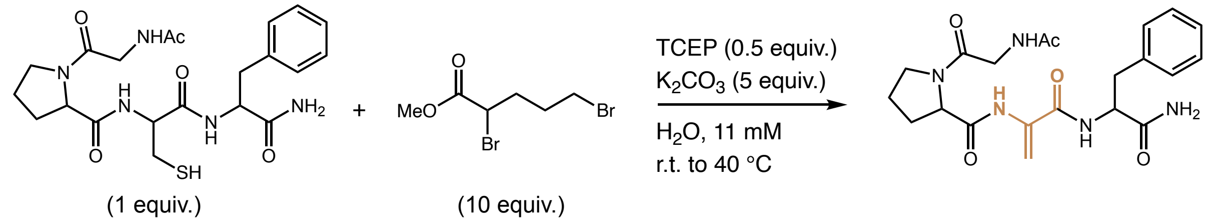

Ac-Gly-Pro-Dha-Phe-NH$_2$: To a 50 mL round-bottom flask charged with a stir bar, Ac-Gly-Pro-Cys-Phe-NH$_2$ (100 mg, 0.22 mmol, 1 equiv.), TCEP (31.5 mg, 0.11 mmol, 0.5 equiv.), and 20 mL of ddH$_2$O were added. The solution was stirred for 1 hour at room temperature; after which, methyl 2,5-dibromovalerate (591.0 mg, 2.2 mmol, 10 equiv.) and potassium carbonate (149 mg, 1.08 mmol, 5 equiv.) were added. The heterogenous mixture was stirred for another hour at room temperature before being heating to 40 °C for 3 hour. The reaction was monitored by UPLC/MS. Upon completion, the mixture was washed with 20 mL of ethyl acetate to remove excess methyl 2,5-dibromovalerate before being concentrated under reduced pressure. The residual solid was then redissolved in minimal methanol, filtered to remove impure solid, and purified by normal phase flash chromatography (0 – 10% methanol in methylene chloride, product elutes at 10% methanol in methylene chloride. Isolated yield = 51.2% (52.2 mg).

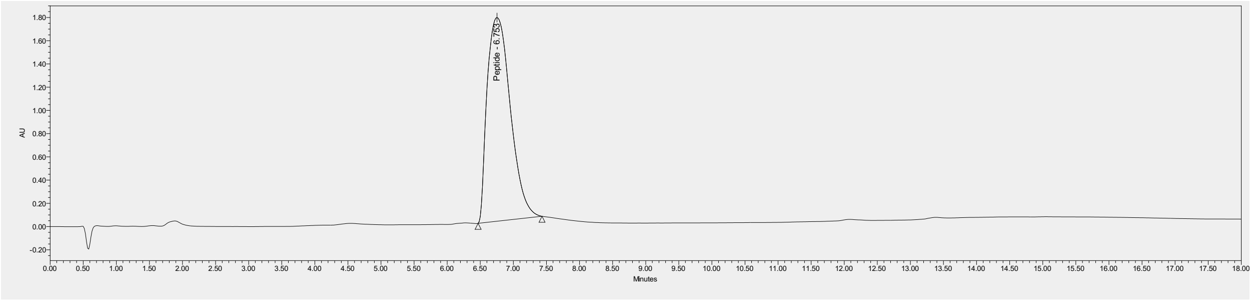

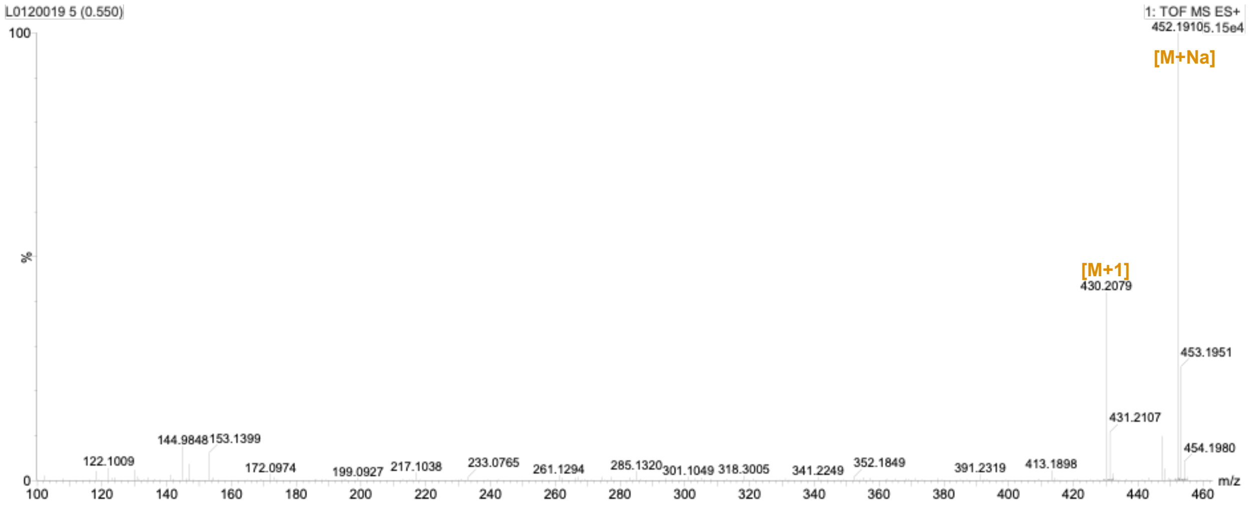

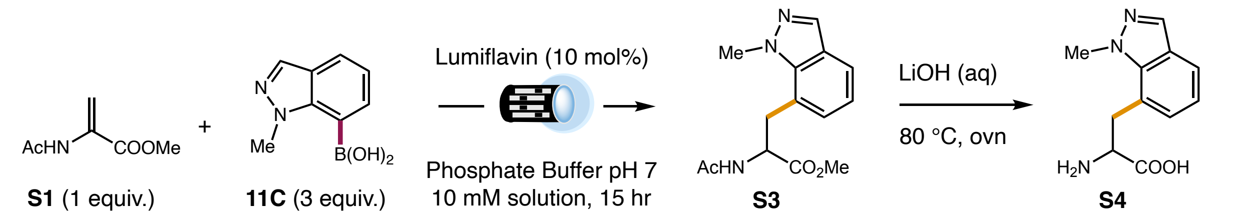

Methyl 2-acetamido-3-(1-methyl-1*H*-indazol-7-yl)propanoate (S3): To a 100-mL round-bottom flask charged with a stir bar, methyl 2-acetamidoacrylate (50 mg, 0.35 mmol, 1 equiv.), 1-methyl-1H-indazole-7-boronic acid **11C** (184.8 mg, 1.05 mmol, 3 equiv.), lumiflavin (8.97 mg, 0.035 mmol, 0.10 equiv.), 3.5 mL of a 0.1 M solution of phosphate buffer pH 7 (N_2 sparged for 5 minutes), and 31.5 mL ddH_2O (N_2 sparged for 5 minutes) were added. Then, the resulting solution was degassed by sparging with nitrogen for 5 min, parafilmed, and placed 5 cm away from two 40 W blue LED lamps. The sample was irradiated for 15 hours with stirring at 550 rpm and fan cooling. After 15 hours, 10 equiv. of L-cysteine and 20 mL of sodium bicarbonate (saturated aq.) were added to the stirring solution at room temperature. After being stirred for 2 hours, the sample was extracted using ethyl acetate and washed with brine (saturated aq.). The organic layers were combined and concentrated by rotary evaporation followed by high vacuum. The product was then purified by reverse phase flash chromatography using acetonitrile:water to give 29.6% isolated yield (28.5 mg).

2-amino-3-(1-methyl-1*H*-indazol-7-yl)propanoic acid (S4): To a 2-dram vial charged with a stir bar, methyl 2-acetamido-3-(1-methyl-1*H*-indazol-7-yl)propanoate (25 mg, 0.091 mmol, 1 equiv.), LiOH (217.5 mg, 9.1 mmol, 100 equiv.), and 5 mL of ddH_2O were added. The reaction was stirred at 80 °C overnight and monitored by TLC. Upon consumption of the starting material, the solution was concentrated, redissolved in minimal ddH_2O, and purified by reverse phase HPLC using acetonitrile:water to give 24.6% isolated yield (4.9 mg).

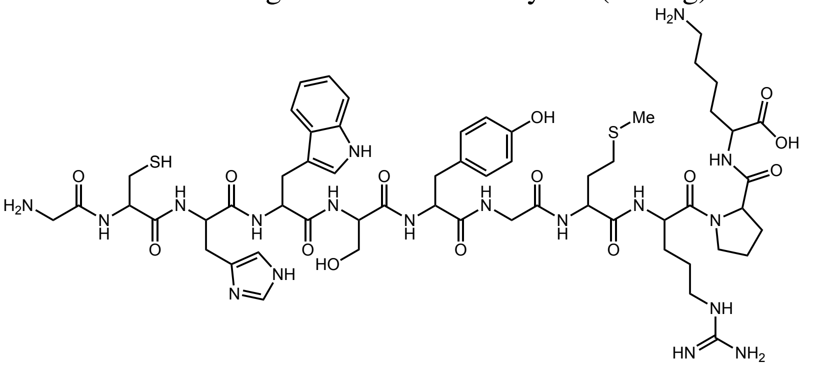

H_2N-Gly-Cys-His-Trp-Ser-Tyr-Gly-Met-Arg-Pro-Lys-CO_2H was synthesized by solid phase peptide synthesis using Fmoc-protected amino acids on 2-chlorotrityl chloride resin, cleaved with 95:2.5:2.5 TFA:TIPS:H_2O solution, and purified by reverse phase flash chromatography.

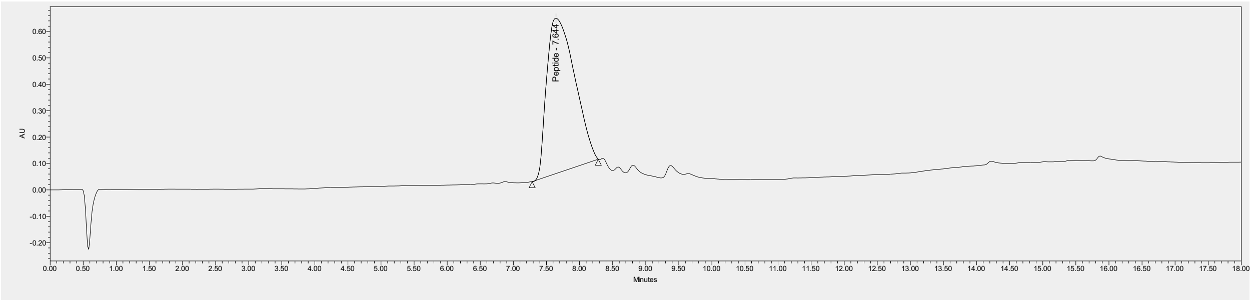

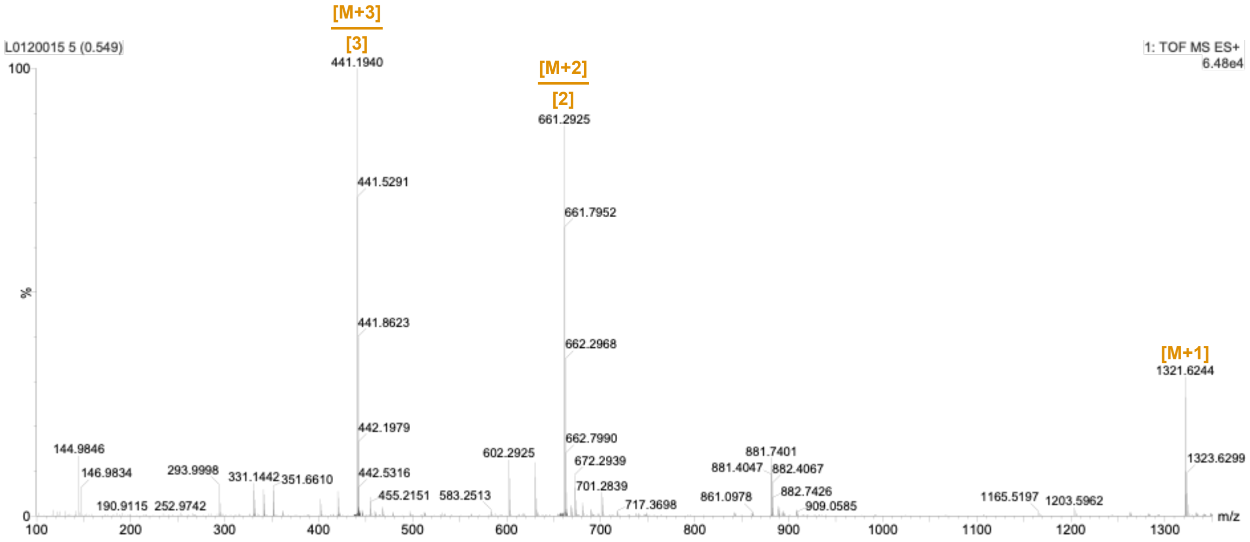

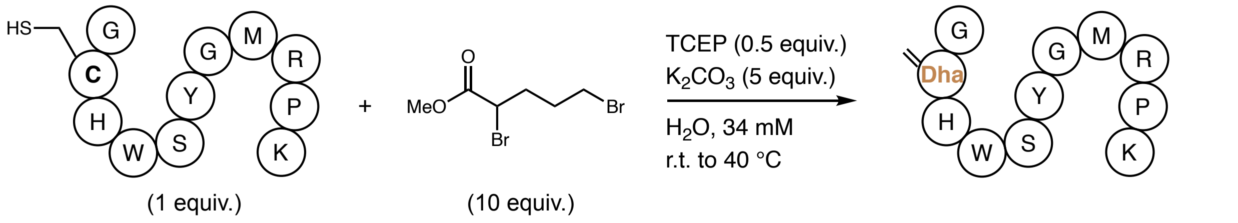

H_2N-Gly-Dha-His-Trp-Ser-Tyr-Gly-Met-Arg-Pro-Lys-CO_2H: To a 2-dram vial charged with a stir bar, H_2N-Gly-Cys-His-Trp-Ser-Tyr-Gly-Met-Arg-Pro-Lys-CO_2H (91 mg, 0.068 mmol, 1 equiv.), TCEP (9.74 mg, 0.034 mmol, 0.5 equiv.) and 2 mL of ddH_2O was added. The solution was stirred for 1 hour at room temperature; after which, methyl 2,5-dibromovalerate (184.28 mg, 0.68 mmol, 10 equiv.), and potassium carbonate (46.9 mg, 0.34 mmol, 5 equiv.) were added. The heterogenous mixture was stirred for another hour at room temperature before being heating to 40 °C for 3 hours. The reaction was monitored by UPLC/MS. Upon completion, the mixture was concentrated under reduced pressure. The residual solid was then redissolved in minimal water and purified by reverse phase flash chromatography using acetonitrile:water mix. Isolated yield = 22.7% yield (21 mg).

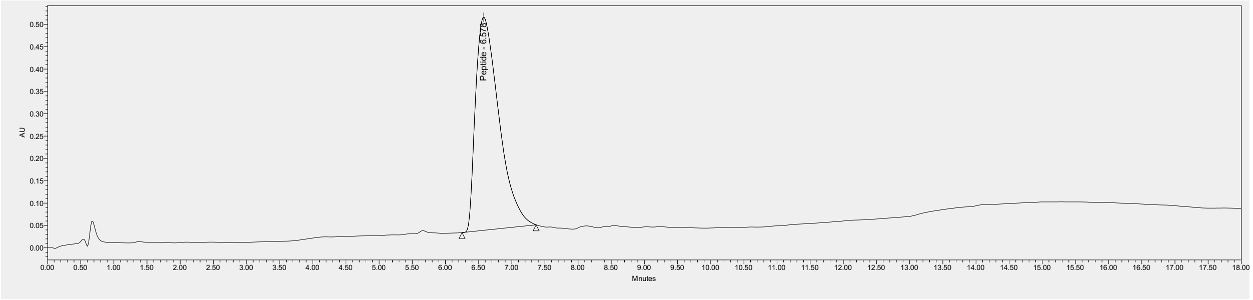

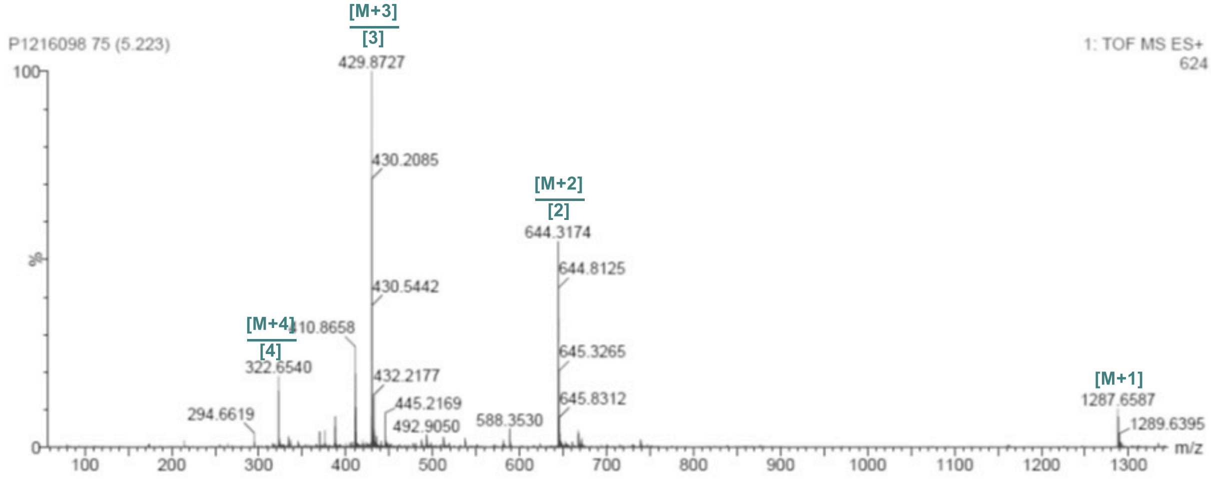

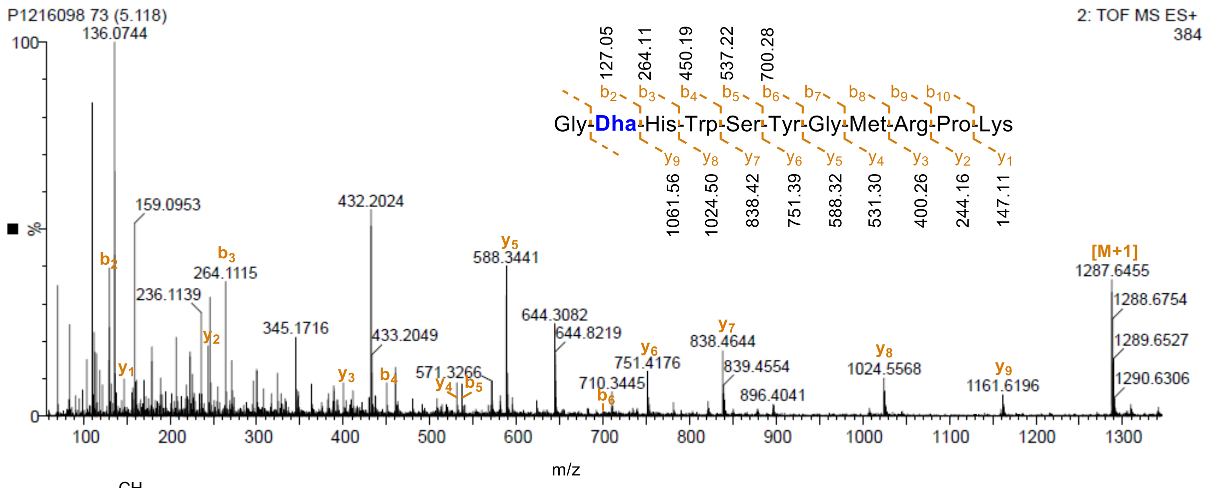

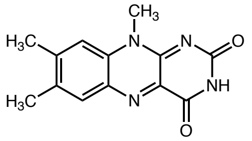

Lumiflavin was synthesized according to the reported procedure.[1] **^{1}H NMR** (400 MHz, DMSO-d_6) δ 11.31 (s, 1H), 7.90 (s, 1H), 7.78 (s, 1H), 3.96 (s, 3H), 2.50 (s, 3H), 2.40 (s, 3H). **^{13}C NMR** (126 MHz, DMSO) δ 160.01, 155.59, 150.57, 146.59, 137.11, 135.87, 133.56, 131.63, 130.85, 116.45, 31.92, 20.66, 18.85.

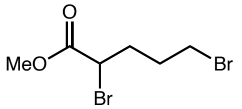

Methyl 2,5-dibromovalerate was synthesized according to the reported procedure.[2] **^{1}H NMR** (400 MHz, Chloroform-d) δ 4.26 (dd, J = 8.2, 6.1 Hz, 1H), 3.79 (s, 3H), 3.43 (t, J = 6.4 Hz, 2H), 2.43 – 1.79 (m, 4H).

Extinction Coefficients

All extinction coefficients were taken of the completely unprotected amino acids. Extinction coefficients were calculated by measuring the absorbance at 214 nm with a UV-Vis spectrophotometer at 4-5 different concentrations of each amino acid. The concentrations were then plotted with the measured absorbances as a line using Beer-Lambert law ($A = \varepsilon c$) where ε is equal to the slope of the line. All R^2 values of the lines measured were greater than 0.95 except for glycine (0.935). We used our own values measured for all amino acids but used the extinction coefficient for the peptide bond that had been reported.[3]

Measured extinction coefficients of proteinogenic amino acids

Table S6. Measured versus reported[3] extinction coefficients for proteinogenic AAs

Measured:	ε	Reported:	ε
L-Glycine =	20.7	L-Glycine =	21
L-Lysine =	32.5	L-Lysine =	41
L-Cysteine =	223.0	L-Cysteine =	225
L-Phenylalanine =	4,406.4	L-Phenylalanine =	5,200
L-Methionine =	891.2	L-Methionine =	980
L-Tryptophan =	28,582	L-Tryptophan =	29,050
L-Histidine =	4,363.5	L-Histidine =	5,125
L-Tyrosine =	4,640.8	L-Tyrosine =	5,375
L-Proline =	652.0	Peptide bond =	923

The contributions from the acetyl group on the n-terminus plus the c-terminal amide of our peptide were determined by subtracting the extinction coefficient of the peptide Ac-G-P-C-F-NH_2 from the extinction coefficient of the peptide G-P-C-F. The extinction coefficient for proline has been shown to vary depending on its environment.[3] Therefore, we determined the extinction coefficient for the proline in our specific peptide, Ac-G-P-C-F-NH_2, by subtracting the extinction coefficient of the whole peptide minus all the components except proline.
Ac-G-P-C-F-NH_2 = 9,121.4 – [20.7 (Gly) + 223.0 (Cys) + 4,406.4 (Phe) + 2,769 (3 Peptide Bonds) + 1,050.3 (Acetyl + C-terminal Amide) = 652.0 = Proline.

Measured extinction coefficients of non-proteinogenic amino acids

Table S7. Measured extinction coefficients for non-proteinogenic AAs

NPAA	ε	NPAA	ε
L-2-Naphthylalanine =	20,986	L-2-Thienylalanine =	2,809.6
H-p-Phenyl-L-Phenylalanine =	15,956	L-3-Pyridylalanine =	1,060.6
3-Benzothienyl-L-alanine =	14,625	D-3-Cyclohexylalanine =	61.3
(S)-2-Amino-3-quinolin-2-yl-propionic acid =	11,676	Acetyl + C-terminal Amide =	1,050.3
1-Methyl-1H-indazol-7-alanine =	15,311		

Measured extinction coefficients of substituted phenylalanine derivatives

Due to the inaccessibility of all 96 amino acids from our plate, we measured the extinction coefficients of four derivatives of phenylalanine and determined the average to be similar to the value for phenylalanine itself. As a result, we made the assumption that the substitutions would be approximately the same as the core amino acid itself.

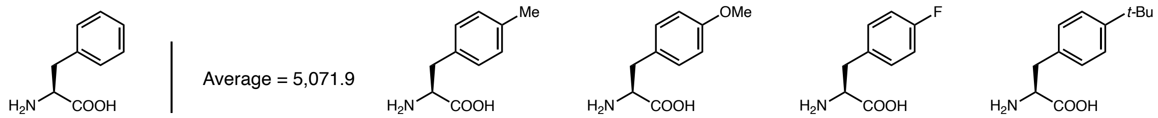

AA:	L-Phenylalanine	4-Me-L-Phenylalanine	4-MeO-L-Phenylalanine	4-F-L-Phenylalanine	4-*t*-B*u*-L-Phenylalanine
ε	4,406.4	6,845	2,830	2,334.2	8,278.3

Figure S2. Extinction coefficients of substituted phenylalanine derivatives

Assumptions to determine yields

Table S8. Assumptions made to determine the yields of all 96 peptides by LC analysis

Molecule	Assumption	Molecule	Assumption
Ac-GPXF-NH_2 =	3 Peptide bonds	Aromatics =	L-Phenylalanine
Pyridines =	L-3-Pyridylalanine	Naphthyls =	L-2-Naphthylalanine
Quinolines/Isoquinolines =	(S)-2-Amino-3-quinolin-2-yl-propionic acid	Benzyl + Phenylalanine =	L-Phenylalanine + L-Phenylalanine
Pyrimidines =	L-3-Pyridylalanine	Biphenyl =	H-p-Phenyl-L-Phenylalanine
Indoles =	L-Tryptophan	Pyridine + Phenylalanine =	L-3-Pyridylalanine + L-Phenylalanine
Indazole/Azaindazoles =	1-Methyl-1H-indazol-7-alanine	Pyrazole + Phenylalanine =	L-Histidine + L-Phenylalanine
Benzothiophenes =	3-Benzothienyl-L-alanine	Aliphatics =	Average of natural alkyls (37.8)
Pyrazoles =	L-Histidine	Aliphatic + Aryl group =	Average alkyl + L-Phenylalanine
Thiophene =	L-2-Thienylalanine	Methionine =	L-Methionine

Example calculation to determine yield

Note: The flow rate used for the LC analysis was 0.5 mL/min, and the path length of the Waters Acquity UPLC H-Class was 1 cm. The UPLC reported all absorbance measurements as microAU×sec. All injections volumes were 10 μL, and the reactions were run on a 0.00233 mmol scale.

Example calculation to determine the amount and yield of product **1A'**:

Beer-Lambert's Law Modified for HPLC[4]: $A \times F = 10^3 \times \varepsilon \times b \times N_0$

Where: A = absorbance (AU×min), F = flow rate (mL/min), ε = extinction coefficient (AU×L/mol×cm), b = path length (cm), N_0 = mol injected (mol)

Extinction Coefficient for **1A'** = [20.7 (Gly) + 652.0 (Pro) + 1060.6 (Pyr) + 4,406.4 (Phe) + [923 × 3] (3 Peptide bonds) + 1050.3 (Acetyl + C-terminal Amide)] = <u>9,959 AU×L/mol×cm</u>

Absorbance for **1A'** = 708,389 + 1,432,789 = <u>2,141,178 microAU×sec</u>

$N_0 = A \times F / 10^3 \times \varepsilon \times b$
N_0 = [(2,141,178 μAU×sec) × (1 min/60 sec) × (1 AU/10^6 μAU))] × (0.5 mL/min) / (10^3 mL/L) × (9,959 AU×L/mol×cm) × (1 cm) = <u>1.79 × 10^{-9} mol</u> (injected mol)

Concentration of product (M) = injected mol / injected L
M = 1.79 × 10^{-9} mol / 0.00001 L = <u>1.79 × 10^{-4} M</u>

Amount of product (mg) = concentration of product (M) × volume of sample (M) × MW of product
Amount = (1.79 × 10^{-4} M)×(0.001 L)×(538.6 g/mol)×(1000 mg/1 g) = <u>0.096 mg of product **1A'**</u>

Yield = (amount of pdt (mg) / theoretical amount of pdt (mg)) × 100%
Yield = (0.096 mg)/[(0.00233 mmol) × (538.6 mg/mmol)] = 0.0768 × 100% = <u>7.7% yield of product **1A'**</u>

Thrombin Inhibition Studies

Procedure: The ability of peptides to inhibit Thrombin was tested using the Sigma Thrombin Inhibitor Screening Kit (MAK243). The test peptides were solvated in water and transferred to the assay plates using ECHO550 (Beckman Inc.). The assay was miniaturized further in 384 well plate format. First, Thrombin (12.5 μL) was preincubated with 80 μM of peptides (0.5 μL) at room temperature for 15 minutes, followed by the addition of the synthetic AMC-based peptide substrate (12 μL). The released fluorophore AMC from proteolytic cleavage of the substrate by Thrombin at 37 ^{0}C was then quantified by a BioTek Neo fluorescence reader at Ex/Em = 350/450 nm. PPACK dichloride was used as a positive control, and buffer (no thrombin) was used as a negative control. Percent inhibition at 30 minutes of incubation with the 72 screening grade peptides was calculated as an average of two independent experiments. For the Ac-Gly-Pro-Phe-Phe-NH_2 inhibitor and the resynthesized (L)- and (D)-diastereomer of Ac-Gly-Pro-[N(Me)-2-Ind]-Phe-NH_2 **3C'**, percent inhibition at 30 minutes incubation was calculated as an average of three independent experiments.

Table S9. Thrombin inhibition data

Peptide:	1st run:	2nd run:	Average:	Standard Deviation:
PPACK	100	-	-	-
Buffer	0	-	-	-
1A'	92.3	92.0	92.1	0.1
2A'	8.5	8.8	8.7	0.1
5A'	-10.7	-6.6	-8.6	1.4
6A'	-6.0	0.8	-2.6	2.4
7A'	-11.3	-2.6	-6.9	3.1
8A'	22.4	23.5	22.9	0.4
10A'	18.6	20.1	19.4	0.5
11A'	16.7	19.3	18.0	0.9
12A'	5.2	9.5	7.4	1.5
1B'	-16.8	-13.7	-15.3	1.1
3B'	70.1	69.4	69.8	0.2
4B'	-2.1	-2.9	-2.5	0.3
5B'	12.1	14.5	13.3	0.8
6B'	14.0	12.9	13.4	0.4
7B'	-14.6	-12.1	-13.3	0.9
8B'	-11.3	-5.9	-8.6	1.9
9B'	6.0	8.5	7.2	0.9
10B'	4.3	5.5	4.9	0.4
1C'	23.0	22.6	22.8	0.1
3C'	96.7	97.0	96.9	0.1
4C'	82.5	81.5	82.0	0.4
5C'	29.7	29.8	29.7	0.0
6C'	79.4	79.0	79.2	0.1
7C'	-14.2	-10.6	-12.4	1.3
8C'	-18.3	-15.8	-17.1	0.9
9C'	-10.4	-7.0	-8.7	1.2
10C'	12.7	12.6	12.7	0.0
11C'	5.0	8.9	7.0	1.4
12C'	-2.5	2.7	0.1	1.8
2D'	79.1	77.7	78.4	0.5
3D'	77.8	76.9	77.3	0.3
5D'	51.5	49.8	50.7	0.6

6D'	-5.9	-3.1	-4.5	1.0
7D'	2.4	6.1	4.2	1.3
11D'	-9.4	-5.2	-7.3	1.5
1E'	80.2	79.9	80.1	0.1
3E'	48.5	49.1	48.8	0.2
5E'	-1.3	2.7	0.7	1.4
6E'	-13.7	-9.9	-11.8	1.3
7E'	-6.8	-0.5	-3.6	2.2
8E'	-21.5	-16.9	-19.2	1.6
9E'	2.6	8.1	5.4	2.0
10E'	3.5	6.0	4.7	0.9
11E'	8.9	15.7	12.3	2.4
12E'	71.5	70.6	71.0	0.3
1F'	40.3	39.3	39.8	0.4
3F'	23.1	28.3	25.7	1.9
7F'	-4.6	-0.7	-2.7	1.4
8F'	-29.3	-24.7	-27.0	1.6
9F'	37.8	36.4	37.1	0.5
10F'	0.1	6.5	3.3	2.3
11F'	2.3	5.9	4.1	1.3
12F'	11.6	14.6	13.1	1.1
1G'	11.0	13.4	12.2	0.8
2G'	87.1	86.7	86.9	0.1
3G'	67.4	67.6	67.5	0.0
4G'	34.1	33.6	33.9	0.2
5G'	-5.5	0.2	-2.6	2.0
6G'	-11.0	-6.7	-8.8	1.5
7G'	21.1	21.1	21.1	0.0
8G'	12.8	16.8	14.8	1.4
9G'	-15.8	-11.6	-13.7	1.5
10G'	-3.9	-1.8	-2.9	0.8
11G'	-16.4	-14.6	-15.5	0.7
12G'	18.3	18.0	18.2	0.1
1H'	-3.1	0.9	-1.1	1.4
4H'	1.4	3.4	2.4	0.7
6H'	0.3	5.1	2.7	1.7
8H'	10	10.4	10.2	0.1
10H'	2.5	4.1	3.3	0.6
11H'	18.6	17.0	17.8	0.6
12H'	15.4	15.2	15.3	0.1

Peptide:	1st run:	2nd run:	3rd run:	Average:
L-*N*-methyl-2-indole 3C’	64.0	68.7	67.7	66.8
D-*N*-methyl-2-indole 3C’	27.0	29.2	33.5	29.9
Ac-G-P-F-F-NH_2	9.4	5.5	6.5	7.1

Analytical Data of Products

Amino Acids

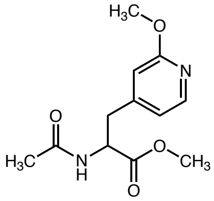

Methyl 2-acetamido-3-(2-methoxypyridin-4-yl)propanoate
Isolated Yield 49.0% (43 mg). **^{1}H NMR** (400 MHz, Chloroform-*d*) δ 8.12 (d, *J* = 5.0 Hz, 1H), 6.68 (d, *J* = 5.1 Hz, 1H), 6.55 (s, 1H), 6.04 (d, *J* = 7.8 Hz, 1H), 4.91 (q, *J* = 6.1 Hz, 1H), 3.95 (s, 3H), 3.76 (s, 3H), 3.40 – 2.77 (m, 2H), 2.02 (s, 3H). **^{13}C NMR** (101 MHz, Chloroform-*d*) δ 171.57, 170.12, 164.26, 149.07, 146.25, 118.07, 111.38, 54.10, 52.76, 52.49, 37.25, 23.18. **HRMS (ESI-ToF):** m/z calculated for $C_{12}H_{17}N_2O_4$ [MH]$^+$: 253.1188. Found 253.1183.

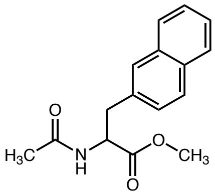

Methyl 2-acetamido-3-(naphthalen-2-yl)propanoate
Isolated Yield 52.7% (50 mg). **^{1}H NMR** (400 MHz, Chloroform-*d*) δ 7.89 – 7.67 (m, 3H), 7.64 – 7.52 (m, 1H), 7.53 – 7.33 (m, 2H), 7.23 (dd, *J* = 8.4, 1.8 Hz, 1H), 6.10 (d, *J* = 7.3 Hz, 1H), 4.97 (dt, *J* = 7.8, 5.9 Hz, 1H), 3.72 (s, 3H), 3.27 (qd, *J* = 13.9, 5.9 Hz, 2H), 1.96 (s, 3H). **^{13}C NMR** (101 MHz, Chloroform-*d*) δ 172.26, 169.86, 133.49, 132.55, 128.35, 128.09, 127.75, 127.75, 127.63, 127.30, 126.30, 125.88, 53.27, 52.44, 38.06, 23.16. **HRMS (ESI-ToF):** m/z calculated for $C_{16}H_{18}NO_3$ [MH]$^+$: 272.1287. Found 272.1274.

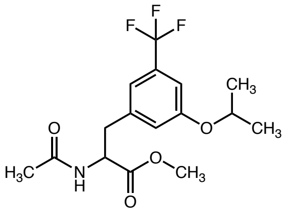

Methyl 2-acetamido-3-(3-isopropoxy-5-(trifluoromethyl)phenyl)propanoate
Isolated Yield 43.6% (53 mg). **^{1}H NMR** (400 MHz, Chloroform-*d*) δ 6.99 (t, *J* = 2.0 Hz, 1H), 6.87 (dq, *J* = 1.6, 0.8 Hz, 1H), 6.78 (t, *J* = 1.9 Hz, 1H), 5.99 (d, *J* = 7.6 Hz, 1H), 4.88 (dt, *J* = 7.6, 5.7 Hz, 1H), 4.54 (hept, *J* = 6.1 Hz, 1H), 3.74 (s, 3H), 3.14 (qd, *J* = 13.8, 5.6 Hz, 2H), 2.01 (s, 3H), 1.34 (s, 3H), 1.33 (s, 3H). **^{13}C NMR** (101 MHz, Chloroform-*d*) δ 171.86, 169.81, 158.36, 138.52, 132.19, 131.88, 120.11, 118.08 (d, *J* = 3.8 Hz), 111.44 (d, *J* = 4.0 Hz), 70.52, 53.18, 52.60, 37.89, 23.26, 22.02. **^{19}F NMR** (376 MHz, Chloroform-*d*) δ -62.75. **HRMS (ESI-ToF):** m/z calculated for $C_{16}H_{21}F_3NO_4$ [MH]$^+$: 348.1423. Found 348.1403.

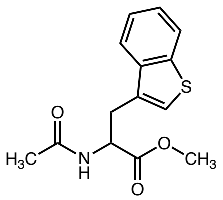

Methyl 2-acetamido-3-(benzo[*b*]thiophen-3-yl)propanoate
Isolated Yield 25.8% (25 mg). **^{1}H NMR** (400 MHz, Chloroform-*d*) δ 8.06 – 7.80 (m, 1H), 7.81 – 7.63 (m, 1H), 7.64 – 7.30 (m, 2H), 7.13 (s, 1H), 6.10 (d, *J* = 7.7 Hz, 1H), 5.00 (dt, *J* = 7.7, 5.7 Hz, 1H), 3.69 (s, 3H), 3.56 – 2.99 (m, 2H), 1.96 (s, 3H). **^{13}C NMR** (101 MHz, Chloroform-*d*) δ 172.20, 169.94, 140.37, 139.05, 130.86, 128.19, 124.57, 124.27, 123.91, 123.02, 121.63, 52.60, 30.80, 23.28. **HRMS (ESI-ToF):** m/z calculated for $C_{14}H_{16}NO_3S$ $[MH]^+$: 278.0851. Found 278.0831.

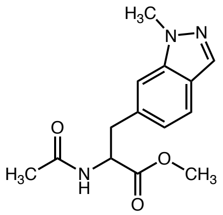

Methyl 2-acetamido-3-(1-methyl-1*H*-indazol-6-yl)propanoate
Isolated Yield 46.7% (45 mg). **^{1}H NMR** (400 MHz, Chloroform-*d*) δ 8.07 – 7.77 (m, 1H), 7.61 (dd, *J* = 8.2, 0.8 Hz, 1H), 7.11 (d, *J* = 1.3 Hz, 1H), 6.87 (dd, *J* = 8.3, 1.3 Hz, 1H), 6.18 (d, *J* = 7.8 Hz, 1H), 4.93 (dt, *J* = 7.8, 6.0 Hz, 1H), 4.00 (s, 3H), 3.70 (s, 3H), 3.24 (qd, *J* = 13.9, 6.0 Hz, 2H), 1.96 (s, 3H). **^{13}C NMR** (101 MHz, Chloroform-*d*) δ 172.18, 169.83, 140.24, 134.64, 132.60, 123.19, 122.25, 121.22, 109.22, 53.44, 52.43, 38.38, 35.47, 23.17. **HRMS (ESI-ToF):** m/z calculated for $C_{14}H_{18}N_3O_3$ $[MH]^+$: 276.1348. Found 276.1345.

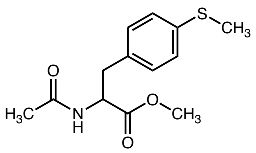

Methyl 2-acetamido-3-(4-(methylthio)phenyl)propanoate
Isolated Yield 60.9% (57 mg). **^{1}H NMR** (400 MHz, Chloroform-*d*) δ 7.23 – 7.07 (m, 2H), 7.04 – 6.89 (m, 2H), 6.07 (d, *J* = 7.6 Hz, 1H), 4.83 (dt, *J* = 7.8, 5.8 Hz, 1H), 3.70 (s, 3H), 3.05 (qd, *J* = 13.9, 5.8 Hz, 2H), 2.44 (s, 3H), 1.96 (s, 3H). **^{13}C NMR** (101 MHz, Chloroform-*d*) δ 172.10, 169.76, 137.24, 132.67, 129.75, 126.73, 53.15, 52.40, 37.29, 23.13, 15.81. **HRMS (ESI-ToF):** m/z calculated for $C_{13}H_{17}NO_3SNa$ $[MNa]^+$: 290.0827. Found 290.0805.

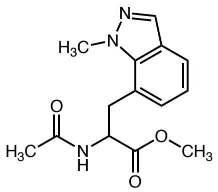

Methyl 2-acetamido-3-(1-methyl-1*H*-indazol-7-yl)propanoate
Isolated Yield 29.6% (28.5 mg). **^{1}H NMR** (400 MHz, Chloroform-*d*) δ 7.97 (s, 1H), 7.63 (dd, *J* = 6.6, 2.6 Hz, 1H), 7.12 – 6.95 (m, 2H), 6.02 (d, *J* = 8.0 Hz, 1H), 5.16 – 4.79 (m, 1H), 4.34 (s, 3H), 3.58 (s, 3H), 3.57 – 3.46 (m, 2H), 1.98 (s, 3H). **^{13}C NMR** (126 MHz, Chloroform-*d*) δ 172.23,

169.87, 138.86, 133.17, 128.47, 125.91, 120.75, 120.70, 118.76, 53.78, 52.49, 39.45, 35.19, 23.27. **HRMS (ESI-ToF):** m/z calculated for $C_{14}H_{18}N_3O_3$ $[MH]^+$: 276.1348. Found 276.1317.

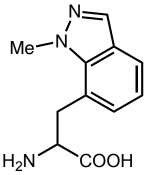

2-amino-3-(1-methyl-1*H*-indazol-7-yl)propanoic acid
Isolated Yield 24.6% (4.9 mg). **^{1}H NMR** (400 MHz, Deuterium Oxide) δ 8.02 (s, 1H), 7.71 (dd, *J* = 8.1, 1.2 Hz, 1H), 7.22 (dd, *J* = 7.1, 1.1 Hz, 1H), 7.12 (dd, *J* = 8.1, 7.0 Hz, 1H), 4.22 (s, 3H), 3.82 (dd, *J* = 9.2, 5.9 Hz, 1H), 3.73 (dd, *J* = 14.9, 5.8 Hz, 1H), 3.24 (dd, *J* = 14.9, 9.2 Hz, 1H).

Peptides

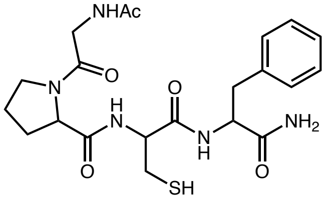

Ac-Gly-Pro-Cys-Phe-NH$_2$
^{1}H NMR (400 MHz, Deuterium Oxide) δ 7.59 – 7.04 (m, 5H), 4.59 (dd, *J* = 8.9, 6.1 Hz, 1H), 4.55 – 4.29 (m, 2H), 4.06 (s, 2H), 3.70 – 3.49 (m, 2H), 3.17 (dd, *J* = 13.9, 6.0 Hz, 1H), 2.98 (dd, *J* = 14.1, 8.9 Hz, 1H), 2.90 – 2.63 (m, 2H), 2.19 (dq, *J* = 12.7, 7.8 Hz, 1H), 2.06 – 1.90 (m, 5H), 1.77 (dq, *J* = 12.3, 6.2 Hz, 1H).

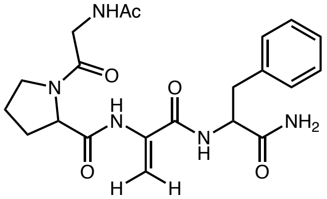

Ac-Gly-Pro-Dha-Phe-NH$_2$
^{1}H NMR (400 MHz, Deuterium Oxide) δ 7.39 – 7.18 (m, 5H), 5.54 (dd, *J* = 11.4, 1.3 Hz, 2H), 4.59 (dd, *J* = 8.8, 6.1 Hz, 1H), 4.41 (dd, *J* = 8.5, 5.2 Hz, 1H), 4.05 (d, *J* = 2.2 Hz, 2H), 3.79 – 3.41 (m, 2H), 3.21 (dd, *J* = 13.8, 6.1 Hz, 1H), 3.02 (dd, *J* = 13.9, 8.9 Hz, 1H), 2.25 (ddd, *J* = 12.4, 8.2, 6.0 Hz, 1H), 2.10 – 1.76 (m, 6H).

3C’ Isomers

Crude mixture of both diastereomers:

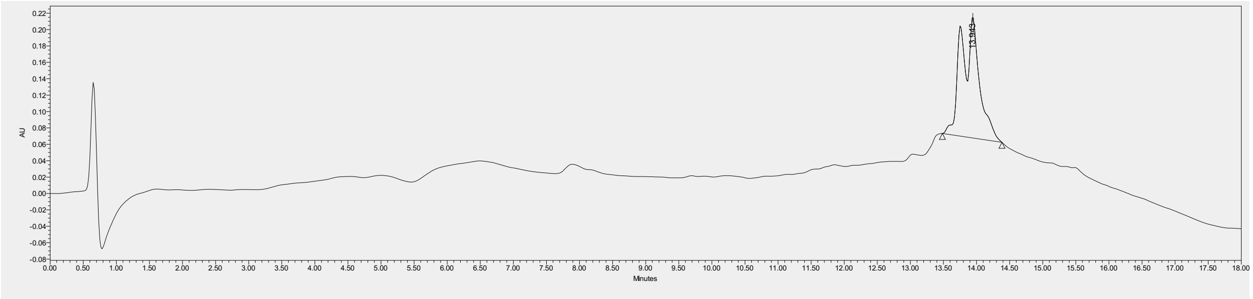

Pure products:

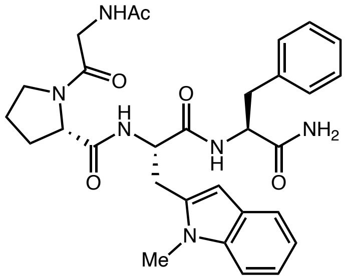

(L)-Isomer

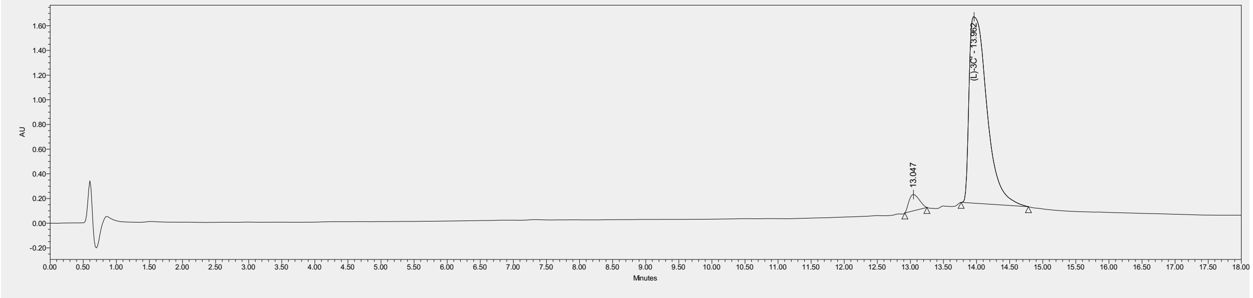

	Name	Retention Time	Area	% Area
1		13.047	1443948	4.84
2	(L)-3C'	13.962	28365465	95.16

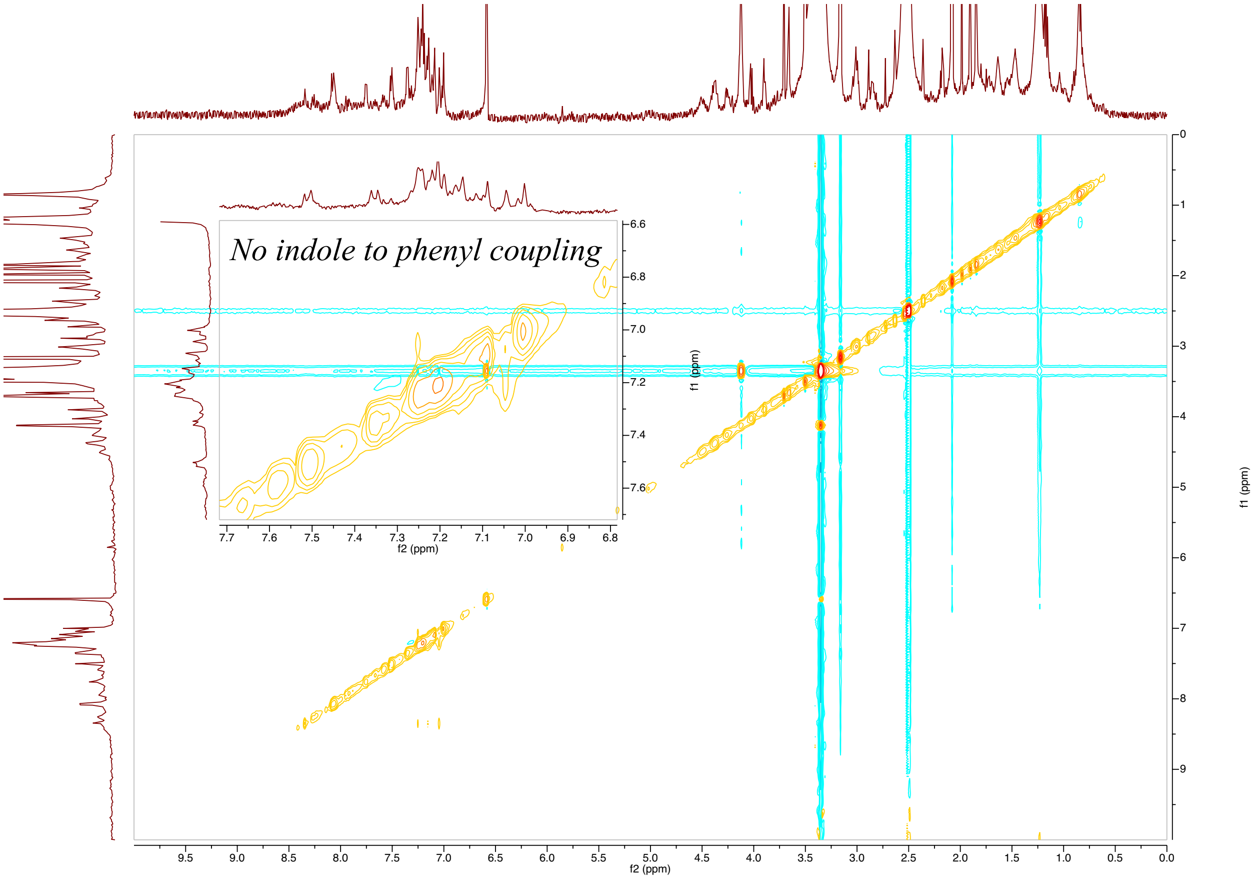

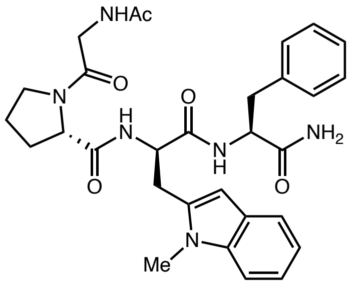

(D)-Isomer

Indole and Phenyl on same side of peptide

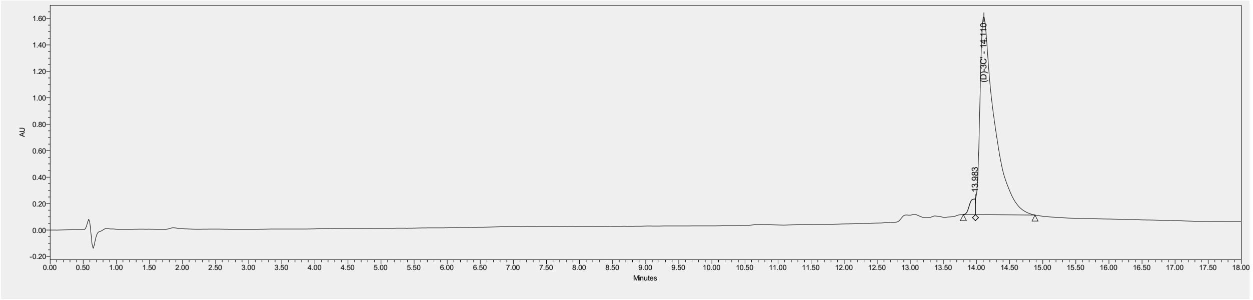

	Name	Retention Time	Area	% Area
1		13.983	707558	2.96
2	(D)-3C'	14.110	23201032	97.04

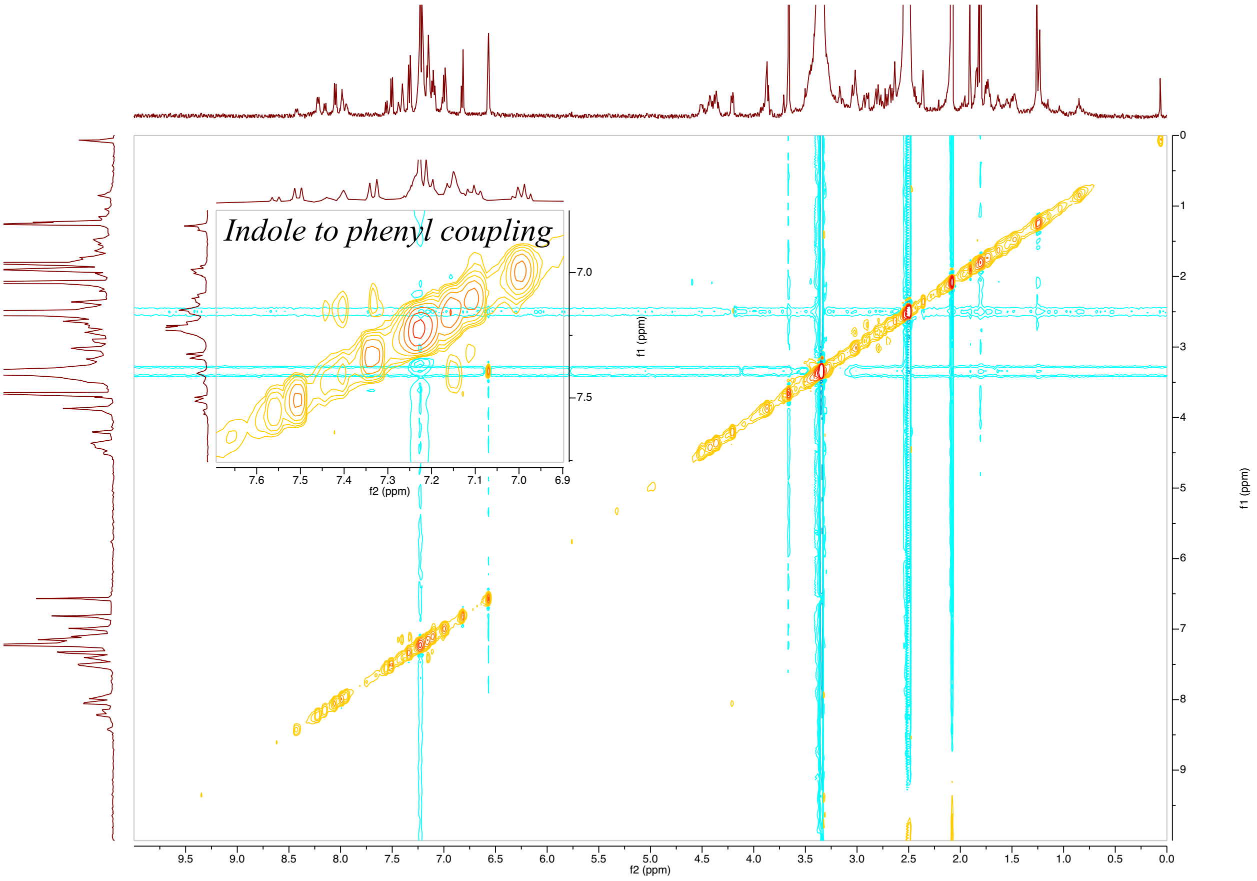

NMR Spectra

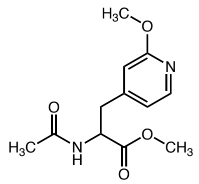
H3C
O
N
O
H3C
N
H
O
O
CH3

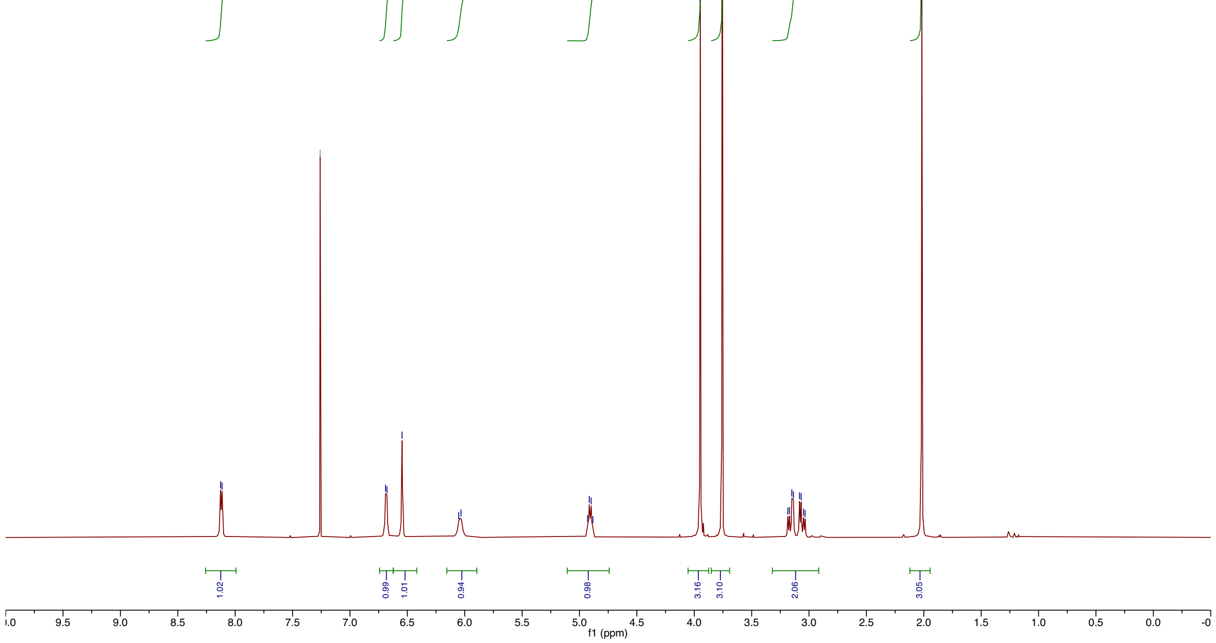
8.13
8.11
7.26 CDCl3
6.69
6.68
6.55
6.05
6.03
4.93
4.91
4.90
4.88
3.95
3.76
3.18
3.17
3.15
3.14
3.08
3.07
3.05
3.03
2.02
1.02
0.99
1.01
0.94
0.98
3.16
3.10
2.06
3.05
f1 (ppm)

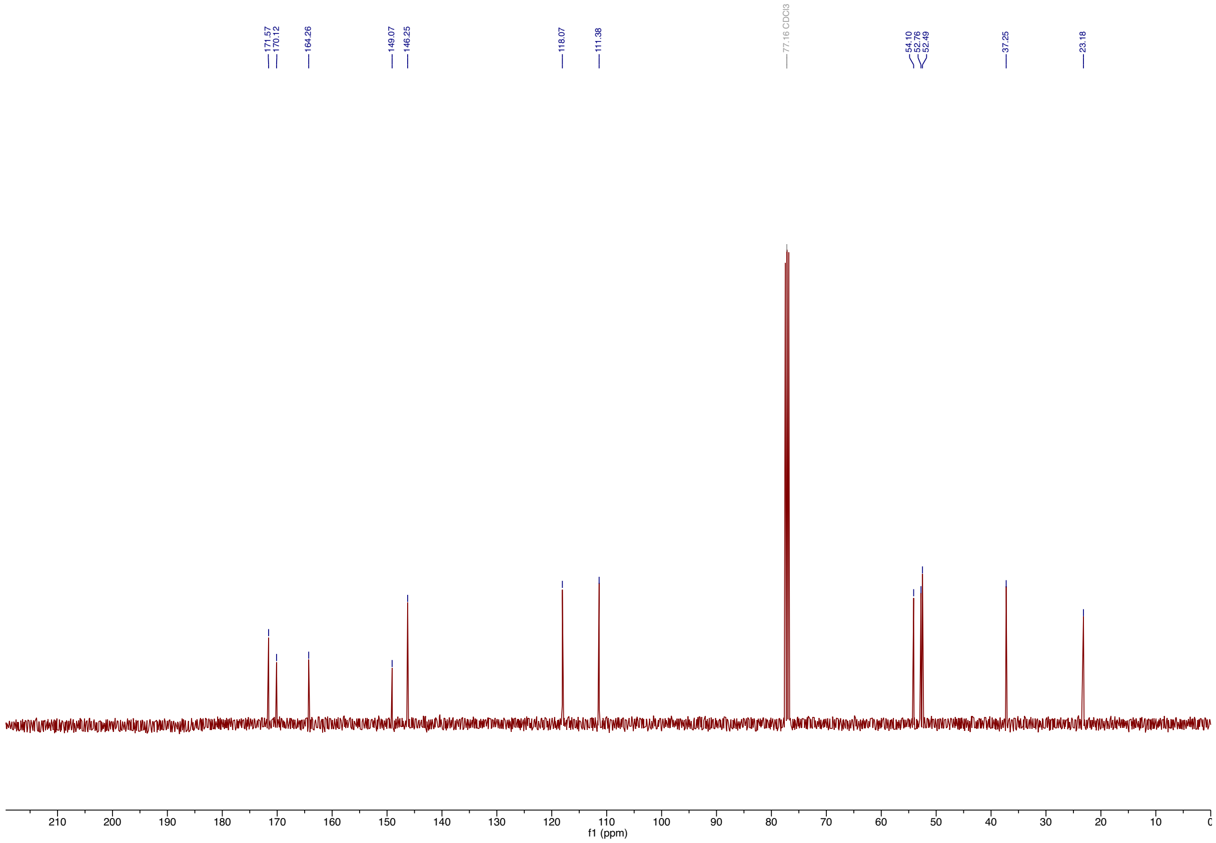
171.57
170.12
164.26
149.07
146.25
118.07
111.38
77.16 CDCl3
54.10
52.76
52.49
37.25
23.18
f1 (ppm)

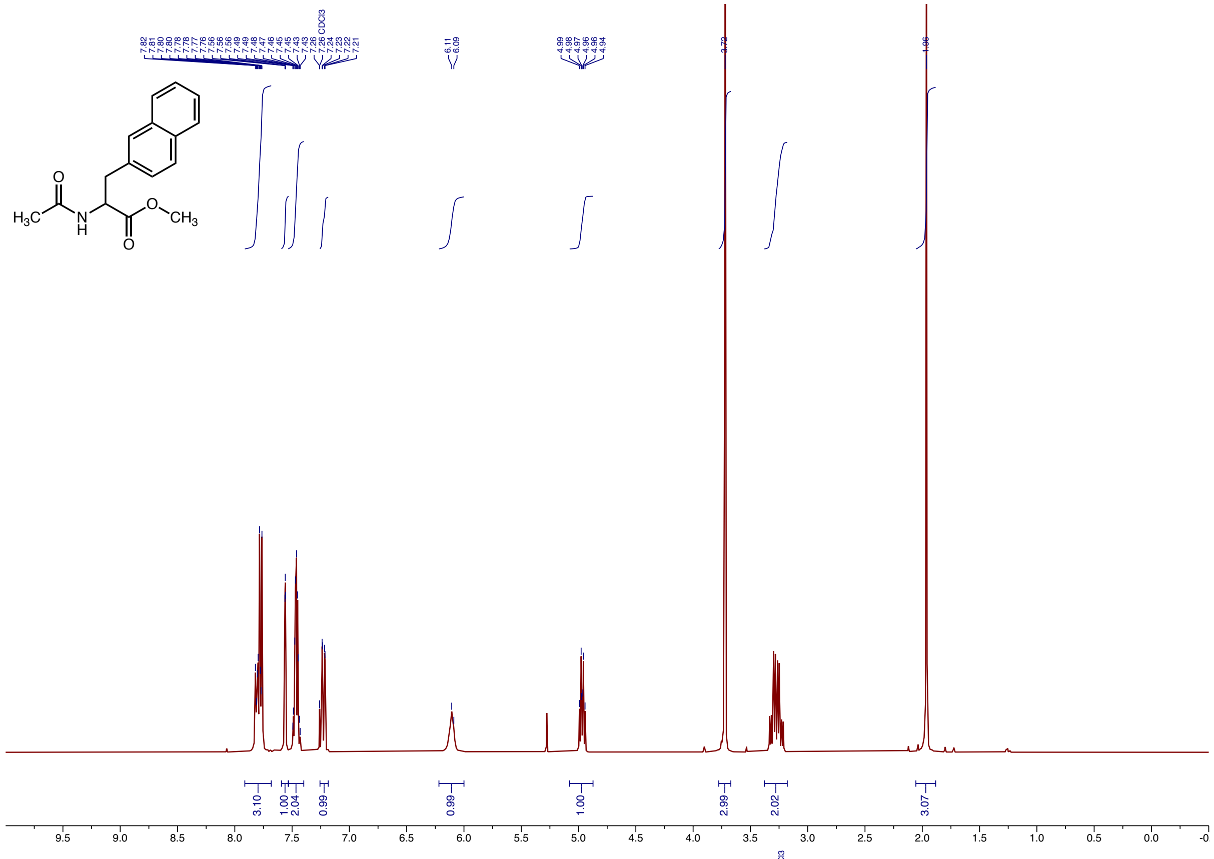
7.82 7.81 7.80 7.80 7.78 7.78 7.77 7.76 7.56 7.56 7.56 7.49 7.48 7.47 7.46 7.45 7.45 7.43 7.43 7.26 7.26 CDCl3 7.24 7.23 7.22 7.21
6.11 6.09
4.99 4.98 4.97 4.96 4.96 4.94
3.72
1.96
H3C
N
H
O
O
O
CH3
3.10
1.00
2.04
0.99
0.99
1.00
2.99
2.02
3.07

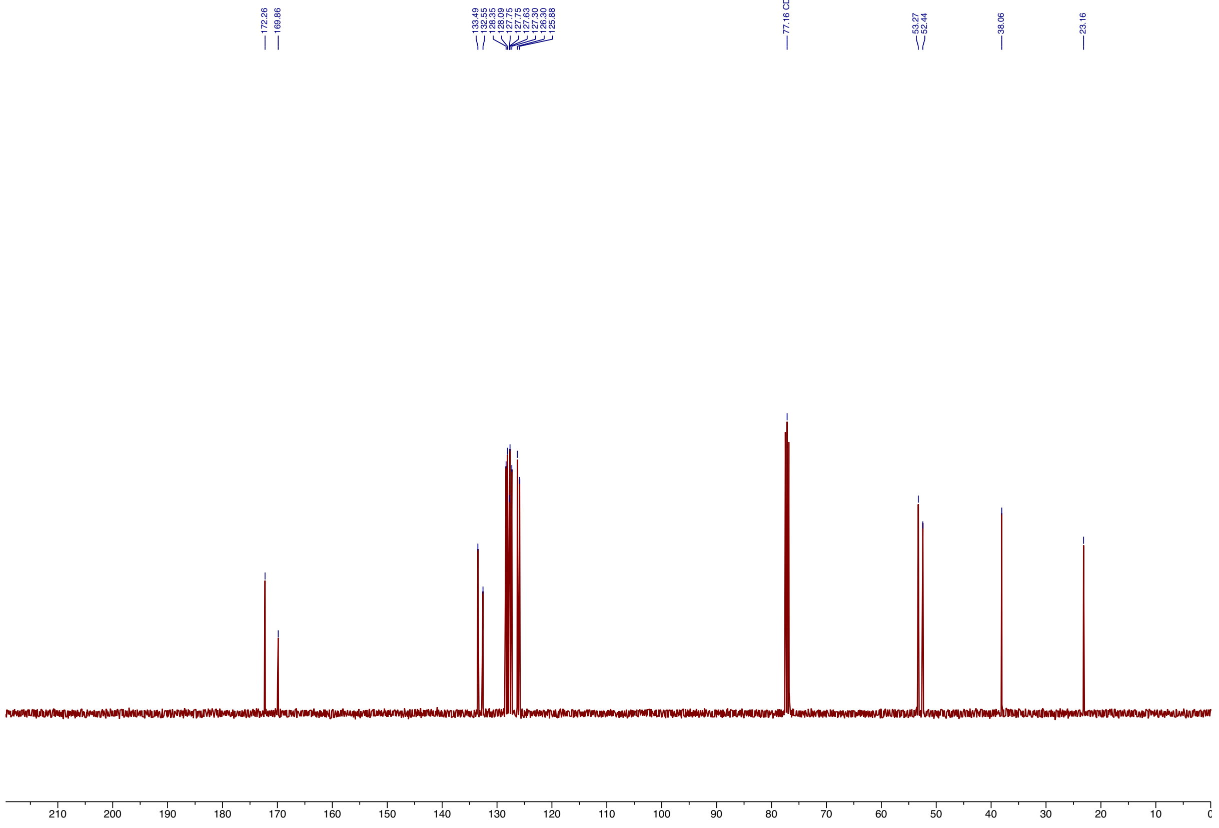
172.26
169.86
133.49 132.55 128.35 128.09 127.75 127.75 127.63 127.30 126.30 125.88
77.16 CDCl3
53.27
52.44
38.06
23.16

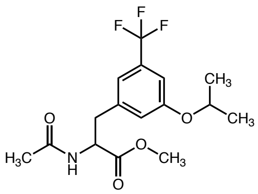
F
F
F
CH3
O
CH3
O
H3C
N
H
O
O
CH3

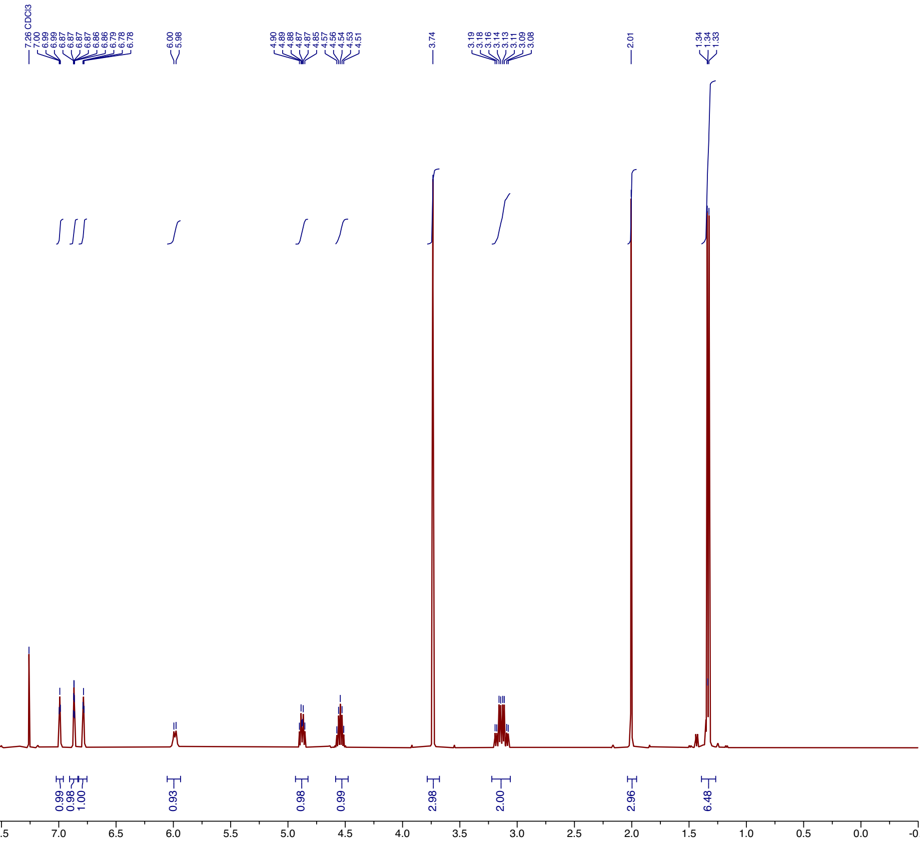
7.26 CDCl3
7.00
6.99
6.99
6.87
6.87
6.87
6.86
6.86
6.79
6.78
6.78
6.00
5.98
4.90
4.89
4.88
4.87
4.87
4.85
4.57
4.56
4.54
4.53
4.51
3.74
3.19
3.18
3.16
3.14
3.13
3.11
3.09
3.08
2.01
1.34
1.34
1.33
0.99
0.98
1.00
0.93
0.98
0.99
2.98
2.00
2.96
6.48
9.5 9.0 8.5 8.0 7.5 7.0 6.5 6.0 5.5 5.0 4.5 4.0 3.5 3.0 2.5 2.0 1.5 1.0 0.5 0.0 -0

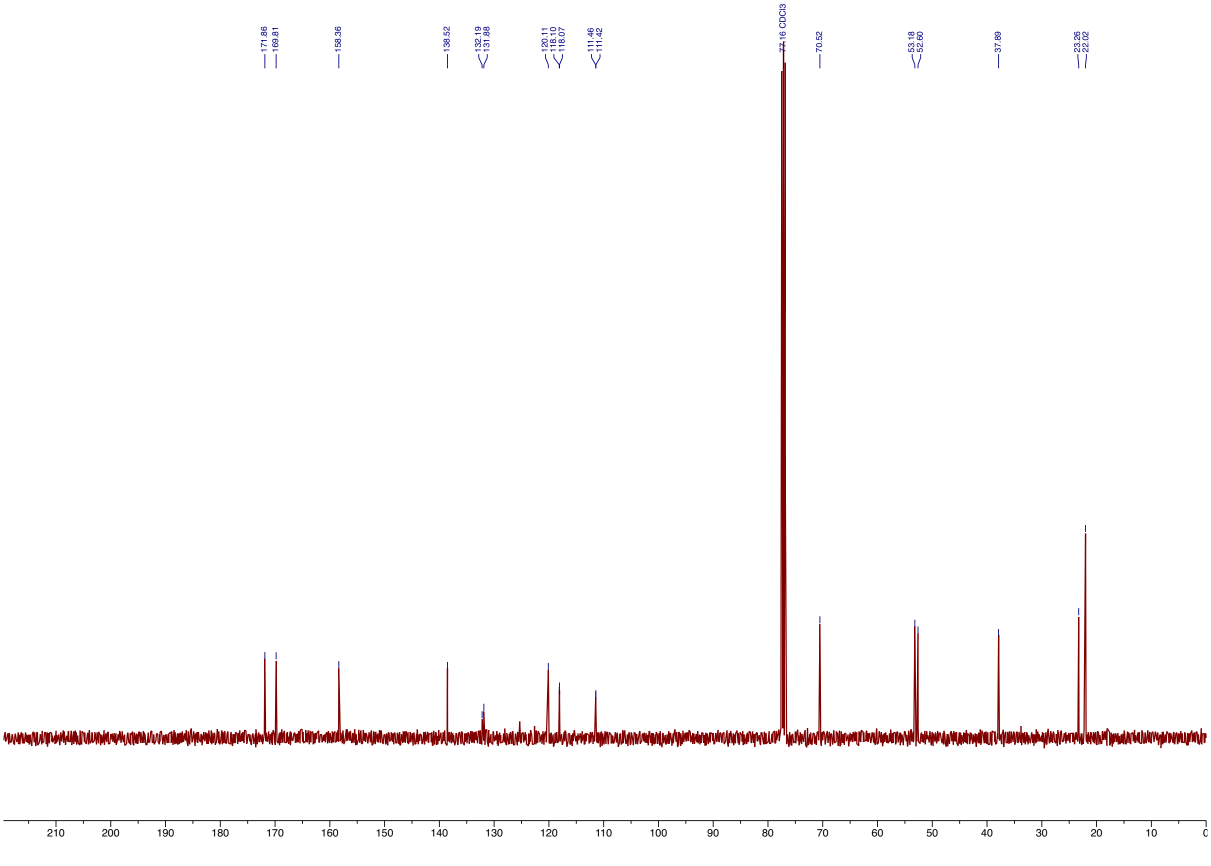
171.86
169.81
158.36
138.52
132.19
131.88
120.11
118.10
118.07
111.46
111.42
77.16 CDCl3
70.52
53.18
52.60
37.89
23.26
22.02
210 200 190 180 170 160 150 140 130 120 110 100 90 80 70 60 50 40 30 20 10 0

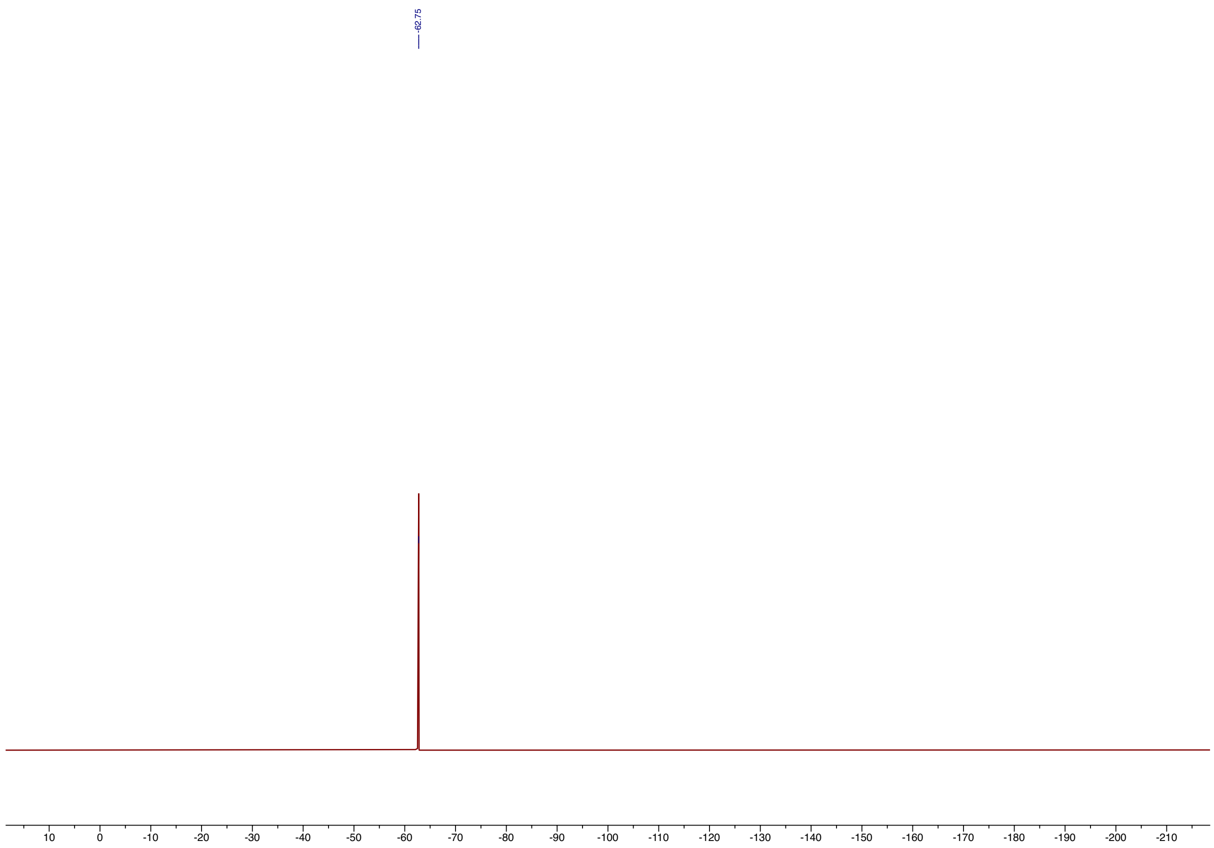
-62.75
10 0 -10 -20 -30 -40 -50 -60 -70 -80 -90 -100 -110 -120 -130 -140 -150 -160 -170 -180 -190 -200 -210

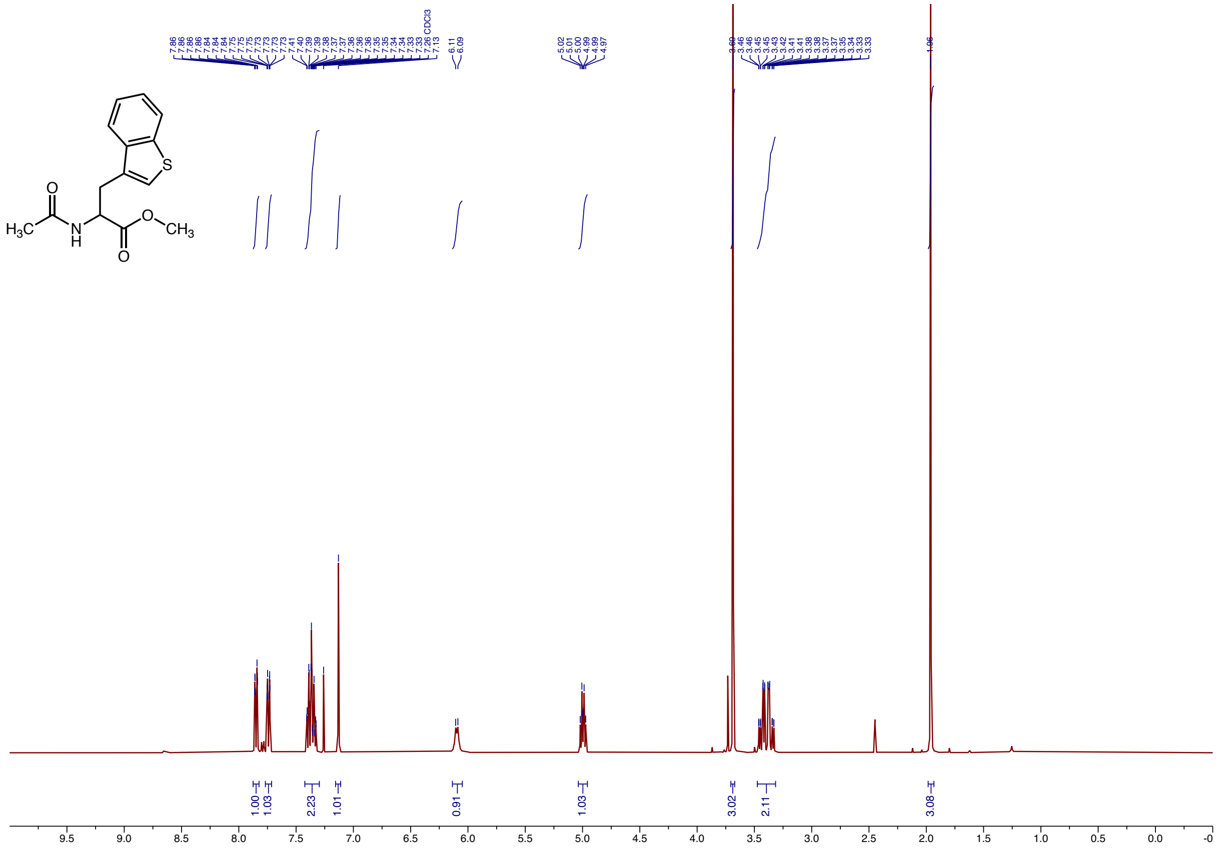

H3C
N
H
O
O
O
CH3
S
CDCl3
1.00
1.03
2.23
1.01
0.91
1.03
3.02
2.11
3.08
9.5 9.0 8.5 8.0 7.5 7.0 6.5 6.0 5.5 5.0 4.5 4.0 3.5 3.0 2.5 2.0 1.5 1.0 0.5 0.0

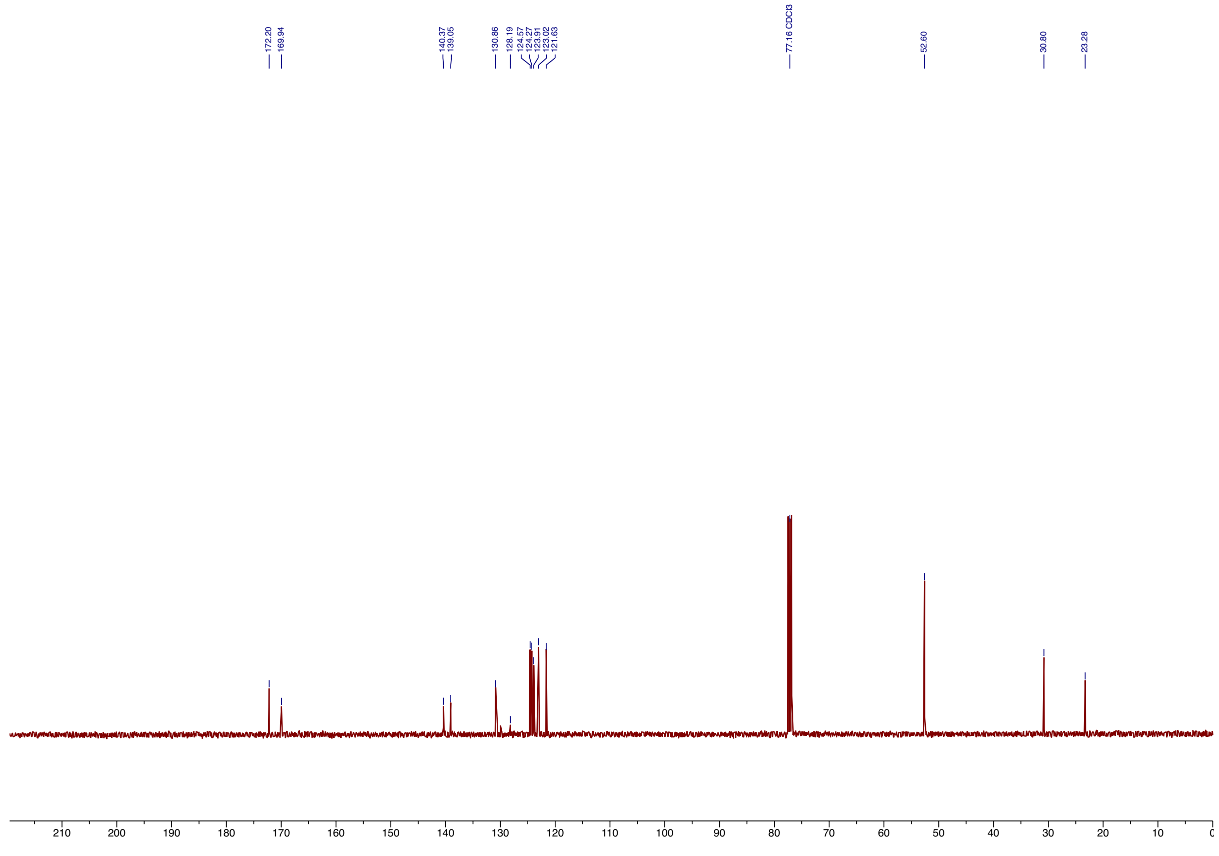

172.20
169.94
140.37
139.05
130.86
128.19
124.57
124.27
123.91
123.02
121.63
77.16 CDCl3
52.60
30.80
23.28
210 200 190 180 170 160 150 140 130 120 110 100 90 80 70 60 50 40 30 20 10

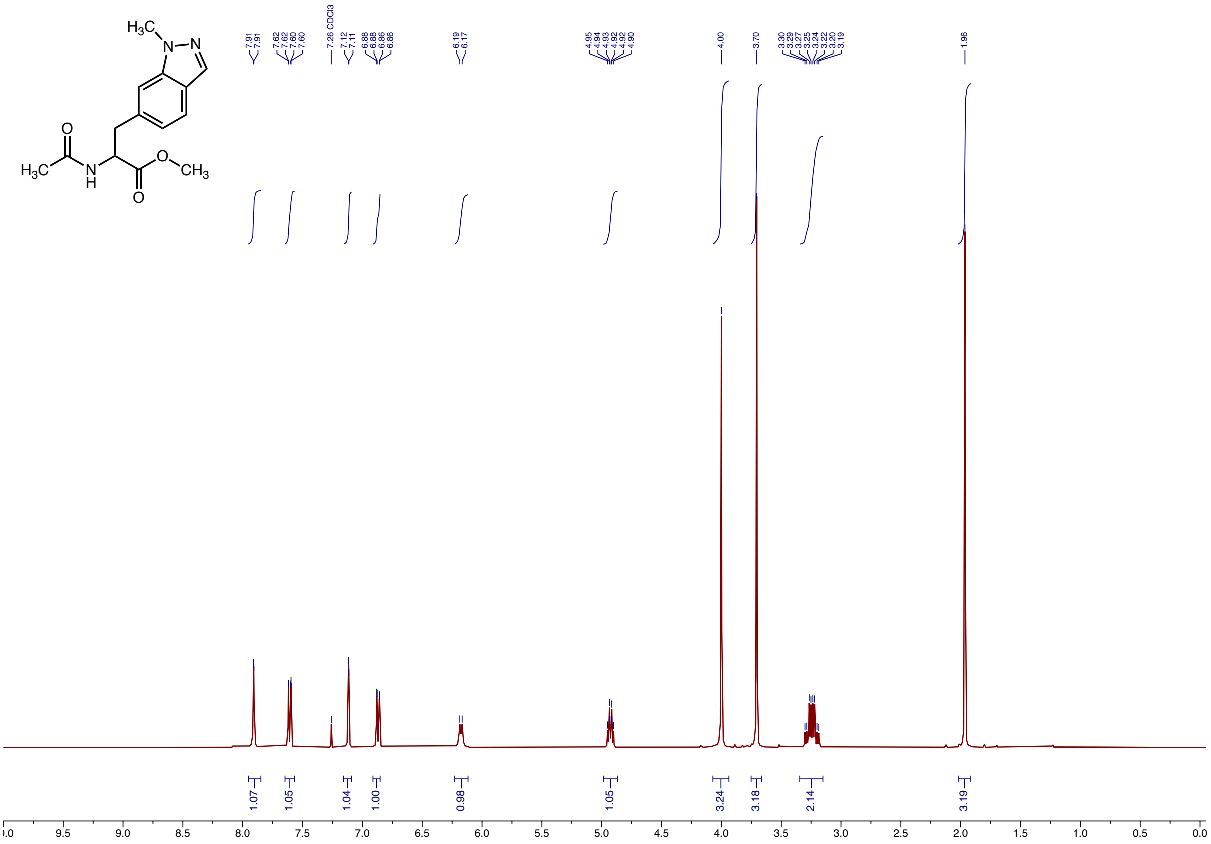
H3C
N
N
O
H3C
N
H
O
O
CH3
7.91
7.91
7.62
7.62
7.60
7.60
7.26 CDCl3
7.12
7.11
6.88
6.88
6.86
6.86
6.19
6.17
4.95
4.94
4.93
4.92
4.92
4.90
4.00
3.70
3.30
3.29
3.27
3.25
3.24
3.22
3.20
3.19
1.96
1.07
1.05
1.04
1.00
0.98
1.05
3.24
3.18
2.14
3.19

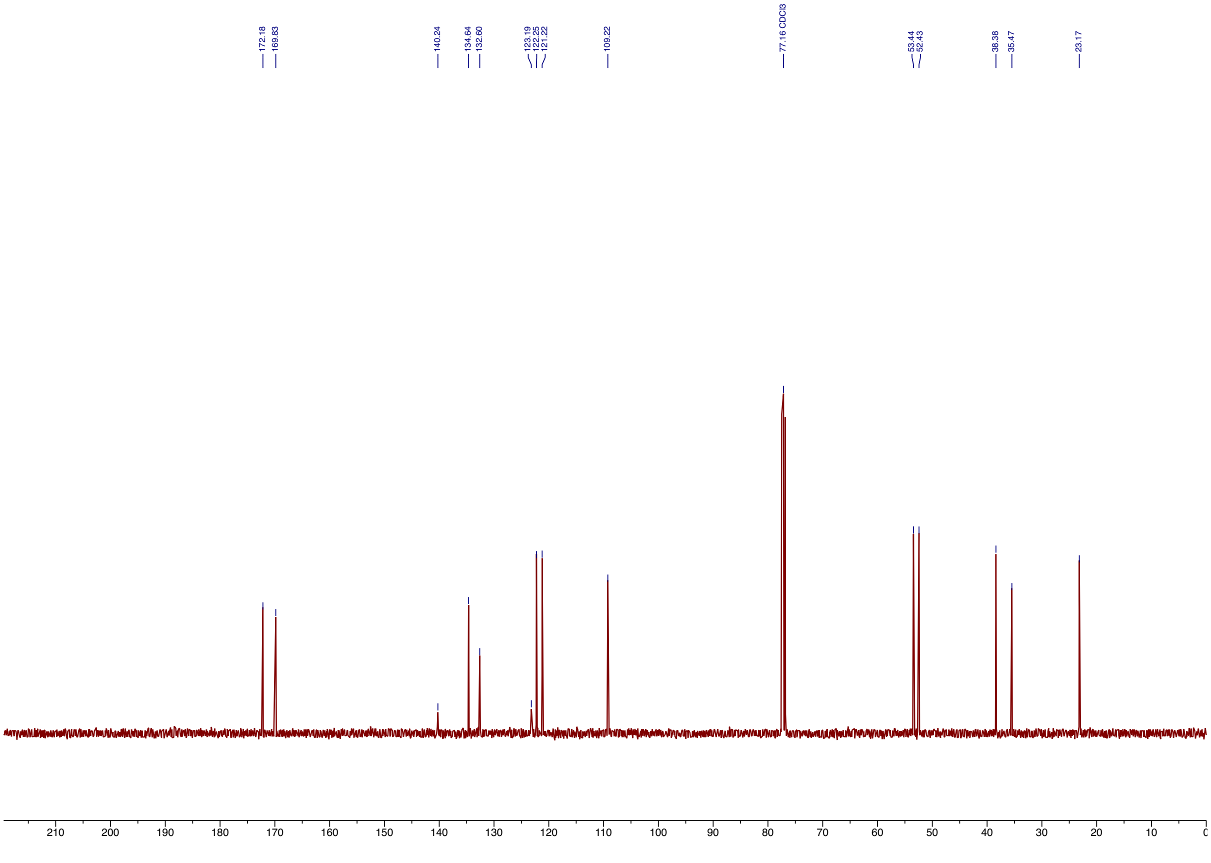
172.18
169.83
140.24
134.64
132.60
123.19
122.25
121.22
109.22
77.16 CDCl3
53.44
52.43
38.38
35.47
23.17

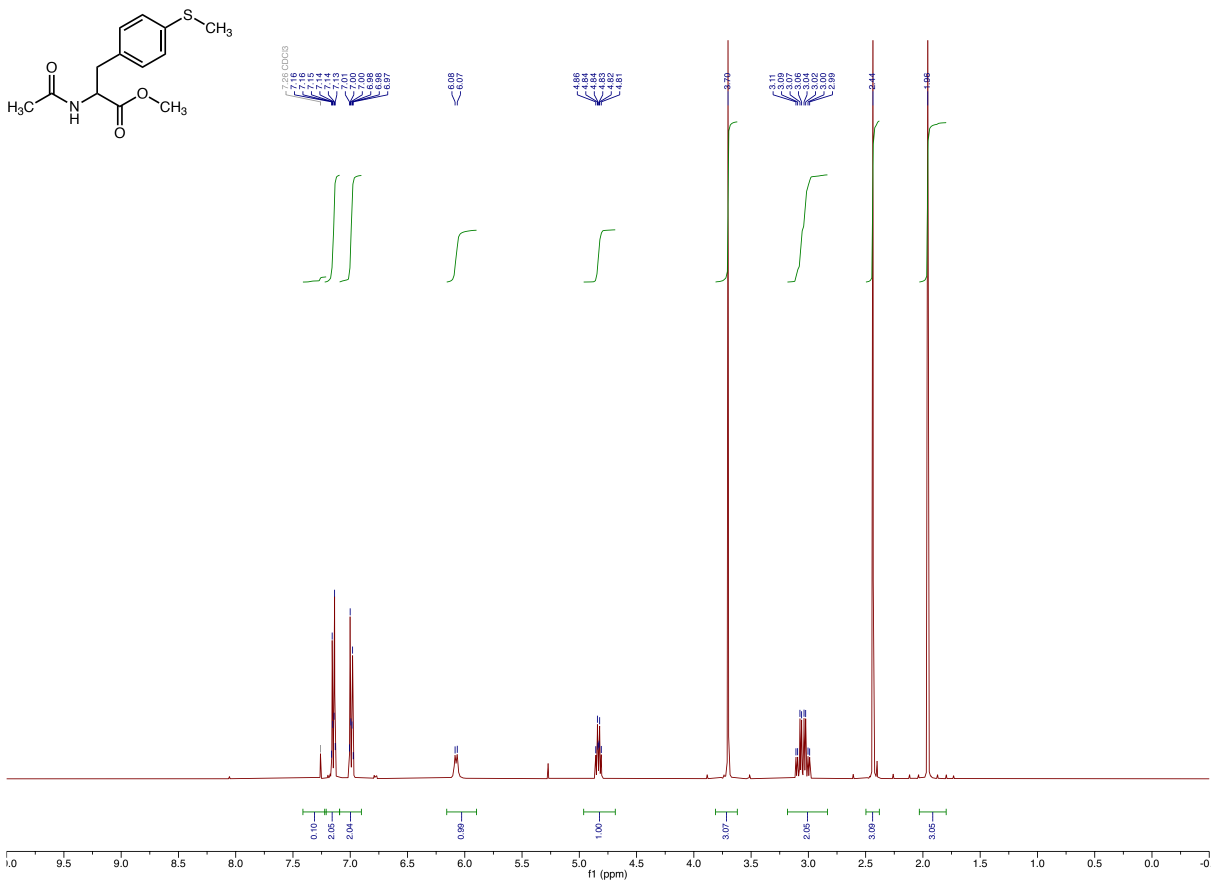
S
CH3
O
H3C
N
H
O
CH3
O
7.26 CDCl3
7.16
7.15
7.14
7.13
7.01
7.00
6.98
6.98
6.97
6.08
6.07
4.86
4.84
4.84
4.83
4.82
4.81
3.70
3.11
3.09
3.07
3.06
3.04
3.02
3.00
2.99
2.44
1.96
0.10
2.05
2.04
0.99
1.00
3.07
2.05
3.09
3.05
f1 (ppm)

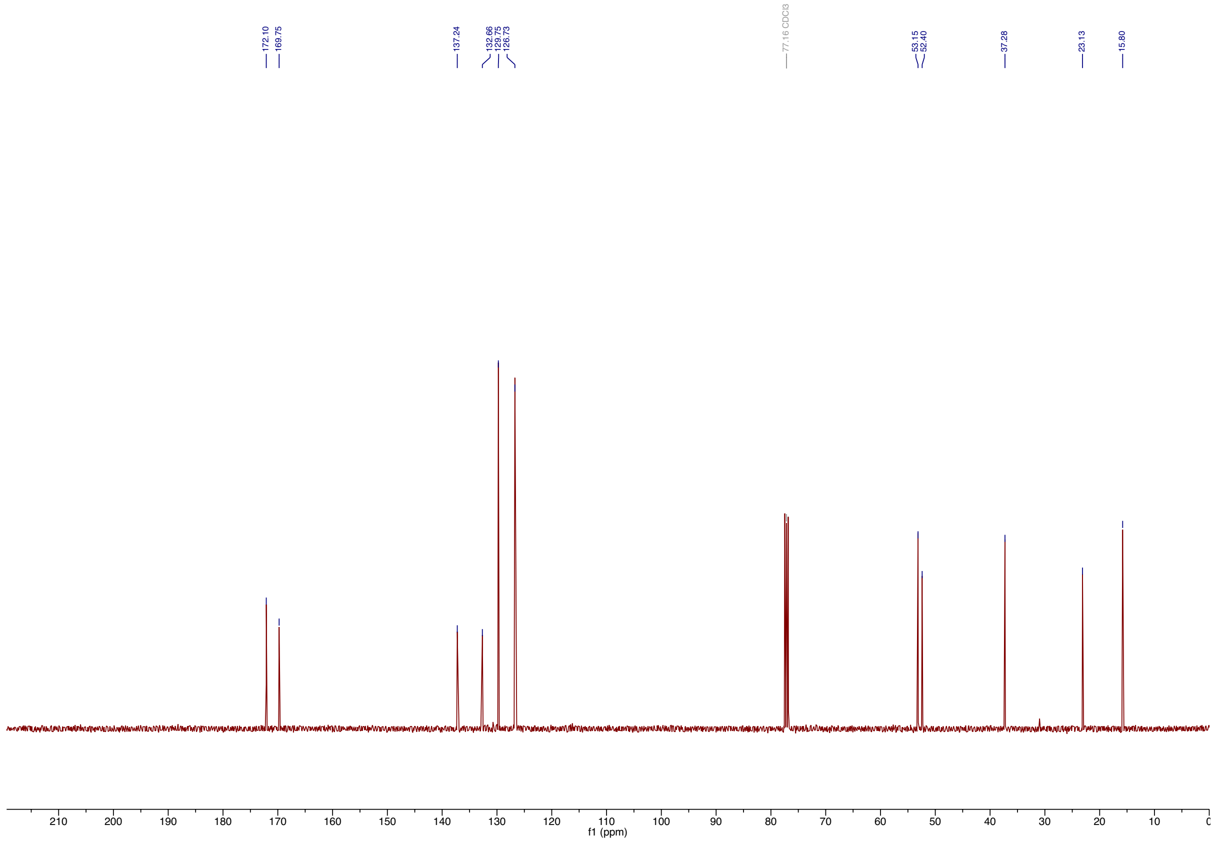
172.10
169.75
137.24
132.66
129.75
126.73
77.16 CDCl3
53.15
52.40
37.28
23.13
15.80
f1 (ppm)

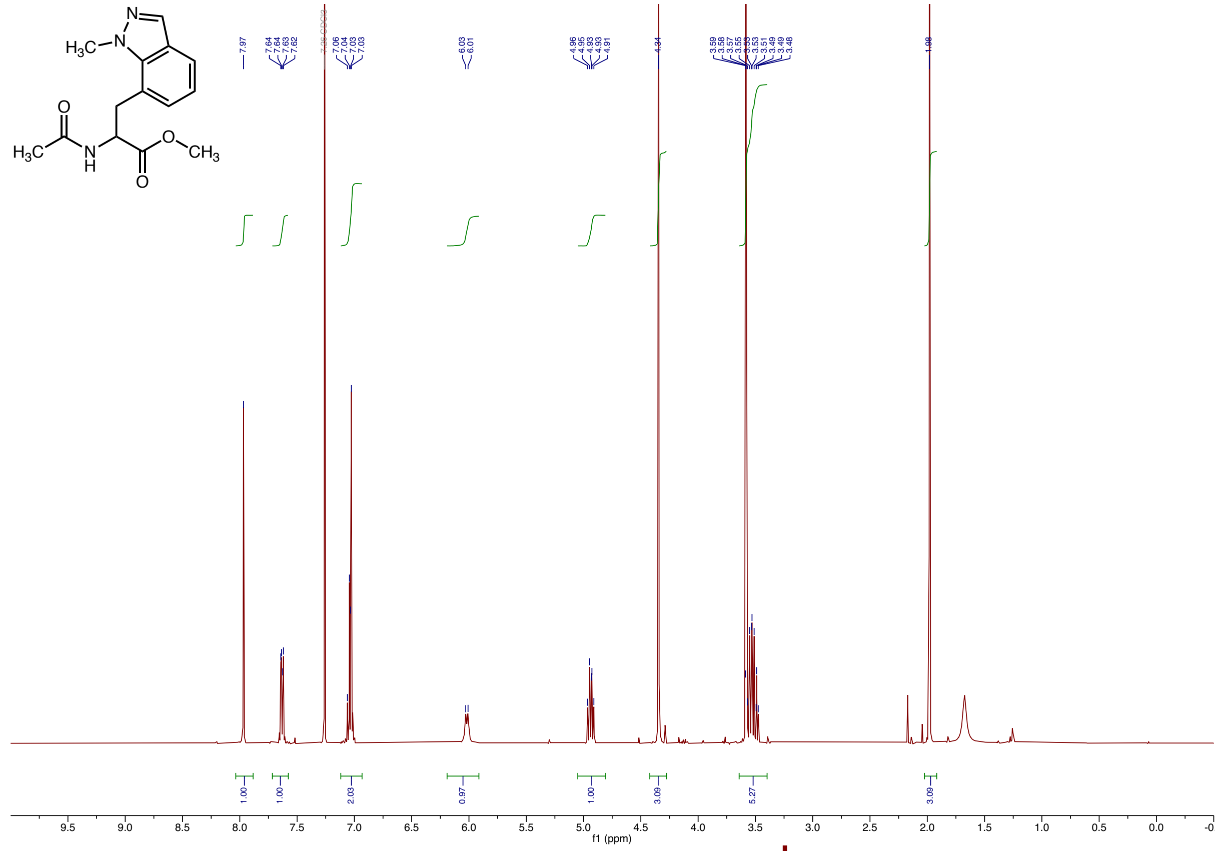
N
N
H_3C
O
H_3C
N
H
O
CH_3
O
7.97
7.64
7.64
7.63
7.62
7.06
7.04
7.03
7.03
6.03
6.01
4.96
4.95
4.93
4.93
4.91
4.34
3.59
3.58
3.57
3.55
3.53
3.53
3.51
3.49
3.49
3.48
1.98
1.00
1.00
2.03
0.97
1.00
3.09
5.27
3.09
f1 (ppm)

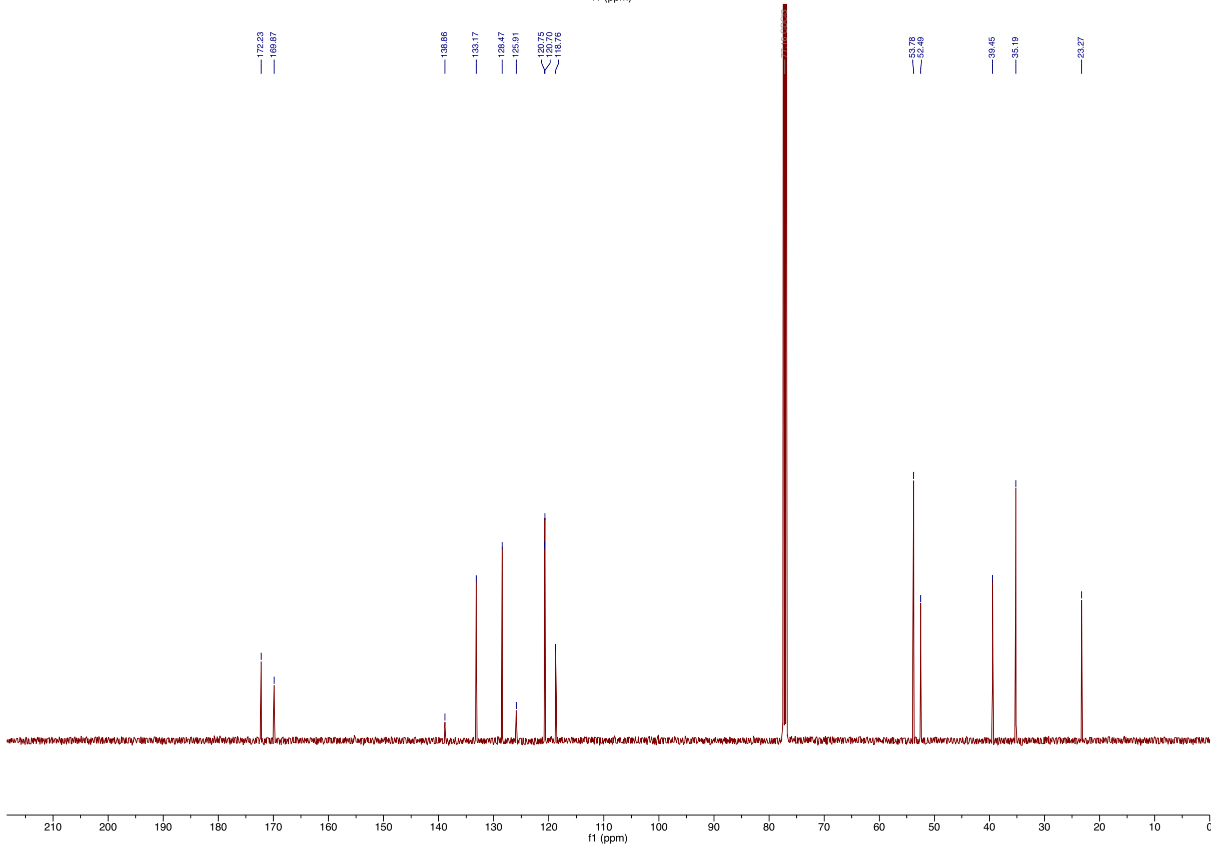
172.23
169.87
138.86
133.17
128.47
125.91
120.75
120.70
118.76
53.78
52.49
39.45
35.19
23.27
f1 (ppm)

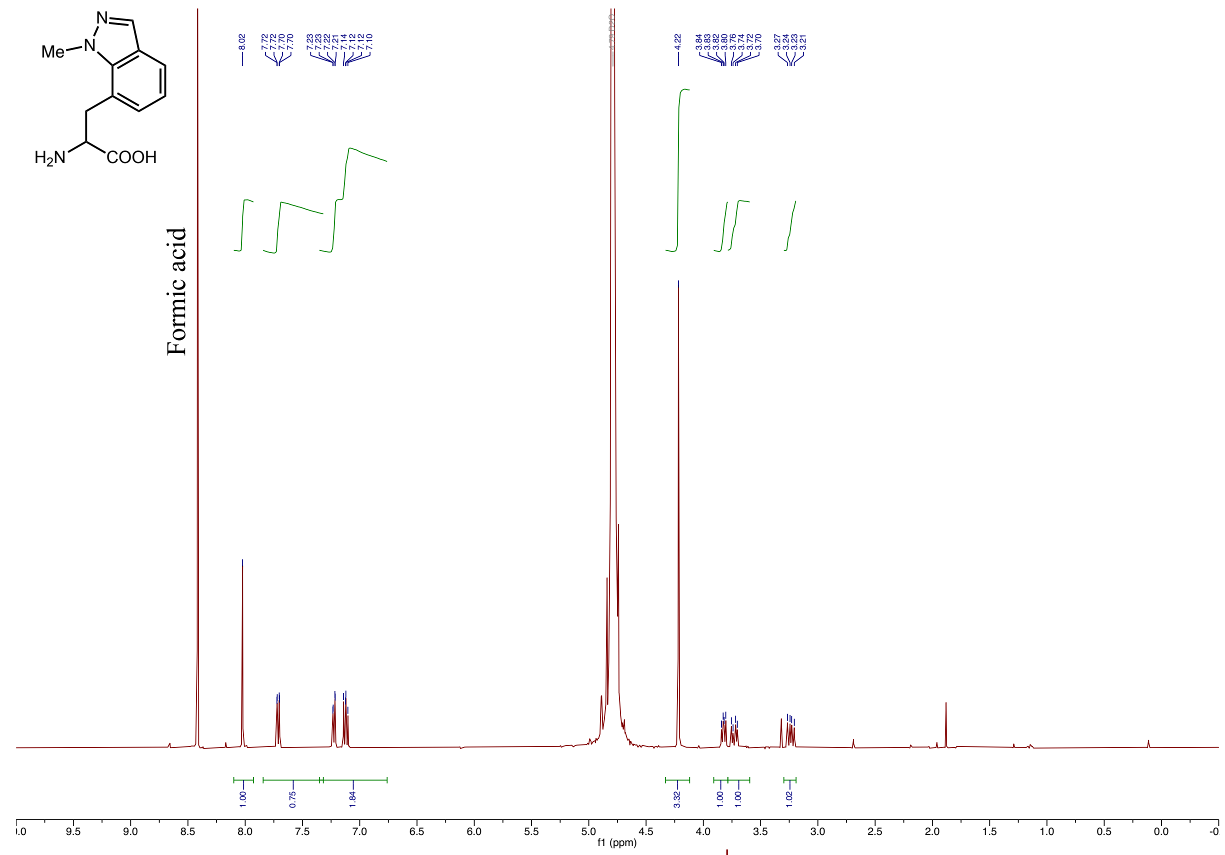
Formic acid
Me
N
N
H2N
COOH
f1 (ppm)

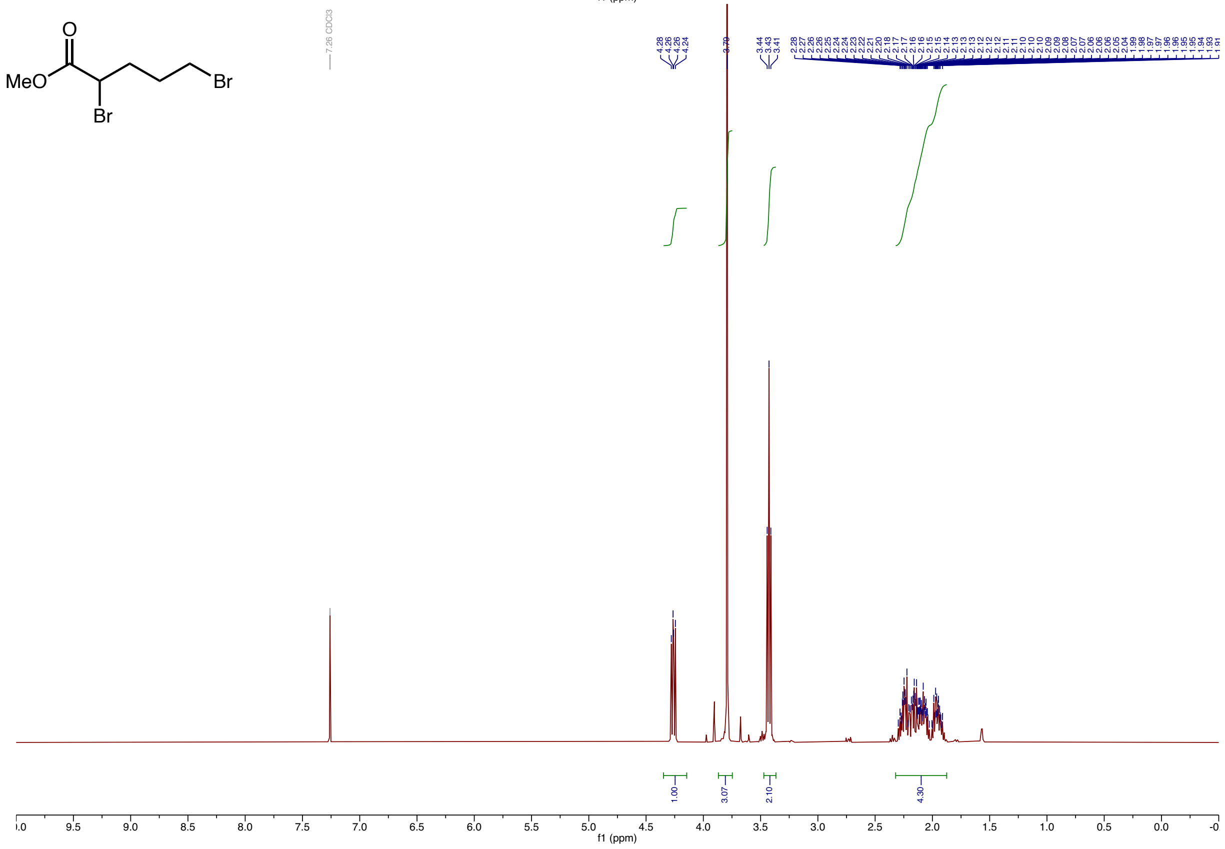
MeO
O
Br
Br
7.26 CDCl3
f1 (ppm)

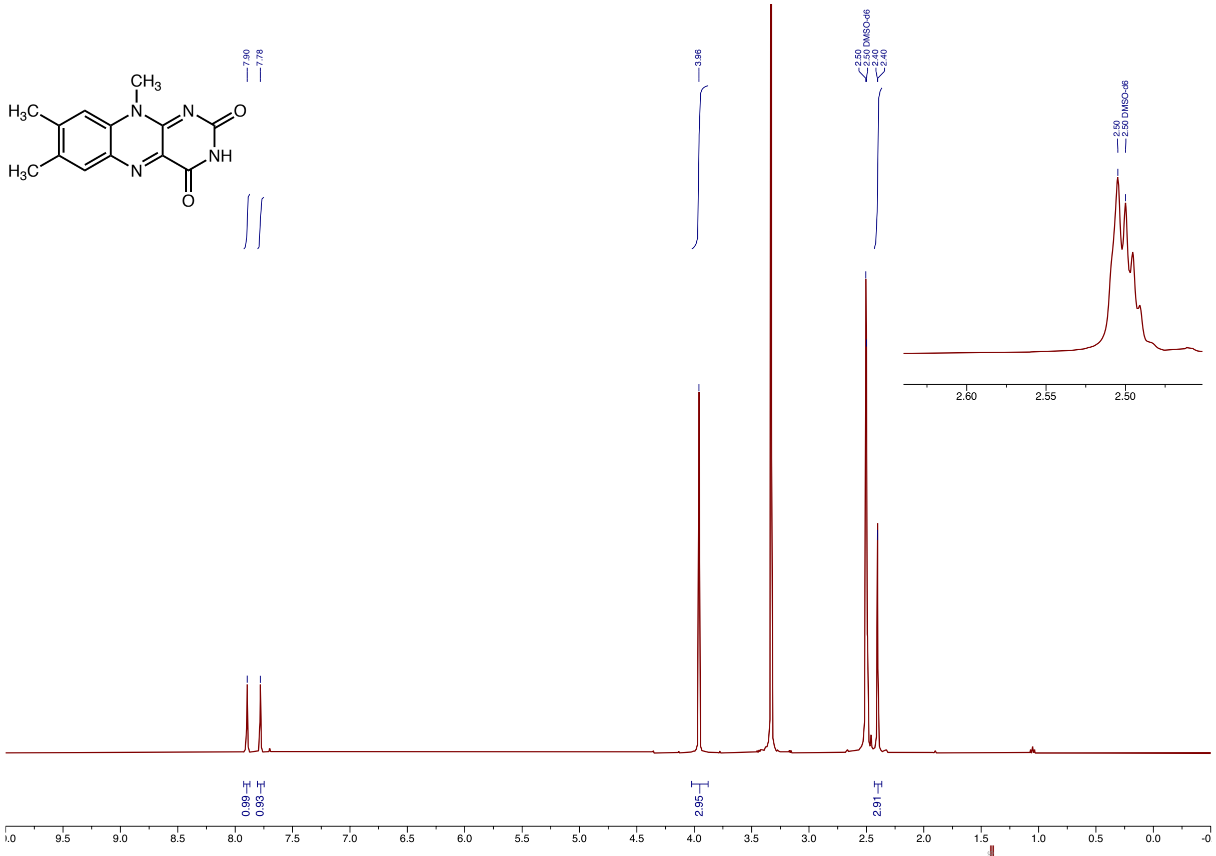

CH3
H3C
H3C
N
N
O
NH
N
O
7.90
7.78
3.96
2.50
2.50 DMSO-d6
2.40
2.40
2.50
2.50 DMSO-d6
2.60
2.55
2.50
0.99
0.93
2.95
2.91

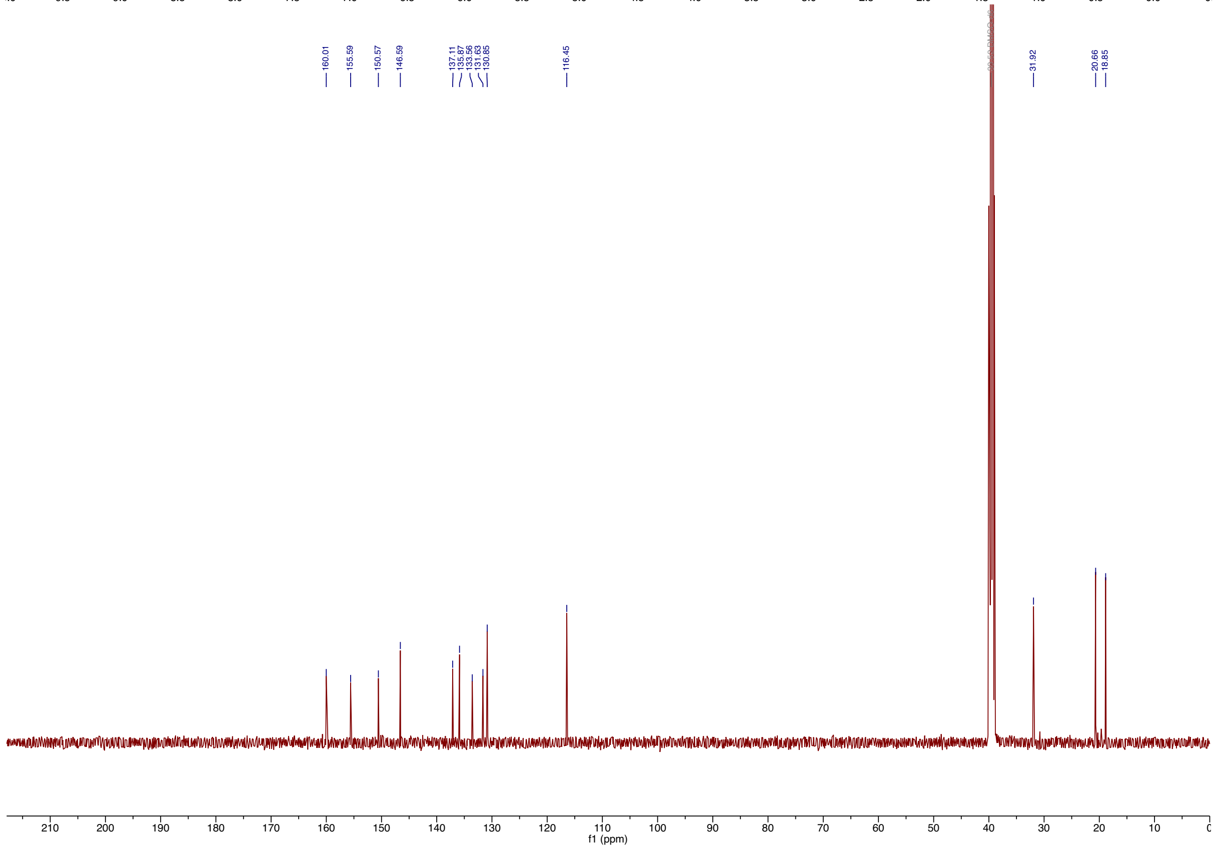

160.01
155.59
150.57
146.59
137.11
135.87
133.56
131.63
130.85
116.45
31.92
20.66
18.85
f1 (ppm)

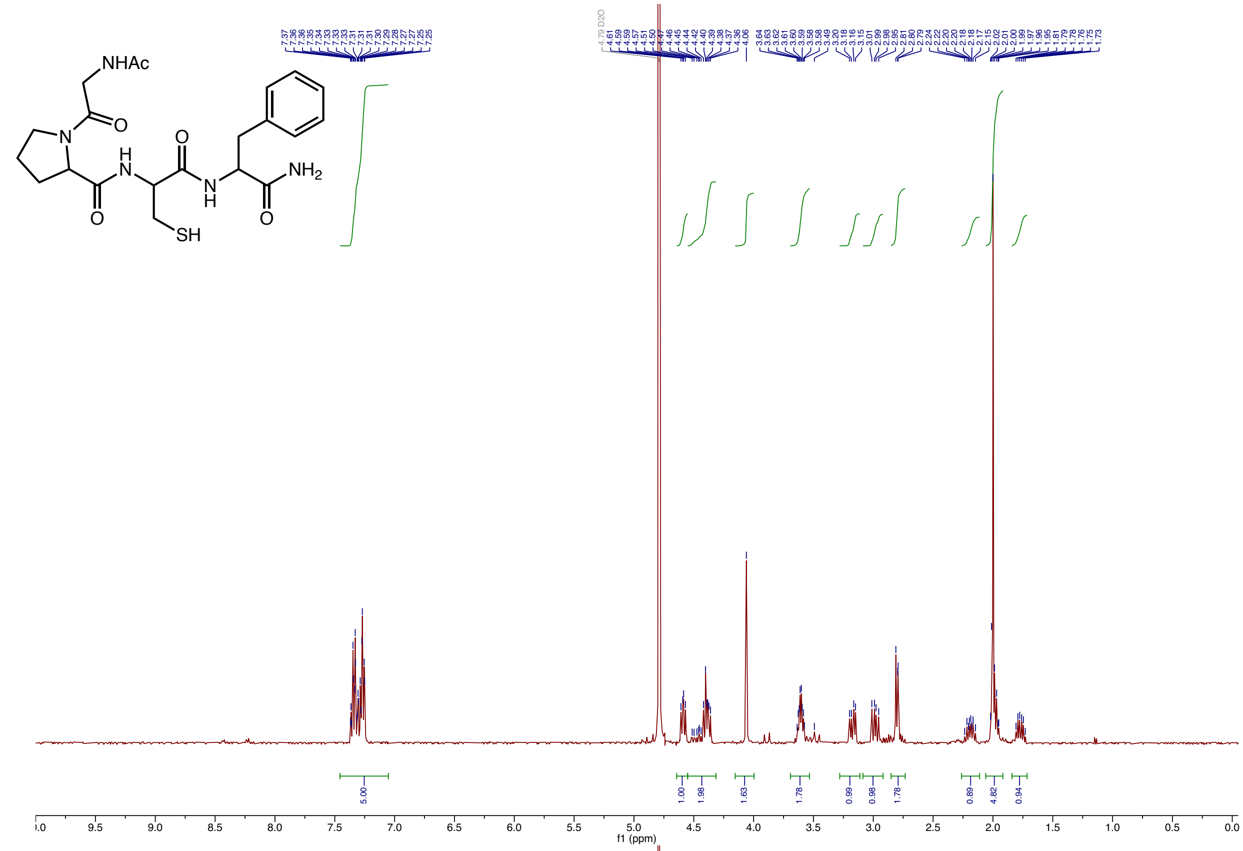
NHAc
SH
f1 (ppm)

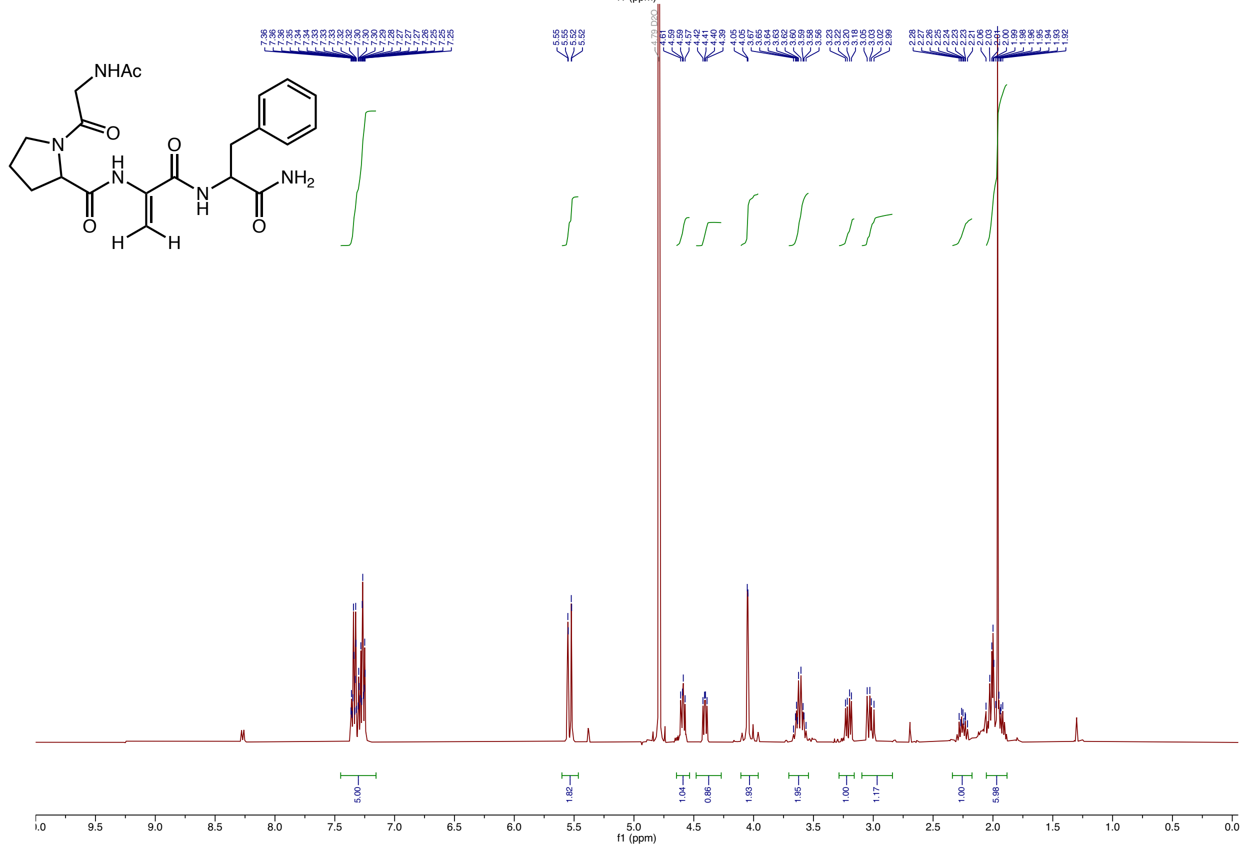
NHAc
f1 (ppm)

Peptide Selectivity

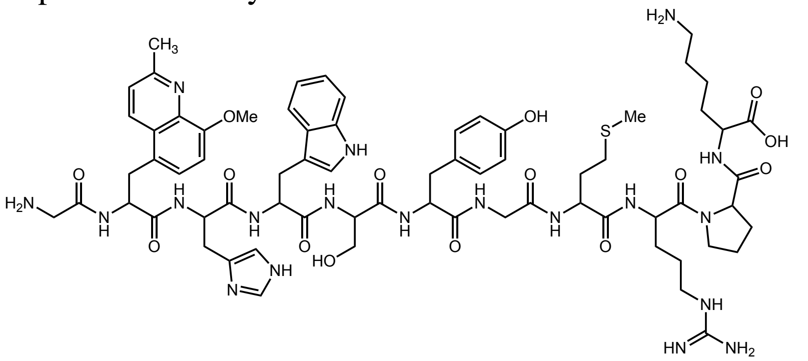

H_2N-Gly-**[(8-MeO-2-Me)-5-Qin]**-His-Trp-Ser-Tyr-Gly-Met-Arg-Pro-Lys-CO_2H

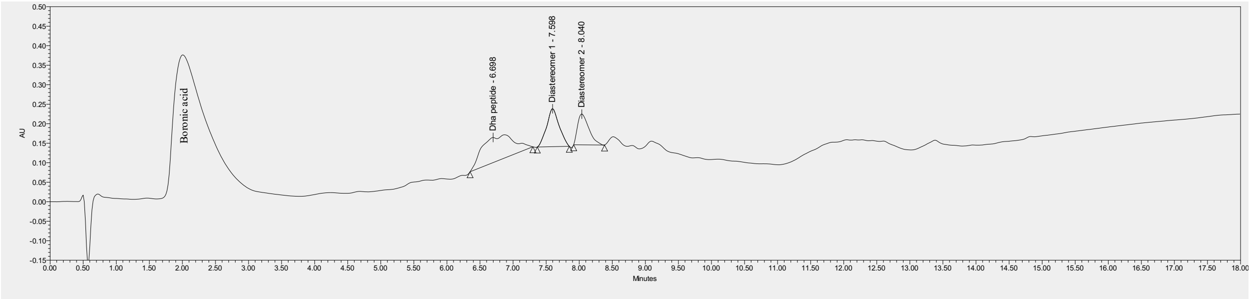

	Name	Retention Time	Area	% Area
1	Dha peptide	6.698	2168823	49.35
2	Diastereomer 1	7.598	1287871	29.31
3	Diastereomer 2	8.040	937734	21.34

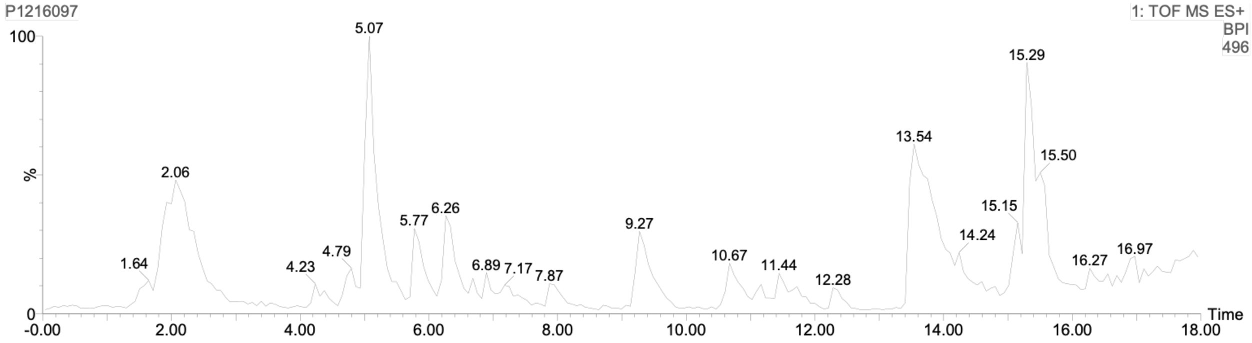

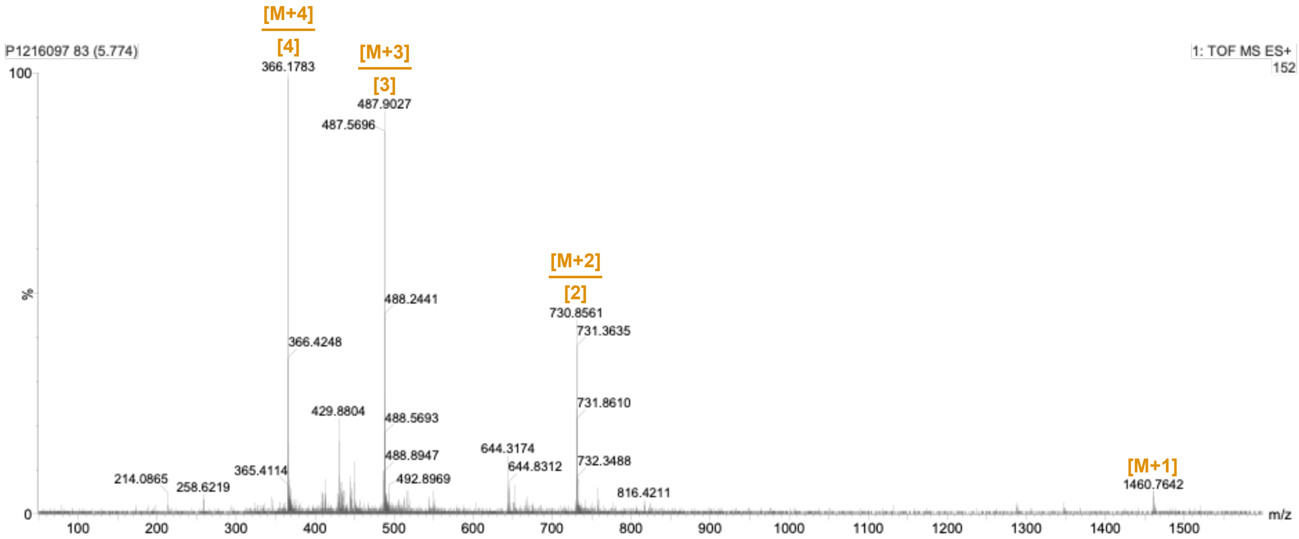
P1216097 83 (5.774)
1: TOF MS ES+
152
[M+4]
[4]
366.1783
[M+3]
[3]
487.9027
487.5696
[M+2]
[2]
730.8561
731.3635
488.2441
366.4248
731.8610
429.8804
488.5693
644.3174
488.8947
644.8312
732.3488
365.4114
214.0865
258.6219
492.8969
816.4211
[M+1]
1460.7642
%
m/z

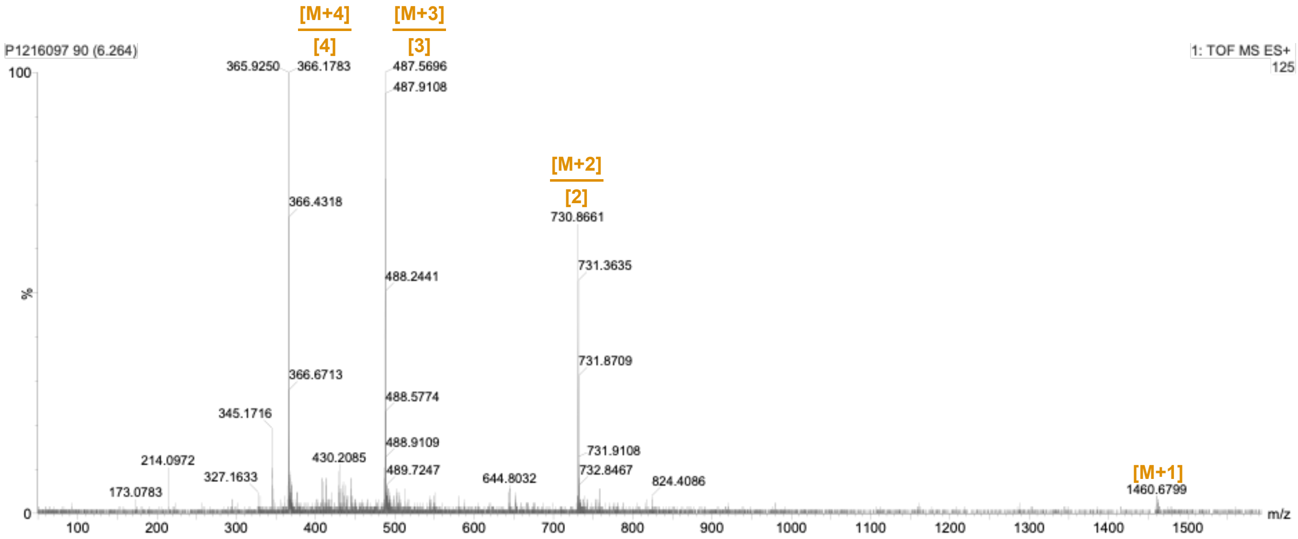
P1216097 90 (6.264)
1: TOF MS ES+
125
[M+4]
[4]
365.9250
366.1783
[M+3]
[3]
487.5696
487.9108
[M+2]
[2]
730.8661
366.4318
731.3635
488.2441
731.8709
366.6713
488.5774
345.1716
488.9109
731.9108
214.0972
430.2085
489.7247
732.8467
327.1633
644.8032
824.4086
173.0783
[M+1]
1460.6799
%
m/z

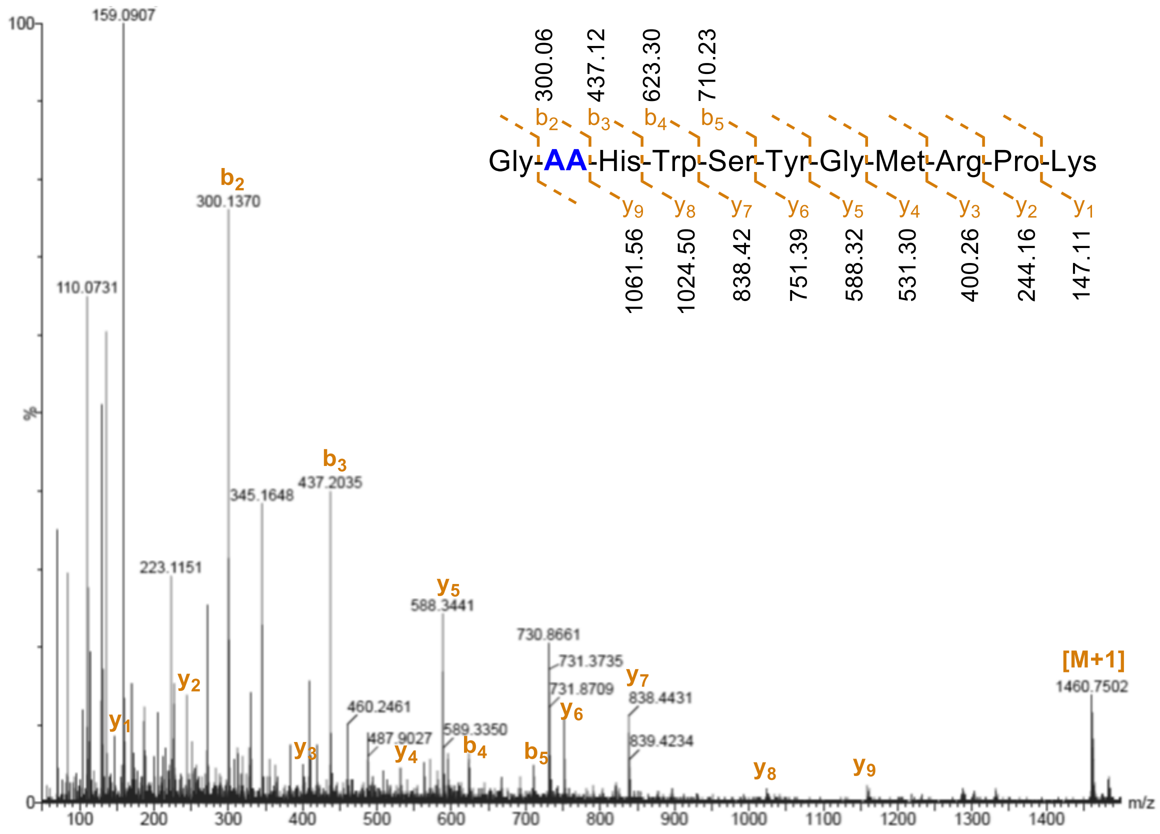

Gly-AA-His-Trp-Ser-Tyr-Gly-Met-Arg-Pro-Lys
b2 b3 b4 b5
300.06 437.12 623.30 710.23
y9 y8 y7 y6 y5 y4 y3 y2 y1
1061.56 1024.50 838.42 751.39 588.32 531.30 400.26 244.16 147.11
159.0907
b2 300.1370
110.0731
b3 437.2035
345.1648
223.1151
y5 588.3441
730.8661
731.3735
731.8709
[M+1] 1460.7502
y7 838.4431
839.4234
460.2461
487.9027
589.3350
y1 y2 y3 y4 b4 b5 y6 y8 y9
%
m/z

Plate Data

Liquid Chromatography analyses were performed on a Waters Acquity H-Class UPLC. Mass Spectrometry analyses were performed on a Quatro Ultima triple quadrupole mass analyzer with an electrospray ion source connected through column (without UV). The same column was used on both instruments, Waters Zorbax StableBondC18 3.5 µm, 2.1 x 100 mm column, but the elution times did not match exactly.

Method: 0.5 mL/min flow rate. A = Acetonitrile with 1% formic acid modifier, B = ddH_2O with 1% formic acid modifier.

Time:	%A:	%B:
0.0	5.0	95.0
2.0	5.0	95.0
3.0	10.0	90.0
10.0	20.0	80.0
12.0	30.0	70.0
16.5	95.0	5.0
18.0	95.0	5.0

Table S10. 96-well plate results

Peptide:	MW:	Purity:	Amount:	Yield:
1A'	538.6	77.0%	0.096 mg	7.7%
2A'	556.6	51.6%	0.130 mg	10.0%
3A'	556.6	36.3%	0.107 mg	8.3%
4A'	614.7	36.3%	0.060 mg	4.2%
5A'	572.7	95.0%	0.206 mg	15.4%
6A'	566.7	91.7%	0.319 mg	24.2%
7A'	538.6	64.8%	0.217 mg	17.3%
8A'	568.6	81.1%	0.237 mg	17.9%
9A'	538.6	17.4%	0.035 mg	2.8%
10A'	556.6	61.9%	0.197 mg	15.2%
11A'	623.7	96.2%	0.163 mg	11.2%
12A'	554.7	85.5%	0.280 mg	21.6%
1B'	552.6	68.8%	0.226 mg	17.5%
2B'	552.6	46.1%	0.184 mg	14.3%
3B'	558.6	56.9%	0.043 mg	3.3%
4B'	558.6	91.1%	0.126 mg	9.7%

5B'	572.7	89.0%	0.193 mg	14.4%
6B'	602.7	78.2%	0.208 mg	14.8%
7B'	558.6	92.0%	0.217 mg	16.7%
8B'	558.6	92.3%	0.375 mg	28.8%
9B'	558.6	90.4%	0.254 mg	19.5%
10B'	558.6	73.6%	0.078 mg	6.0%
11B'	552.6	46.0%	0.059 mg	4.6%
12B'	555.6	42.8%	0.052 mg	4.0%
1C'	601.7	86.1%	0.078 mg	5.5%
2C'	569.6	41.7%	0.036 mg	2.7%
3C'	560.7	89.0%	0.047 mg	3.6%
4C'	546.6	77.8%	0.083 mg	6.5%
5C'	546.6	54.2%	0.123 mg	9.6%
6C'	547.6	62.0%	0.080 mg	6.2%
7C'	547.6	77.9%	0.162 mg	12.7%
8C'	561.6	69.2%	0.433 mg	33.1%
9C'	561.6	84.6%	0.311 mg	23.8%
10C'	561.6	74.9%	0.186 mg	14.2%
11C'	561.6	88.2%	0.230 mg	17.5%
12C'	548.6	57.3%	0.038 mg	3.0%
1D'	548.6	11.5%	0.003 mg	0.2%
2D'	561.6	66.5%	0.039 mg	3.0%
3D'	564.7	55.7%	0.066 mg	5.0%
4D'	563.7	17.1%	0.010 mg	0.7%
5D'	563.7	59.6%	0.021 mg	1.6%
6D'	565.6	75.0%	0.246 mg	18.7%
7D'	551.6	82.8%	0.216 mg	16.8%
8D'	497.6	35.3%	0.015 mg	1.3%
9D'	497.6	28.3%	0.025 mg	2.1%
10D'	511.6	5.2%	0.004 mg	0.3%
11D'	513.6	68.8%	0.090 mg	7.5%
12D'	513.6	48.4%	0.038 mg	3.2%
1E'	521.6	58.4%	0.072 mg	5.9%
2E'	535.7	45.8%	0.196 mg	15.7%
3E'	547.6	63.9%	0.042 mg	3.3%
4E'	563.7	46.7%	0.018 mg	1.4%
5E'	553.7	78.7%	0.228 mg	17.7%
6E'	537.6	84.9%	0.281 mg	22.4%
7E'	537.6	79.6%	0.178 mg	14.2%
8E'	537.6	79.5%	0.222 mg	17.7%

9E'	567.6	79.2%	0.296 mg	22.4%
10E'	567.6	88.7%	0.308 mg	23.3%
11E'	567.6	65.2%	0.086 mg	6.5%
12E'	621.7	59.1%	0.087 mg	6.0%
1F'	555.6	76.0%	0.189 mg	14.6%
2F'	613.7	27.0%	0.010 mg	0.7%
3F'	597.7	81.7%	0.614 mg	44.1%
4F'	599.7	37.2%	0.012 mg	0.8%
5F'	597.7	24.9%	0.019 mg	1.3%
6F'	535.7	21.0%	0.087 mg	6.9%
7F'	564.6	65.7%	0.186 mg	14.1%
8F'	622.7	82.8%	0.305 mg	21.0%
9F'	583.7	53.4%	0.017 mg	1.2%
10F'	584.7	78.8%	0.218 mg	16.0%
11F'	557.7	72.8%	0.044 mg	3.4%
12F'	557.7	86.1%	0.084 mg	6.5%
1G'	551.6	87.9%	0.297 mg	23.1%
2G'	521.6	73.5%	0.057 mg	4.7%
3G'	573.6	76.9%	0.134 mg	10.0%
4G'	555.6	71.7%	0.149 mg	11.5%
5G'	553.7	82.4%	0.454 mg	35.2%
6G'	633.7	86.7%	0.262 mg	17.8%
7G'	581.6	71.2%	0.056 mg	4.2%
8G'	535.6	80.6%	0.158 mg	12.7%
9G'	551.6	67.7%	0.133 mg	10.4%
10G'	572.7	66.2%	0.095 mg	7.1%
11G'	565.7	78.9%	0.228 mg	17.3%
12G'	581.7	69.2%	0.376 mg	27.7%
1H'	529.6	81.1%	0.192 mg	15.6%
2H'	551.6	49.7%	0.112 mg	8.7%
3H'	517.6	38.1%	0.124 mg	10.3%
4H'	600.7	69.7%	0.178 mg	12.7%
5H	543.7	12.4%	0.029 mg	2.3%
6H'	491.6	51.1%	0.193 mg	16.8%
7H'	600.7	35.2%	0.042 mg	3.0%
8H'	485.6	50.9%	0.161 mg	14.2%
9H'	586.7	28.5%	0.112 mg	8.2%
10H'	473.6	68.9%	0.316 mg	28.7%
11H'	513.6	75.1%	0.156 mg	13.1%
12H'	487.6	53.6%	0.090 mg	7.9%

1A’: MW = 538.6 g/mol, Purity = 77.0%, Yield = 7.7% [0.096 mg]

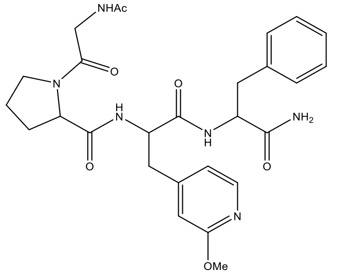

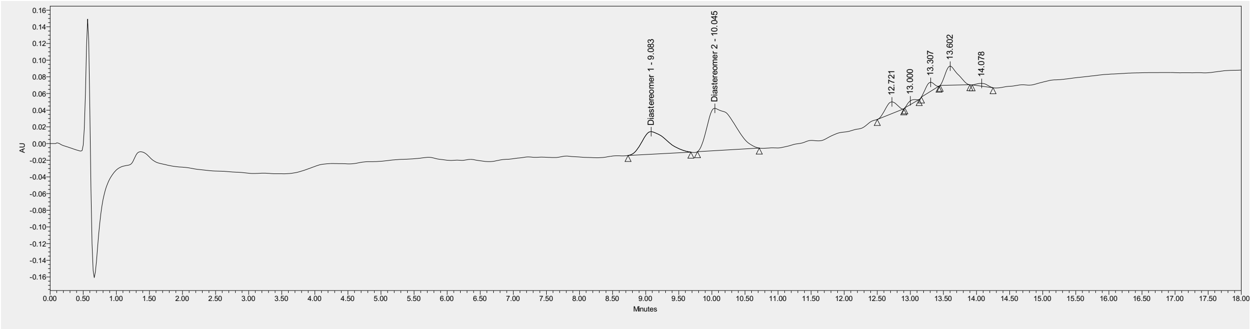

	Name	Retention Time	Area	% Area
1	Diastereomer 1	9.083	708389	25.47
2	Diastereomer 2	10.045	1432789	51.51
3		12.721	170064	6.11
4		13.000	35460	1.27
5		13.307	97753	3.51
6		13.602	300746	10.81
7		14.078	36556	1.31

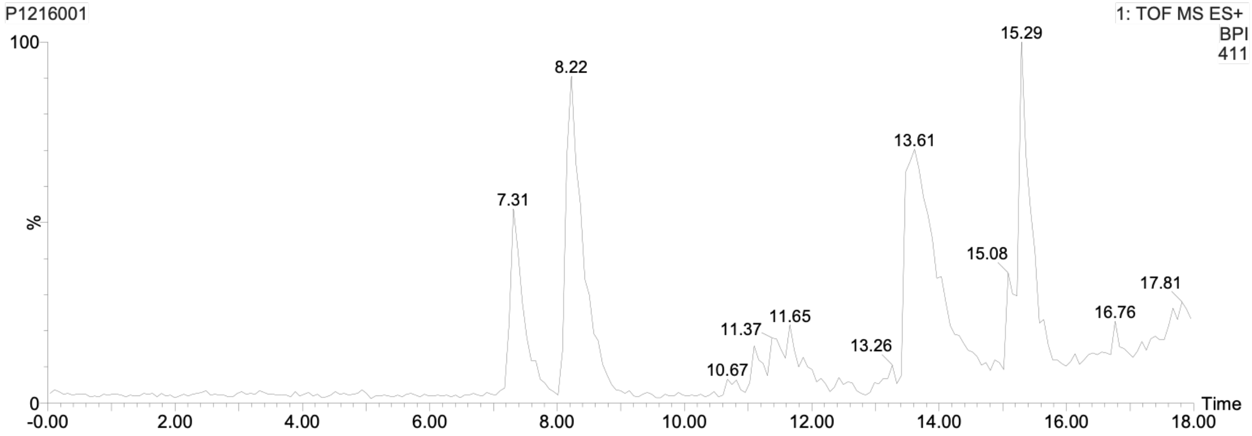

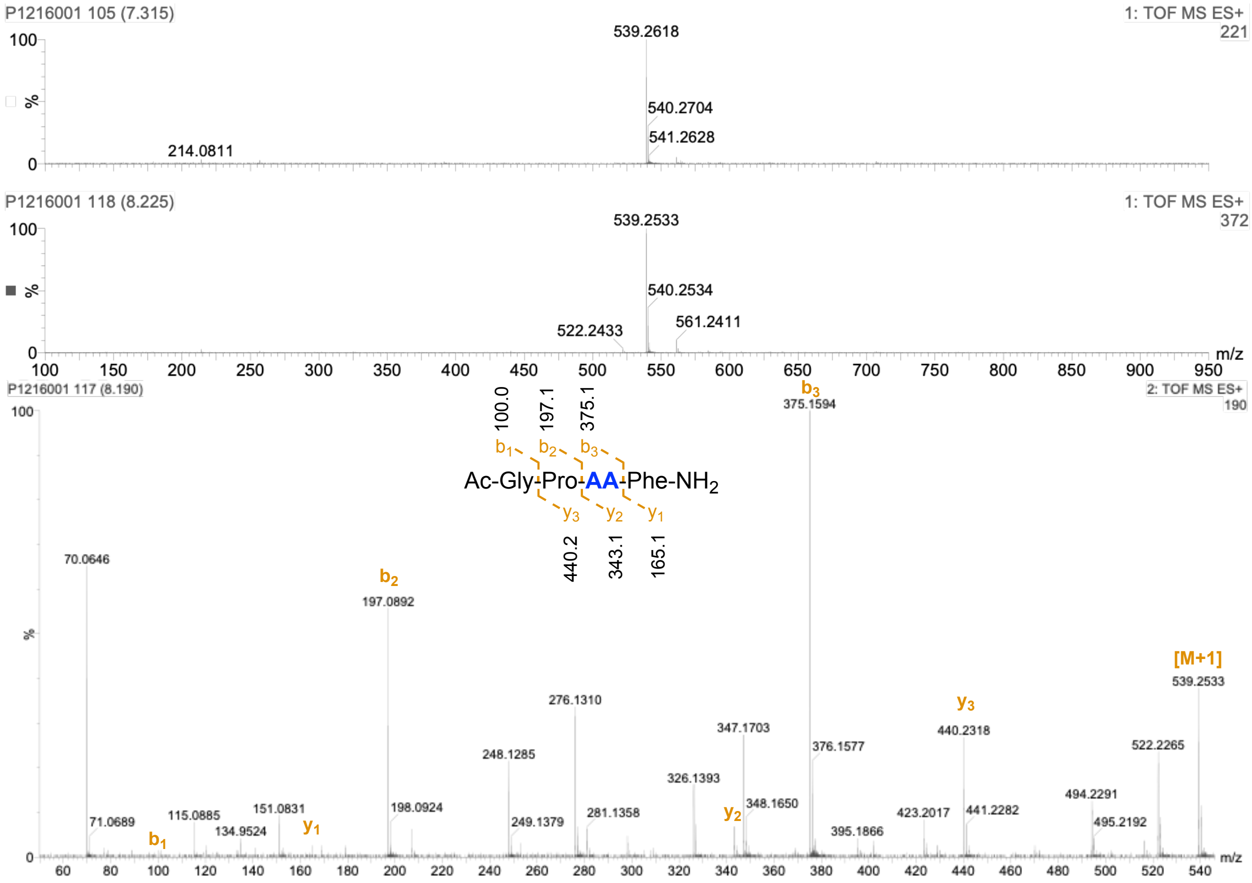
P1216001 105 (7.315)
1: TOF MS ES+
221
539.2618
540.2704
541.2628
214.0811
P1216001 118 (8.225)
1: TOF MS ES+
372
539.2533
540.2534
561.2411
522.2433
m/z
P1216001 117 (8.190)
2: TOF MS ES+
190
Ac-Gly-Pro-AA-Phe-NH2
100.0
197.1
375.1
440.2
343.1
165.1
b3
375.1594
70.0646
b2
197.0892
[M+1]
539.2533
276.1310
y3
440.2318
347.1703
376.1577
522.2265
248.1285
326.1393
494.2291
71.0689
115.0885
151.0831
198.0924
249.1379
281.1358
348.1650
395.1866
423.2017
441.2282
495.2192
134.9524
b1
y1
y2
m/z

2A’: MW = 556.6, Purity = 51.6% , Yield = 10.0% [0.13 mg]

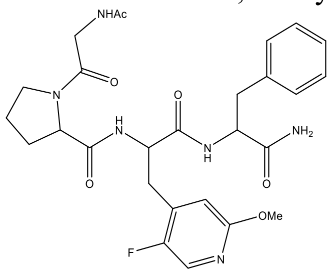

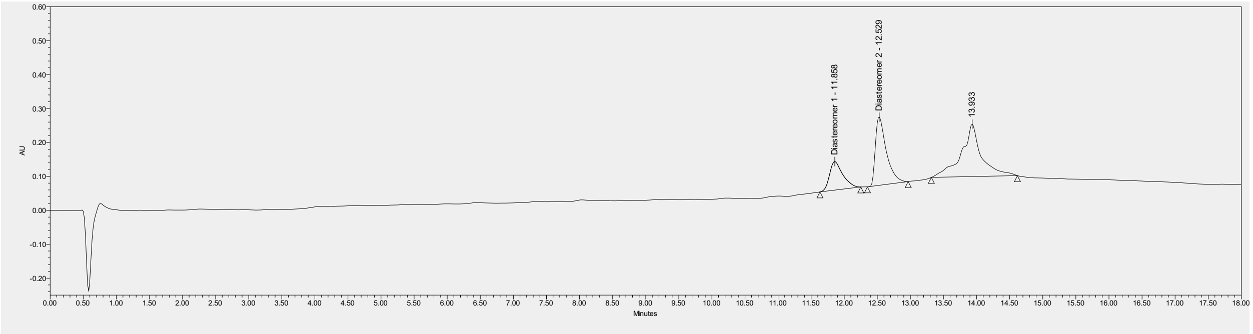

	Name	Retention Time	Area	% Area
1	Diastereomer 1	11.858	1167576	17.31
2	Diastereomer 2	12.529	2314842	34.31
3		13.933	3264156	48.38

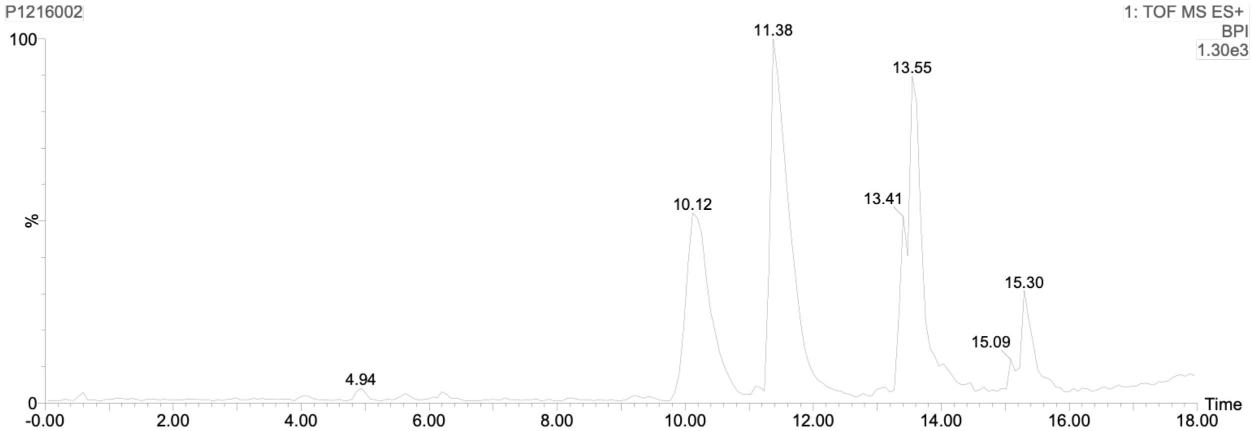

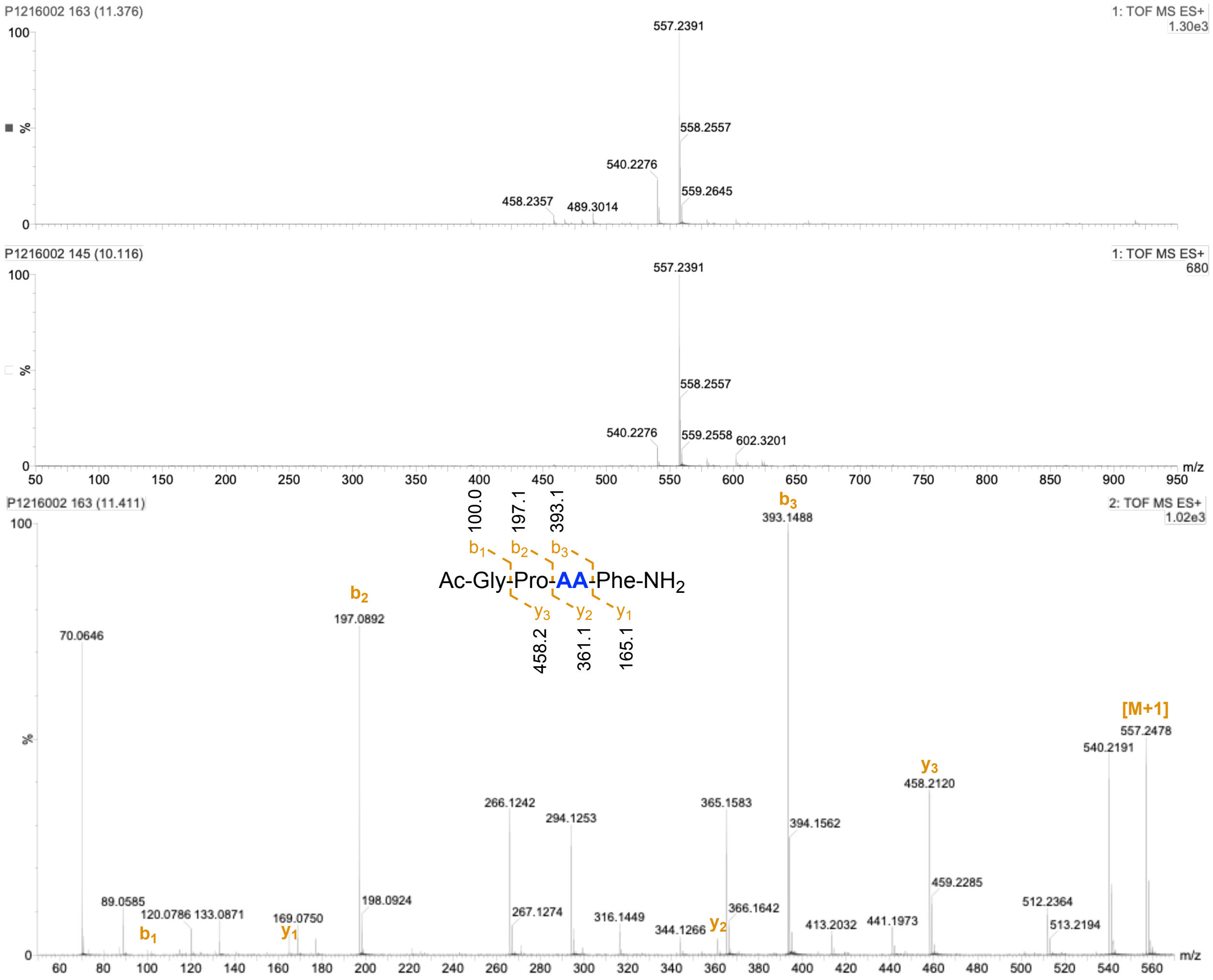
P1216002 163 (11.376)
1: TOF MS ES+
1.30e3
557.2391
558.2557
540.2276
559.2645
458.2357
489.3014
P1216002 145 (10.116)
1: TOF MS ES+
680
557.2391
558.2557
540.2276
559.2558
602.3201
m/z
P1216002 163 (11.411)
2: TOF MS ES+
1.02e3
Ac-Gly-Pro-AA-Phe-NH2
b3
393.1488
b2
197.0892
70.0646
[M+1]
557.2478
540.2191
y3
458.2120
266.1242
294.1253
365.1583
394.1562
459.2285
89.0585
120.0786
133.0871
169.0750
198.0924
267.1274
316.1449
344.1266
366.1642
413.2032
441.1973
512.2364
513.2194
b1
y1
y2
m/z

3A’: MW = 556.6, Purity = 36.3%, Yield = 8.3% [0.11 mg]

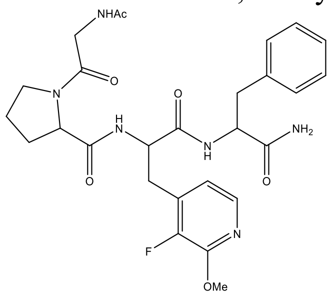

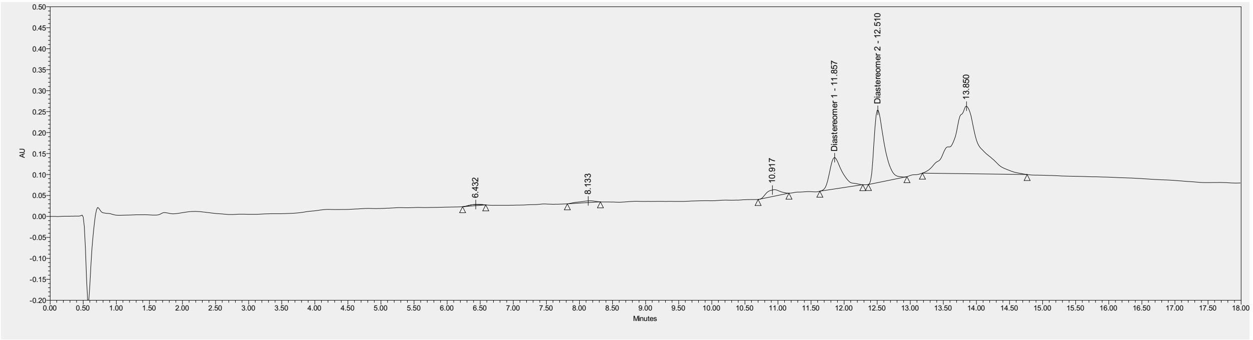

	Name	Retention Time	Area	% Area
1		6.432	35074	0.44
2		8.133	72544	0.91
3		10.917	226520	2.85
4	Diastereomer 1	11.857	1003370	12.62
5	Diastereomer 2	12.510	1878973	23.63
6		13.850	4736735	59.56

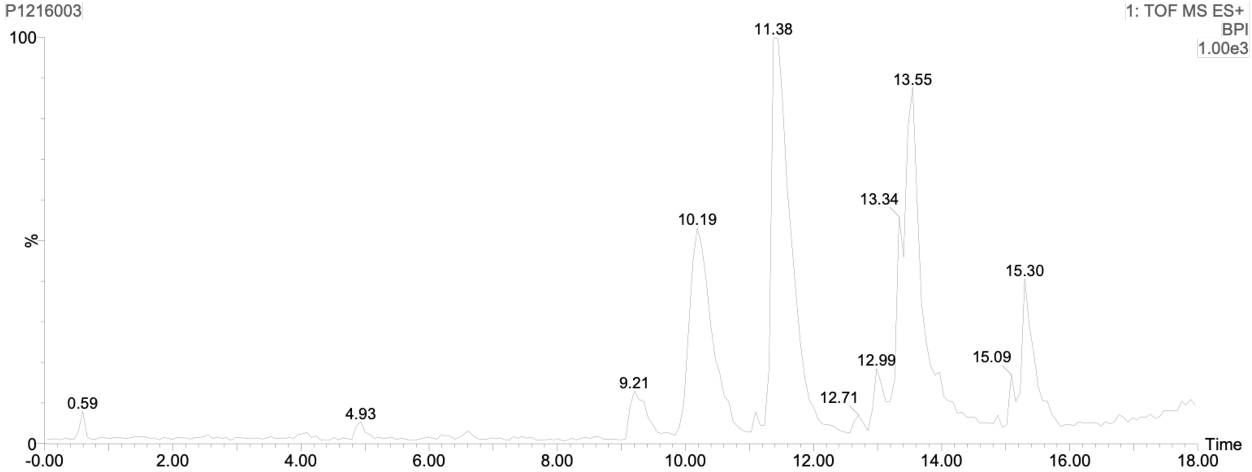

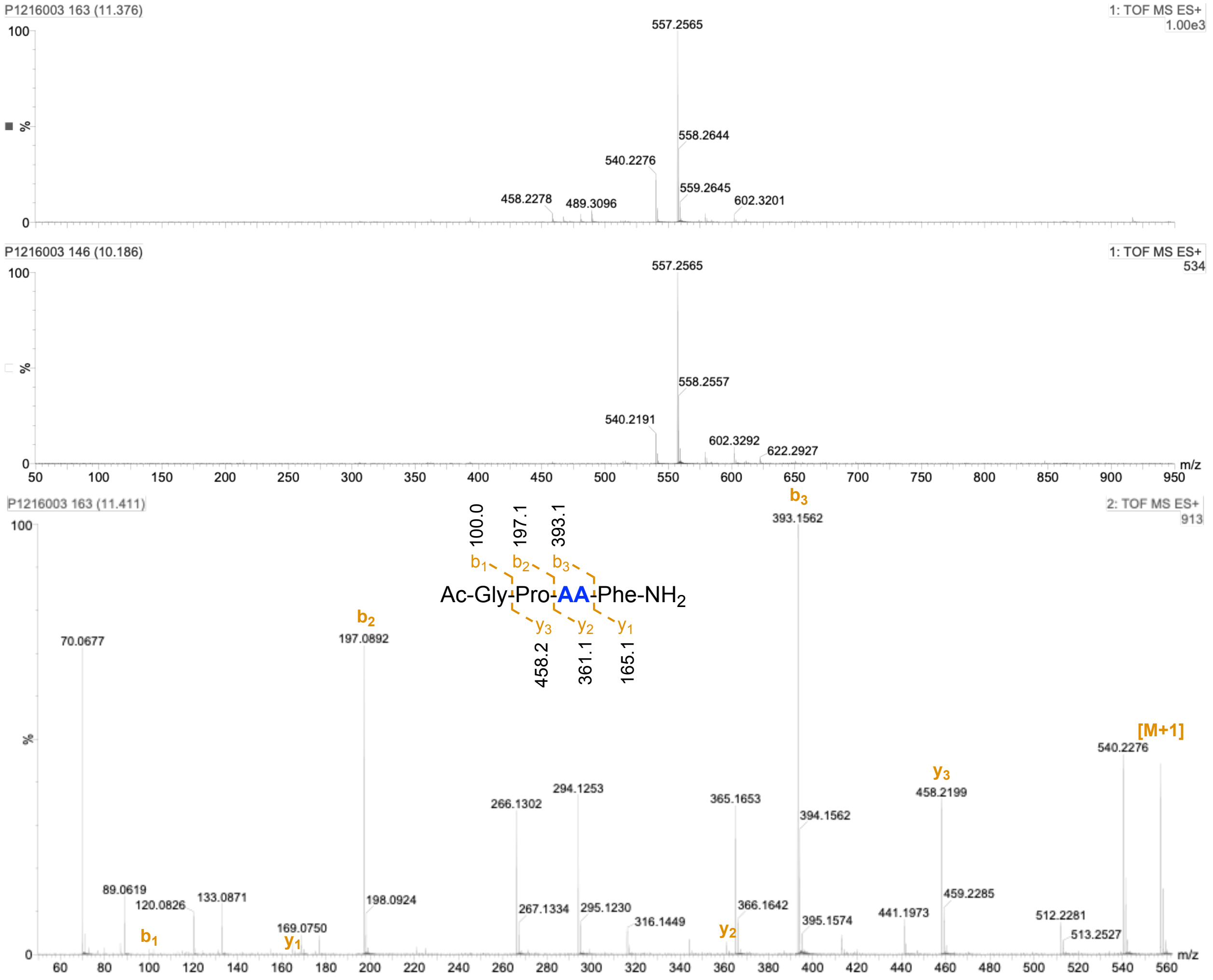

P1216003 163 (11.376)
1: TOF MS ES+
1.00e3
100
%
0
557.2565
558.2644
540.2276
559.2645
602.3201
458.2278
489.3096
P1216003 146 (10.186)
1: TOF MS ES+
534
557.2565
558.2557
540.2191
602.3292
622.2927
m/z
50 100 150 200 250 300 350 400 450 500 550 600 650 700 750 800 850 900 950
P1216003 163 (11.411)
2: TOF MS ES+
913
Ac-Gly-Pro-AA-Phe-NH2
b1 b2 b3
100.0 197.1 393.1
y3 y2 y1
458.2 361.1 165.1
b3
393.1562
b2
197.0892
70.0677
[M+1]
540.2276
y3
458.2199
266.1302
294.1253
365.1653
394.1562
89.0619
120.0826
133.0871
198.0924
169.0750
267.1334
295.1230
316.1449
366.1642
395.1574
441.1973
459.2285
512.2281
513.2527
b1
y1
y2
60 80 100 120 140 160 180 200 220 240 260 280 300 320 340 360 380 400 420 440 460 480 500 520 540 560

4A’: MW = 614.7, Purity = 36.3%, Yield = 4.2% [0.06 mg]

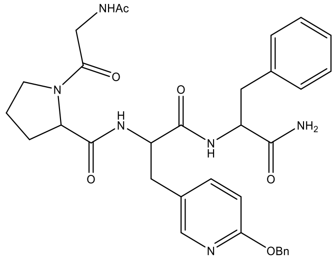

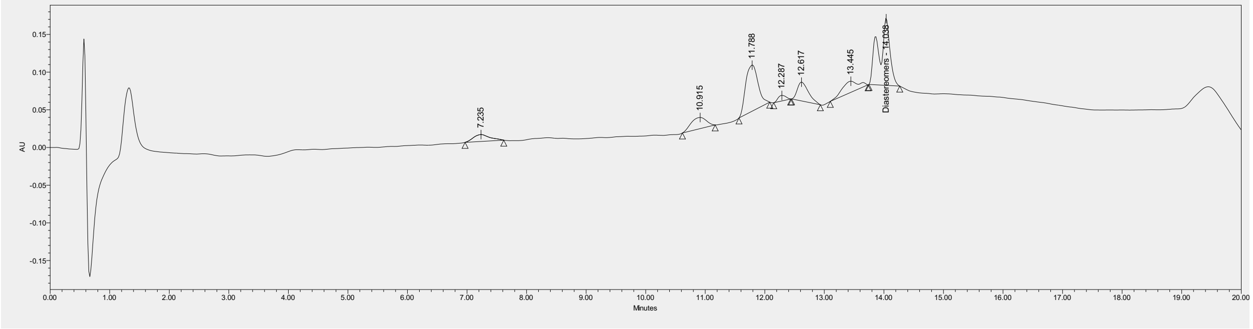

	Name	Retention Time	Area	% Area
1		7.235	176821	5.55
2		10.915	272606	8.56
3		11.788	896333	28.14
4		12.287	67824	2.13
5		12.617	326932	10.27
6		13.445	286984	9.01
7	Diastereomers	14.038	1157225	36.34

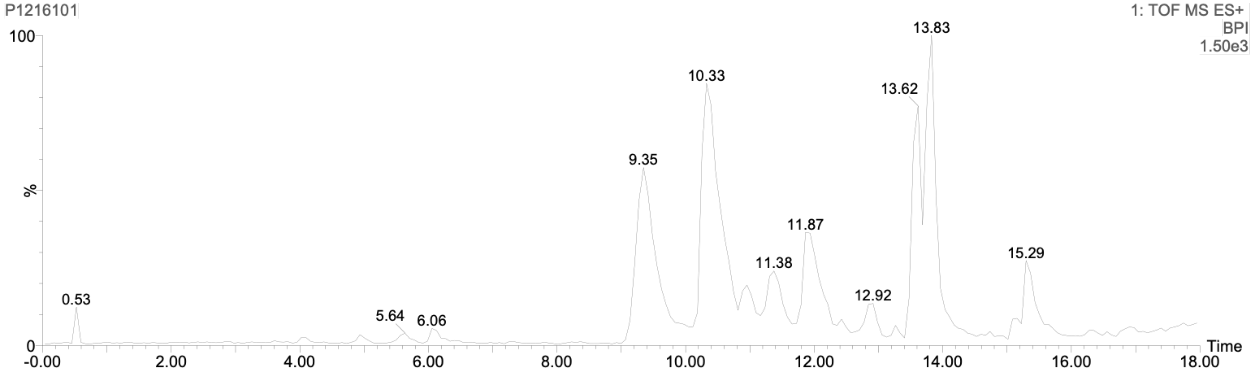

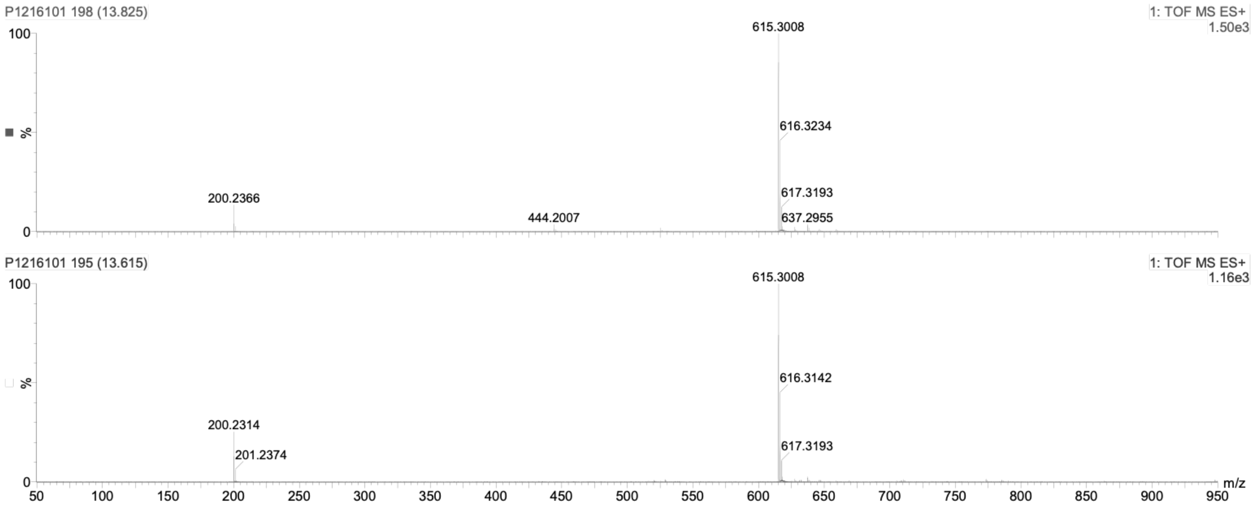
P1216101 198 (13.825)
1: TOF MS ES+
1.50e3
615.3008
616.3234
617.3193
637.2955
200.2366
444.2007
P1216101 195 (13.615)
1: TOF MS ES+
1.16e3
615.3008
616.3142
617.3193
200.2314
201.2374
m/z

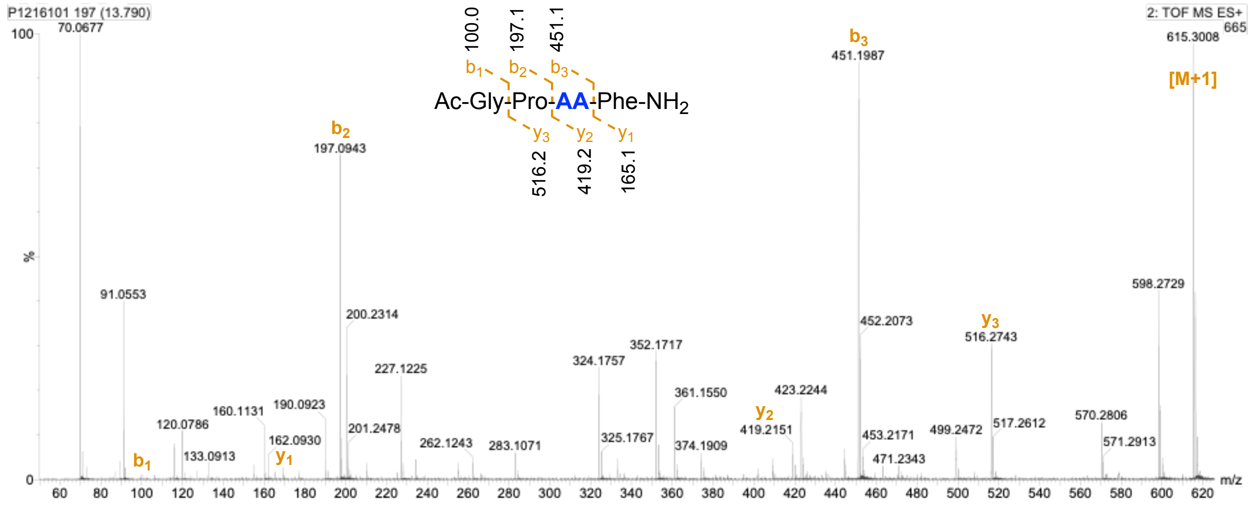
P1216101 197 (13.790)
2: TOF MS ES+
665
Ac-Gly-Pro-AA-Phe-NH2
100.0
197.1
451.1
516.2
419.2
165.1
b1
b2
b3
y1
y2
y3
[M+1]
615.3008
70.0677
91.0553
120.0786
133.0913
160.1131
162.0930
190.0923
197.0943
200.2314
201.2478
227.1225
262.1243
283.1071
324.1757
325.1767
352.1717
361.1550
374.1909
419.2151
423.2244
451.1987
452.2073
453.2171
471.2343
499.2472
516.2743
517.2612
570.2806
571.2913
598.2729
m/z

5A': MW = 572.7, Purity = 95.0% , Yield = 15.4% [0.21 mg]

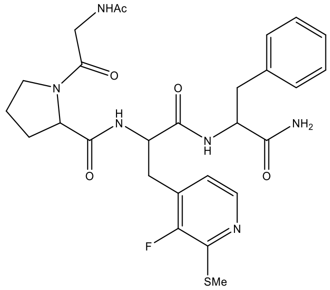

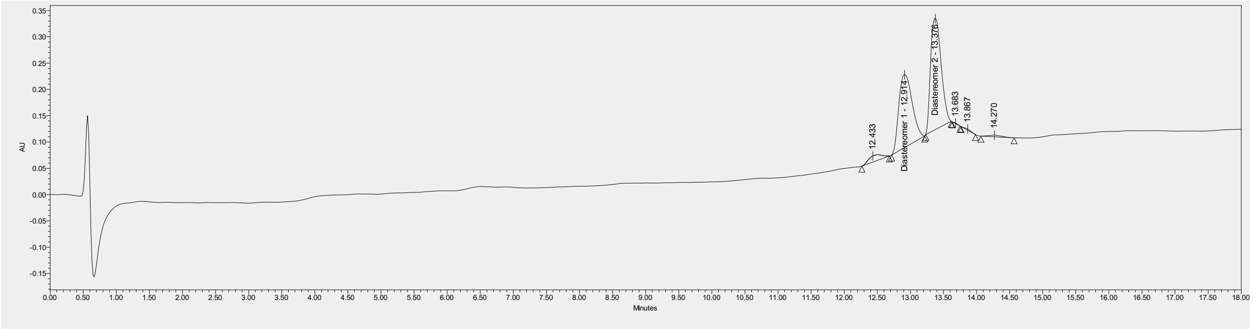

	Name	Retention Time	Area	% Area
1		12.433	162641	3.60
2	Diastereomer 1	12.914	1946297	43.08
3	Diastereomer 2	13.376	2346200	51.93
4		13.683	4790	0.11
5		13.867	16592	0.37
6		14.270	41836	0.93

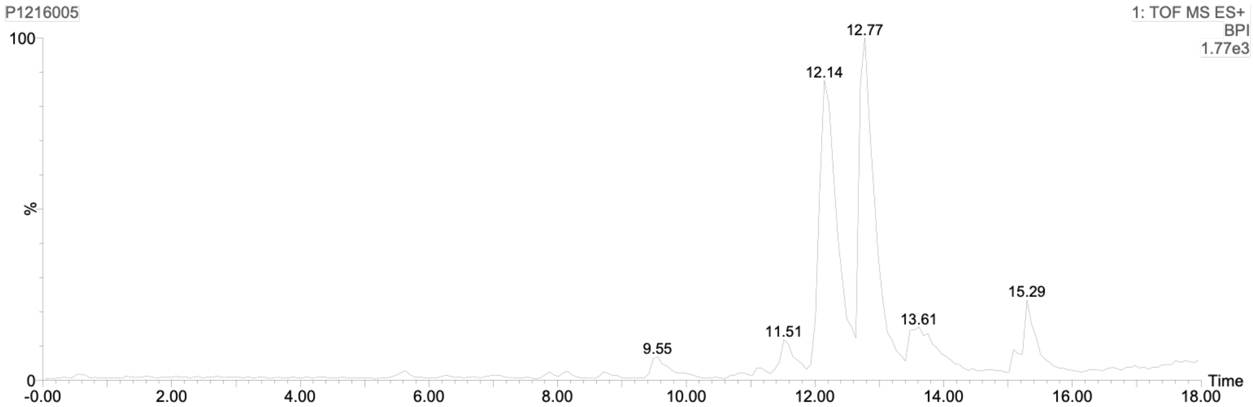

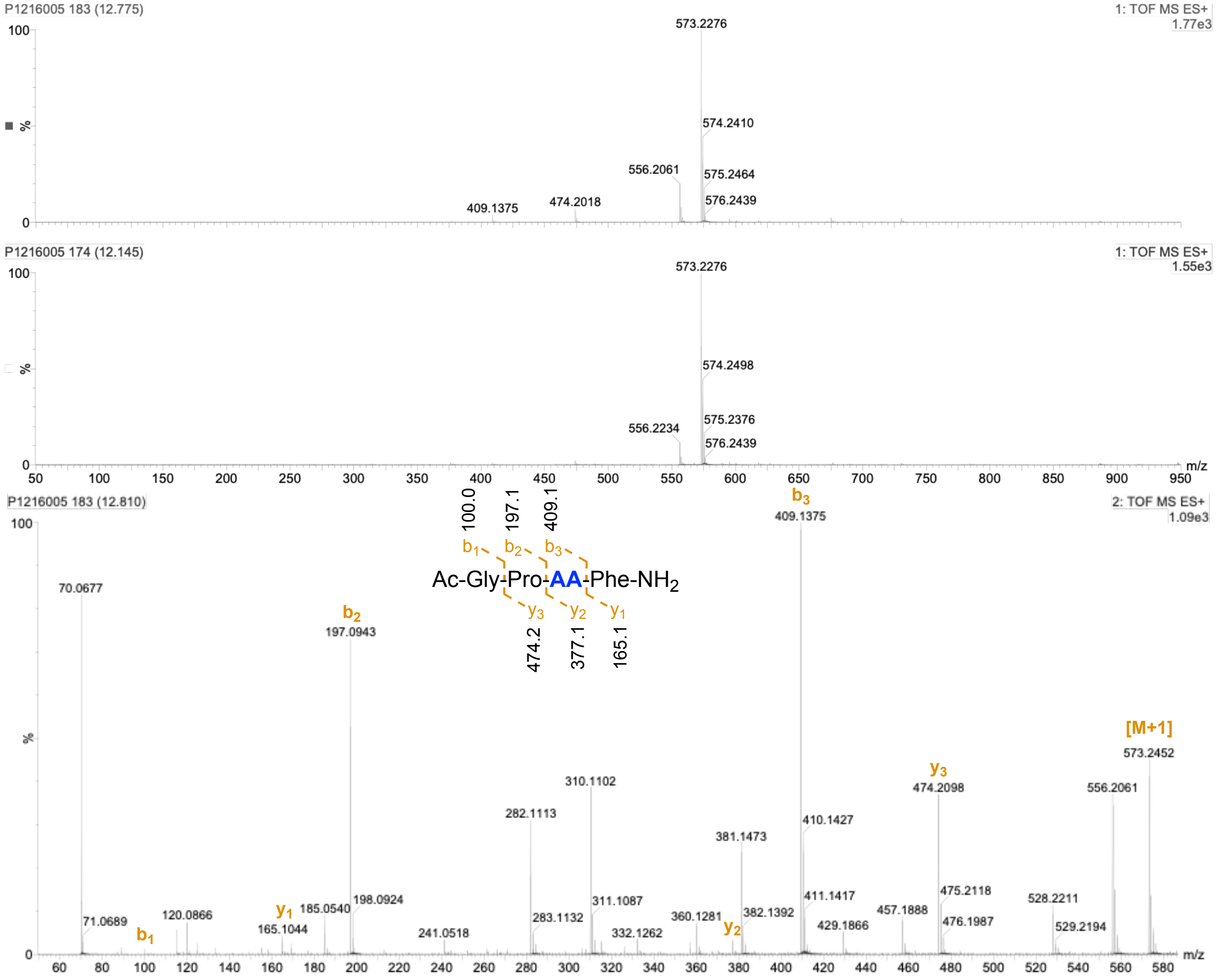
P1216005 183 (12.775)
1: TOF MS ES+
1.77e3
573.2276
574.2410
556.2061
575.2464
576.2439
409.1375
474.2018
P1216005 174 (12.145)
1: TOF MS ES+
1.55e3
573.2276
574.2498
575.2376
556.2234
576.2439
m/z
P1216005 183 (12.810)
2: TOF MS ES+
1.09e3
100.0
197.1
409.1
b1 b2 b3
Ac-Gly-Pro-AA-Phe-NH2
y3 y2 y1
474.2
377.1
165.1
b3
409.1375
70.0677
b2
197.0943
[M+1]
573.2452
y3
474.2098
556.2061
310.1102
282.1113
410.1427
381.1473
%

6A’: MW = 566.7, Purity = 91.7%, Yield = 24.2% [0.32 mg]

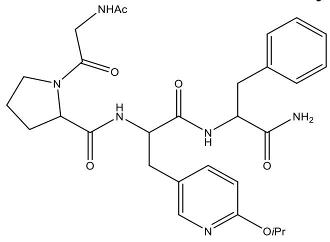

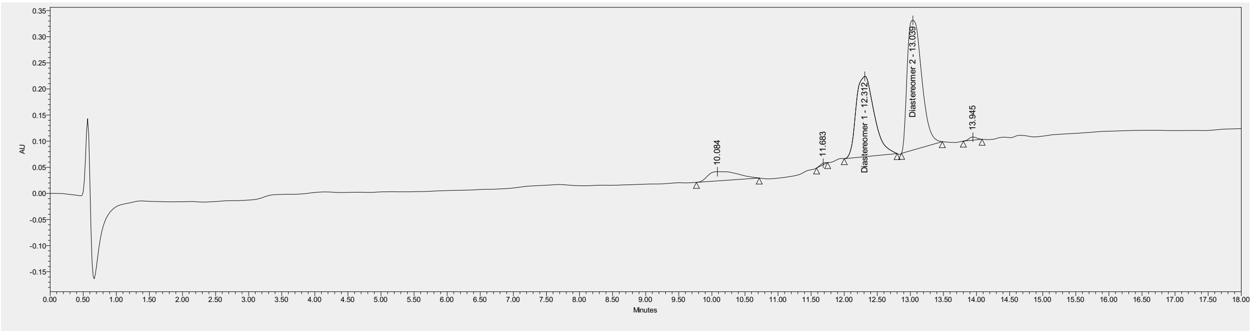

	Name	Retention Time	Area	% Area
1		10.084	547509	7.45
2		11.683	17756	0.24
3	Diastereomer 1	12.312	2935241	39.94
4	Diastereomer 2	13.039	3799736	51.71
5		13.945	48614	0.66

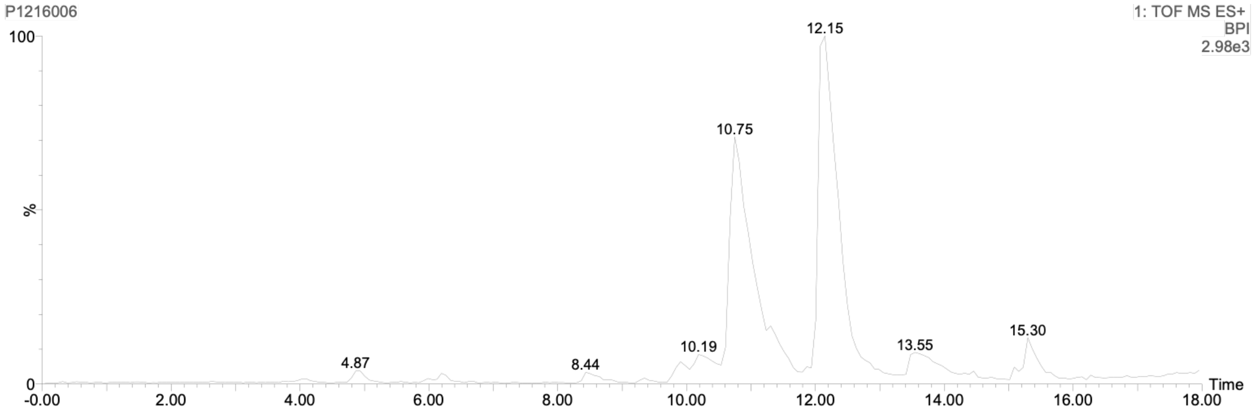

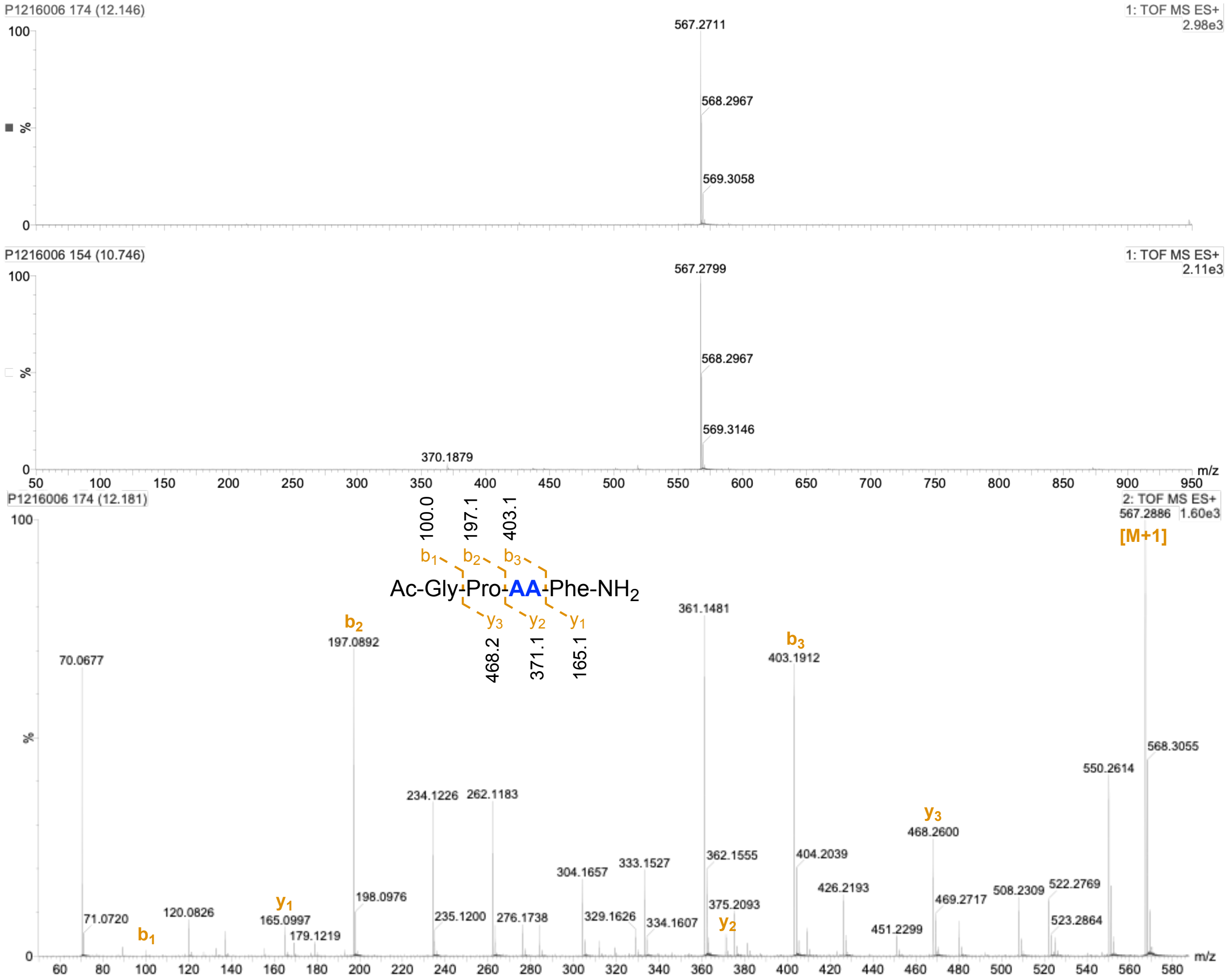
P1216006 174 (12.146)
1: TOF MS ES+
2.98e3
567.2711
568.2967
569.3058
P1216006 154 (10.746)
1: TOF MS ES+
2.11e3
567.2799
568.2967
569.3146
370.1879
m/z
P1216006 174 (12.181)
2: TOF MS ES+
567.2886 1.60e3
[M+1]
Ac-Gly-Pro-AA-Phe-NH2
b1 b2 b3
y3 y2 y1
100.0 197.1 403.1
468.2 371.1 165.1
b2
197.0892
b3
403.1912
y3
468.2600
y1
165.0997
b1
y2
70.0677
71.0720
120.0826
179.1219
198.0976
234.1226
235.1200
262.1183
276.1738
304.1657
329.1626
333.1527
334.1607
361.1481
362.1555
375.2093
404.2039
426.2193
451.2299
469.2717
508.2309
522.2769
523.2864
550.2614
568.3055
%
m/z

7A': MW = 538.6, Purity = 64.8% , Yield = 17.3% [0.22 mg]

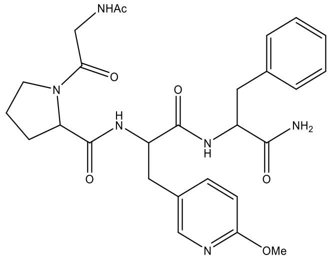

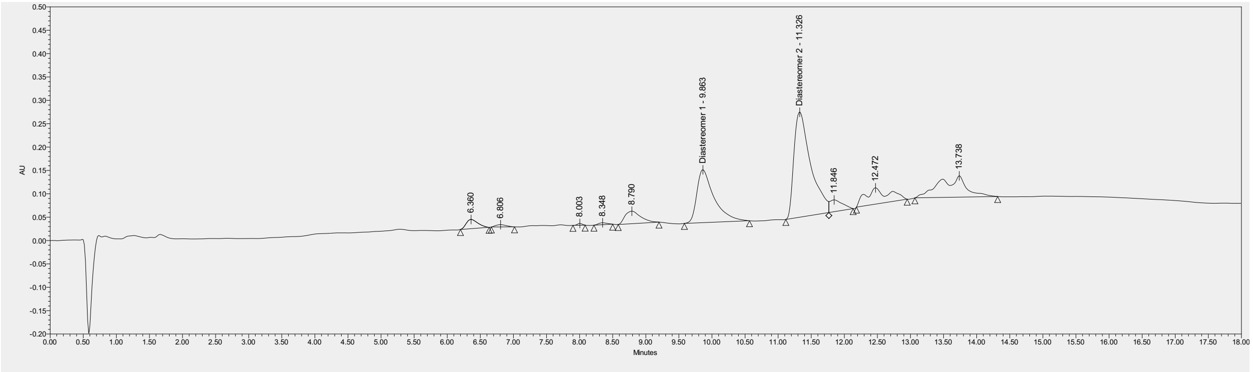

	Name	Retention Time	Area	% Area
1		6.360	243101	2.61
2		6.806	54944	0.59
3		8.003	19553	0.21
4		8.348	37482	0.40
5		8.790	441959	4.75
6	Diastereomer 1	9.863	2051406	22.05
7	Diastereomer 2	11.326	3976868	42.74
8		11.846	335546	3.61
9		12.472	839715	9.02
10		13.738	1304791	14.02

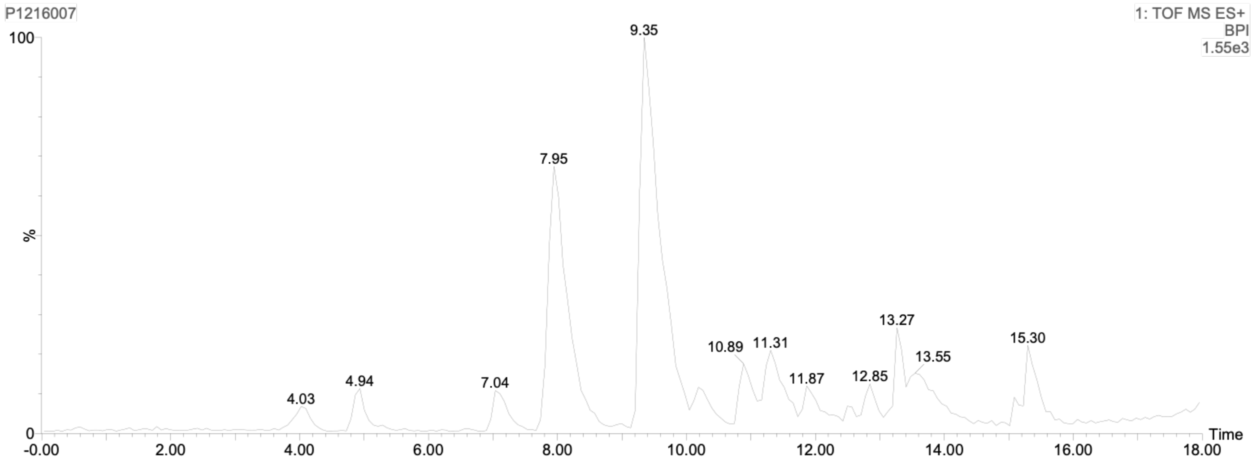

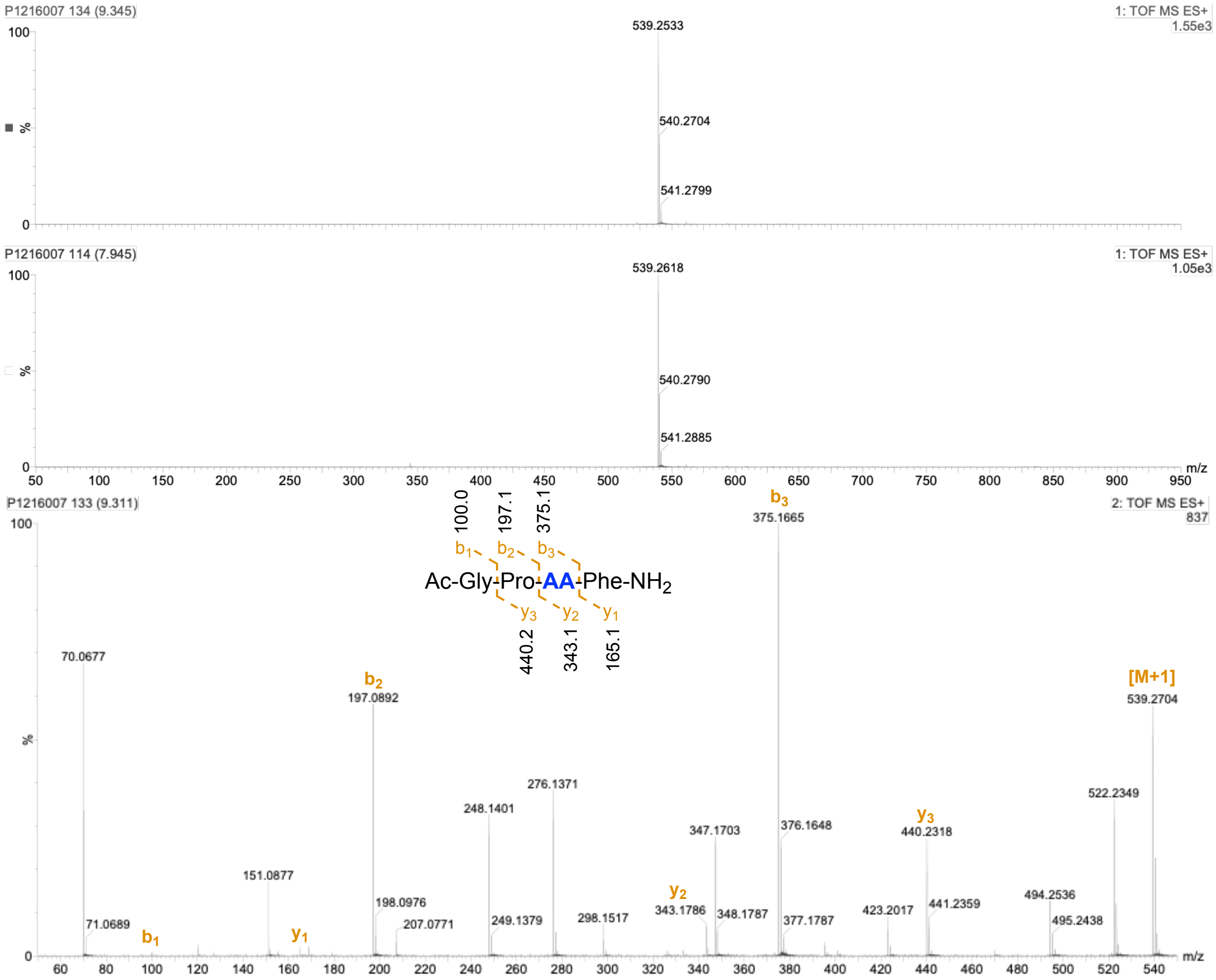
P1216007 134 (9.345)
1: TOF MS ES+
1.55e3
539.2533
540.2704
541.2799
P1216007 114 (7.945)
1: TOF MS ES+
1.05e3
539.2618
540.2790
541.2885
m/z
P1216007 133 (9.311)
2: TOF MS ES+
837
Ac-Gly-Pro-AA-Phe-NH2
b1 b2 b3
100.0 197.1 375.1
y3 y2 y1
440.2 343.1 165.1
b3
375.1665
70.0677
b2
197.0892
[M+1]
539.2704
276.1371
248.1401
522.2349
347.1703
376.1648
y3
440.2318
151.0877
y2
343.1786
198.0976
441.2359
494.2536
71.0689
207.0771
249.1379
298.1517
348.1787
377.1787
423.2017
495.2438
b1
y1
m/z

8A': MW = 568.6, Purity = 81.1%, Yield = 17.9% [0.24 mg]

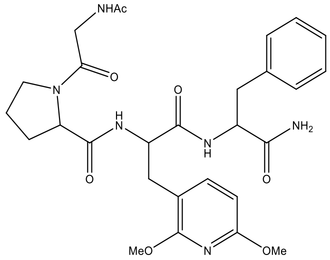

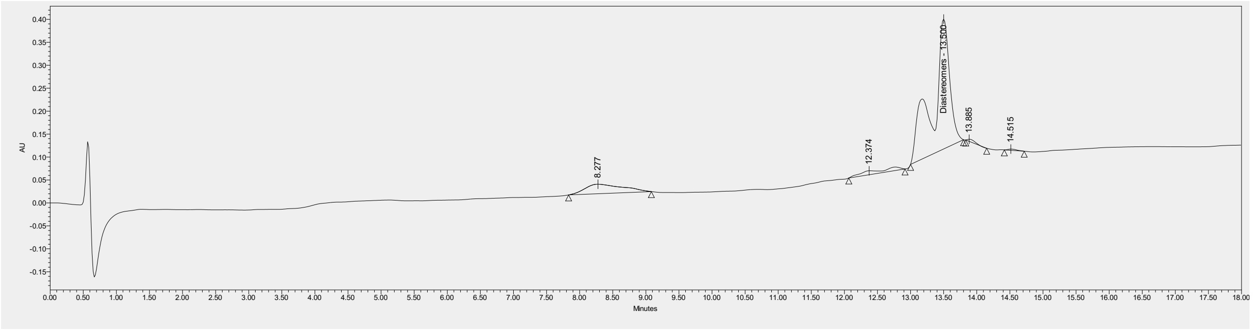

	Name	Retention Time	Area	% Area
1		8.277	818820	13.35
2		12.374	270674	4.41
3	Diastereomers	13.500	4974023	81.10
4		13.885	44795	0.73
5		14.515	24841	0.41

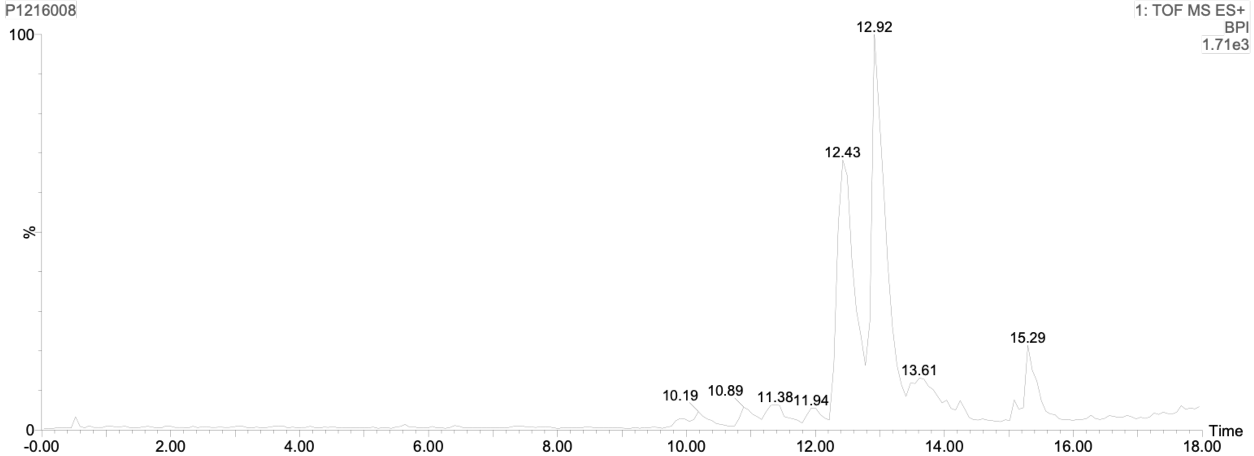

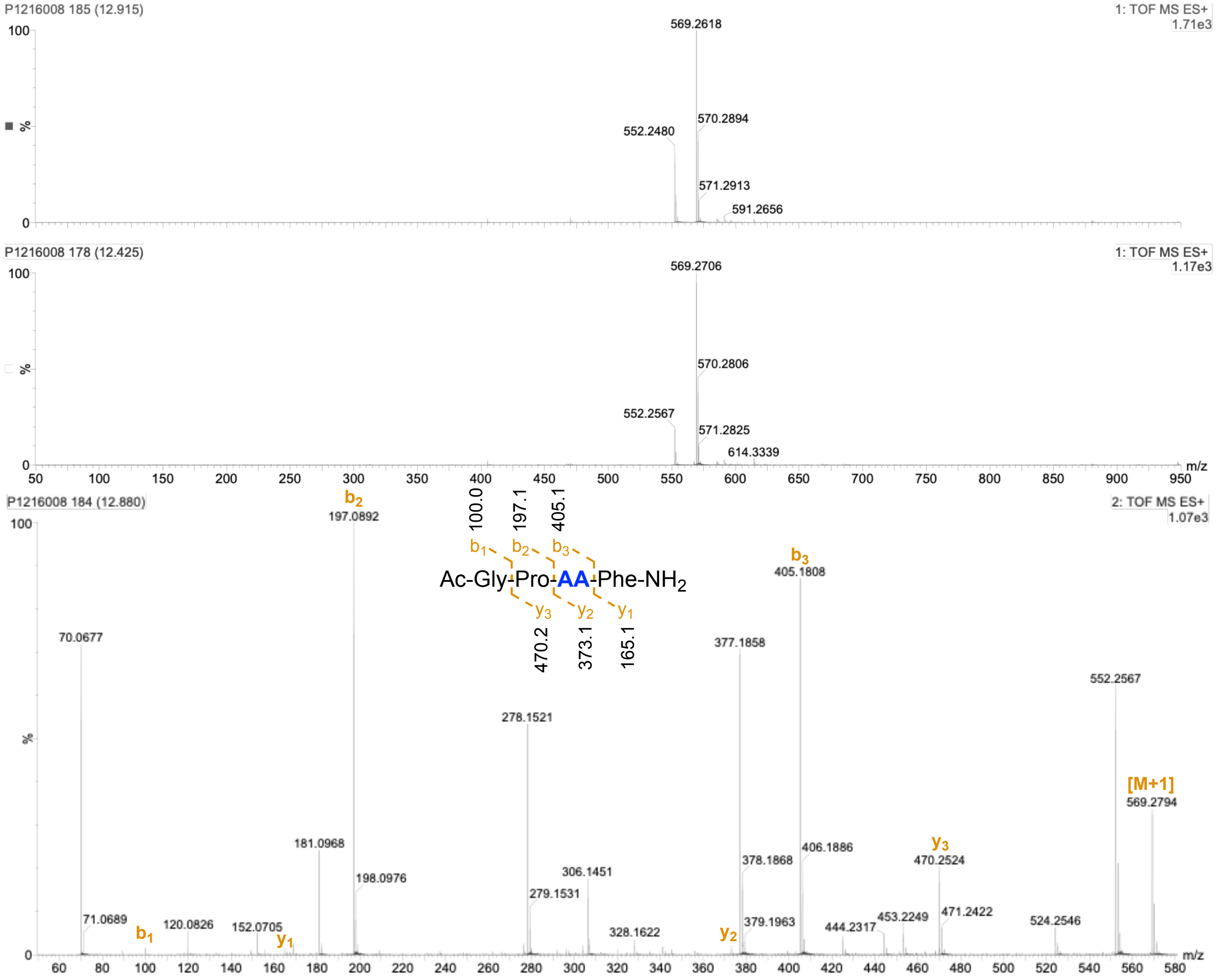

P1216008 185 (12.915)
1: TOF MS ES+
1.71e3
569.2618
570.2894
552.2480
571.2913
591.2656
P1216008 178 (12.425)
1: TOF MS ES+
1.17e3
569.2706
570.2806
552.2567
571.2825
614.3339
m/z
P1216008 184 (12.880)
2: TOF MS ES+
1.07e3
Ac-Gly-Pro-AA-Phe-NH2
b1 b2 b3
100.0 197.1 405.1
y3 y2 y1
470.2 373.1 165.1
b2
197.0892
70.0677
b3
405.1808
377.1858
552.2567
278.1521
[M+1]
569.2794
181.0968
406.1886
y3
470.2524
198.0976
378.1868
306.1451
279.1531
71.0689
b1
120.0826
152.0705
y1
328.1622
y2
379.1963
444.2317
453.2249
471.2422
524.2546
m/z

9A’: MW = 538.6, Purity = 17.4%, Yield = 2.8% [0.035 mg]

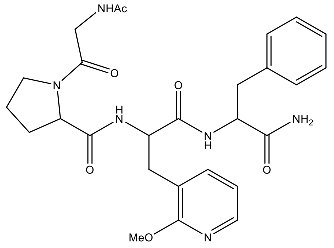

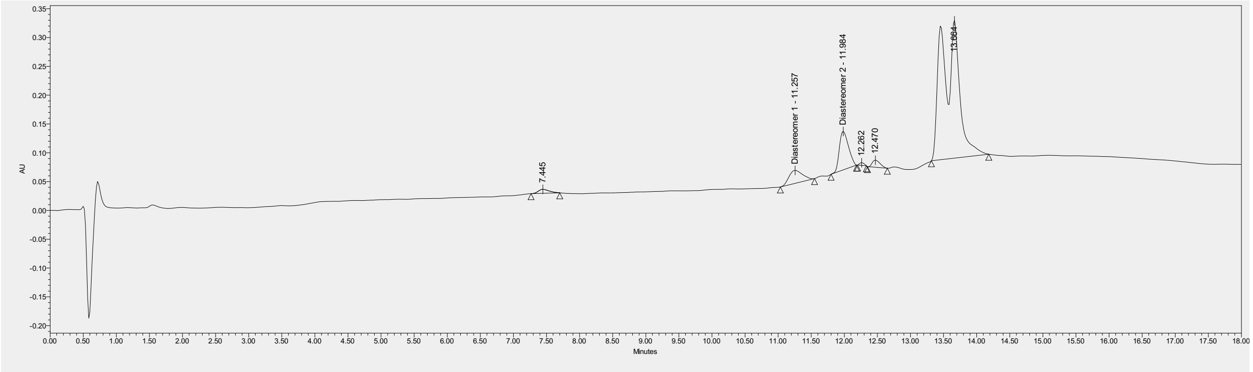

	Name	Retention Time	Area	% Area
1		7.445	79019	1.43
2	Diastereomer 1	11.257	320073	5.79
3	Diastereomer 2	11.984	639923	11.57
4		12.262	22771	0.41
5		12.470	100891	1.82
6		13.664	4367606	78.98

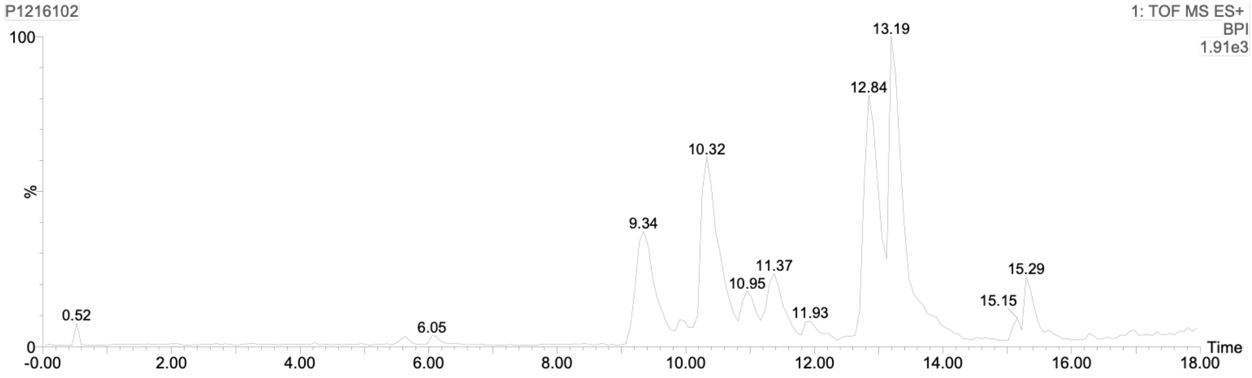

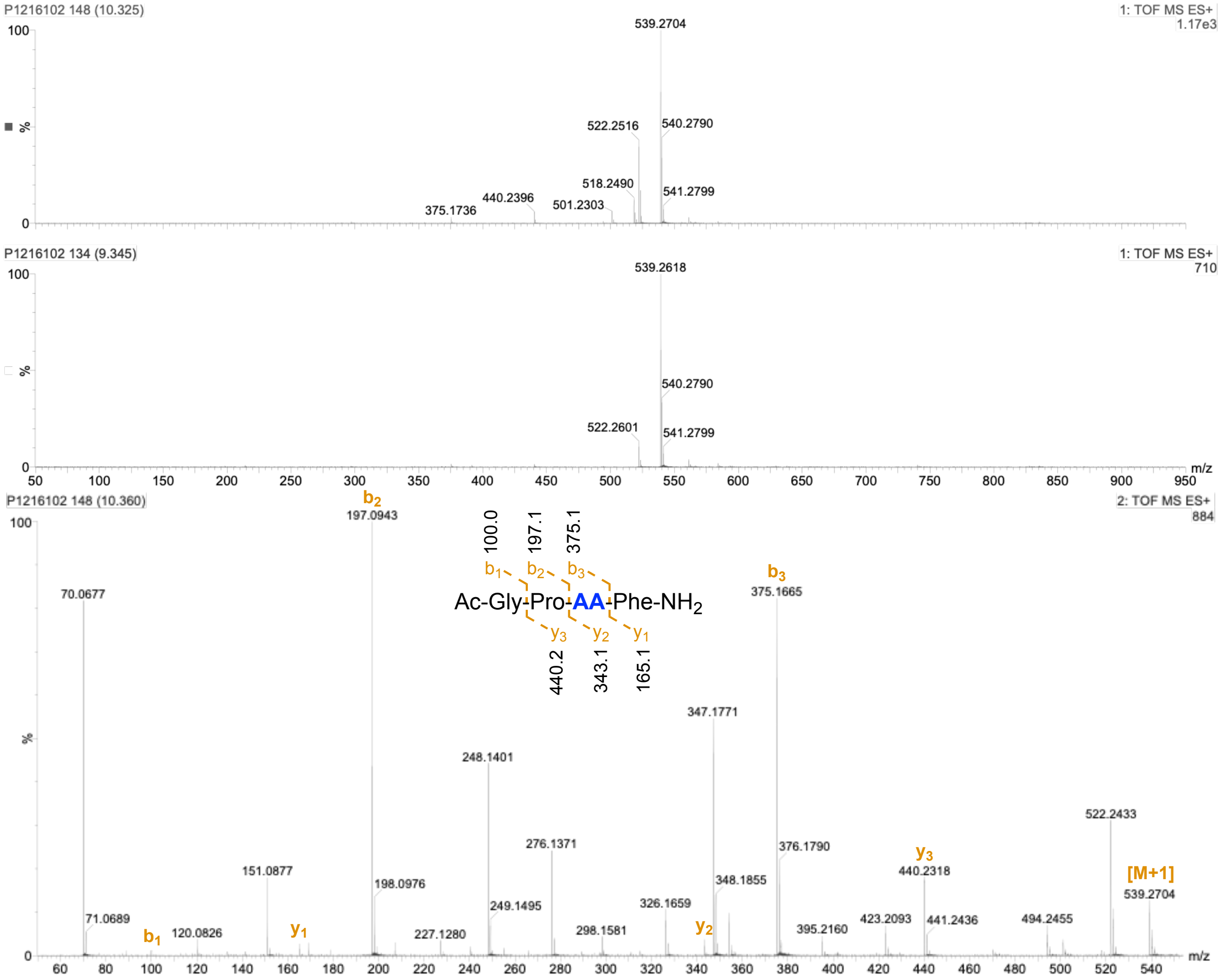

P1216102 148 (10.325)
1: TOF MS ES+
1.17e3
539.2704
522.2516
540.2790
518.2490
541.2799
375.1736
440.2396
501.2303
P1216102 134 (9.345)
1: TOF MS ES+
710
539.2618
540.2790
522.2601
541.2799
m/z
P1216102 148 (10.360)
2: TOF MS ES+
884
b2
197.0943
Ac-Gly-Pro-AA-Phe-NH2
100.0
197.1
375.1
440.2
343.1
165.1
70.0677
b3
375.1665
347.1771
248.1401
522.2433
276.1371
376.1790
y3
440.2318
[M+1]
539.2704
151.0877
198.0976
348.1855
326.1659
249.1495
71.0689
b1
120.0826
y1
227.1280
298.1581
y2
395.2160
423.2093
441.2436
494.2455
m/z

10A': MW = 556.6, Purity = 61.9%, Yield = 15.2% [0.20 mg]

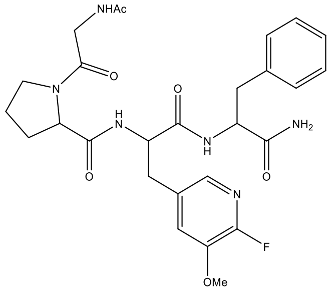

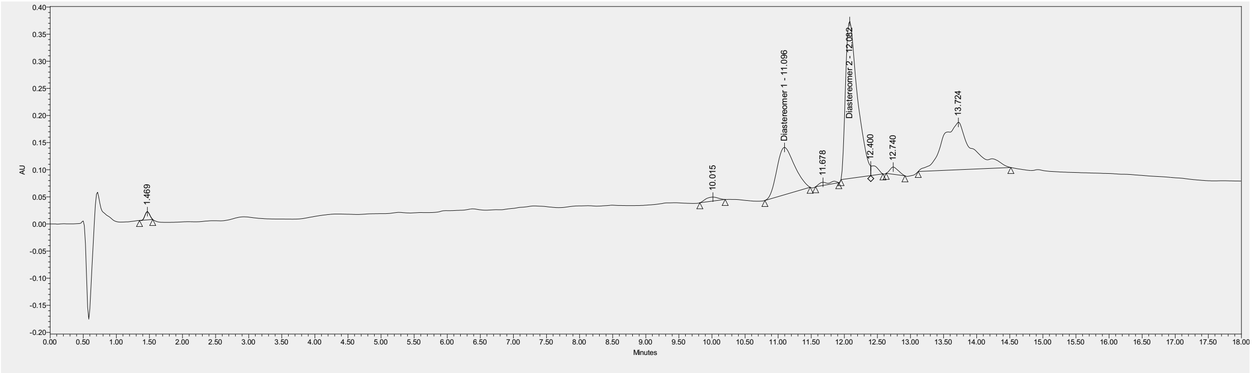

	Name	Retention Time	Area	% Area
1		1.469	75484	0.88
2		10.015	99685	1.17
3	Diastereomer 1	11.096	1629105	19.10
4		11.678	72677	0.85
5	Diastereomer 2	12.082	3650747	42.80
6		12.400	124123	1.46
7		12.740	116955	1.37
8		13.724	2761940	32.38

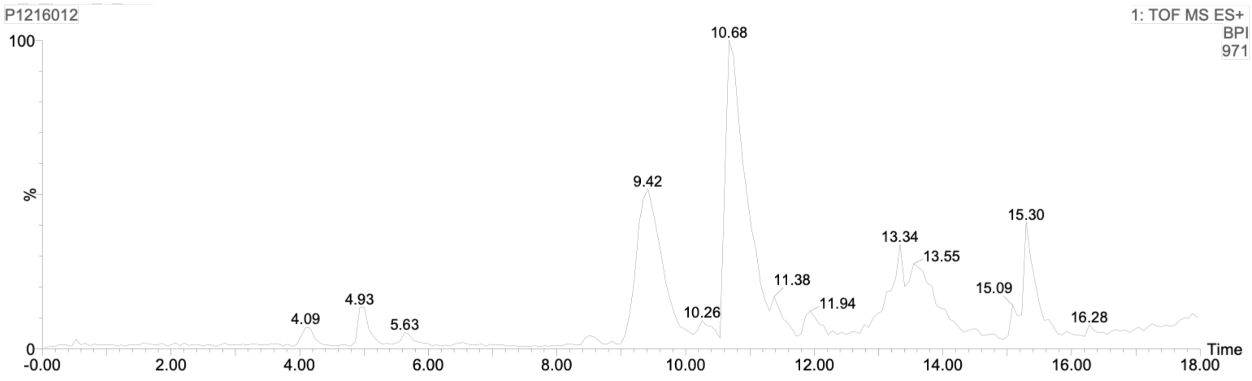

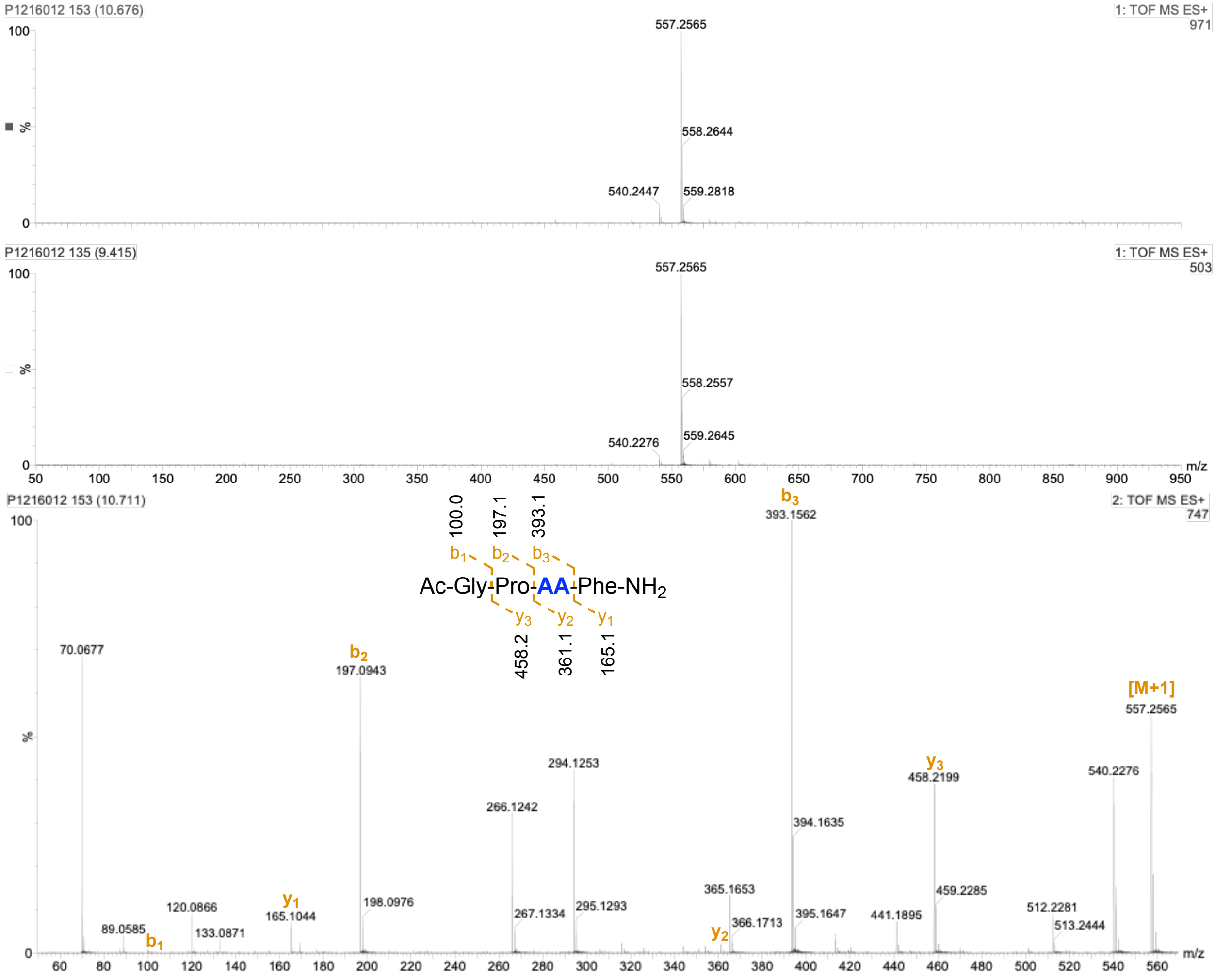
P1216012 153 (10.676)
1: TOF MS ES+
971
557.2565
558.2644
540.2447
559.2818
P1216012 135 (9.415)
1: TOF MS ES+
503
557.2565
558.2557
540.2276
559.2645
m/z
P1216012 153 (10.711)
2: TOF MS ES+
747
Ac-Gly-Pro-AA-Phe-NH2
100.0
197.1
393.1
458.2
361.1
165.1
b3
393.1562
70.0677
b2
197.0943
[M+1]
557.2565
294.1253
y3
458.2199
540.2276
266.1242
394.1635
365.1653
459.2285
120.0866
y1
165.1044
198.0976
267.1334
295.1293
395.1647
441.1895
512.2281
89.0585
b1
133.0871
y2
366.1713
513.2444
m/z

11A': MW = 623.7, Purity = 96.2%, Yield = 11.2% [0.16 mg]

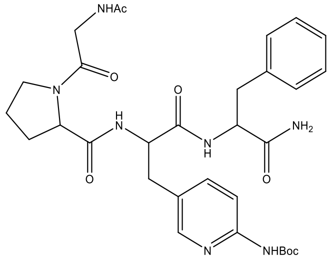

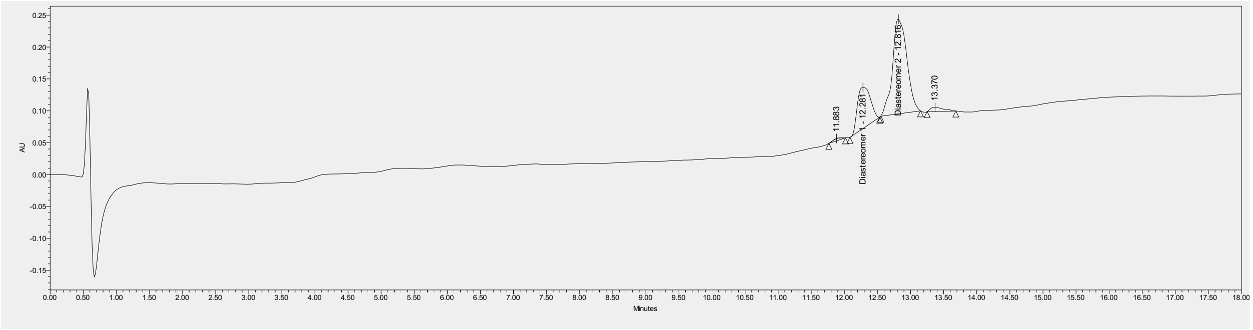

	Name	Retention Time	Area	% Area
1		11.883	32108	0.99
2	Diastereomer 1	12.281	882241	27.10
3	Diastereomer 2	12.816	2249611	69.10
4		13.370	91744	2.82

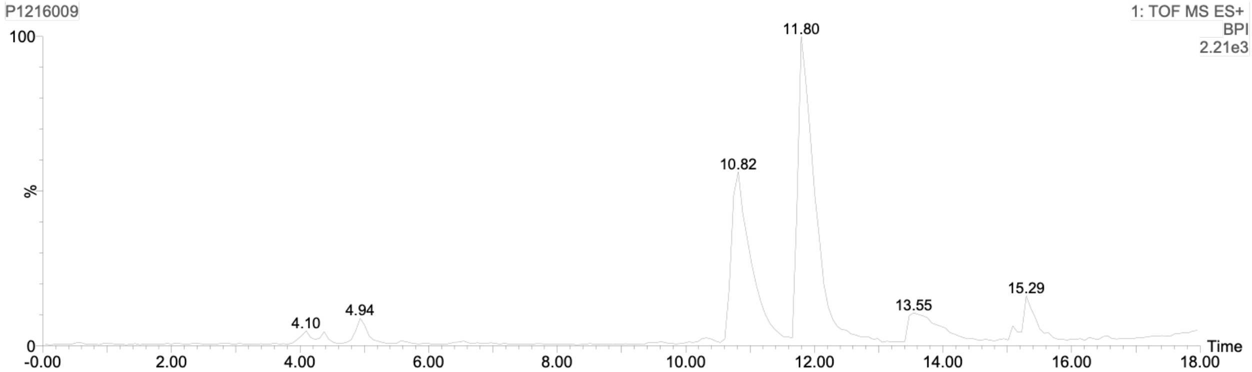

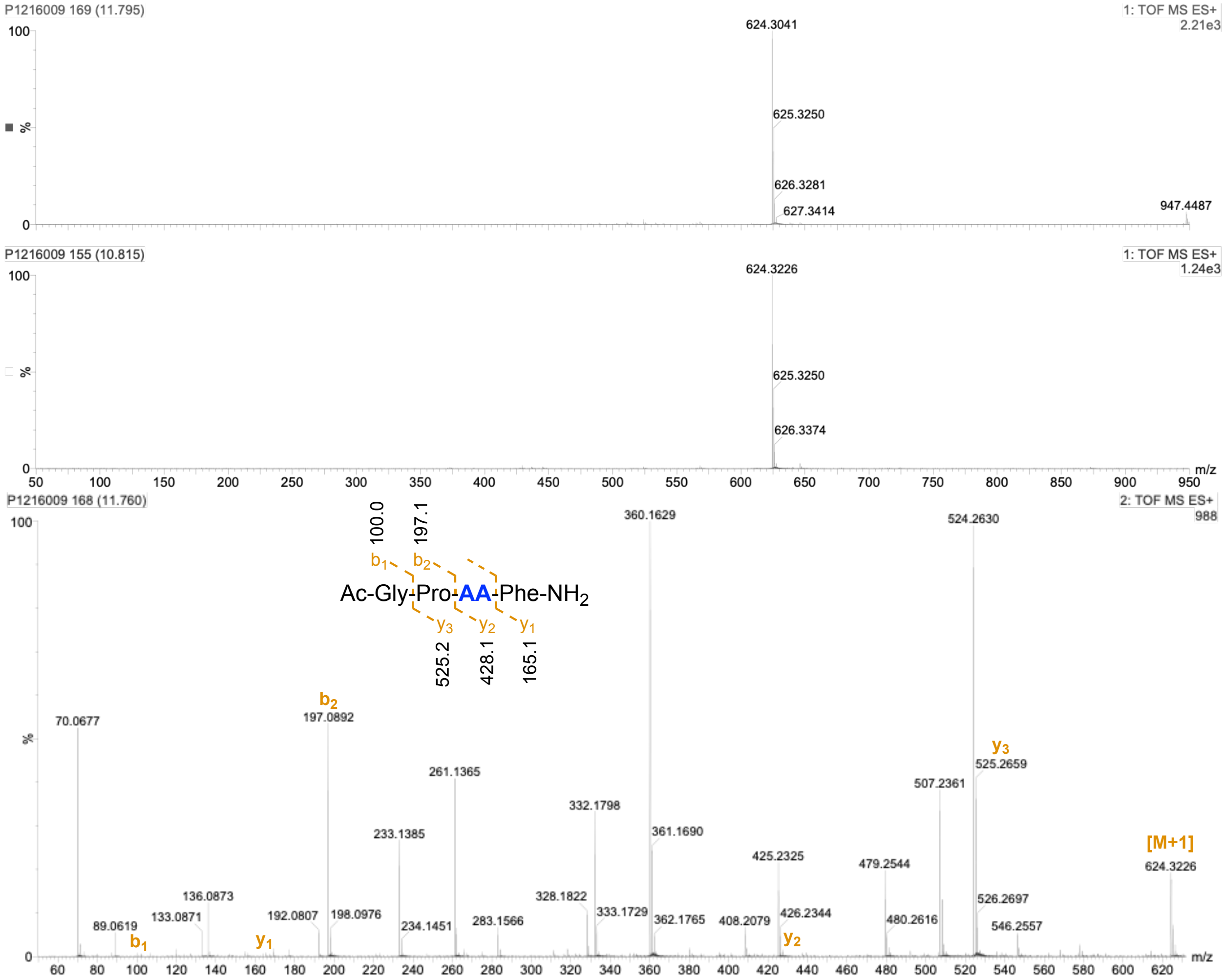
P1216009 169 (11.795)
1: TOF MS ES+
2.21e3
624.3041
625.3250
626.3281
627.3414
947.4487
P1216009 155 (10.815)
1: TOF MS ES+
1.24e3
624.3226
625.3250
626.3374
m/z
P1216009 168 (11.760)
2: TOF MS ES+
988
Ac-Gly-Pro-AA-Phe-NH2
b1 b2 y3 y2 y1
100.0
197.1
525.2
428.1
165.1
360.1629
524.2630
70.0677
197.0892
261.1365
332.1798
233.1385
361.1690
425.2325
479.2544
507.2361
525.2659
[M+1]
624.3226
136.0873
133.0871
89.0619
192.0807
198.0976
234.1451
283.1566
328.1822
333.1729
362.1765
408.2079
426.2344
480.2616
526.2697
546.2557
m/z

12A': MW = 554.7, Purity = 85.5% , Yield = 21.6% [0.28 mg]

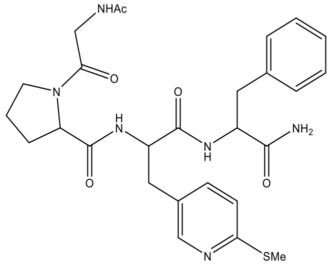

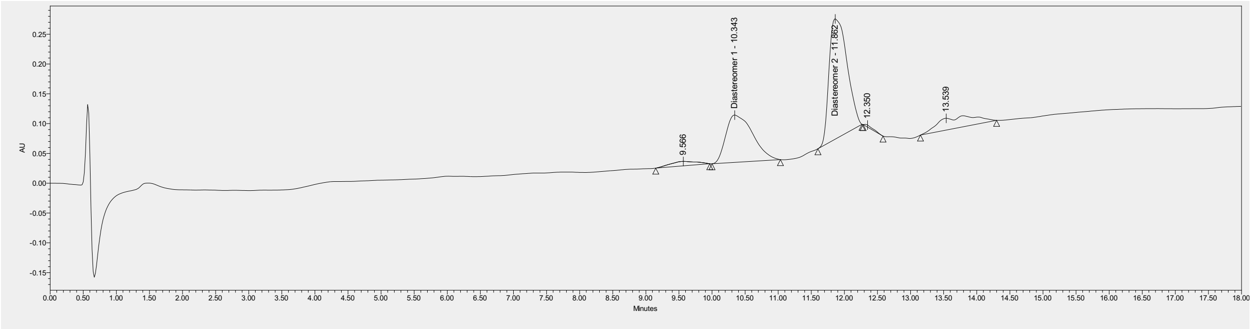

	Name	Retention Time	Area	% Area
1		9.566	210062	2.98
2	Diastereomer 1	10.343	2232302	31.70
3	Diastereomer 2	11.862	3789879	53.81
4		12.350	32135	0.46
5		13.539	778419	11.05

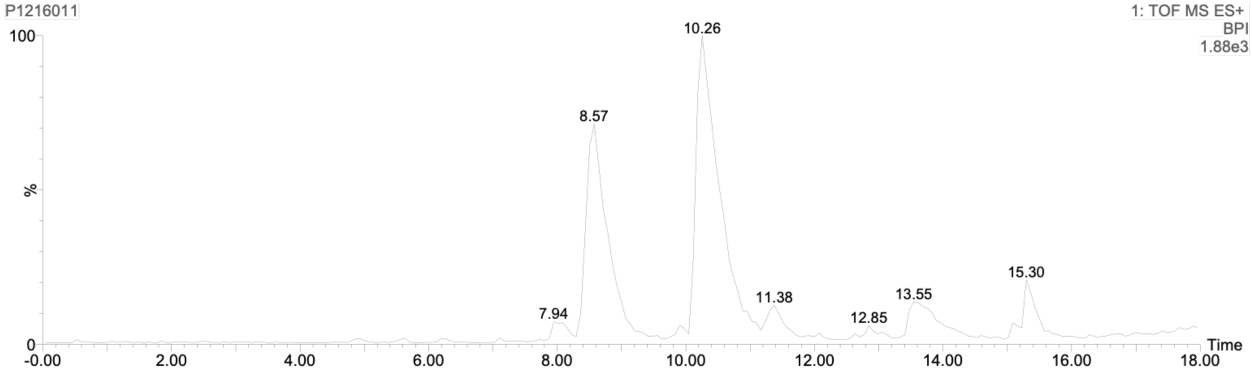

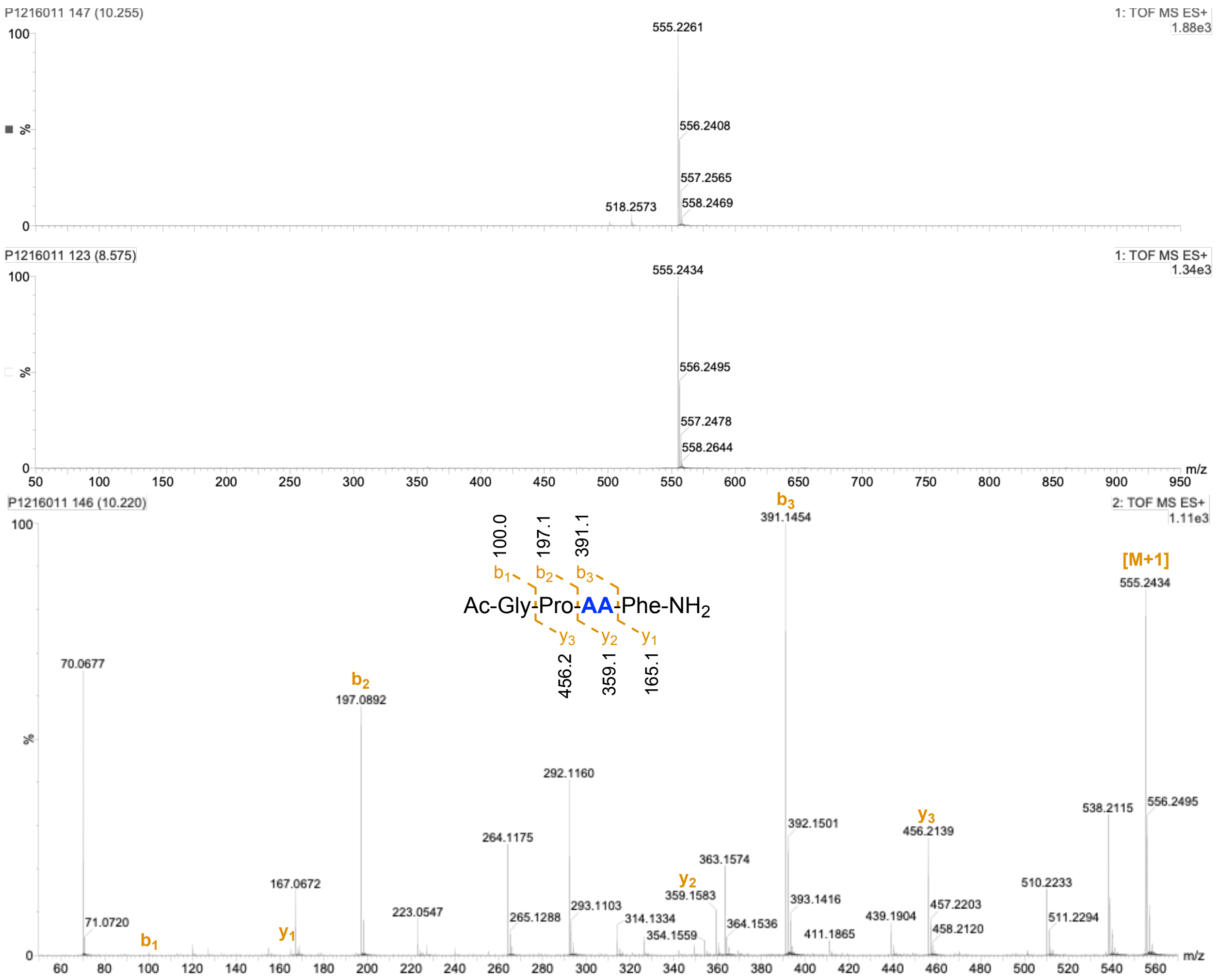
P1216011 147 (10.255)
1: TOF MS ES+
1.88e3
555.2261
556.2408
557.2565
558.2469
518.2573
P1216011 123 (8.575)
1: TOF MS ES+
1.34e3
555.2434
556.2495
557.2478
558.2644
m/z
P1216011 146 (10.220)
2: TOF MS ES+
1.11e3
Ac-Gly-Pro-AA-Phe-NH2
b1 b2 b3
100.0 197.1 391.1
y3 y2 y1
456.2 359.1 165.1
b3
391.1454
[M+1]
555.2434
70.0677
b2
197.0892
292.1160
264.1175
392.1501
y3
456.2139
538.2115
556.2495
363.1574
y2
359.1583
167.0672
510.2233
223.0547
265.1288
293.1103
314.1334
354.1559
364.1536
393.1416
411.1865
439.1904
457.2203
458.2120
511.2294
71.0720
b1
y1
m/z

1B’: MW = 552.6, Purity = 68.8%, Yield = 17.5% [0.23 mg]

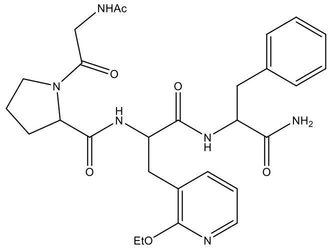

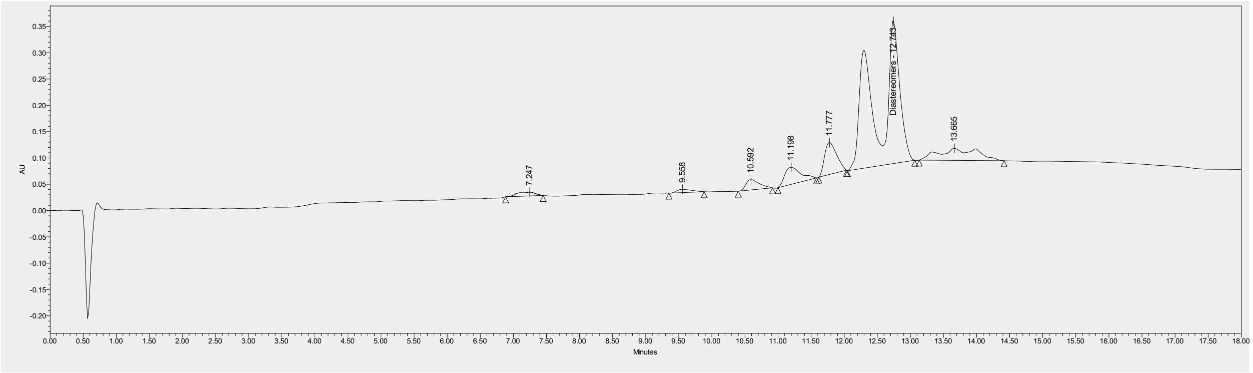

	Name	Retention Time	Area	% Area
1		7.247	139436	1.57
2		9.558	96400	1.09
3		10.592	277723	3.13
4		11.198	547354	6.18
5		11.777	765829	8.64
6	Diastereomers	12.743	6100197	68.83
7		13.665	935889	10.56

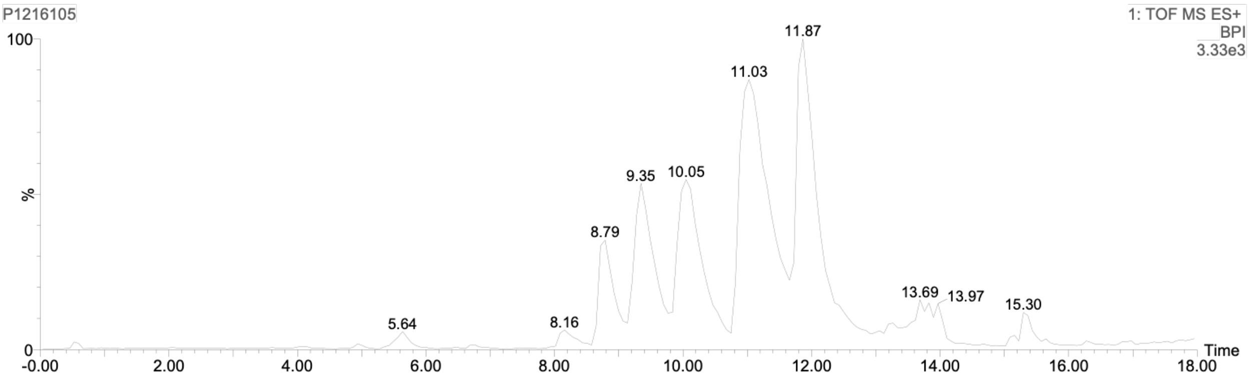

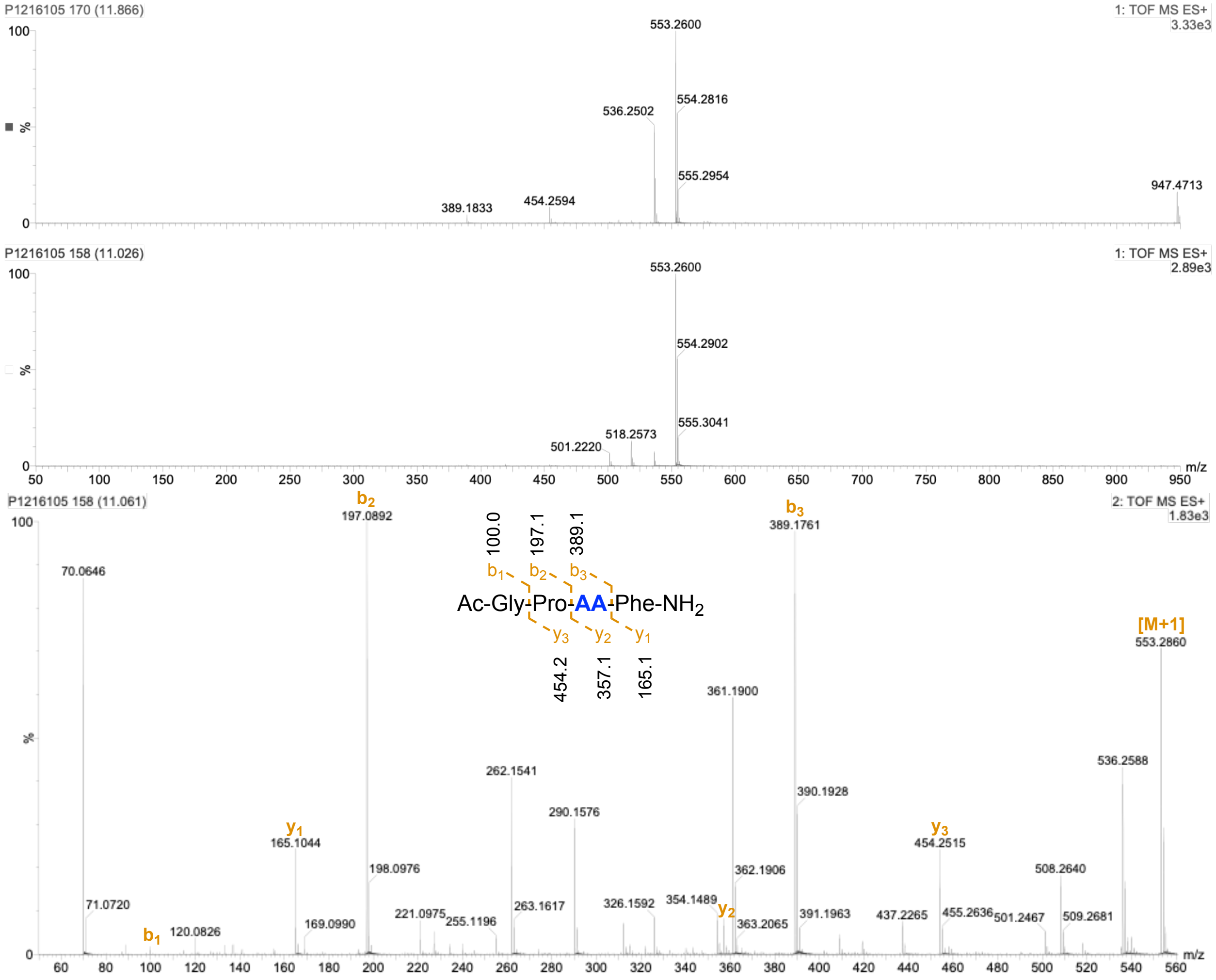
P1216105 170 (11.866)
1: TOF MS ES+
3.33e3
553.2600
554.2816
536.2502
555.2954
947.4713
389.1833
454.2594
P1216105 158 (11.026)
1: TOF MS ES+
2.89e3
553.2600
554.2902
555.3041
518.2573
501.2220
m/z
P1216105 158 (11.061)
2: TOF MS ES+
1.83e3
b2
197.0892
b3
389.1761
70.0646
Ac-Gly-Pro-AA-Phe-NH2
100.0
197.1
389.1
454.2
357.1
165.1
[M+1]
553.2860
361.1900
536.2588
262.1541
390.1928
290.1576
y1
165.1044
y3
454.2515
198.0976
362.1906
508.2640
71.0720
b1
120.0826
169.0990
221.0975
255.1196
263.1617
326.1592
354.1489
y2
363.2065
391.1963
437.2265
455.2636
501.2467
509.2681

2B’: MW = 552.6, Purity = 46.1%, Yield = 14.3% [0.18 mg]

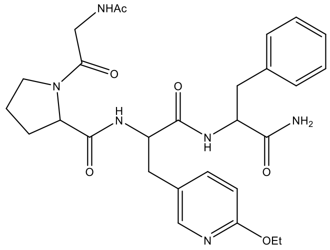

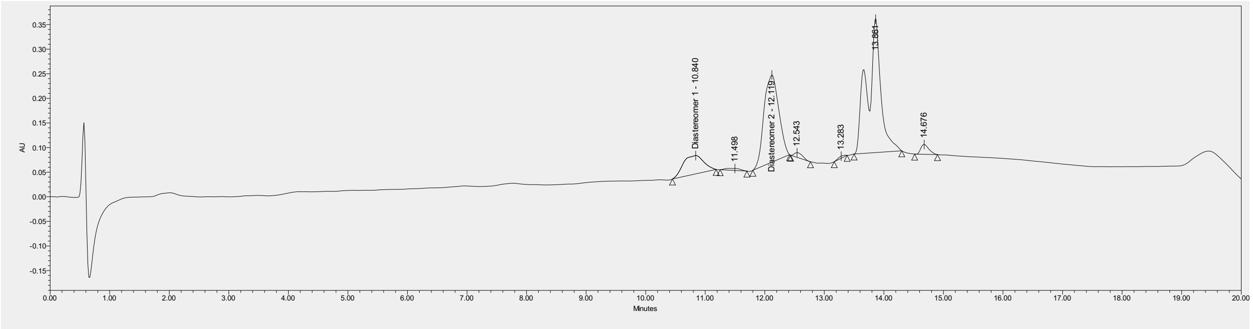

	Name	Retention Time	Area	% Area
1	Diastereomer 1	10.840	915908	10.63
2		11.498	69924	0.81
3	Diastereomer 2	12.119	3055292	35.45
4		12.543	95775	1.11
5		13.283	34527	0.40
6		13.861	4257742	49.40
7		14.676	190406	2.21

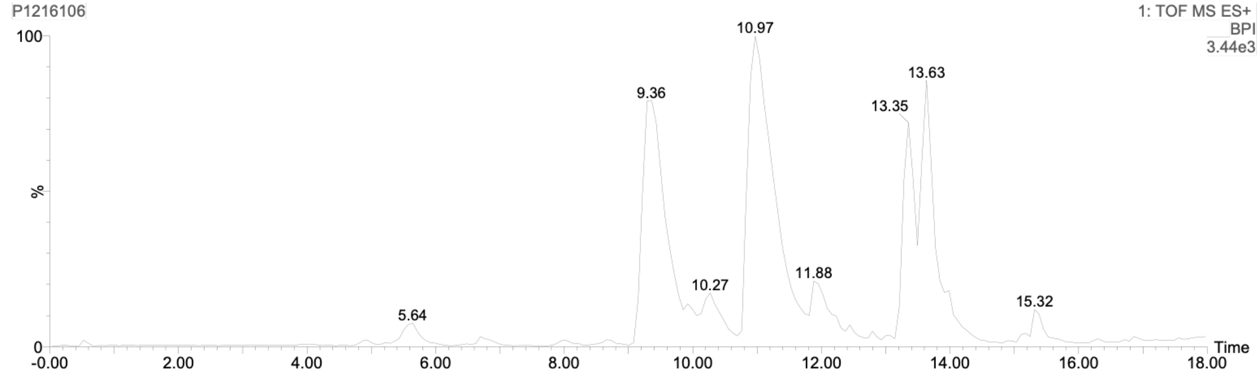

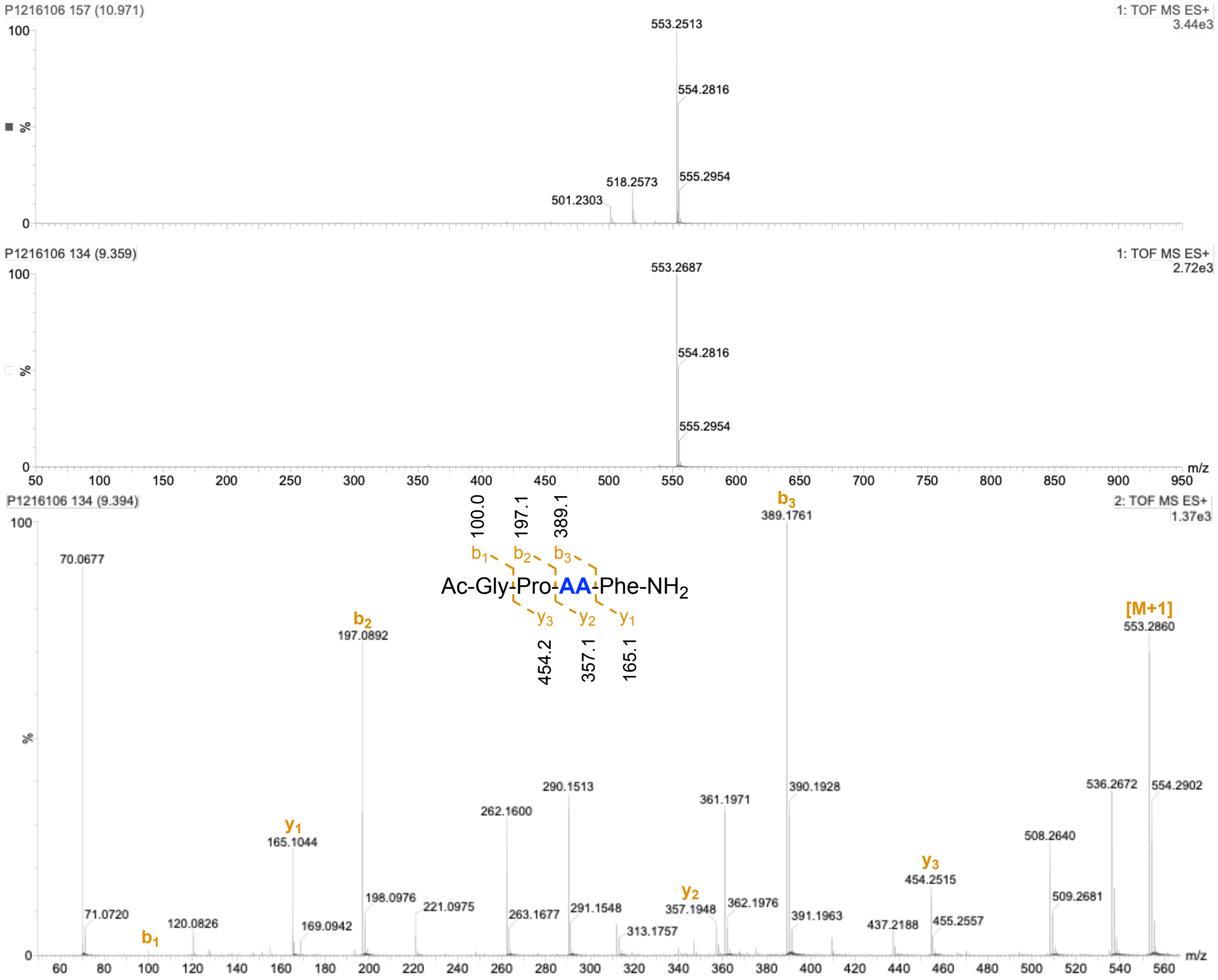
P1216106 157 (10.971)
1: TOF MS ES+
3.44e3
553.2513
554.2816
518.2573
555.2954
501.2303
P1216106 134 (9.359)
1: TOF MS ES+
2.72e3
553.2687
554.2816
555.2954
m/z
P1216106 134 (9.394)
2: TOF MS ES+
1.37e3
Ac-Gly-Pro-AA-Phe-NH2
b1 b2 b3
100.0 197.1 389.1
y3 y2 y1
454.2 357.1 165.1
b3
389.1761
70.0677
b2
197.0892
[M+1]
553.2860
290.1513
262.1600
361.1971
390.1928
536.2672
554.2902
y1
165.1044
508.2640
y3
454.2515
y2
357.1948
198.0976
221.0975
263.1677
291.1548
362.1976
391.1963
437.2188
455.2557
509.2681
71.0720
120.0826
169.0942
313.1757
b1
%

3B': MW = 558.6, Purity = 56.9%, Yield = 3.3% [0.043 mg]

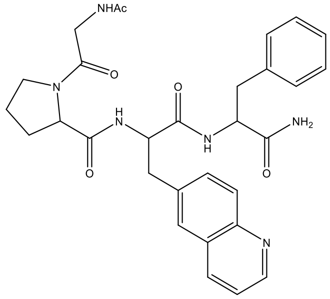

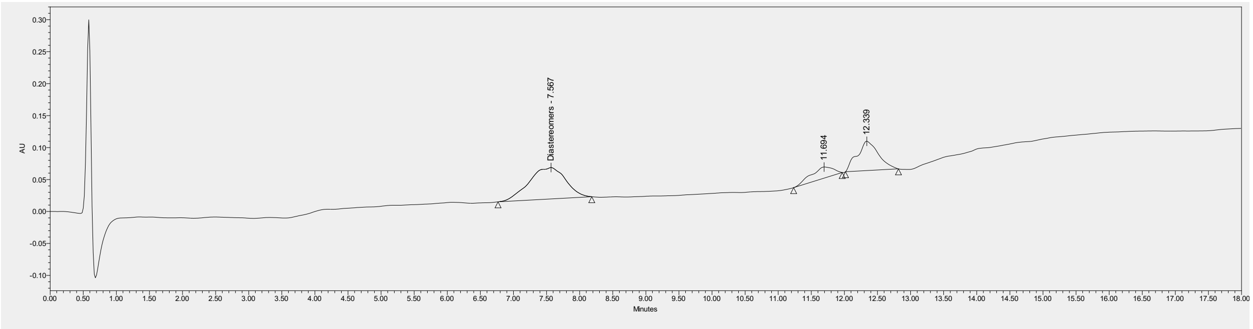

	Name	Retention Time	Area	% Area
1	Diastereomers	7.567	1905181	56.87
2		11.694	417853	12.47
3		12.339	1026994	30.66

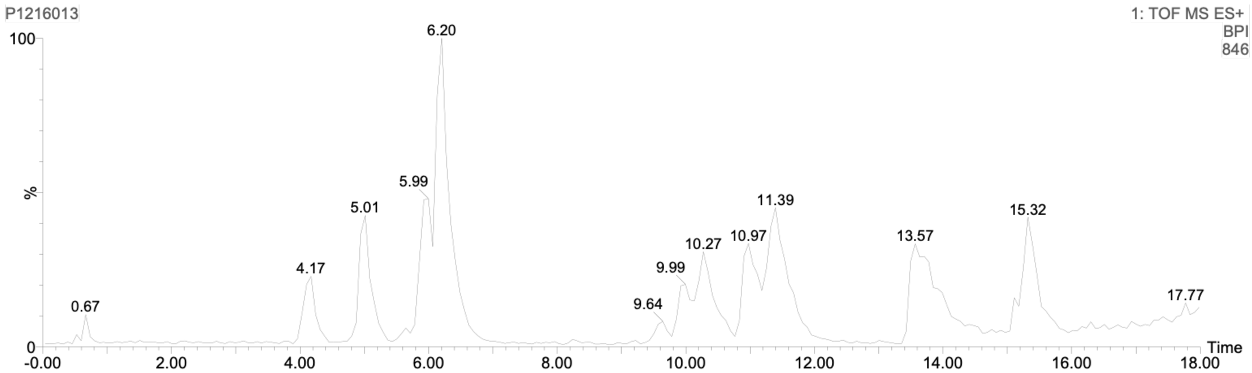

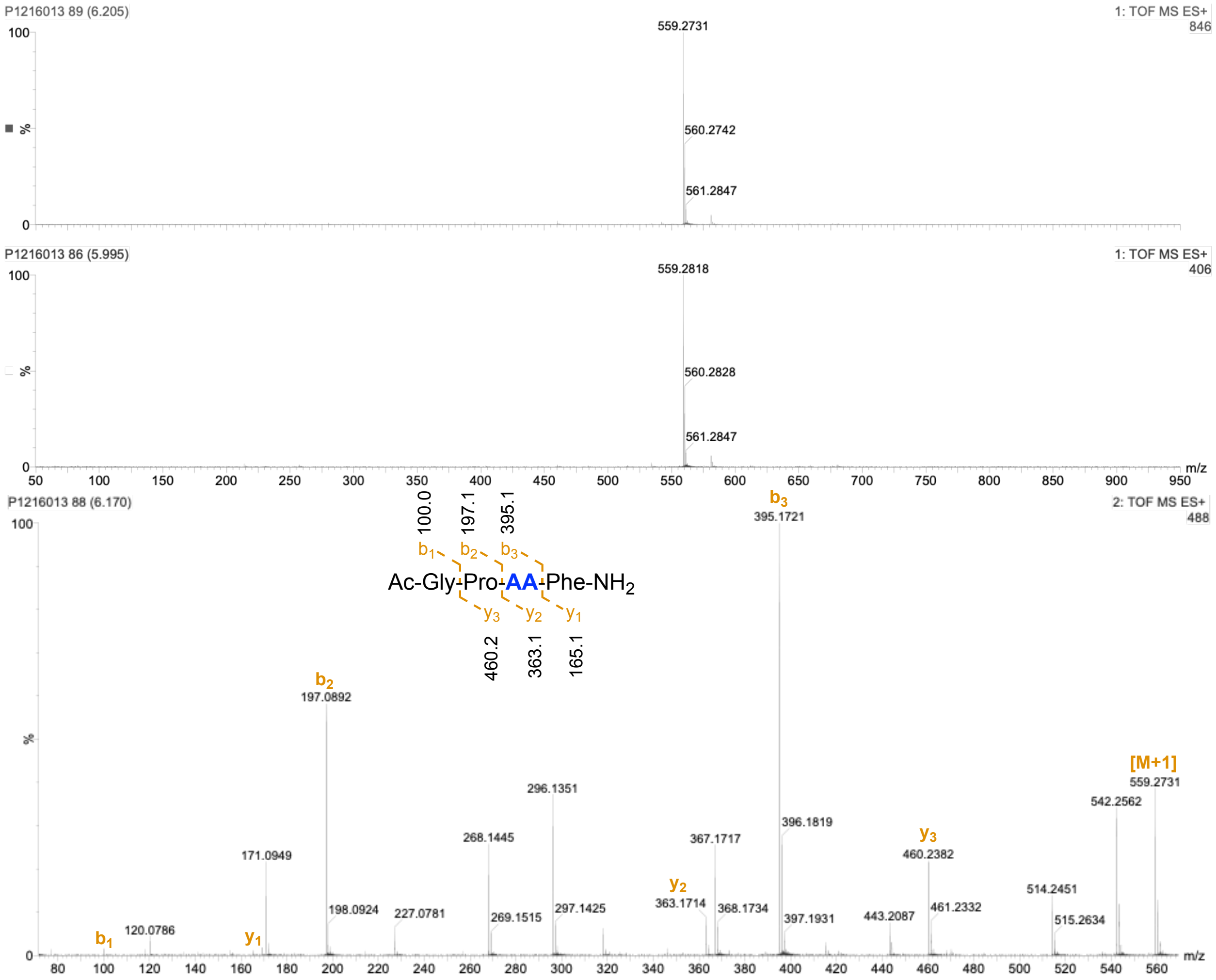
P1216013 89 (6.205)
1: TOF MS ES+
846
559.2731
560.2742
561.2847
P1216013 86 (5.995)
1: TOF MS ES+
406
559.2818
560.2828
561.2847
m/z
P1216013 88 (6.170)
2: TOF MS ES+
488
Ac-Gly-Pro-AA-Phe-NH2
b1 b2 b3
100.0 197.1 395.1
y3 y2 y1
460.2 363.1 165.1
b3
395.1721
b2
197.0892
[M+1]
559.2731
542.2562
296.1351
396.1819
y3
460.2382
171.0949
268.1445
367.1717
y2
363.1714
514.2451
198.0924
227.0781
269.1515
297.1425
368.1734
397.1931
443.2087
461.2332
515.2634
b1
120.0786
y1
m/z

4B’: MW = 558.6, Purity = 91.1%, Yield = 9.7% [0.13 mg]

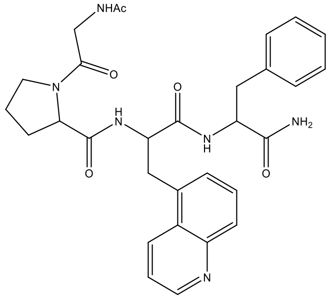

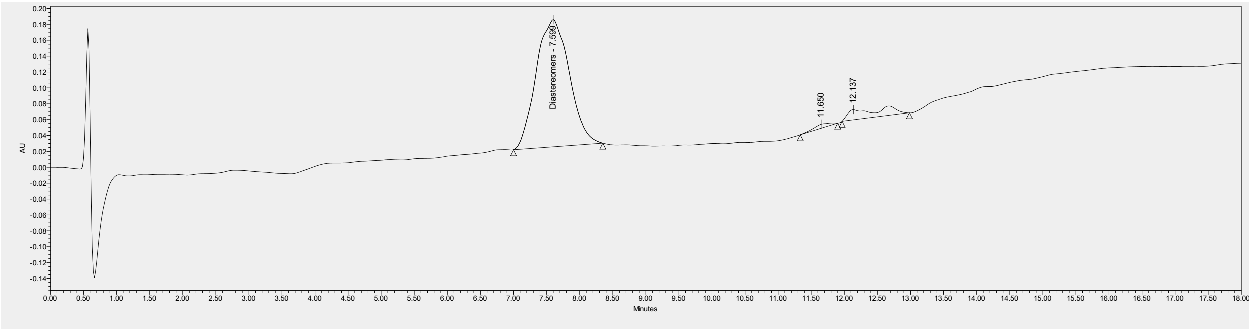

	Name	Retention Time	Area	% Area
1	Diastereomers	7.599	5585025	91.09
2		11.650	86251	1.41
3		12.137	460252	7.51

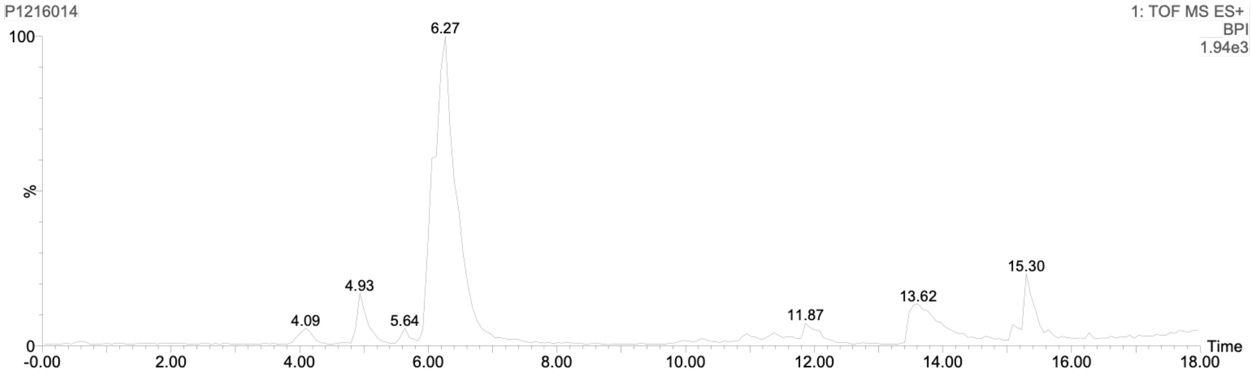

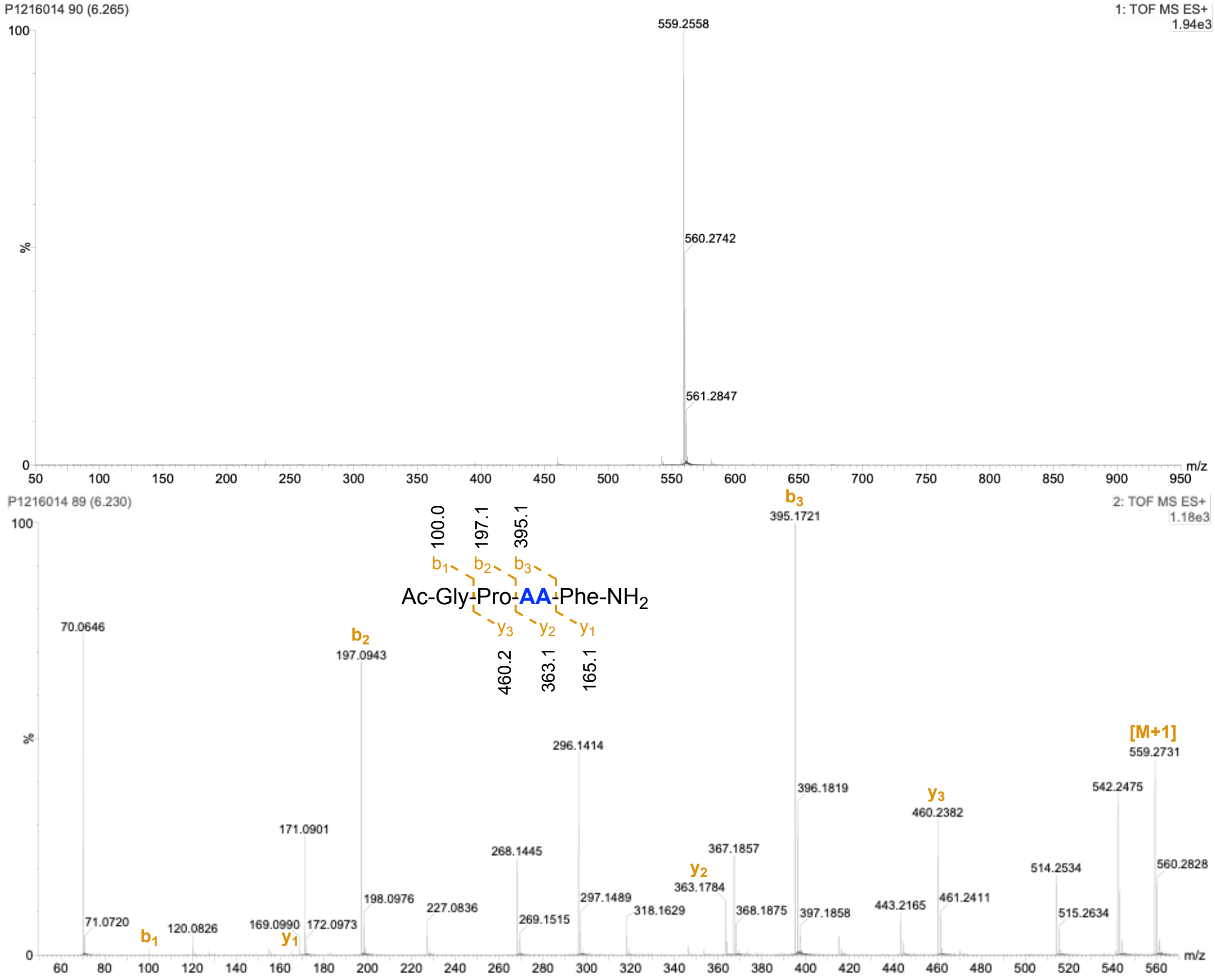
P1216014 90 (6.265)
1: TOF MS ES+
1.94e3
559.2558
560.2742
561.2847
P1216014 89 (6.230)
2: TOF MS ES+
1.18e3
Ac-Gly-Pro-AA-Phe-NH2
b1 b2 b3
y3 y2 y1
100.0
197.1
395.1
460.2
363.1
165.1
b3
395.1721
70.0646
b2
197.0943
296.1414
[M+1]
559.2731
396.1819
542.2475
y3
460.2382
171.0901
268.1445
367.1857
y2
363.1784
514.2534
560.2828
198.0976
227.0836
297.1489
318.1629
368.1875
397.1858
443.2165
461.2411
515.2634
71.0720
b1
120.0826
169.0990
y1
172.0973
269.1515
m/z
%

5B': MW = 572.7, Purity = 89.0%, Yield = 14.4% [0.19 mg]

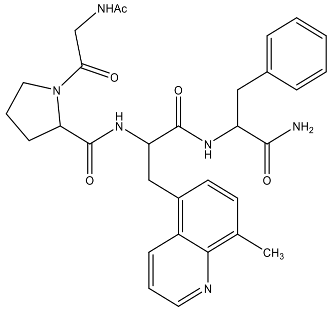

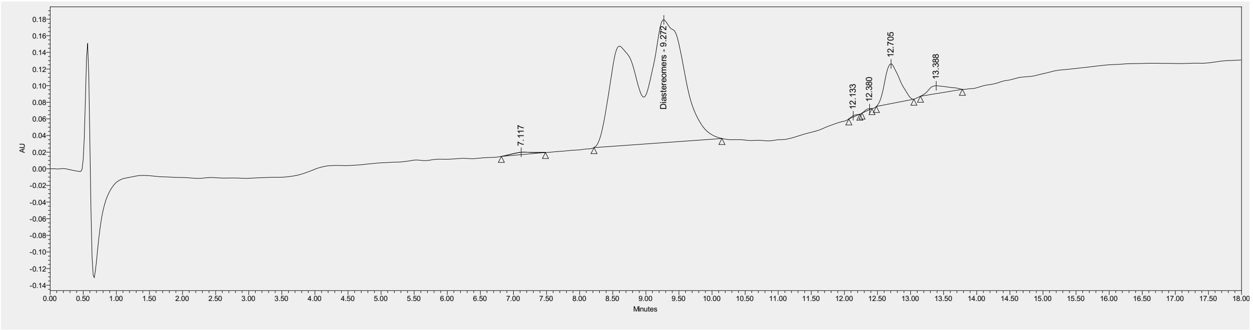

	Name	Retention Time	Area	% Area
1		7.117	59826	0.64
2	Diastereomers	9.272	8307687	88.95
3		12.133	9705	0.10
4		12.380	10083	0.11
5		12.705	754383	8.08
6		13.388	197753	2.12

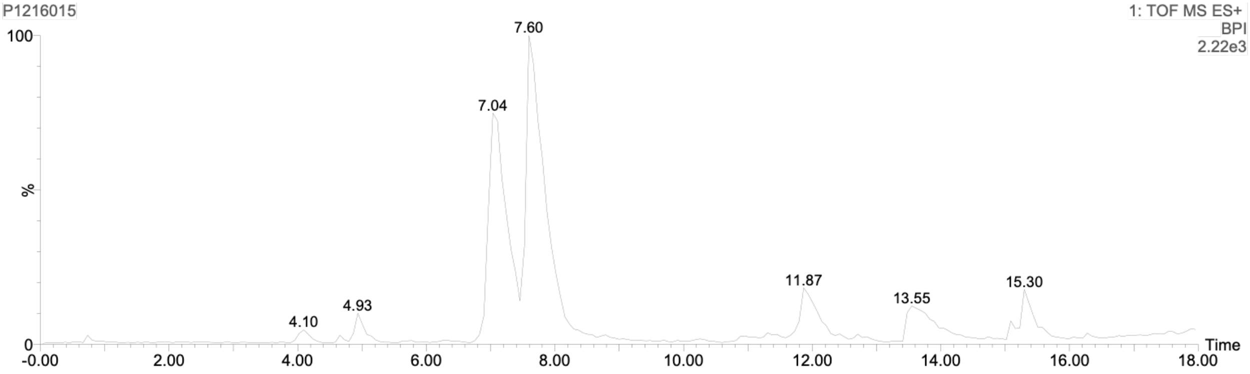

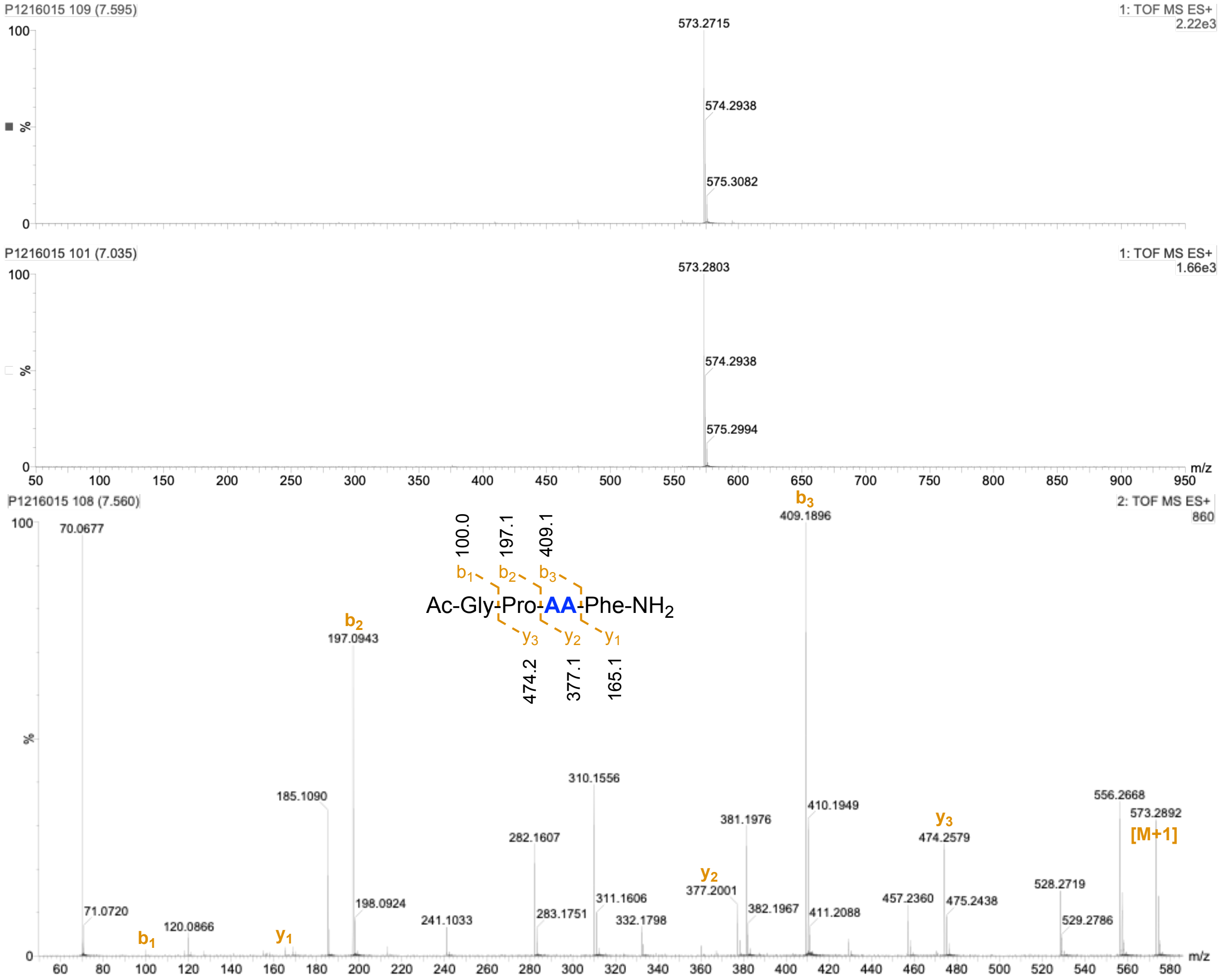
P1216015 109 (7.595)
1: TOF MS ES+
2.22e3
573.2715
574.2938
575.3082
P1216015 101 (7.035)
1: TOF MS ES+
1.66e3
573.2803
574.2938
575.2994
m/z
P1216015 108 (7.560)
2: TOF MS ES+
860
Ac-Gly-Pro-AA-Phe-NH2
b1 100.0
b2 197.1
b3 409.1
y3 474.2
y2 377.1
y1 165.1
70.0677
71.0720
120.0866
185.1090
b2
197.0943
198.0924
241.1033
282.1607
283.1751
310.1556
311.1606
332.1798
y2
377.2001
381.1976
382.1967
b3
409.1896
410.1949
411.2088
457.2360
y3
474.2579
475.2438
528.2719
529.2786
556.2668
573.2892
[M+1]
b1
y1
m/z

6B’: MW = 602.7, Purity = 78.2%, Yield = 14.8% [0.21 mg]

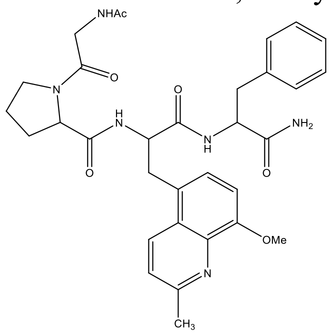

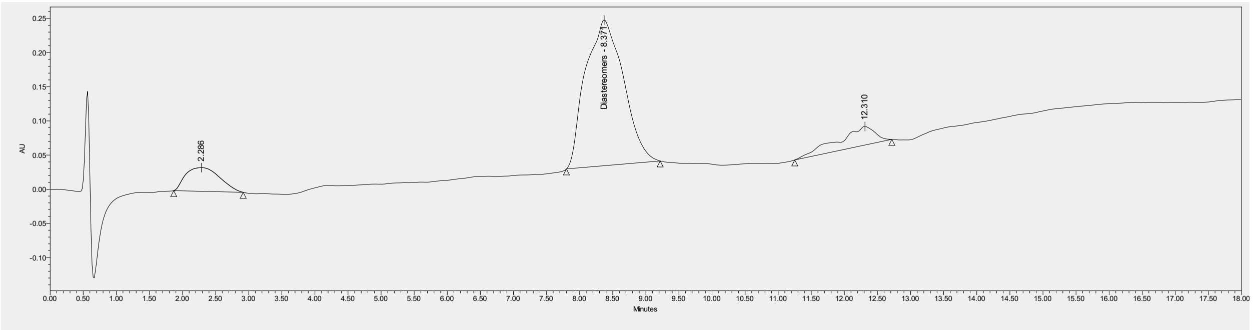

	Name	Retention Time	Area	% Area
1		2.286	1244568	11.45
2	Diastereomers	8.371	8500843	78.24
3		12.310	1119486	10.30

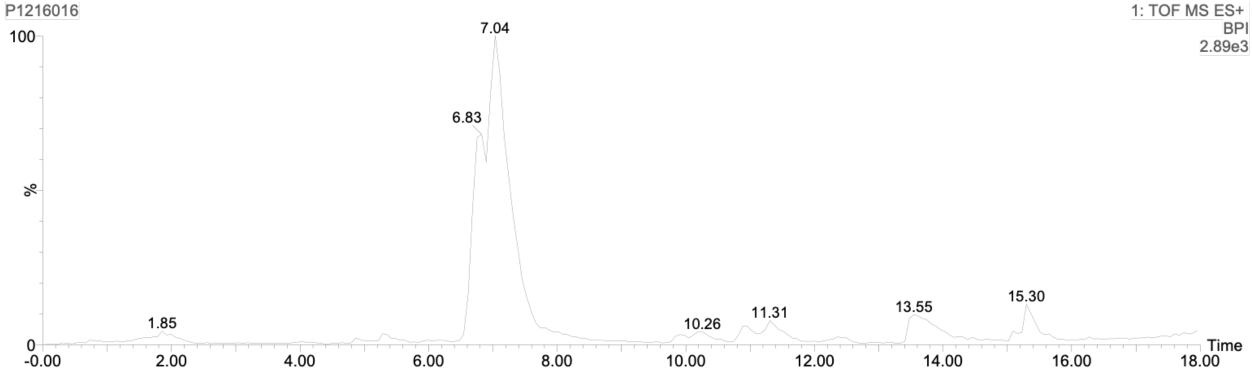

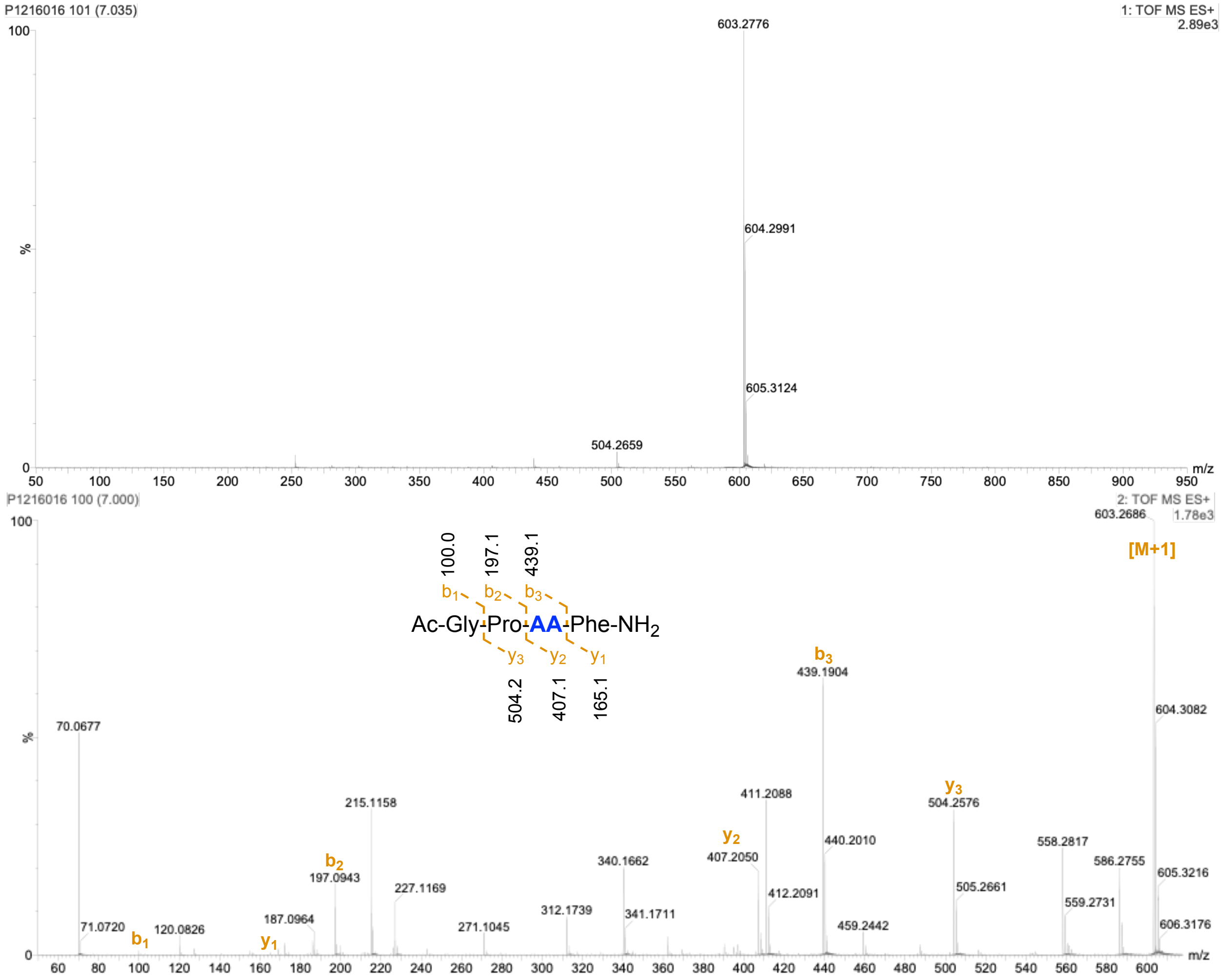

P1216016 101 (7.035)
1: TOF MS ES+
2.89e3
603.2776
604.2991
605.3124
504.2659
m/z
P1216016 100 (7.000)
2: TOF MS ES+
1.78e3
603.2686
[M+1]
100.0
197.1
439.1
b1 b2 b3
Ac-Gly-Pro-AA-Phe-NH2
y3 y2 y1
504.2
407.1
165.1
b3
439.1904
604.3082
70.0677
411.2088
y3
504.2576
215.1158
y2
407.2050
440.2010
558.2817
586.2755
b2
197.0943
340.1662
605.3216
227.1169
412.2091
505.2661
559.2731
312.1739
341.1711
187.0964
71.0720
b1
120.0826
y1
271.1045
459.2442
606.3176
m/z

7B': MW = 558.6, Purity = 92.0%, Yield = 16.7% [0.22 mg]

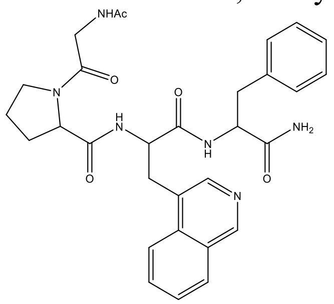

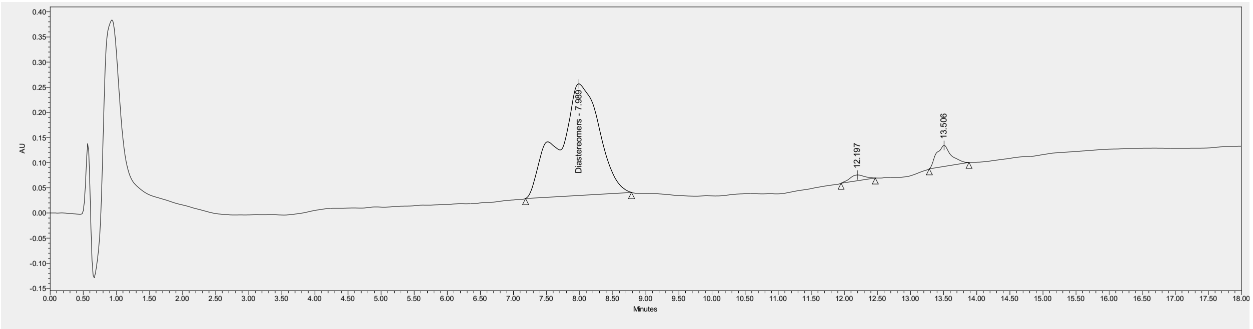

	Name	Retention Time	Area	% Area
1	Diastereomers	7.989	9599552	91.98
2		12.197	179405	1.72
3		13.506	657199	6.30

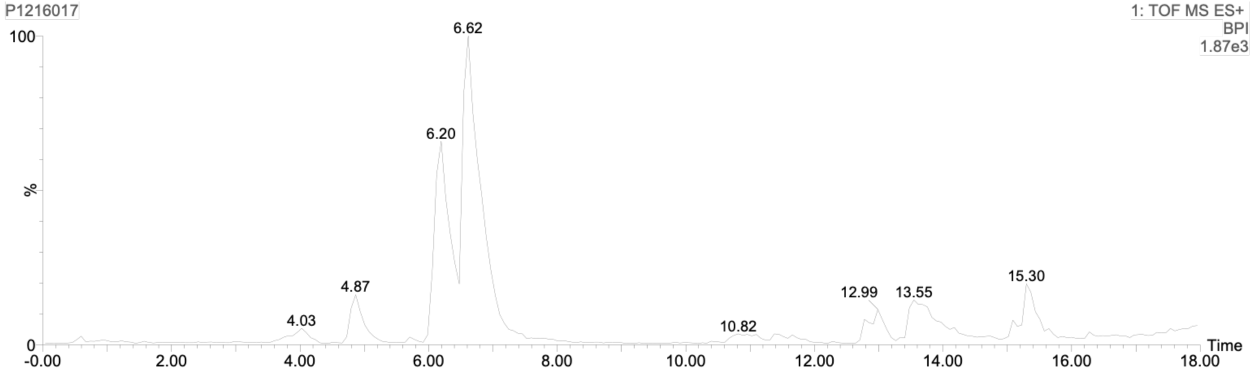

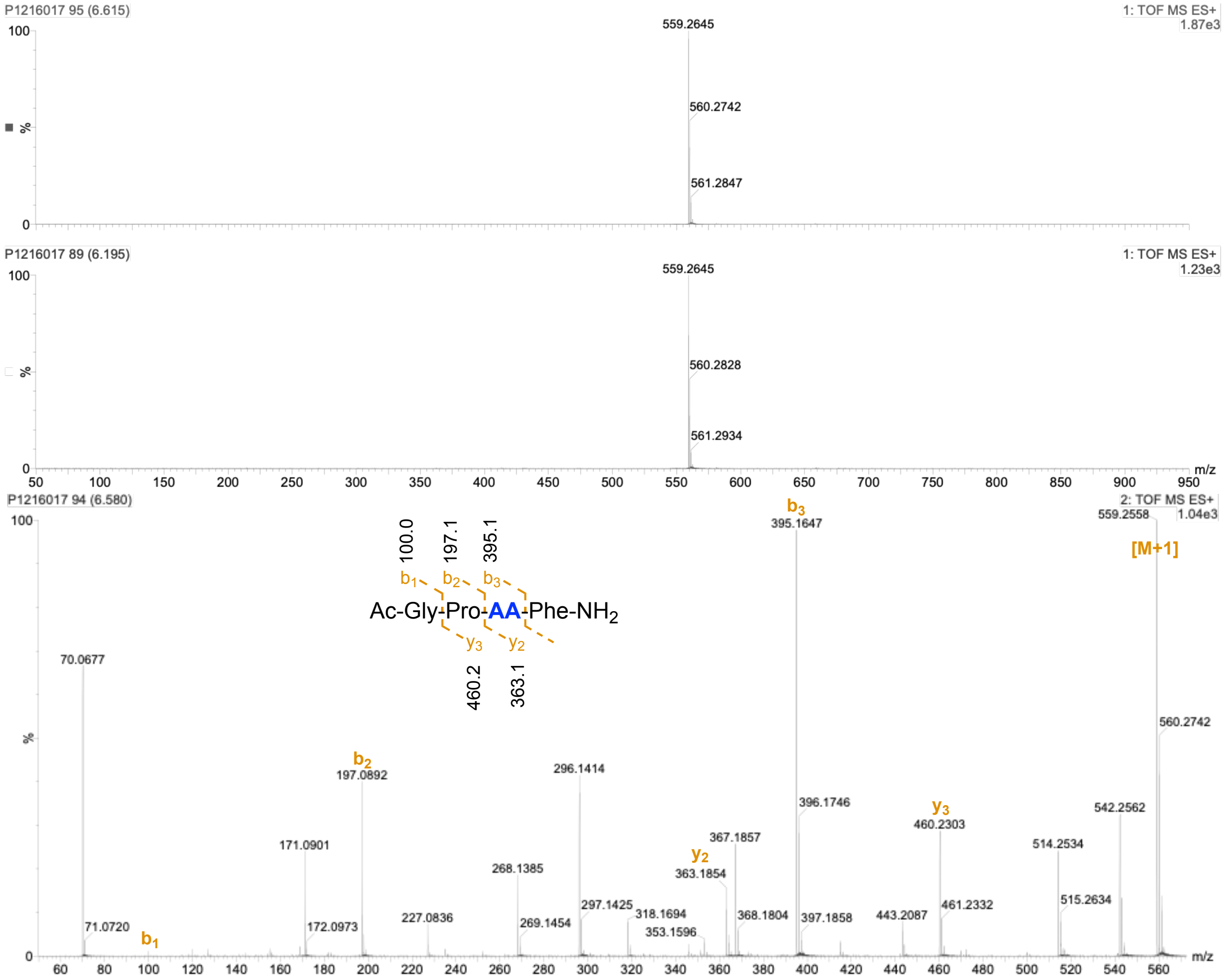
P1216017 95 (6.615)
1: TOF MS ES+
1.87e3
559.2645
560.2742
561.2847
P1216017 89 (6.195)
1: TOF MS ES+
1.23e3
559.2645
560.2828
561.2934
m/z
P1216017 94 (6.580)
2: TOF MS ES+
1.04e3
Ac-Gly-Pro-AA-Phe-NH2
b1 b2 b3
y3 y2
100.0
197.1
395.1
460.2
363.1
b3
395.1647
559.2558
[M+1]
70.0677
560.2742
b2
197.0892
296.1414
396.1746
y3
460.2303
542.2562
171.0901
367.1857
514.2534
y2
363.1854
268.1385
297.1425
318.1694
461.2332
515.2634
71.0720
b1
172.0973
227.0836
269.1454
353.1596
368.1804
397.1858
443.2087
m/z

8B': MW = 558.6, Purity = 92.3%, Yield = 28.8% [0.38 mg]

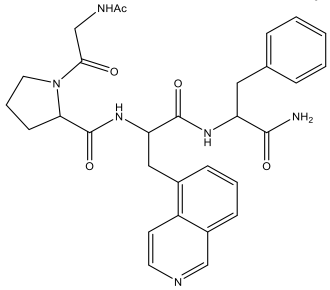

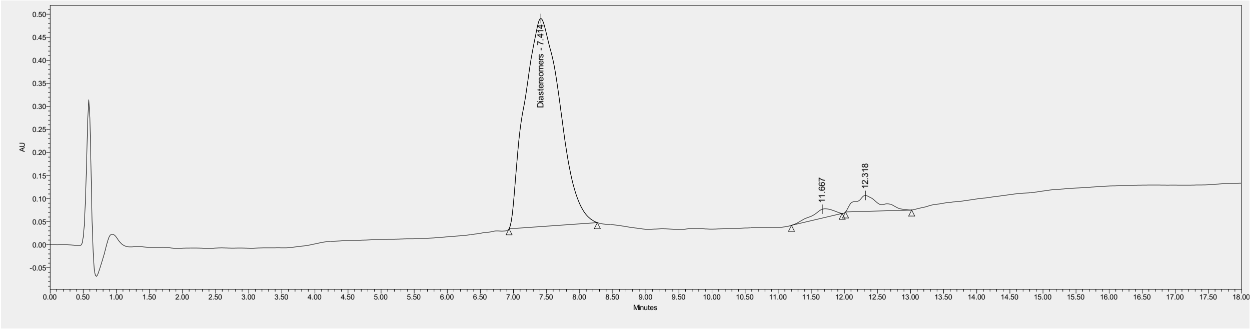

	Name	Retention Time	Area	% Area
1	Diastereomers	7.414	16593572	92.32
2		11.667	436159	2.43
3		12.318	944441	5.25

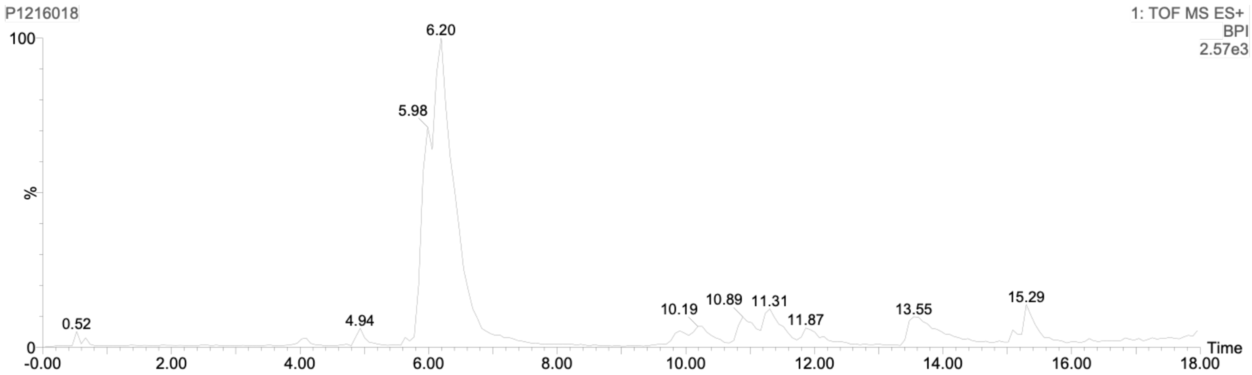

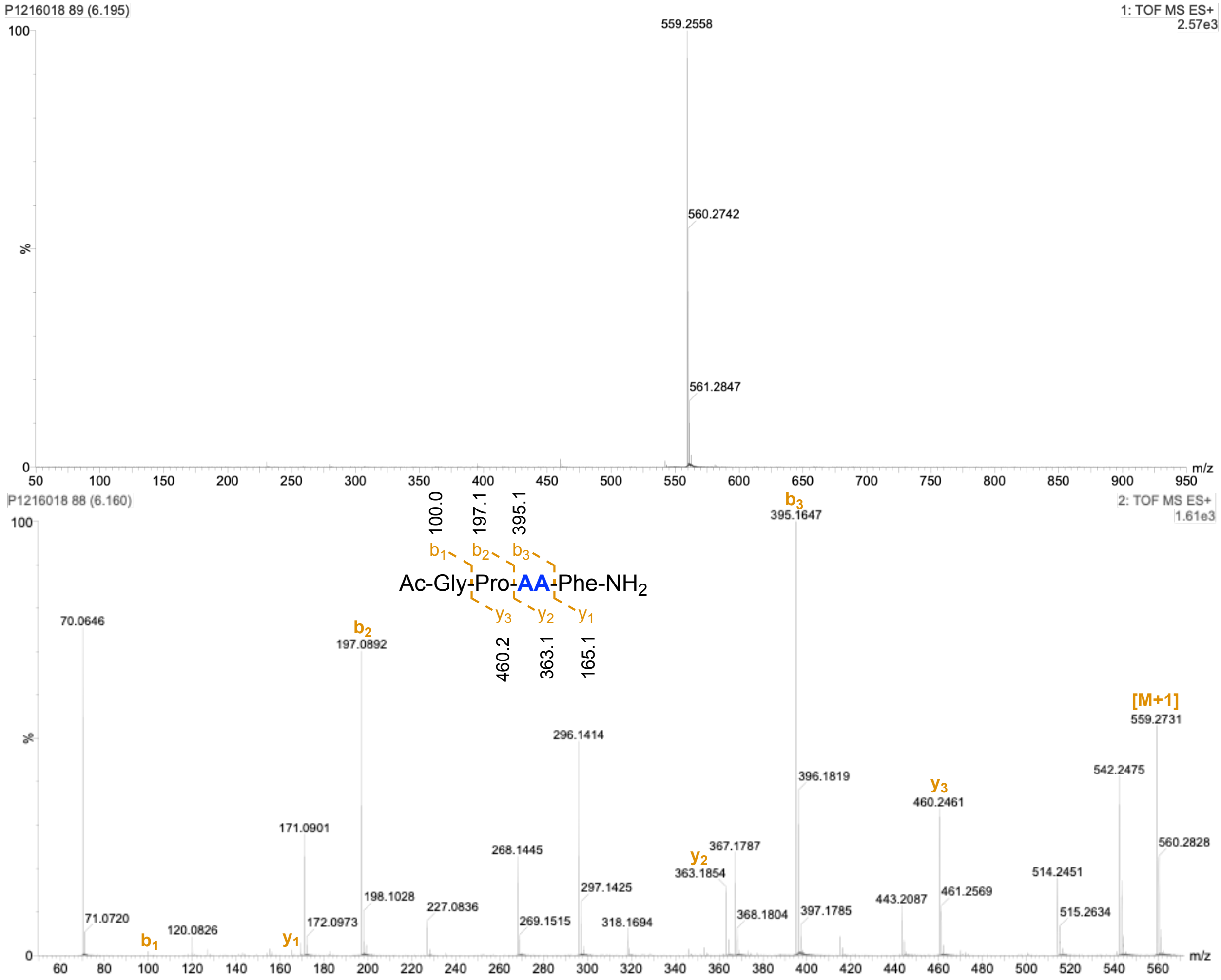

P1216018 89 (6.195)
1: TOF MS ES+
2.57e3
559.2558
560.2742
561.2847
%
m/z
P1216018 88 (6.160)
2: TOF MS ES+
1.61e3
Ac-Gly-Pro-AA-Phe-NH2
100.0
197.1
395.1
460.2
363.1
165.1
b1
b2
b3
y3
y2
y1
70.0646
71.0720
120.0826
171.0901
172.0973
197.0892
198.1028
227.0836
268.1445
269.1515
296.1414
297.1425
318.1694
363.1854
367.1787
368.1804
395.1647
396.1819
397.1785
443.2087
460.2461
461.2569
514.2451
515.2634
542.2475
[M+1]
559.2731
560.2828

9B’: MW = 558.6, Purity = 90.4%, Yield = 19.5% [0.25 mg]

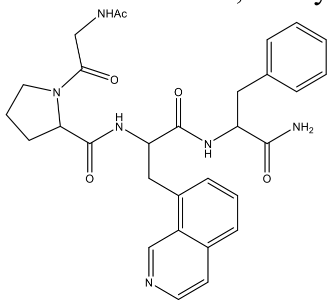

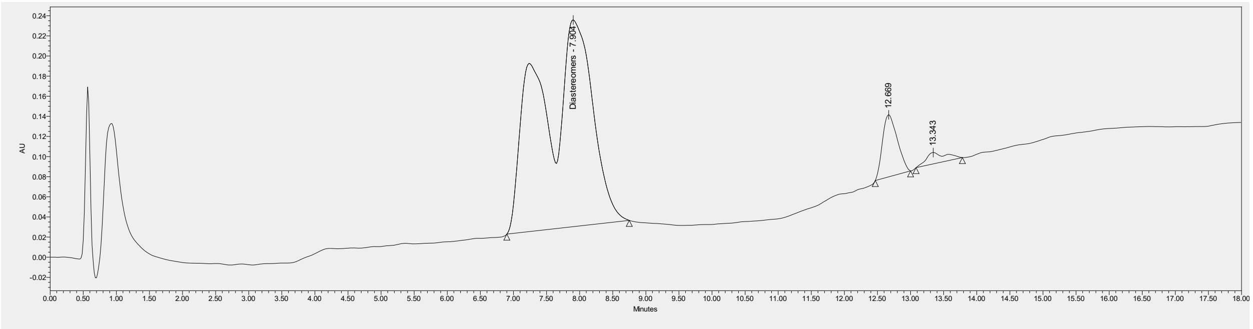

	Name	Retention Time	Area	% Area
1	Diastereomers	7.904	11205820	90.38
2		12.669	960905	7.75
3		13.343	231676	1.87

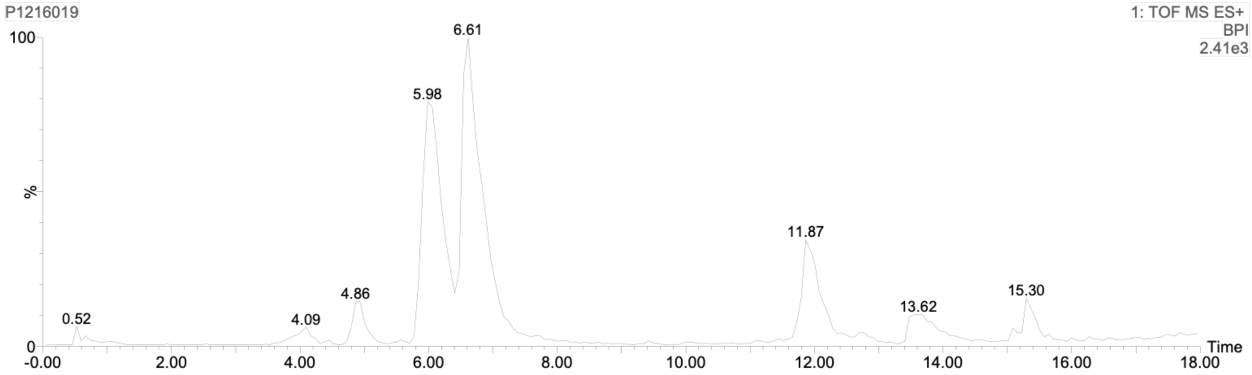

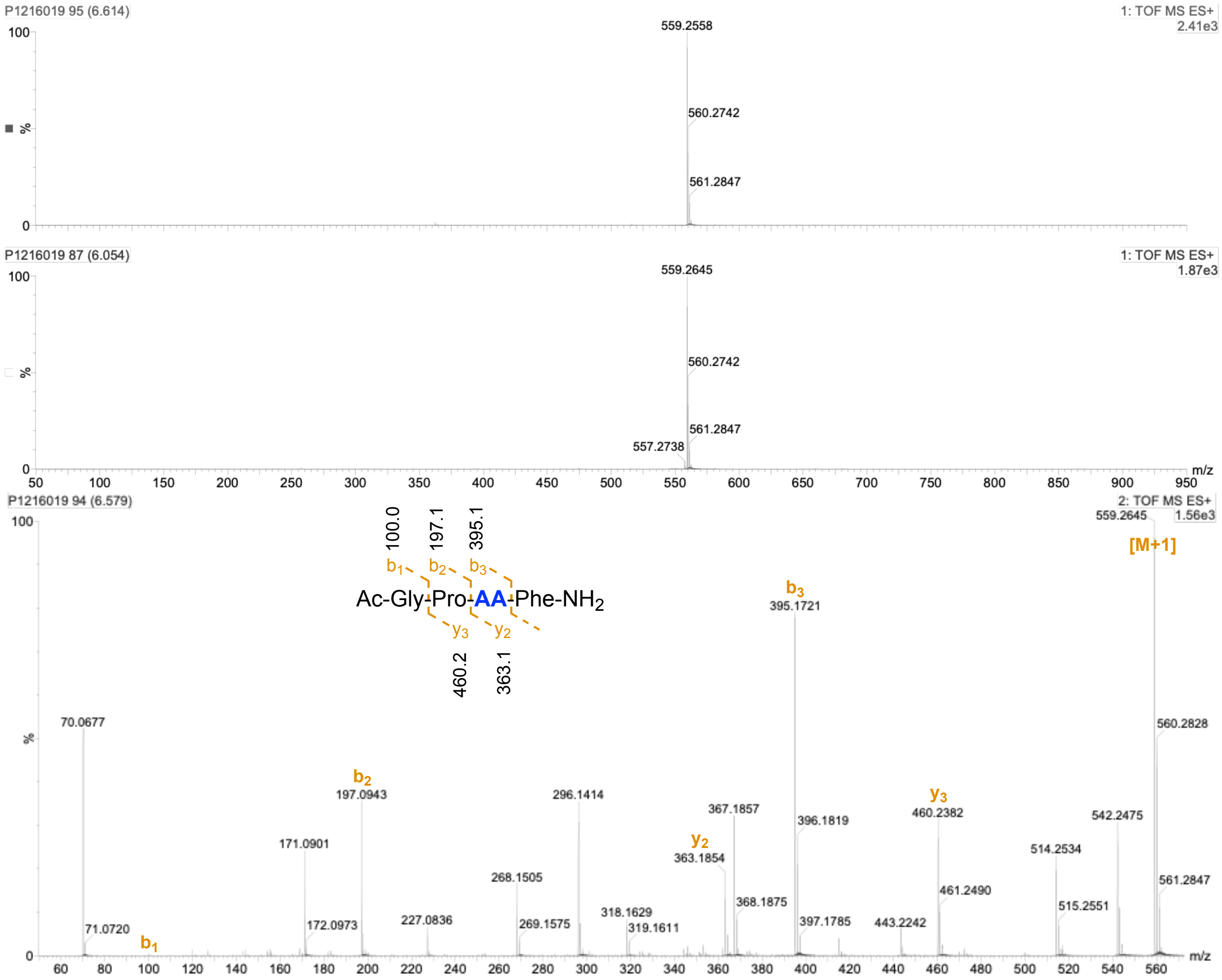

P1216019 95 (6.614)
1: TOF MS ES+
2.41e3
559.2558
560.2742
561.2847
P1216019 87 (6.054)
1: TOF MS ES+
1.87e3
559.2645
560.2742
561.2847
557.2738
m/z
P1216019 94 (6.579)
2: TOF MS ES+
559.2645
1.56e3
[M+1]
Ac-Gly-Pro-AA-Phe-NH2
b1 b2 b3 y3 y2
100.0
197.1
395.1
460.2
363.1
b3
395.1721
70.0677
560.2828
b2
197.0943
296.1414
367.1857
396.1819
y3
460.2382
542.2475
y2
363.1854
171.0901
514.2534
268.1505
461.2490
561.2847
368.1875
515.2551
318.1629
71.0720
b1
172.0973
227.0836
269.1575
319.1611
397.1785
443.2242
m/z

10B': MW = 558.6, Purity = 73.6%, Yield = 6.0% [0.078 mg]

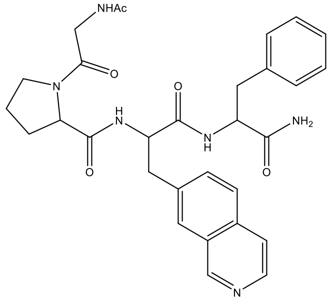

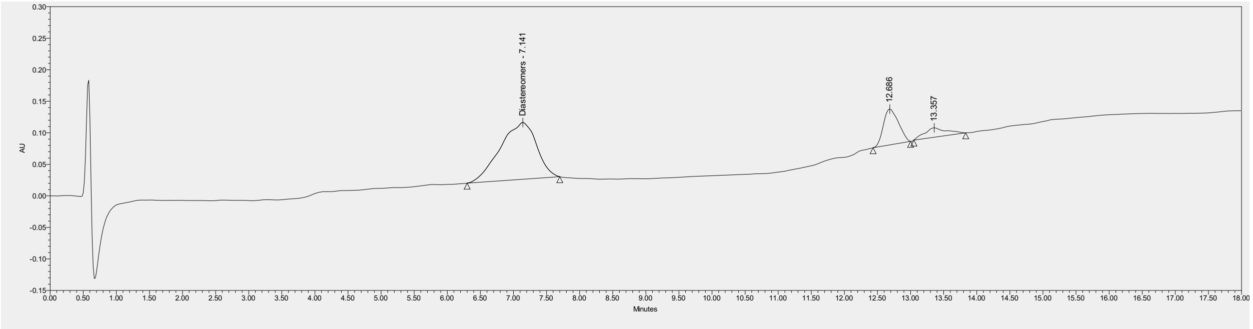

	Name	Retention Time	Area	% Area
1	Diastereomers	7.141	3459832	73.60
2		12.686	898364	19.11
3		13.357	342932	7.29

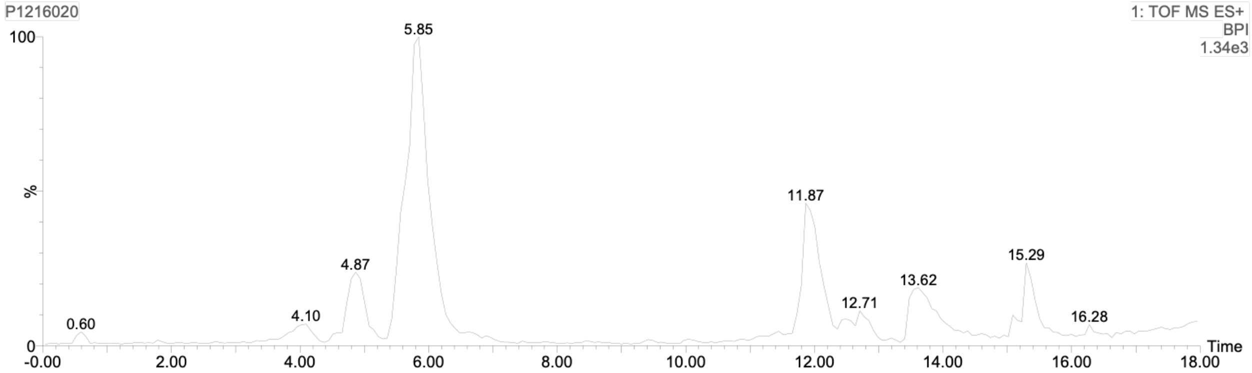

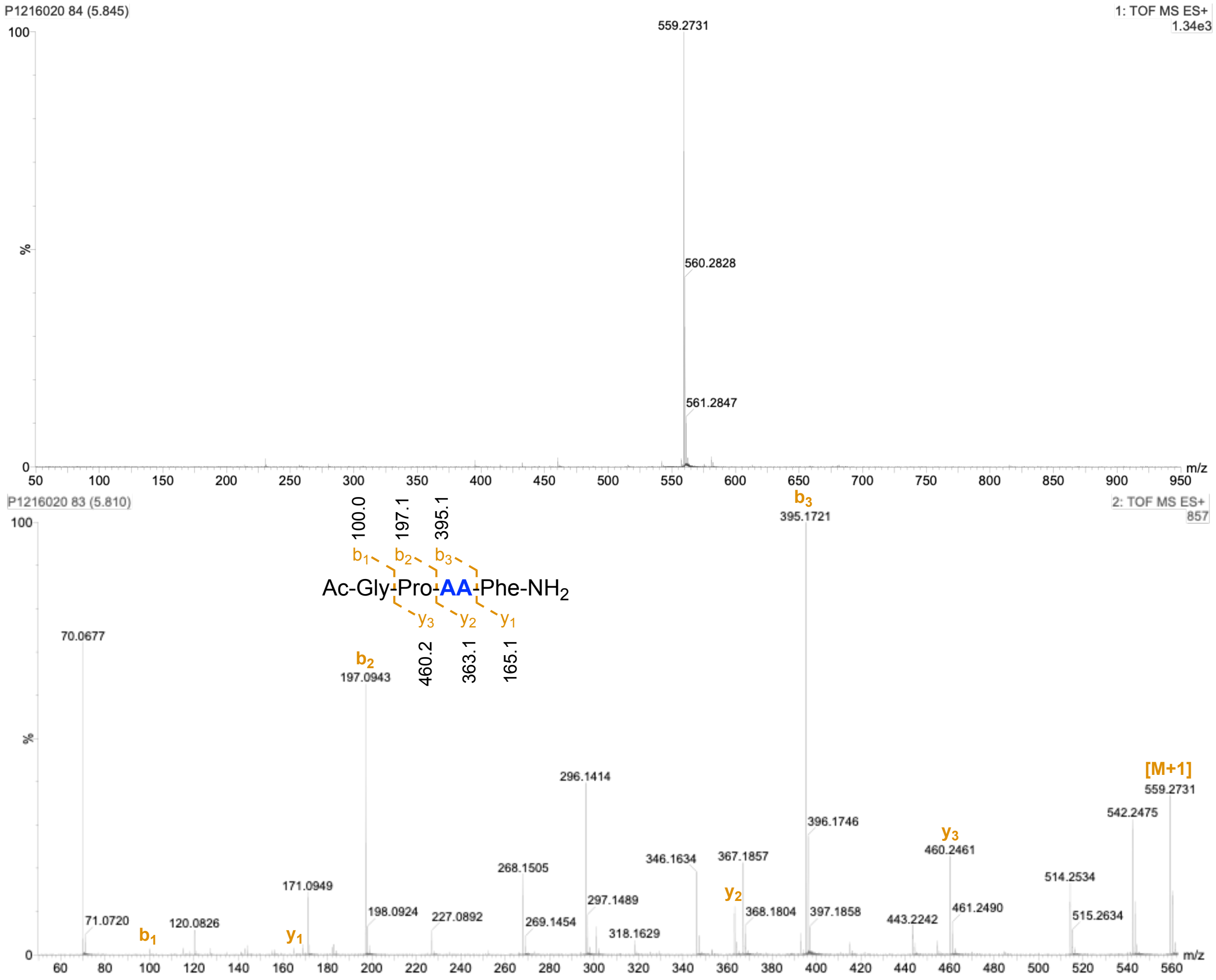
P1216020 84 (5.845)
1: TOF MS ES+
1.34e3
559.2731
560.2828
561.2847
%
m/z
P1216020 83 (5.810)
2: TOF MS ES+
857
100.0
197.1
395.1
b1
b2
b3
Ac-Gly-Pro-AA-Phe-NH2
y3
y2
y1
460.2
363.1
165.1
b3
395.1721
70.0677
b2
197.0943
296.1414
[M+1]
559.2731
542.2475
396.1746
y3
460.2461
346.1634
367.1857
268.1505
514.2534
171.0949
y2
297.1489
461.2490
198.0924
368.1804
397.1858
71.0720
227.0892
443.2242
515.2634
120.0826
269.1454
b1
y1
318.1629

11B': MW = 552.6, Purity = 46.0%, Yield = 4.6% [0.059 mg]

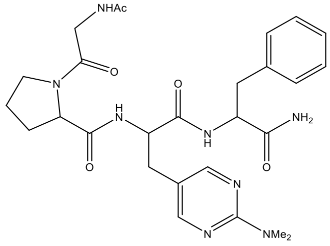

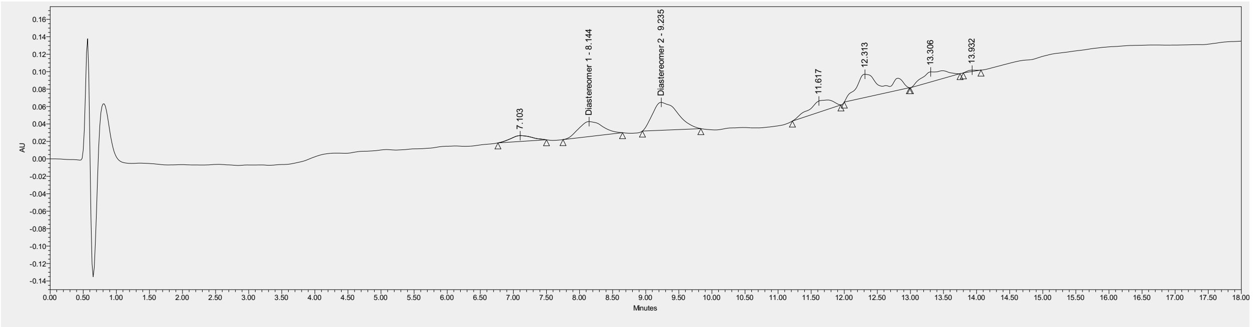

	Name	Retention Time	Area	% Area
1		7.103	150863	5.40
2	Diastereomer 1	8.144	436926	15.64
3	Diastereomer 2	9.235	847343	30.33
4		11.617	310463	11.11
5		12.313	741239	26.53
6		13.306	291918	10.45
7		13.932	14989	0.54

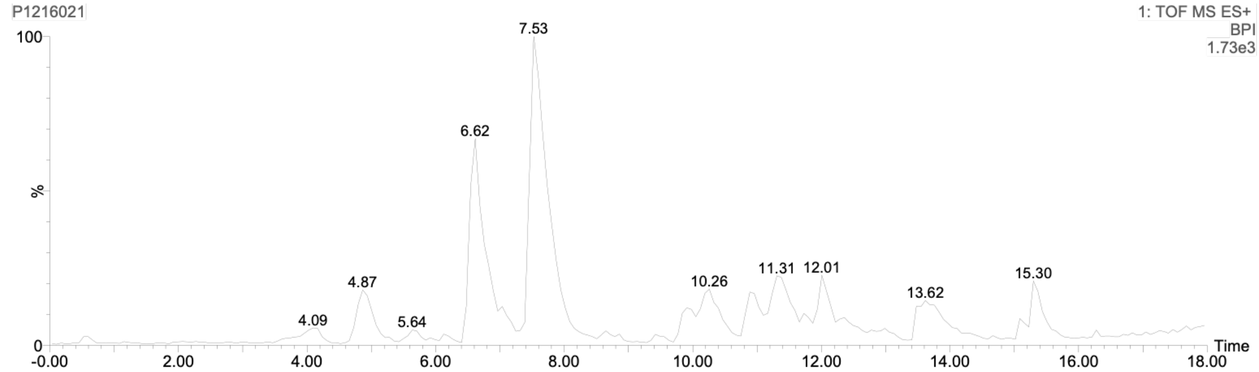

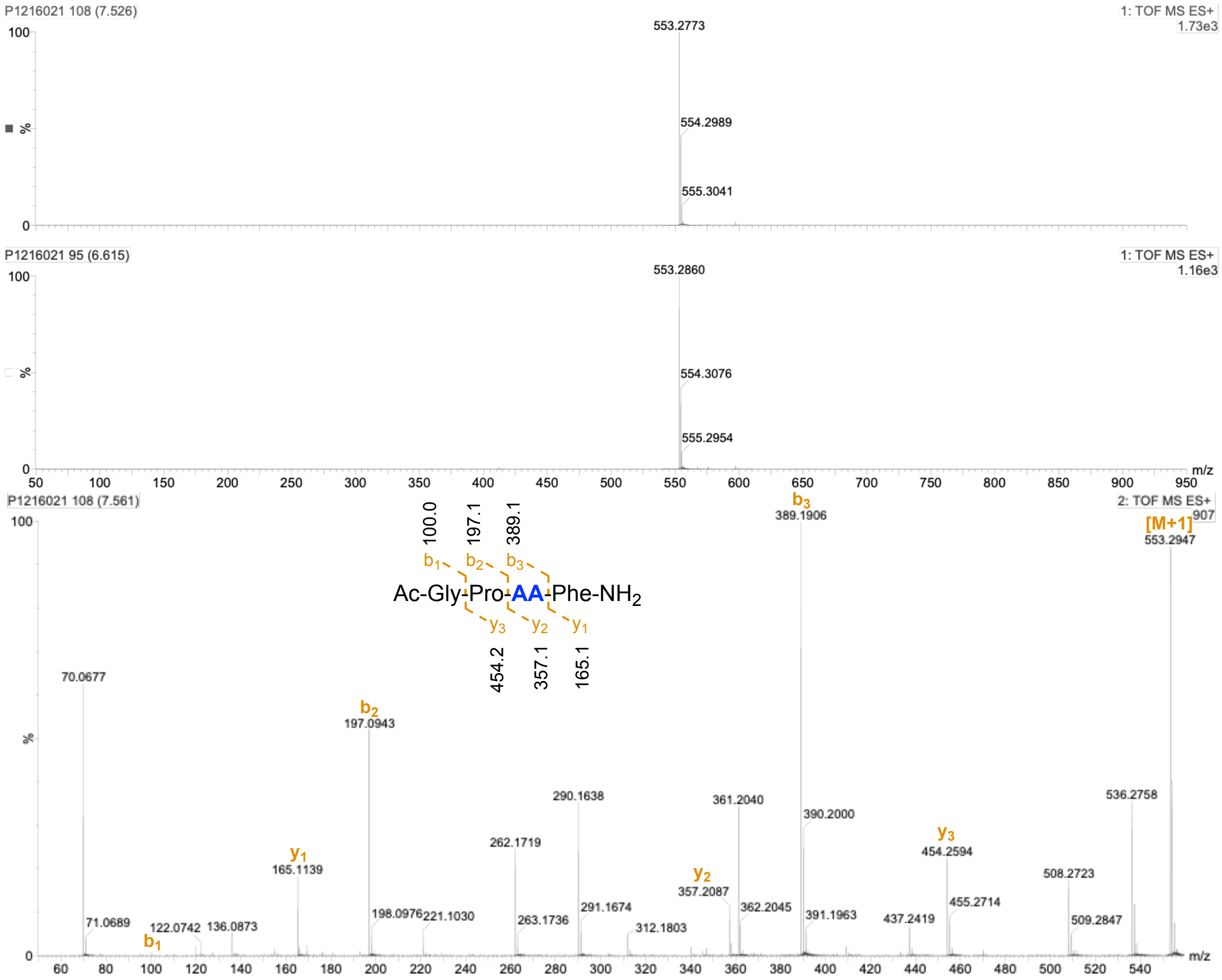
P1216021 108 (7.526)
1: TOF MS ES+
1.73e3
553.2773
554.2989
555.3041
P1216021 95 (6.615)
1: TOF MS ES+
1.16e3
553.2860
554.3076
555.2954
m/z
P1216021 108 (7.561)
2: TOF MS ES+
907
Ac-Gly-Pro-AA-Phe-NH2
b1 b2 b3
y3 y2 y1
100.0
197.1
389.1
454.2
357.1
165.1
b3
389.1906
[M+1]
553.2947
70.0677
b2
197.0943
290.1638
361.2040
390.2000
536.2758
262.1719
y3
454.2594
y1
165.1139
y2
357.2087
508.2723
71.0689
122.0742
136.0873
b1
198.0976
221.1030
263.1736
291.1674
312.1803
362.2045
391.1963
437.2419
455.2714
509.2847
%
m/z

12B’: MW = 555.6, Purity = 42.8%, Yield = 4.0% [0.052 mg]

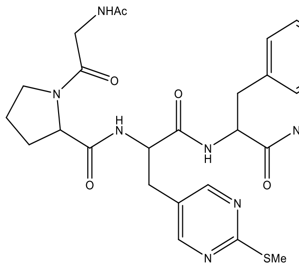

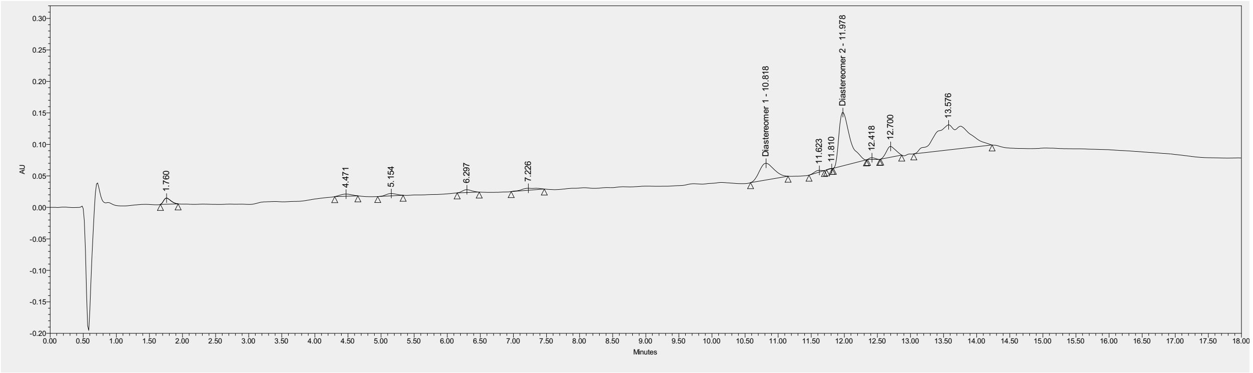

	Name	Retention Time	Area	% Area
1		1.760	73132	2.24
2		4.471	41225	1.26
3		5.154	40381	1.24
4		6.297	44364	1.36
5		7.226	49957	1.53
6	Diastereomer 1	10.818	410558	12.56
7		11.623	22421	0.69
8		11.810	1905	0.06
9	Diastereomer 2	11.978	989484	30.27
10		12.418	16523	0.51
11		12.700	165337	5.06
12		13.576	1413453	43.24

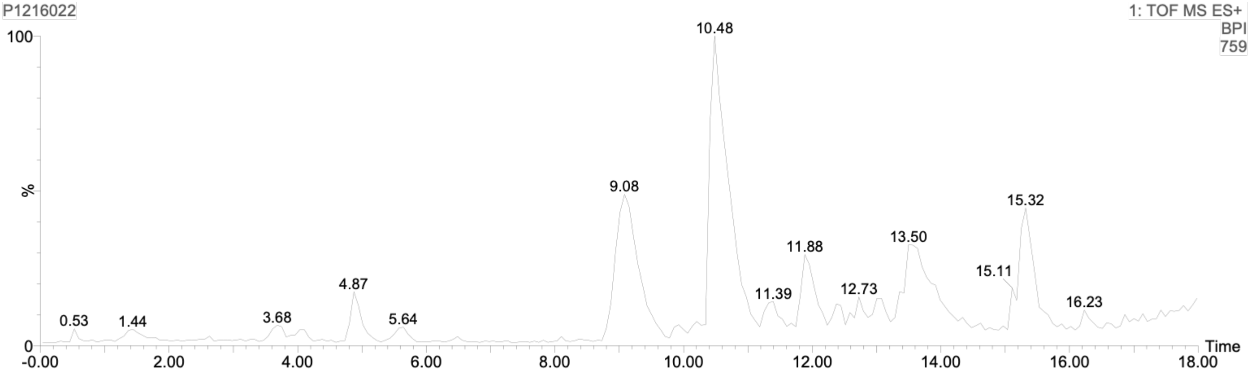

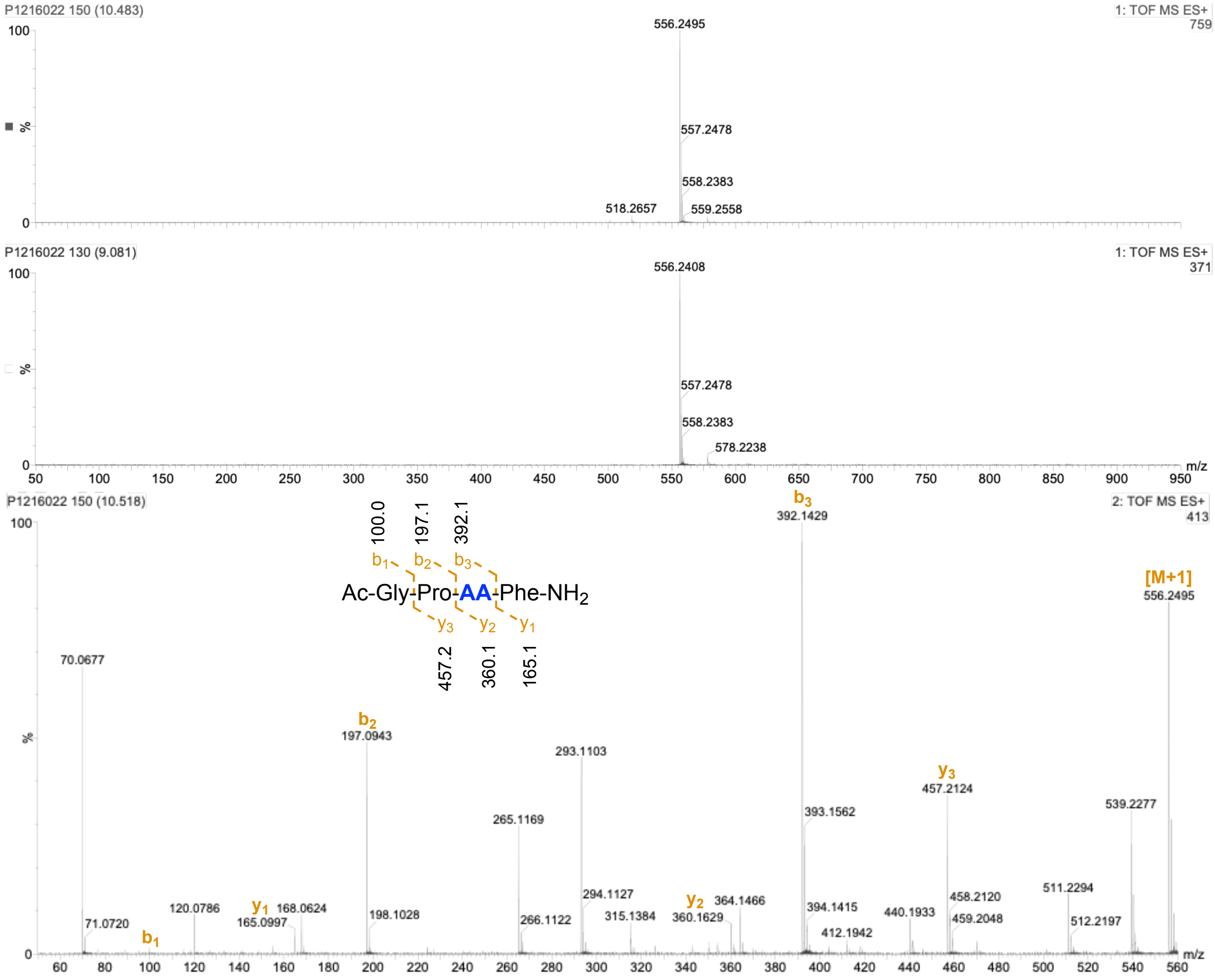

P1216022 150 (10.483)
1: TOF MS ES+
759
556.2495
557.2478
558.2383
518.2657
559.2558
P1216022 130 (9.081)
1: TOF MS ES+
371
556.2408
557.2478
558.2383
578.2238
m/z
P1216022 150 (10.518)
2: TOF MS ES+
413
Ac-Gly-Pro-AA-Phe-NH2
100.0
197.1
392.1
457.2
360.1
165.1
b3
392.1429
[M+1]
556.2495
70.0677
b2
197.0943
293.1103
y3
457.2124
539.2277
265.1169
393.1562
511.2294
294.1127
y2
364.1466
458.2120
120.0786
y1
168.0624
198.1028
394.1415
440.1933
71.0720
165.0997
266.1122
315.1384
360.1629
459.2048
512.2197
b1
412.1942
%

1C': MW = 601.7, Purity = 86.1%, Yield = 5.5% [0.078 mg]

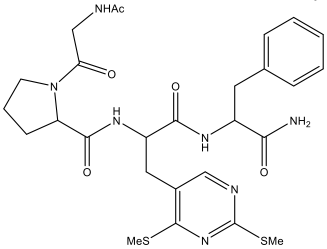

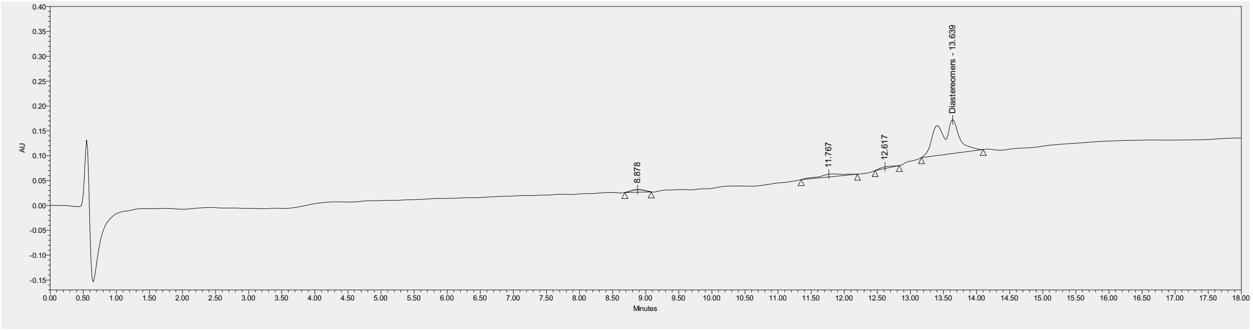

	Name	Retention Time	Area	% Area
1		8.878	72076	4.02
2		11.767	133926	7.47
3		12.617	42934	2.39
4	Diastereomers	13.639	1543967	86.12

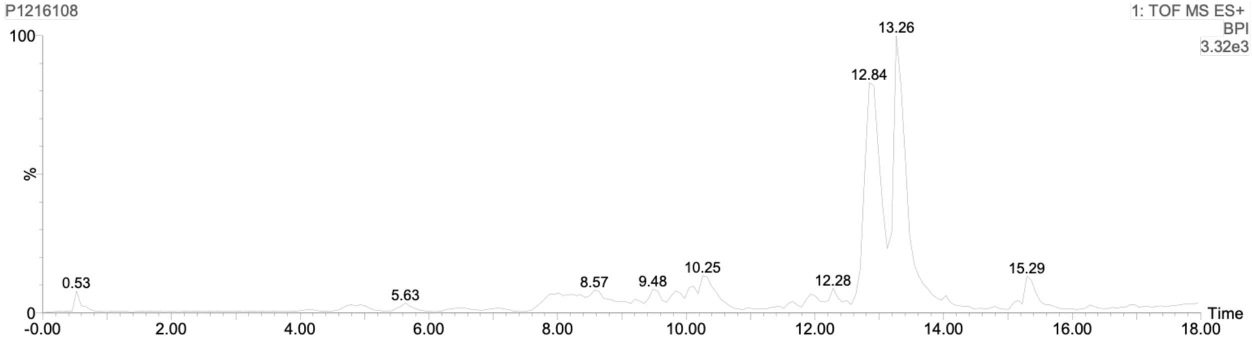

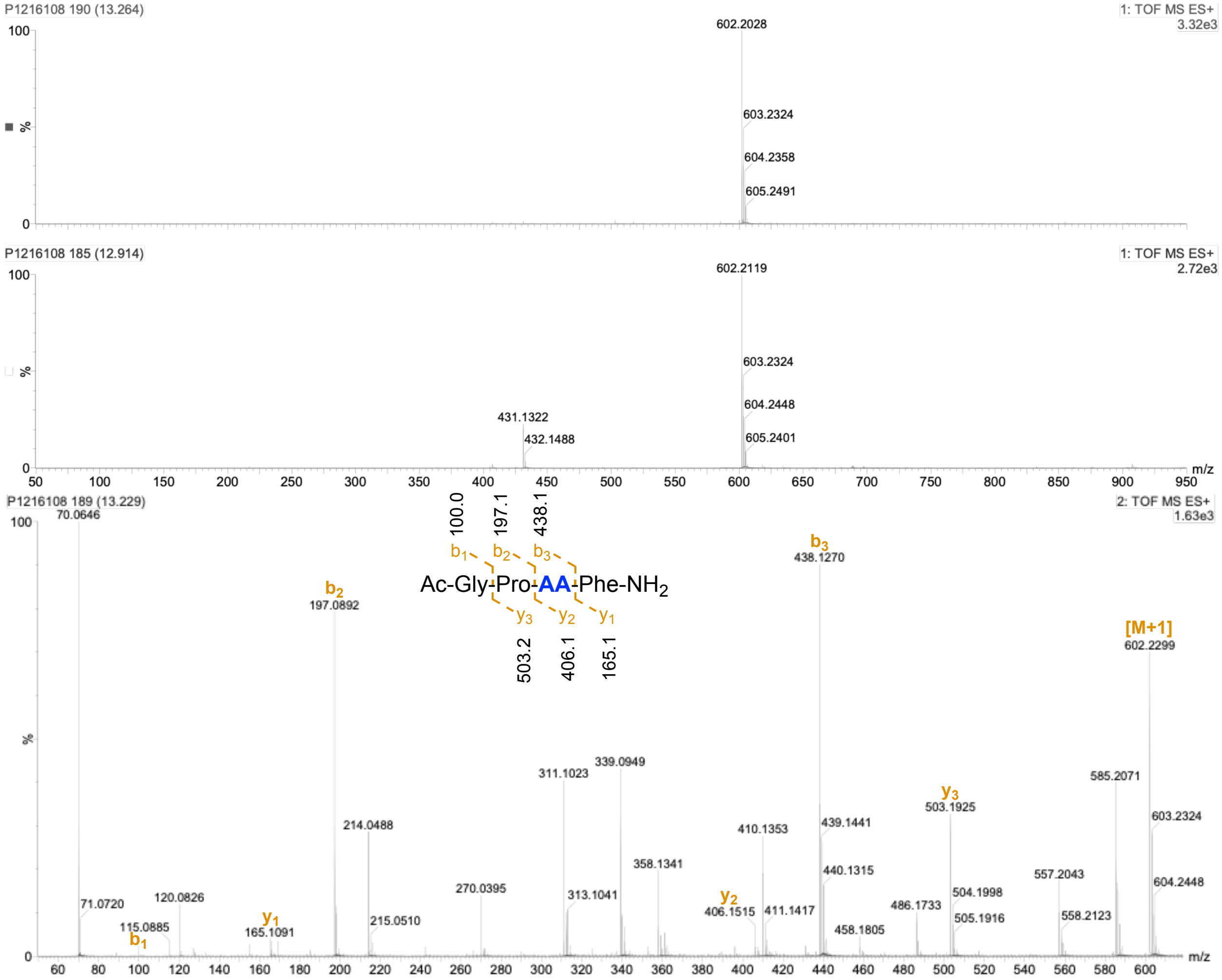

P1216108 190 (13.264)
1: TOF MS ES+
3.32e3
602.2028
603.2324
604.2358
605.2491
P1216108 185 (12.914)
1: TOF MS ES+
2.72e3
602.2119
603.2324
604.2448
605.2401
431.1322
432.1488
m/z
P1216108 189 (13.229)
2: TOF MS ES+
1.63e3
70.0646
100.0
197.1
438.1
Ac-Gly-Pro-AA-Phe-NH2
503.2
406.1
165.1
b3
438.1270
b2
197.0892
[M+1]
602.2299
339.0949
311.1023
585.2071
y3
503.1925
603.2324
214.0488
410.1353
439.1441
358.1341
440.1315
557.2043
604.2448
270.0395
504.1998
120.0826
313.1041
71.0720
y2
406.1515
411.1417
486.1733
505.1916
558.2123
115.0885
b1
y1
165.1091
215.0510
458.1805
m/z

2C’: MW = 569.6, Purity = 41.7%, Yield = 2.7% [0.036 mg]

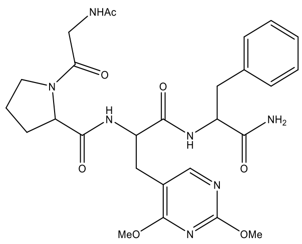

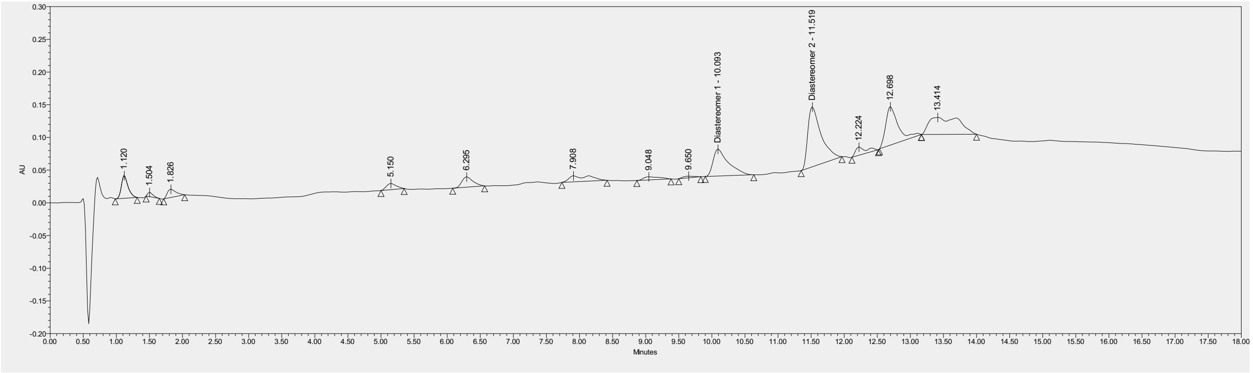

	Name	Retention Time	Area	% Area
1		1.120	244153	5.22
2		1.504	35955	0.77
3		1.826	116045	2.48
4		5.150	98509	2.11
5		6.295	184294	3.94
6		7.908	198591	4.25
7		9.048	81194	1.74
8		9.650	30191	0.65
9	Diastereomer 1	10.093	660702	14.13
10	Diastereomer 2	11.519	1289024	27.58
11		12.224	139175	2.98
12		12.698	756886	16.19
13		13.414	839561	17.96

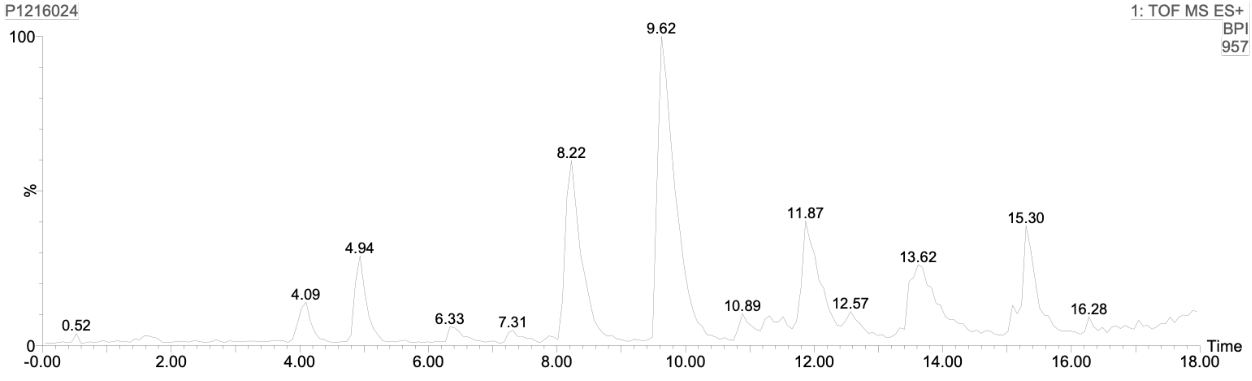

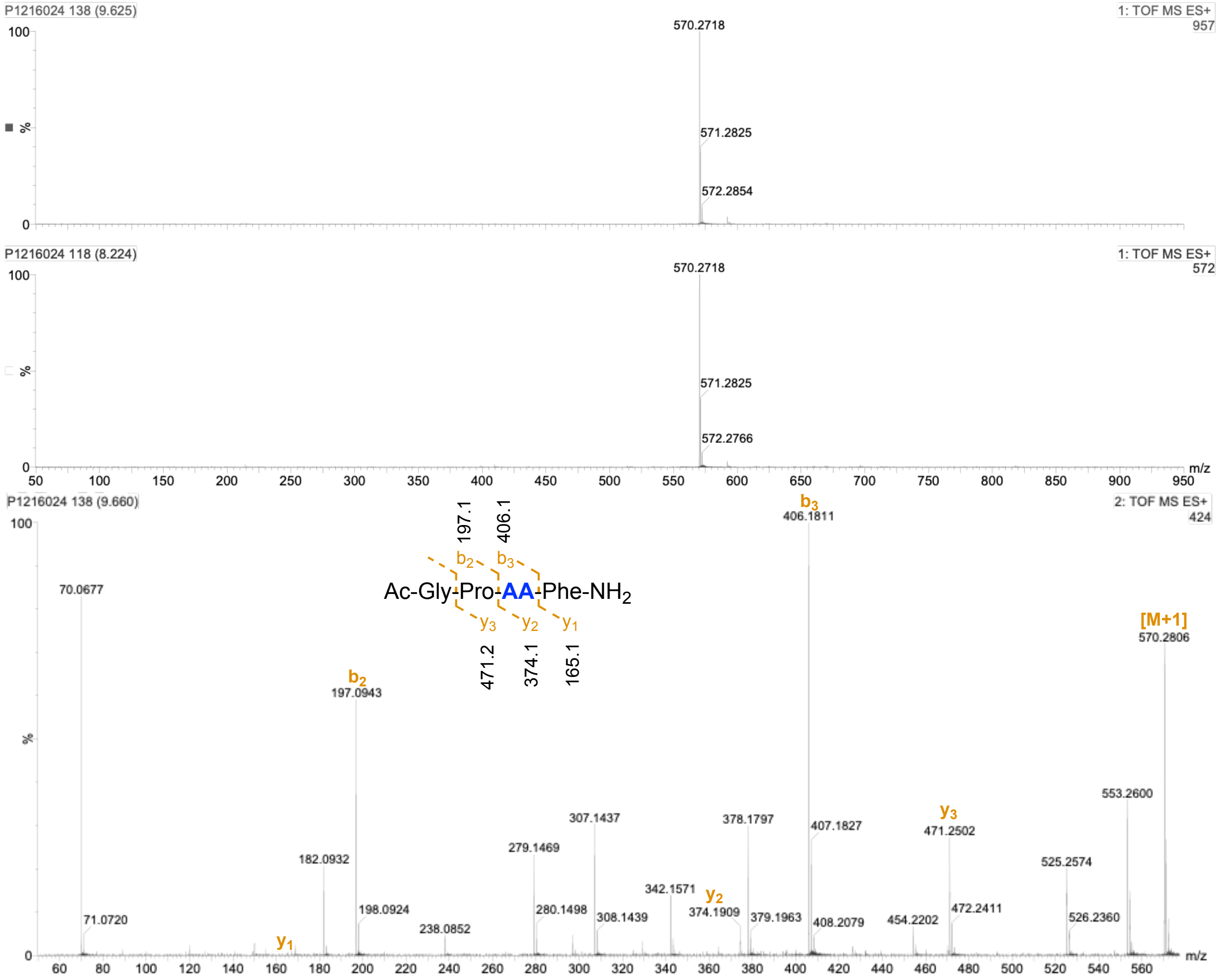
P1216024 138 (9.625)
1: TOF MS ES+
957
570.2718
571.2825
572.2854
P1216024 118 (8.224)
1: TOF MS ES+
572
570.2718
571.2825
572.2766
m/z
P1216024 138 (9.660)
2: TOF MS ES+
424
Ac-Gly-Pro-AA-Phe-NH2
b2 b3 y3 y2 y1
197.1
406.1
471.2
374.1
165.1
b3
406.1811
[M+1]
570.2806
70.0677
b2
197.0943
553.2600
307.1437
378.1797
407.1827
y3
471.2502
279.1469
182.0932
525.2574
342.1571
y2
374.1909
280.1498
308.1439
379.1963
472.2411
198.0924
71.0720
408.2079
454.2202
526.2360
238.0852
y1
m/z

3C’: MW = 560.7, Purity = 89.0%, Yield = 3.6% [0.047 mg]

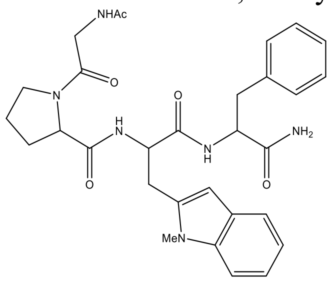

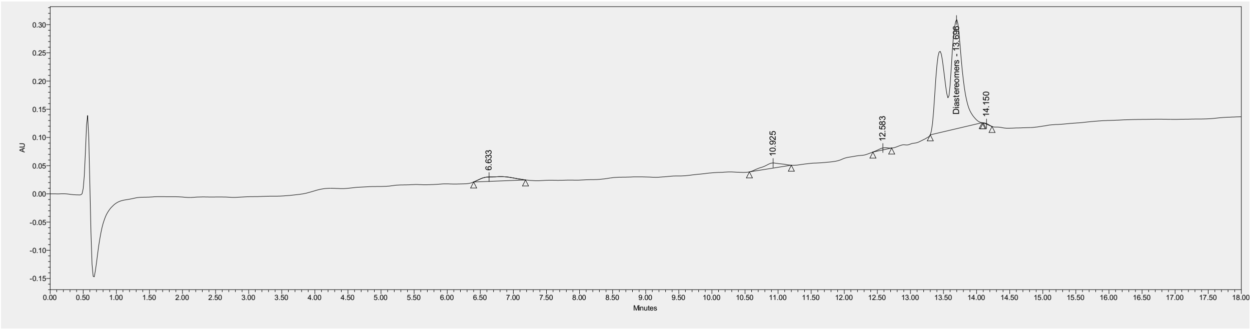

	Name	Retention Time	Area	% Area
1		6.633	246352	5.86
2		10.925	178094	4.24
3		12.583	32669	0.78
4	Diastereomers	13.696	3739460	89.00
5		14.150	4847	0.12

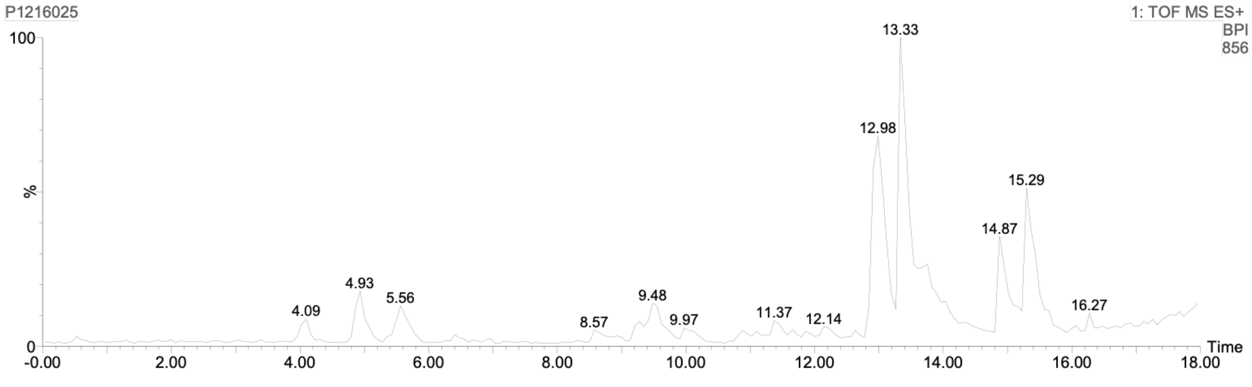

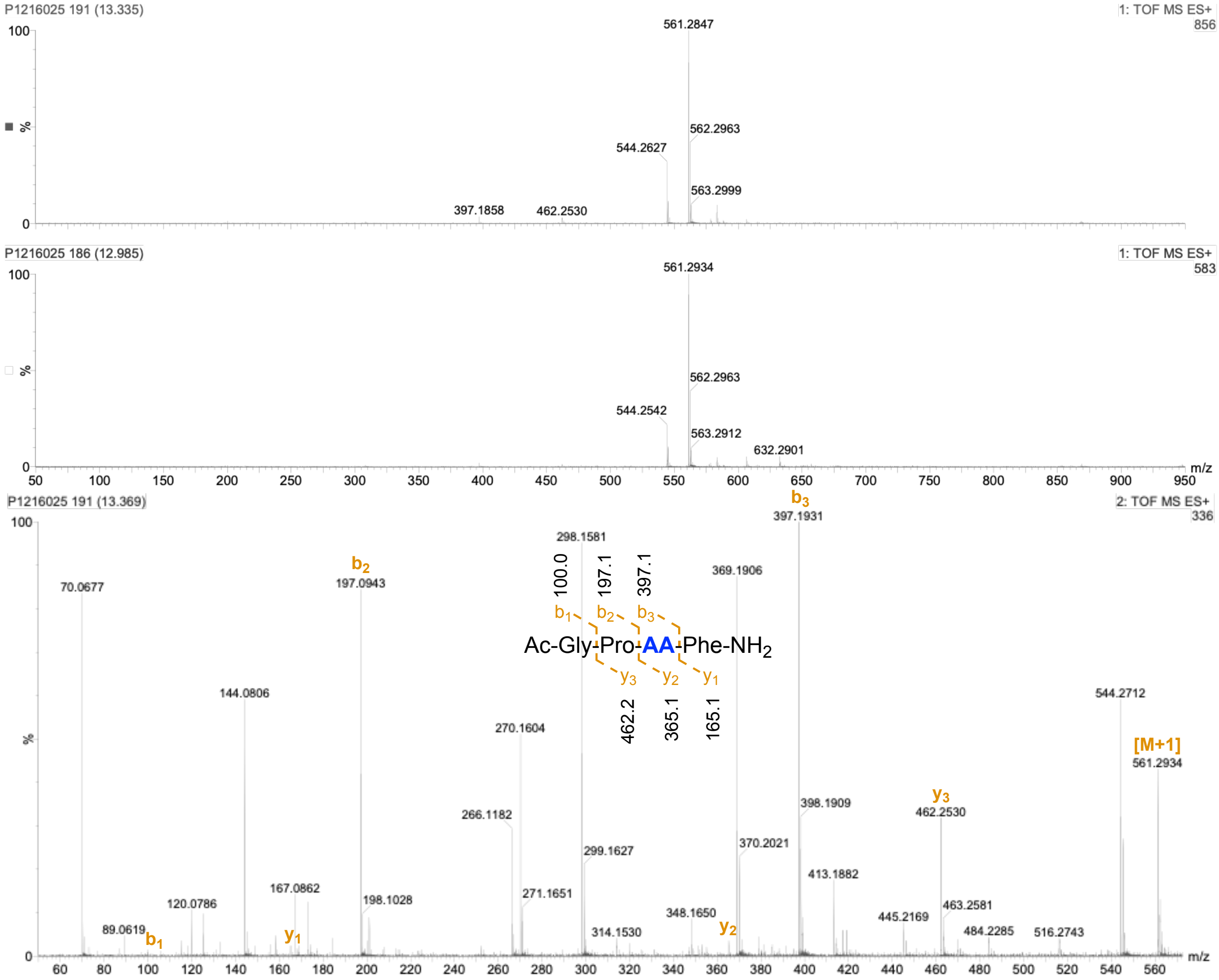
P1216025 191 (13.335)
1: TOF MS ES+
856
561.2847
562.2963
544.2627
563.2999
397.1858
462.2530
P1216025 186 (12.985)
1: TOF MS ES+
583
561.2934
562.2963
544.2542
563.2912
632.2901
m/z
P1216025 191 (13.369)
2: TOF MS ES+
336
b3
397.1931
298.1581
369.1906
b2
197.0943
70.0677
100.0
197.1
397.1
Ac-Gly-Pro-AA-Phe-NH2
462.2
365.1
165.1
144.0806
270.1604
544.2712
[M+1]
561.2934
y3
462.2530
398.1909
266.1182
299.1627
370.2021
413.1882
167.0862
120.0786
198.1028
271.1651
348.1650
445.2169
463.2581
89.0619
b1
y1
314.1530
y2
484.2285
516.2743
m/z

4C': MW = 546.6, Purity = 77.8%, Yield = 6.5% [0.083 mg]

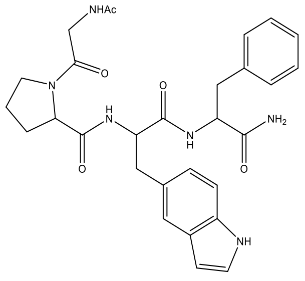

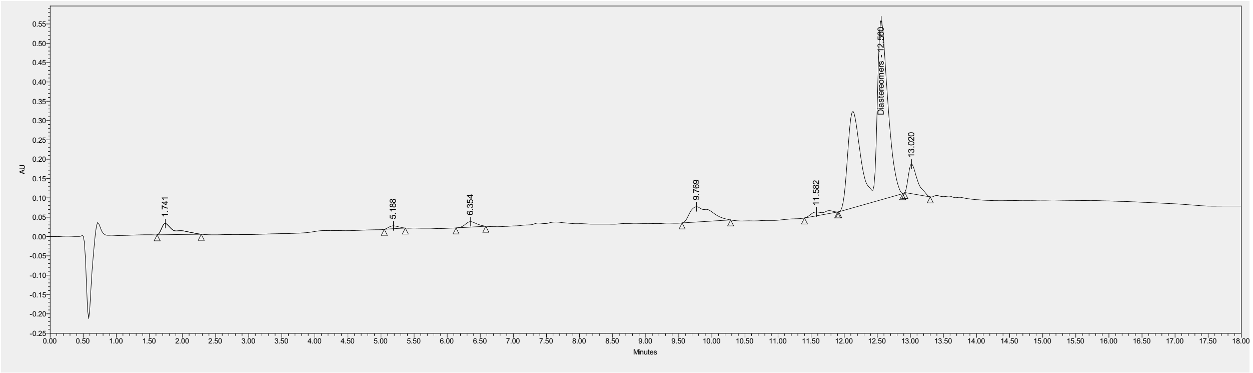

	Name	Retention Time	Area	% Area
1		1.741	415418	3.79
2		5.188	75320	0.69
3		6.354	167137	1.52
4		9.769	878128	8.00
5		11.582	170220	1.55
6	Diastereomers	12.560	8538789	77.83
7		13.020	726476	6.62

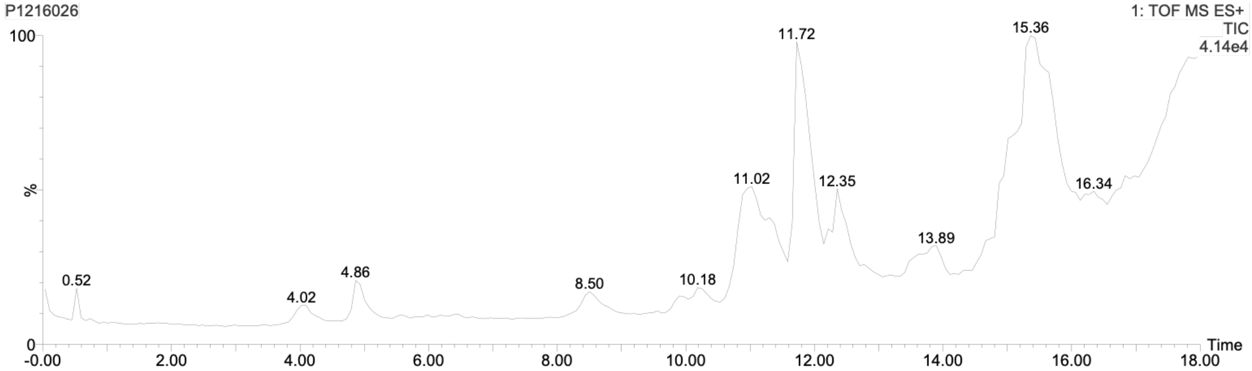

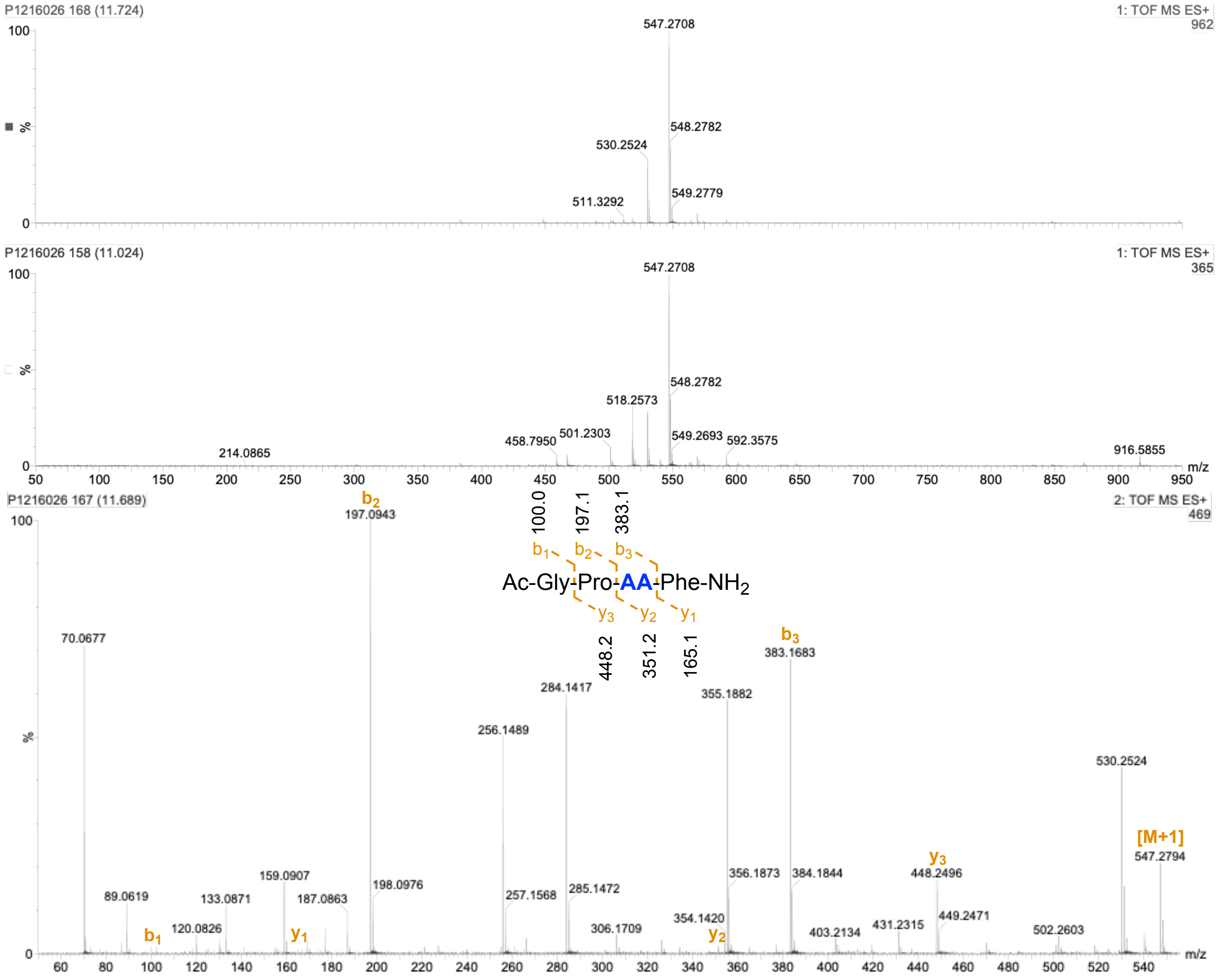
P1216026 168 (11.724)
1: TOF MS ES+
962
547.2708
548.2782
530.2524
511.3292
549.2779
P1216026 158 (11.024)
1: TOF MS ES+
365
547.2708
548.2782
518.2573
501.2303
458.7950
549.2693
592.3575
214.0865
916.5855
m/z
P1216026 167 (11.689)
2: TOF MS ES+
469
Ac-Gly-Pro-AA-Phe-NH2
100.0
197.1
383.1
448.2
351.2
165.1
b1
b2
b3
y1
y2
y3
197.0943
70.0677
383.1683
284.1417
355.1882
256.1489
530.2524
[M+1]
547.2794
448.2496
159.0907
356.1873
384.1844
198.0976
89.0619
133.0871
187.0863
285.1472
257.1568
449.2471
354.1420
120.0826
306.1709
431.2315
403.2134
502.2603
m/z

5C’: MW = 546.6, Purity = 54.2%, Yield = 9.6% [0.12 mg]

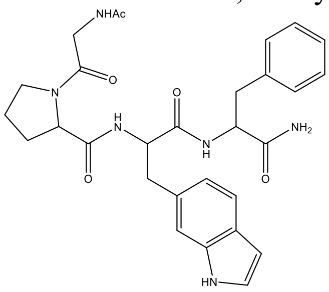

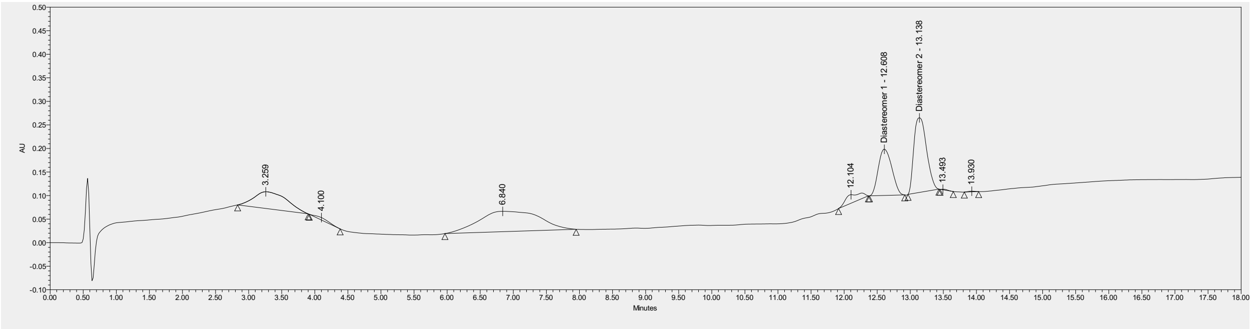

	Name	Retention Time	Area	% Area
1		3.129	689450	3.70
2		3.481	321067	1.72
3		6.231	7537015	40.41
4	Diastereomer 1	11.821	4641464	24.89
5	Diastereomer 2	12.522	5460276	29.28

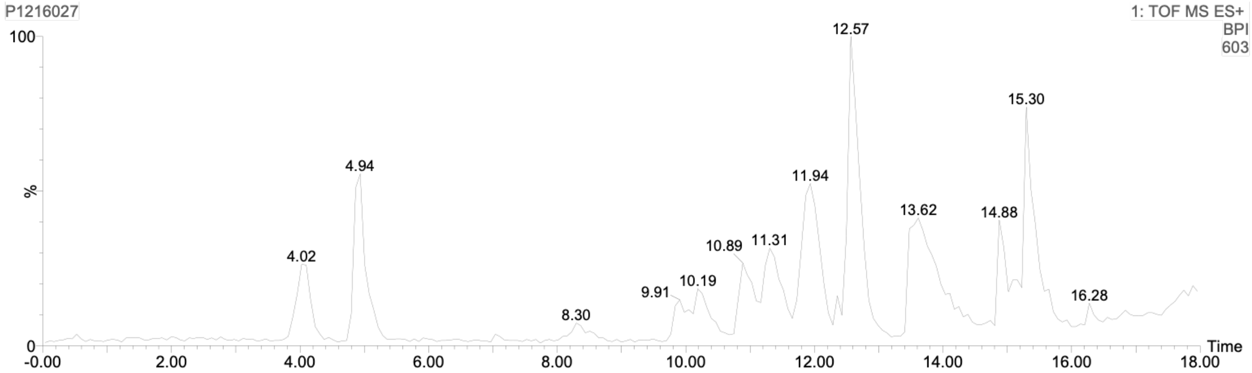

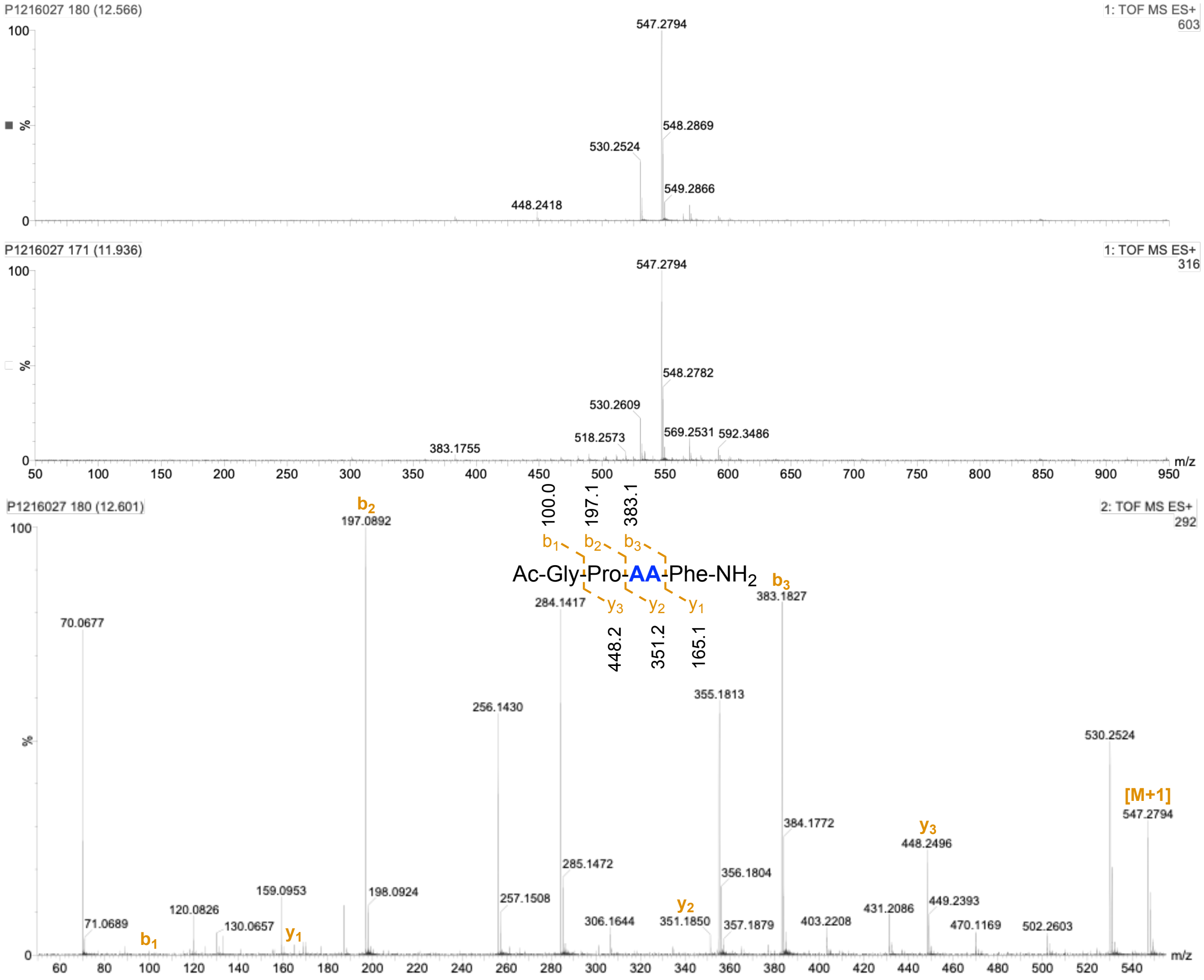
P1216027 180 (12.566)
1: TOF MS ES+
603
547.2794
548.2869
530.2524
549.2866
448.2418
P1216027 171 (11.936)
1: TOF MS ES+
316
547.2794
548.2782
530.2609
518.2573
569.2531
592.3486
383.1755
m/z
P1216027 180 (12.601)
2: TOF MS ES+
292
Ac-Gly-Pro-AA-Phe-NH2
b1 b2 b3
y3 y2 y1
100.0
197.1
383.1
448.2
351.2
165.1
b2
197.0892
70.0677
284.1417
b3
383.1827
256.1430
355.1813
530.2524
[M+1]
547.2794
384.1772
y3
448.2496
285.1472
356.1804
159.0953
198.0924
257.1508
y2
351.1850
449.2393
431.2086
120.0826
71.0689
130.0657
306.1644
357.1879
403.2208
470.1169
502.2603
b1
y1
m/z

6C’: MW = 547.6, Purity = 62.0%, Yield = 6.2% [0.080 mg]

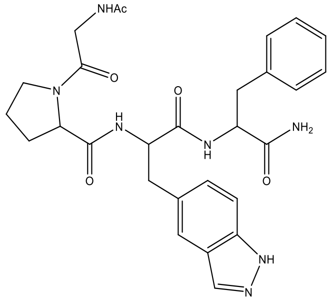

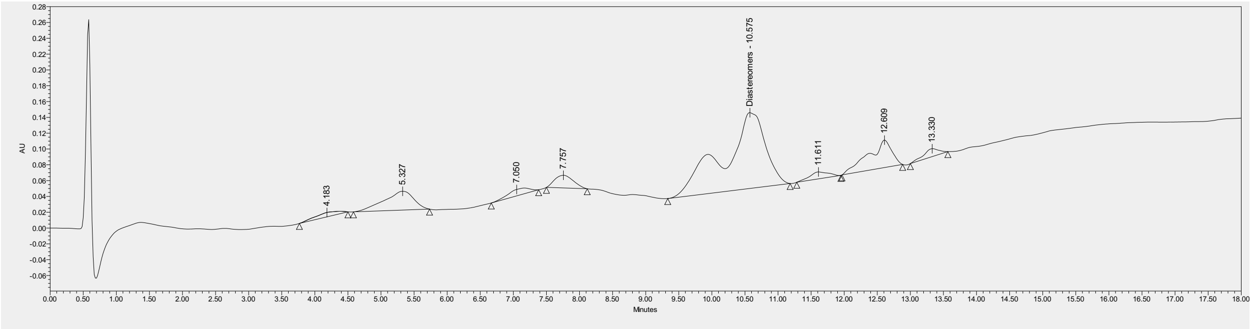

	Name	Retention Time	Area	% Area
1		4.183	142485	2.09
2		5.327	744279	10.93
3		7.050	179673	2.64
4		7.757	315844	4.64
5	Diastereomers	10.575	4224271	62.02
6		11.611	173919	2.55
7		12.609	865817	12.71
8		13.330	164924	2.42

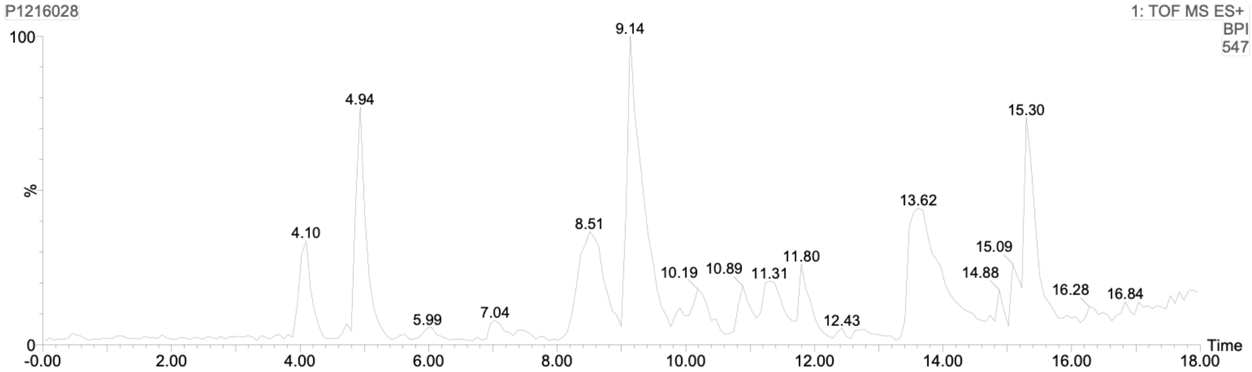

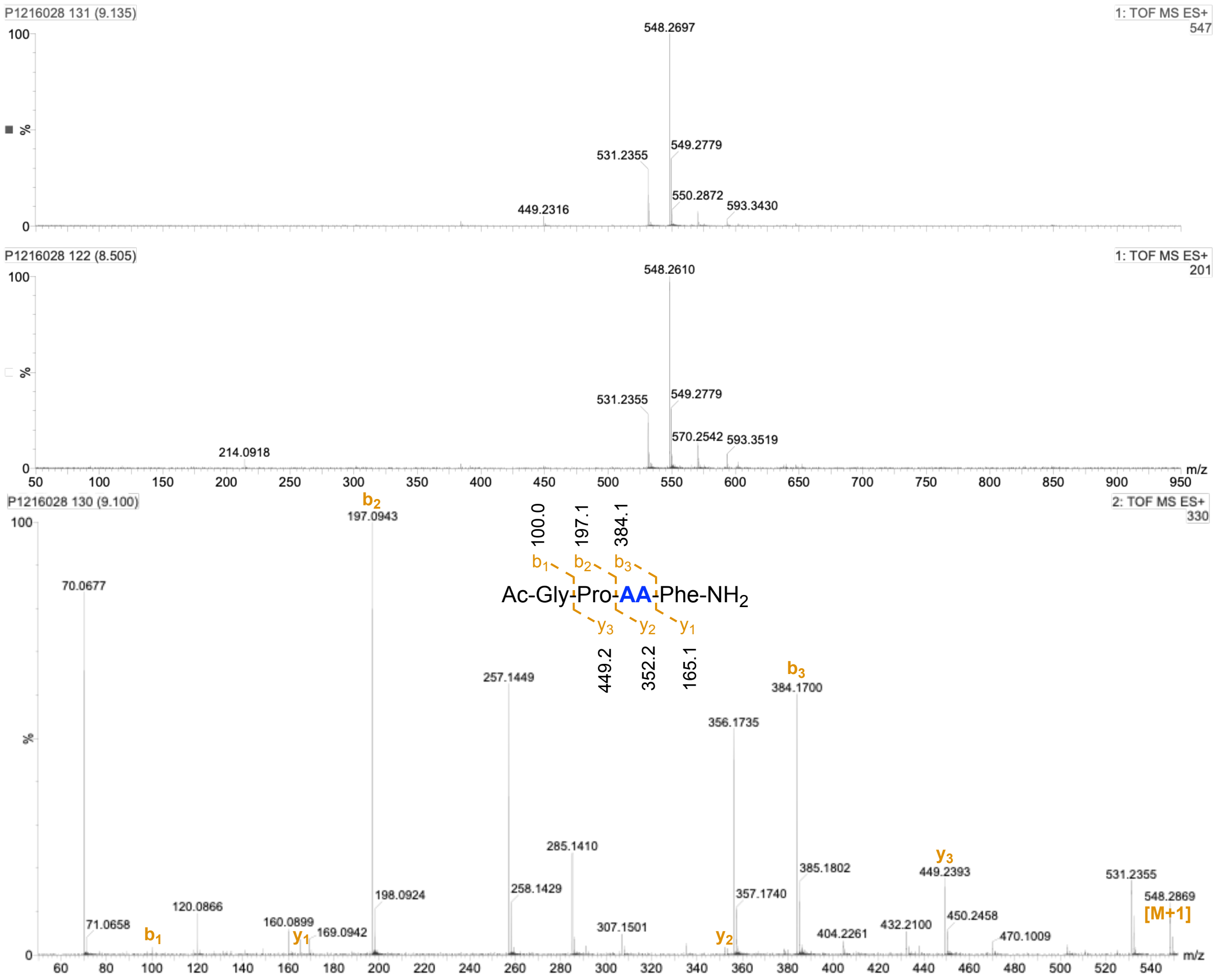
P1216028 131 (9.135)
1: TOF MS ES+
547
548.2697
531.2355
549.2779
550.2872
593.3430
449.2316
P1216028 122 (8.505)
1: TOF MS ES+
201
548.2610
531.2355
549.2779
570.2542
593.3519
214.0918
m/z
P1216028 130 (9.100)
2: TOF MS ES+
330
b2
197.0943
100.0
197.1
384.1
Ac-Gly-Pro-AA-Phe-NH2
449.2
352.2
165.1
70.0677
257.1449
b3
384.1700
356.1735
285.1410
385.1802
y3
449.2393
531.2355
548.2869
[M+1]
198.0924
258.1429
357.1740
120.0866
71.0658
160.0899
169.0942
307.1501
404.2261
432.2100
450.2458
470.1009
b1
y1
y2
m/z

7C': MW = 547.6, Purity = 77.9%, Yield = 12.7% [0.16 mg]

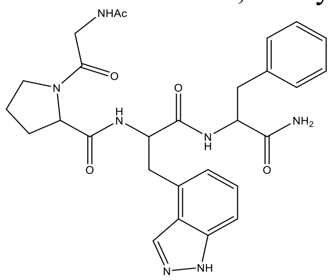

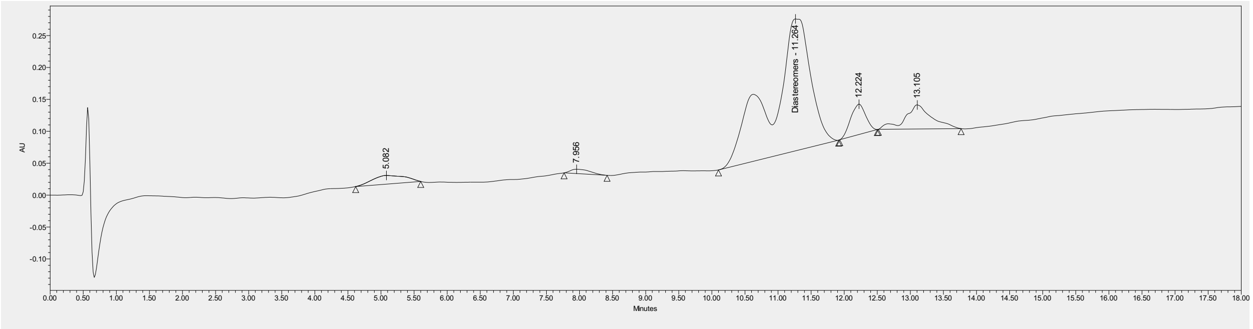

	Name	Retention Time	Area	% Area
1		5.082	466551	4.23
2		7.956	143479	1.30
3	Diastereomers	11.264	8597511	77.92
4		12.224	755364	6.85
5		13.105	1070331	9.70

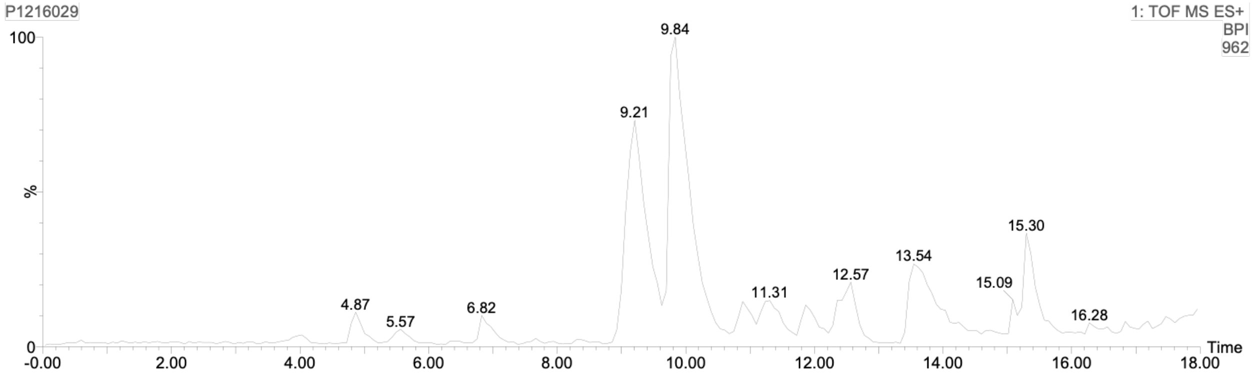

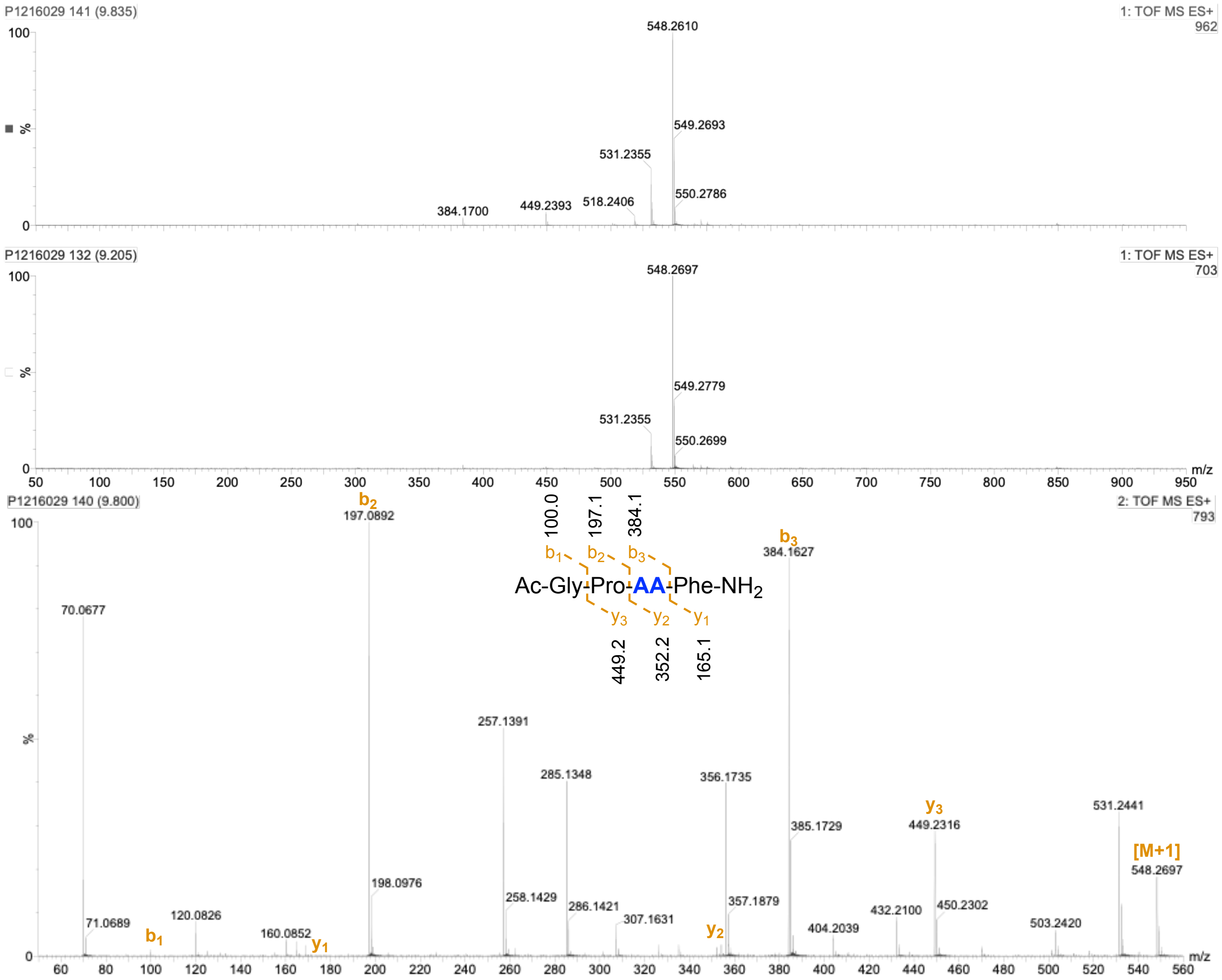
P1216029 141 (9.835)
1: TOF MS ES+
962
548.2610
549.2693
531.2355
550.2786
384.1700
449.2393
518.2406
P1216029 132 (9.205)
1: TOF MS ES+
703
548.2697
549.2779
531.2355
550.2699
m/z
P1216029 140 (9.800)
2: TOF MS ES+
793
Ac-Gly-Pro-AA-Phe-NH2
b1 b2 b3
100.0 197.1 384.1
y3 y2 y1
449.2 352.2 165.1
b2 197.0892
b3 384.1627
70.0677
257.1391
285.1348
356.1735
385.1729
y3 449.2316
531.2441
[M+1] 548.2697
198.0976
258.1429
286.1421
307.1631
357.1879
432.2100
450.2302
404.2039
503.2420
71.0689
120.0826
160.0852
b1
y1
y2
%
m/z

8C': MW = 561.6, Purity = 69.2%, Yield = 33.1% [0.43 mg]

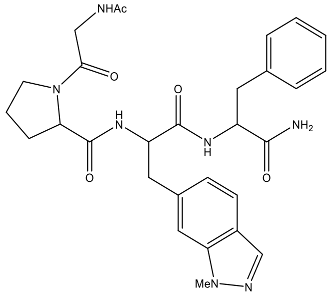

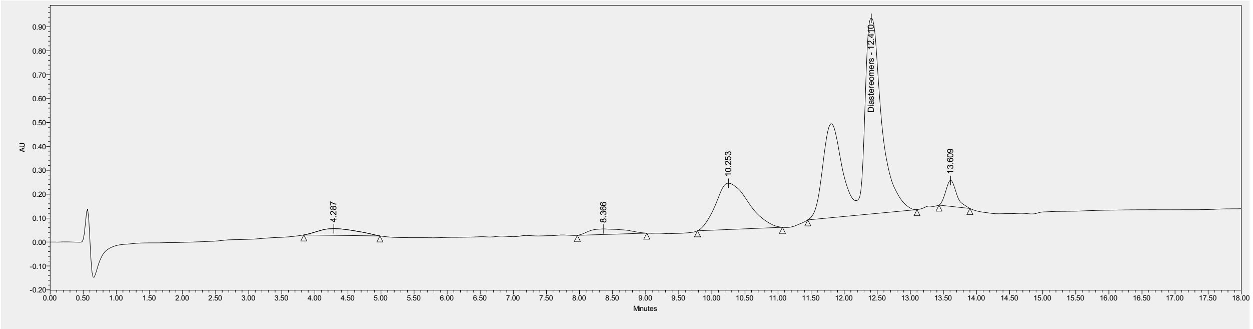

	Name	Retention Time	Area	% Area
1		4.287	1154892	3.56
2		8.366	887289	2.74
3		10.253	6703786	20.69
4	Diastereomers	12.410	22416973	69.19
5		13.609	1237419	3.82

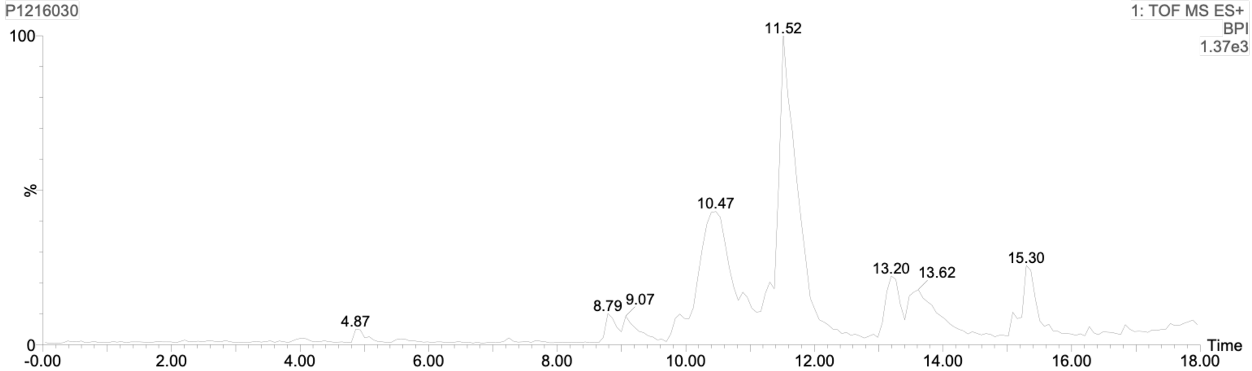

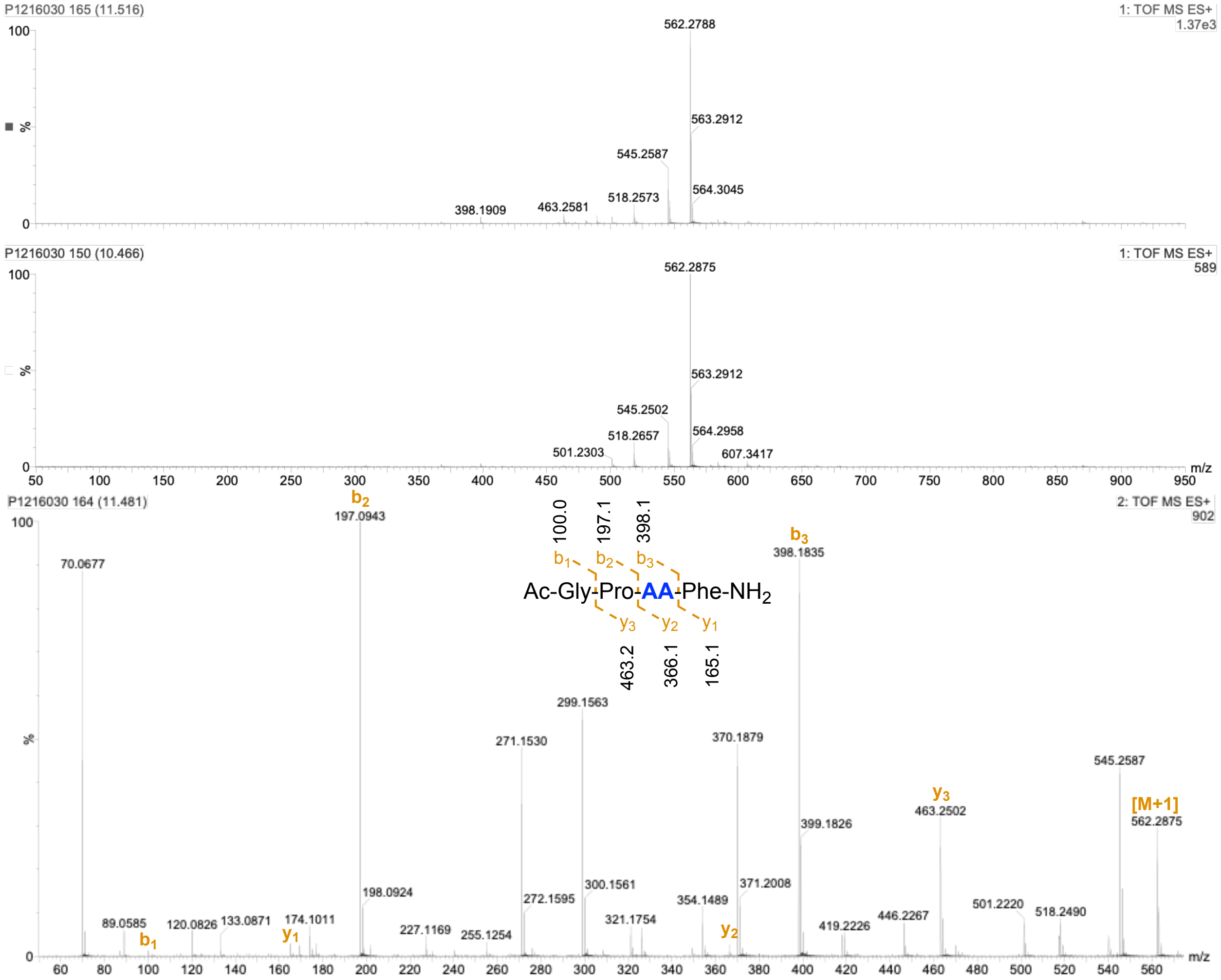

P1216030 165 (11.516)
1: TOF MS ES+
1.37e3
562.2788
563.2912
545.2587
564.3045
518.2573
463.2581
398.1909
P1216030 150 (10.466)
1: TOF MS ES+
589
562.2875
563.2912
545.2502
518.2657
564.2958
501.2303
607.3417
m/z
P1216030 164 (11.481)
2: TOF MS ES+
902
b2
197.0943
70.0677
Ac-Gly-Pro-AA-Phe-NH2
b1 b2 b3
100.0 197.1 398.1
y3 y2 y1
463.2 366.1 165.1
b3
398.1835
299.1563
271.1530
370.1879
545.2587
y3
463.2502
[M+1]
562.2875
399.1826
198.0924
272.1595
300.1561
371.2008
354.1489
321.1754
446.2267
501.2220
518.2490
419.2226
89.0585
120.0826
133.0871
174.1011
227.1169
255.1254
b1
y1
y2
m/z

9C': MW = 561.6, Purity = 84.6%, Yield = 23.8% [0.31 mg]

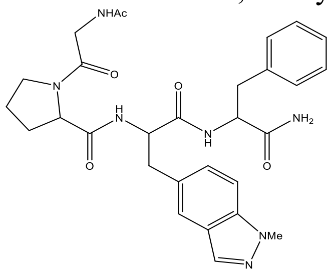

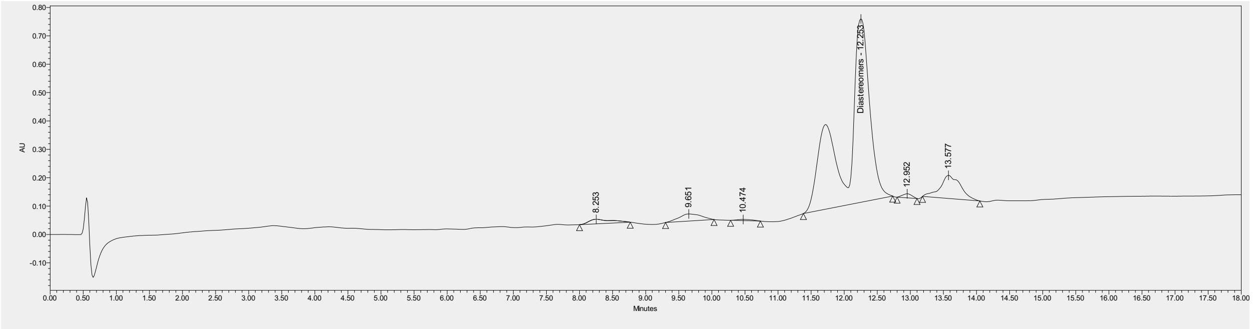

	Name	Retention Time	Area	% Area
1		8.253	377277	1.98
2		9.651	587998	3.09
3		10.474	69300	0.36
4	Diastereomers	12.253	16085566	84.60
5		12.952	141046	0.74
6		13.577	1752051	9.21

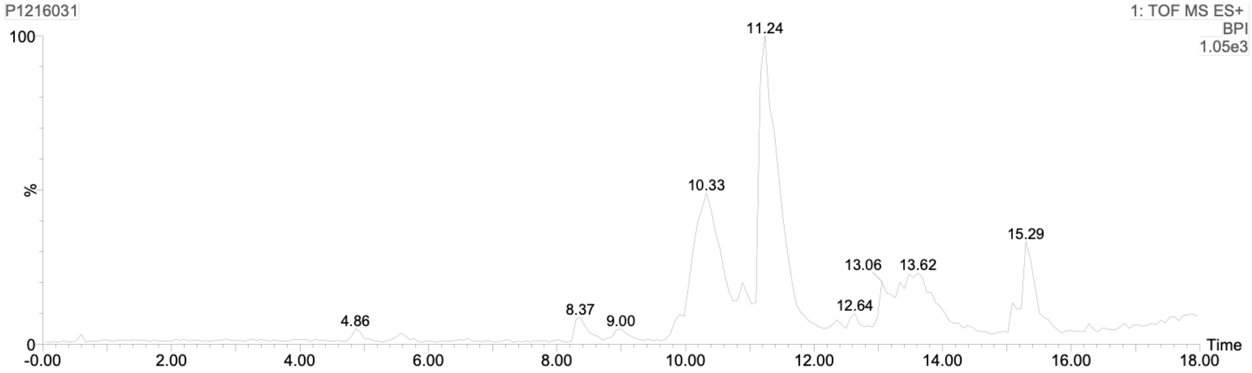

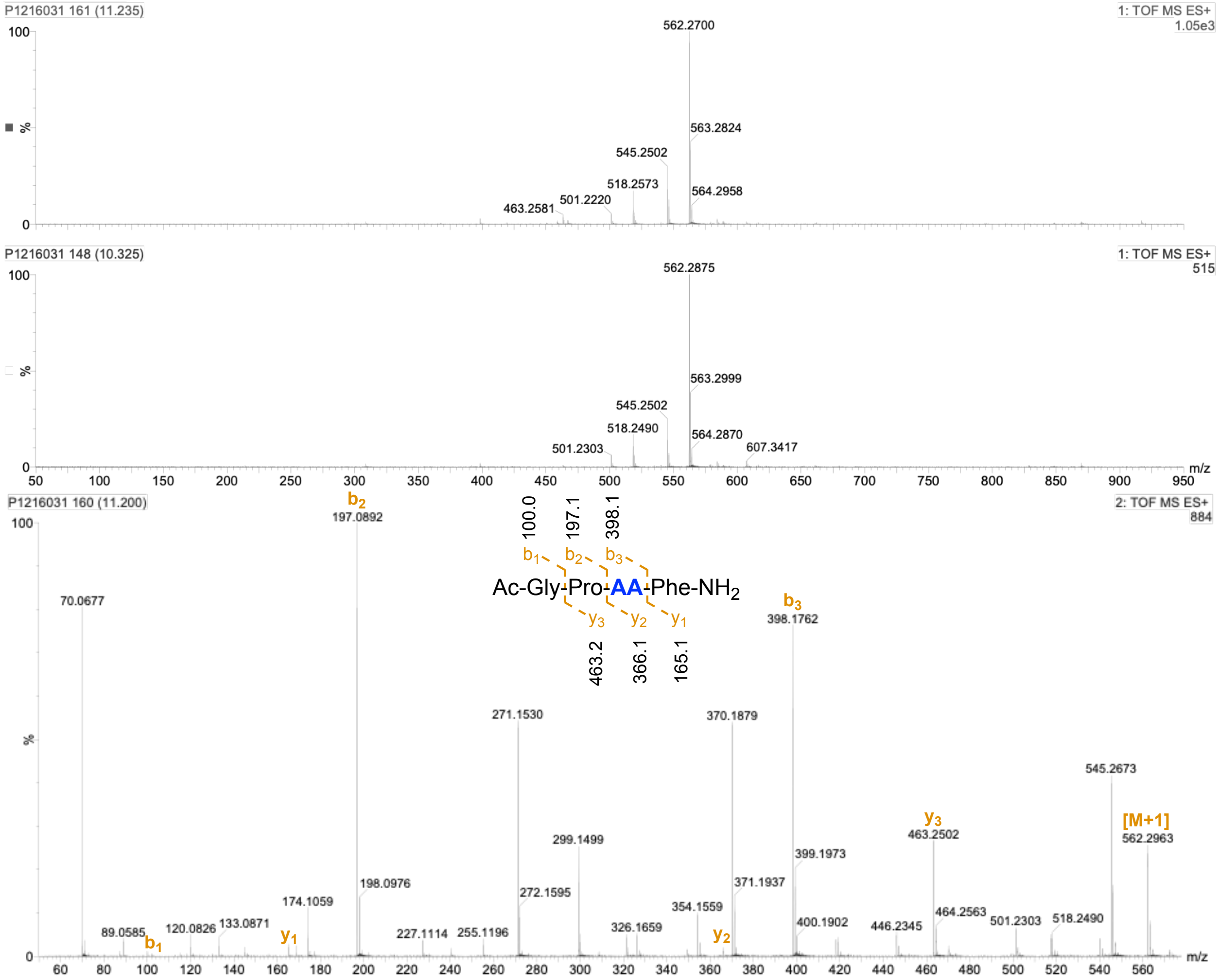
P1216031 161 (11.235)
1: TOF MS ES+
1.05e3
562.2700
563.2824
545.2502
518.2573
564.2958
463.2581
501.2220
P1216031 148 (10.325)
1: TOF MS ES+
515
562.2875
563.2999
545.2502
518.2490
564.2870
607.3417
501.2303
m/z
P1216031 160 (11.200)
2: TOF MS ES+
884
b2
197.0892
Ac-Gly-Pro-AA-Phe-NH2
100.0
197.1
398.1
463.2
366.1
165.1
70.0677
b3
398.1762
271.1530
370.1879
545.2673
y3
463.2502
[M+1]
562.2963
299.1499
399.1973
198.0976
272.1595
371.1937
174.1059
354.1559
464.2563
89.0585
120.0826
133.0871
227.1114
255.1196
326.1659
400.1902
446.2345
501.2303
518.2490
b1
y1
y2
m/z

10C': MW = 561.6, Purity = 74.9%, Yield = 14.2% [0.19 mg]

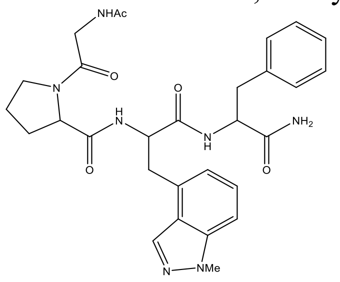

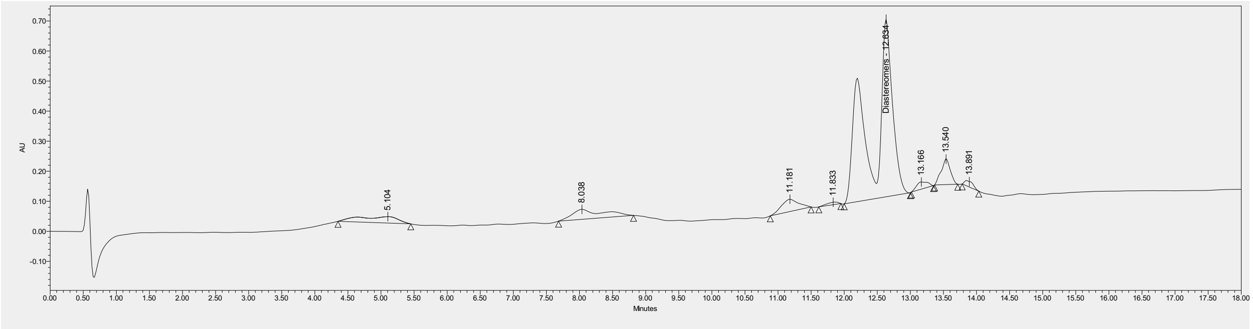

	Name	Retention Time	Area	% Area
1		5.104	861030	5.37
2		8.038	1060676	6.61
3		11.181	741179	4.62
4		11.833	100574	0.63
5	Diastereomers	12.634	12009543	74.87
6		13.166	282876	1.76
7		13.540	815690	5.09
8		13.891	168922	1.05

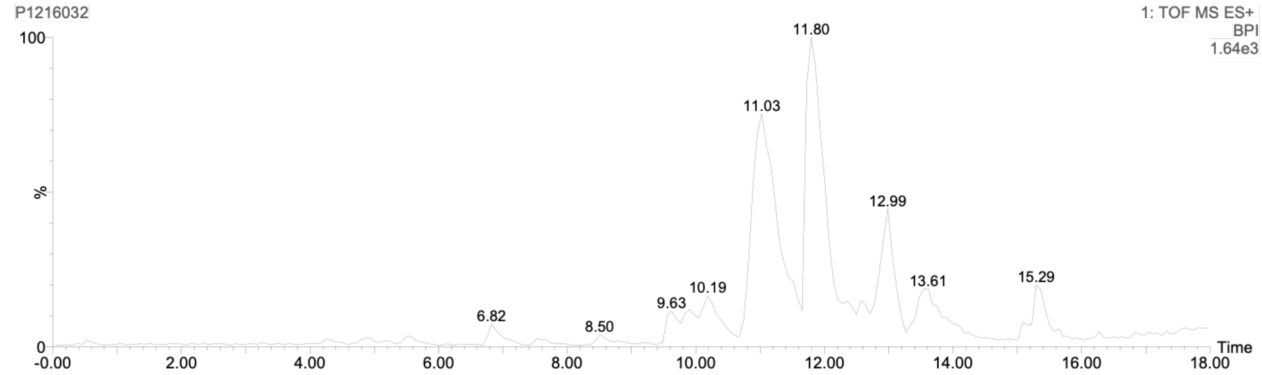

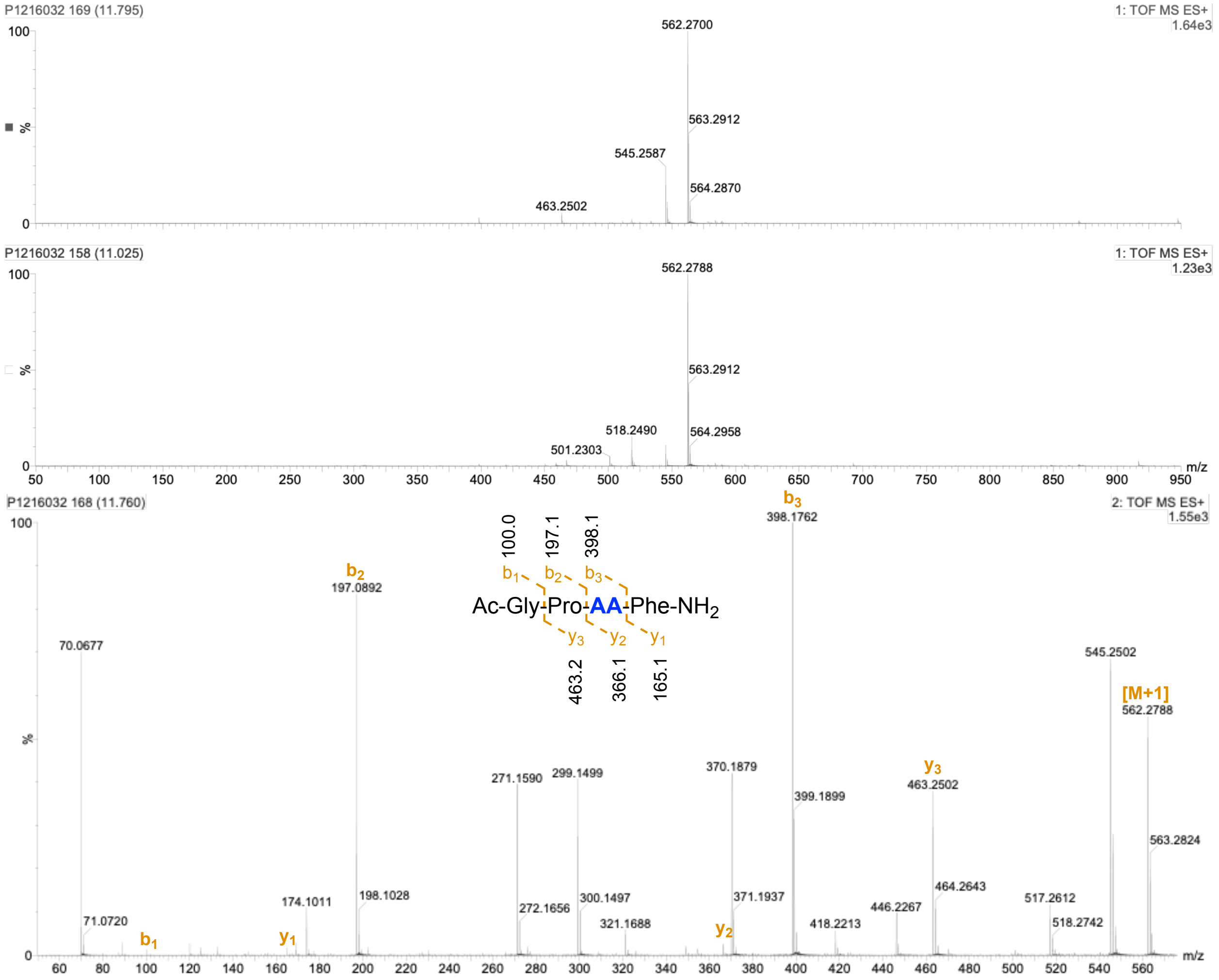
P1216032 169 (11.795)
1: TOF MS ES+
1.64e3
562.2700
563.2912
545.2587
564.2870
463.2502
P1216032 158 (11.025)
1: TOF MS ES+
1.23e3
562.2788
563.2912
518.2490
564.2958
501.2303
m/z
P1216032 168 (11.760)
2: TOF MS ES+
1.55e3
b3
398.1762
b2
197.0892
Ac-Gly-Pro-AA-Phe-NH2
100.0
197.1
398.1
463.2
366.1
165.1
70.0677
545.2502
[M+1]
562.2788
271.1590
299.1499
370.1879
y3
463.2502
399.1899
563.2824
174.1011
198.1028
272.1656
300.1497
371.1937
446.2267
464.2643
517.2612
518.2742
71.0720
321.1688
418.2213
m/z

11C': MW = 561.6, Purity = 88.2%, Yield = 17.5% [0.23 mg]

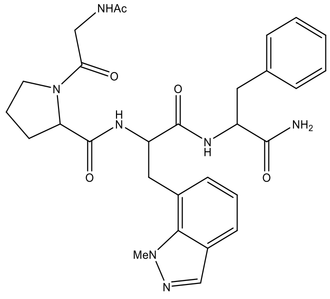

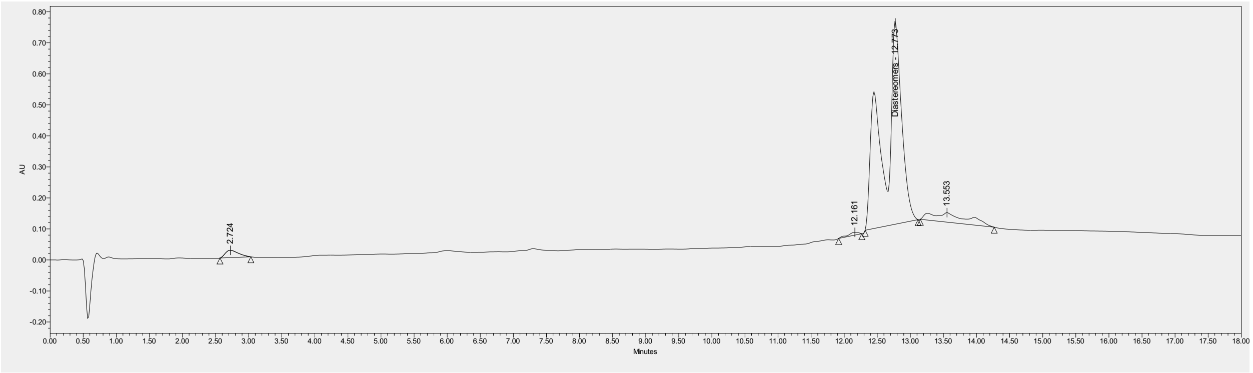

	Name	Retention Time	Area	% Area
1		2.724	327793	2.43
2		12.161	105623	0.78
3	Diastereomers	12.773	11878770	88.20
4		13.553	1156521	8.59

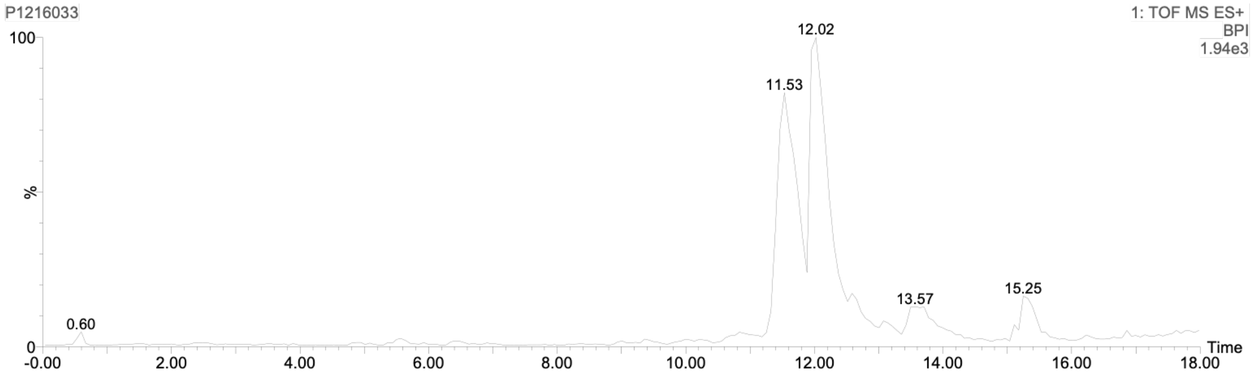

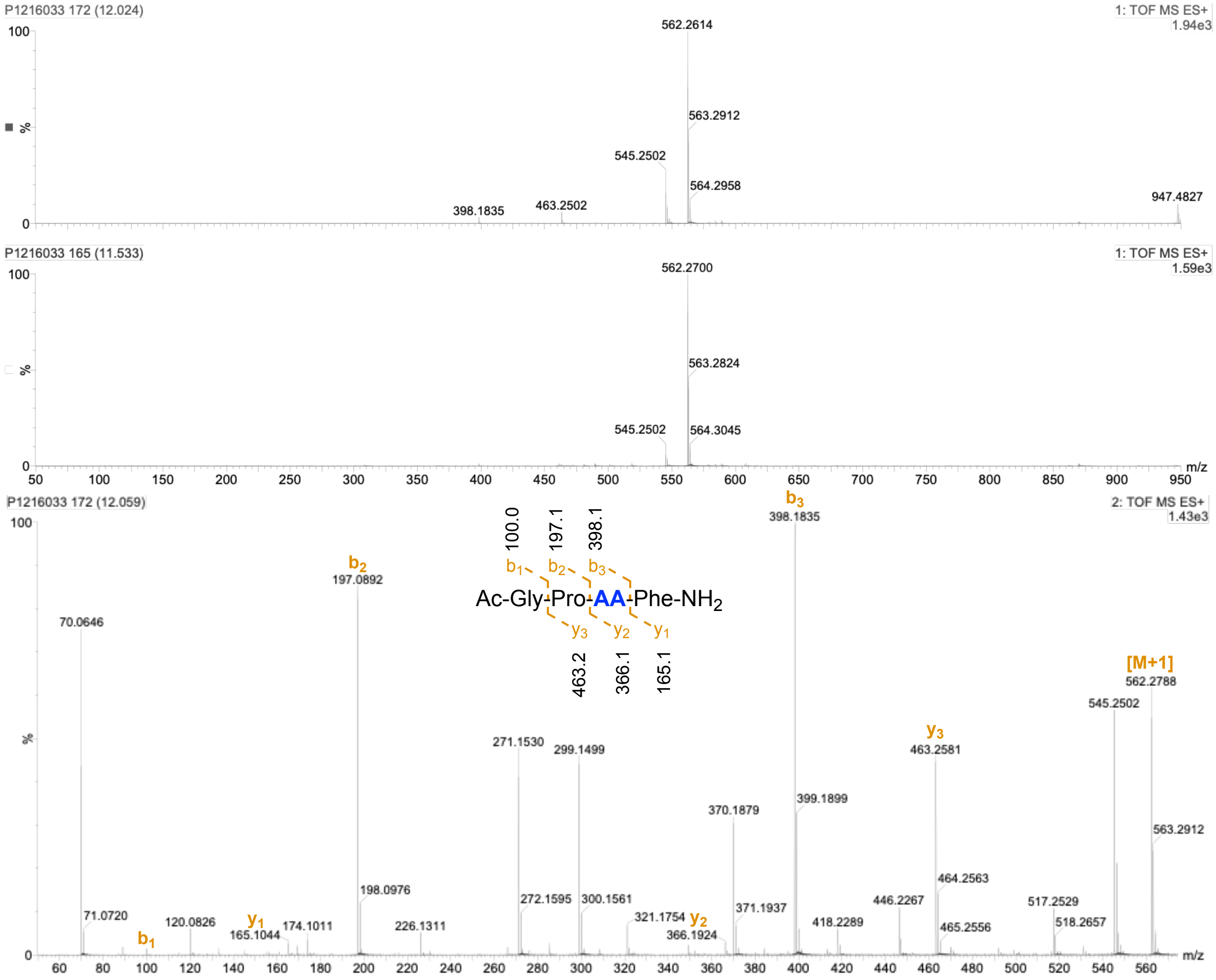
P1216033 172 (12.024)
1: TOF MS ES+
1.94e3
562.2614
563.2912
545.2502
564.2958
463.2502
398.1835
947.4827
P1216033 165 (11.533)
1: TOF MS ES+
1.59e3
562.2700
563.2824
545.2502
564.3045
m/z
P1216033 172 (12.059)
2: TOF MS ES+
1.43e3
Ac-Gly-Pro-AA-Phe-NH2
b1 b2 b3
100.0 197.1 398.1
y3 y2 y1
463.2 366.1 165.1
b3
398.1835
b2
197.0892
70.0646
[M+1]
562.2788
545.2502
y3
463.2581
271.1530
299.1499
370.1879
399.1899
563.2912
464.2563
198.0976
272.1595
300.1561
446.2267
517.2529
71.0720
120.0826
y1
165.1044
174.1011
226.1311
321.1754
y2
366.1924
371.1937
418.2289
465.2556
518.2657
b1
m/z

12C': MW = 548.6, Purity = 57.3%, Yield = 3.0% [0.038 mg]

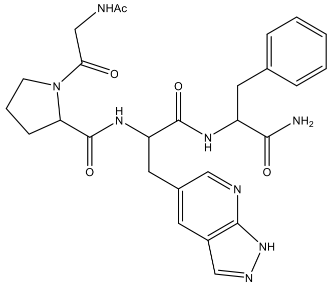

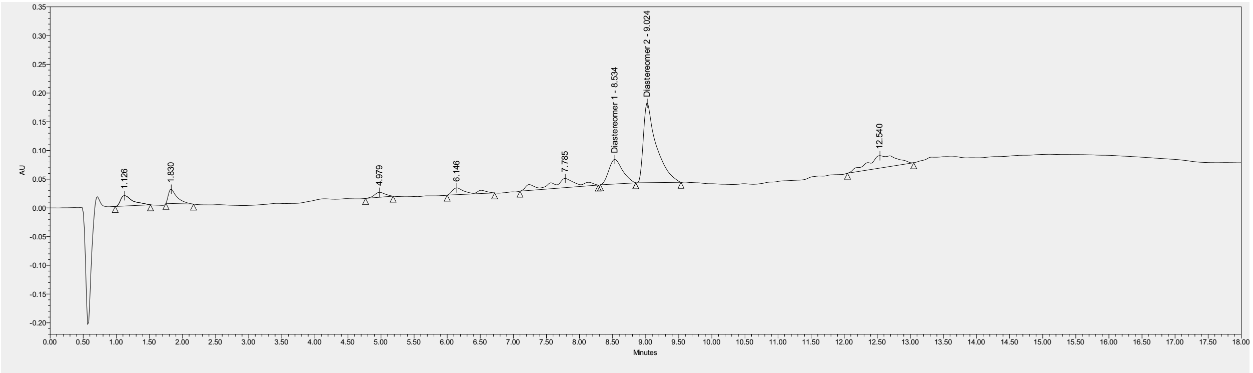

	Name	Retention Time	Area	% Area
1		1.126	235936	5.41
2		1.830	225089	5.16
3		4.979	96237	2.21
4		6.146	194459	4.46
5		7.785	473400	10.85
6	Diastereomer 1	8.534	618425	14.17
7	Diastereomer 2	9.024	1879938	43.08
8		12.540	640590	14.68

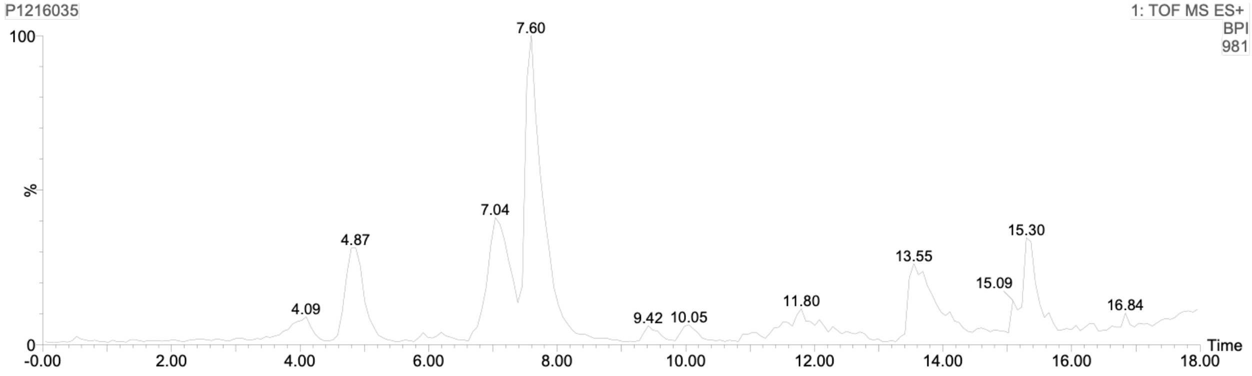

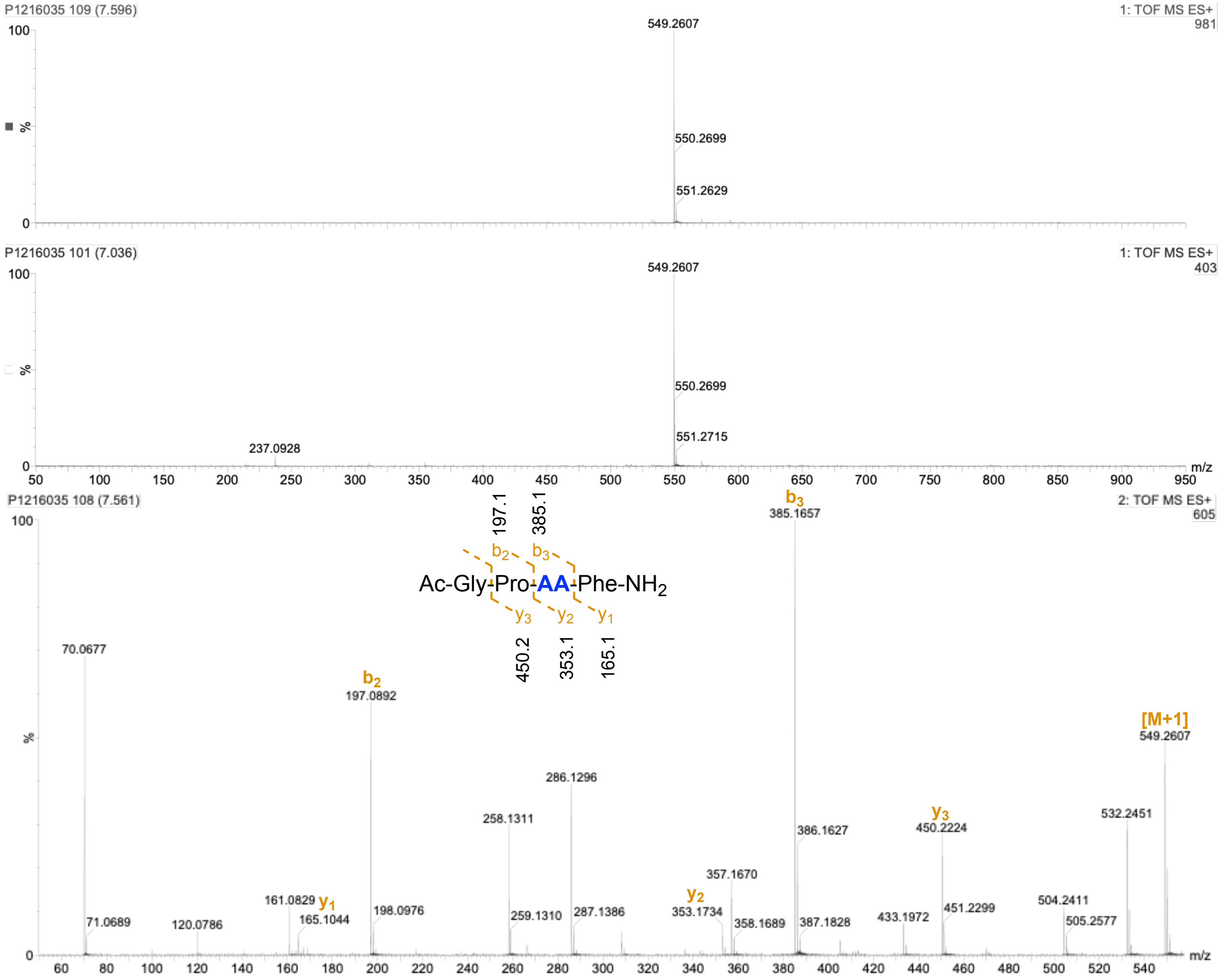

P1216035 109 (7.596)
1: TOF MS ES+
981
549.2607
550.2699
551.2629
P1216035 101 (7.036)
1: TOF MS ES+
403
549.2607
550.2699
551.2715
237.0928
m/z
P1216035 108 (7.561)
2: TOF MS ES+
605
Ac-Gly-Pro-AA-Phe-NH2
197.1
385.1
450.2
353.1
165.1
b3
385.1657
70.0677
b2
197.0892
[M+1]
549.2607
286.1296
258.1311
532.2451
386.1627
y3
450.2224
357.1670
y2
353.1734
161.0829
y1
165.1044
71.0689
120.0786
198.0976
259.1310
287.1386
358.1689
387.1828
433.1972
451.2299
504.2411
505.2577
m/z
%

1D’: MW = 548.6, Purity = 11.5%, Yield = 0.23% [0.003 mg]

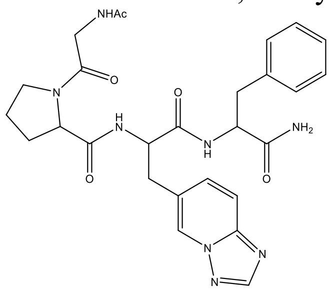

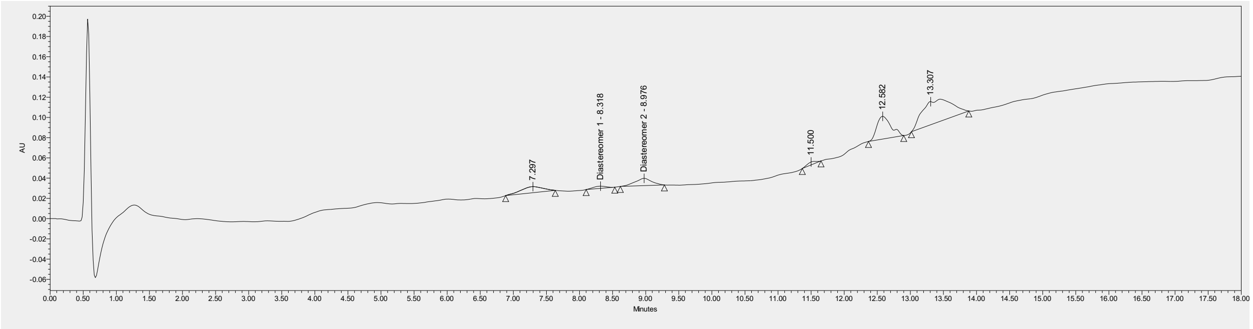

	Name	Retention Time	Area	% Area
1		7.297	139899	10.29
2	Diastereomer 1	8.318	29730	2.19
3	Diastereomer 2	8.976	126595	9.31
4		11.500	26953	1.98
5		12.582	331263	24.36
6		13.307	705304	51.87

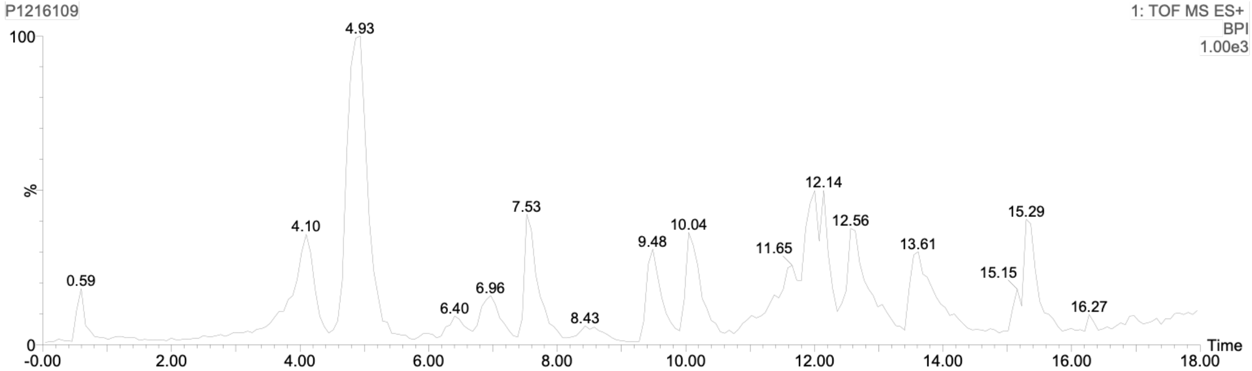

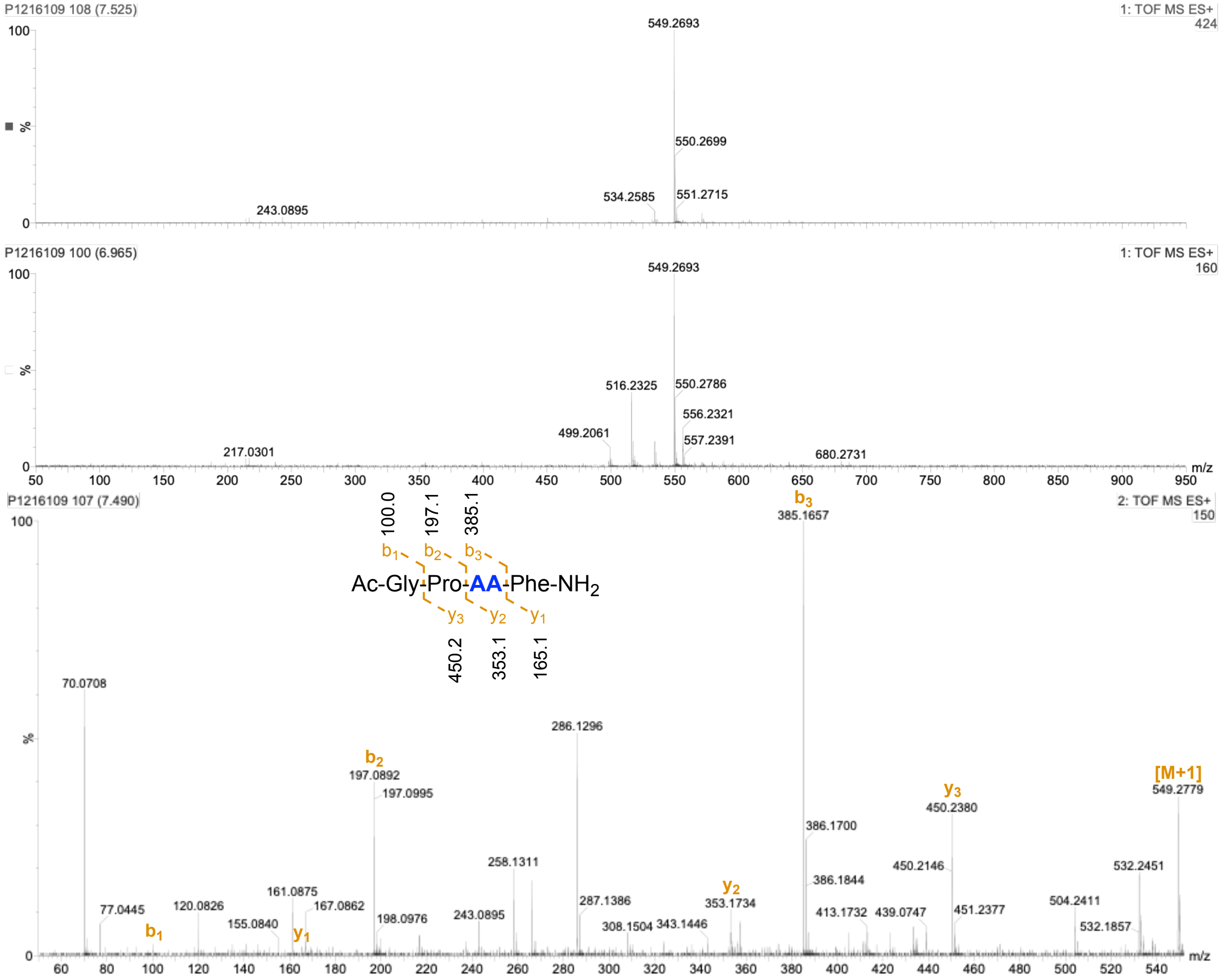
P1216109 108 (7.525)
1: TOF MS ES+
424
549.2693
550.2699
534.2585
551.2715
243.0895
P1216109 100 (6.965)
1: TOF MS ES+
160
549.2693
516.2325
550.2786
556.2321
499.2061
557.2391
217.0301
680.2731
m/z
P1216109 107 (7.490)
2: TOF MS ES+
150
100.0
197.1
385.1
b1
b2
b3
Ac-Gly-Pro-AA-Phe-NH2
y3
y2
y1
450.2
353.1
165.1
b3
385.1657
70.0708
286.1296
b2
197.0892
197.0995
[M+1]
549.2779
y3
450.2380
386.1700
258.1311
450.2146
532.2451
386.1844
y2
353.1734
77.0445
120.0826
161.0875
167.0862
155.0840
198.0976
243.0895
287.1386
308.1504
343.1446
413.1732
439.0747
451.2377
504.2411
532.1857
b1
y1
m/z

2D': MW = 561.6, Purity = 66.5%, Yield = 3.0% [0.039 mg]

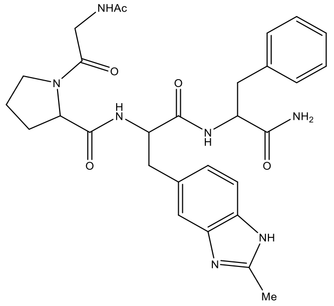

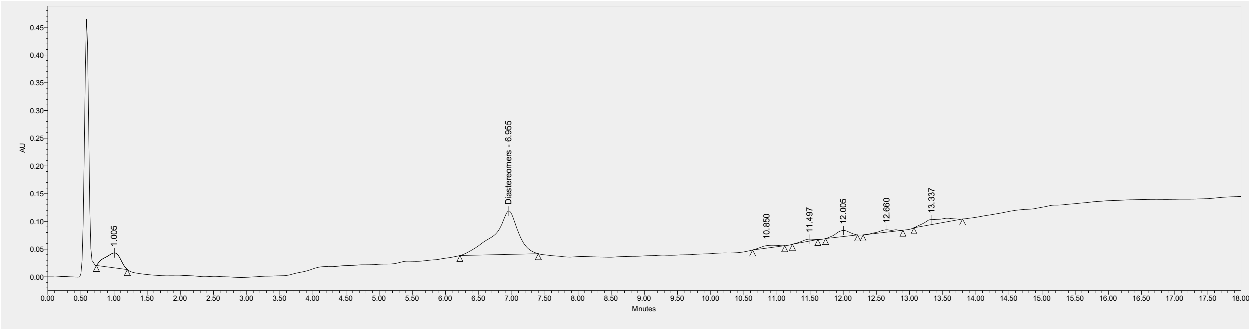

	Name	Retention Time	Area	% Area
1		1.005	437775	14.58
2	Diastereomers	6.955	1997507	66.54
3		10.850	72370	2.41
4		11.497	42924	1.43
5		12.005	161425	5.38
6		12.660	67307	2.24
7		13.337	222700	7.42

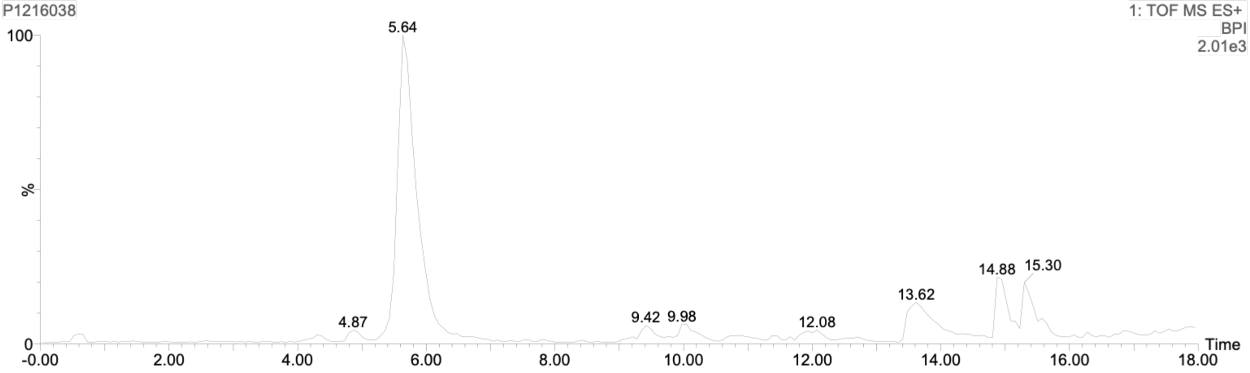

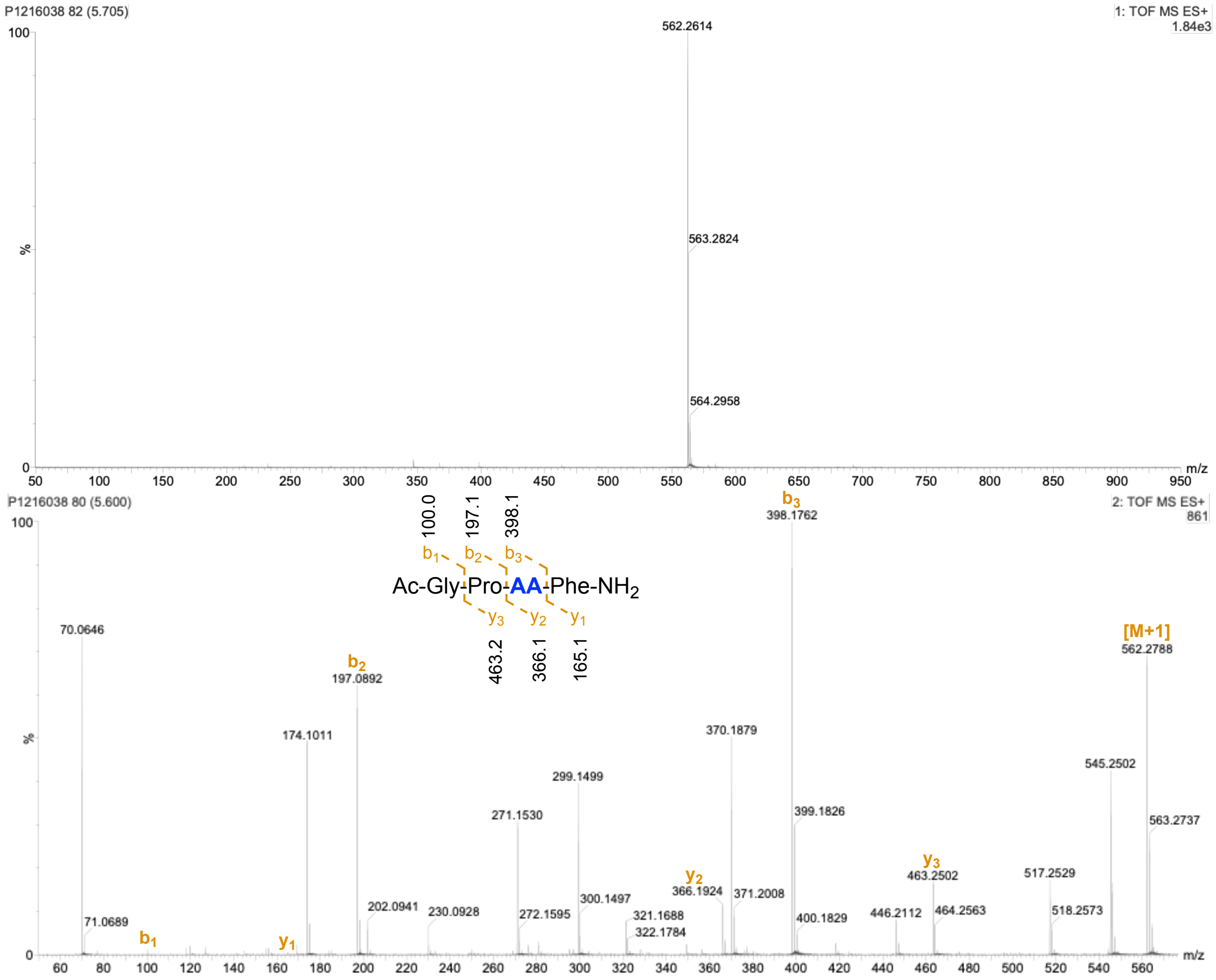
P1216038 82 (5.705)
1: TOF MS ES+
1.84e3
562.2614
563.2824
564.2958
P1216038 80 (5.600)
2: TOF MS ES+
861
Ac-Gly-Pro-AA-Phe-NH2
100.0
197.1
398.1
463.2
366.1
165.1
b3 398.1762
[M+1] 562.2788
70.0646
b2 197.0892
174.1011
370.1879
299.1499
545.2502
271.1530
399.1826
563.2737
y2 366.1924
y3 463.2502
517.2529
371.2008
300.1497
202.0941
230.0928
272.1595
321.1688
322.1784
400.1829
446.2112
464.2563
518.2573
71.0689
b1
y1
m/z
%

3D': MW = 564.7, Purity = 55.7%, Yield = 5.0% [0.066 mg]

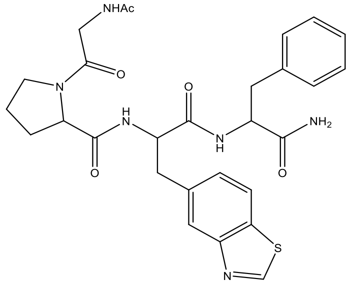

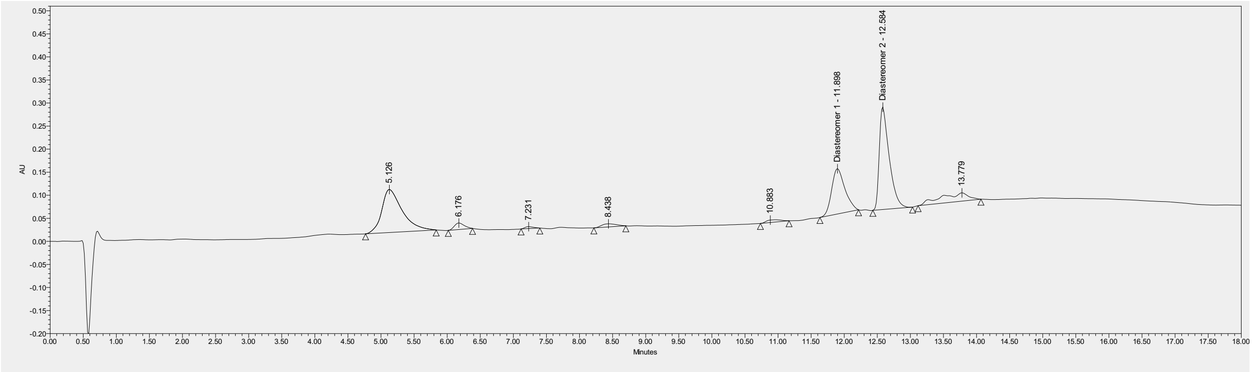

	Name	Retention Time	Area	% Area
1		5.126	2011698	30.35
2		6.176	147197	2.22
3		7.231	37430	0.56
4		8.438	104811	1.58
5		10.883	78565	1.19
6	Diastereomer 1	11.898	1369881	20.67
7	Diastereomer 2	12.584	2319147	34.99
8		13.779	559313	8.44

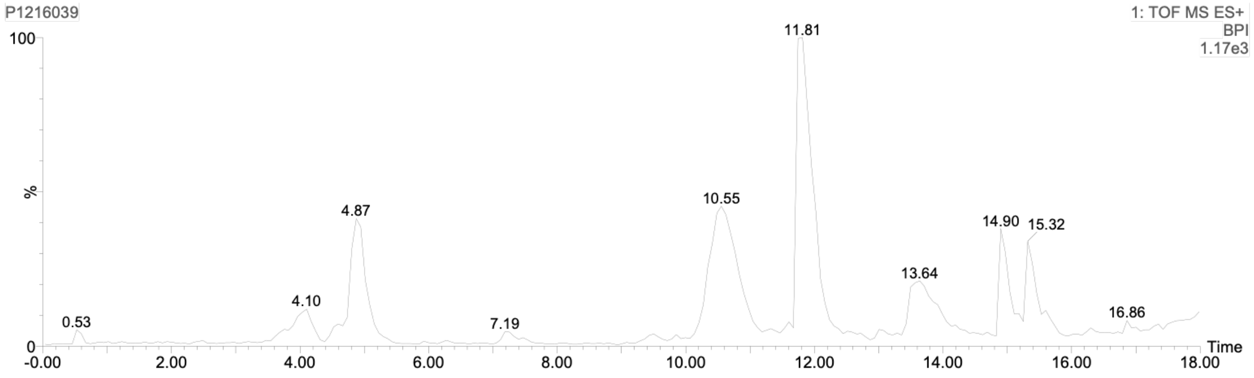

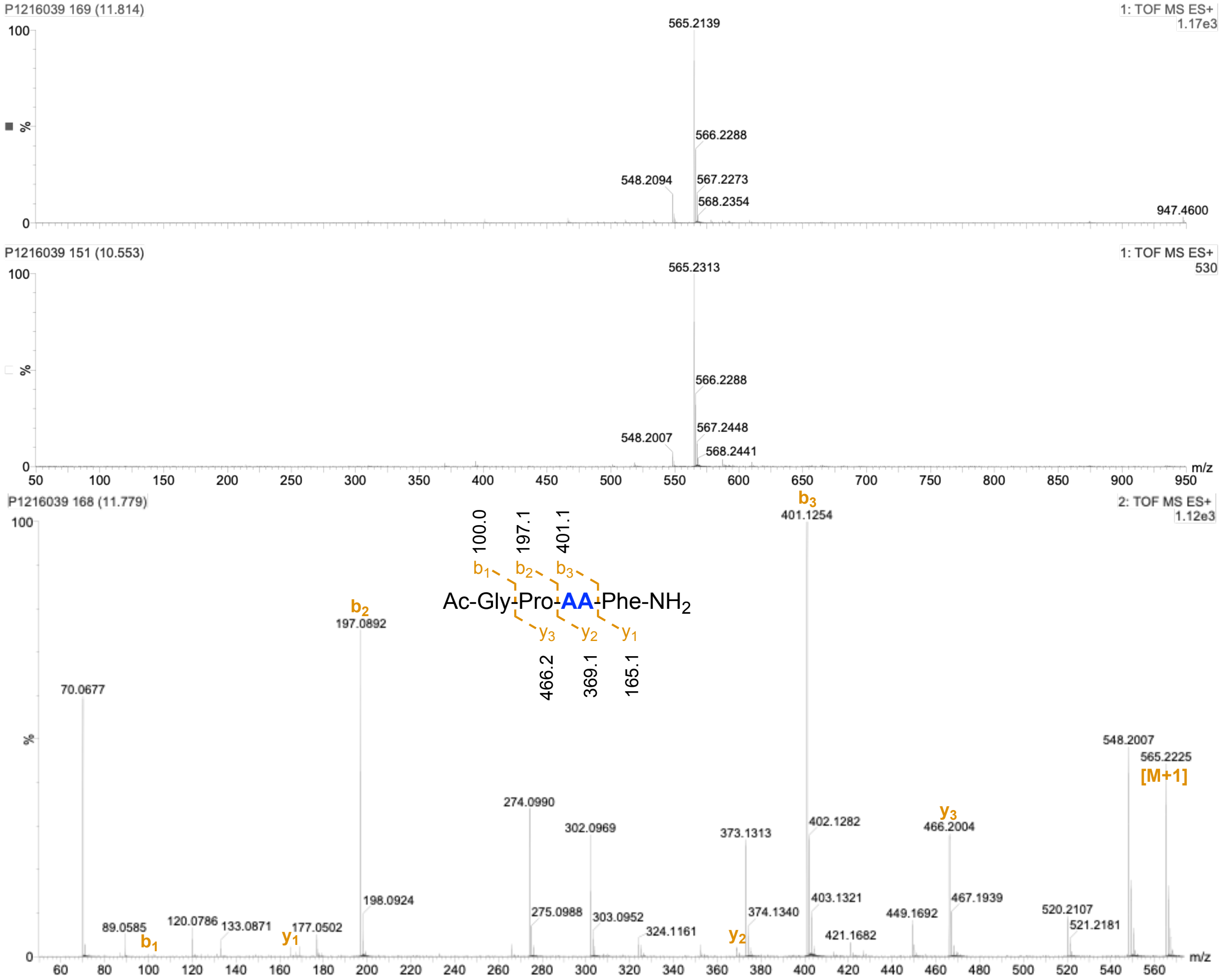
P1216039 169 (11.814)
1: TOF MS ES+
1.17e3
565.2139
566.2288
548.2094
567.2273
568.2354
947.4600
P1216039 151 (10.553)
1: TOF MS ES+
530
565.2313
566.2288
548.2007
567.2448
568.2441
m/z
P1216039 168 (11.779)
2: TOF MS ES+
1.12e3
Ac-Gly-Pro-AA-Phe-NH2
b1 b2 b3
100.0 197.1 401.1
y3 y2 y1
466.2 369.1 165.1
b3
401.1254
b2
197.0892
70.0677
548.2007
565.2225
[M+1]
274.0990
302.0969
373.1313
402.1282
y3
466.2004
198.0924
89.0585
b1
120.0786
133.0871
y1
177.0502
275.0988
303.0952
324.1161
y2
374.1340
403.1321
421.1682
449.1692
467.1939
520.2107
521.2181
m/z

4D’: MW = 563.7, Purity = 17.1%, Yield = 0.73% [0.010 mg]

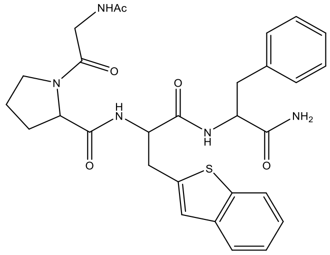

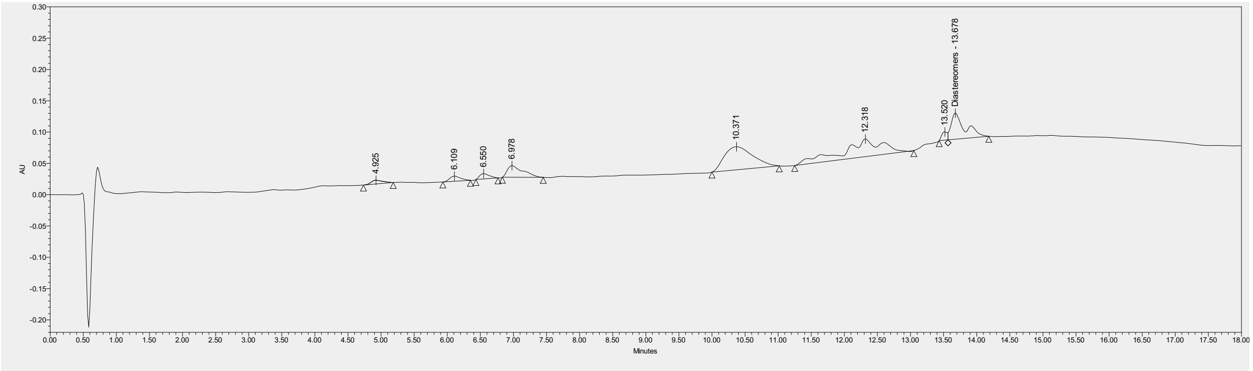

	Name	Retention Time	Area	% Area
1		4.925	72683	2.07
2		6.109	94316	2.68
3		6.550	87350	2.48
4		6.978	308128	8.76
5		10.371	1108002	31.51
6		12.318	1167326	33.20
7		13.520	77965	2.22
8	Diastereomers	13.678	600755	17.08

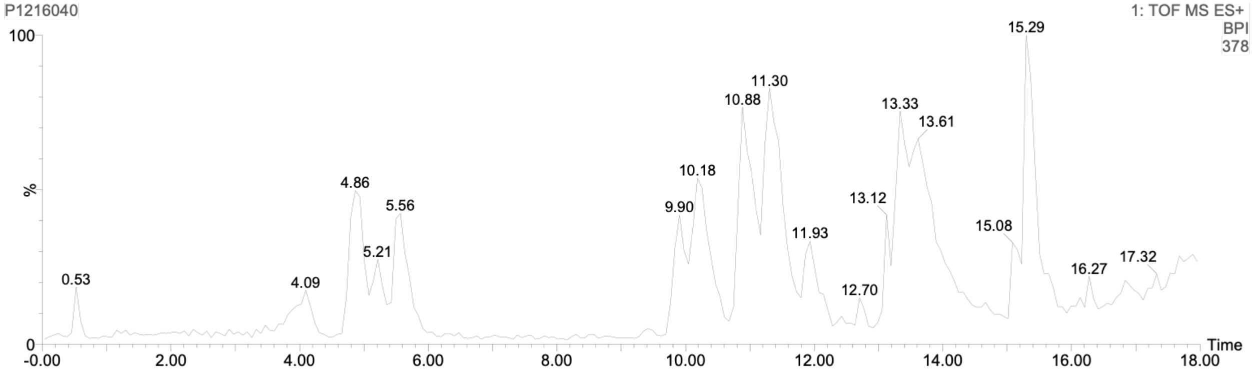

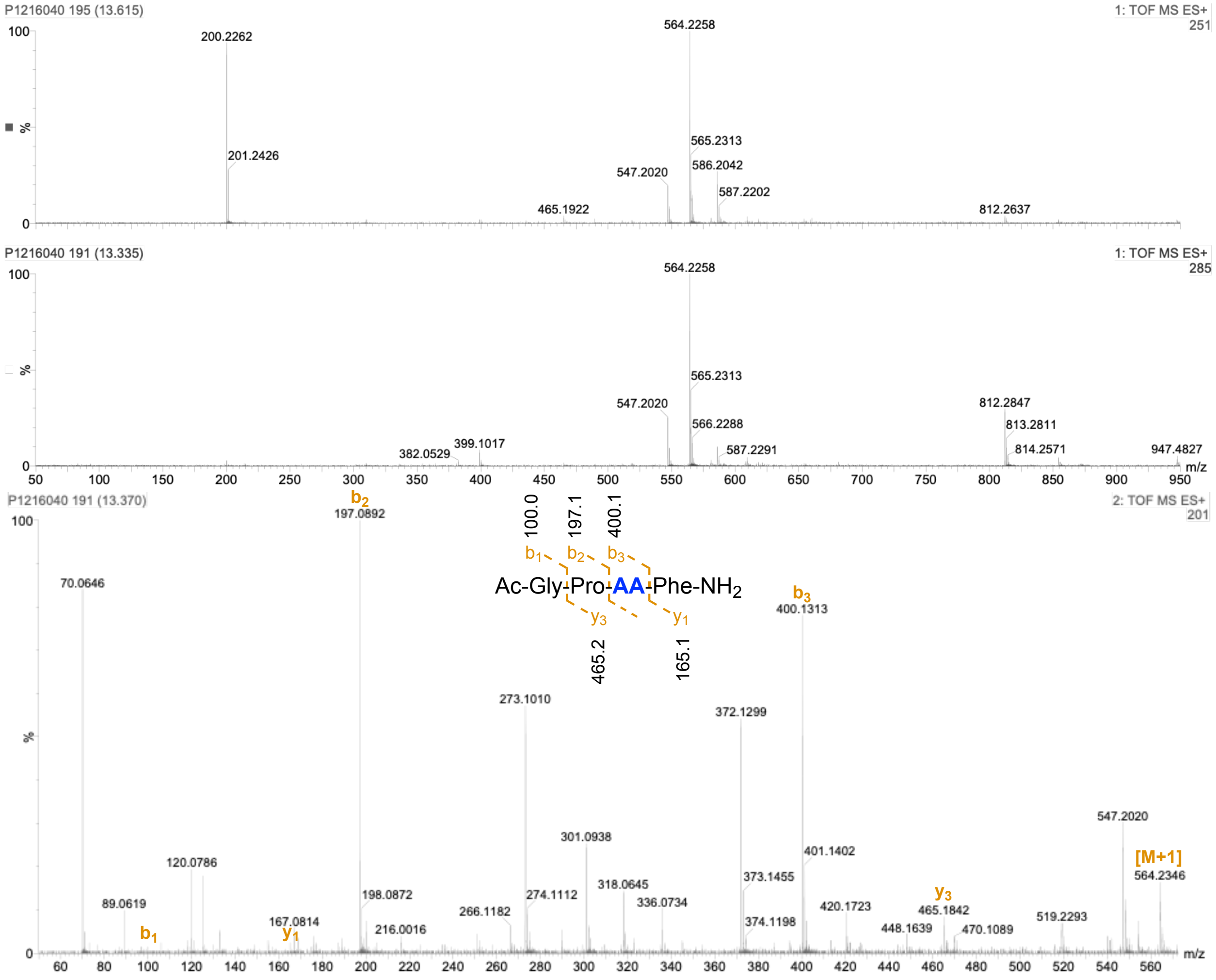

P1216040 195 (13.615)
1: TOF MS ES+
251
200.2262
564.2258
201.2426
565.2313
547.2020
586.2042
587.2202
465.1922
812.2637
P1216040 191 (13.335)
1: TOF MS ES+
285
564.2258
565.2313
547.2020
812.2847
813.2811
566.2288
814.2571
947.4827
382.0529
399.1017
587.2291
m/z
P1216040 191 (13.370)
2: TOF MS ES+
201
b2
197.0892
100.0
197.1
400.1
Ac-Gly-Pro-AA-Phe-NH2
465.2
165.1
70.0646
b3
400.1313
273.1010
372.1299
301.0938
401.1402
547.2020
[M+1]
564.2346
120.0786
318.0645
373.1455
336.0734
420.1723
y3
465.1842
89.0619
198.0872
266.1182
274.1112
b1
y1
167.0814
216.0016
374.1198
448.1639
470.1089
519.2293
m/z

5D': MW = 563.7, Purity = 59.6%, Yield = 1.6% [0.021 mg]

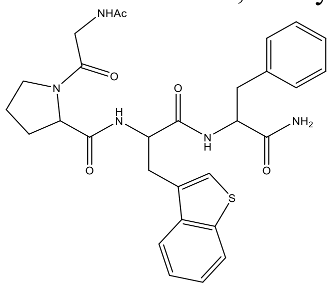

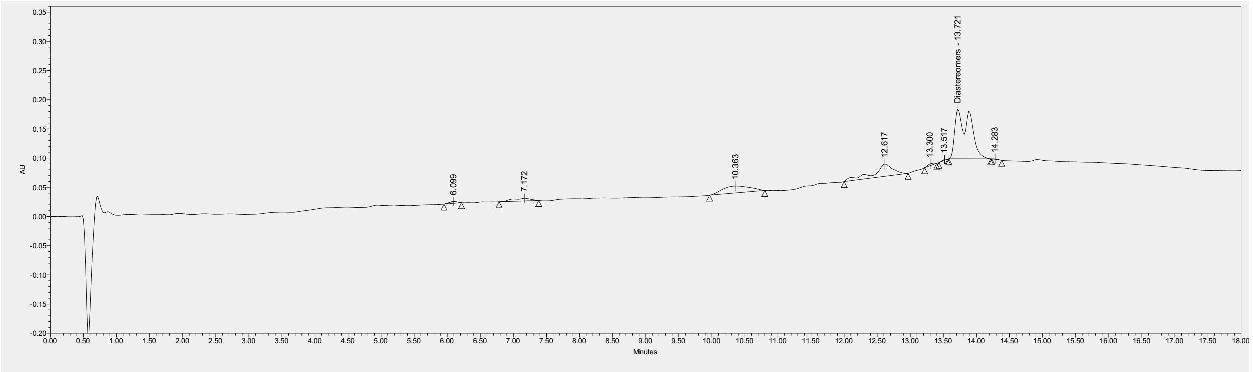

	Name	Retention Time	Area	% Area
1		6.099	25698	1.14
2		7.172	89723	3.98
3		10.363	340361	15.08
4		12.617	425364	18.85
5		13.300	21002	0.93
6		13.517	7119	0.32
7	Diastereomers	13.721	1344578	59.59
8		14.283	2625	0.12

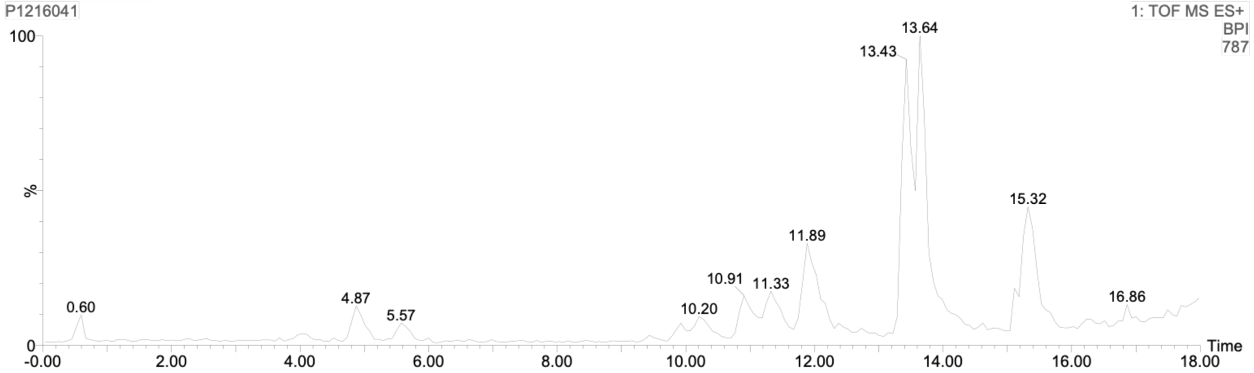

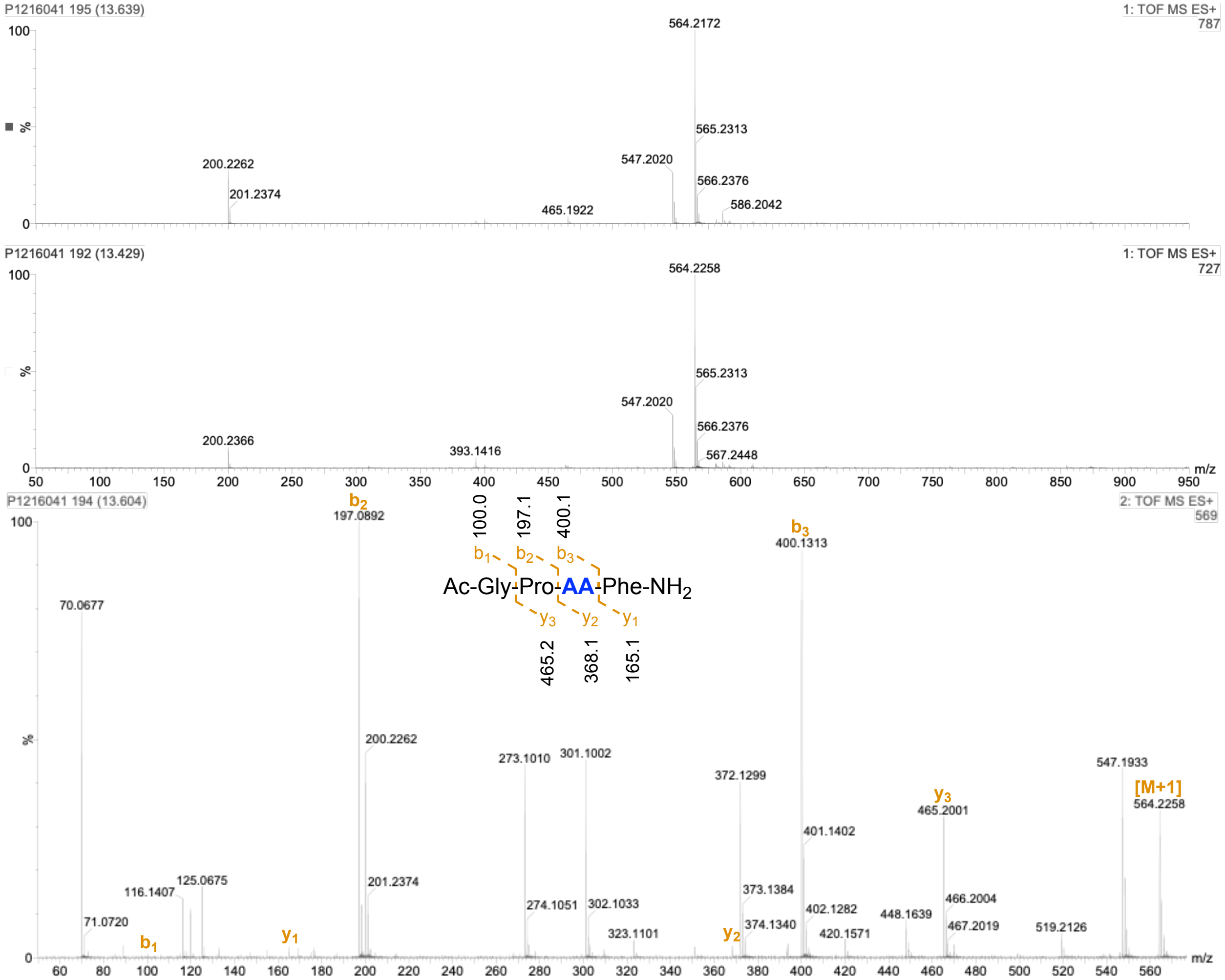
P1216041 195 (13.639)
1: TOF MS ES+
787
564.2172
565.2313
547.2020
200.2262
201.2374
566.2376
586.2042
465.1922
P1216041 192 (13.429)
1: TOF MS ES+
727
564.2258
565.2313
547.2020
566.2376
200.2366
393.1416
567.2448
m/z
P1216041 194 (13.604)
2: TOF MS ES+
569
b2
197.0892
100.0
197.1
400.1
b1
b2
b3
Ac-Gly-Pro-AA-Phe-NH2
y3
y2
y1
465.2
368.1
165.1
b3
400.1313
70.0677
200.2262
273.1010
301.1002
372.1299
547.1933
[M+1]
564.2258
y3
465.2001
401.1402
116.1407
125.0675
201.2374
373.1384
71.0720
274.1051
302.1033
402.1282
448.1639
466.2004
374.1340
467.2019
519.2126
b1
y1
323.1101
y2
420.1571
m/z

6D': MW = 565.6, Purity = 75.0%, Yield = 18.7% [0.25 mg]

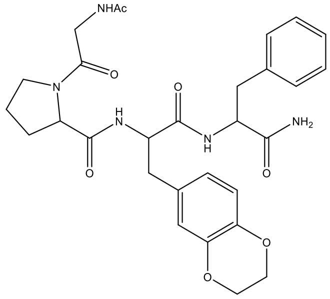

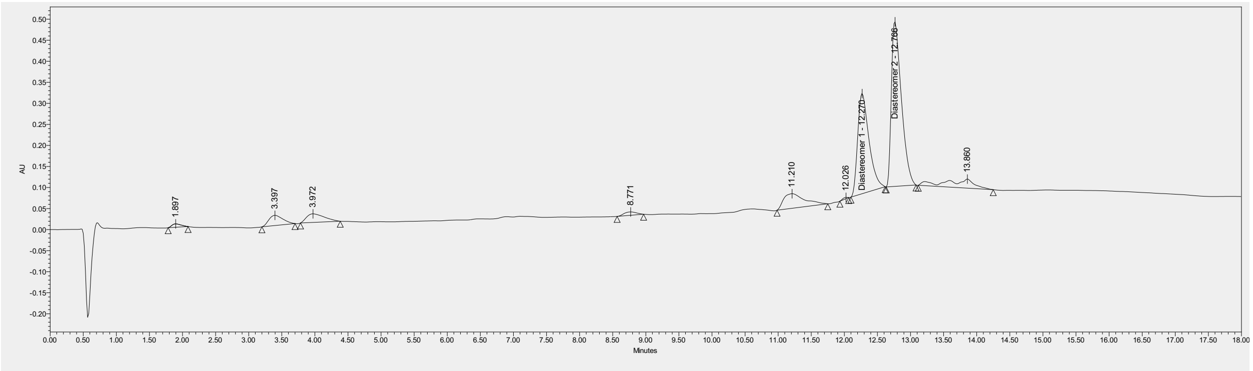

	Name	Retention Time	Area	% Area
1		1.897	77417	0.84
2		3.397	358664	3.87
3		3.972	379586	4.10
4		8.771	104258	1.13
5		11.210	769781	8.31
6		12.026	18190	0.20
7	Diastereomer 1	12.270	2835935	30.61
8	Diastereomer 2	12.766	4110785	44.37
9		13.860	609721	6.58

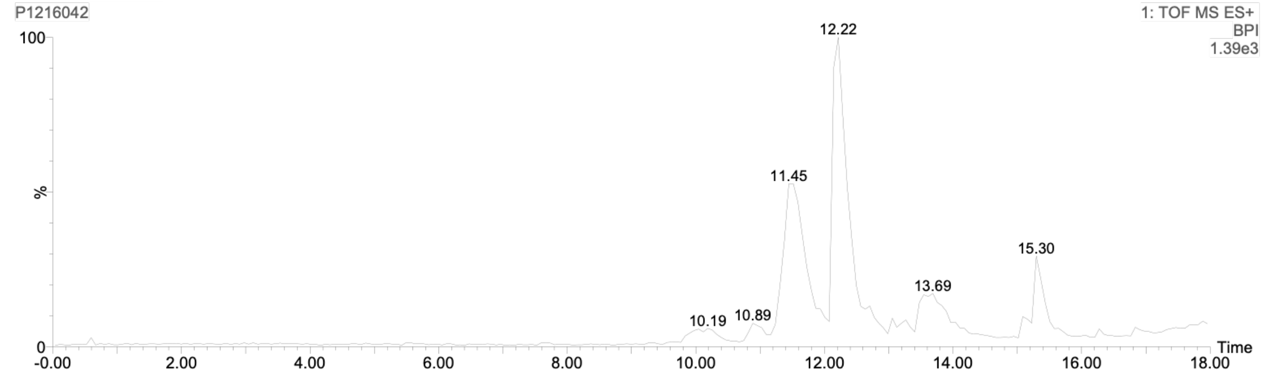

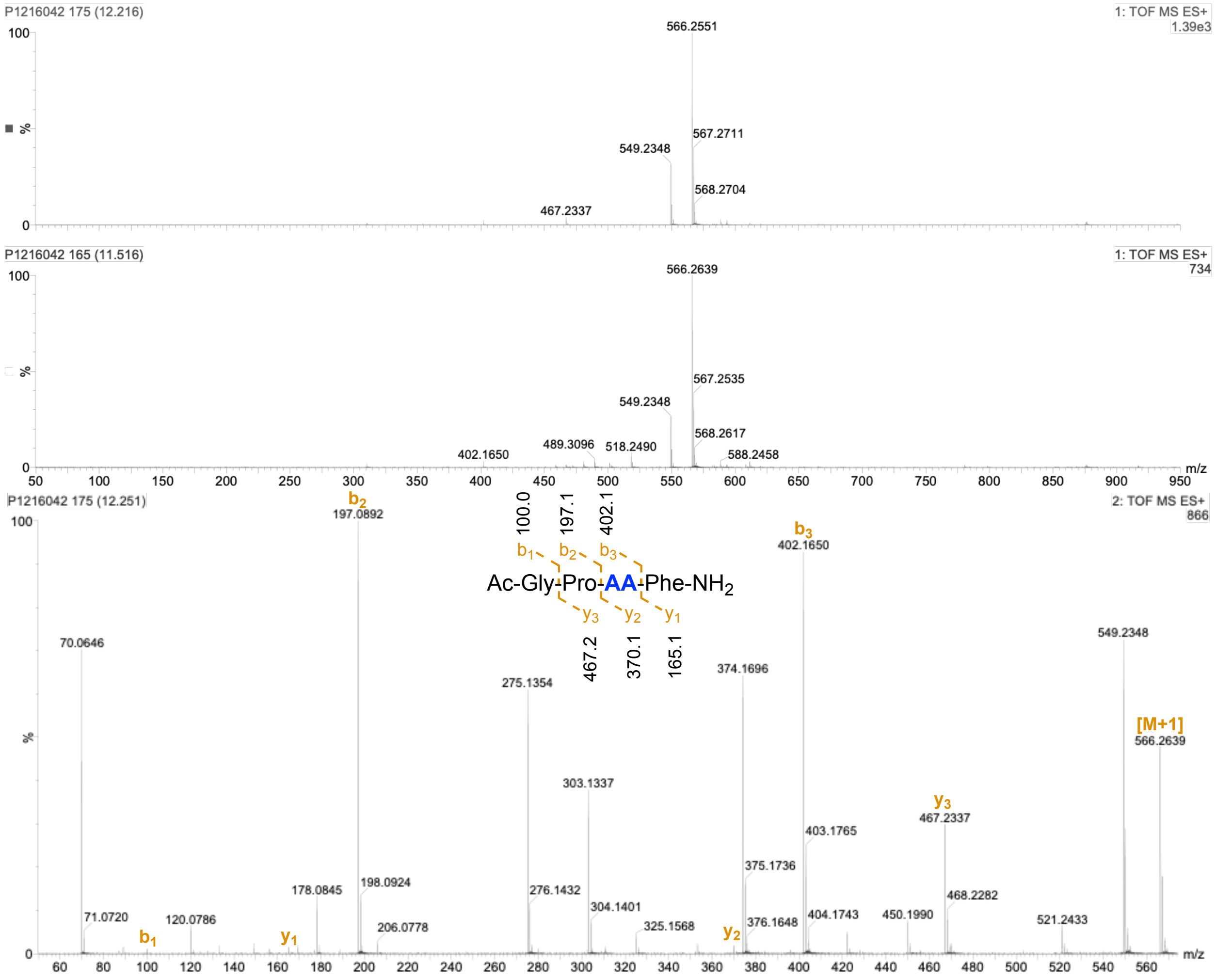
P1216042 175 (12.216)
1: TOF MS ES+
1.39e3
566.2551
567.2711
549.2348
568.2704
467.2337
P1216042 165 (11.516)
1: TOF MS ES+
734
566.2639
567.2535
549.2348
568.2617
489.3096
518.2490
402.1650
588.2458
m/z
P1216042 175 (12.251)
2: TOF MS ES+
866
b2
197.0892
b3
402.1650
Ac-Gly-Pro-AA-Phe-NH2
100.0
197.1
402.1
467.2
370.1
165.1
70.0646
275.1354
374.1696
549.2348
[M+1]
566.2639
303.1337
y3
467.2337
403.1765
375.1736
178.0845
198.0924
276.1432
304.1401
468.2282
71.0720
120.0786
325.1568
376.1648
404.1743
450.1990
521.2433
206.0778
b1
y1
y2
m/z

7D’: MW = 551.6, Purity = 82.8%, Yield = 16.8% [0.22 mg]

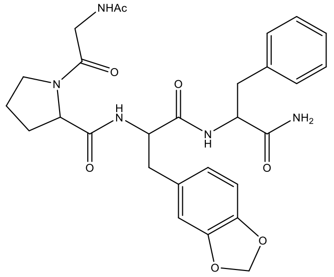

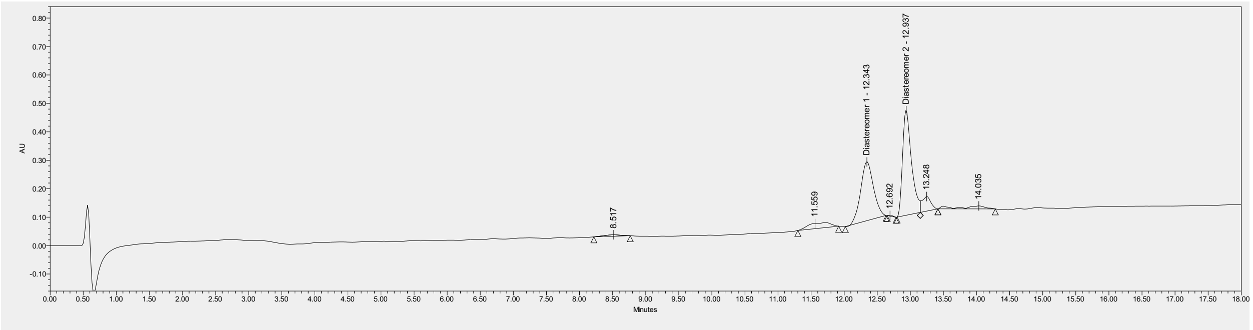

	Name	Retention Time	Area	% Area
1		8.517	88885	1.17
2		11.559	433533	5.73
3	Diastereomer 1	12.343	2801638	37.03
4		12.692	13378	0.18
5	Diastereomer 2	12.937	3459808	45.73
6		13.248	485206	6.41
7		14.035	282896	3.74

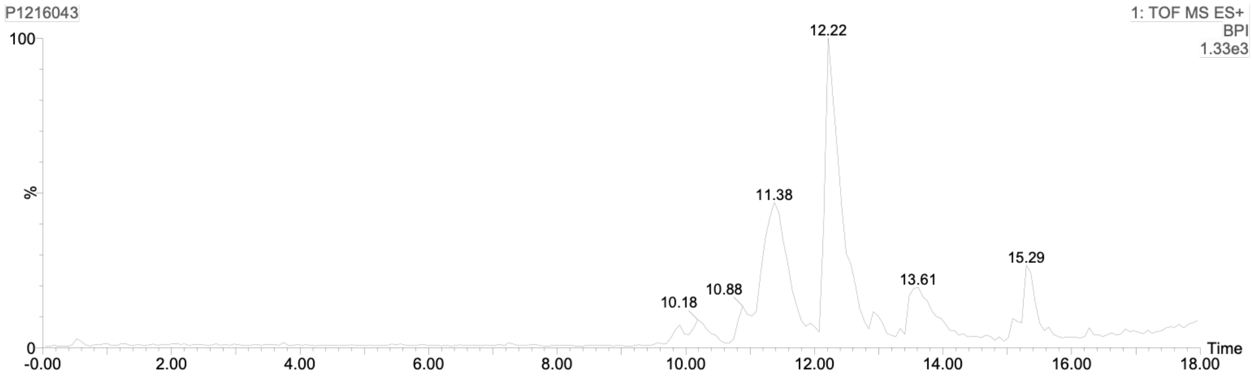

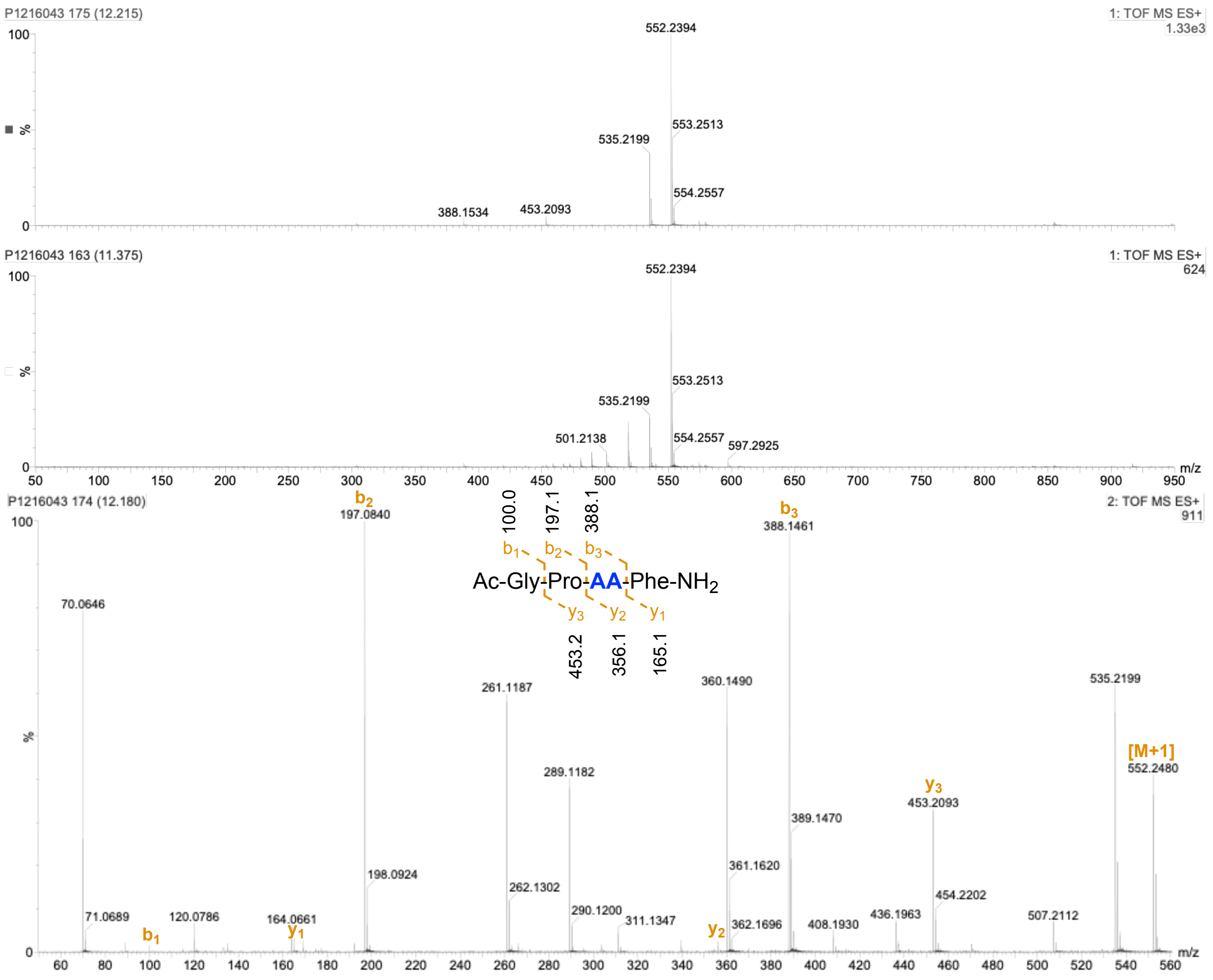
P1216043 175 (12.215)
1: TOF MS ES+
1.33e3
552.2394
553.2513
535.2199
554.2557
388.1534
453.2093
P1216043 163 (11.375)
1: TOF MS ES+
624
552.2394
553.2513
535.2199
501.2138
554.2557
597.2925
m/z
P1216043 174 (12.180)
2: TOF MS ES+
911
Ac-Gly-Pro-AA-Phe-NH2
b1
b2
b3
y1
y2
y3
100.0
197.1
388.1
453.2
356.1
165.1
b2
197.0840
b3
388.1461
70.0646
261.1187
360.1490
535.2199
[M+1]
552.2480
289.1182
y3
453.2093
389.1470
198.0924
361.1620
262.1302
454.2202
71.0689
120.0786
164.0661
290.1200
311.1347
362.1696
408.1930
436.1963
507.2112
b1
y1
y2
%
m/z

8D': MW = 497.6, Purity = 35.3%, Yield = 1.3% [0.015 mg]

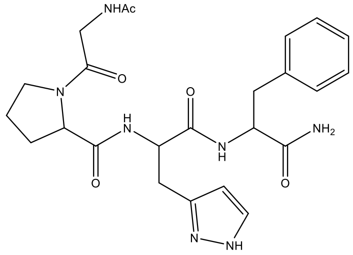

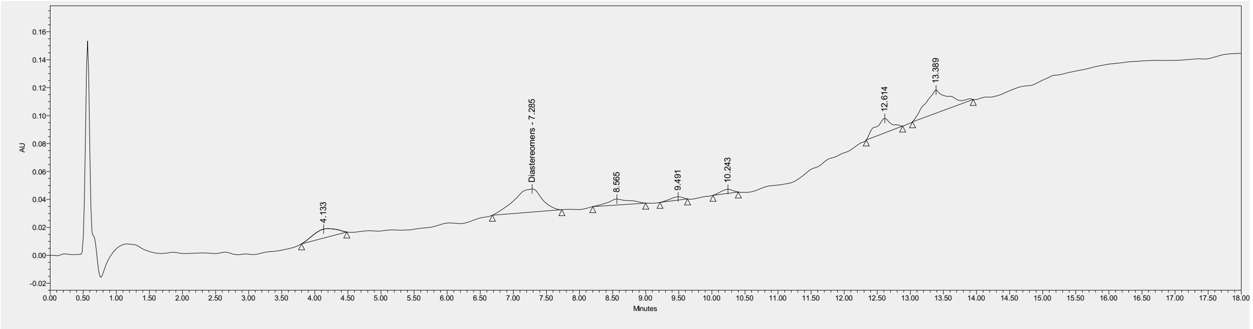

	Name	Retention Time	Area	% Area
1		4.133	153453	11.01
2	Diastereomers	7.285	492081	35.31
3		8.565	99977	7.17
4		9.491	31815	2.28
5		10.243	36572	2.62
6		12.614	176606	12.67
7		13.389	403133	28.93

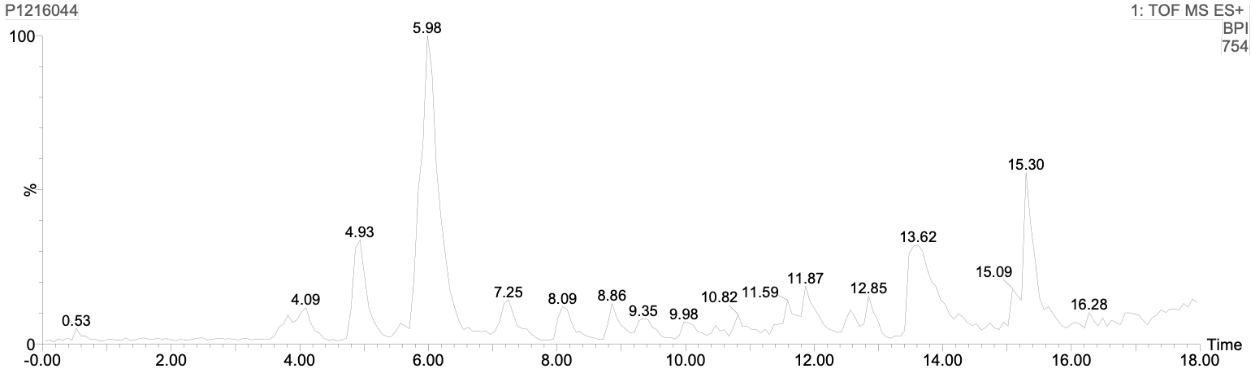

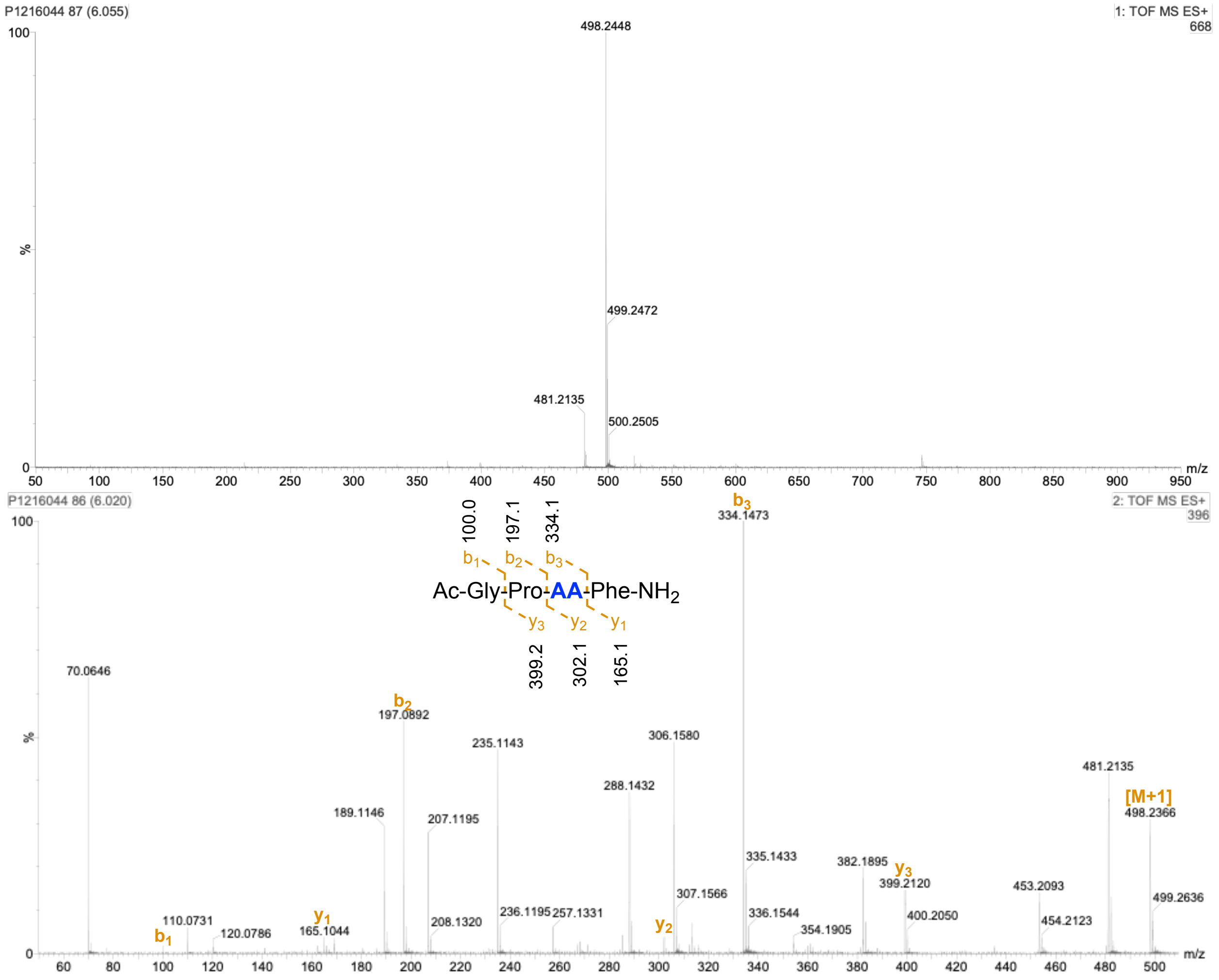
P1216044 87 (6.055)
1: TOF MS ES+
668
498.2448
499.2472
481.2135
500.2505
m/z
P1216044 86 (6.020)
2: TOF MS ES+
396
Ac-Gly-Pro-AA-Phe-NH2
b1 b2 b3
100.0 197.1 334.1
y3 y2 y1
399.2 302.1 165.1
b3
334.1473
70.0646
b2
197.0892
235.1143
306.1580
288.1432
481.2135
[M+1]
498.2366
189.1146
207.1195
335.1433
382.1895
y3
399.2120
453.2093
499.2636
307.1566
110.0731
b1
120.0786
y1
165.1044
208.1320
236.1195
257.1331
y2
336.1544
354.1905
400.2050
454.2123
m/z

9D': MW = 497.6, Purity = 28.3%, Yield = 2.1% [0.025 mg]

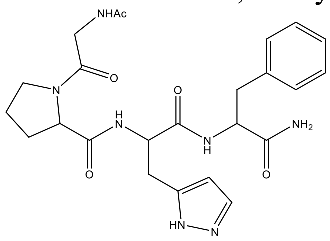

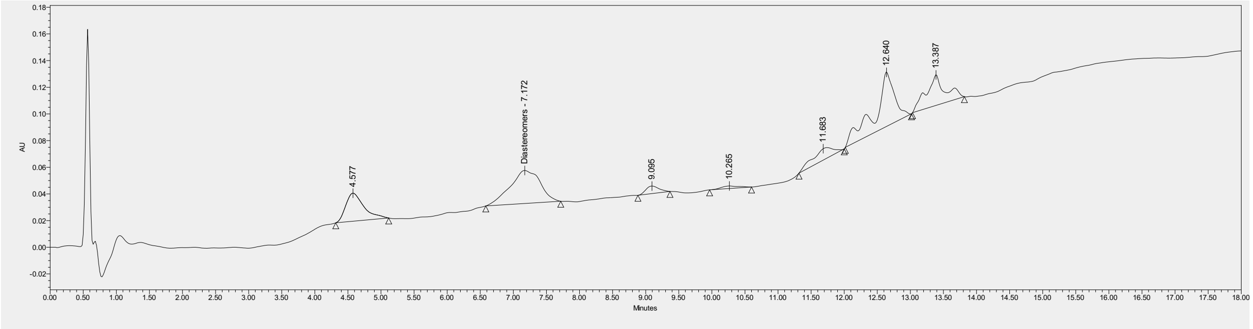

	Name	Retention Time	Area	% Area
1		4.577	403774	14.53
2	Diastereomers	7.172	785457	28.27
3		9.095	78609	2.83
4		10.265	34383	1.24
5		11.683	187582	6.75
6		12.640	835474	30.07
7		13.387	453567	16.32

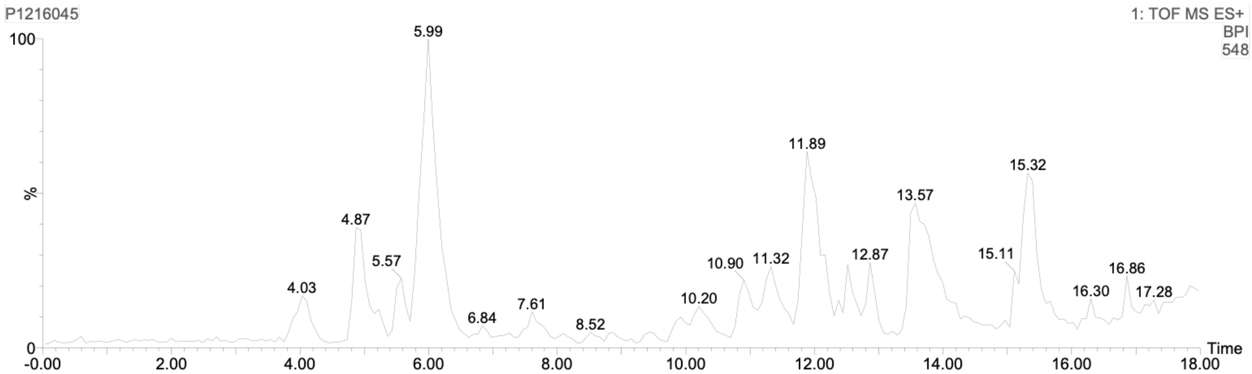

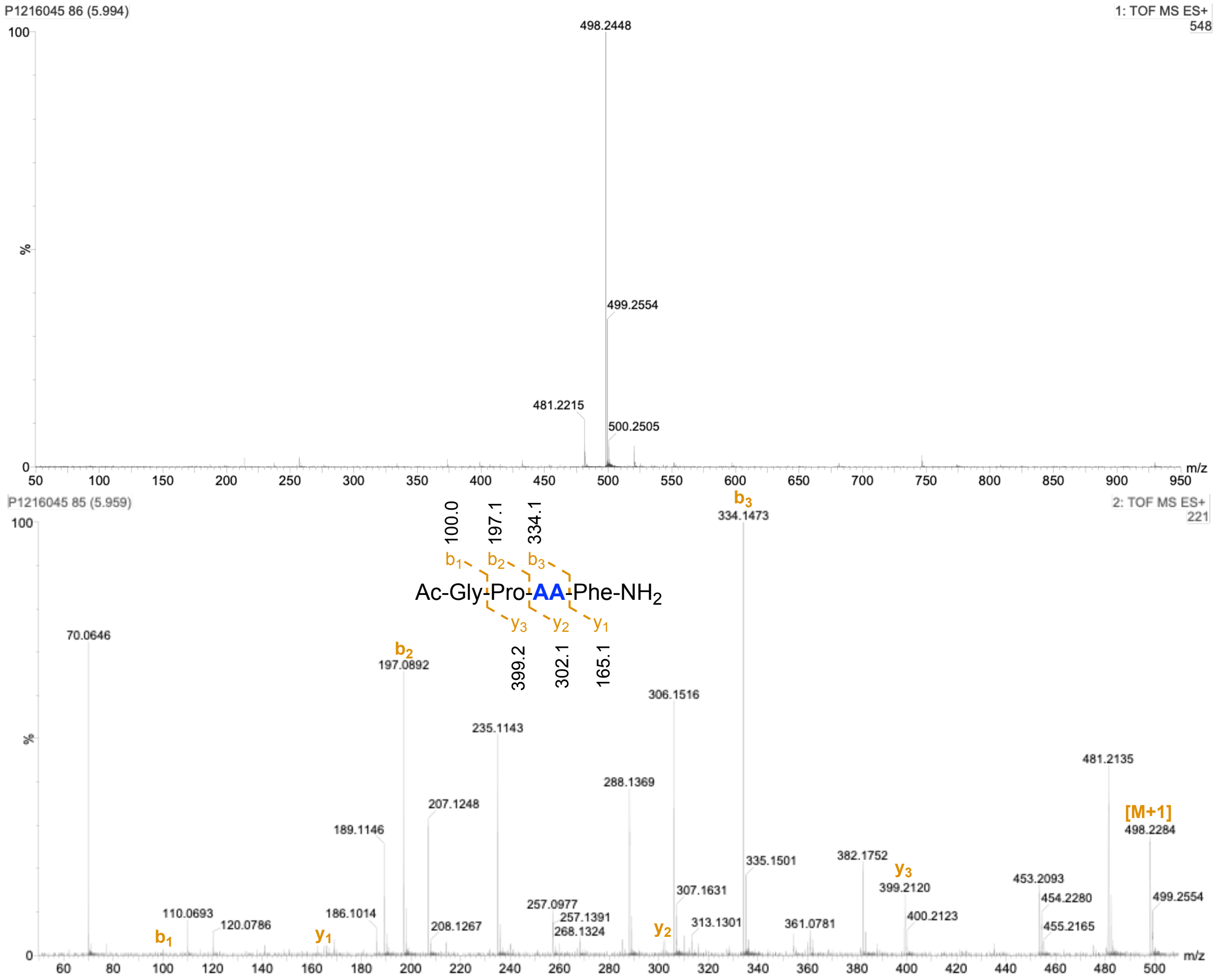

P1216045 86 (5.994)
1: TOF MS ES+
548
498.2448
499.2554
481.2215
500.2505
m/z
P1216045 85 (5.959)
2: TOF MS ES+
221
b3
334.1473
100.0
197.1
334.1
Ac-Gly-Pro-AA-Phe-NH2
399.2
302.1
165.1
70.0646
b2
197.0892
306.1516
235.1143
481.2135
288.1369
207.1248
[M+1]
498.2284
189.1146
335.1501
382.1752
y3
399.2120
453.2093
454.2280
499.2554
110.0693
186.1014
257.0977
257.1391
307.1631
400.2123
120.0786
208.1267
268.1324
313.1301
361.0781
455.2165
b1
y1
y2

10D’: MW = 511.6, Purity = 5.2%, Yield = 0.29% [0.004 mg]

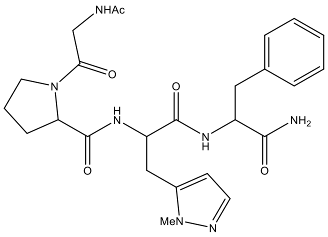

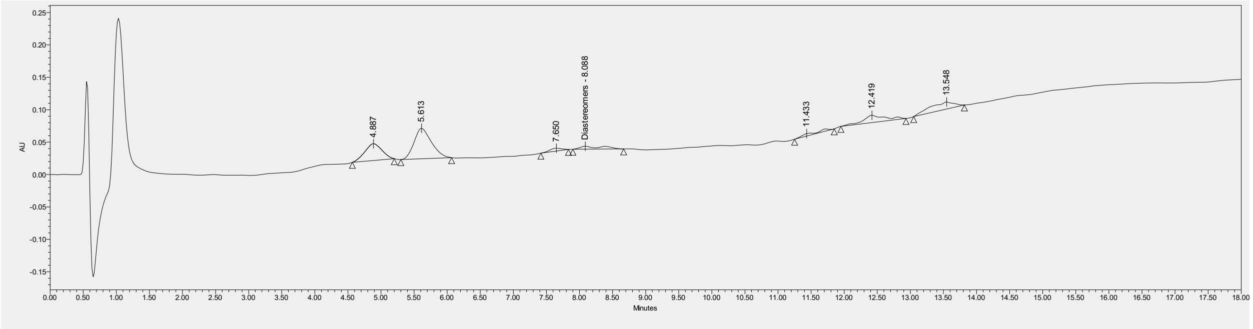

	Name	Retention Time	Area	% Area
1		4.887	468431	22.07
2		5.613	859604	40.49
3		7.650	55995	2.64
4	Diastereomers	8.088	109378	5.15
5		11.433	71822	3.38
6		12.419	260055	12.25
7		13.548	297654	14.02

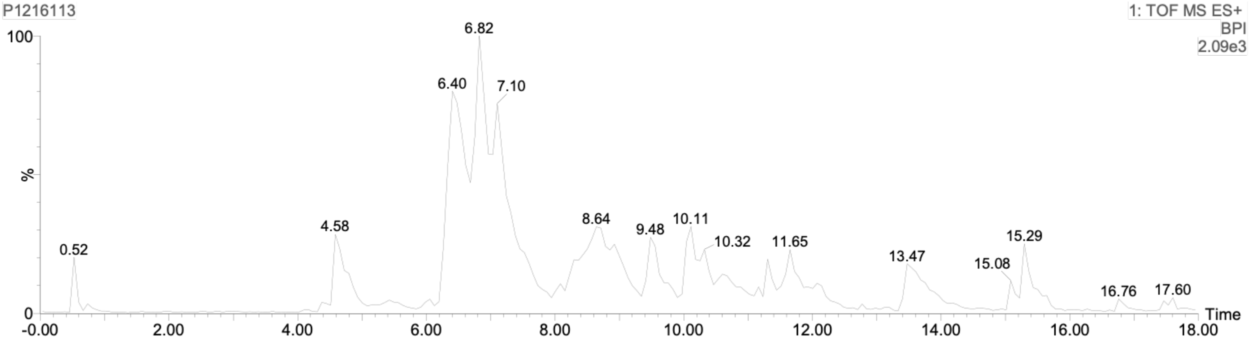

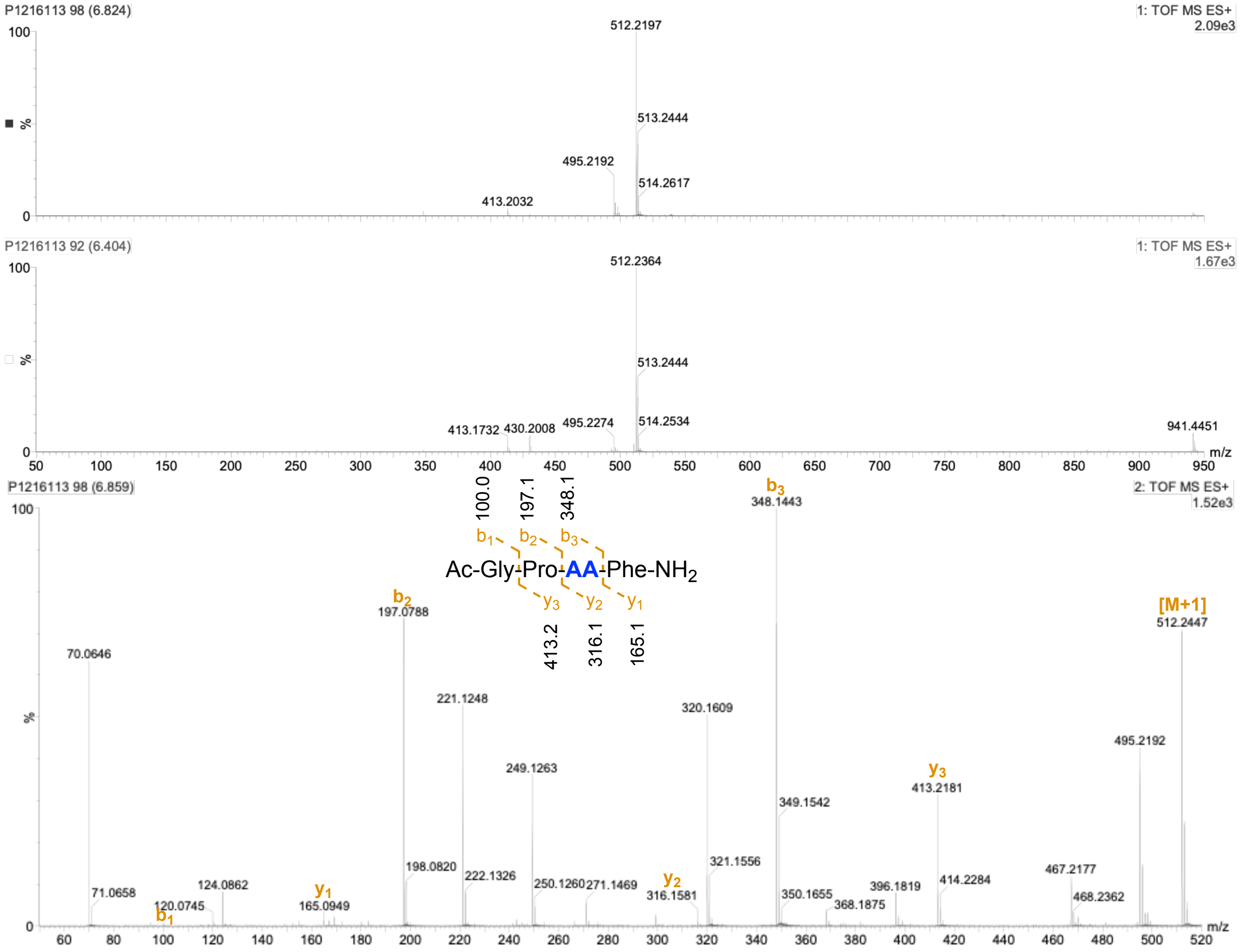

P1216113 98 (6.824)
1: TOF MS ES+
2.09e3
512.2197
513.2444
495.2192
514.2617
413.2032
P1216113 92 (6.404)
1: TOF MS ES+
1.67e3
512.2364
513.2444
413.1732
430.2008
495.2274
514.2534
941.4451
m/z
P1216113 98 (6.859)
2: TOF MS ES+
1.52e3
Ac-Gly-Pro-AA-Phe-NH2
b1 b2 b3
100.0 197.1 348.1
y3 y2 y1
413.2 316.1 165.1
b3
348.1443
b2
197.0788
[M+1]
512.2447
70.0646
221.1248
320.1609
495.2192
249.1263
y3
413.2181
349.1542
321.1556
198.0820
222.1326
467.2177
71.0658
124.0862
y1
165.0949
y2
316.1581
250.1260
271.1469
350.1655
396.1819
414.2284
368.1875
468.2362
120.0745
b1
%
m/z

11D': MW = 513.6, Purity = 68.8%, Yield = 7.5% [0.090 mg]

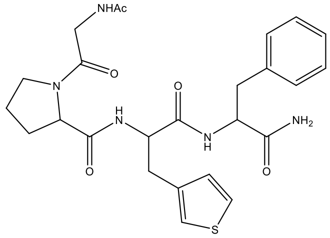

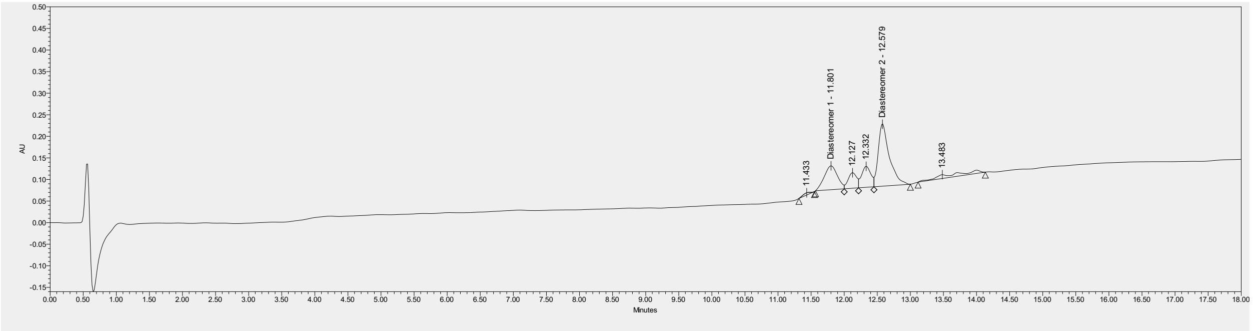

	Name	Retention Time	Area	% Area
1		11.433	38481	1.08
2	Diastereomer 1	11.801	765423	21.44
3		12.127	324157	9.08
4		12.332	480241	13.45
5	Diastereomer 2	12.579	1691680	47.39
6		13.483	269945	7.56

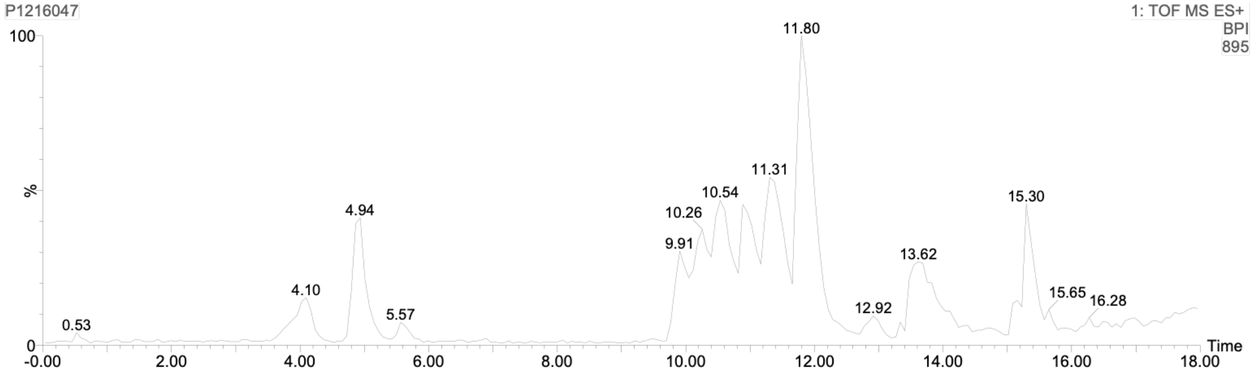

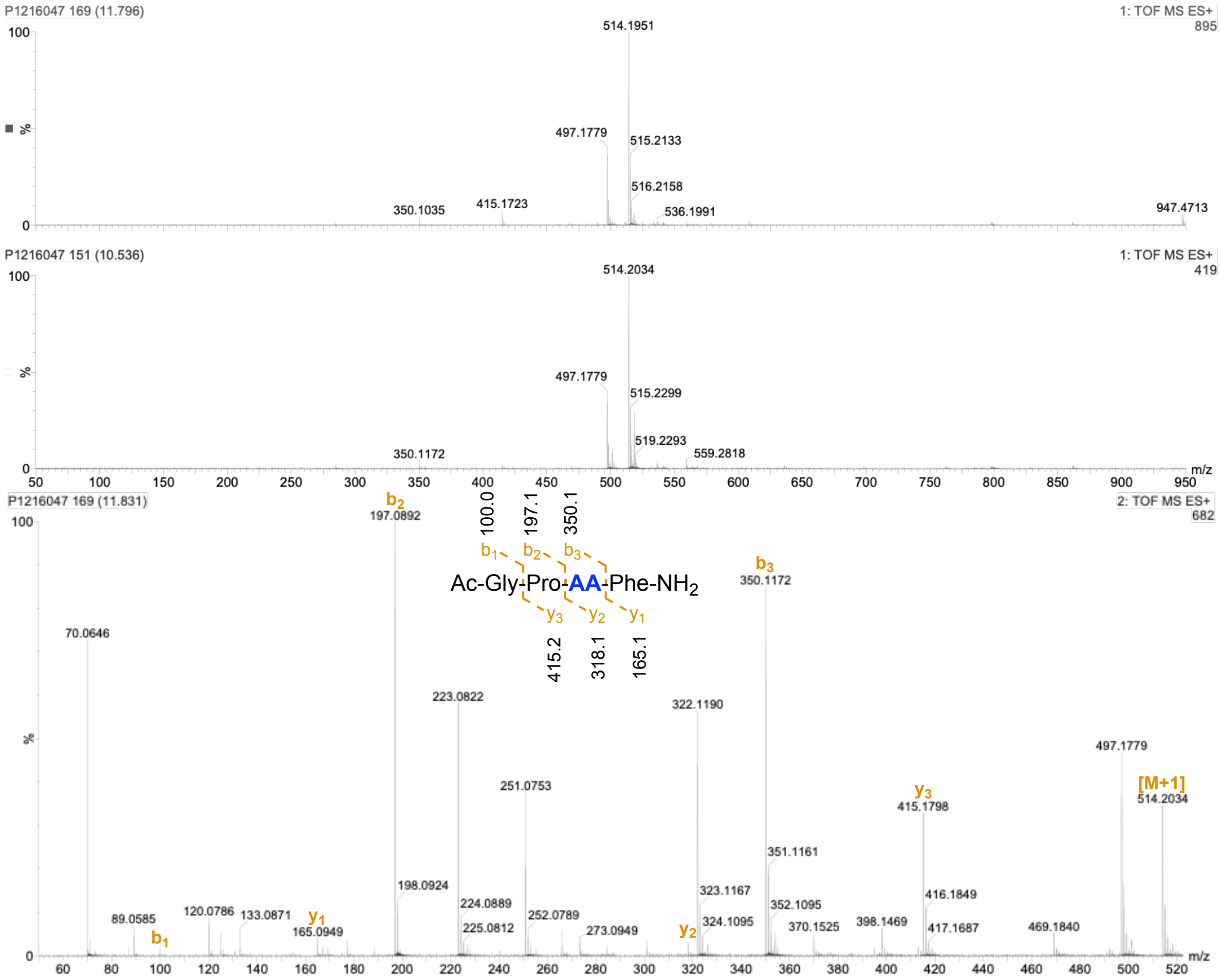
P1216047 169 (11.796)
1: TOF MS ES+
895
514.1951
497.1779
515.2133
516.2158
350.1035
415.1723
536.1991
947.4713
P1216047 151 (10.536)
1: TOF MS ES+
419
514.2034
497.1779
515.2299
519.2293
559.2818
350.1172
P1216047 169 (11.831)
2: TOF MS ES+
682
b2
197.0892
100.0
197.1
350.1
Ac-Gly-Pro-AA-Phe-NH2
415.2
318.1
165.1
b3
350.1172
70.0646
223.0822
322.1190
251.0753
497.1779
[M+1]
514.2034
y3
415.1798
351.1161
198.0924
416.1849
323.1167
224.0889
352.1095
120.0786
133.0871
89.0585
y1
165.0949
b1
225.0812
252.0789
273.0949
y2
324.1095
370.1525
398.1469
417.1687
469.1840
m/z

12D': MW = 513.6, Purity = 48.4%, Yield = 3.2% [0.038 mg]

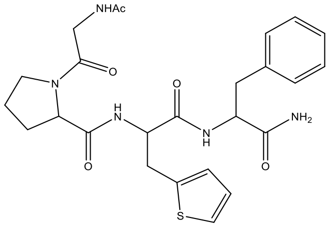

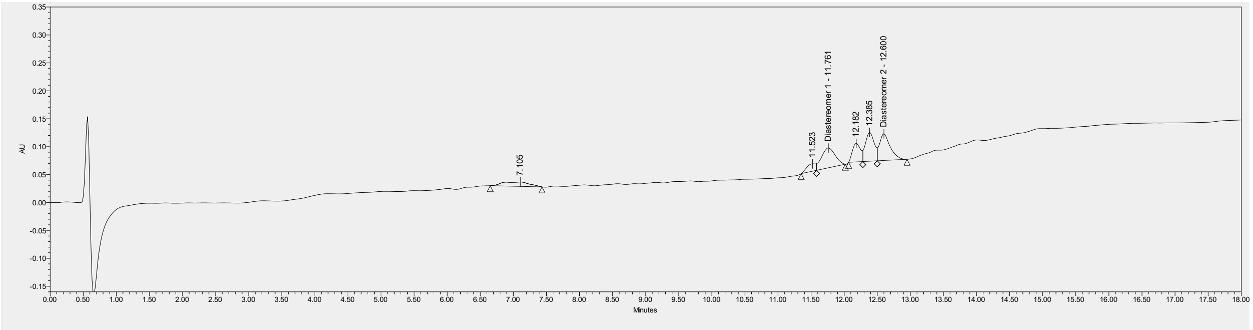

	Name	Retention Time	Area	% Area
1		7.105	221663	10.23
2		11.523	129609	5.98
3	Diastereomer 1	11.761	512350	23.64
4		12.182	287272	13.26
5		12.385	480605	22.18
6	Diastereomer 2	12.600	535542	24.71

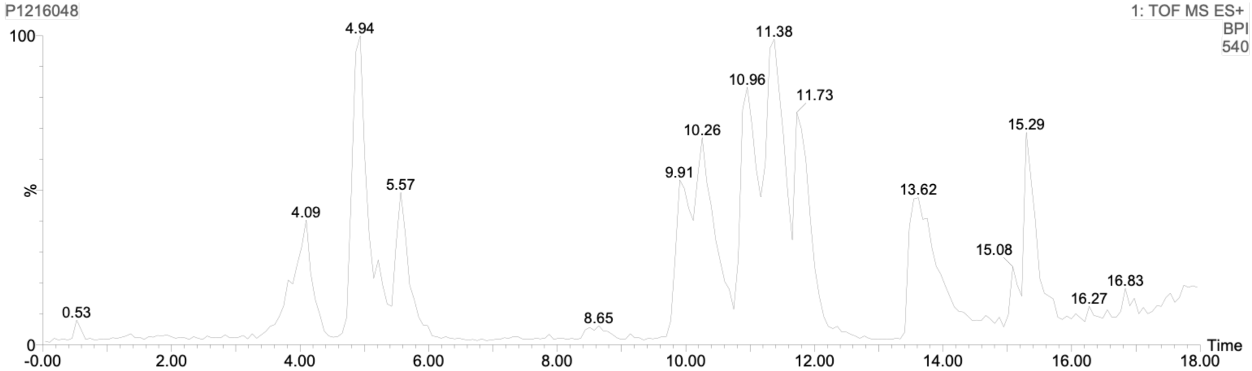

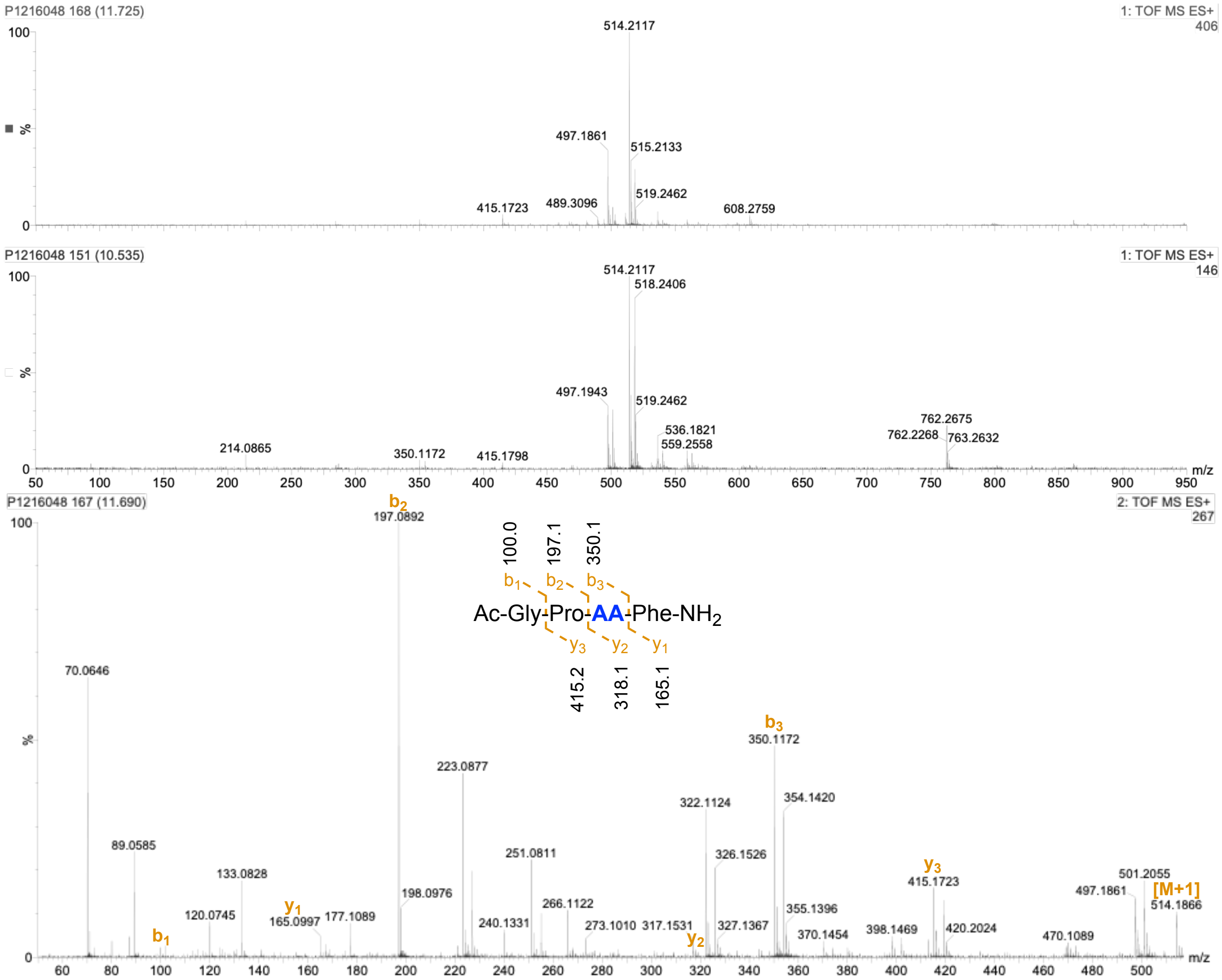

P1216048 168 (11.725)
1: TOF MS ES+
406
514.2117
497.1861
515.2133
519.2462
415.1723
489.3096
608.2759
P1216048 151 (10.535)
1: TOF MS ES+
146
514.2117
518.2406
497.1943
519.2462
214.0865
350.1172
415.1798
536.1821
559.2558
762.2675
762.2268
763.2632
m/z
P1216048 167 (11.690)
2: TOF MS ES+
267
b2
197.0892
Ac-Gly-Pro-AA-Phe-NH2
100.0
197.1
350.1
415.2
318.1
165.1
70.0646
89.0585
133.0828
120.0745
b1
y1
165.0997
177.1089
198.0976
223.0877
251.0811
240.1331
266.1122
273.1010
317.1531
y2
322.1124
326.1526
327.1367
b3
350.1172
354.1420
355.1396
370.1454
398.1469
y3
415.1723
420.2024
470.1089
497.1861
501.2055
[M+1]
514.1866
m/z

1E': MW = 521.6, Purity = 58.4%, Yield = 5.9% [0.072 mg]

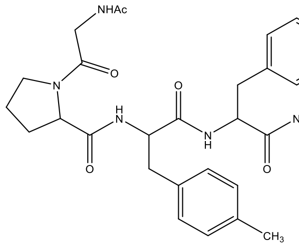

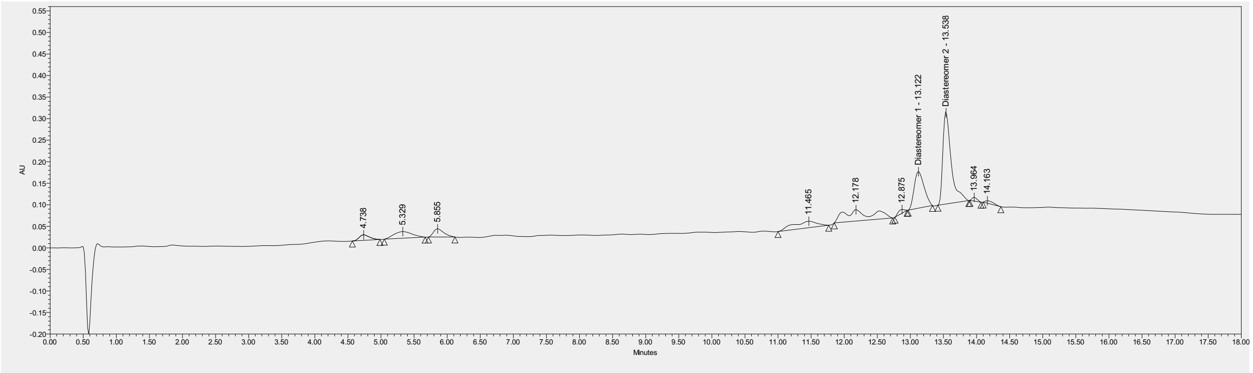

	Name	Retention Time	Area	% Area
1		4.738	138340	2.94
2		5.329	295104	6.28
3		5.855	206634	4.39
4		11.465	390770	8.31
5		12.178	763660	16.24
6		12.875	60693	1.29
7	Diastereomer 1	13.122	878147	18.68
8	Diastereomer 2	13.538	1867833	39.73
9		13.964	46627	0.99
10		14.163	53918	1.15

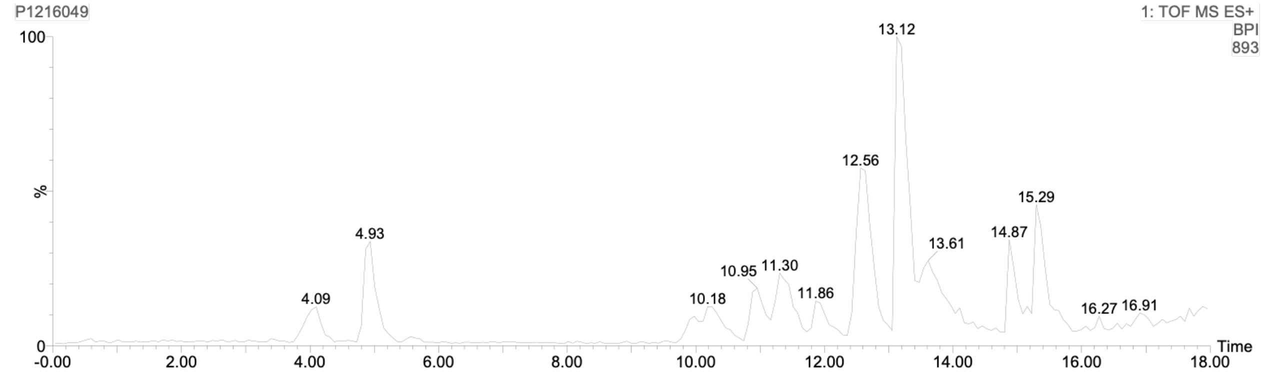

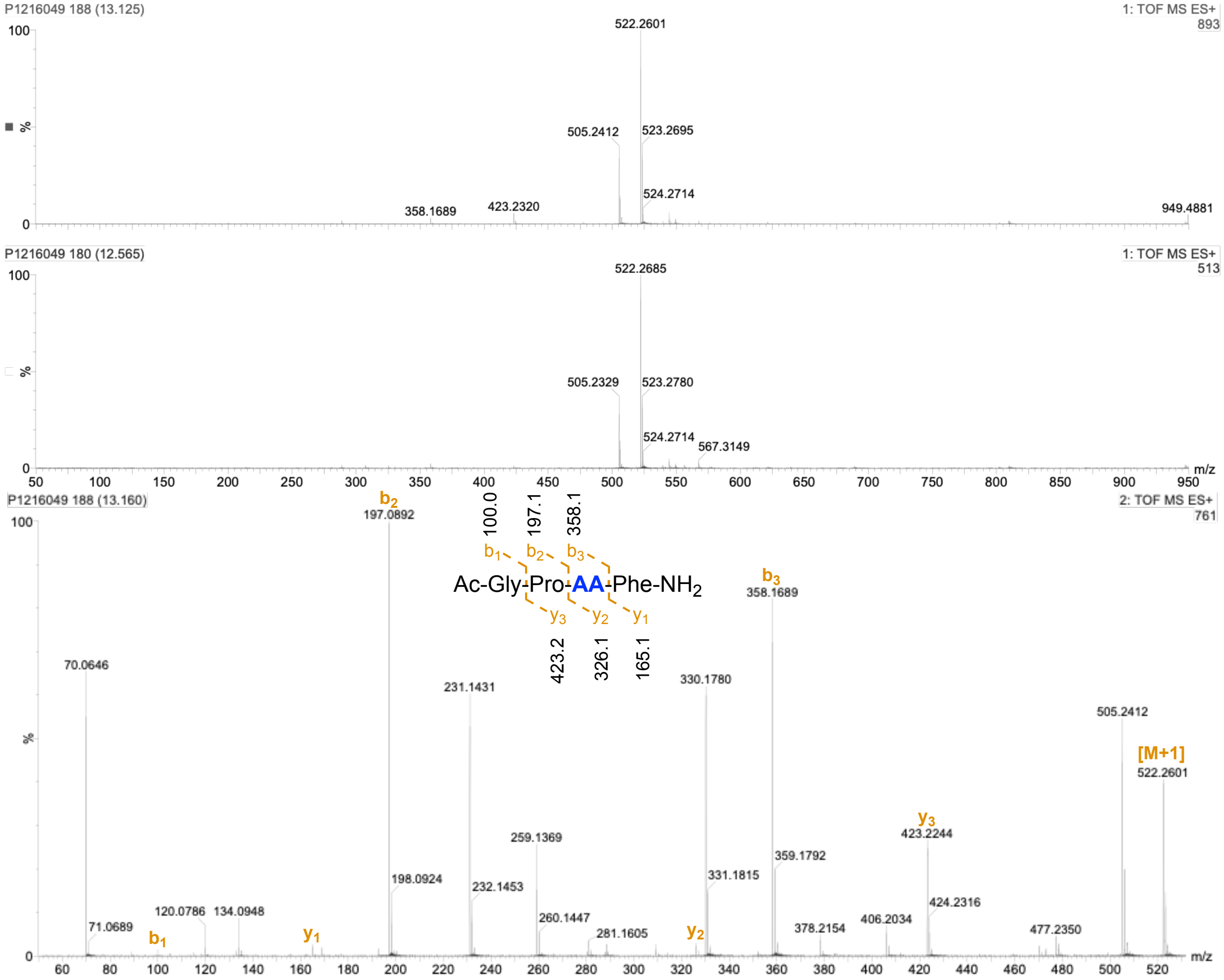
P1216049 188 (13.125)
1: TOF MS ES+
893
522.2601
505.2412
523.2695
524.2714
358.1689
423.2320
949.4881
P1216049 180 (12.565)
1: TOF MS ES+
513
522.2685
505.2329
523.2780
524.2714
567.3149
m/z
P1216049 188 (13.160)
2: TOF MS ES+
761
b2
197.0892
100.0
197.1
358.1
b1
b2
b3
Ac-Gly-Pro-AA-Phe-NH2
y3
y2
y1
423.2
326.1
165.1
b3
358.1689
70.0646
231.1431
330.1780
505.2412
[M+1]
522.2601
y3
423.2244
259.1369
359.1792
198.0924
232.1453
331.1815
424.2316
120.0786
134.0948
260.1447
406.2034
71.0689
378.2154
477.2350
281.1605
b1
y1
y2
m/z

2E': MW = 535.7, Purity = 45.8%, Yield = 15.7% [0.20 mg]

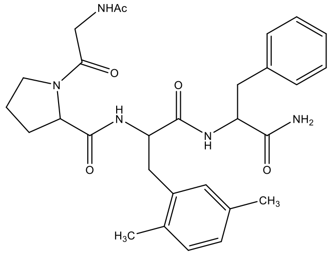

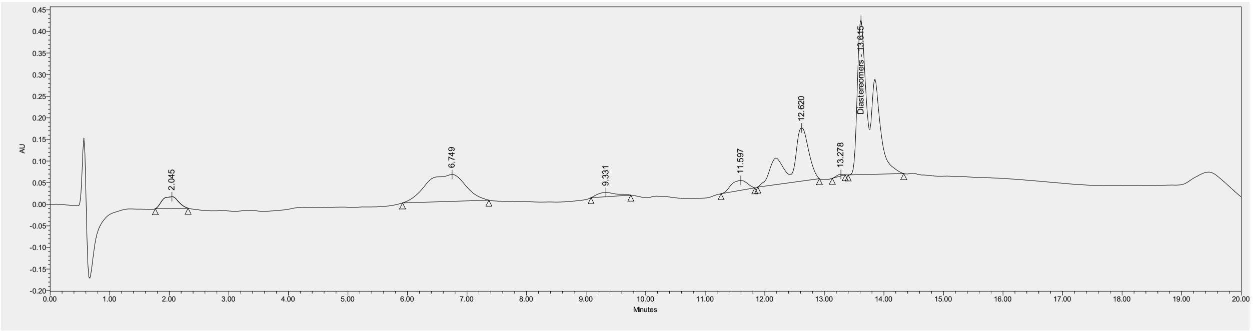

	Name	Retention Time	Area	% Area
1		2.045	515344	4.05
2		6.749	2875647	22.57
3		9.331	219245	1.72
4		11.597	435764	3.42
5		12.620	2824625	22.17
6		13.278	29275	0.23
7	Diastereomers	13.615	5840347	45.84

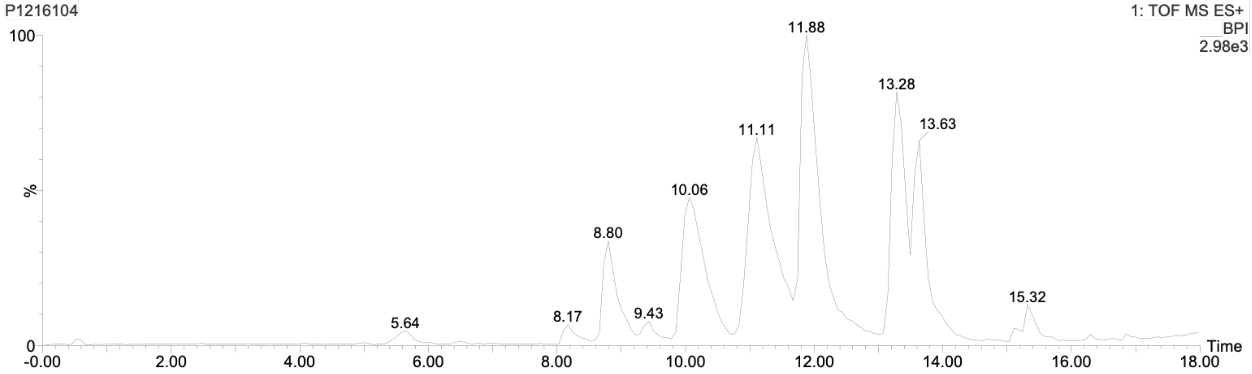

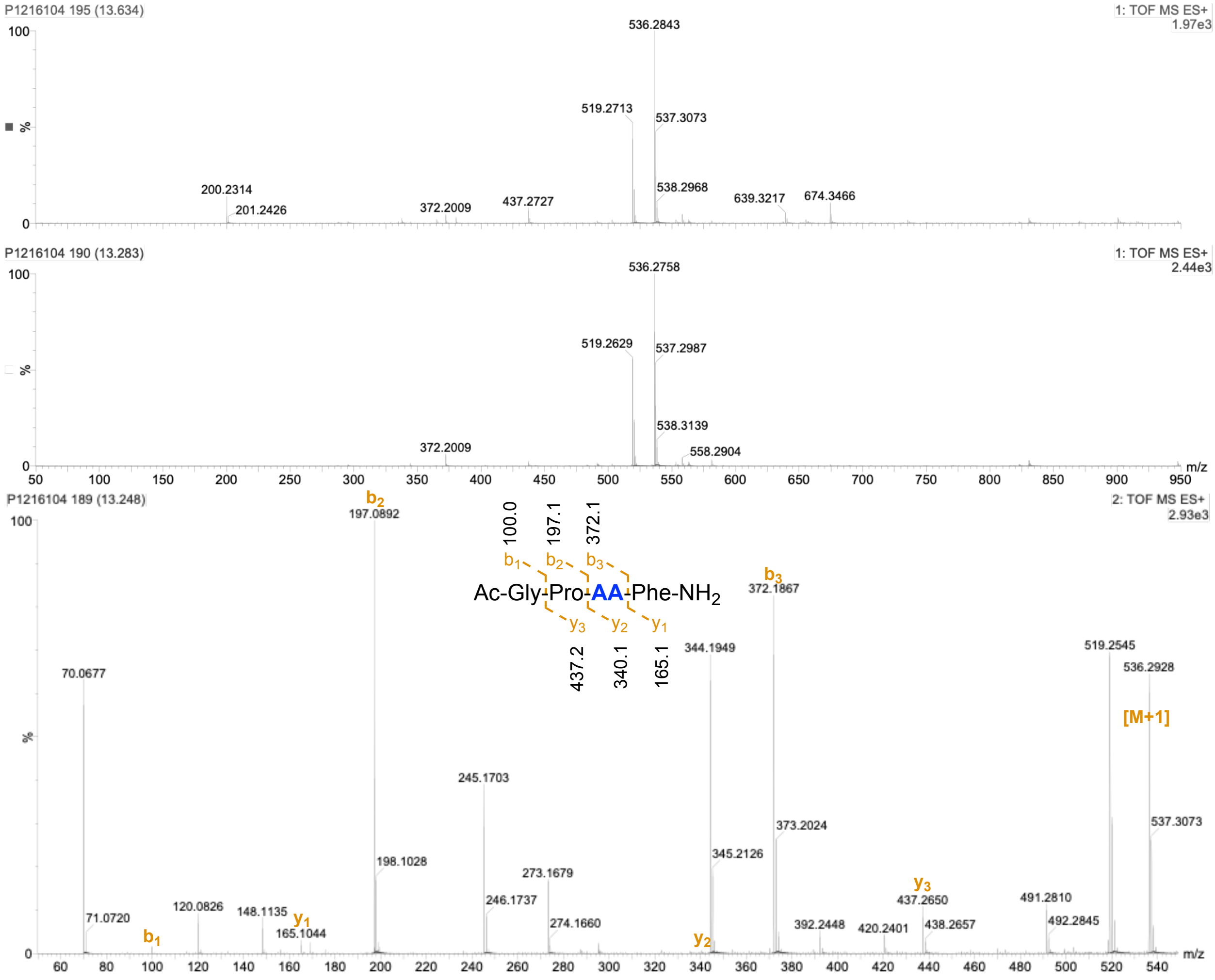
P1216104 195 (13.634)
1: TOF MS ES+
1.97e3
536.2843
519.2713
537.3073
200.2314
201.2426
372.2009
437.2727
538.2968
639.3217
674.3466
P1216104 190 (13.283)
1: TOF MS ES+
2.44e3
536.2758
519.2629
537.2987
538.3139
372.2009
558.2904
m/z
P1216104 189 (13.248)
2: TOF MS ES+
2.93e3
b2
197.0892
100.0
197.1
372.1
b1 b2 b3
Ac-Gly-Pro-AA-Phe-NH2
y3 y2 y1
437.2
340.1
165.1
b3
372.1867
344.1949
519.2545
536.2928
[M+1]
70.0677
245.1703
373.2024
537.3073
345.2126
198.1028
273.1679
y3
437.2650
491.2810
246.1737
71.0720
120.0826
148.1135
y1
165.1044
b1
274.1660
y2
392.2448
420.2401
438.2657
492.2845
m/z

3E’: MW = 547.6, Purity = 63.9%, Yield = 3.3% [0.042 mg]

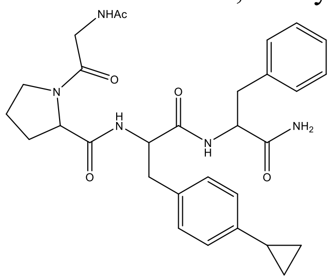

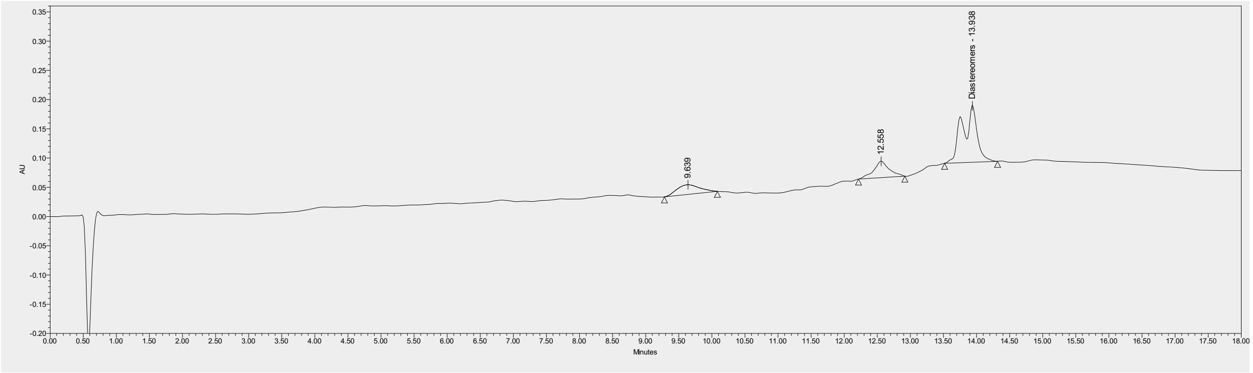

	Name	Retention Time	Area	% Area
1		9.639	402424	16.87
2		12.558	459959	19.28
3	Diastereomers	13.938	1523338	63.85

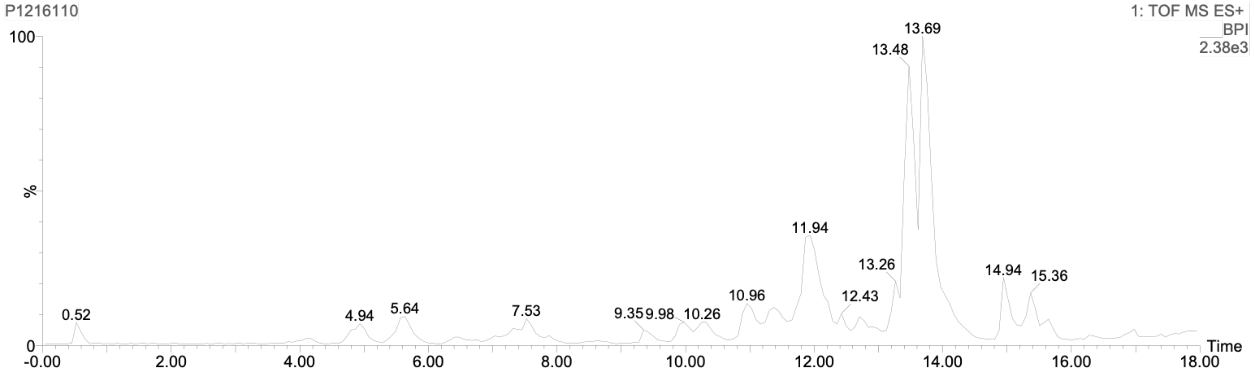

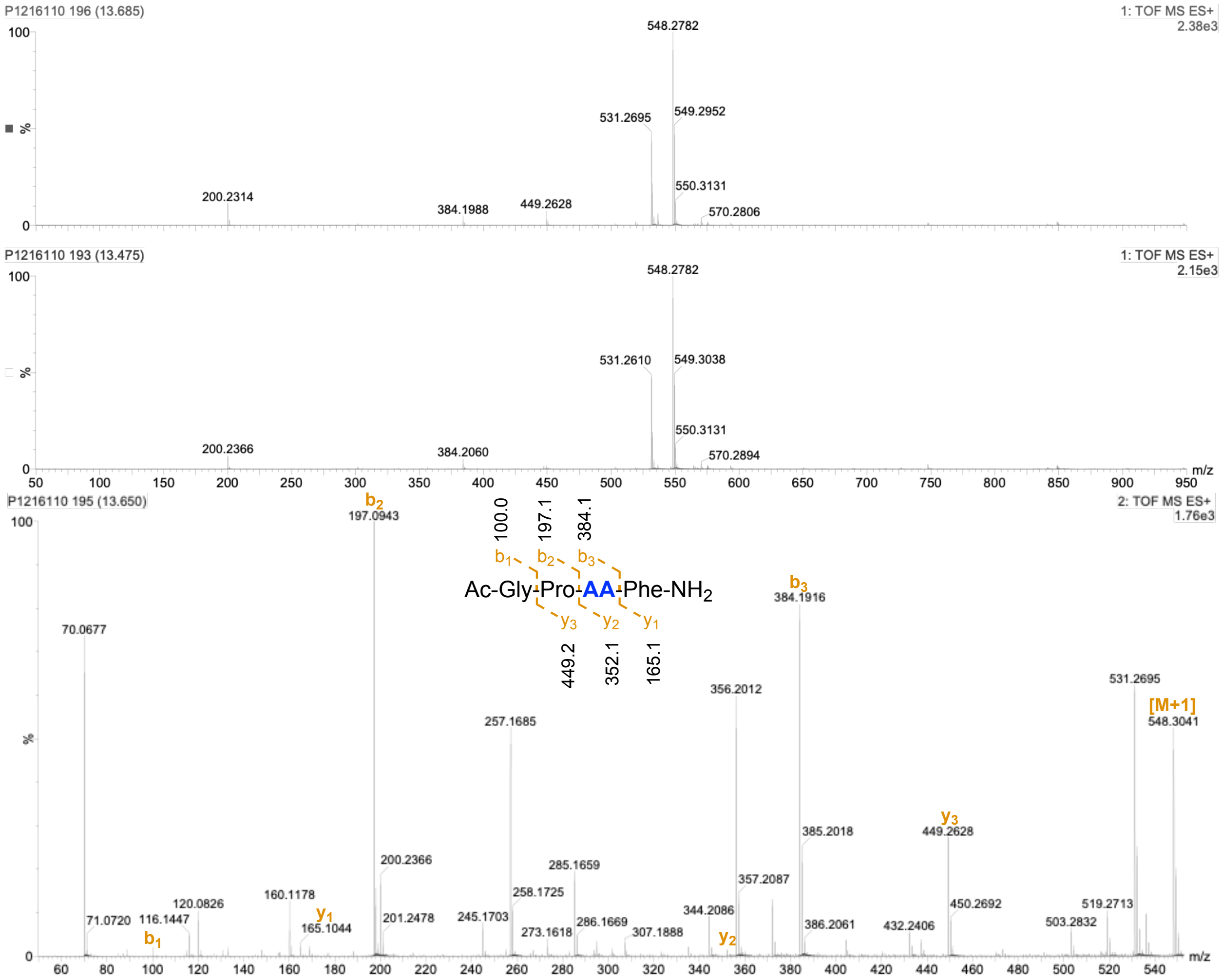
P1216110 196 (13.685)
1: TOF MS ES+
2.38e3
548.2782
531.2695
549.2952
550.3131
200.2314
384.1988
449.2628
570.2806
P1216110 193 (13.475)
1: TOF MS ES+
2.15e3
548.2782
531.2610
549.3038
550.3131
200.2366
384.2060
570.2894
m/z
P1216110 195 (13.650)
2: TOF MS ES+
1.76e3
b2
197.0943
Ac-Gly-Pro-AA-Phe-NH2
b1 b2 b3
y3 y2 y1
100.0
197.1
384.1
449.2
352.1
165.1
b3
384.1916
70.0677
531.2695
[M+1]
548.3041
356.2012
257.1685
y3
449.2628
385.2018
285.1659
200.2366
357.2087
258.1725
120.0826
160.1178
450.2692
519.2713
71.0720
116.1447
y1
165.1044
201.2478
245.1703
344.2086
b1
273.1618
286.1669
307.1888
y2
386.2061
432.2406
503.2832
m/z

4E’: MW = 563.7, Purity = 46.7%, Yield 1.4% [0.018 mg]

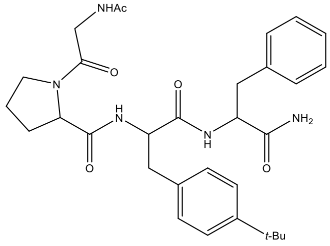

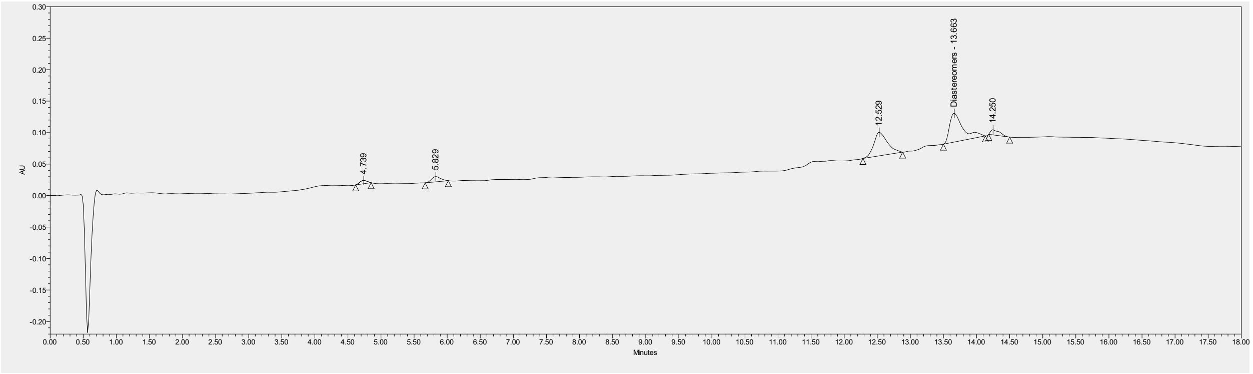

	Name	Retention Time	Area	% Area
1		4.739	40503	2.94
2		5.829	80196	5.82
3		12.529	534366	38.80
4	Diastereomers	13.663	642632	46.66
5		14.250	79493	5.77

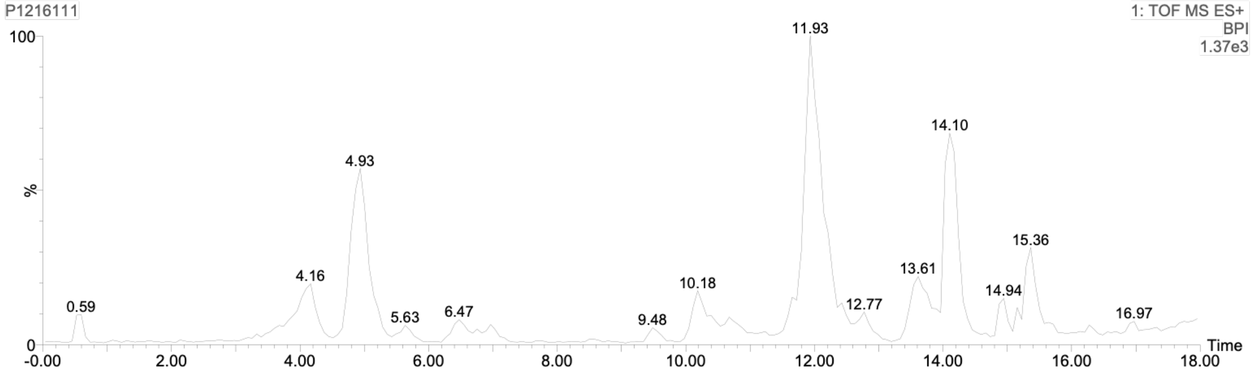

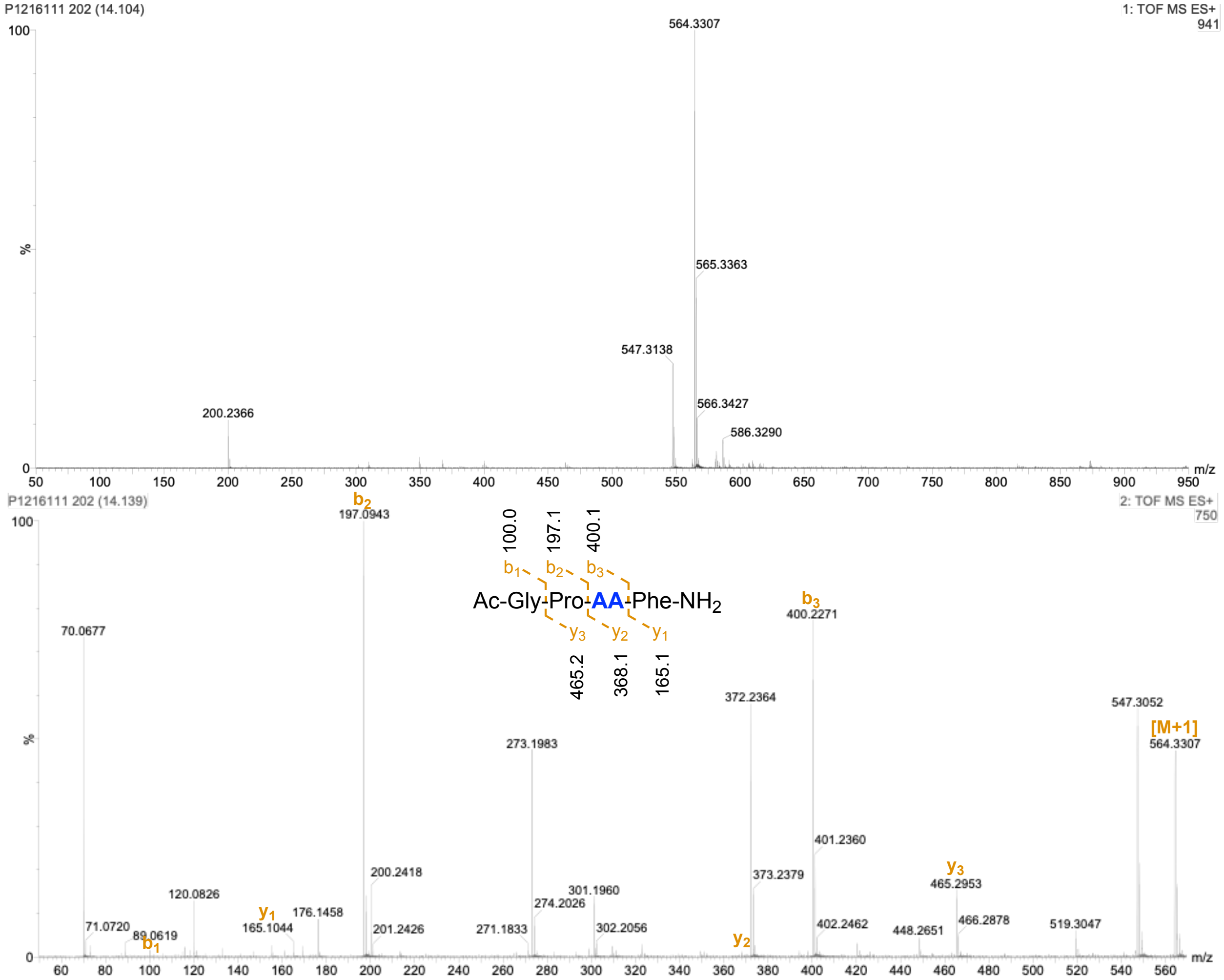
P1216111 202 (14.104)
1: TOF MS ES+
941
564.3307
565.3363
547.3138
200.2366
566.3427
586.3290
%
m/z
P1216111 202 (14.139)
2: TOF MS ES+
750
b2
197.0943
100.0
197.1
400.1
b1
b2
b3
Ac-Gly-Pro-AA-Phe-NH2
y3
y2
y1
465.2
368.1
165.1
b3
400.2271
70.0677
372.2364
547.3052
[M+1]
564.3307
273.1983
401.2360
200.2418
120.0826
301.1960
373.2379
y3
465.2953
y1
176.1458
274.2026
71.0720
165.1044
89.0619
b1
201.2426
271.1833
302.2056
402.2462
448.2651
466.2878
519.3047
y2

5E': MW = 553.7, Purity = 78.7%, Yield = 17.7% [0.23 mg]

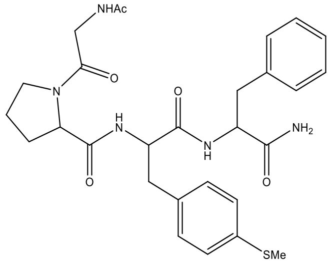

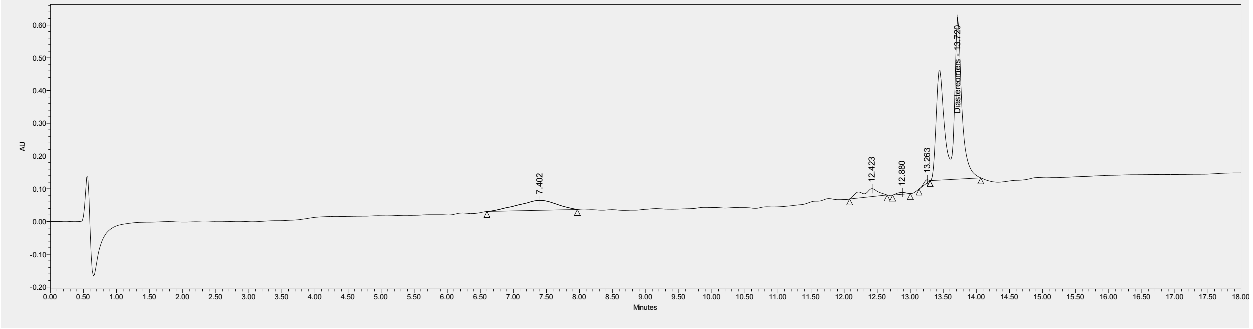

	Name	Retention Time	Area	% Area
1		7.402	1243595	14.86
2		12.423	436623	5.22
3		12.880	55570	0.66
4		13.263	50532	0.60
5	Diastereomers	13.720	6581170	78.65

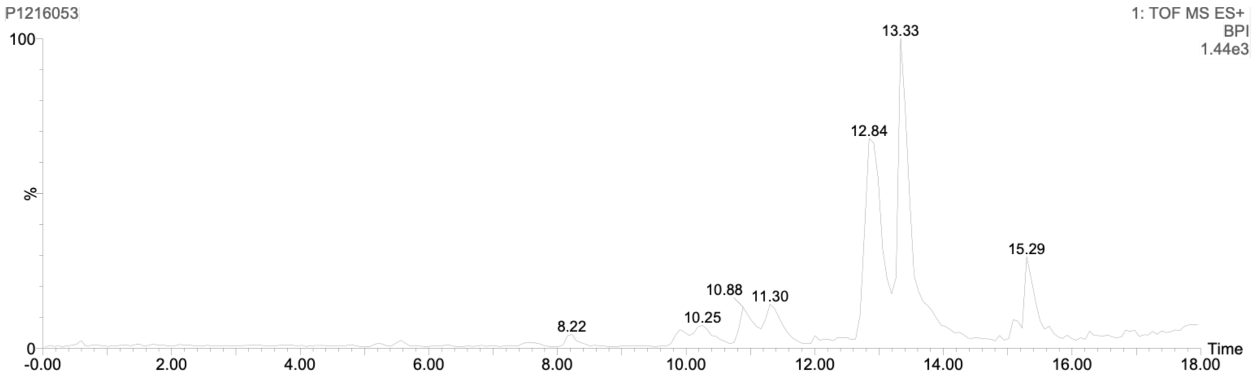

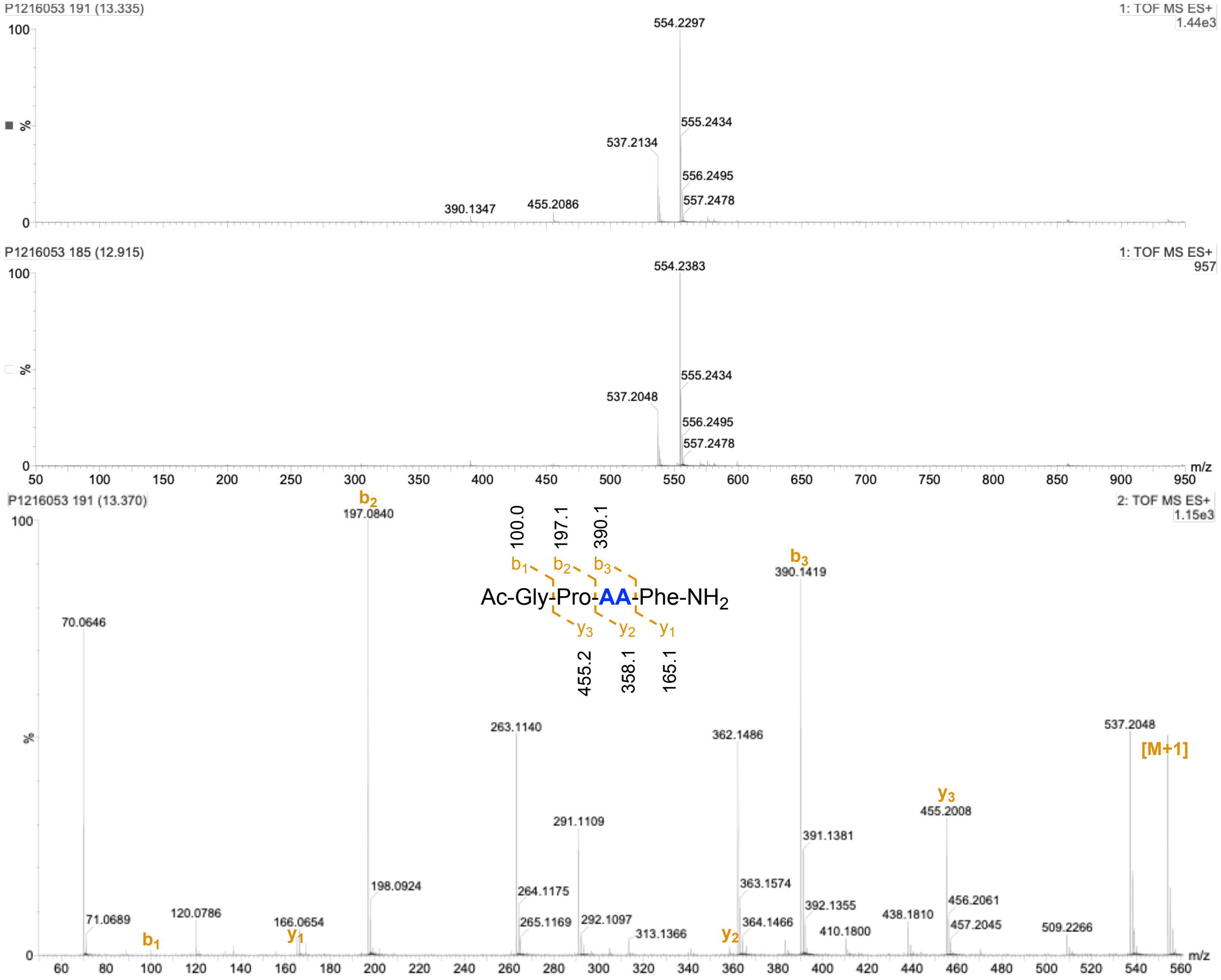
P1216053 191 (13.335)
1: TOF MS ES+
1.44e3
554.2297
555.2434
537.2134
556.2495
557.2478
390.1347
455.2086
P1216053 185 (12.915)
1: TOF MS ES+
957
554.2383
555.2434
537.2048
556.2495
557.2478
m/z
P1216053 191 (13.370)
2: TOF MS ES+
1.15e3
b2
197.0840
100.0
197.1
390.1
b1
b2
b3
Ac-Gly-Pro-AA-Phe-NH2
y3
y2
y1
455.2
358.1
165.1
b3
390.1419
70.0646
263.1140
362.1486
537.2048
[M+1]
y3
455.2008
291.1109
391.1381
198.0924
264.1175
363.1574
456.2061
71.0689
120.0786
166.0654
265.1169
292.1097
364.1466
392.1355
438.1810
457.2045
509.2266
b1
y1
313.1366
y2
410.1800
m/z

6E’: MW = 537.6, Purity = 84.9%, Yield = 22.4% [0.28 mg]

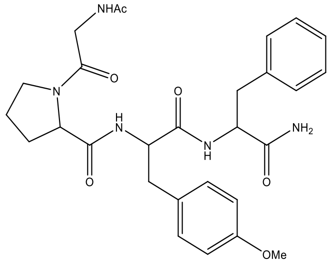

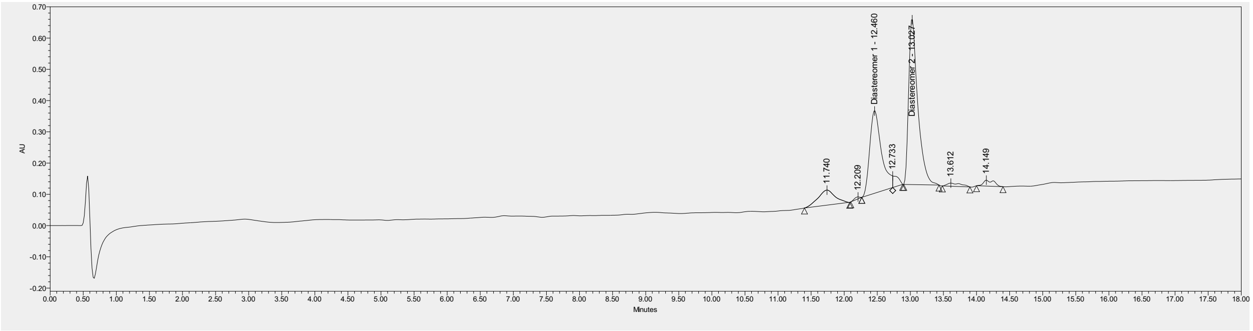

	Name	Retention Time	Area	% Area
1		11.740	862232	8.79
2		12.209	39611	0.40
3	Diastereomer 1	12.460	3251919	33.14
4		12.733	214576	2.19
5	Diastereomer 2	13.027	5079842	51.76
6		13.612	153208	1.56
7		14.149	212322	2.16

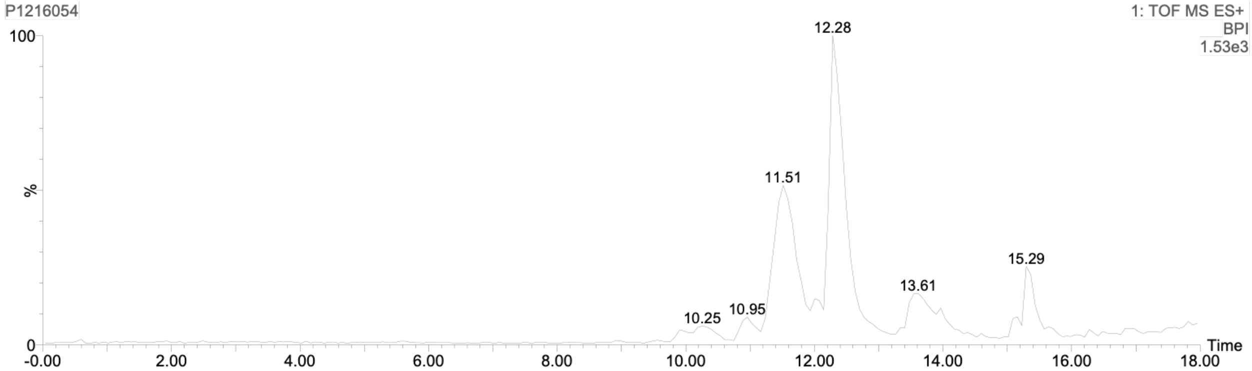

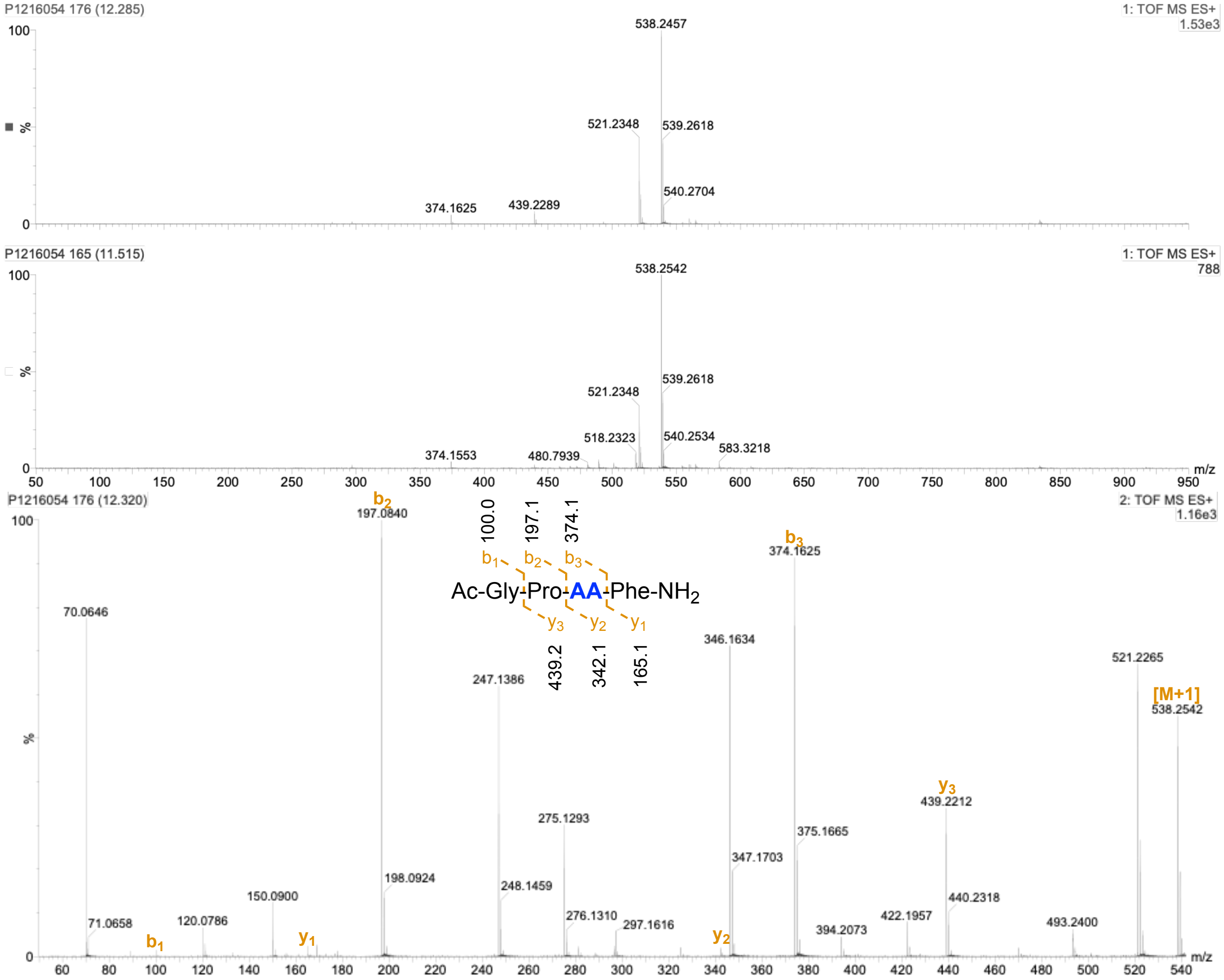
P1216054 176 (12.285)
1: TOF MS ES+
1.53e3
538.2457
521.2348
539.2618
540.2704
374.1625
439.2289
P1216054 165 (11.515)
1: TOF MS ES+
788
538.2542
539.2618
521.2348
518.2323
540.2534
583.3218
374.1553
480.7939
m/z
P1216054 176 (12.320)
2: TOF MS ES+
1.16e3
b2
197.0840
100.0
197.1
374.1
Ac-Gly-Pro-AA-Phe-NH2
439.2
342.1
165.1
b3
374.1625
70.0646
346.1634
247.1386
521.2265
[M+1]
538.2542
y3
439.2212
275.1293
375.1665
347.1703
198.0924
248.1459
150.0900
440.2318
422.1957
276.1310
297.1616
394.2073
493.2400
71.0658
120.0786
b1
y1
y2
m/z

7E’: MW = 537.6, Purity = 79.6%, Yield = 14.2% [0.18 mg]

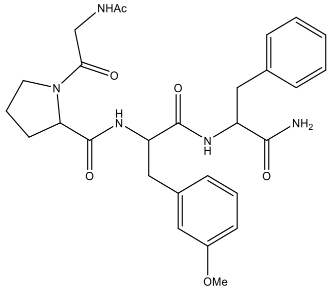

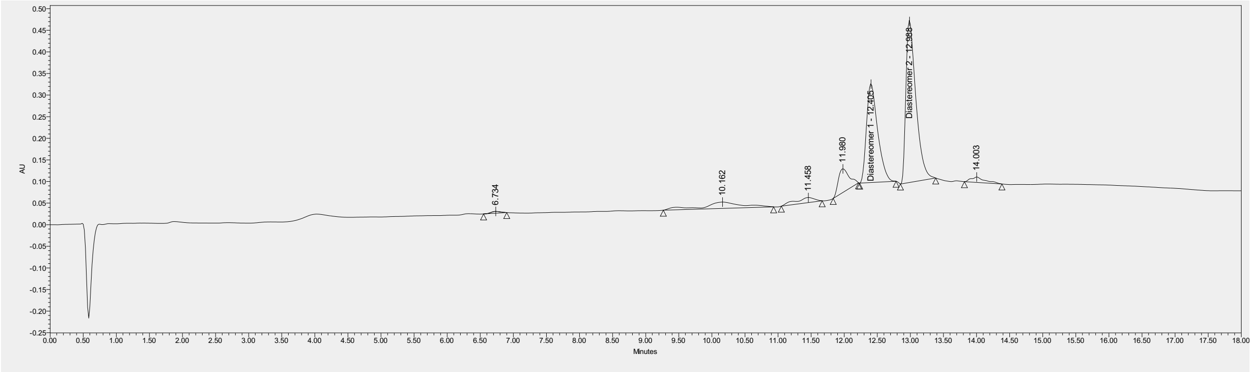

	Name	Retention Time	Area	% Area
1		6.734	41449	0.50
2		10.162	598228	7.19
3		11.458	245398	2.95
4		11.980	624962	7.51
5	Diastereomer 1	12.405	2687783	32.32
6	Diastereomer 2	12.988	3930732	47.26
7		14.003	188078	2.26

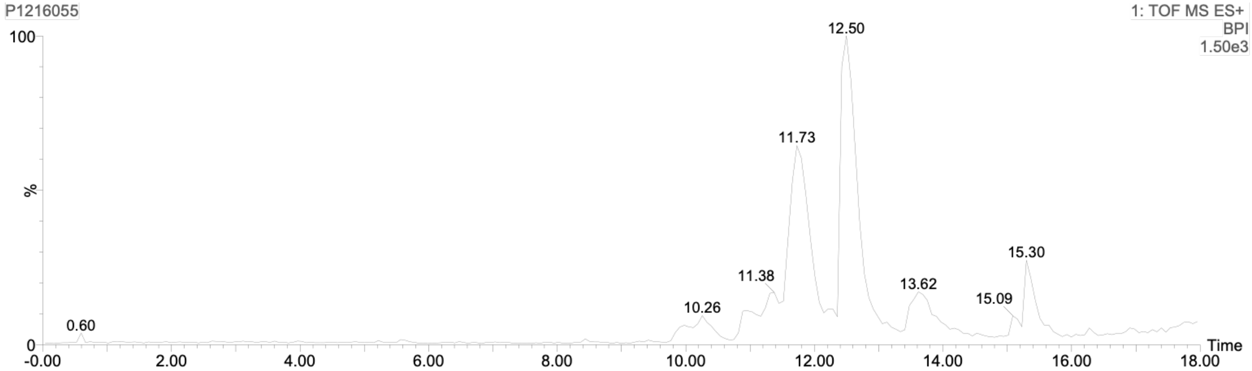

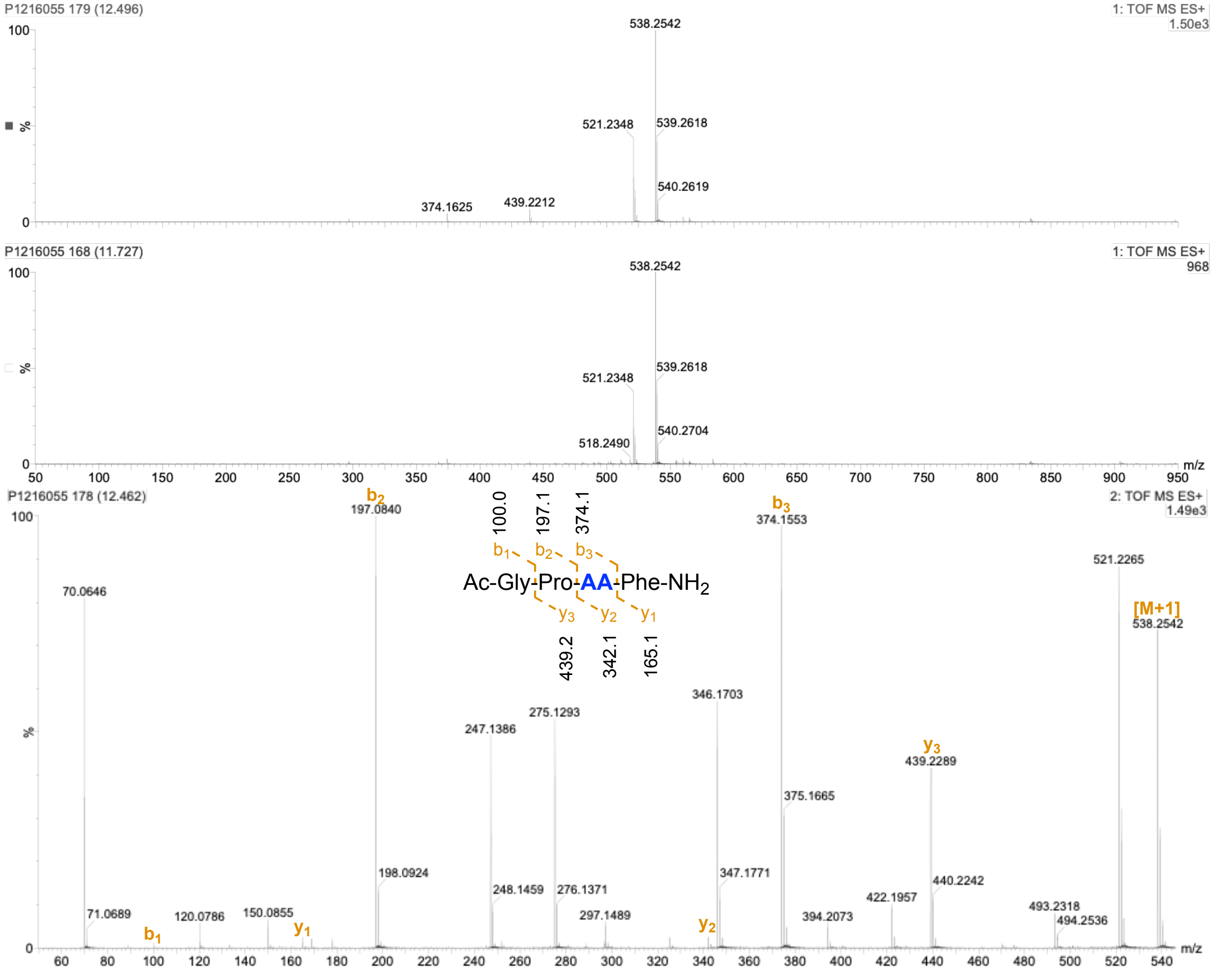
P1216055 179 (12.496)
1: TOF MS ES+
1.50e3
538.2542
521.2348
539.2618
540.2619
374.1625
439.2212
P1216055 168 (11.727)
1: TOF MS ES+
968
538.2542
521.2348
539.2618
518.2490
540.2704
m/z
P1216055 178 (12.462)
2: TOF MS ES+
1.49e3
Ac-Gly-Pro-AA-Phe-NH2
100.0
197.1
374.1
439.2
342.1
165.1
b1
b2
b3
y1
y2
y3
197.0840
374.1553
521.2265
[M+1]
538.2542
70.0646
247.1386
275.1293
346.1703
439.2289
375.1665
198.0924
248.1459
276.1371
347.1771
440.2242
422.1957
493.2318
494.2536
71.0689
120.0786
150.0855
297.1489
394.2073
m/z

8E’: MW = 537.6, Purity = 79.5%, Yield = 17.7% [0.22 mg]

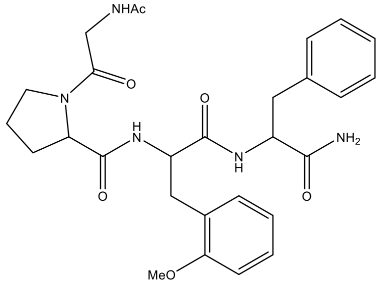

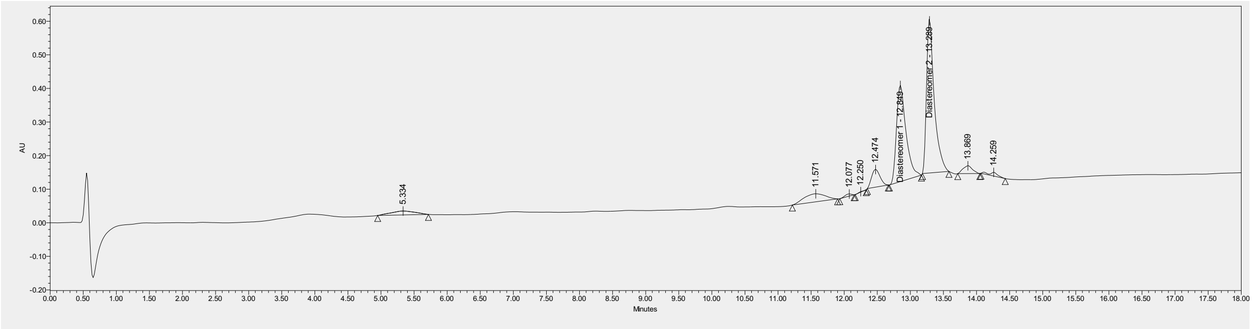

	Name	Retention Time	Area	% Area
1		5.334	297174	3.59
2		11.571	545449	6.59
3		12.077	46009	0.56
4		12.250	6909	0.08
5		12.474	468999	5.66
6	Diastereomer 1	12.849	2873822	34.70
7	Diastereomer 2	13.289	3710645	44.80
8		13.869	231876	2.80
9		14.259	102051	1.23

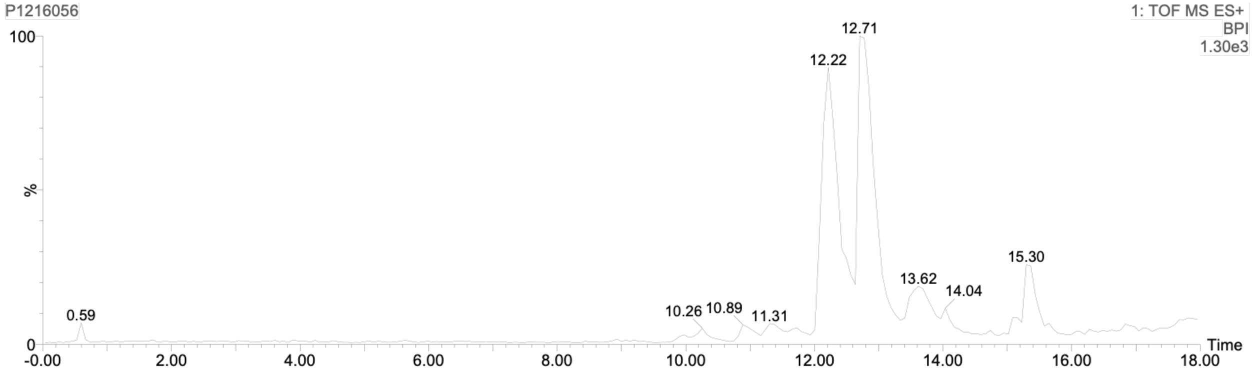

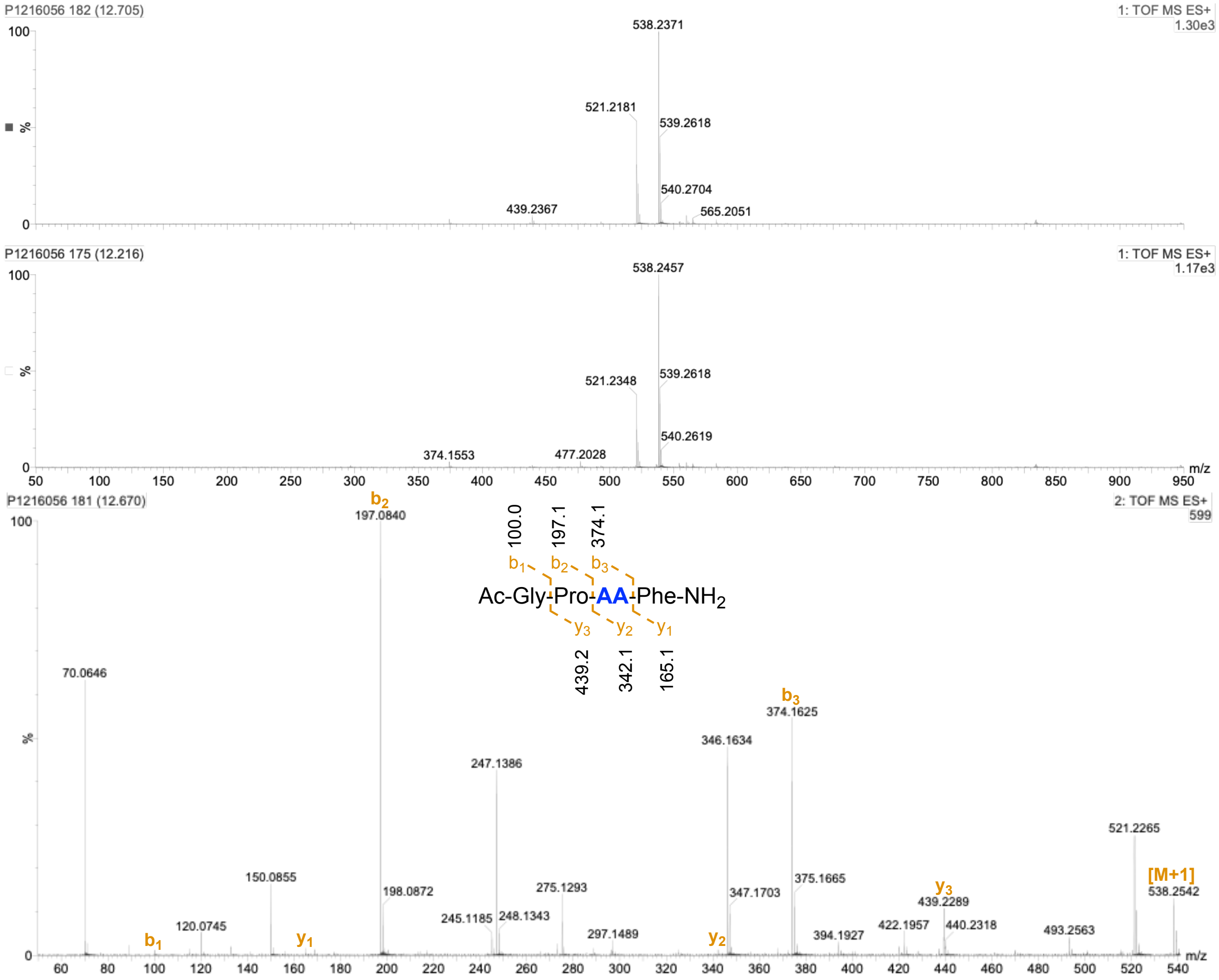
P1216056 182 (12.705)
1: TOF MS ES+
1.30e3
538.2371
521.2181
539.2618
540.2704
439.2367
565.2051
P1216056 175 (12.216)
1: TOF MS ES+
1.17e3
538.2457
521.2348
539.2618
540.2619
374.1553
477.2028
m/z
P1216056 181 (12.670)
2: TOF MS ES+
599
Ac-Gly-Pro-AA-Phe-NH2
b1 b2 b3
100.0 197.1 374.1
y3 y2 y1
439.2 342.1 165.1
b2 197.0840
70.0646
b3 374.1625
346.1634
247.1386
521.2265
[M+1] 538.2542
150.0855
198.0872
275.1293
347.1703
375.1665
y3 439.2289
120.0745
245.1185
248.1343
297.1489
394.1927
422.1957
440.2318
493.2563
b1
y1
y2
m/z
%

9E': MW = 567.6, Purity = 79.2%, Yield = 22.4% [0.30 mg]

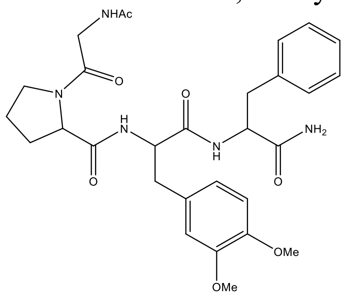

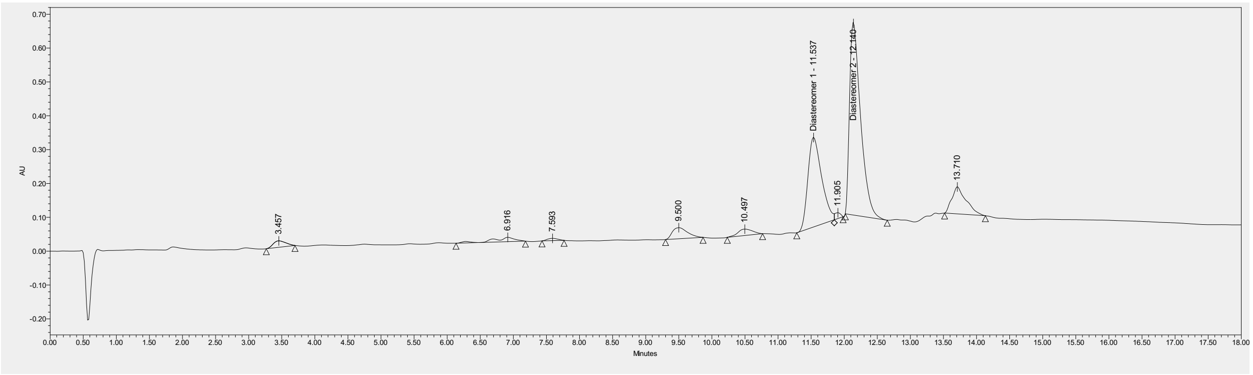

	Name	Retention Time	Area	% Area
1		3.457	252982	1.92
2		6.916	286735	2.18
3		7.593	74817	0.57
4		9.500	525492	4.00
5		10.497	289317	2.20
6	Diastereomer 1	11.537	3814306	29.01
7		11.905	104615	0.80
8	Diastereomer 2	12.140	6598771	50.19
9		13.710	1199691	9.13

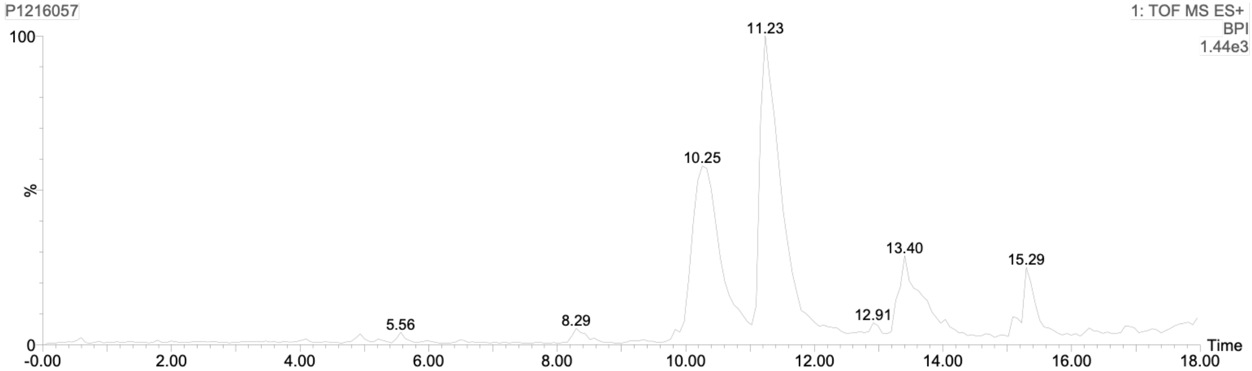

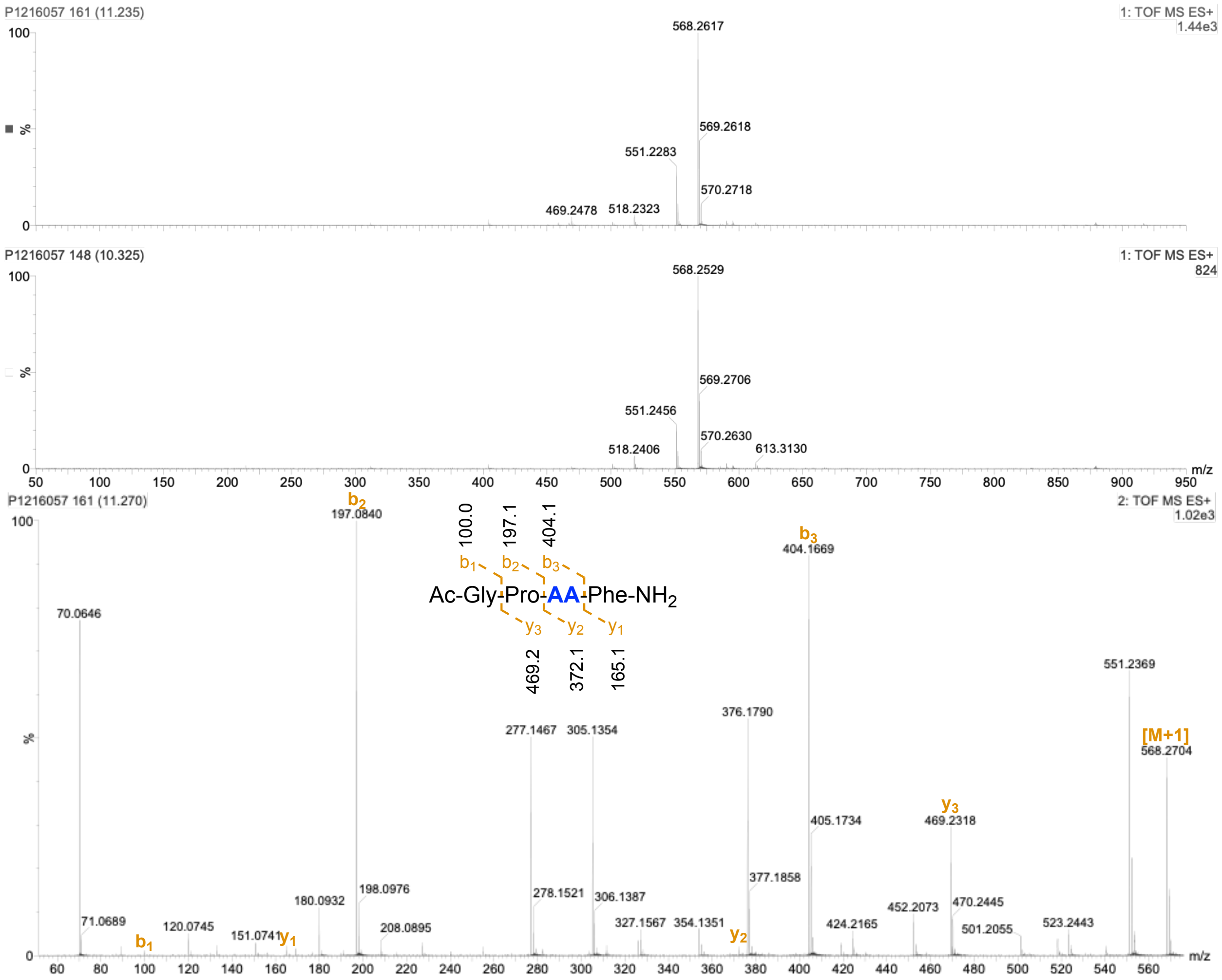
P1216057 161 (11.235)
1: TOF MS ES+
1.44e3
568.2617
569.2618
551.2283
570.2718
469.2478
518.2323
P1216057 148 (10.325)
1: TOF MS ES+
824
568.2529
569.2706
551.2456
570.2630
613.3130
518.2406
m/z
P1216057 161 (11.270)
2: TOF MS ES+
1.02e3
b2
197.0840
100.0
197.1
404.1
Ac-Gly-Pro-AA-Phe-NH2
469.2
372.1
165.1
b3
404.1669
70.0646
551.2369
376.1790
277.1467
305.1354
[M+1]
568.2704
y3
469.2318
405.1734
377.1858
278.1521
306.1387
198.0976
180.0932
452.2073
470.2445
71.0689
120.0745
151.0741
327.1567
354.1351
424.2165
501.2055
523.2443
208.0895
b1
y1
y2
m/z

10E': MW = 567.6, Purity = 88.7%, Yield = 23.3% [0.31 mg]

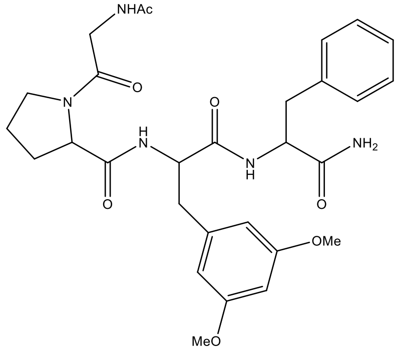

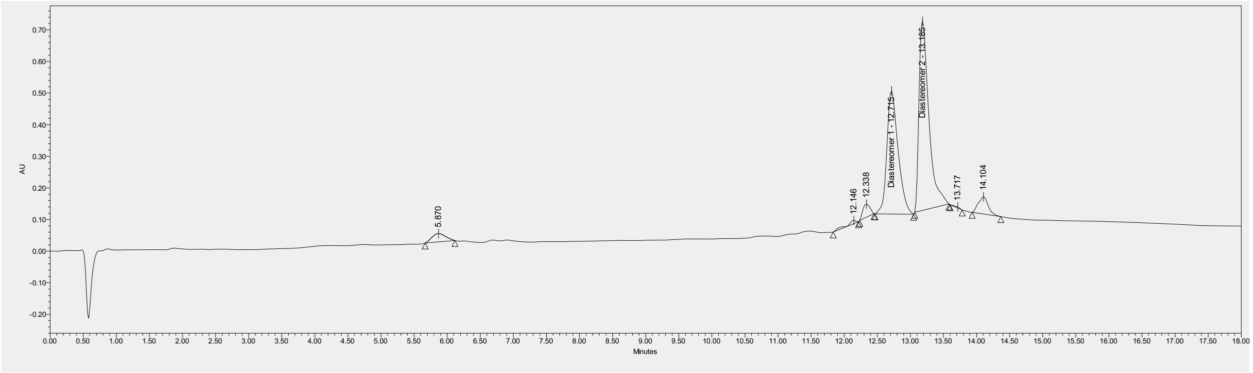

	Name	Retention Time	Area	% Area
1		5.870	380960	3.12
2		12.146	102226	0.84
3		12.338	313936	2.57
4	Diastereomer 1	12.715	4598051	37.71
5	Diastereomer 2	13.185	6217556	50.99
6		13.717	20466	0.17
7		14.104	560347	4.60

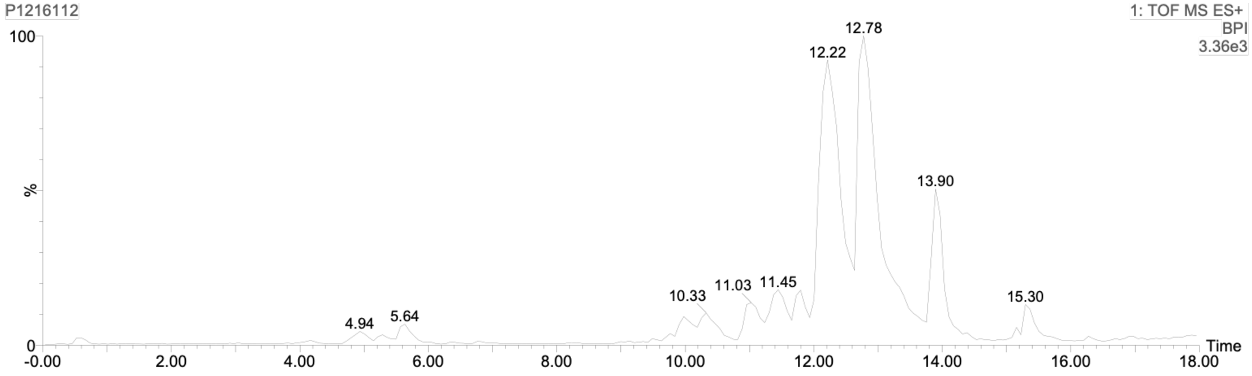

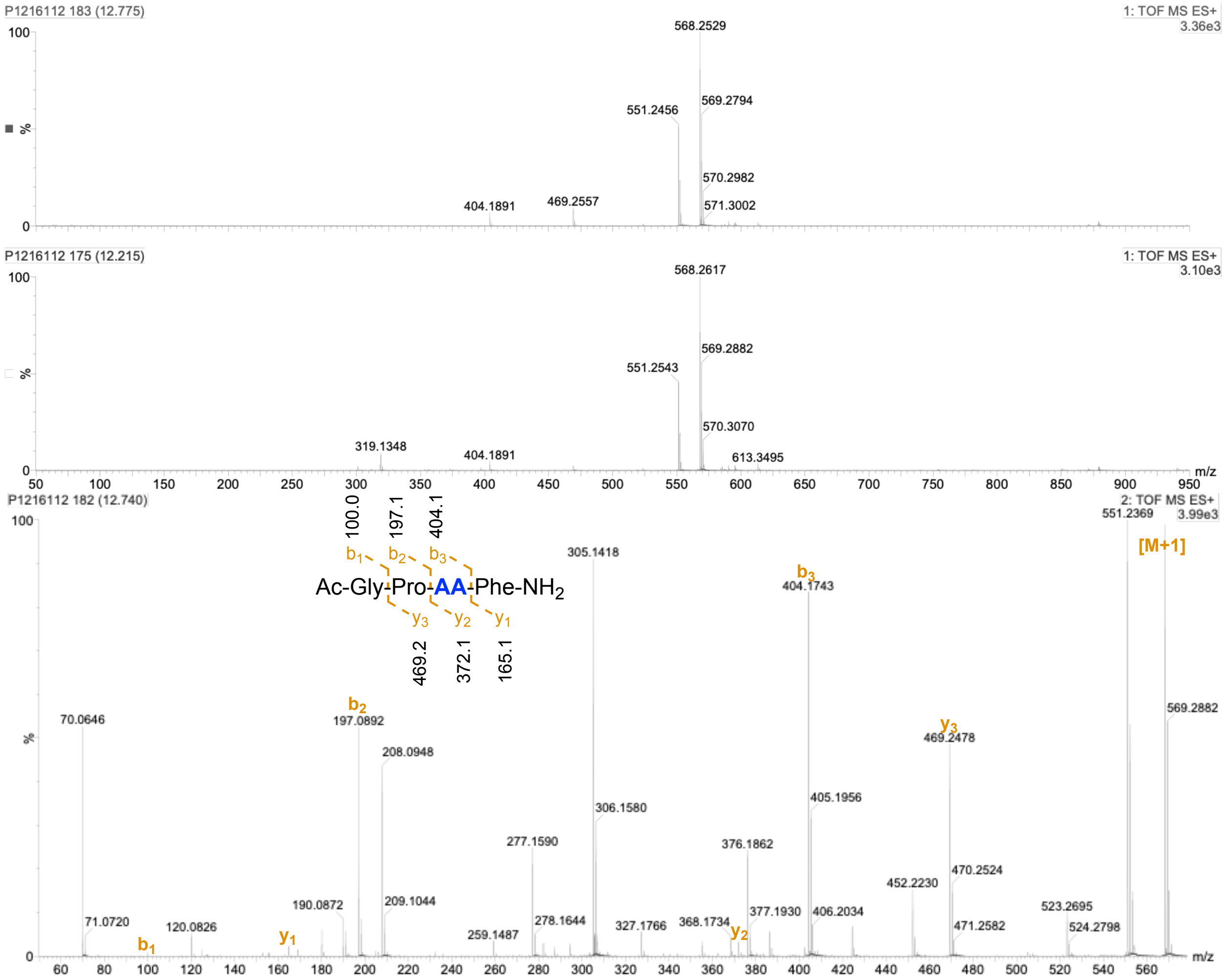
P1216112 183 (12.775)
1: TOF MS ES+
3.36e3
568.2529
551.2456
569.2794
570.2982
571.3002
404.1891
469.2557
P1216112 175 (12.215)
1: TOF MS ES+
3.10e3
568.2617
551.2543
569.2882
570.3070
613.3495
319.1348
404.1891
m/z
P1216112 182 (12.740)
2: TOF MS ES+
3.99e3
551.2369
[M+1]
Ac-Gly-Pro-AA-Phe-NH2
100.0
197.1
404.1
469.2
372.1
165.1
305.1418
404.1743
70.0646
197.0892
208.0948
469.2478
569.2882
306.1580
405.1956
277.1590
376.1862
452.2230
470.2524
190.0872
209.1044
71.0720
120.0826
259.1487
278.1644
327.1766
368.1734
377.1930
406.2034
471.2582
523.2695
524.2798
m/z

11E’: MW = 567.6, Purity = 65.2%, Yield = 6.5% [0.086 mg]

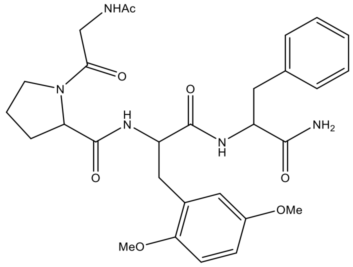

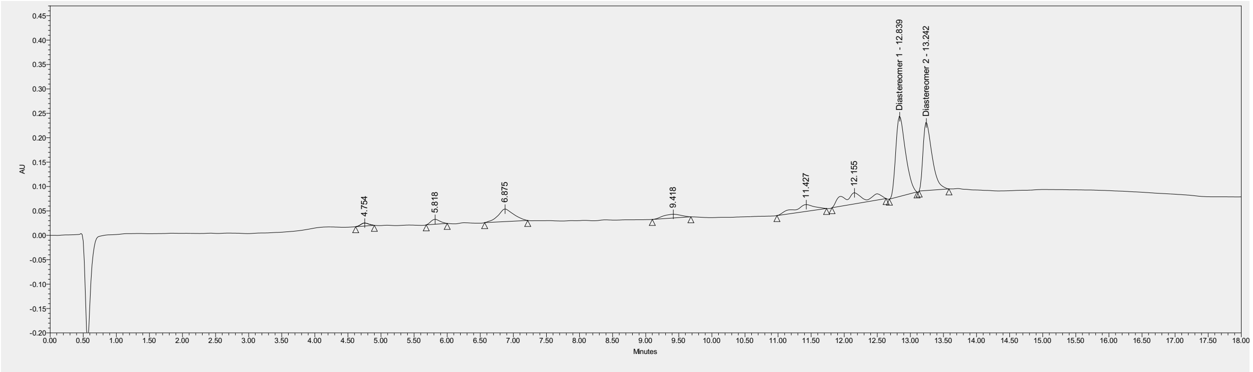

	Name	Retention Time	Area	% Area
1		4.754	51671	1.12
2		5.818	96192	2.08
3		6.875	425233	9.21
4		9.418	146780	3.18
5		11.427	310613	6.73
6		12.155	576944	12.50
7	Diastereomer 1	12.839	1690409	36.61
8	Diastereomer 2	13.242	1318978	28.57

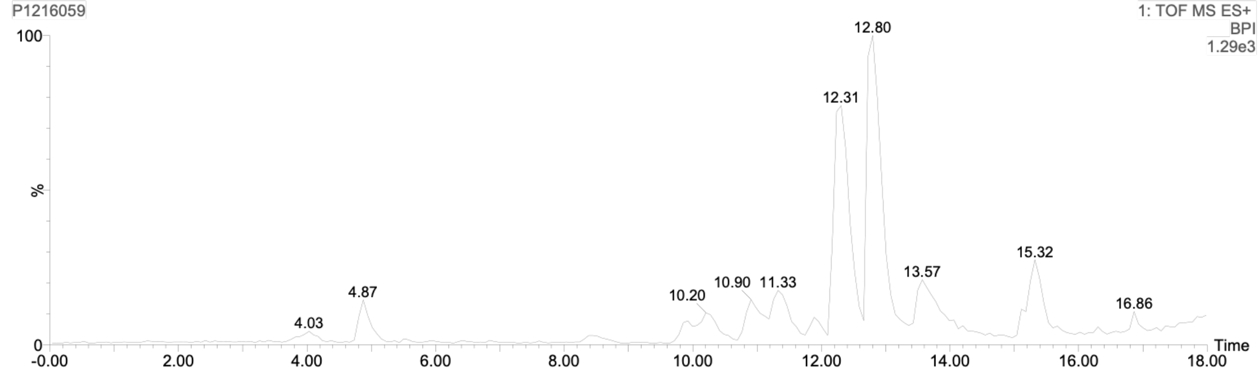

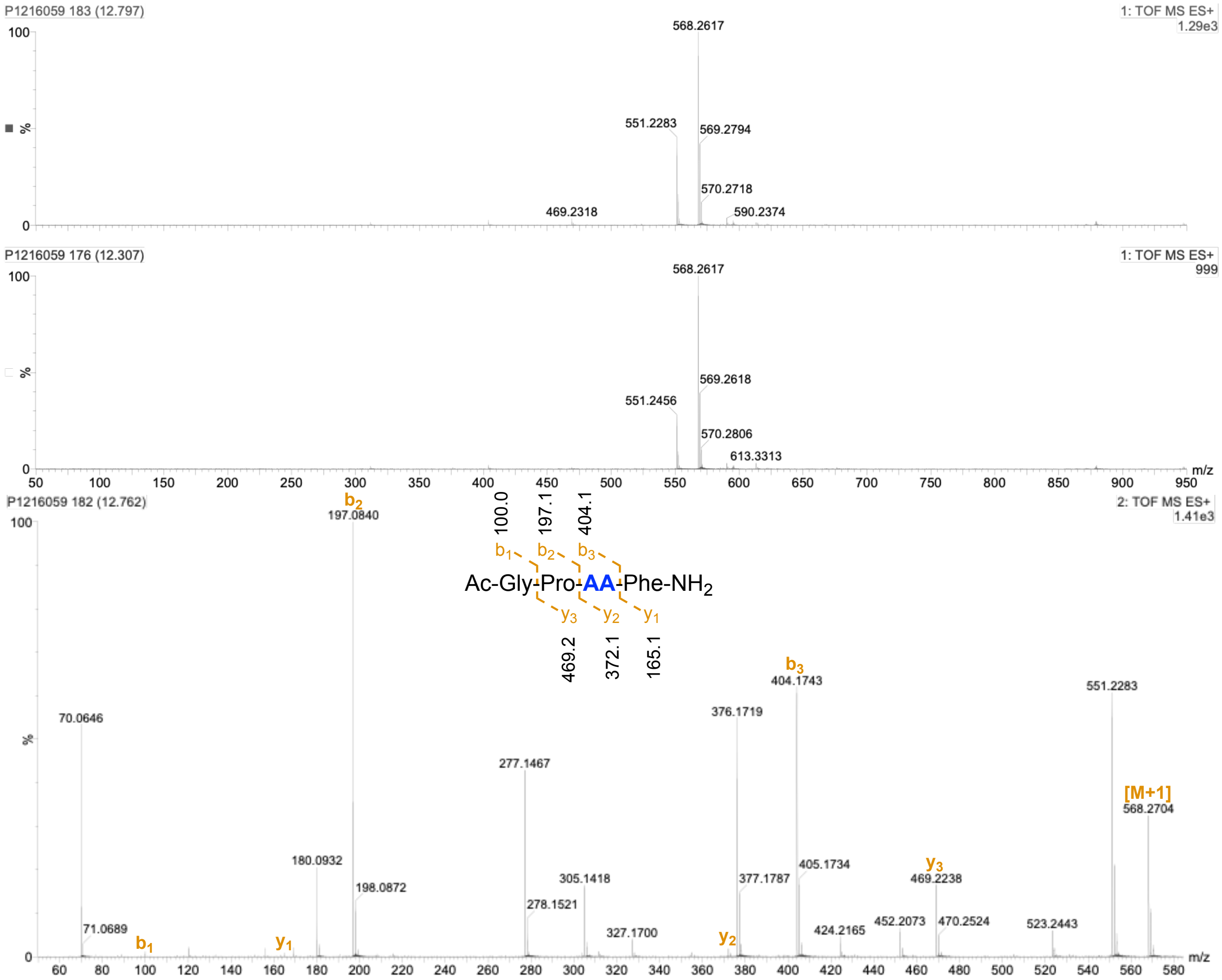

P1216059 183 (12.797)
1: TOF MS ES+
1.29e3
568.2617
551.2283
569.2794
570.2718
469.2318
590.2374
P1216059 176 (12.307)
1: TOF MS ES+
999
568.2617
569.2618
551.2456
570.2806
613.3313
m/z
P1216059 182 (12.762)
2: TOF MS ES+
1.41e3
Ac-Gly-Pro-AA-Phe-NH2
b1 b2 b3
y3 y2 y1
100.0
197.1
404.1
469.2
372.1
165.1
b2
197.0840
b3
404.1743
551.2283
70.0646
376.1719
277.1467
[M+1]
568.2704
180.0932
198.0872
305.1418
405.1734
y3
469.2238
278.1521
377.1787
71.0689
327.1700
424.2165
452.2073
470.2524
523.2443
b1
y1
y2

12E’: MW = 621.7, Purity = 59.1%, Yield = 6.0% [0.087 mg]

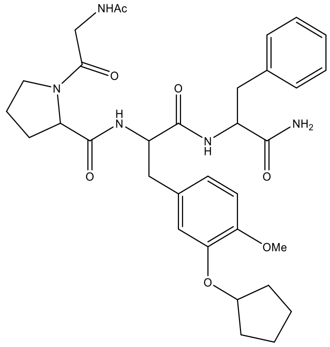

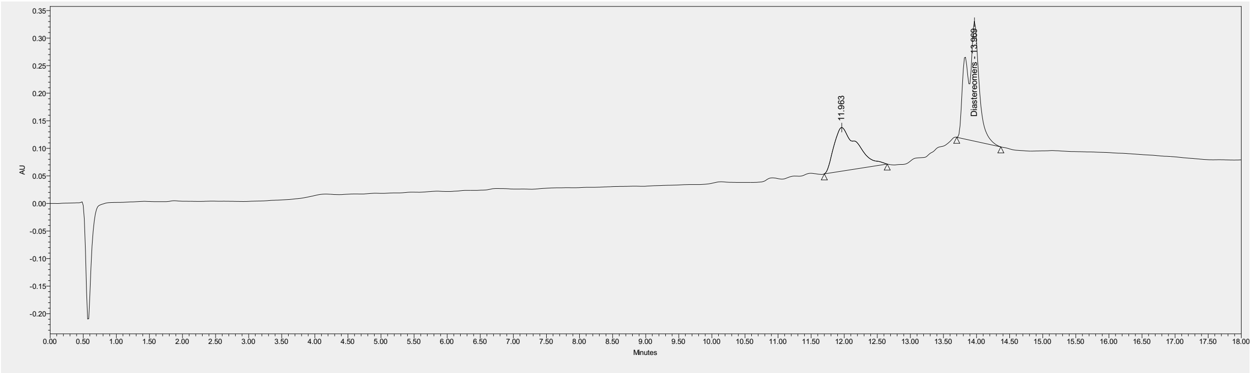

	Name	Retention Time	Area	% Area
1		11.963	1935350	40.90
2	Diastereomers	13.969	2796381	59.10

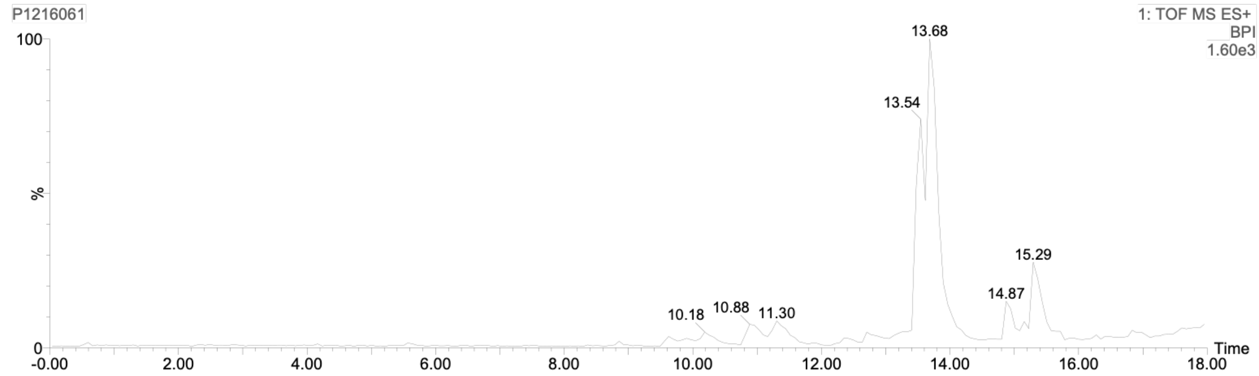

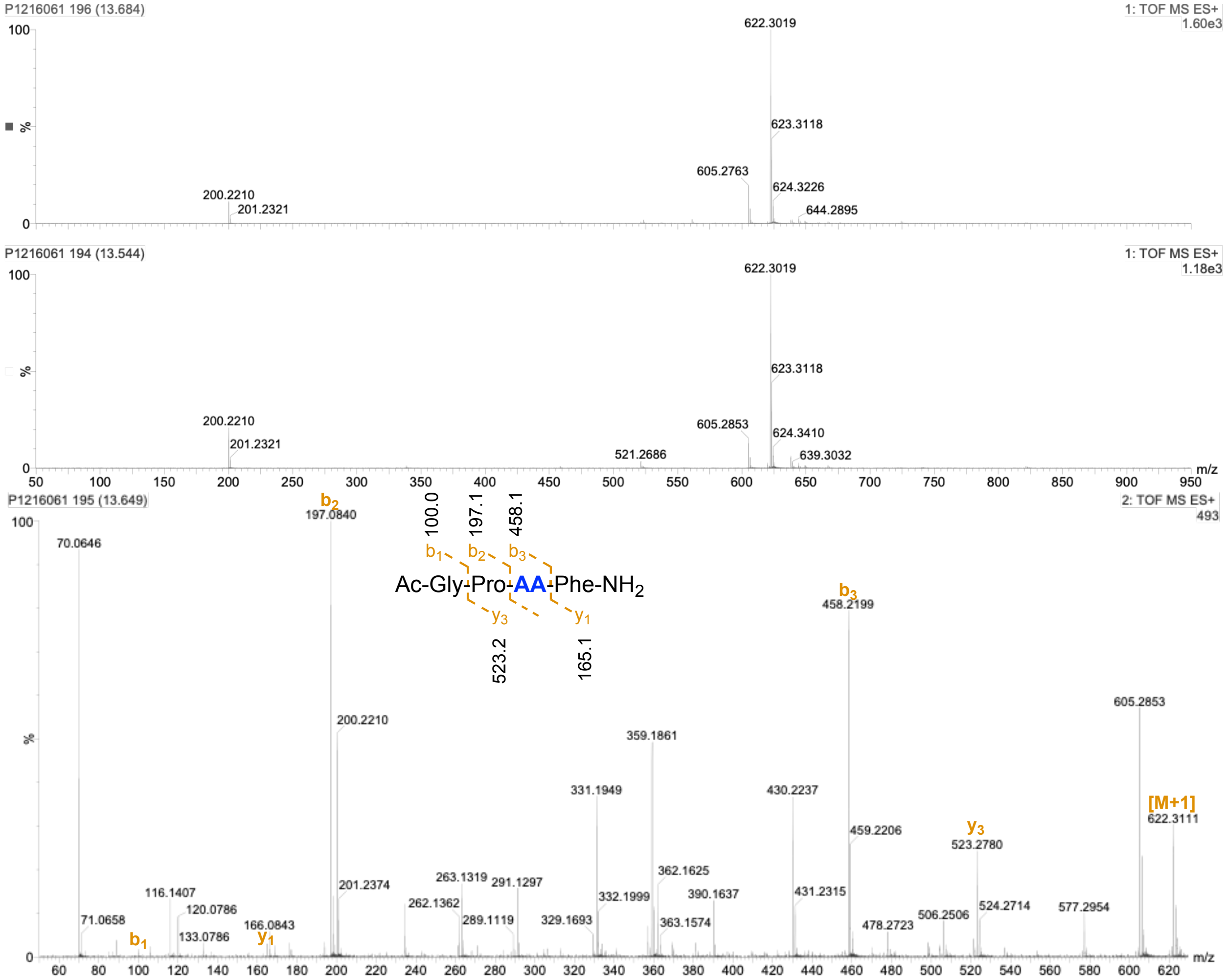

P1216061 196 (13.684)
1: TOF MS ES+
1.60e3
622.3019
623.3118
605.2763
624.3226
644.2895
200.2210
201.2321
P1216061 194 (13.544)
1: TOF MS ES+
1.18e3
622.3019
623.3118
200.2210
201.2321
605.2853
624.3410
521.2686
639.3032
m/z
P1216061 195 (13.649)
2: TOF MS ES+
493
b2
197.0840
70.0646
100.0
197.1
458.1
b1
b2
b3
Ac-Gly-Pro-AA-Phe-NH2
y3
y1
523.2
165.1
b3
458.2199
605.2853
200.2210
359.1861
331.1949
430.2237
[M+1]
622.3111
y3
523.2780
459.2206
116.1407
120.0786
71.0658
b1
133.0786
166.0843
y1
201.2374
263.1319
262.1362
291.1297
289.1119
329.1693
332.1999
362.1625
390.1637
363.1574
431.2315
478.2723
506.2506
524.2714
577.2954
m/z

1F’: MW = 555.6, Purity = 76.0%, Yield = 14.6% [0.19 mg]

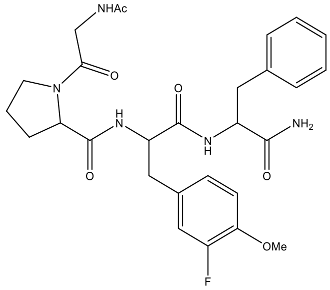

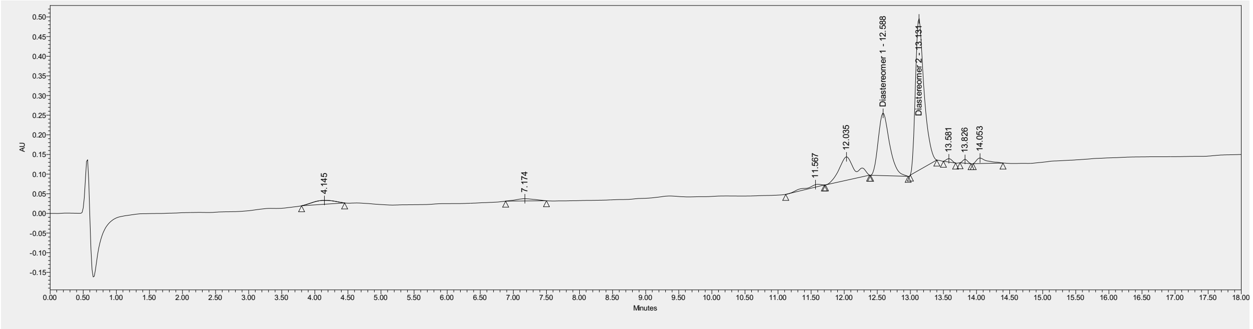

	Name	Retention Time	Area	% Area
1		4.145	230687	3.24
2		7.174	114243	1.60
3		11.567	112673	1.58
4		12.035	987867	13.87
5	Diastereomer 1	12.588	1899373	26.67
6	Diastereomer 2	13.131	3519544	49.42
7		13.581	50403	0.71
8		13.826	49737	0.70
9		14.053	157003	2.20

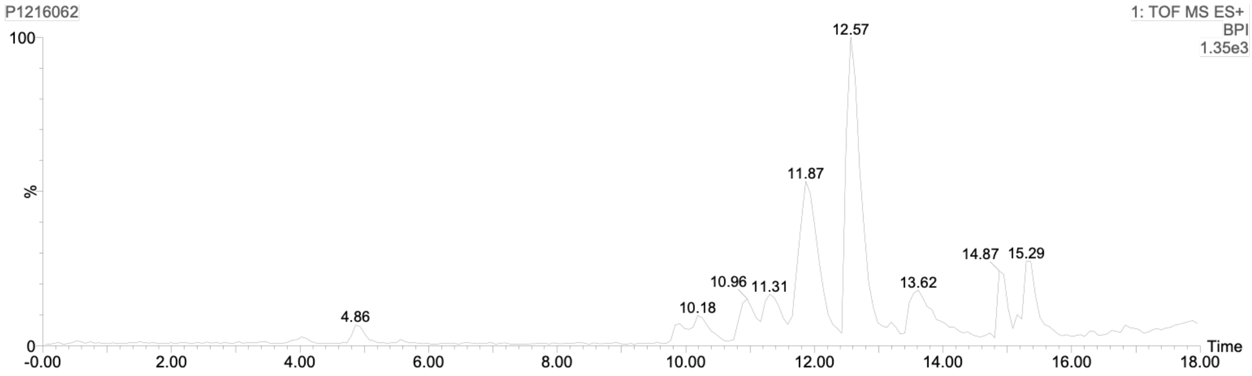

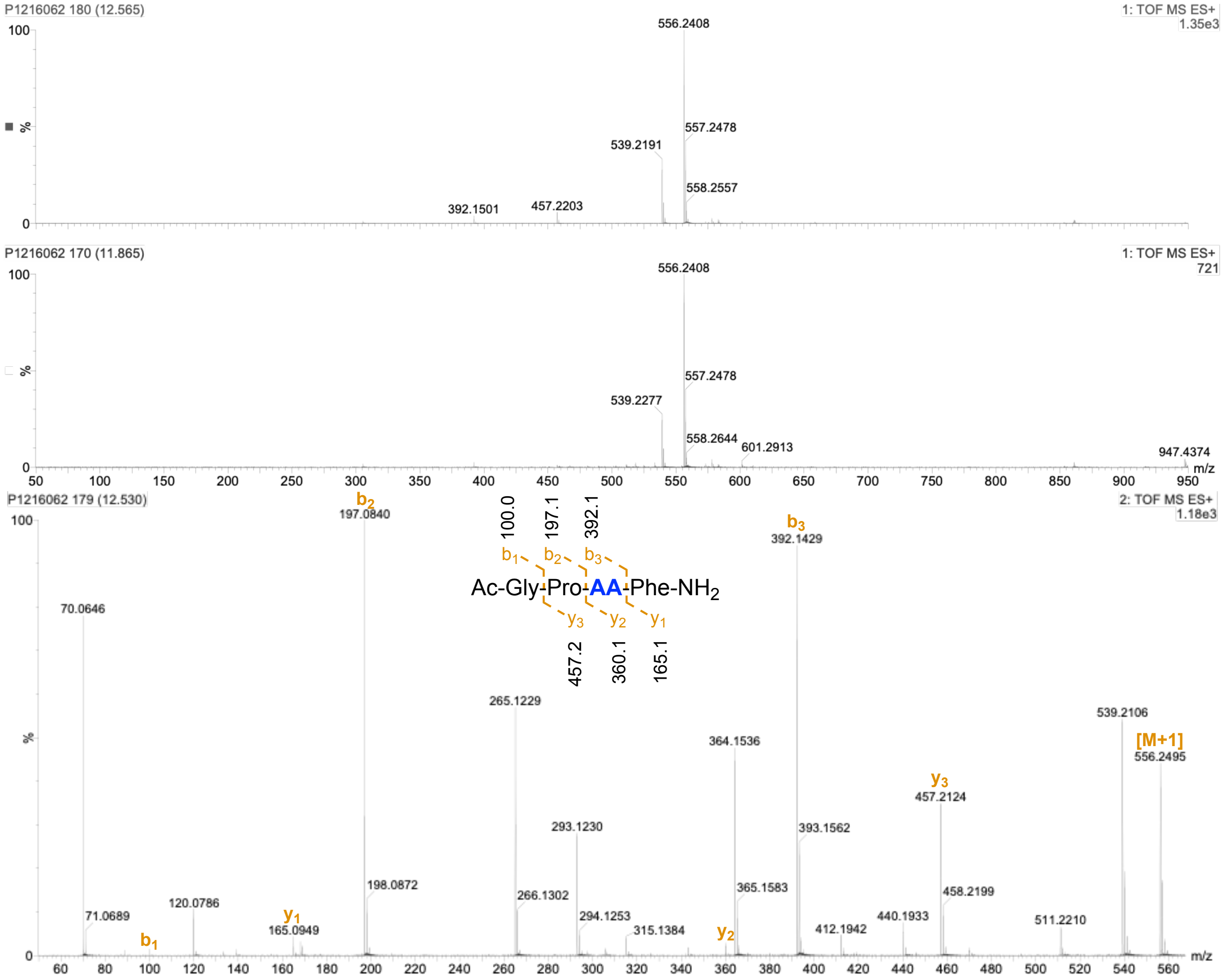

P1216062 180 (12.565)
1: TOF MS ES+
1.35e3
556.2408
557.2478
539.2191
558.2557
392.1501
457.2203
P1216062 170 (11.865)
1: TOF MS ES+
721
556.2408
557.2478
539.2277
558.2644
601.2913
947.4374
m/z
P1216062 179 (12.530)
2: TOF MS ES+
1.18e3
b2
197.0840
Ac-Gly-Pro-AA-Phe-NH2
100.0
197.1
392.1
457.2
360.1
165.1
b3
392.1429
70.0646
265.1229
539.2106
[M+1]
556.2495
364.1536
y3
457.2124
293.1230
393.1562
198.0872
266.1302
365.1583
458.2199
120.0786
71.0689
y1
165.0949
294.1253
315.1384
440.1933
412.1942
511.2210
b1
y2
m/z
%

2F’: MW = 613.7, Purity = 27.0%, Yield = 0.70% [0.010 mg]

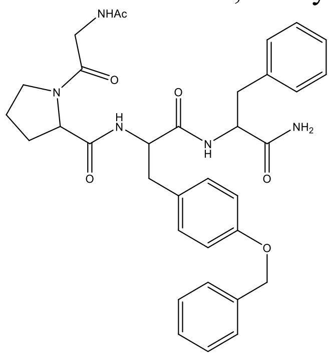

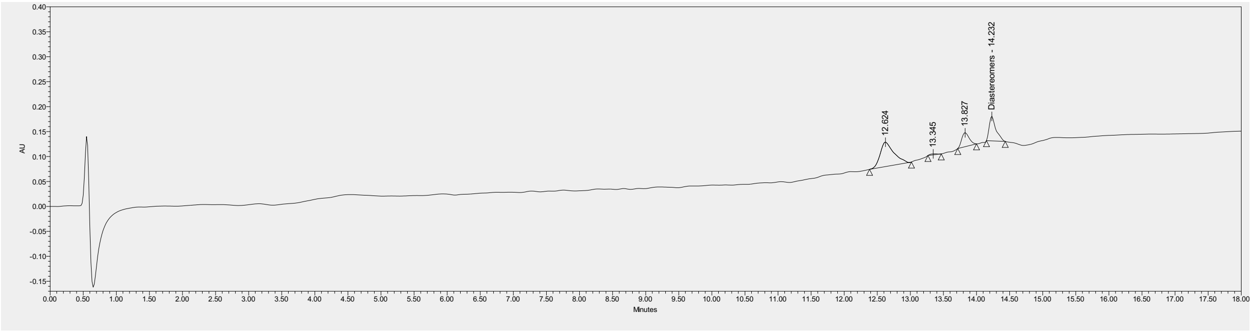

	Name	Retention Time	Area	% Area
1		12.624	701605	54.72
2		13.345	14100	1.10
3		13.827	220174	17.17
4	Diastereomers	14.232	346206	27.00

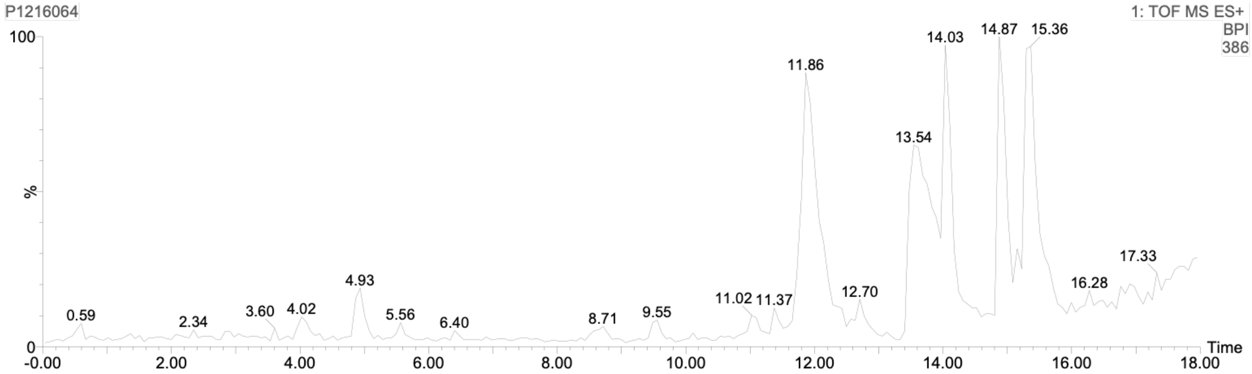

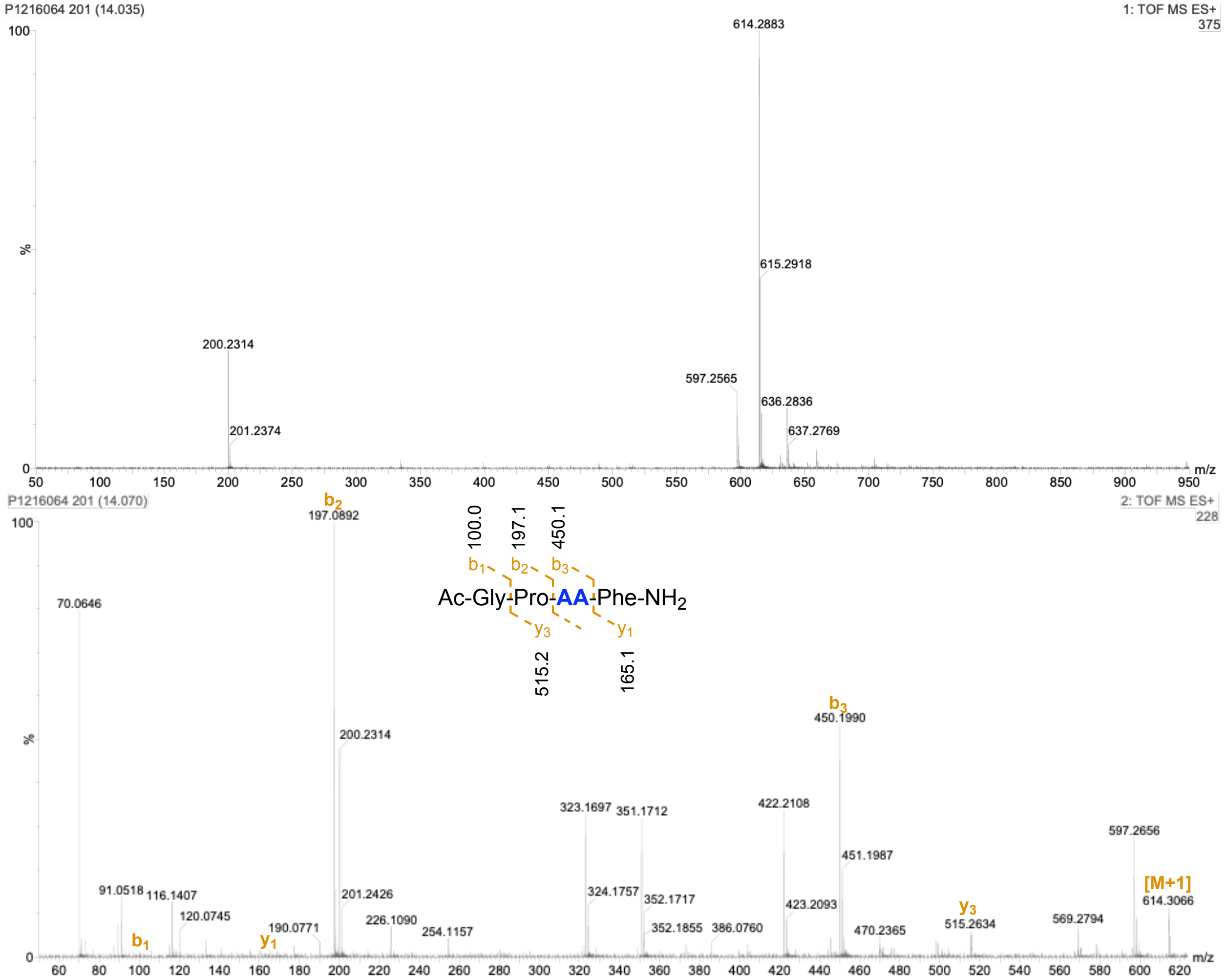
P1216064 201 (14.035)
1: TOF MS ES+
375
614.2883
615.2918
200.2314
597.2565
636.2836
201.2374
637.2769
m/z
%
P1216064 201 (14.070)
2: TOF MS ES+
228
b2
197.0892
100.0
197.1
450.1
b1 b2 b3
Ac-Gly-Pro-AA-Phe-NH2
y3 y1
515.2
165.1
70.0646
200.2314
b3
450.1990
323.1697 351.1712
422.2108
597.2656
451.1987
91.0518 116.1407
324.1757
352.1717
201.2426
[M+1]
614.3066
120.0745
423.2093
y3
190.0771
226.1090
352.1855
386.0760
515.2634
569.2794
254.1157
470.2365
b1
y1

3F’: MW = 597.7, Purity = 81.7%, Yield = 44.1% [0.61 mg]

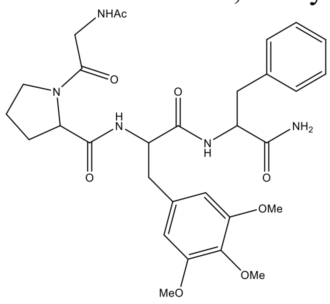

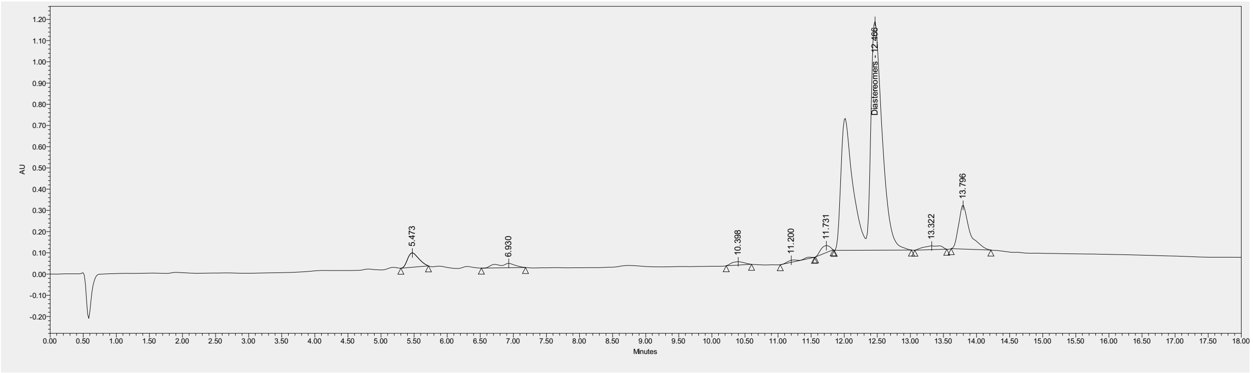

	Name	Retention Time	Area	% Area
1		5.473	796739	3.17
2		6.930	406399	1.62
3		10.398	210541	0.84
4		11.200	137232	0.55
5		11.731	326916	1.30
6	Diastereomers	12.466	20501442	81.69
7		13.322	341290	1.36
8		13.796	2375528	9.47

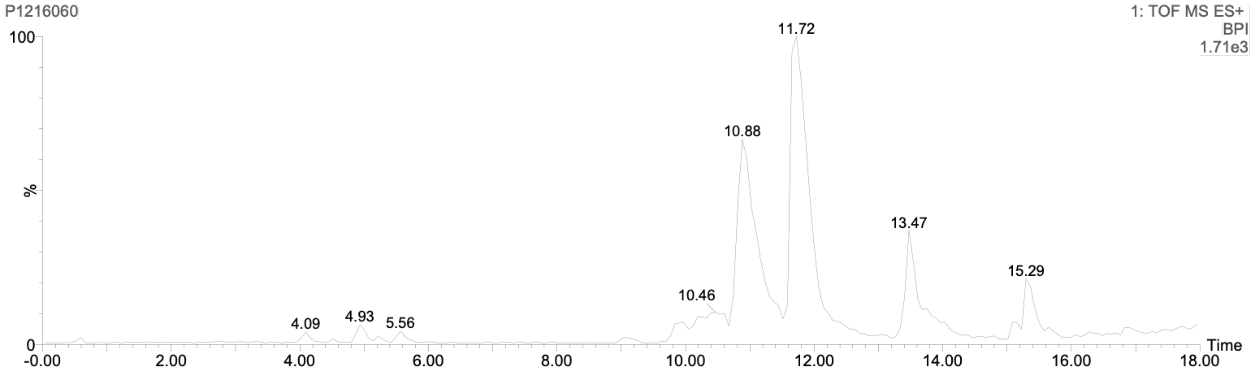

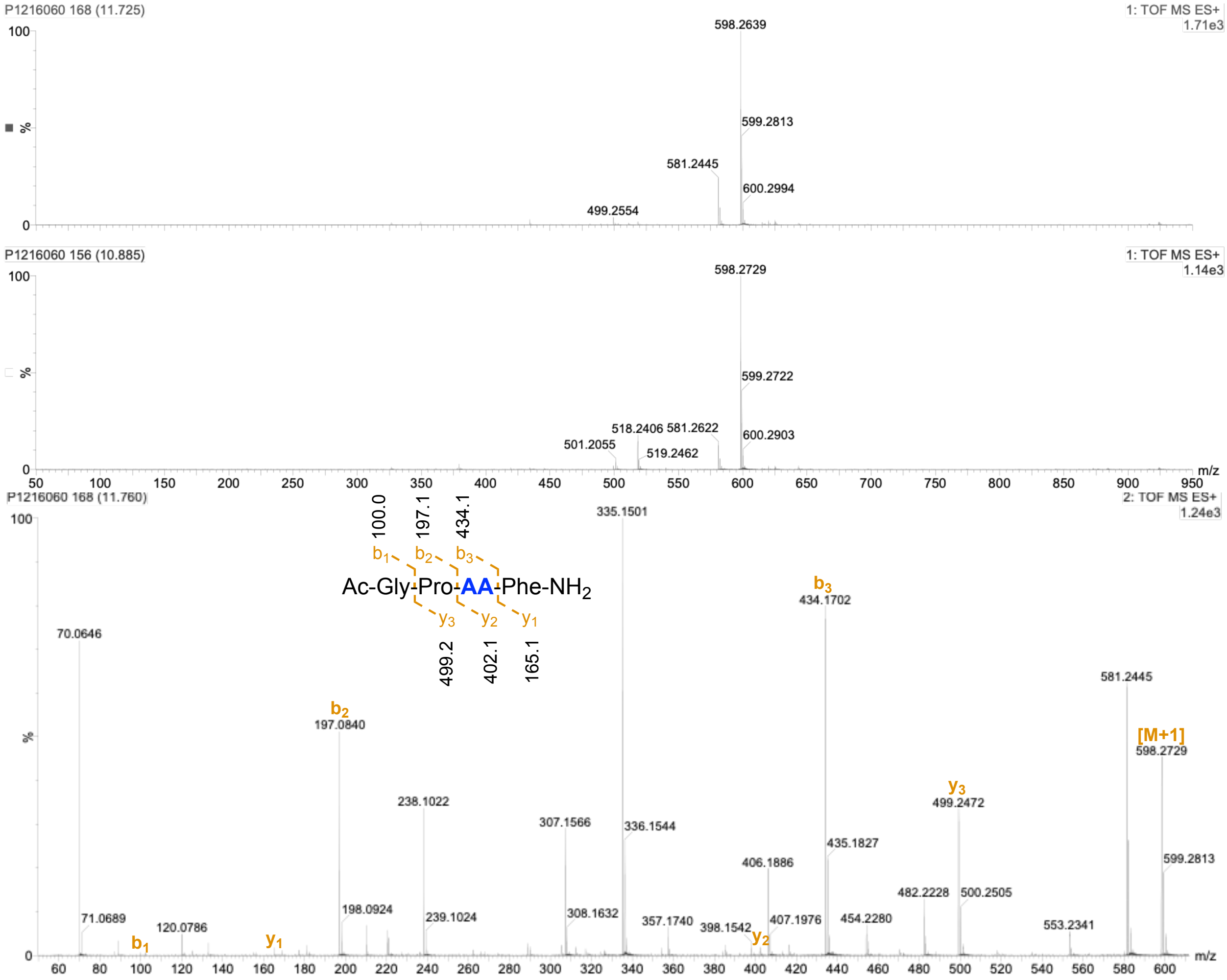

P1216060 168 (11.725)
1: TOF MS ES+
1.71e3
598.2639
599.2813
581.2445
600.2994
499.2554
P1216060 156 (10.885)
1: TOF MS ES+
1.14e3
598.2729
599.2722
518.2406
581.2622
600.2903
501.2055
519.2462
m/z
P1216060 168 (11.760)
2: TOF MS ES+
1.24e3
335.1501
100.0
197.1
434.1
Ac-Gly-Pro-AA-Phe-NH2
499.2
402.1
165.1
b3
434.1702
70.0646
581.2445
b2
197.0840
[M+1]
598.2729
y3
499.2472
238.1022
307.1566
336.1544
435.1827
599.2813
406.1886
482.2228
500.2505
198.0924
71.0689
239.1024
308.1632
357.1740
398.1542
407.1976
454.2280
553.2341
120.0786
b1
y1
y2
m/z
%

4F': MW = 599.7, Purity = 37.2%, Yield = 0.84% [0.012 mg]

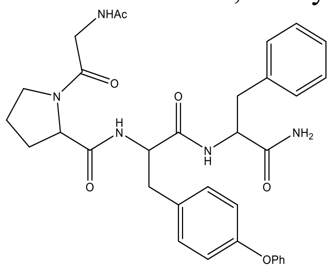

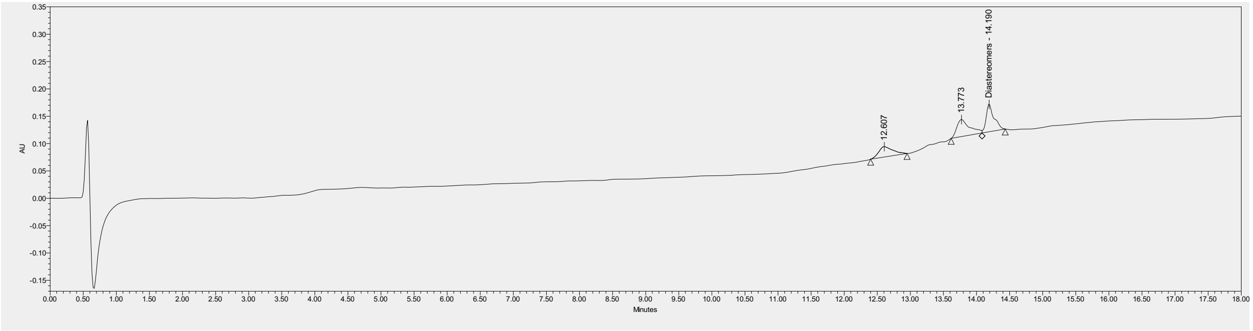

	Name	Retention Time	Area	% Area
1		12.607	281560	25.25
2		13.773	418266	37.52
3	Diastereomers	14.190	415074	37.23

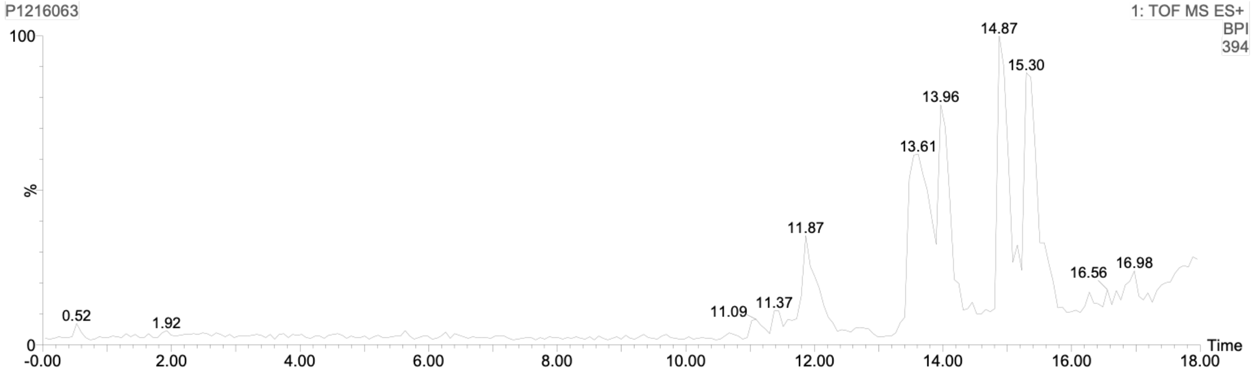

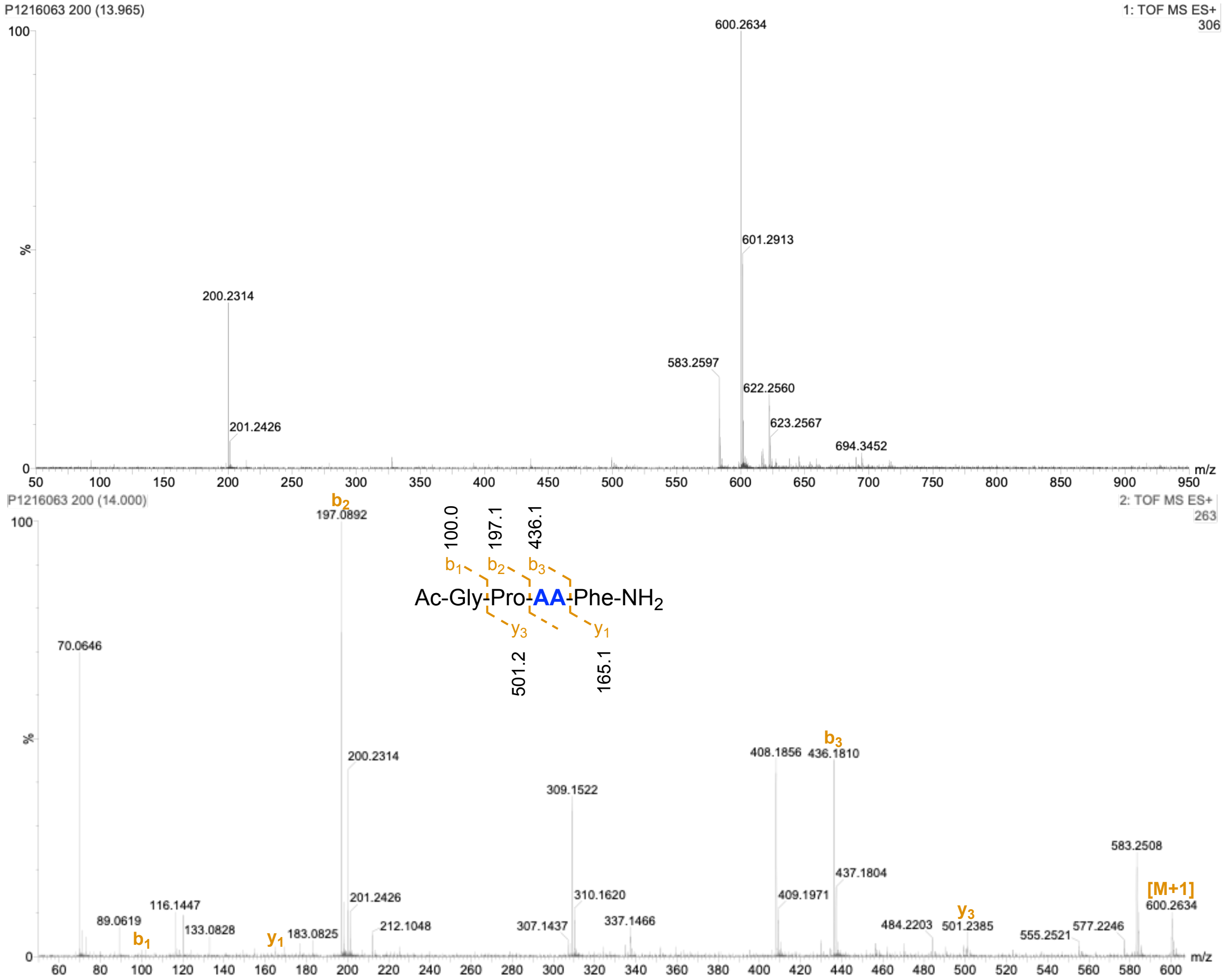
P1216063 200 (13.965)
1: TOF MS ES+
306
600.2634
601.2913
200.2314
583.2597
622.2560
623.2567
201.2426
694.3452
P1216063 200 (14.000)
2: TOF MS ES+
263
b2
197.0892
Ac-Gly-Pro-AA-Phe-NH2
100.0
197.1
436.1
501.2
165.1
70.0646
200.2314
408.1856
b3
436.1810
309.1522
583.2508
437.1804
[M+1]
600.2634
116.1447
89.0619
133.0828
201.2426
310.1620
409.1971
337.1466
212.1048
307.1437
484.2203
y3
501.2385
555.2521
577.2246
b1
y1
183.0825
m/z

5F’: MW = 597.7, Purity = 24.9%, Yield = 1.3% [0.019 mg]

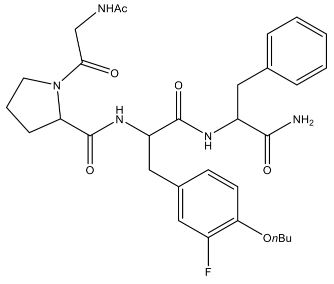

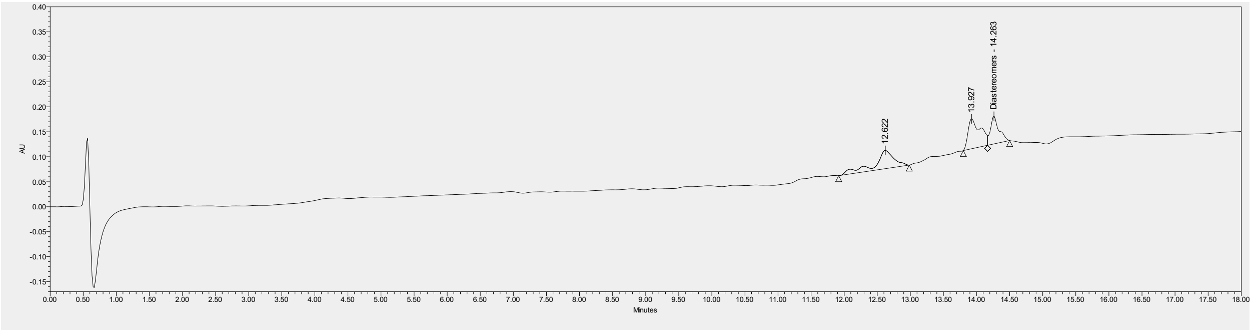

	Name	Retention Time	Area	% Area
1		12.622	742529	36.91
2		13.927	768435	38.20
3	Diastereomers	14.263	500821	24.89

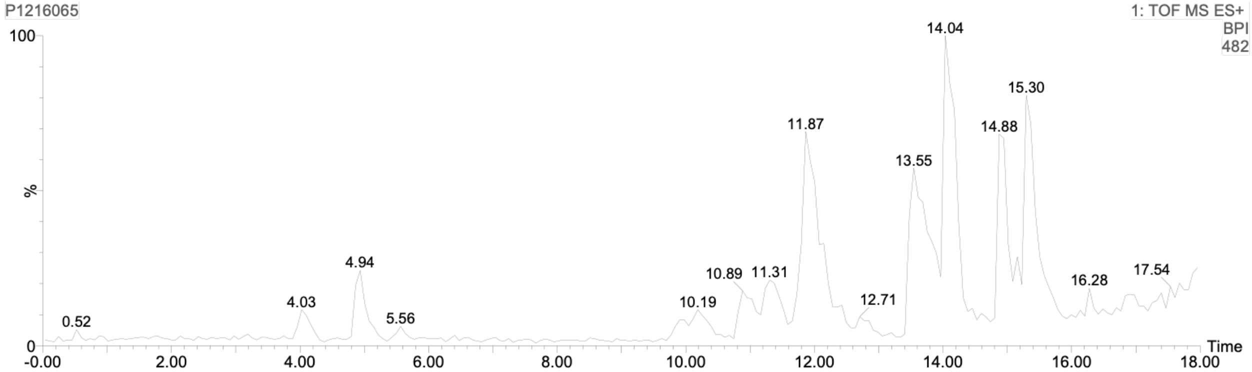

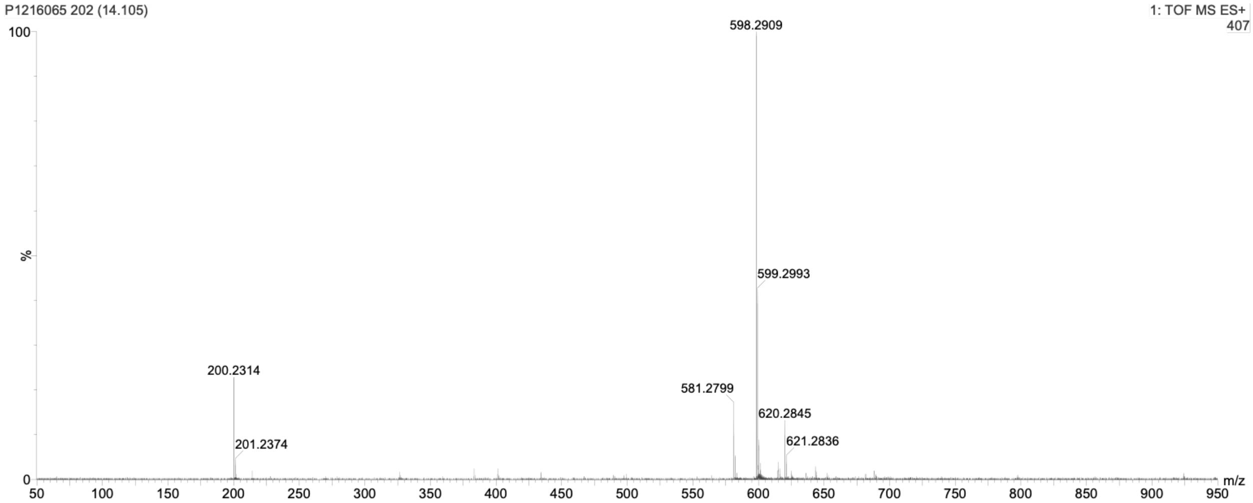
P1216065 202 (14.105)
1: TOF MS ES+
407
598.2909
599.2993
200.2314
201.2374
581.2799
620.2845
621.2836
%
m/z

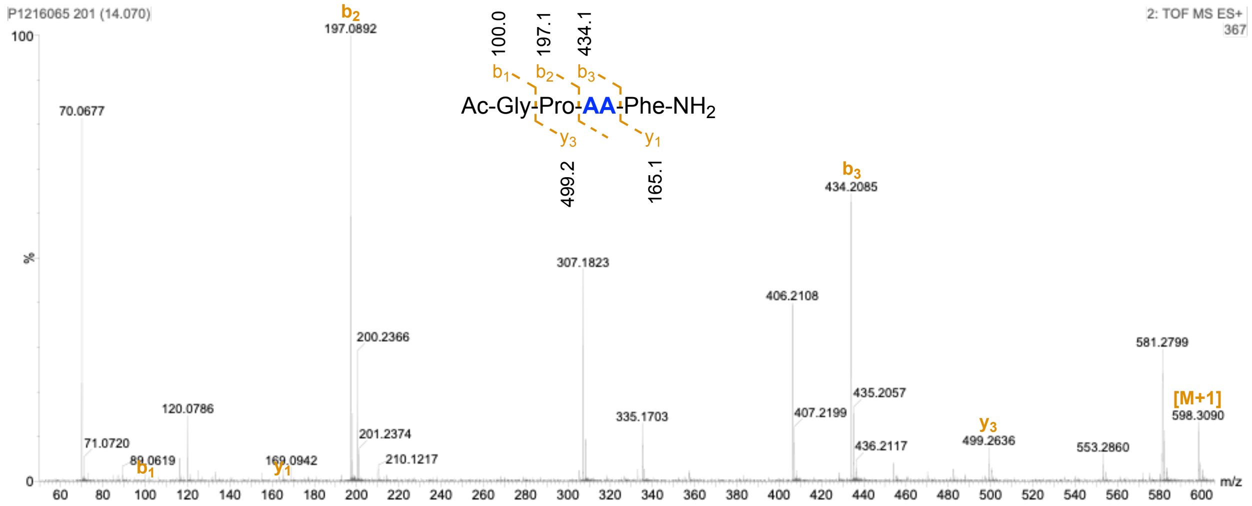
P1216065 201 (14.070)
2: TOF MS ES+
367
b2
197.0892
70.0677
Ac-Gly-Pro-AA-Phe-NH2
100.0
197.1
434.1
499.2
165.1
b1
b2
b3
y3
y1
b3
434.2085
307.1823
406.2108
200.2366
581.2799
120.0786
435.2057
[M+1]
598.3090
335.1703
407.2199
y3
499.2636
201.2374
436.2117
553.2860
71.0720
89.0619
169.0942
210.1217
%
m/z

6F': MW = 535.7, Purity = 21.0%, Yield = 6.9% [0.087 mg]

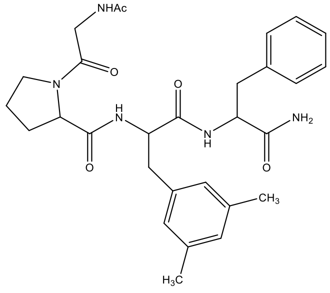

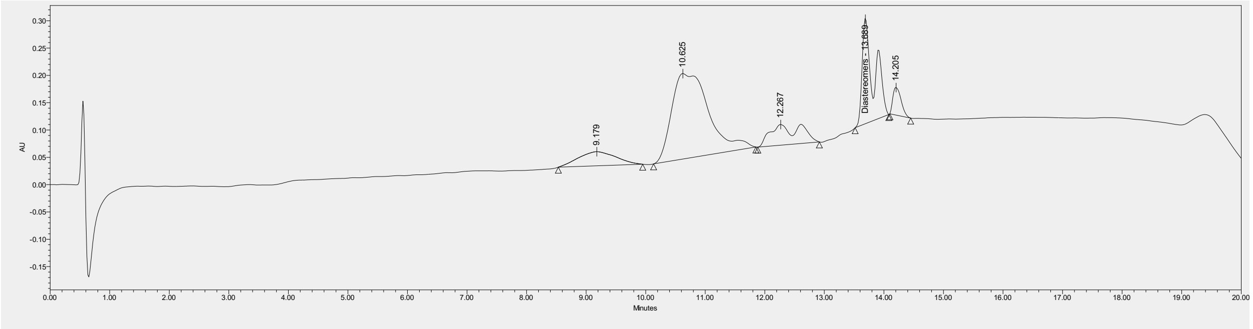

	Name	Retention Time	Area	% Area
1		9.179	1131627	9.22
2		10.625	6760693	55.06
3		12.267	1315720	10.72
4	Diastereomers	13.689	2582297	21.03
5		14.205	488816	3.98

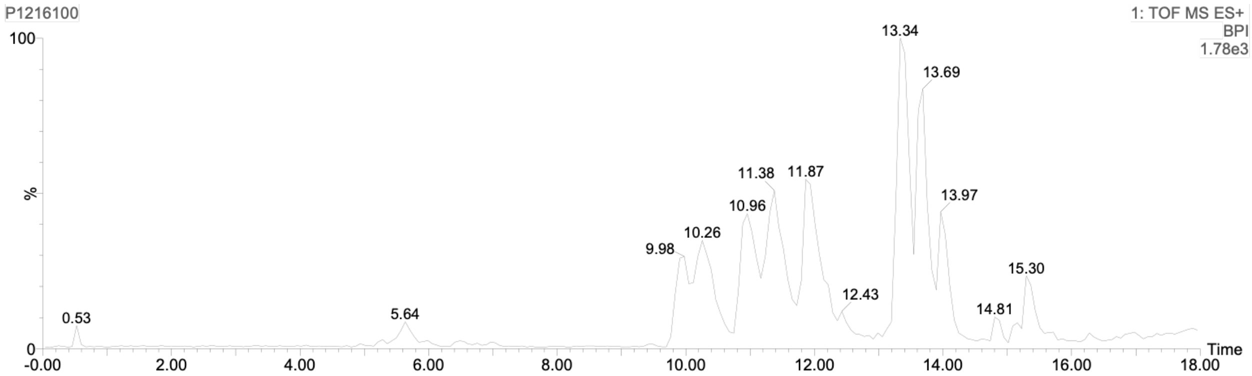

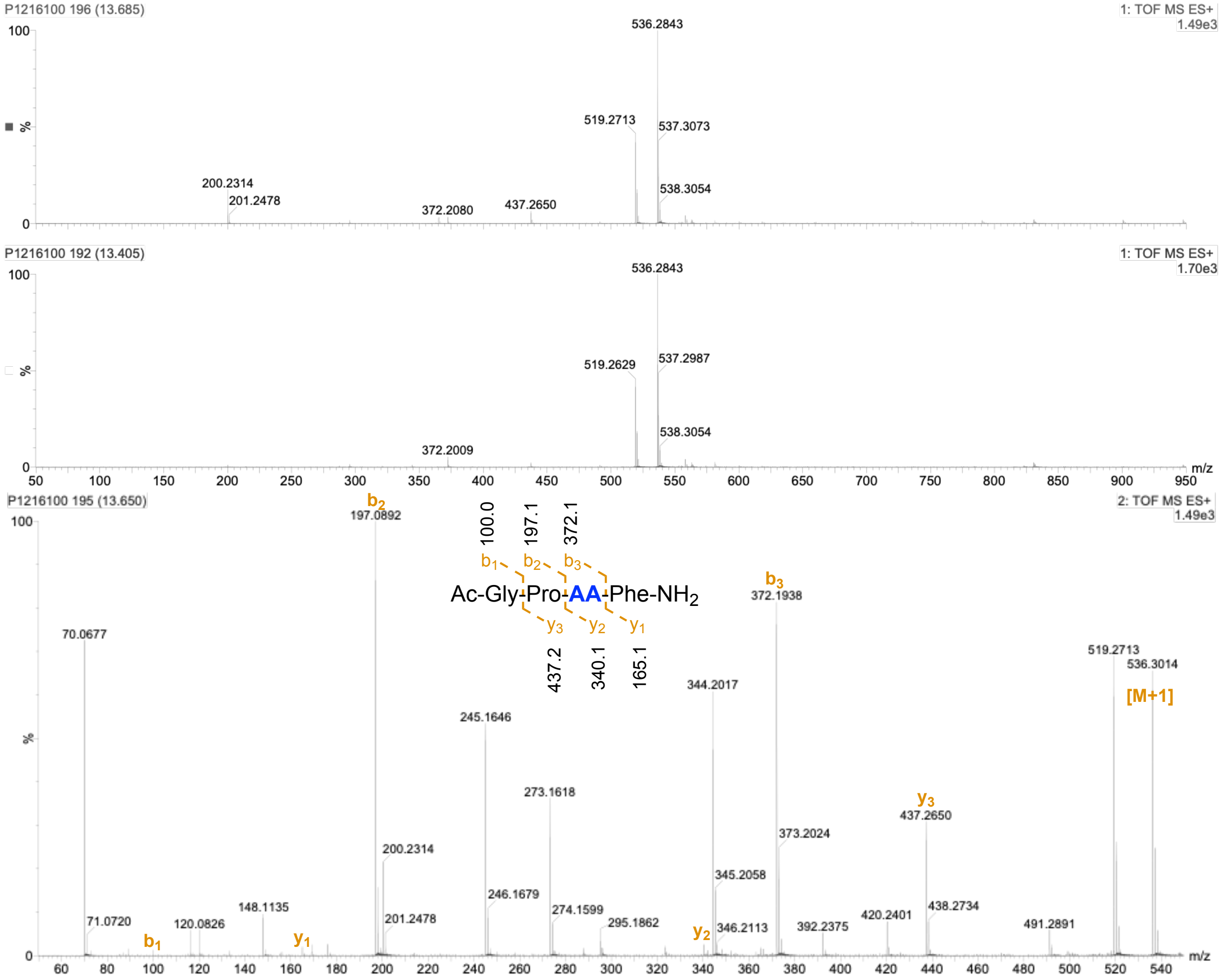
P1216100 196 (13.685)
1: TOF MS ES+
1.49e3
536.2843
519.2713
537.3073
538.3054
200.2314
201.2478
372.2080
437.2650
P1216100 192 (13.405)
1: TOF MS ES+
1.70e3
536.2843
519.2629
537.2987
538.3054
372.2009
m/z
P1216100 195 (13.650)
2: TOF MS ES+
1.49e3
b2
197.0892
100.0
197.1
372.1
b1
b2
b3
Ac-Gly-Pro-AA-Phe-NH2
y3
y2
y1
437.2
340.1
165.1
b3
372.1938
70.0677
519.2713
536.3014
[M+1]
344.2017
245.1646
273.1618
y3
437.2650
373.2024
200.2314
345.2058
246.1679
438.2734
71.0720
120.0826
148.1135
201.2478
274.1599
295.1862
420.2401
491.2891
b1
y1
y2
346.2113
392.2375
m/z

7F': MW = 564.6, Purity = 65.7%, Yield = 14.1% [0.19 mg]

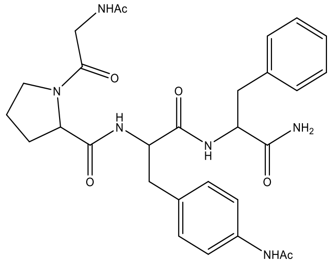

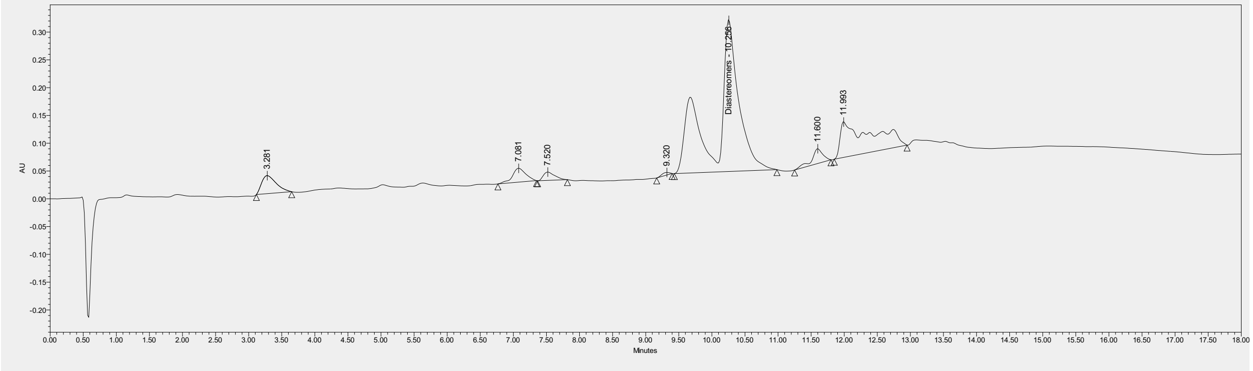

	Name	Retention Time	Area	% Area
1		3.281	496098	4.95
2		7.081	353073	3.53
3		7.520	179813	1.80
4		9.320	39917	0.40
5	Diastereomers	10.256	6577453	65.68
6		11.600	310559	3.10
7		11.993	2057287	20.54

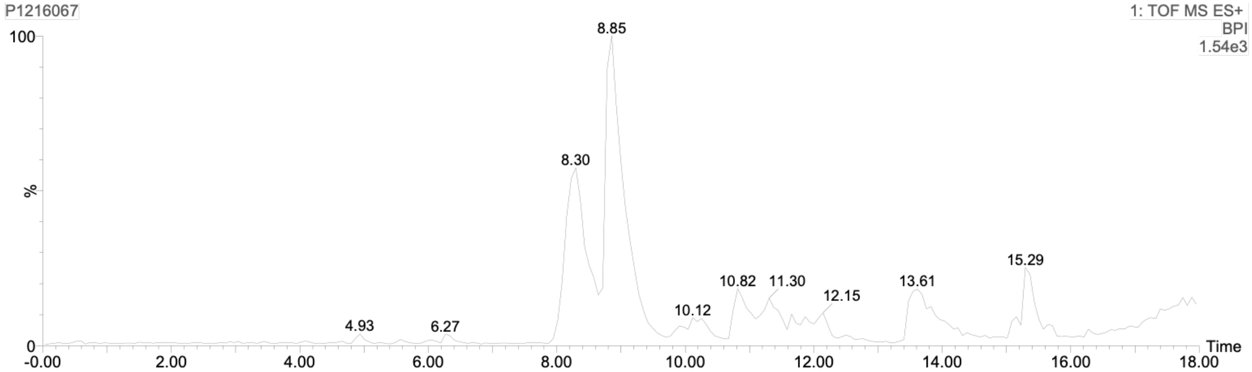

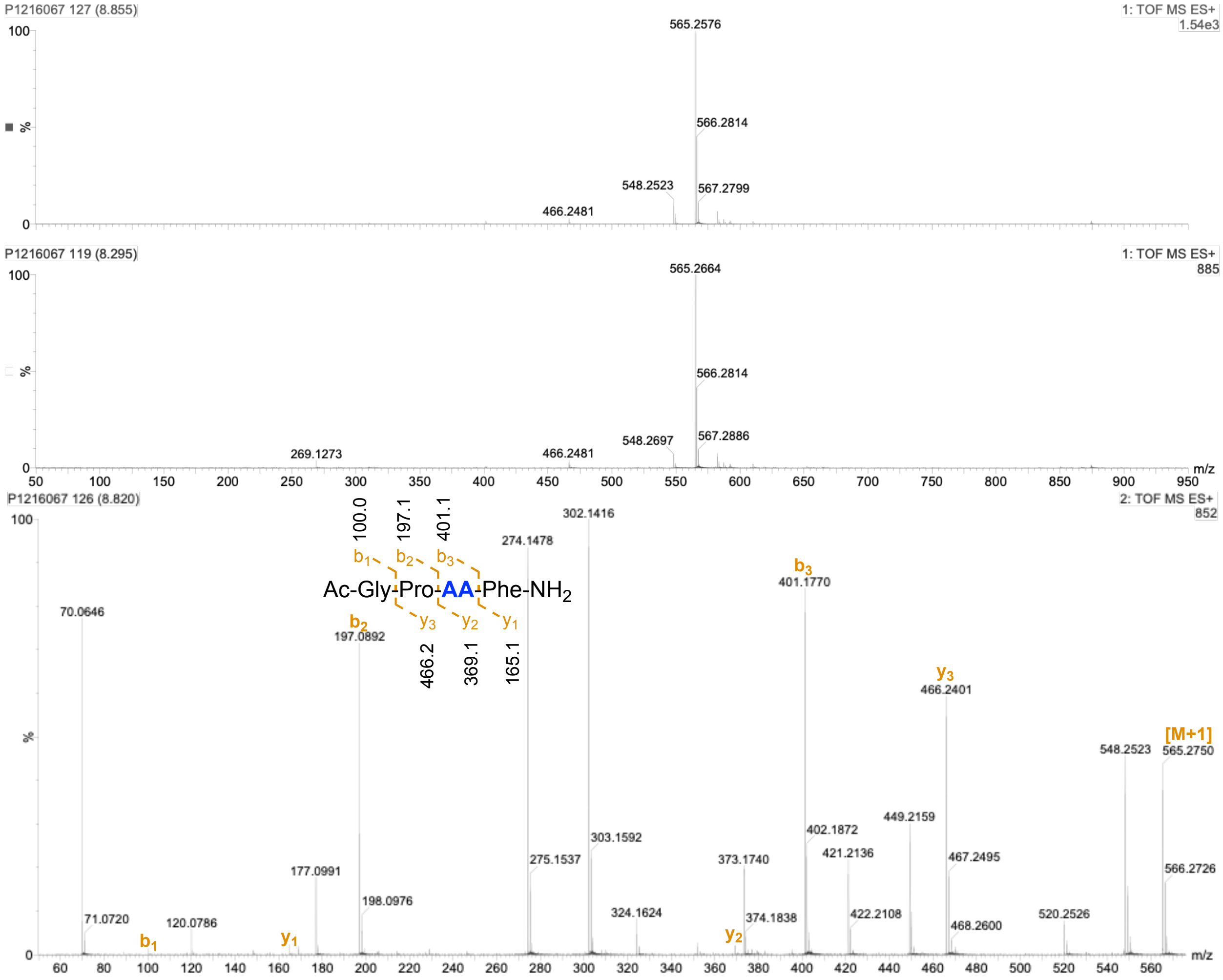

P1216067 127 (8.855)
1: TOF MS ES+
1.54e3
565.2576
566.2814
548.2523
567.2799
466.2481
P1216067 119 (8.295)
1: TOF MS ES+
885
565.2664
566.2814
548.2697
567.2886
466.2481
269.1273
m/z
P1216067 126 (8.820)
2: TOF MS ES+
852
Ac-Gly-Pro-AA-Phe-NH2
b1 b2 b3
y3 y2 y1
100.0
197.1
401.1
466.2
369.1
165.1
302.1416
274.1478
70.0646
b2
197.0892
b3
401.1770
y3
466.2401
[M+1]
565.2750
548.2523
449.2159
402.1872
421.2136
303.1592
373.1740
467.2495
275.1537
566.2726
177.0991
198.0976
324.1624
374.1838
422.2108
520.2526
468.2600
71.0720
120.0786
b1
y1
y2
m/z

8F’: MW = 622.7, Purity = 82.8%, Yield = 21.0% [0.31 mg]

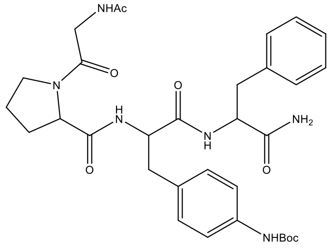

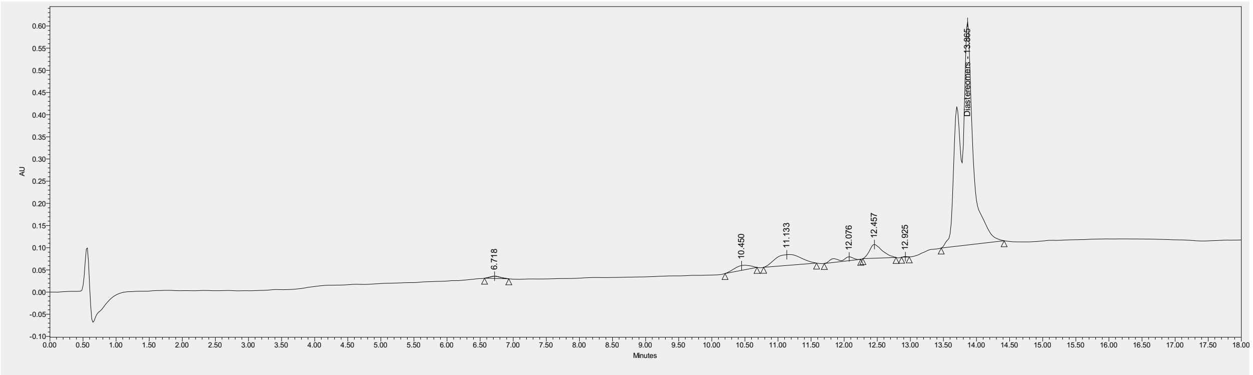

	Name	Retention Time	Area	% Area
1		6.718	56341	0.66
2		10.450	180159	2.10
3		11.133	666593	7.77
4		12.076	148717	1.73
5		12.457	417768	4.87
6		12.925	4582	0.05
7	Diastereomers	13.865	7109923	82.83

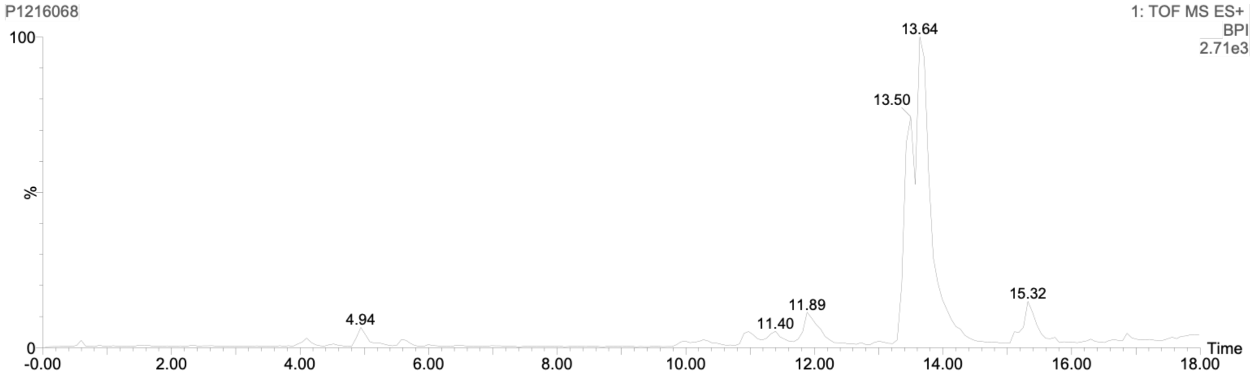

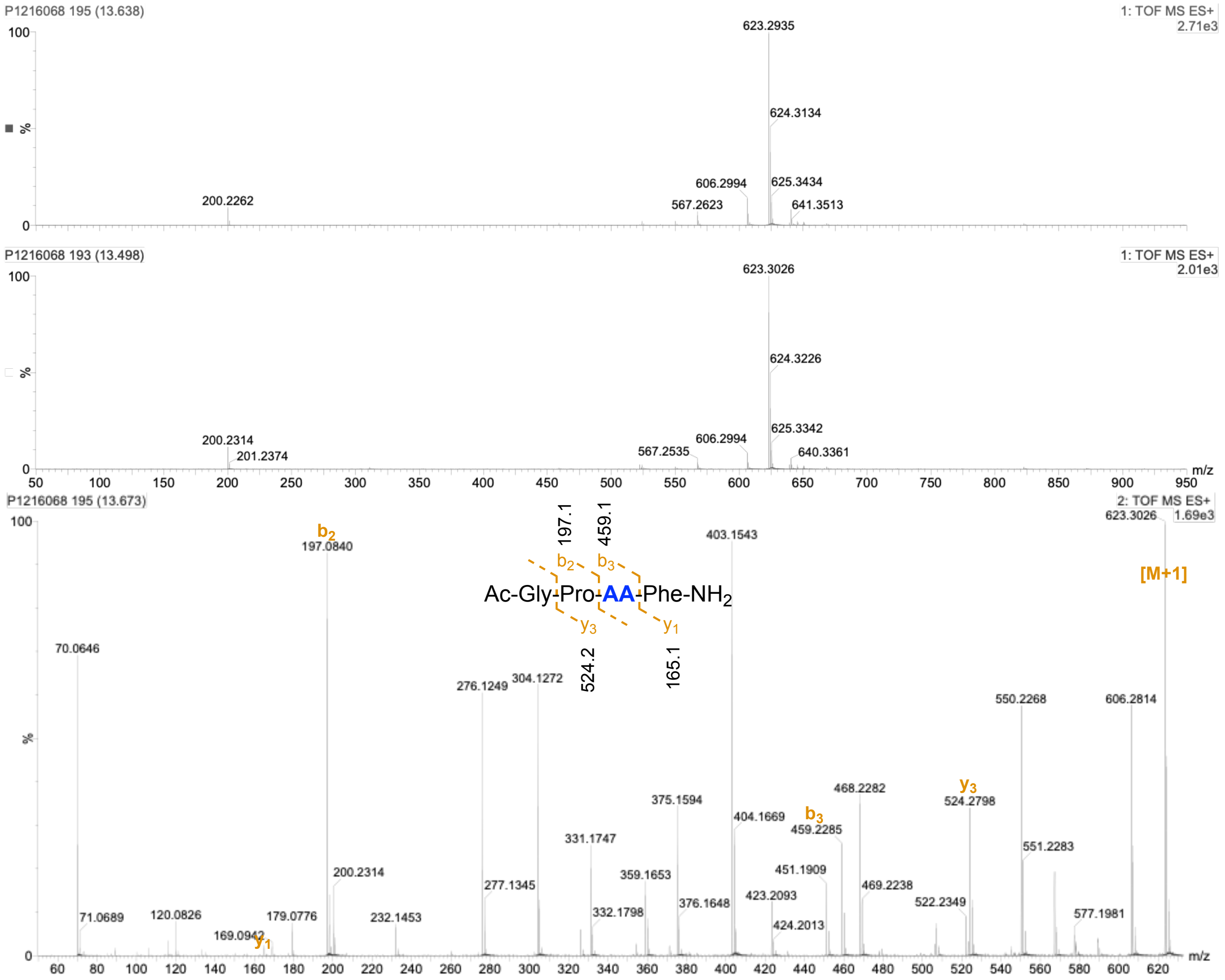

P1216068 195 (13.638)
1: TOF MS ES+
2.71e3
623.2935
624.3134
606.2994
625.3434
200.2262
567.2623
641.3513
P1216068 193 (13.498)
1: TOF MS ES+
2.01e3
623.3026
624.3226
625.3342
200.2314
201.2374
567.2535
606.2994
640.3361
m/z
P1216068 195 (13.673)
2: TOF MS ES+
623.3026
1.69e3
Ac-Gly-Pro-AA-Phe-NH2
197.1
459.1
524.2
165.1
b2
b3
y3
y1
[M+1]
197.0840
403.1543
70.0646
276.1249
304.1272
550.2268
606.2814
375.1594
468.2282
524.2798
404.1669
459.2285
331.1747
551.2283
200.2314
451.1909
359.1653
277.1345
469.2238
423.2093
376.1648
522.2349
71.0689
120.0826
179.0776
232.1453
332.1798
577.1981
424.2013
169.0942

9F': MW = 583.7, Purity = 53.4%, Yield = 1.2% [0.017 mg]

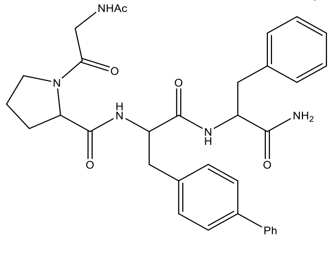

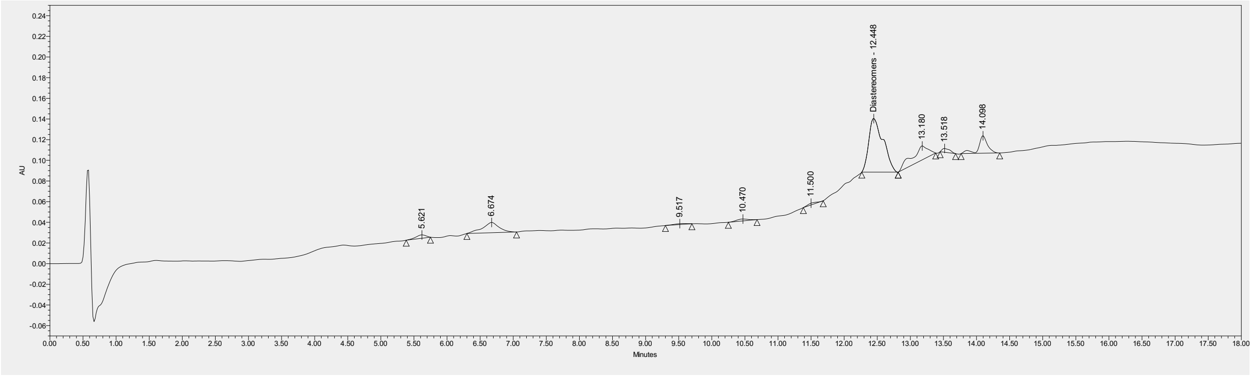

	Name	Retention Time	Area	% Area
1		5.621	35000	2.37
2		6.674	174226	11.82
3		9.517	11326	0.77
4		10.470	22624	1.53
5		11.500	14982	1.02
6	Diastereomers	12.448	787254	53.41
7		13.180	239573	16.25
8		13.518	30960	2.10
9		14.098	158116	10.73

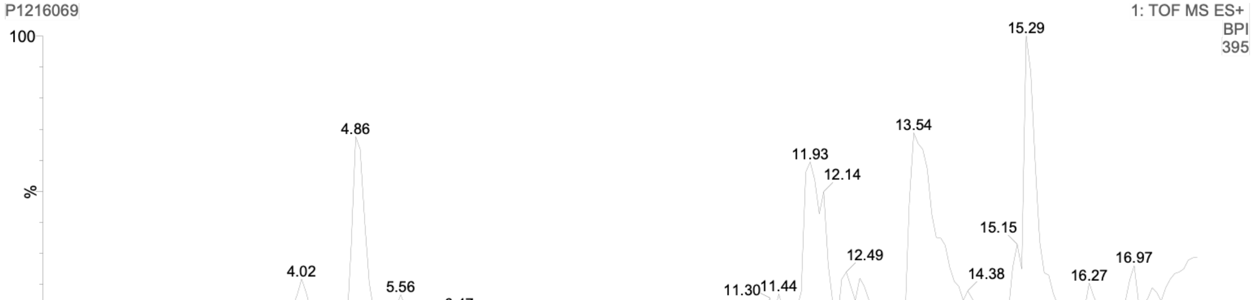

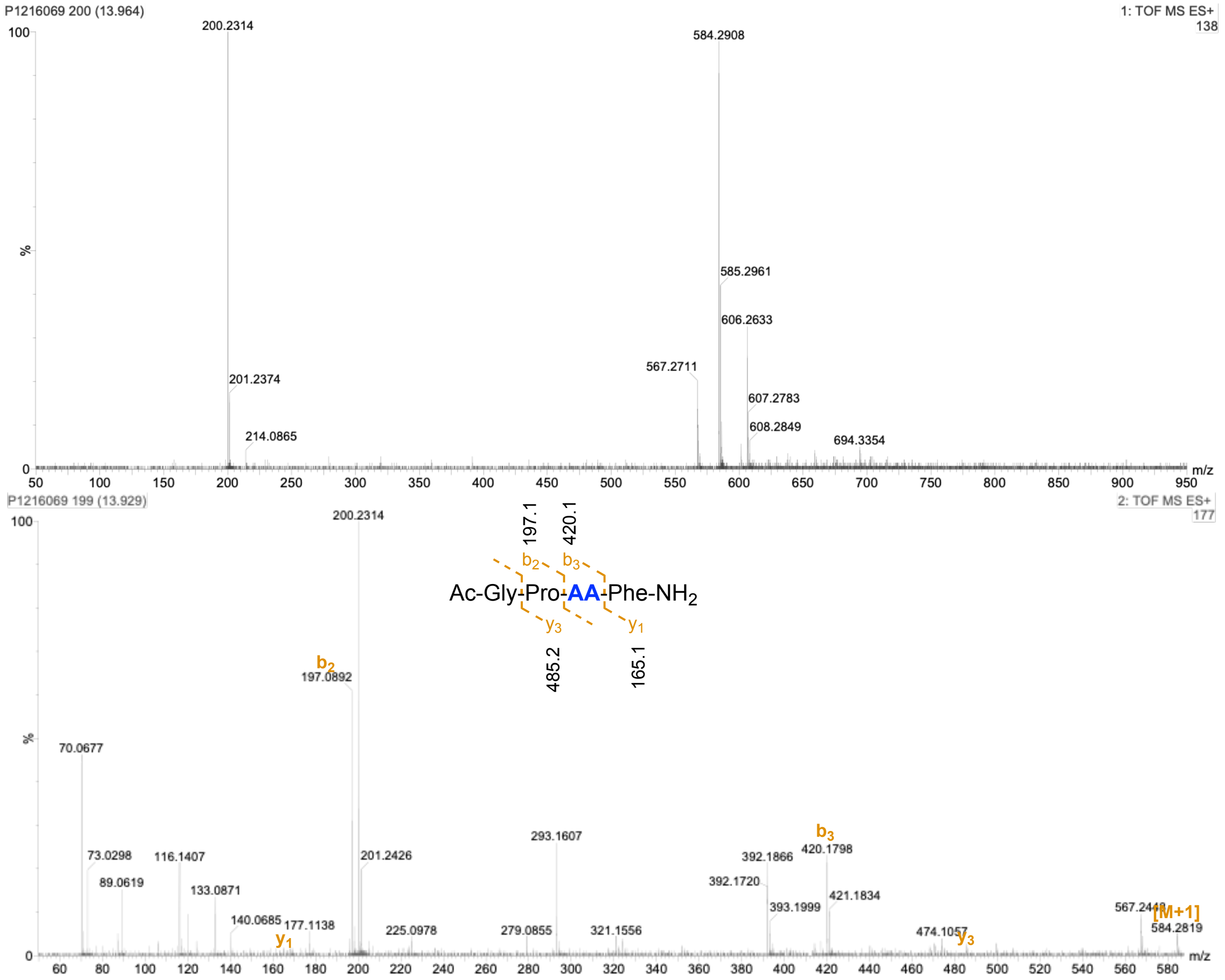
P1216069 200 (13.964)
1: TOF MS ES+
138
200.2314
584.2908
585.2961
606.2633
567.2711
201.2374
607.2783
608.2849
214.0865
694.3354
%
m/z
P1216069 199 (13.929)
2: TOF MS ES+
177
200.2314
197.1
420.1
Ac-Gly-Pro-AA-Phe-NH2
485.2
165.1
b2
b3
y3
y1
197.0892
70.0677
293.1607
420.1798
73.0298
116.1407
201.2426
392.1866
89.0619
133.0871
392.1720
421.1834
393.1999
140.0685
177.1138
225.0978
279.0855
321.1556
474.1057
567.2449
[M+1]
584.2819

10F': MW = 584.7, Purity = 78.8%, Yield = 16.0% [0.22 mg]

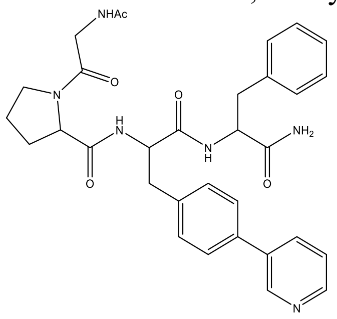

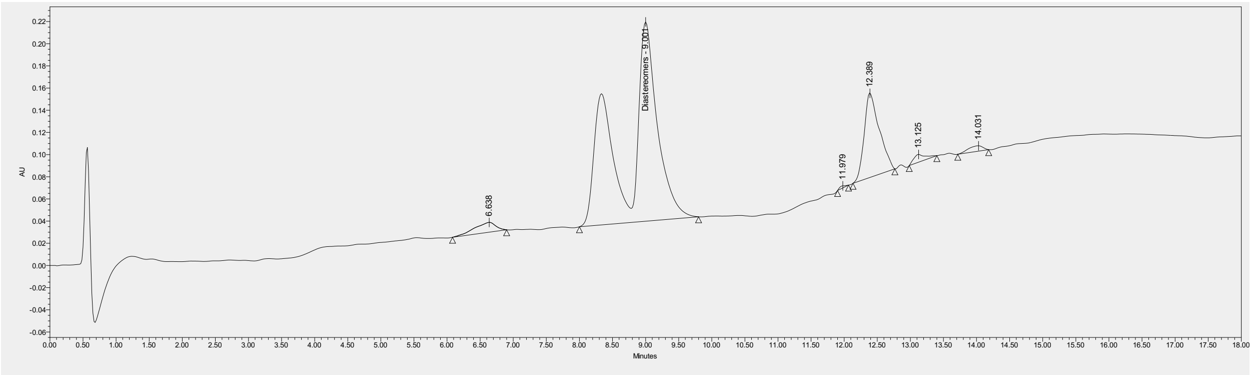

	Name	Retention Time	Area	% Area
1		6.638	203748	2.74
2	Diastereomers	9.001	5849060	78.67
3		11.979	12251	0.16
4		12.389	1207018	16.23
5		13.125	84923	1.14
6		14.031	77733	1.05

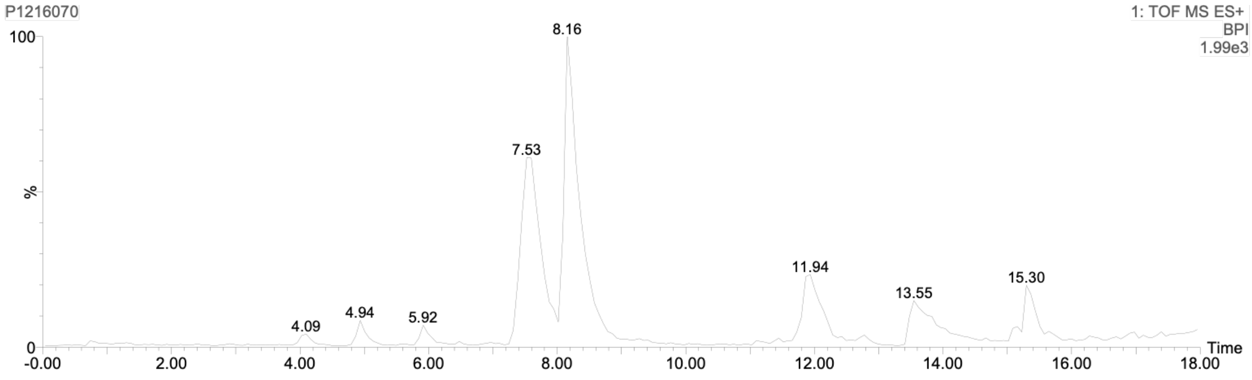

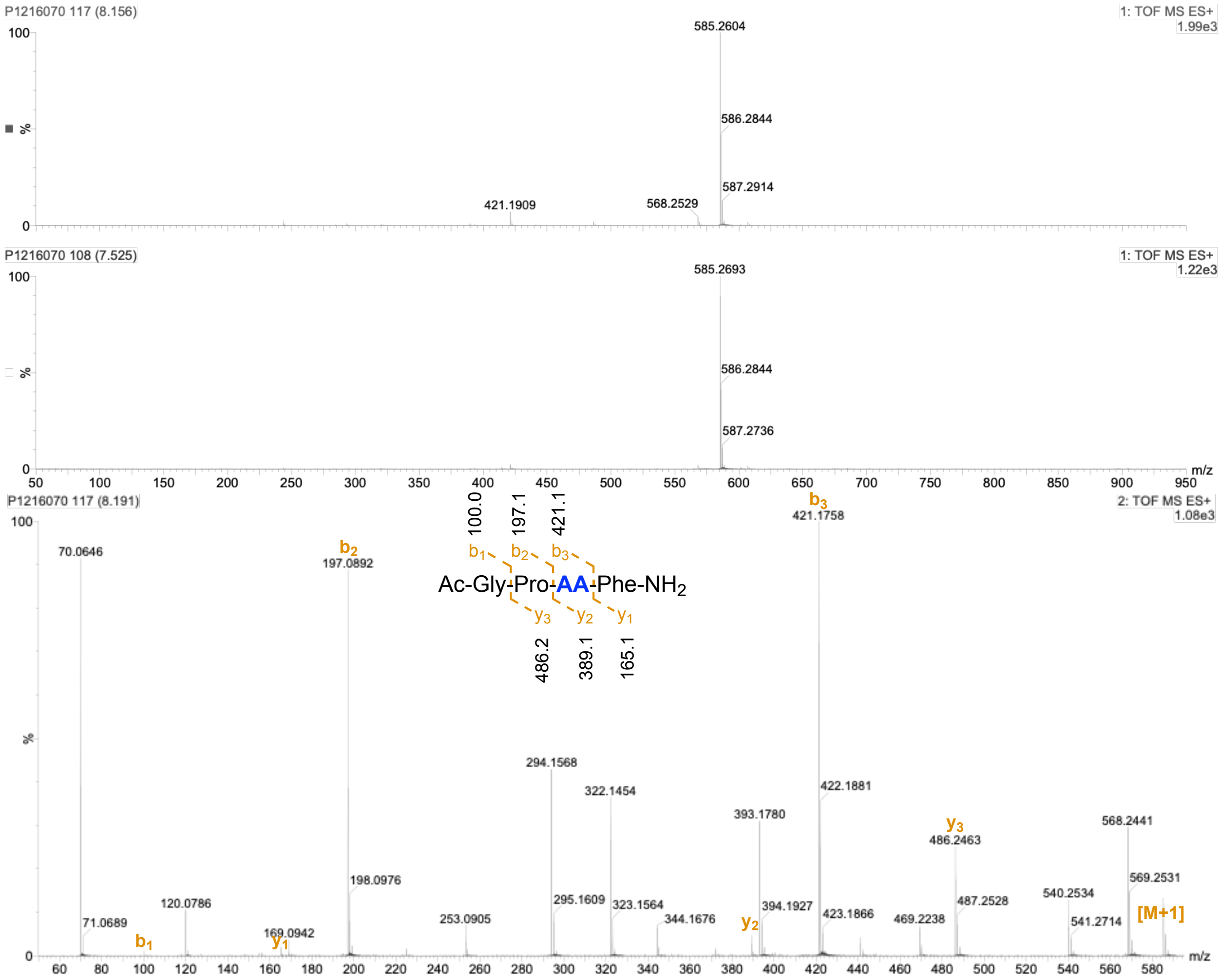

P1216070 117 (8.156)
1: TOF MS ES+
1.99e3
585.2604
586.2844
587.2914
568.2529
421.1909
P1216070 108 (7.525)
1: TOF MS ES+
1.22e3
585.2693
586.2844
587.2736
m/z
P1216070 117 (8.191)
2: TOF MS ES+
1.08e3
Ac-Gly-Pro-AA-Phe-NH2
100.0
197.1
421.1
486.2
389.1
165.1
b1
b2
b3
y1
y2
y3
70.0646
71.0689
120.0786
169.0942
197.0892
198.0976
253.0905
294.1568
295.1609
322.1454
323.1564
344.1676
393.1780
394.1927
421.1758
422.1881
423.1866
469.2238
486.2463
487.2528
540.2534
541.2714
568.2441
569.2531
[M+1]
m/z

11F’: MW = 557.7, Purity = 72.8%, Yield = 3.4% [0.044 mg]

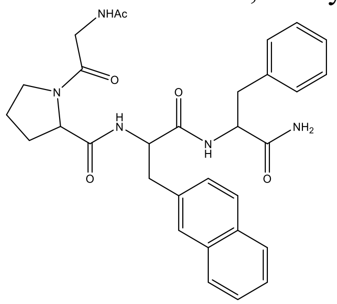

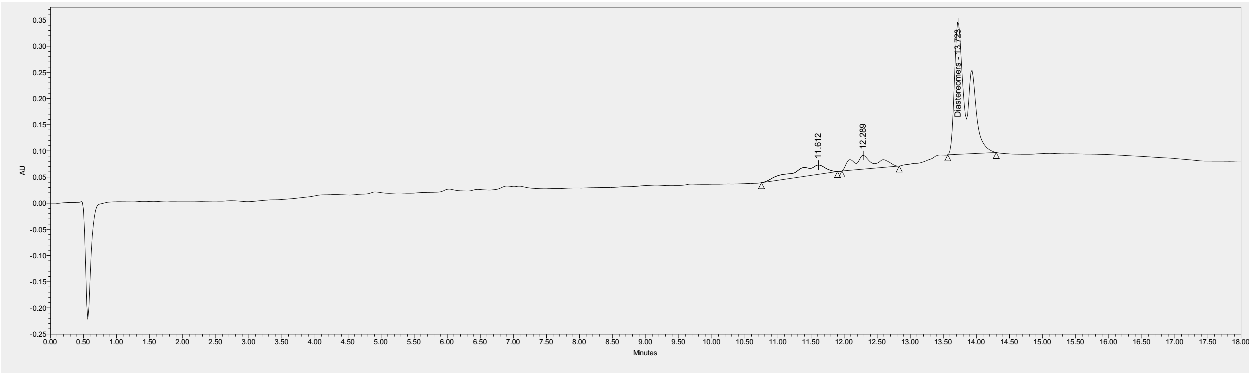

	Name	Retention Time	Area	% Area
1		11.612	663090	13.73
2		12.289	650249	13.46
3	Diastereomers	13.723	3517431	72.81

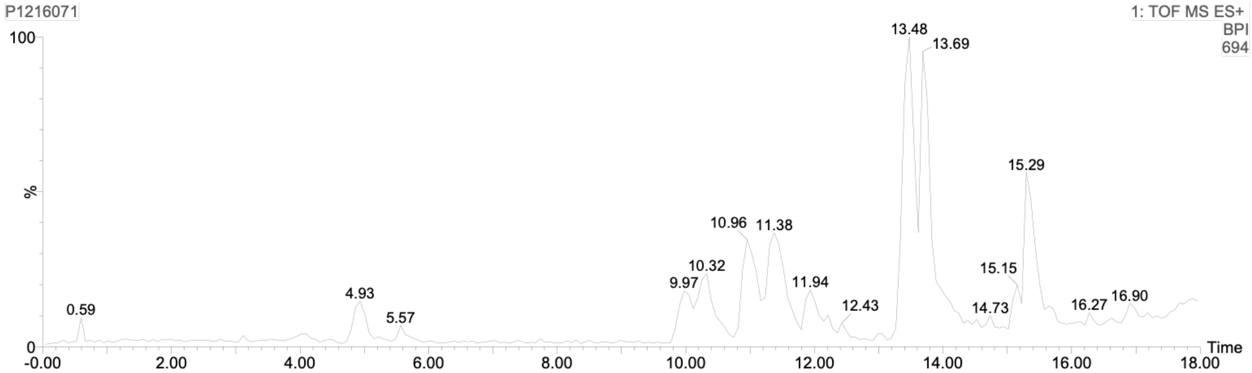

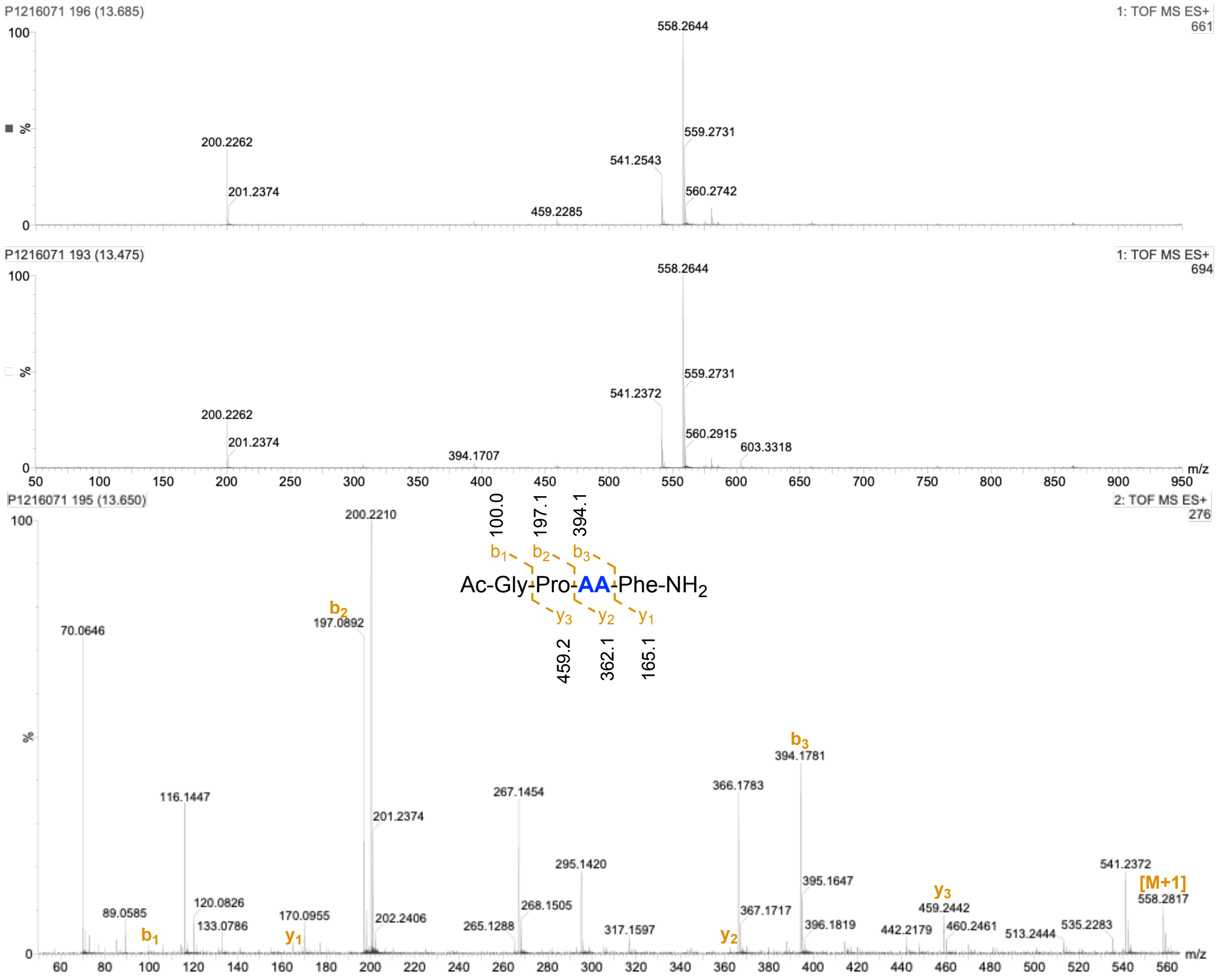

P1216071 196 (13.685)
1: TOF MS ES+
661
558.2644
559.2731
541.2543
200.2262
201.2374
560.2742
459.2285
P1216071 193 (13.475)
1: TOF MS ES+
694
558.2644
559.2731
541.2372
200.2262
201.2374
560.2915
603.3318
394.1707
m/z
P1216071 195 (13.650)
2: TOF MS ES+
276
200.2210
Ac-Gly-Pro-AA-Phe-NH2
100.0
197.1
394.1
459.2
362.1
165.1
70.0646
b2
197.0892
116.1447
b3
394.1781
366.1783
267.1454
201.2374
295.1420
395.1647
y3
459.2442
541.2372
[M+1]
558.2817
89.0585
120.0826
133.0786
170.0955
202.2406
268.1505
265.1288
317.1597
367.1717
396.1819
442.2179
460.2461
513.2444
535.2283
b1
y1
y2
m/z

12F’: MW = 557.7, Purity = 86.1%, Yield = 6.5% [0.084 mg]

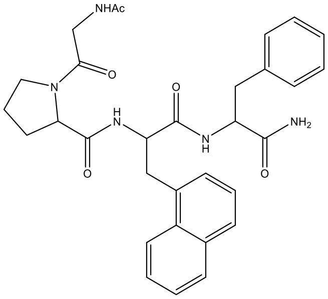

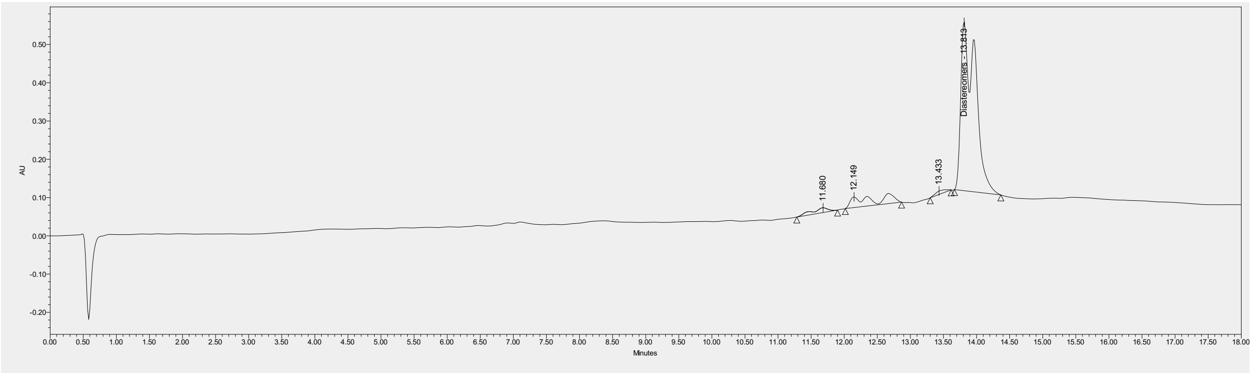

	Name	Retention Time	Area	% Area
1		11.680	227134	2.89
2		12.149	767459	9.76
3		13.433	100495	1.28
4	Diastereomers	13.813	6768724	86.07

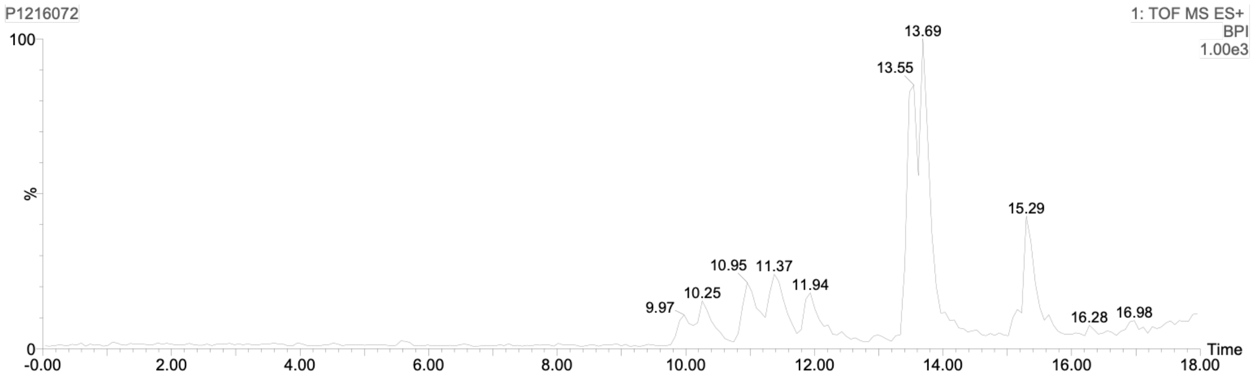

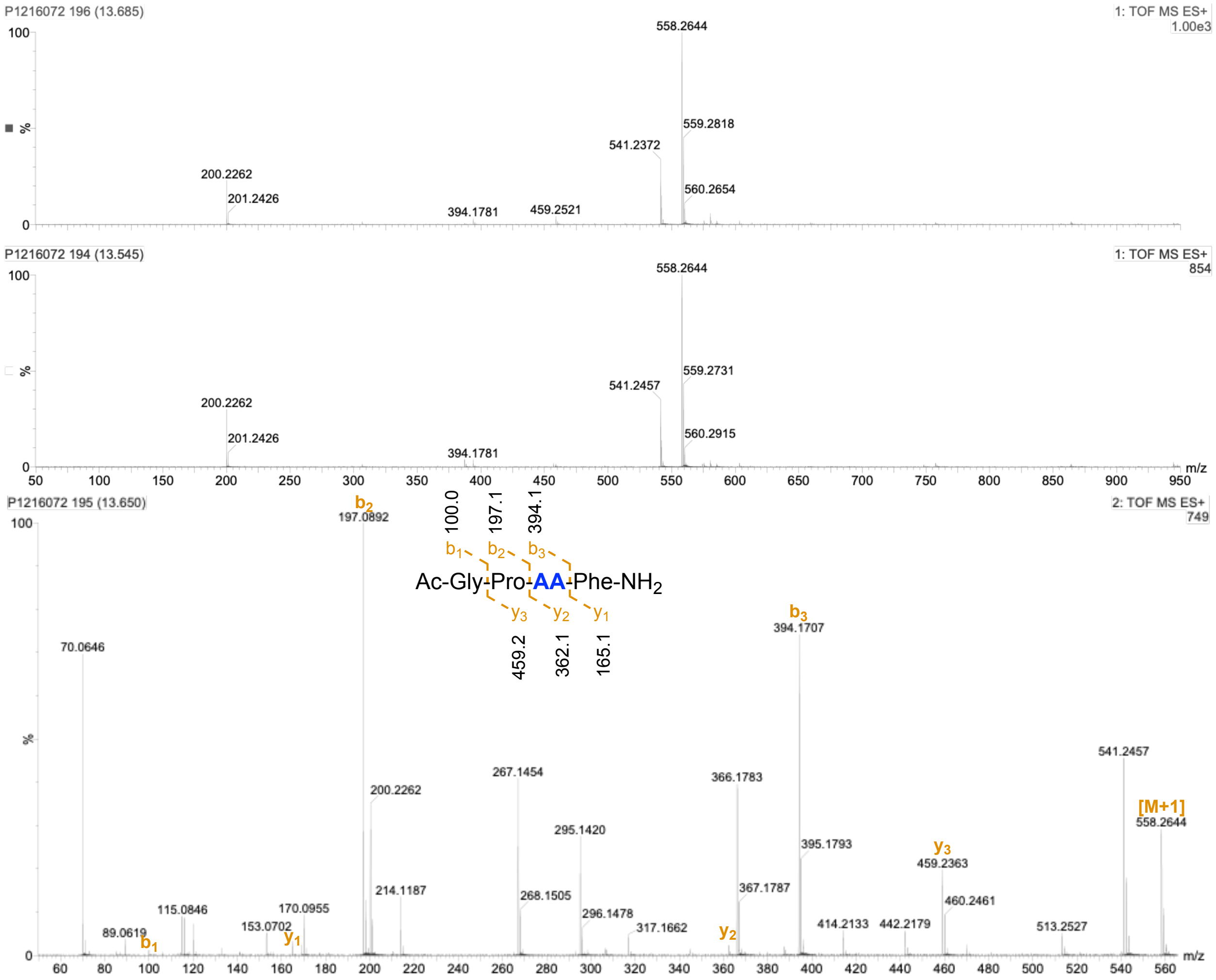
P1216072 196 (13.685)
1: TOF MS ES+
1.00e3
558.2644
559.2818
541.2372
200.2262
201.2426
560.2654
394.1781
459.2521
P1216072 194 (13.545)
1: TOF MS ES+
854
558.2644
559.2731
541.2457
200.2262
201.2426
560.2915
394.1781
m/z
P1216072 195 (13.650)
2: TOF MS ES+
749
b2
197.0892
100.0
197.1
394.1
Ac-Gly-Pro-AA-Phe-NH2
459.2
362.1
165.1
b3
394.1707
70.0646
541.2457
[M+1]
558.2644
267.1454
366.1783
200.2262
295.1420
395.1793
y3
459.2363
214.1187
367.1787
268.1505
460.2461
115.0846
170.0955
296.1478
317.1662
y2
414.2133
442.2179
513.2527
89.0619
b1
153.0702
y1
m/z

1G’: MW = 551.6, Purity = 87.9%, Yield = 23.1% [0.30 mg]

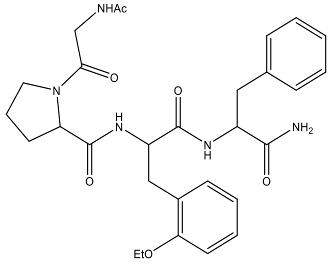

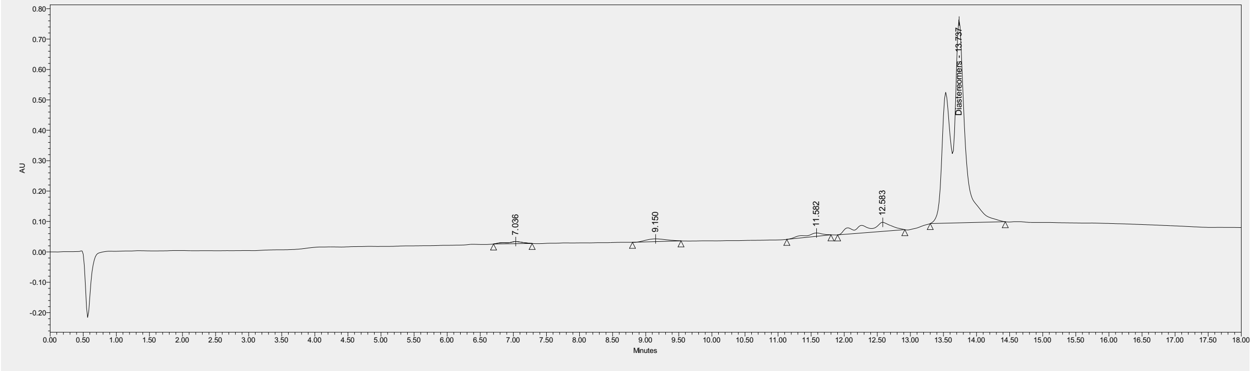

	Name	Retention Time	Area	% Area
1		7.036	125645	1.03
2		9.150	194099	1.59
3		11.582	218590	1.79
4		12.583	941999	7.70
5	Diastereomers	13.737	10750959	87.90

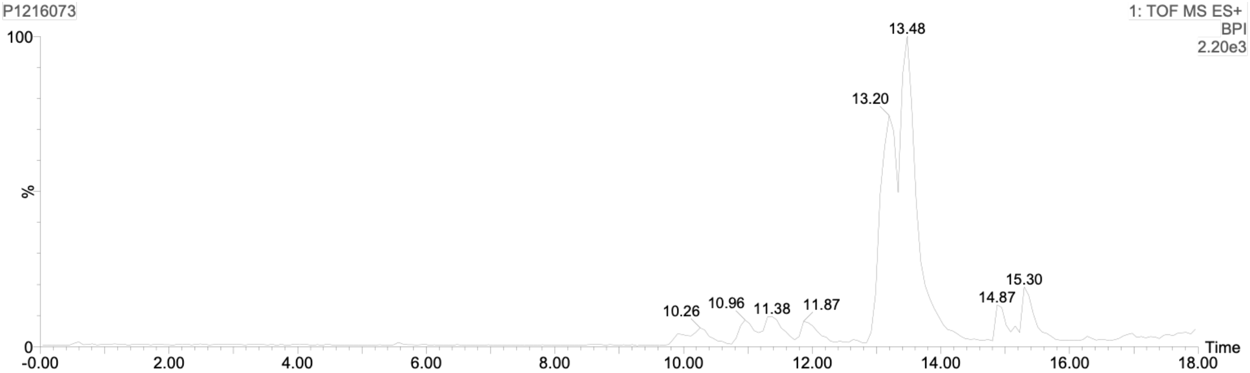

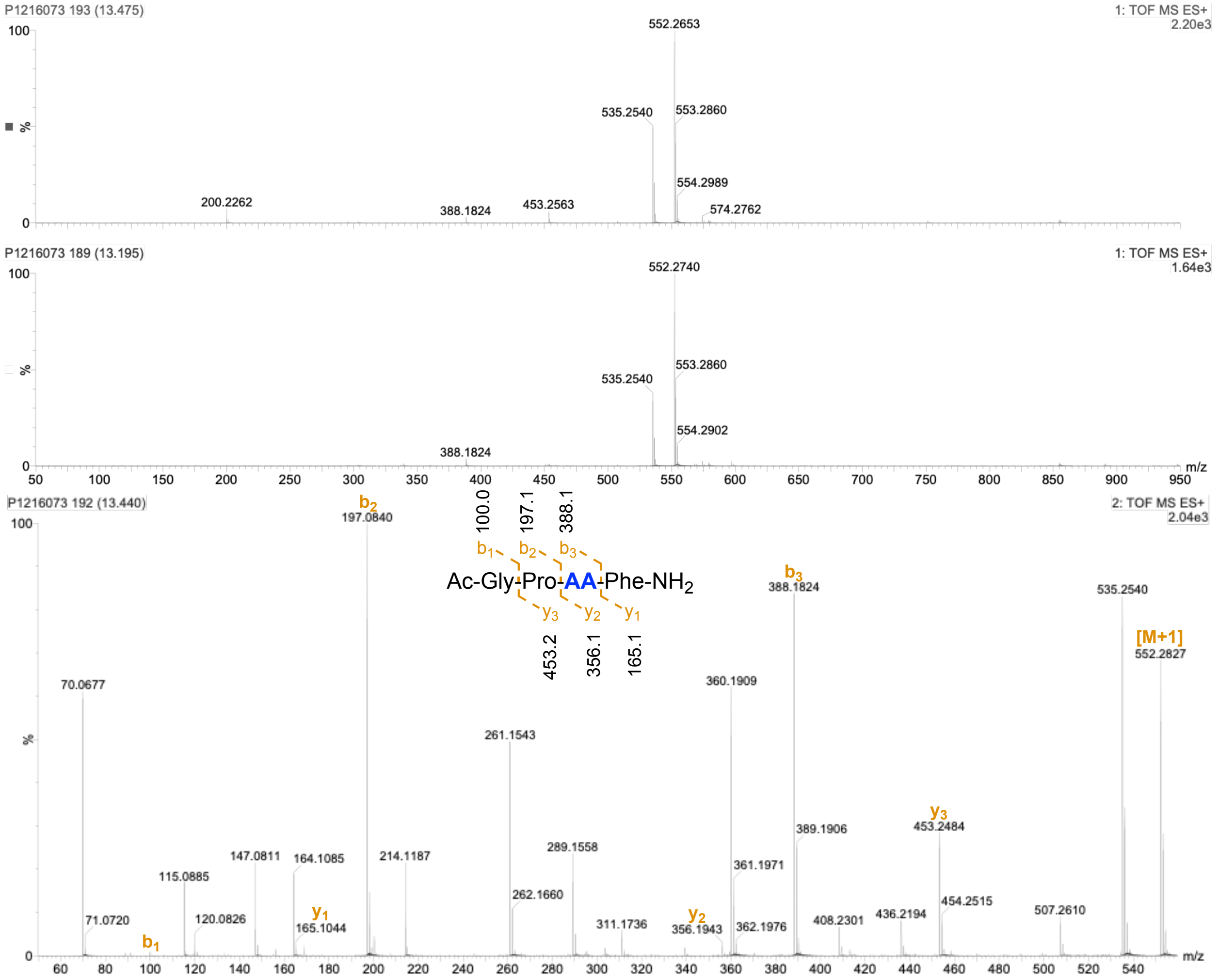
P1216073 193 (13.475)
1: TOF MS ES+
2.20e3
552.2653
535.2540
553.2860
554.2989
200.2262
388.1824
453.2563
574.2762
P1216073 189 (13.195)
1: TOF MS ES+
1.64e3
552.2740
553.2860
535.2540
554.2902
388.1824
m/z
P1216073 192 (13.440)
2: TOF MS ES+
2.04e3
Ac-Gly-Pro-AA-Phe-NH2
100.0
197.1
388.1
453.2
356.1
165.1
b2
197.0840
b3
388.1824
535.2540
[M+1]
552.2827
70.0677
360.1909
261.1543
y3
453.2484
389.1906
289.1558
361.1971
147.0811
164.1085
214.1187
115.0885
262.1660
454.2515
436.2194
507.2610
71.0720
120.0826
y1
165.1044
311.1736
y2
356.1943
362.1976
408.2301
b1
m/z

2G’: MW = 521.6, Purity = 73.5%, Yield = 4.7% [0.057 mg]

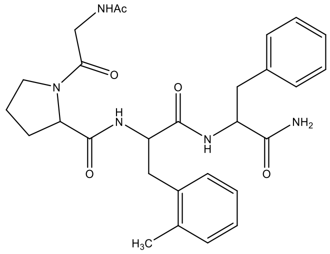

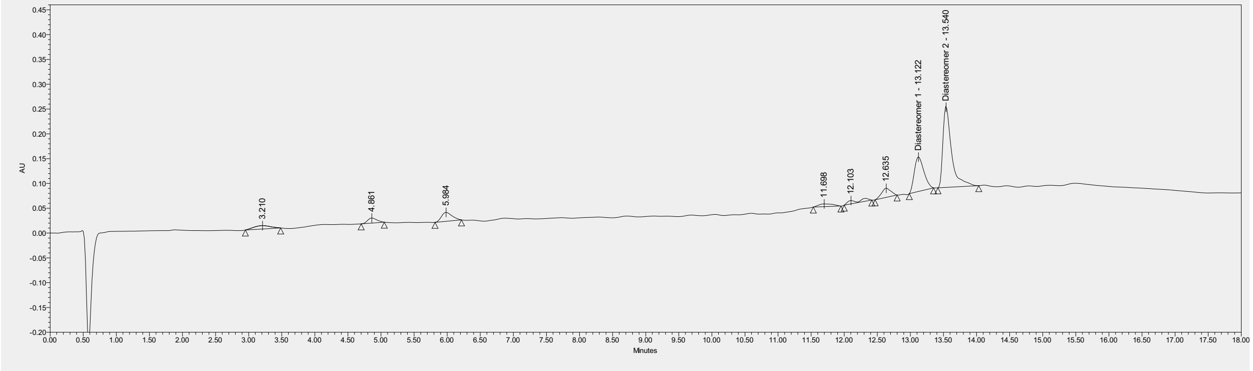

	Name	Retention Time	Area	% Area
1		3.210	123987	4.18
2		4.861	100252	3.38
3		5.984	200970	6.77
4		11.698	84061	2.83
5		12.103	96460	3.25
6		12.635	181686	6.12
7	Diastereomer 1	13.122	700423	23.61
8	Diastereomer 2	13.540	1479393	49.86

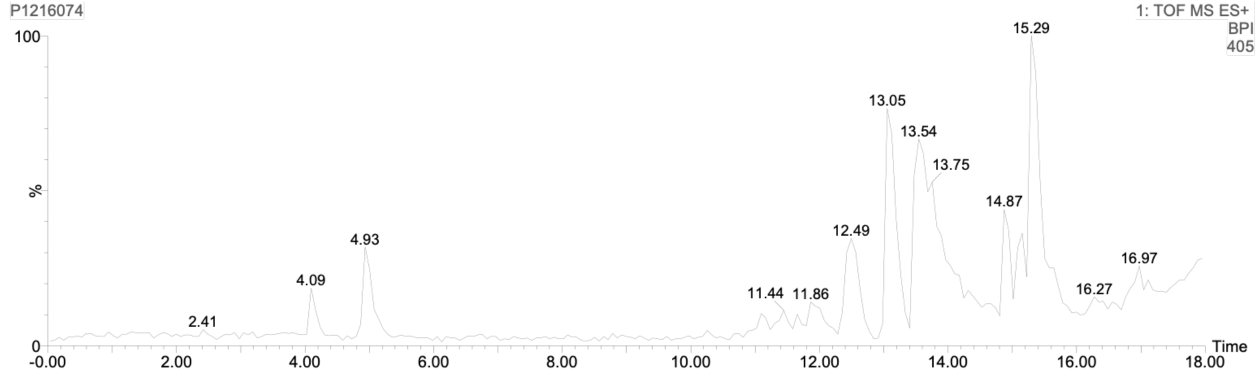

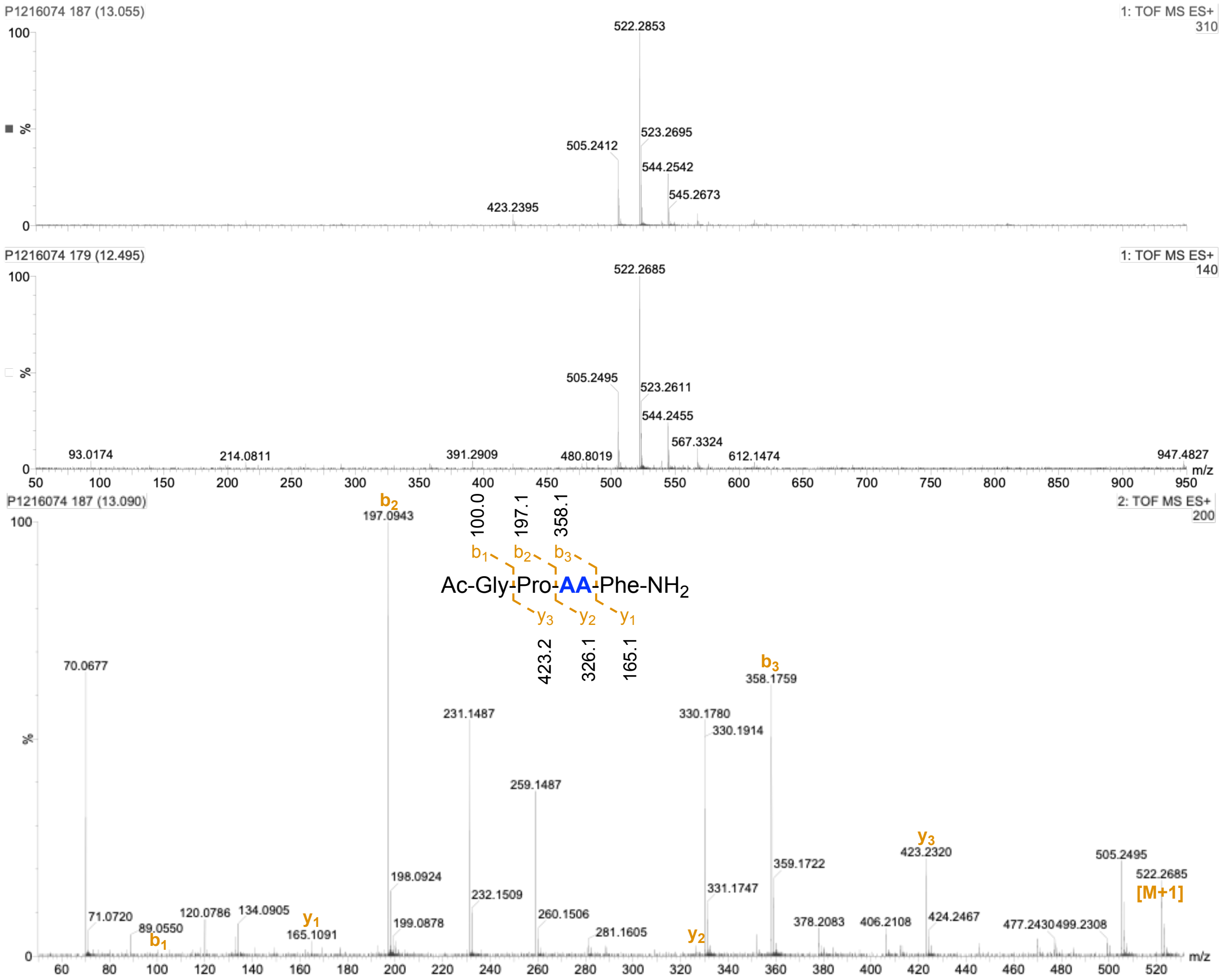
P1216074 187 (13.055)
1: TOF MS ES+
310
522.2853
523.2695
505.2412
544.2542
545.2673
423.2395
P1216074 179 (12.495)
1: TOF MS ES+
140
522.2685
505.2495
523.2611
544.2455
567.3324
93.0174
214.0811
391.2909
480.8019
612.1474
947.4827
m/z
P1216074 187 (13.090)
2: TOF MS ES+
200
b2
197.0943
100.0
197.1
358.1
b1
b2
b3
Ac-Gly-Pro-AA-Phe-NH2
y3
y2
y1
423.2
326.1
165.1
70.0677
b3
358.1759
231.1487
330.1780
330.1914
259.1487
y3
423.2320
505.2495
359.1722
522.2685
198.0924
[M+1]
331.1747
232.1509
71.0720
120.0786
134.0905
y1
260.1506
378.2083
406.2108
424.2467
89.0550
b1
165.1091
199.0878
281.1605
y2
477.2430
499.2308
m/z
%

3G': MW = 573.6, Purity = 76.9%, Yield = 10.0% [0.13 mg]

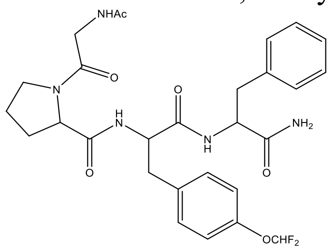

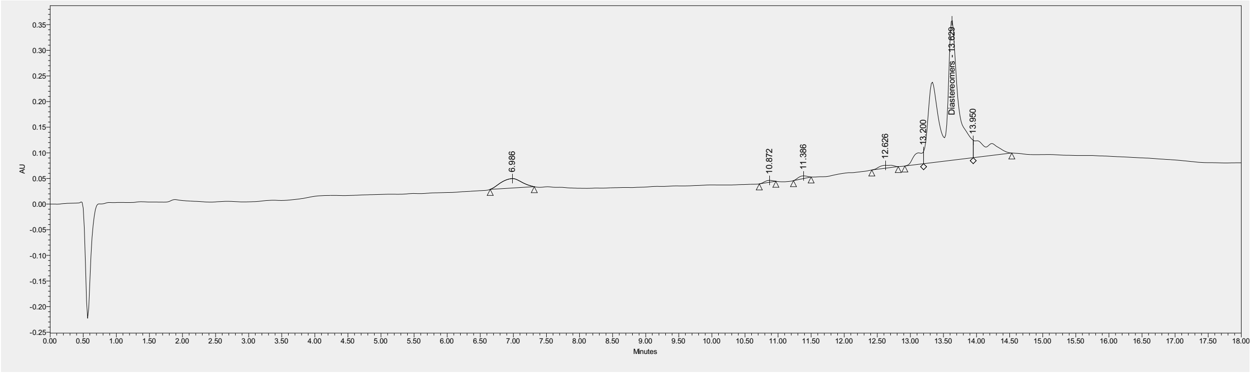

	Name	Retention Time	Area	% Area
1		6.986	402354	6.64
2		10.872	32296	0.53
3		11.386	50046	0.83
4		12.626	79204	1.31
5		13.200	231030	3.81
6	Diastereomers	13.629	4656666	76.86
7		13.950	607219	10.02

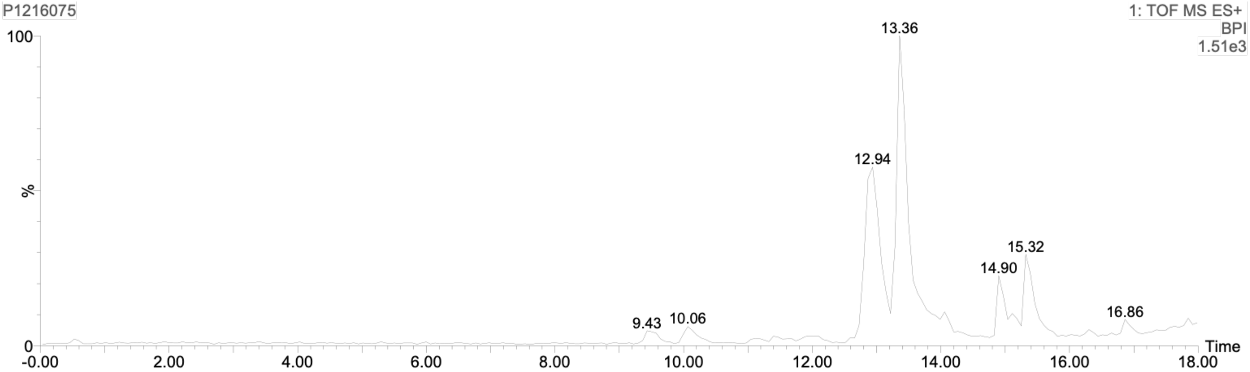

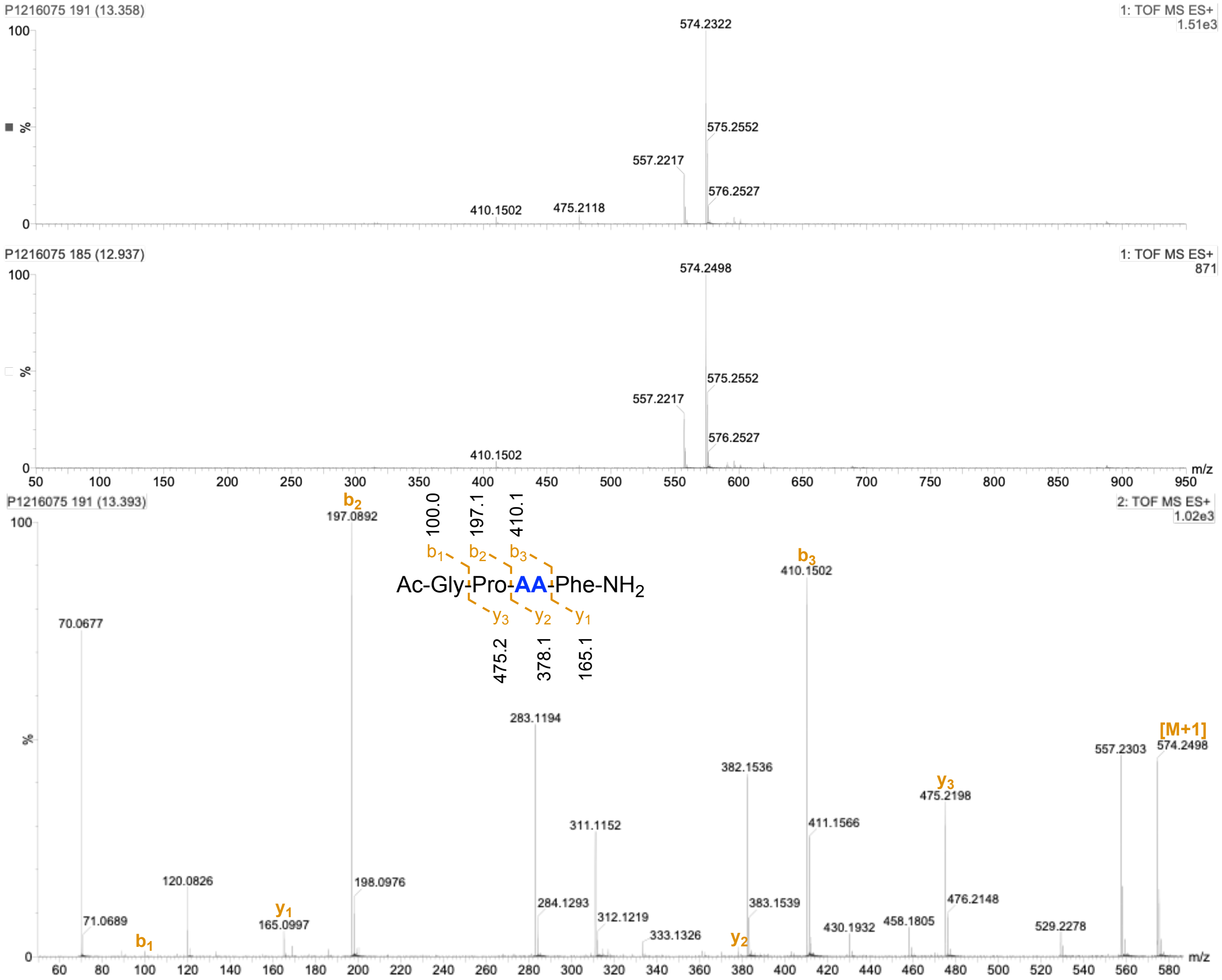

P1216075 191 (13.358)
1: TOF MS ES+
1.51e3
574.2322
575.2552
557.2217
576.2527
410.1502
475.2118
P1216075 185 (12.937)
1: TOF MS ES+
871
574.2498
575.2552
557.2217
576.2527
410.1502
m/z
P1216075 191 (13.393)
2: TOF MS ES+
1.02e3
Ac-Gly-Pro-AA-Phe-NH2
100.0
197.1
410.1
475.2
378.1
165.1
b2
197.0892
b3
410.1502
70.0677
283.1194
[M+1]
574.2498
557.2303
382.1536
y3
475.2198
311.1152
411.1566
120.0826
198.0976
71.0689
y1
165.0997
b1
284.1293
312.1219
333.1326
y2
383.1539
430.1932
458.1805
476.2148
529.2278
m/z

4G': MW = 555.6, Purity = 71.7%, Yield = 11.5% [0.15 mg]

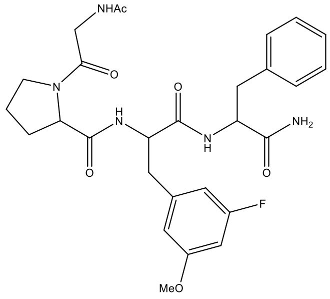

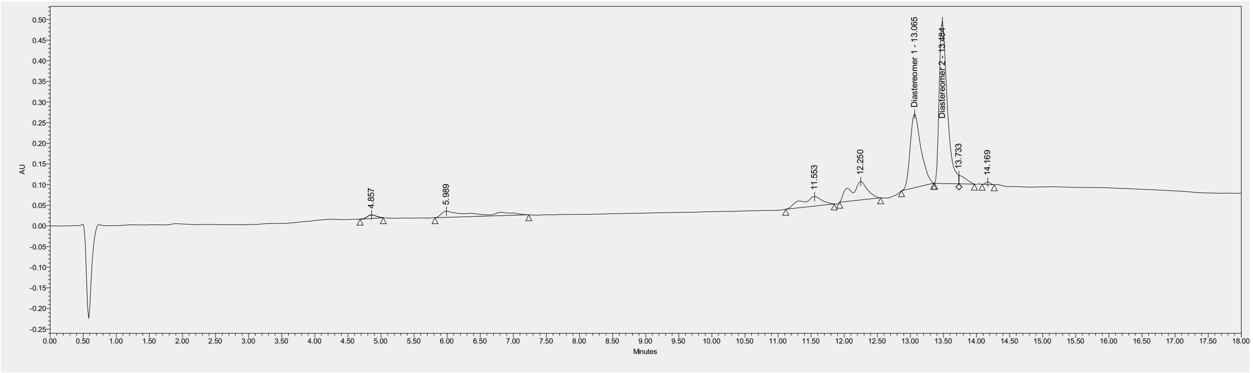

	Name	Retention Time	Area	% Area
1		4.857	80885	1.09
2		5.989	531549	7.14
3		11.553	512758	6.89
4		12.250	804752	10.81
5	Diastereomer 1	13.065	2017411	27.09
6	Diastereomer 2	13.484	3319915	44.58
7		13.733	148709	2.00
8		14.169	30776	0.41

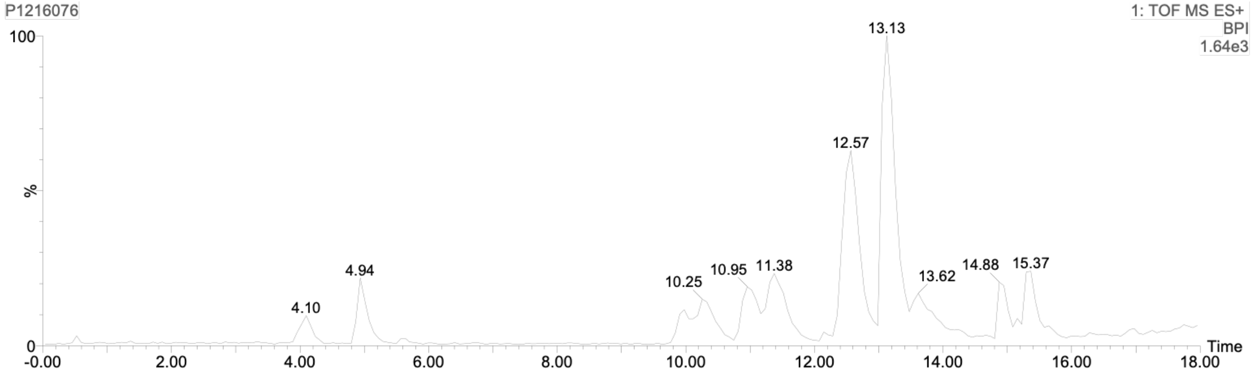

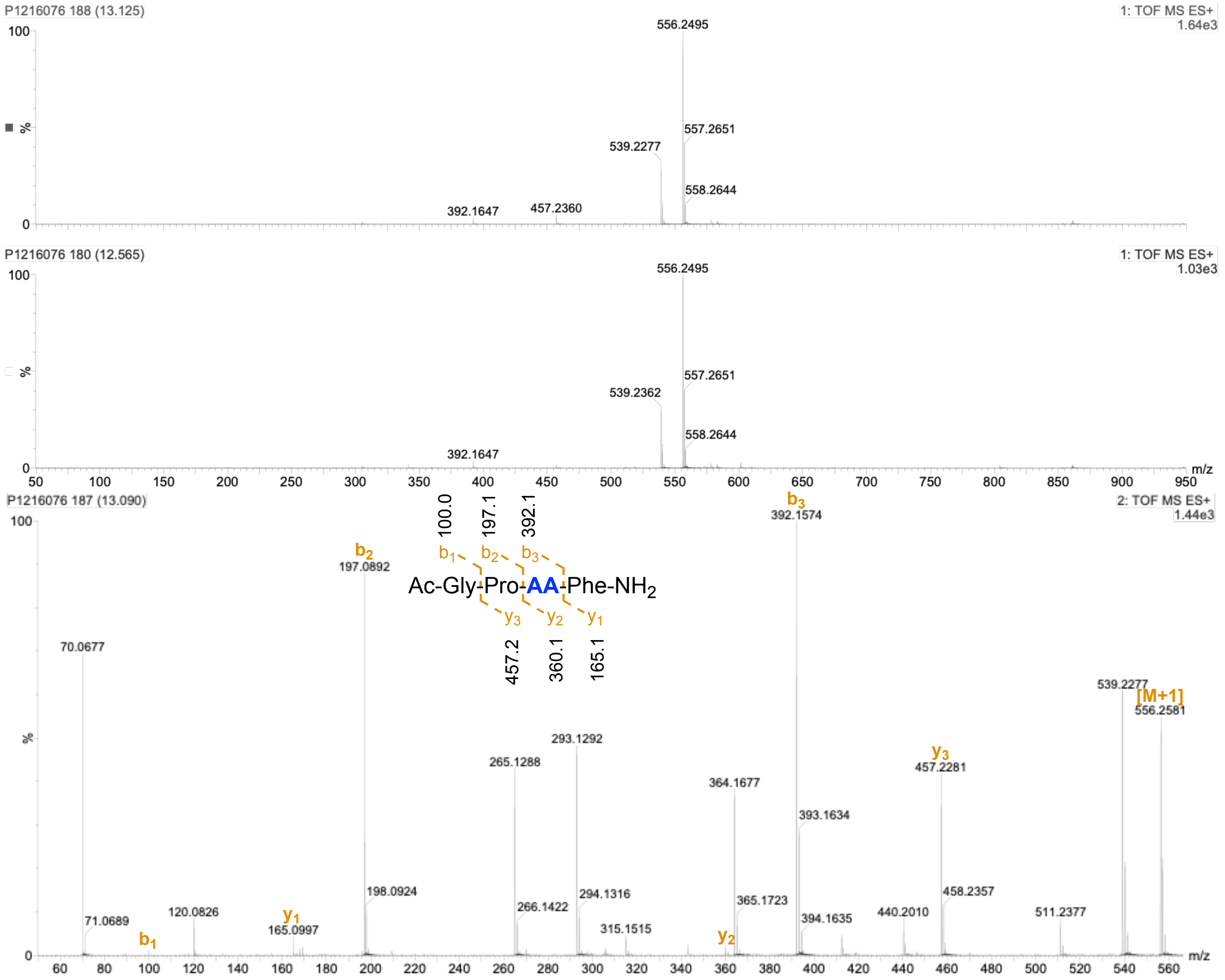
P1216076 188 (13.125)
1: TOF MS ES+
1.64e3
556.2495
557.2651
539.2277
558.2644
392.1647
457.2360
P1216076 180 (12.565)
1: TOF MS ES+
1.03e3
556.2495
557.2651
539.2362
558.2644
392.1647
m/z
P1216076 187 (13.090)
2: TOF MS ES+
1.44e3
Ac-Gly-Pro-AA-Phe-NH2
100.0
197.1
392.1
457.2
360.1
165.1
b2 197.0892
b3 392.1574
70.0677
293.1292
265.1288
364.1677
393.1634
y3 457.2281
539.2277
[M+1] 556.2581
198.0924
294.1316
266.1422
365.1723
458.2357
71.0689
120.0826
y1 165.0997
b1
315.1515
y2
394.1635
440.2010
511.2377
m/z

5G’: MW = 553.7, Purity = 82.4%, Yield = 35.2% [0.45 mg]

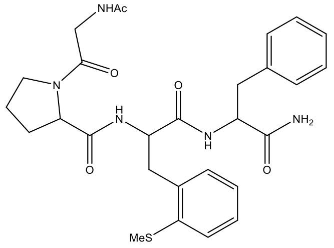

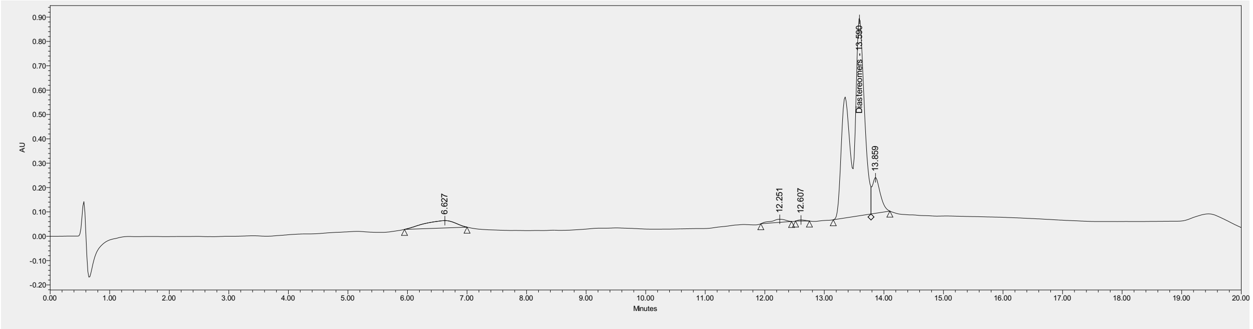

	Name	Retention Time	Area	% Area
1		6.627	1125799	7.09
2		12.251	220958	1.39
3		12.607	38009	0.24
4	Diastereomers	13.590	13083460	82.40
5		13.859	1409523	8.88

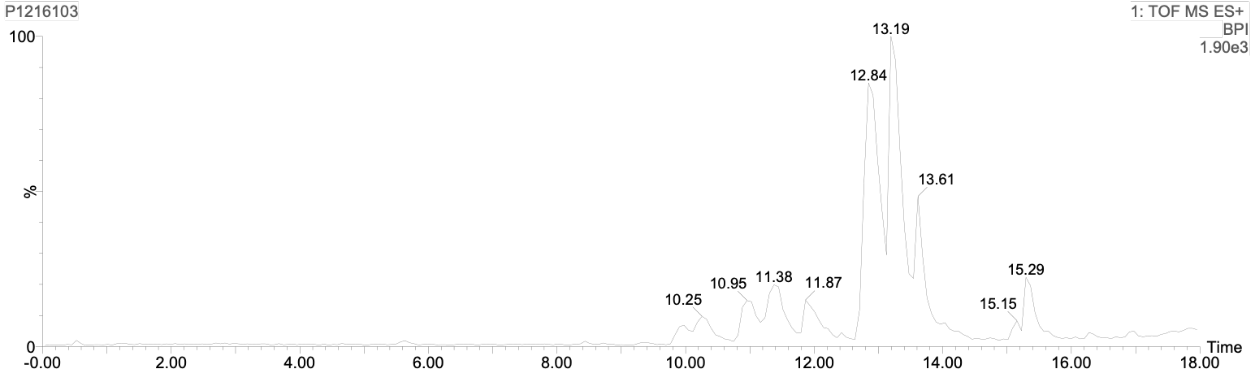

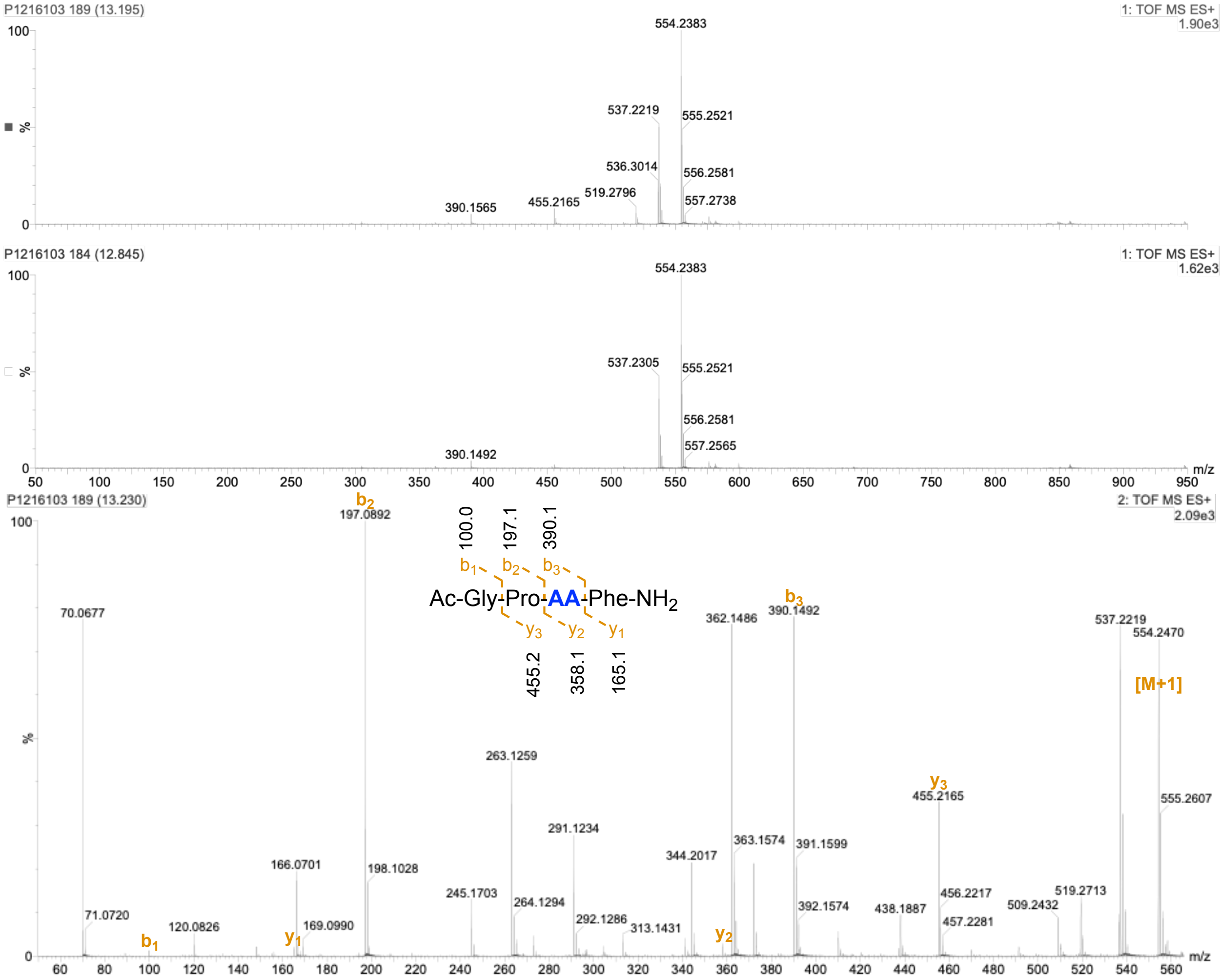
P1216103 189 (13.195)
1: TOF MS ES+
1.90e3
554.2383
537.2219
555.2521
536.3014
556.2581
519.2796
557.2738
455.2165
390.1565
P1216103 184 (12.845)
1: TOF MS ES+
1.62e3
554.2383
537.2305
555.2521
556.2581
557.2565
390.1492
m/z
P1216103 189 (13.230)
2: TOF MS ES+
2.09e3
b2
197.0892
100.0
197.1
390.1
Ac-Gly-Pro-AA-Phe-NH2
455.2
358.1
165.1
70.0677
362.1486
b3
390.1492
537.2219
554.2470
[M+1]
263.1259
y3
455.2165
555.2607
291.1234
363.1574
391.1599
344.2017
166.0701
198.1028
245.1703
264.1294
456.2217
519.2713
392.1574
438.1887
509.2432
71.0720
120.0826
169.0990
292.1286
313.1431
457.2281
b1
y1
y2
%

6G': MW = 633.7, Purity = 86.7%, Yield = 17.8% [0.26 mg]

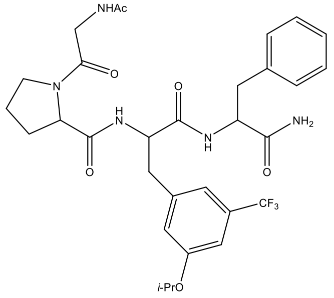

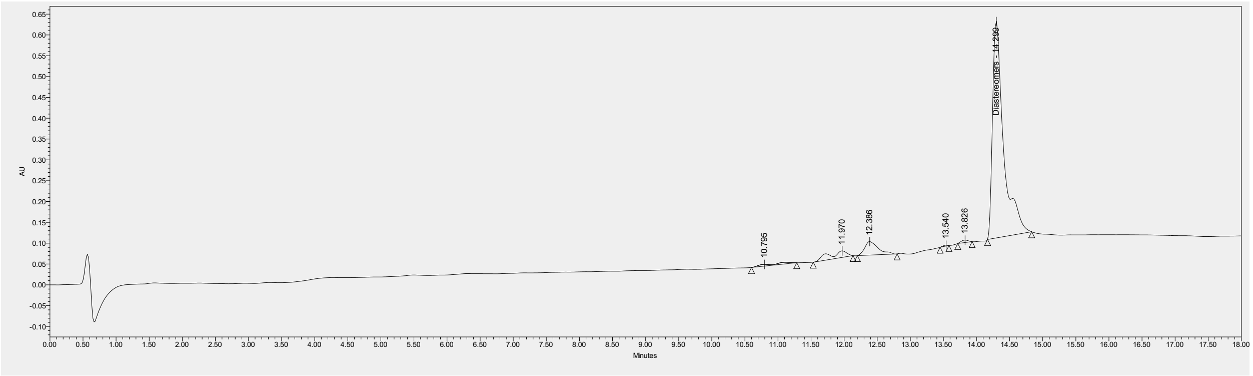

	Name	Retention Time	Area	% Area
1		10.795	104645	1.51
2		11.970	307900	4.44
3		12.386	458808	6.61
4		13.540	10775	0.16
5		13.826	43811	0.63
6	Diastereomers	14.299	6010272	86.65

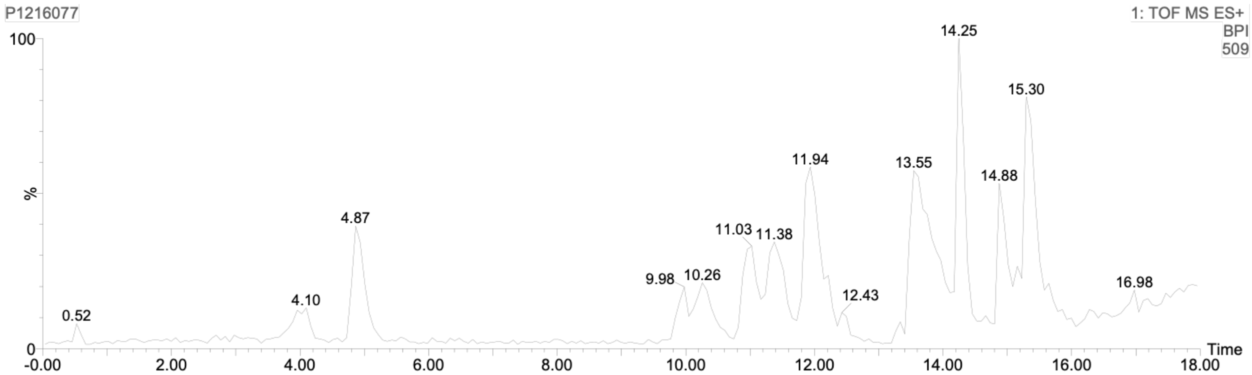

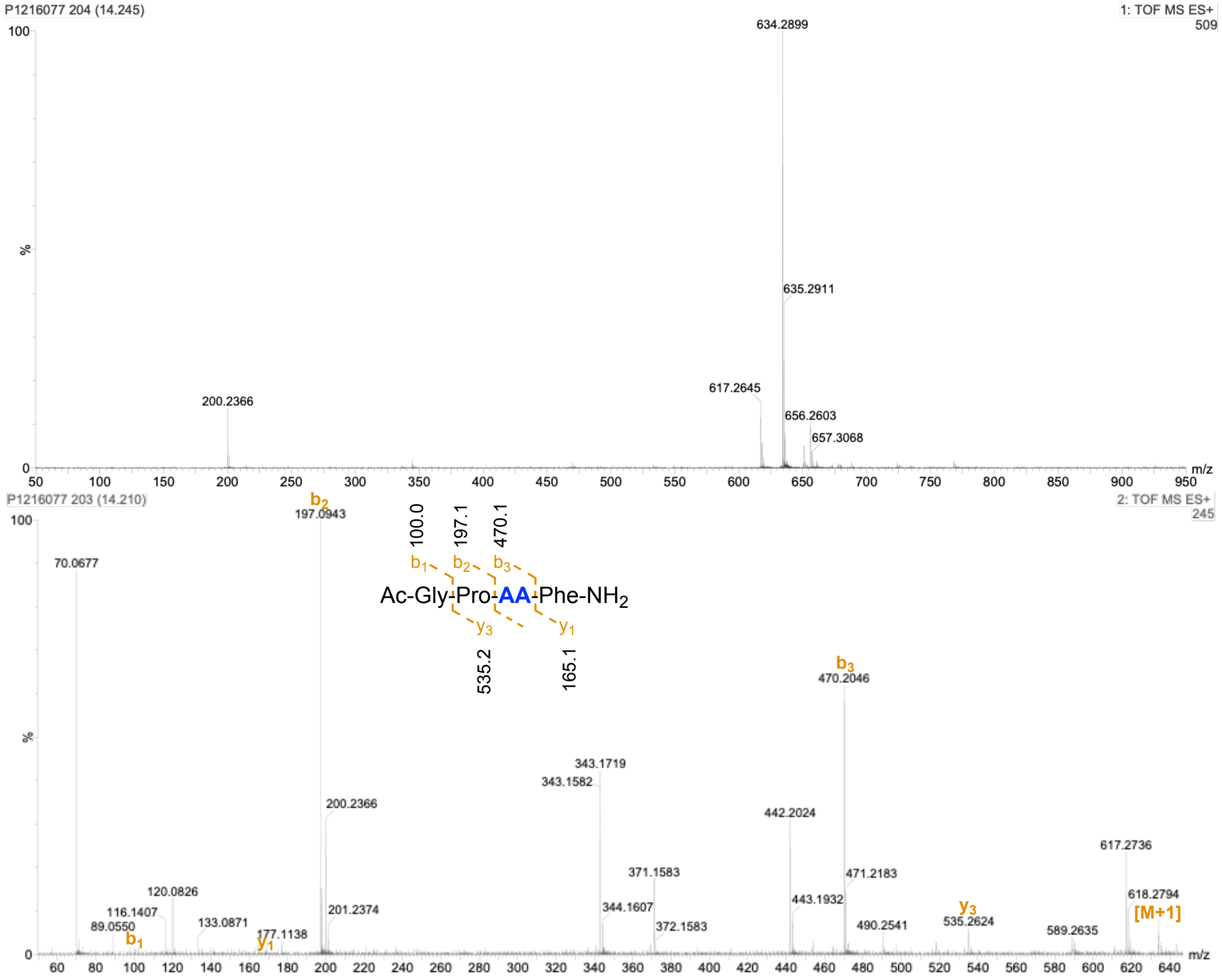
P1216077 204 (14.245)
1: TOF MS ES+
509
634.2899
635.2911
617.2645
200.2366
656.2603
657.3068
m/z
P1216077 203 (14.210)
2: TOF MS ES+
245
b2
197.0943
100.0
197.1
470.1
b1 b2 b3
Ac-Gly-Pro-AA-Phe-NH2
y3 y1
535.2
165.1
70.0677
b3
470.2046
343.1719
343.1582
200.2366
442.2024
617.2736
371.1583
471.2183
120.0826
618.2794
116.1407
344.1607
443.1932
[M+1]
89.0550
133.0871
201.2374
372.1583
490.2541
y3
535.2624
589.2635
b1
y1
177.1138
m/z

7G’: MW = 581.6, Purity = 71.2%, Yield = 4.2% [0.056 mg]

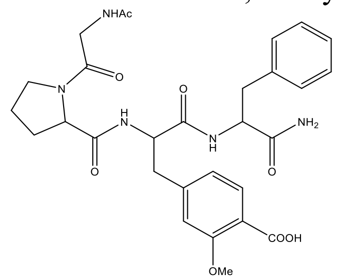

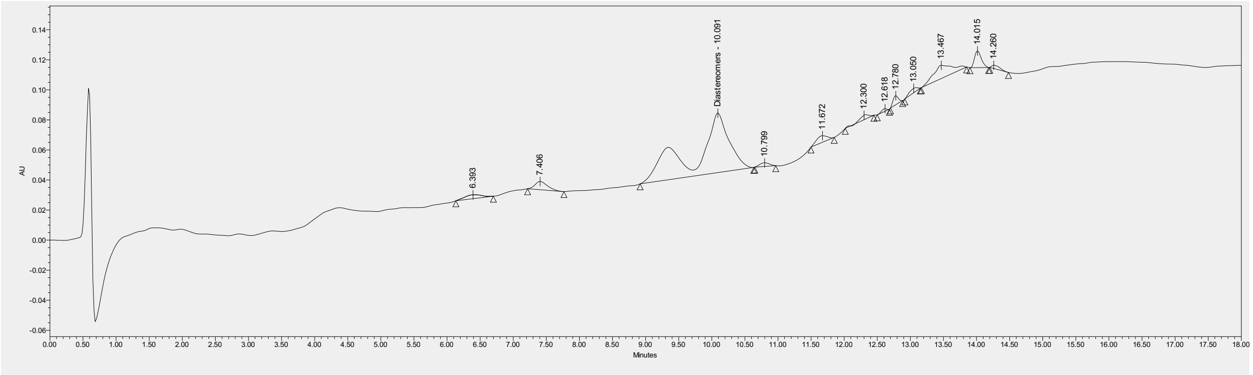

	Name	Retention Time	Area	% Area
1		6.393	46395	2.35
2		7.406	72858	3.68
3	Diastereomers	10.091	1408601	71.22
4		10.799	24918	1.26
5		11.672	50999	2.58
6		12.300	30770	1.56
7		12.618	9877	0.50
8		12.780	33803	1.71
9		13.050	25043	1.27
10		13.467	175686	8.88
11		14.015	79714	4.03
12		14.260	19027	0.96

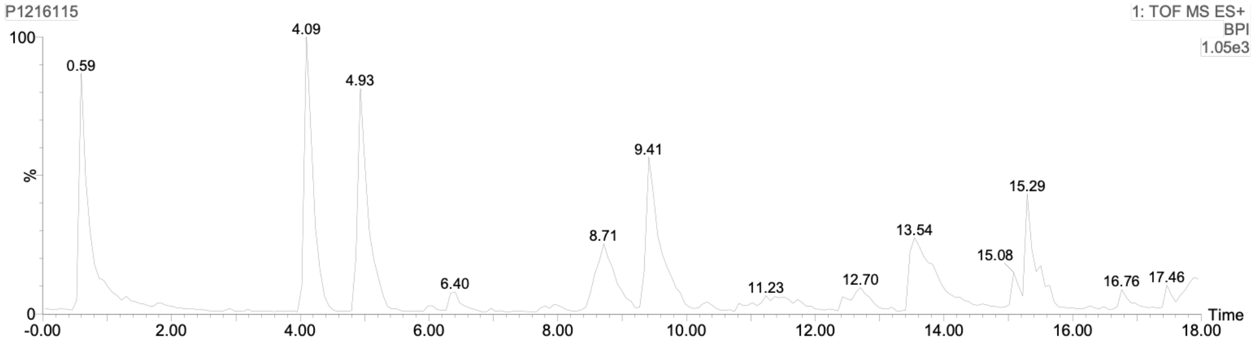

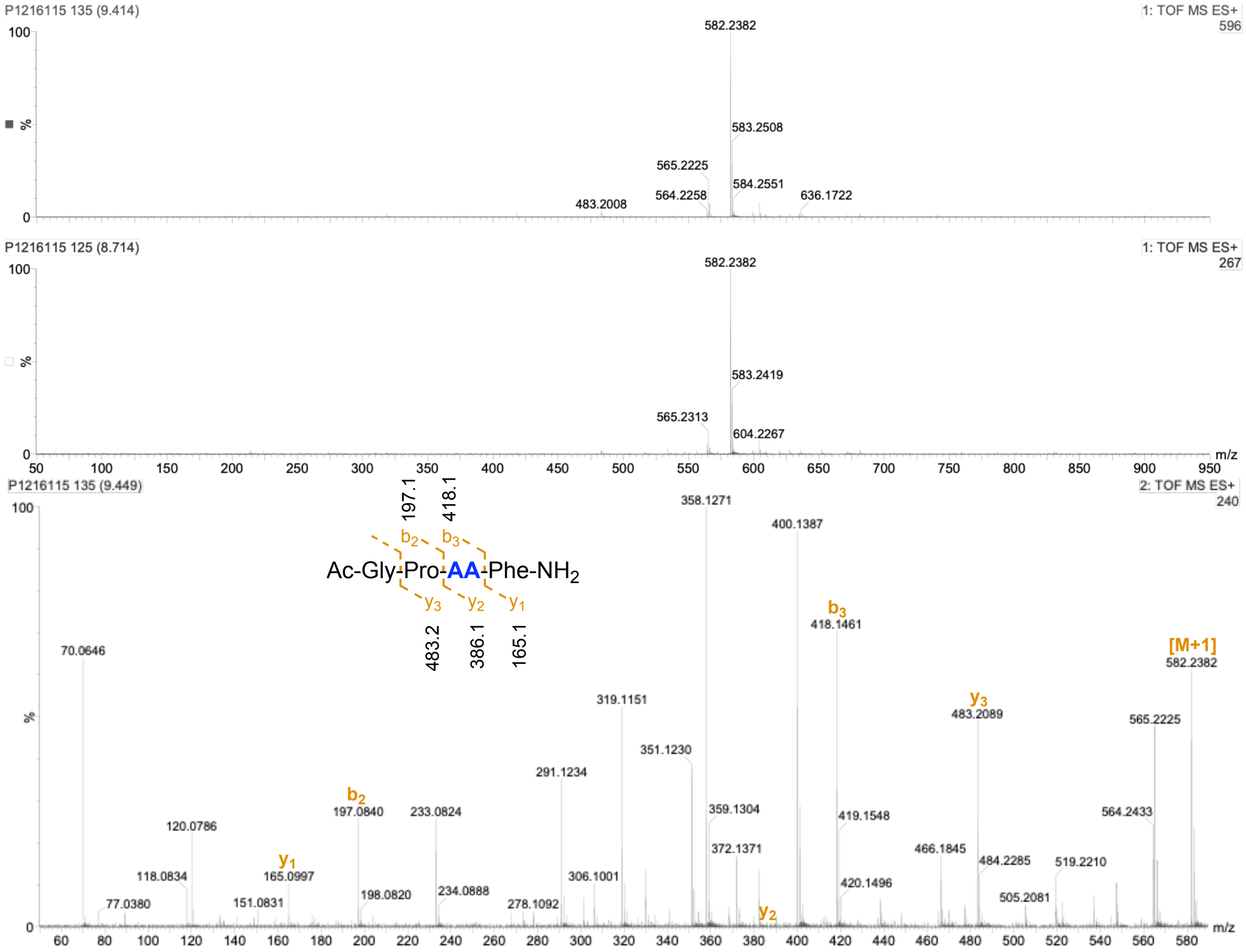

P1216115 135 (9.414)
1: TOF MS ES+
596
582.2382
583.2508
565.2225
584.2551
483.2008
564.2258
636.1722
P1216115 125 (8.714)
1: TOF MS ES+
267
582.2382
583.2419
565.2313
604.2267
m/z
P1216115 135 (9.449)
2: TOF MS ES+
240
Ac-Gly-Pro-AA-Phe-NH2
197.1
418.1
483.2
386.1
165.1
358.1271
400.1387
b3
418.1461
[M+1]
582.2382
70.0646
319.1151
y3
483.2089
565.2225
351.1230
291.1234
b2
197.0840
233.0824
359.1304
419.1548
120.0786
564.2433
372.1371
466.1845
y1
484.2285
118.0834
165.0997
306.1001
519.2210
420.1496
198.0820
234.0888
505.2081
77.0380
151.0831
278.1092
y2

8G’: MW = 535.6, Purity = 80.6%, Yield = 12.7% [0.16 mg]

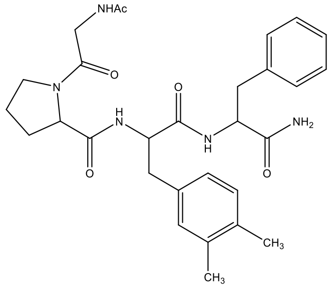

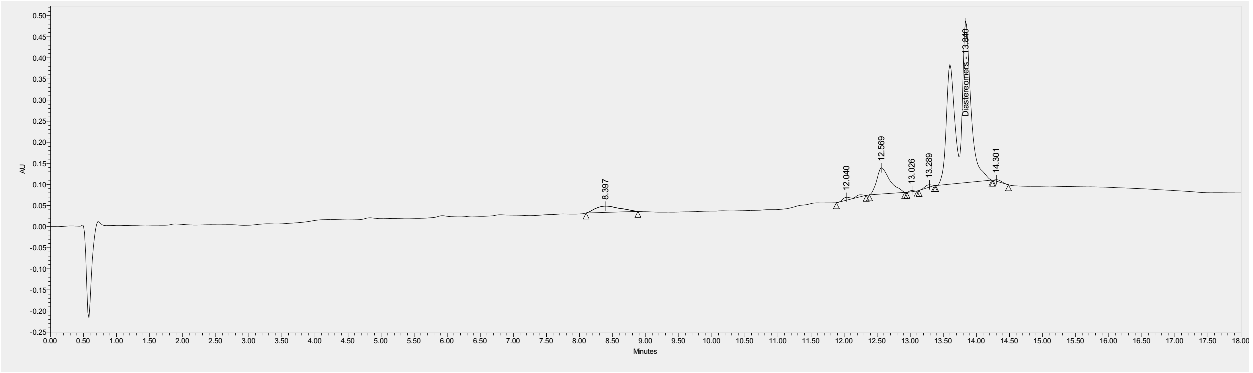

	Name	Retention Time	Area	% Area
1		8.397	389218	5.32
2		12.040	88768	1.21
3		12.569	853896	11.67
4		13.026	13814	0.19
5		13.289	42808	0.59
6	Diastereomers	13.840	5900062	80.63
7		14.301	29067	0.40

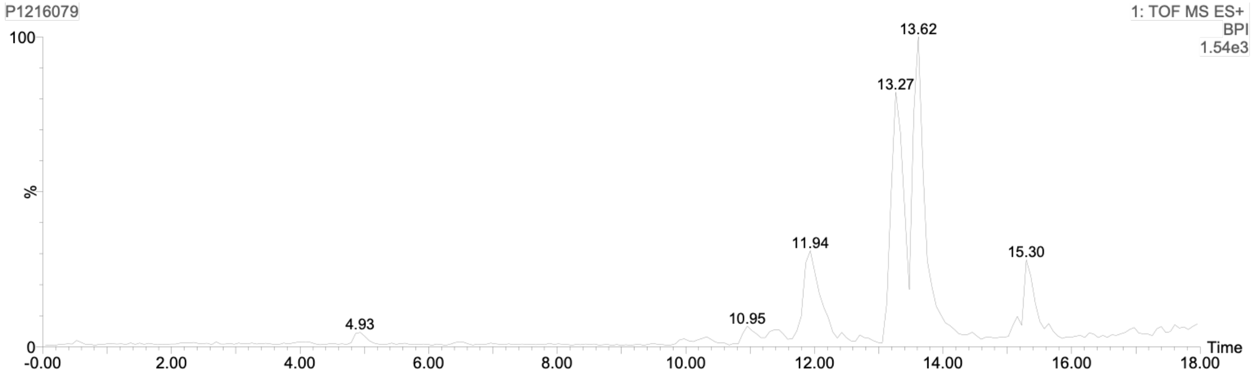

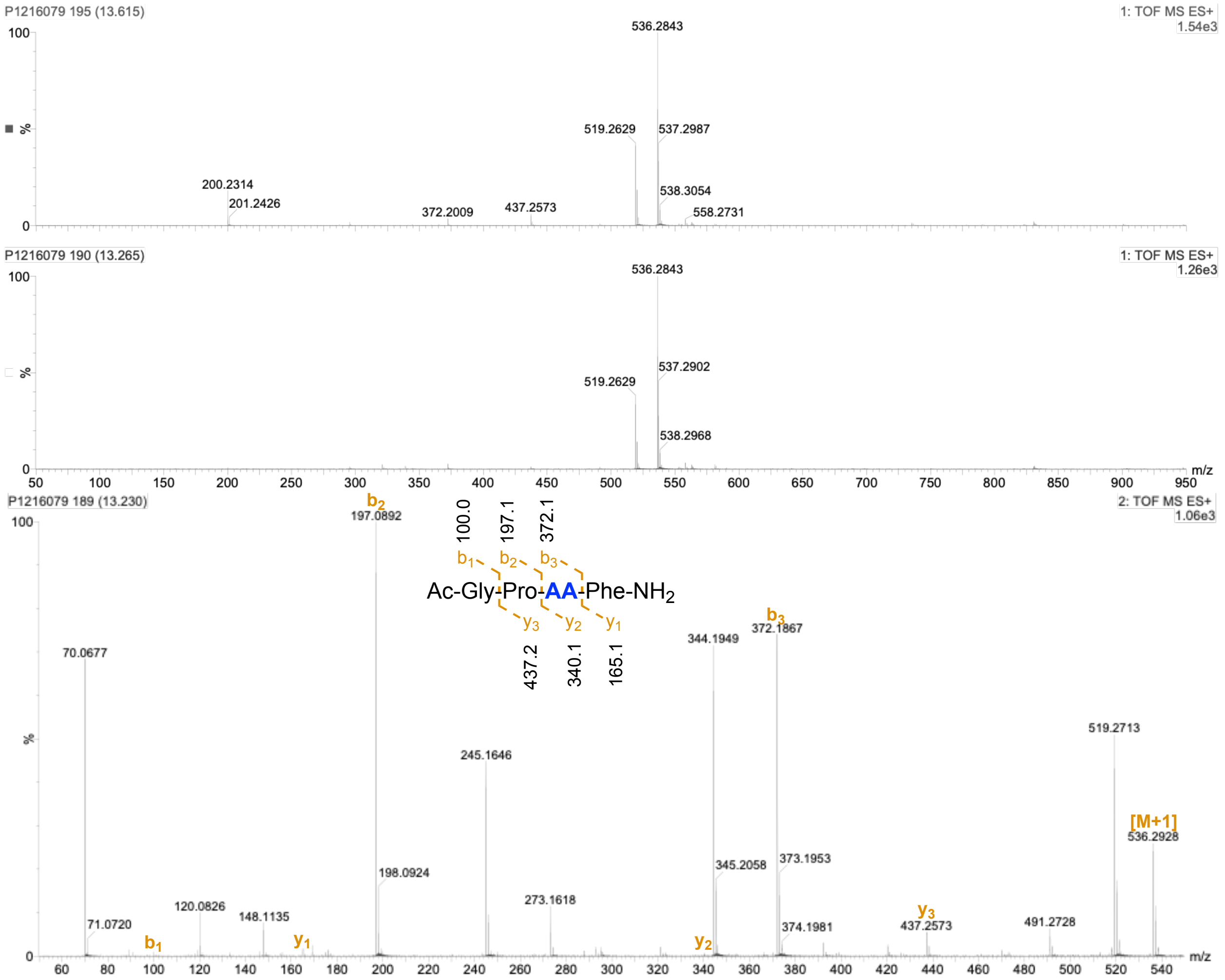
P1216079 195 (13.615)
1: TOF MS ES+
1.54e3
536.2843
519.2629
537.2987
200.2314
201.2426
538.3054
372.2009
437.2573
558.2731
P1216079 190 (13.265)
1: TOF MS ES+
1.26e3
536.2843
537.2902
519.2629
538.2968
m/z
P1216079 189 (13.230)
2: TOF MS ES+
1.06e3
b2
197.0892
100.0
197.1
372.1
Ac-Gly-Pro-AA-Phe-NH2
437.2
340.1
165.1
70.0677
344.1949
b3
372.1867
519.2713
245.1646
[M+1]
536.2928
198.0924
345.2058
373.1953
120.0826
148.1135
273.1618
y3
437.2573
491.2728
71.0720
b1
y1
y2
374.1981
m/z

9G’: MW = 551.6, Purity = 67.7%, Yield = 10.4% [0.13 mg]

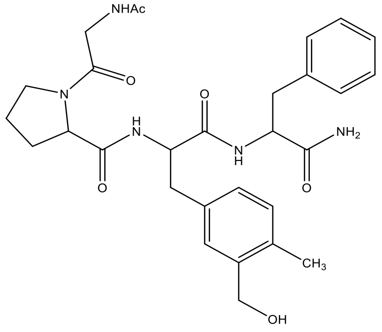

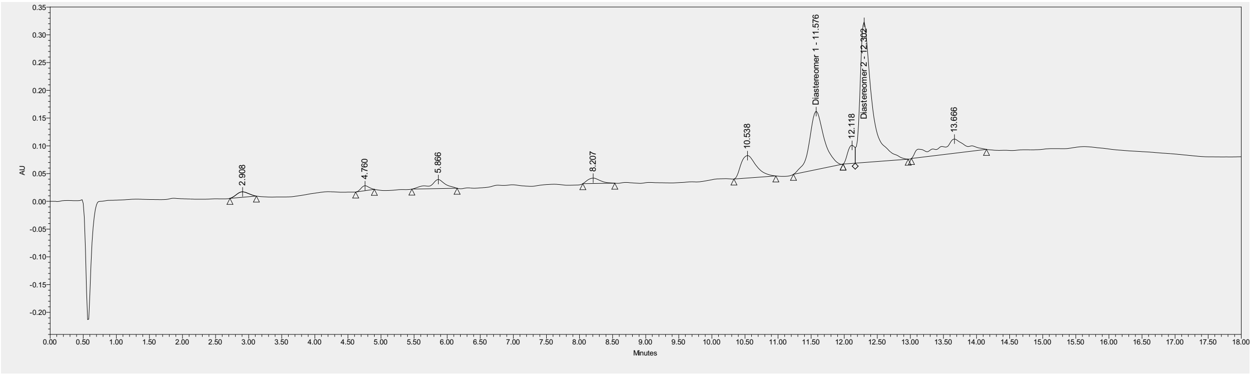

	Name	Retention Time	Area	% Area
1		2.908	123856	1.74
2		4.760	74142	1.04
3		5.866	261269	3.67
4		8.207	129627	1.82
5		10.538	654864	9.20
6	Diastereomer 1	11.576	1641749	23.06
7		12.118	234860	3.30
8	Diastereomer 2	12.302	3179205	44.66
9		13.666	818575	11.50

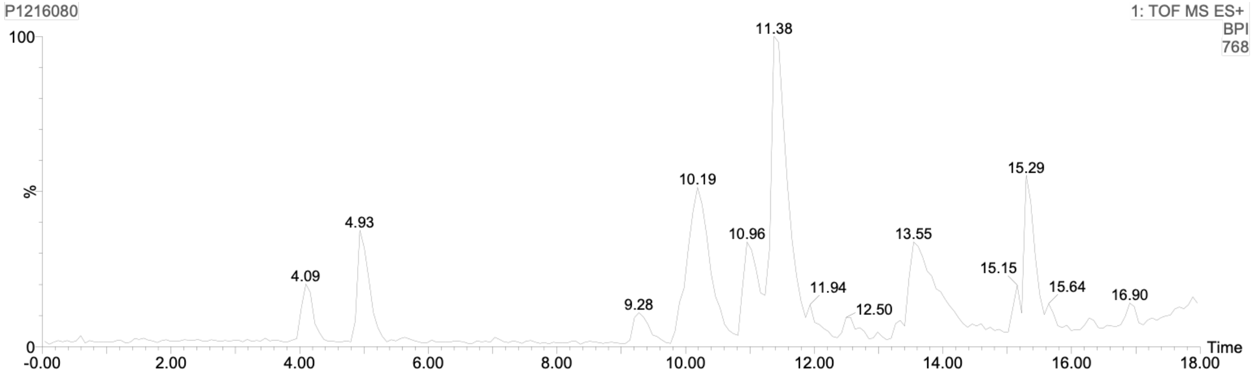

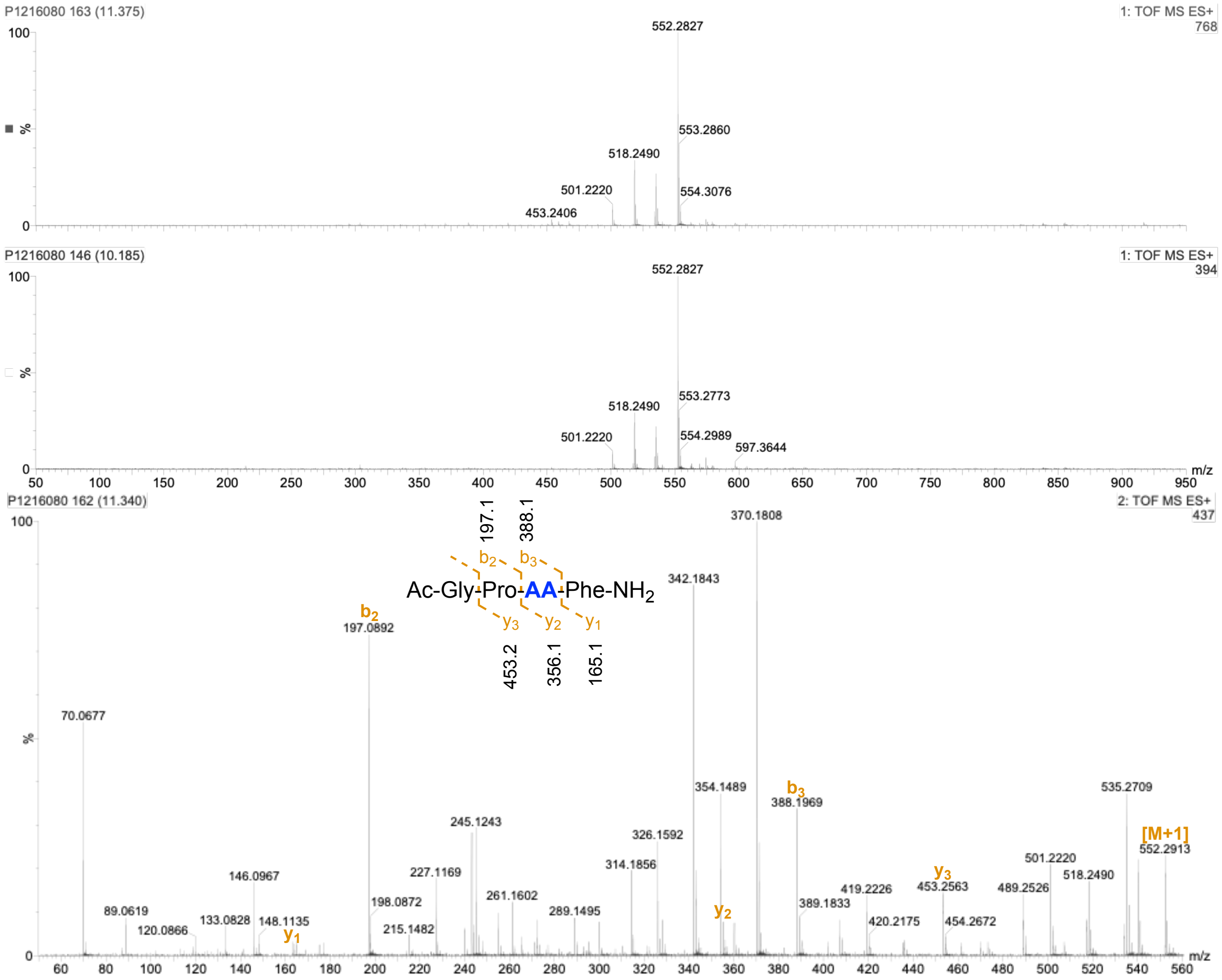
P1216080 163 (11.375)
1: TOF MS ES+
768
552.2827
553.2860
518.2490
501.2220
554.3076
453.2406
P1216080 146 (10.185)
1: TOF MS ES+
394
552.2827
553.2773
518.2490
501.2220
554.2989
597.3644
P1216080 162 (11.340)
2: TOF MS ES+
437
Ac-Gly-Pro-AA-Phe-NH2
197.1
388.1
453.2
356.1
165.1
370.1808
342.1843
b2
197.0892
70.0677
354.1489
b3
388.1969
535.2709
[M+1]
552.2913
245.1243
326.1592
501.2220
y3
453.2563
518.2490
314.1856
419.2226
489.2526
146.0967
227.1169
261.1602
y2
389.1833
89.0619
133.0828
148.1135
198.0872
289.1495
120.0866
y1
215.1482
420.2175
454.2672
m/z

10G': MW = 572.7, Purity = 66.2%, Yield = 7.1% [0.095 mg]

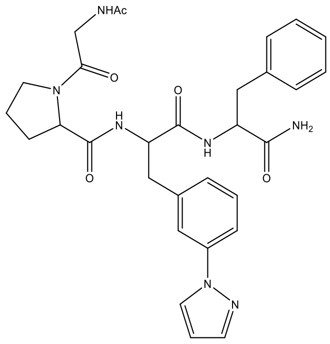

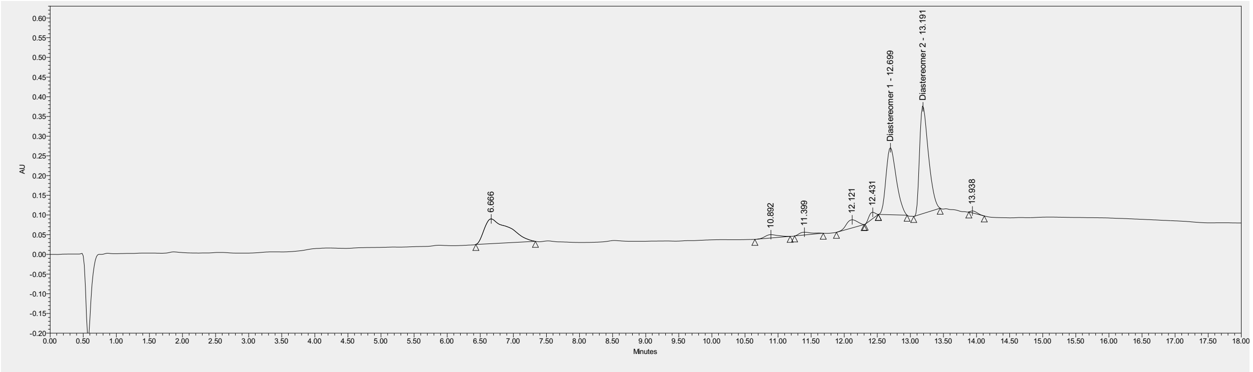

	Name	Retention Time	Area	% Area
1		6.666	1639589	24.72
2		10.892	117137	1.77
3		11.399	93340	1.41
4		12.121	243962	3.68
5		12.431	111953	1.69
6	Diastereomer 1	12.699	1834583	27.66
7	Diastereomer 2	13.191	2557181	38.55
8		13.938	35747	0.54

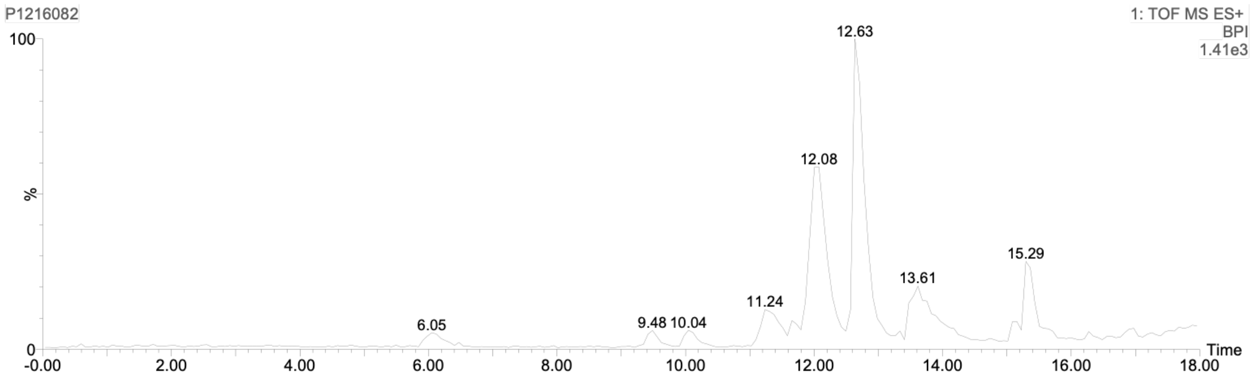

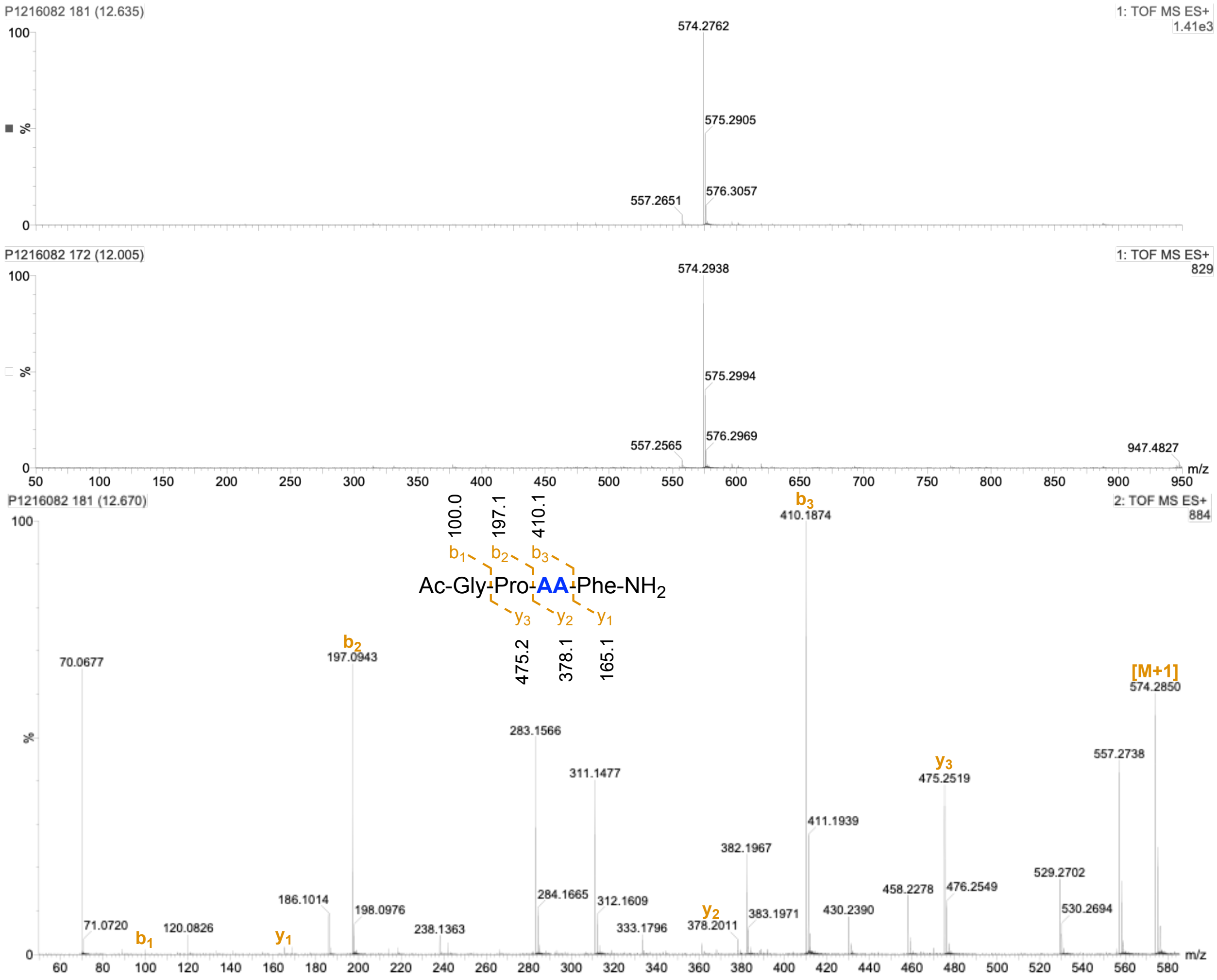
P1216082 181 (12.635)
1: TOF MS ES+
1.41e3
574.2762
575.2905
557.2651
576.3057
P1216082 172 (12.005)
1: TOF MS ES+
829
574.2938
575.2994
557.2565
576.2969
947.4827
m/z
P1216082 181 (12.670)
2: TOF MS ES+
884
Ac-Gly-Pro-AA-Phe-NH2
100.0
197.1
410.1
475.2
378.1
165.1
b3
410.1874
b2
197.0943
70.0677
[M+1]
574.2850
283.1566
311.1477
y3
475.2519
557.2738
411.1939
382.1967
458.2278
476.2549
529.2702
186.1014
198.0976
284.1665
312.1609
530.2694
y2
378.2011
383.1971
430.2390
71.0720
120.0826
238.1363
333.1796
b1
y1
m/z

11G': MW = 565.7, Purity = 78.9%, Yield = 17.3% [0.23 mg]

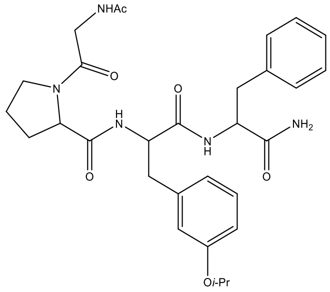

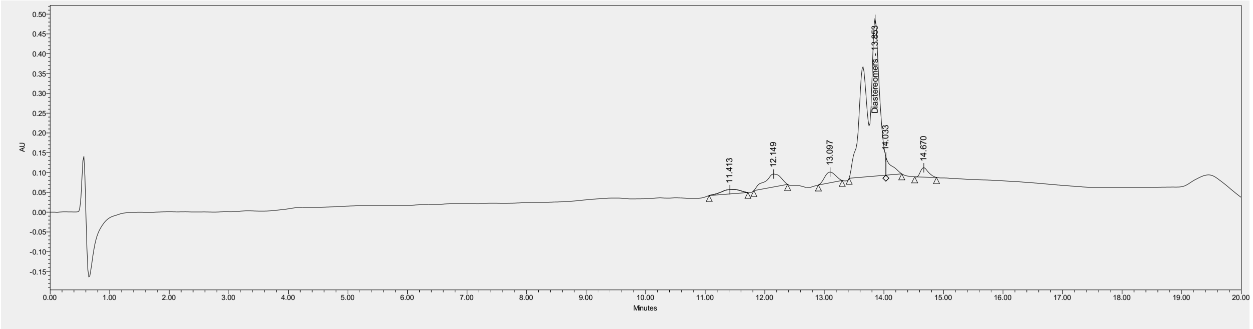

	Name	Retention Time	Area	% Area
1		11.413	239892	2.94
2		12.149	626876	7.68
3		13.097	342532	4.20
4	Diastereomers	13.853	6441616	78.92
5		14.033	296190	3.63
6		14.670	215468	2.64

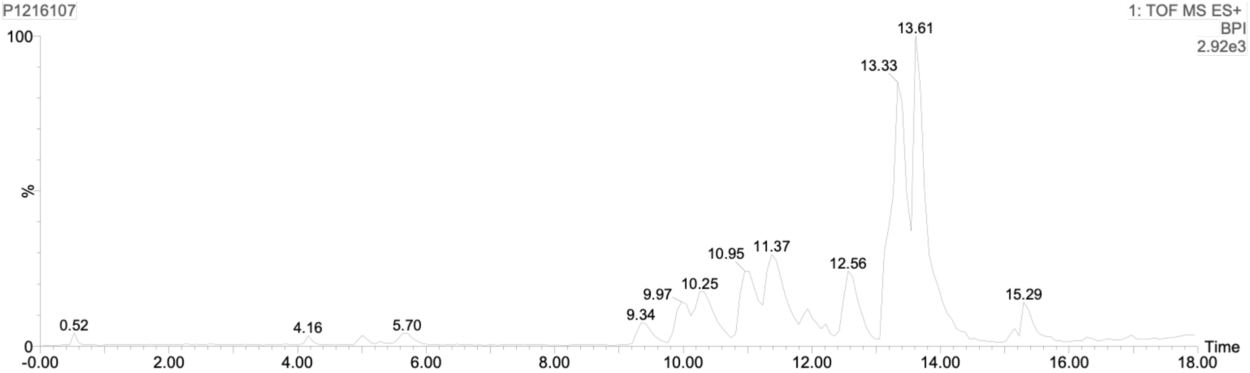

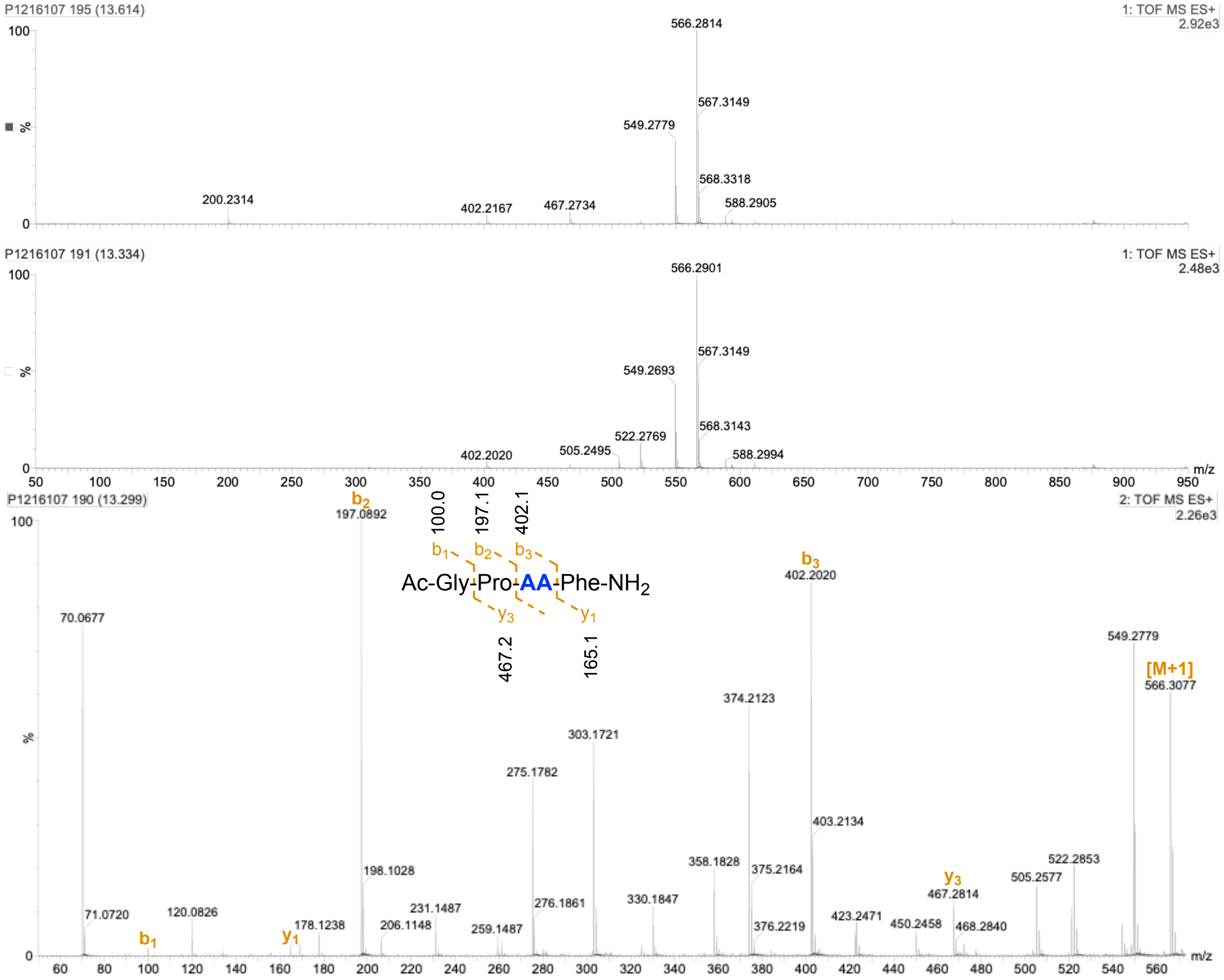
P1216107 195 (13.614)
1: TOF MS ES+
2.92e3
566.2814
567.3149
549.2779
568.3318
588.2905
200.2314
402.2167
467.2734
P1216107 191 (13.334)
1: TOF MS ES+
2.48e3
566.2901
567.3149
549.2693
568.3143
522.2769
505.2495
402.2020
588.2994
m/z
P1216107 190 (13.299)
2: TOF MS ES+
2.26e3
b2
197.0892
100.0
197.1
402.1
Ac-Gly-Pro-AA-Phe-NH2
b1 b2 b3
y3 y1
467.2
165.1
b3
402.2020
70.0677
549.2779
[M+1]
566.3077
374.2123
303.1721
275.1782
403.2134
358.1828
375.2164
198.1028
y3
467.2814
505.2577
522.2853
231.1487
276.1861
330.1847
71.0720
120.0826
b1
y1
178.1238
206.1148
259.1487
376.2219
423.2471
450.2458
468.2840
m/z

12G': MW = 581.7, Purity = 69.2%, Yield = 27.7% [0.38 mg]

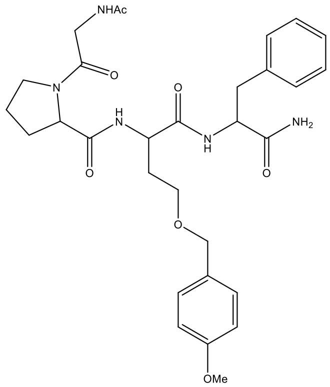

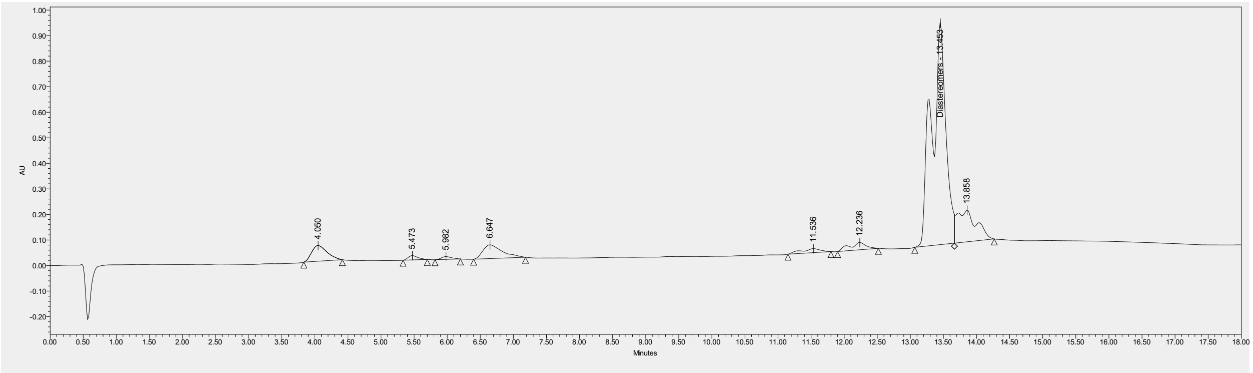

	Name	Retention Time	Area	% Area
1		4.050	1000590	5.36
2		5.473	171377	0.92
3		5.982	117155	0.63
4		6.647	1089826	5.83
5		11.536	324520	1.74
6		12.236	506107	2.71
7	Diastereomers	13.453	12923853	69.18
8		13.858	2548276	13.64

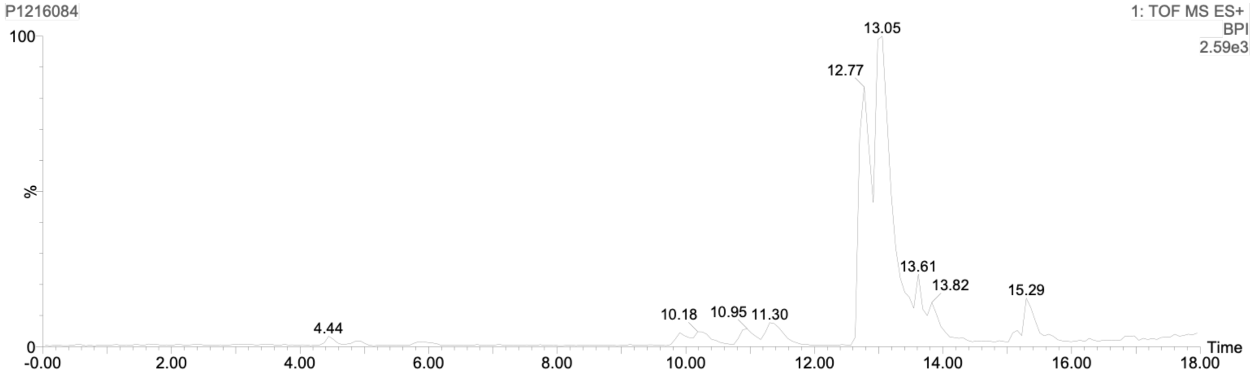

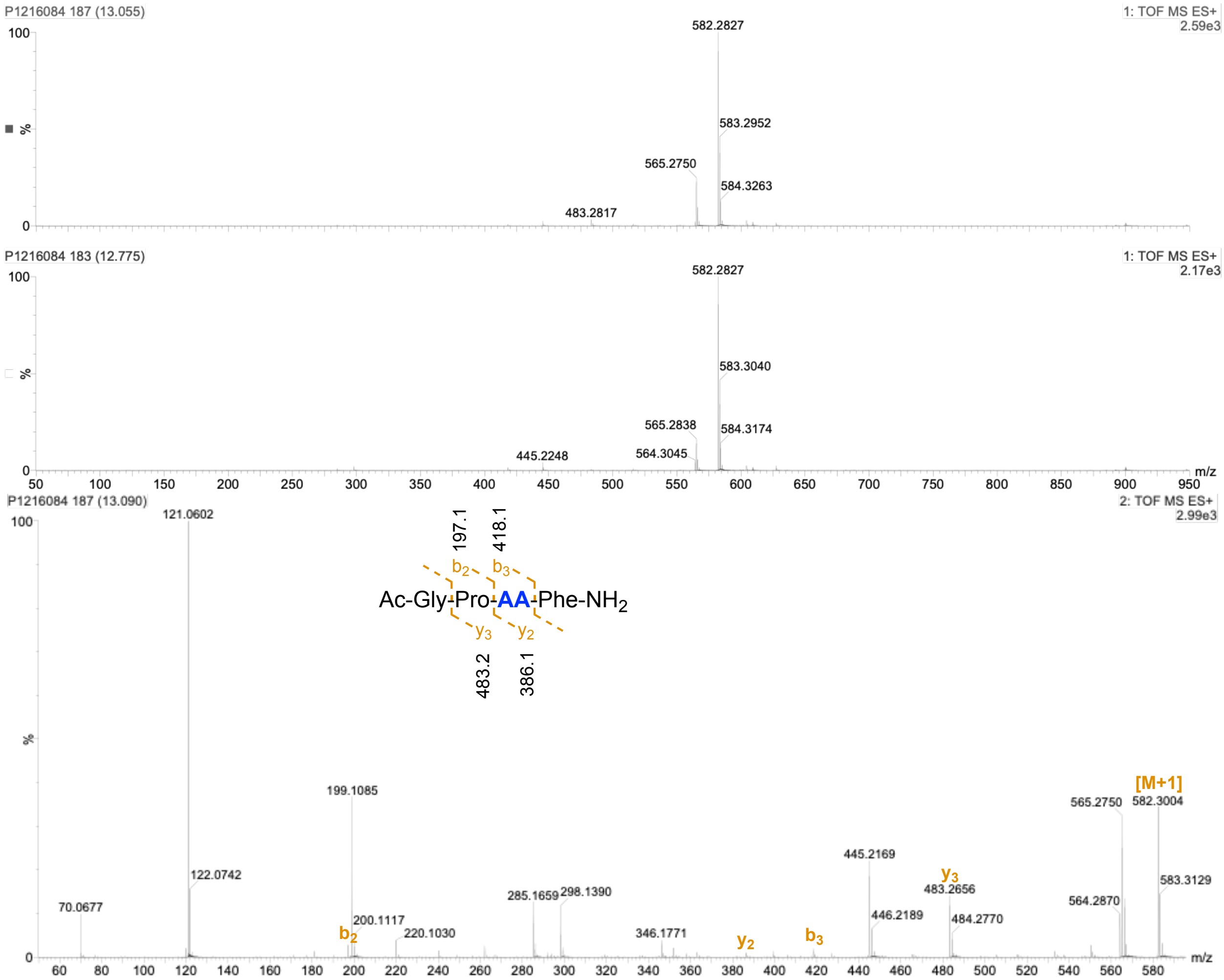

P1216084 187 (13.055)
1: TOF MS ES+
2.59e3
582.2827
583.2952
565.2750
584.3263
483.2817
P1216084 183 (12.775)
1: TOF MS ES+
2.17e3
582.2827
583.3040
565.2838
584.3174
445.2248
564.3045
m/z
P1216084 187 (13.090)
2: TOF MS ES+
2.99e3
121.0602
197.1
418.1
Ac-Gly-Pro-AA-Phe-NH2
483.2
386.1
199.1085
122.0742
70.0677
200.1117
220.1030
285.1659
298.1390
346.1771
445.2169
446.2189
483.2656
484.2770
564.2870
565.2750
[M+1]
582.3004
583.3129
m/z

1H’: MW = 529.6, Purity = 81.1%, Yield = 15.6% [0.19 mg]

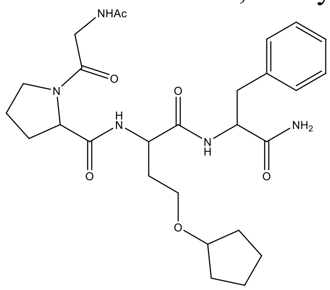

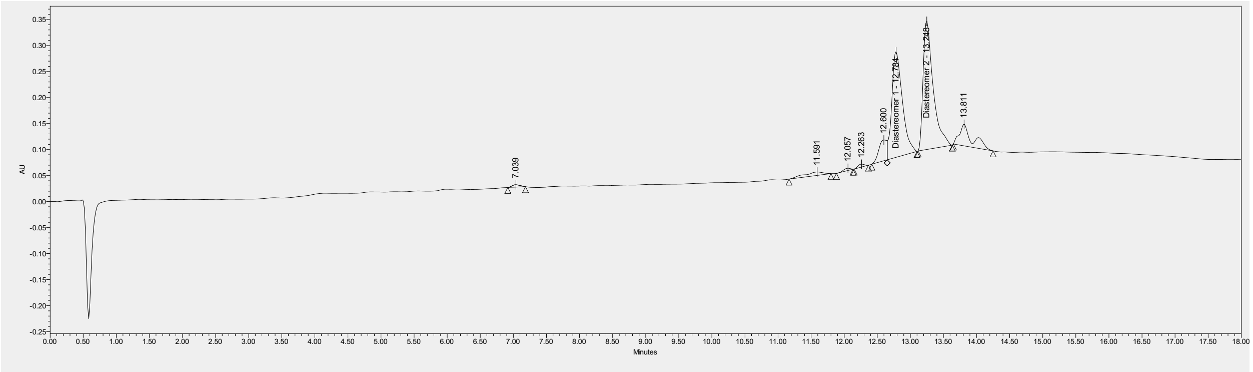

	Name	Retention Time	Area	% Area
1		7.039	37683	0.63
2		11.591	138896	2.31
3		12.057	31112	0.52
4		12.263	38015	0.63
5		12.600	332277	5.53
6	Diastereomer 1	12.784	2261479	37.66
7	Diastereomer 2	13.248	2609071	43.45
8		13.811	556291	9.26

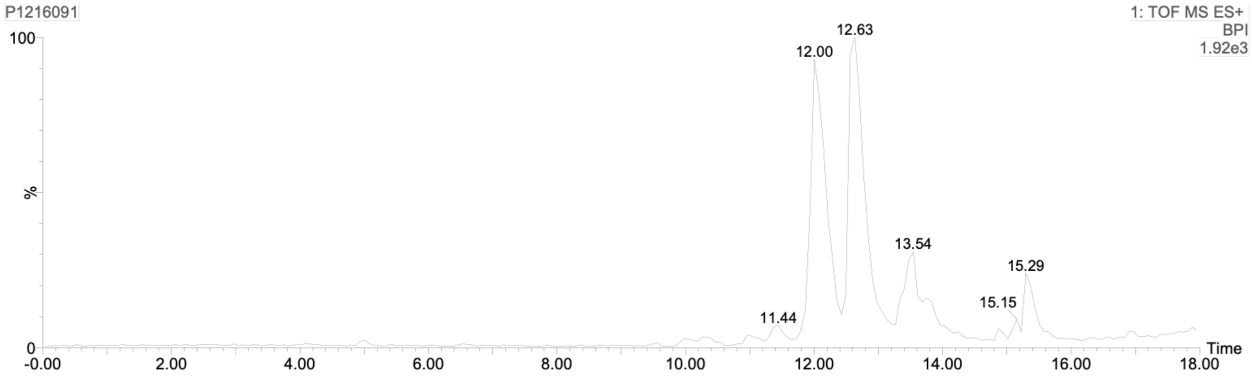

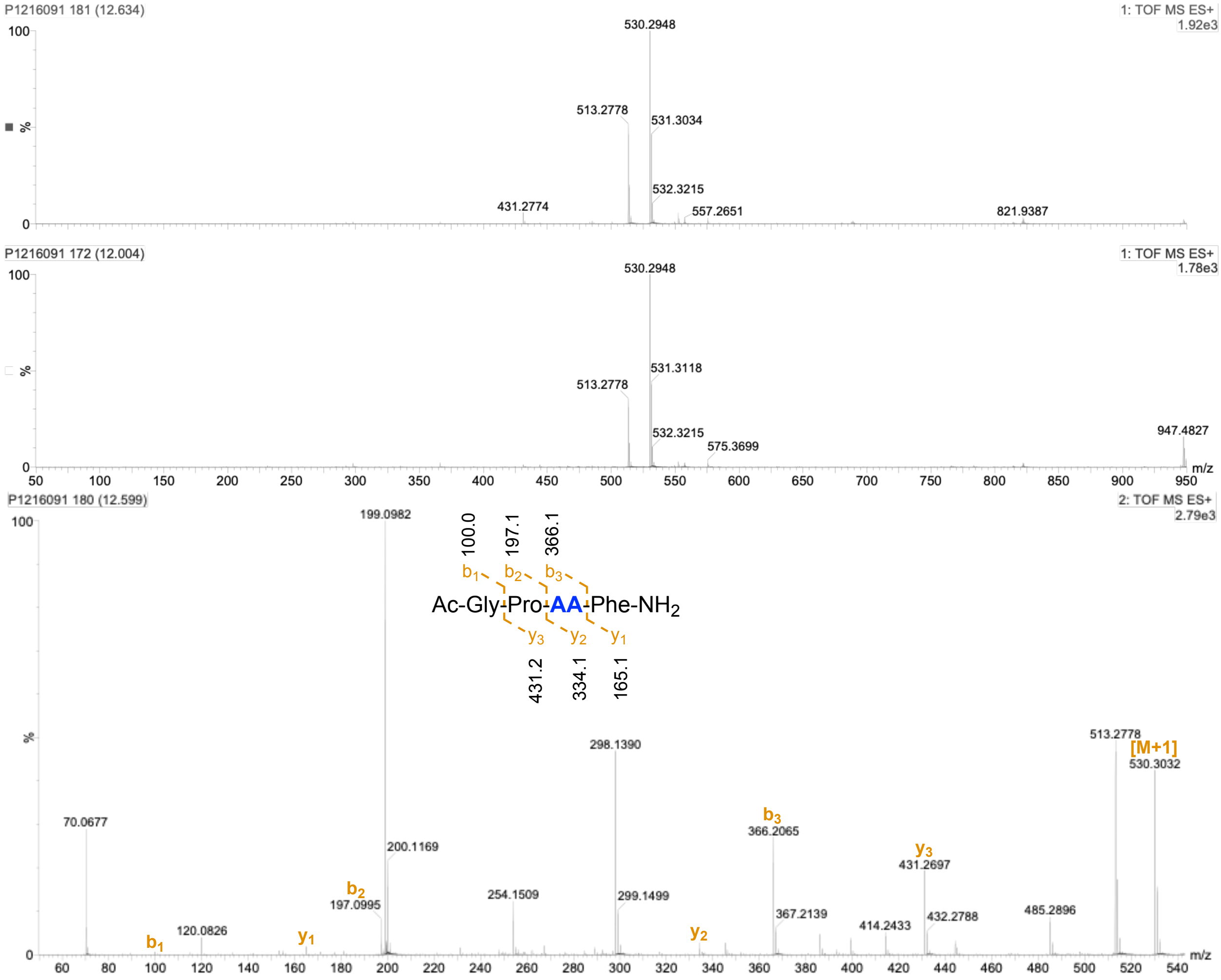

P1216091 181 (12.634)
1: TOF MS ES+
1.92e3
530.2948
513.2778
531.3034
532.3215
431.2774
557.2651
821.9387
P1216091 172 (12.004)
1: TOF MS ES+
1.78e3
530.2948
531.3118
513.2778
532.3215
575.3699
947.4827
m/z
P1216091 180 (12.599)
2: TOF MS ES+
2.79e3
199.0982
100.0
197.1
366.1
Ac-Gly-Pro-AA-Phe-NH2
431.2
334.1
165.1
513.2778
[M+1]
530.3032
298.1390
70.0677
b3
366.2065
200.1169
y3
431.2697
b2
197.0995
254.1509
299.1499
367.2139
414.2433
432.2788
485.2896
120.0826
b1
y1
y2
m/z

2H’: MW = 551.6, Purity = 49.7%, Yield = 8.7% [0.11 mg]

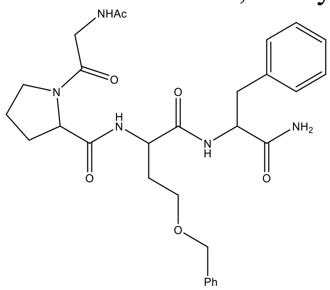

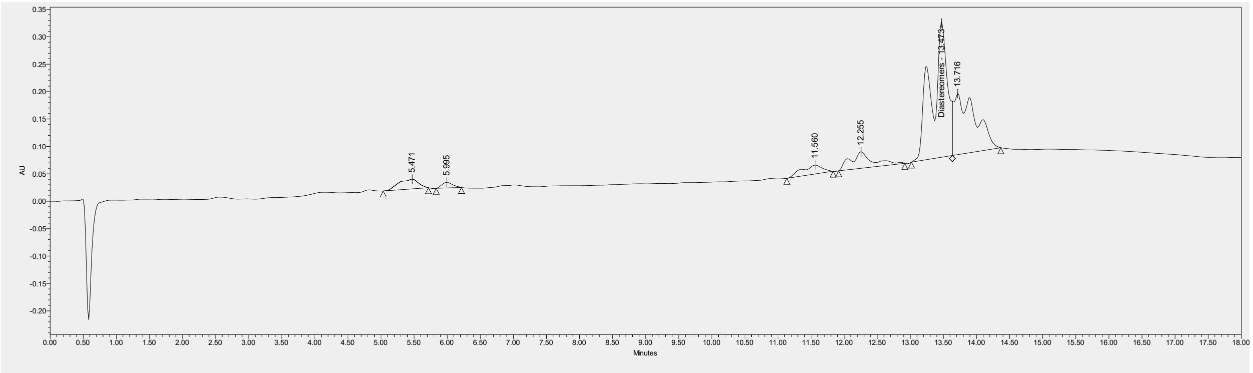

	Name	Retention Time	Area	% Area
1		5.471	375224	4.60
2		5.995	117249	1.44
3		11.560	361290	4.43
4		12.255	692913	8.49
5	Diastereomers	13.473	4056649	49.71
6		13.716	2557082	31.34

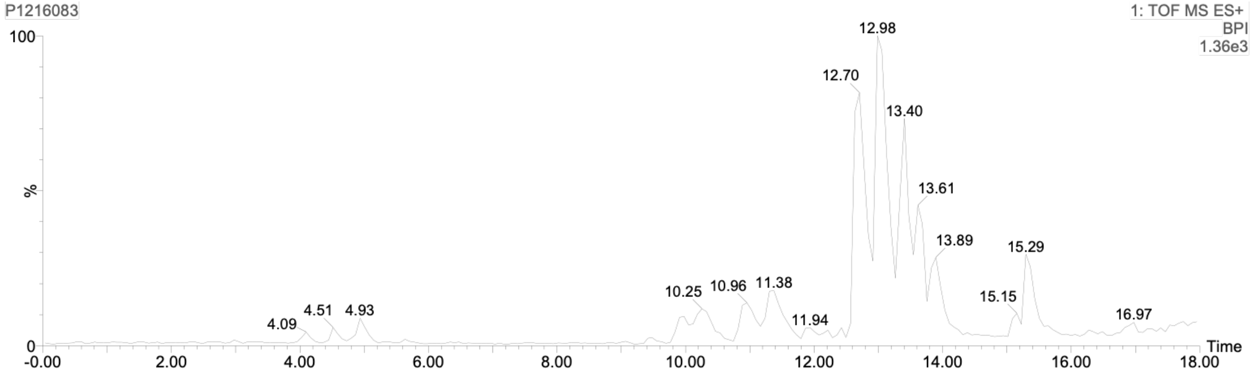

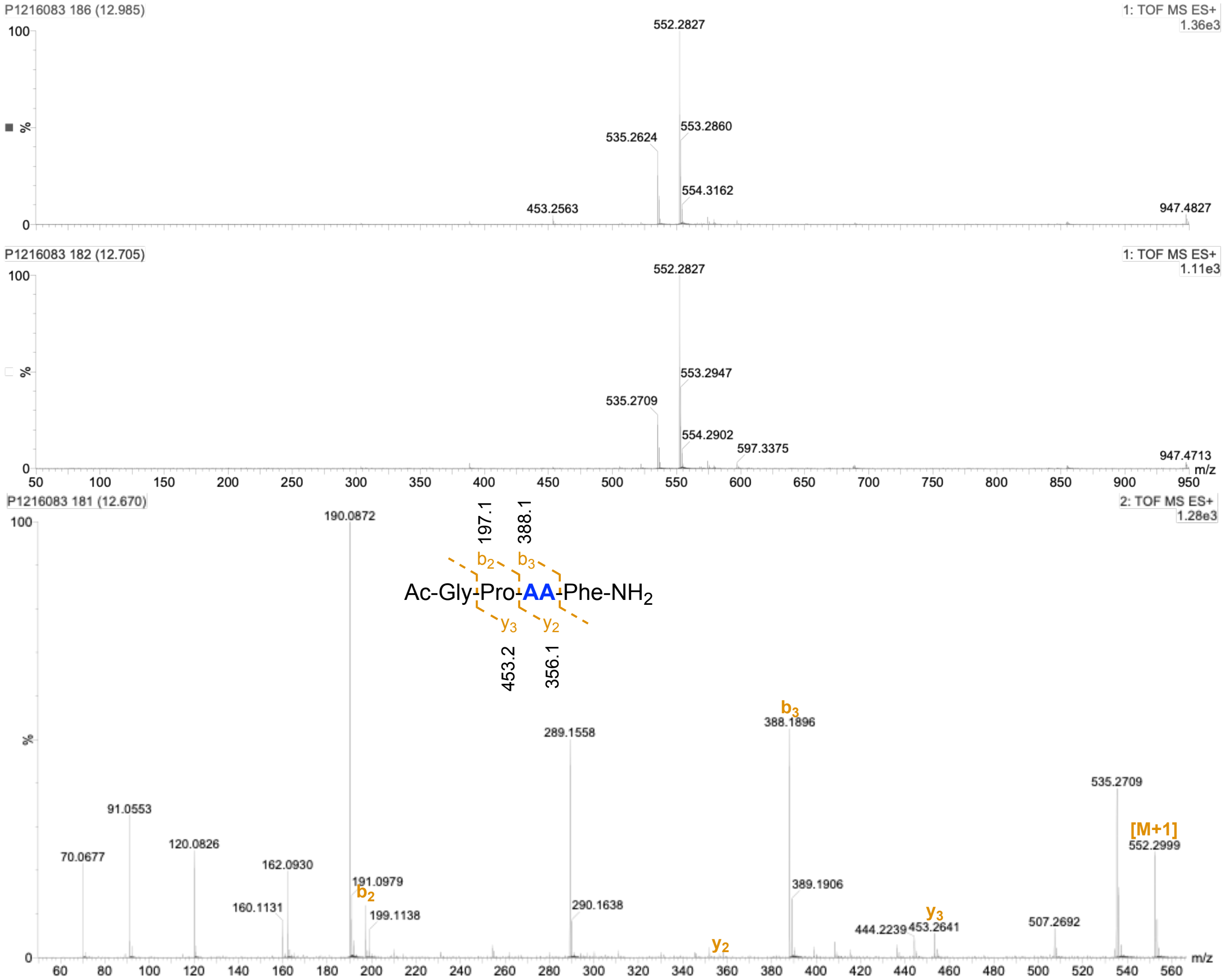
P1216083 186 (12.985)
1: TOF MS ES+
1.36e3
552.2827
553.2860
535.2624
554.3162
453.2563
947.4827
P1216083 182 (12.705)
1: TOF MS ES+
1.11e3
552.2827
553.2947
535.2709
554.2902
597.3375
947.4713
m/z
P1216083 181 (12.670)
2: TOF MS ES+
1.28e3
190.0872
197.1
388.1
b2
b3
Ac-Gly-Pro-AA-Phe-NH2
y3
y2
453.2
356.1
b3
388.1896
289.1558
535.2709
91.0553
[M+1]
552.2999
120.0826
70.0677
162.0930
191.0979
b2
389.1906
160.1131
199.1138
290.1638
y3
444.2239
453.2641
507.2692
y2
m/z
%

3H’: MW = 517.6, Purity = 38.1%, Yield = 10.3% [0.12 mg]

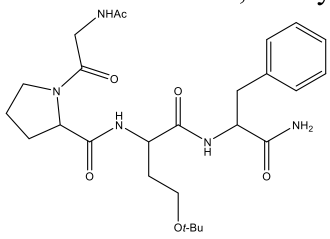

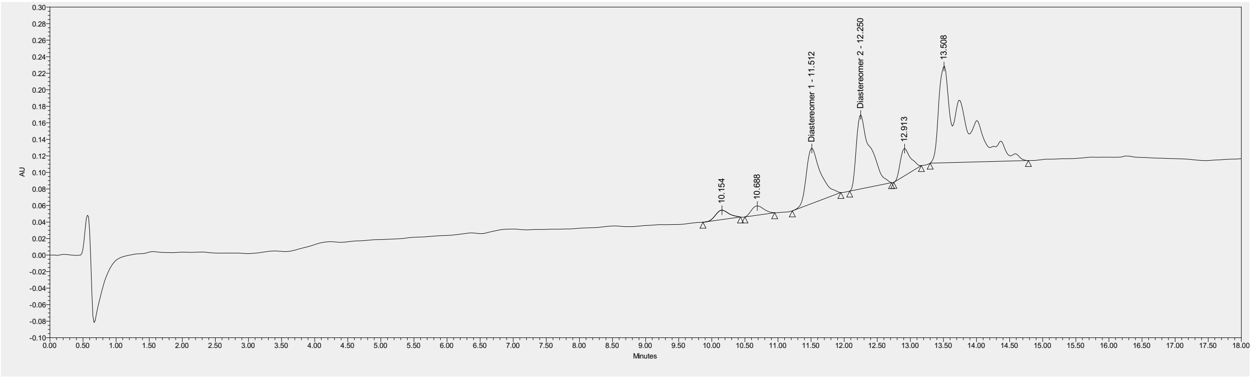

	Name	Retention Time	Area	% Area
1		10.154	160113	2.60
2		10.688	147322	2.39
3	Diastereomer 1	11.512	1010961	16.43
4	Diastereomer 2	12.250	1330428	21.63
5		12.913	360610	5.86
6		13.508	3142800	51.08

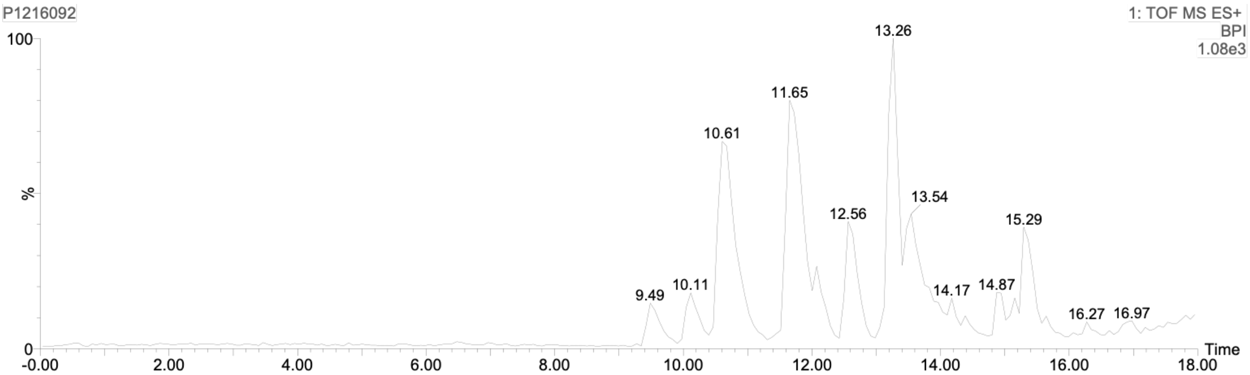

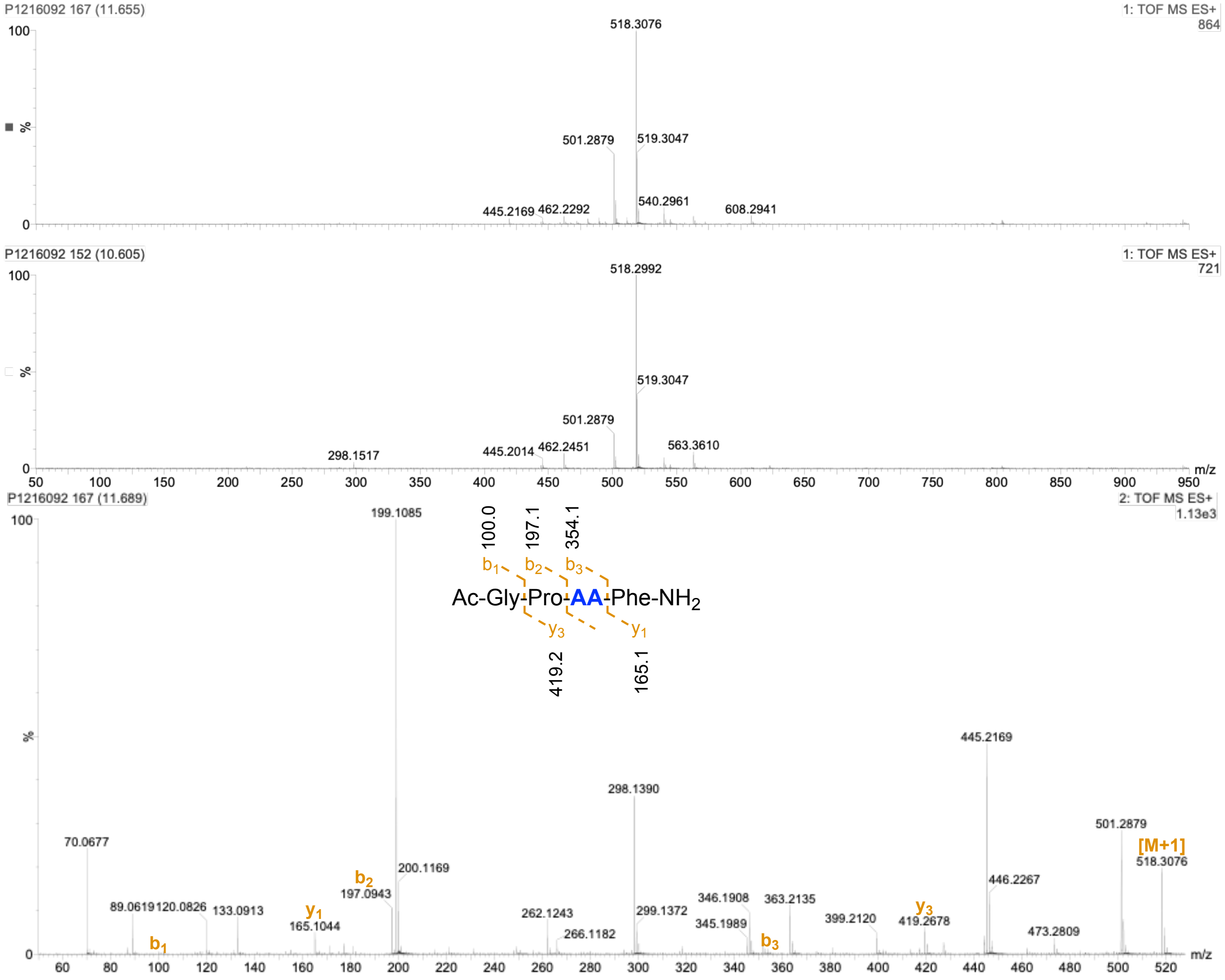
P1216092 167 (11.655)
1: TOF MS ES+
864
518.3076
501.2879
519.3047
445.2169
462.2292
540.2961
608.2941
P1216092 152 (10.605)
1: TOF MS ES+
721
518.2992
519.3047
501.2879
298.1517
445.2014
462.2451
563.3610
m/z
P1216092 167 (11.689)
2: TOF MS ES+
1.13e3
199.1085
100.0
197.1
354.1
Ac-Gly-Pro-AA-Phe-NH2
419.2
165.1
445.2169
298.1390
70.0677
501.2879
[M+1]
518.3076
200.1169
446.2267
89.0619
120.0826
133.0913
165.1044
197.0943
262.1243
266.1182
299.1372
346.1908
345.1989
363.2135
399.2120
419.2678
473.2809
m/z

4H': MW = 600.7, Purity = 69.7%, Yield = 12.7% [0.18 mg]

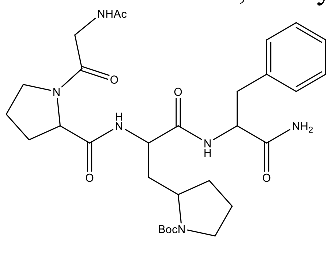

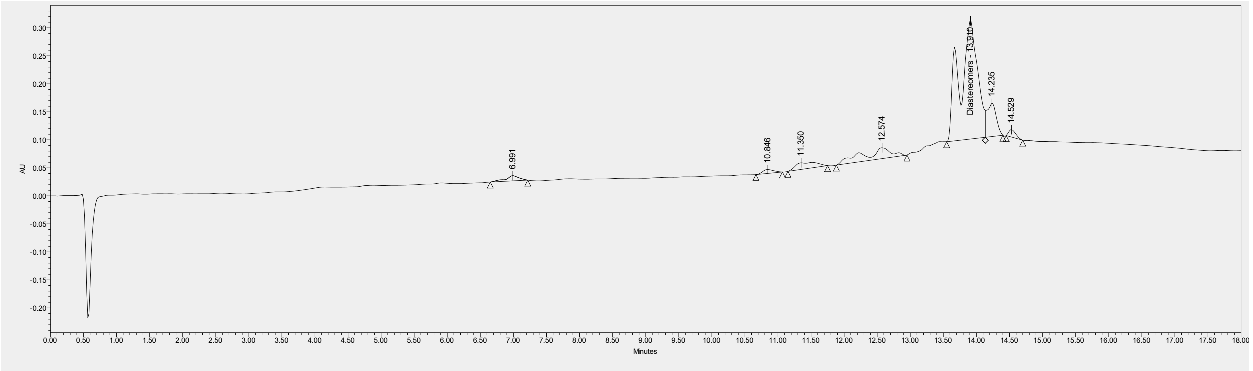

	Name	Retention Time	Area	% Area
1		6.991	127744	2.24
2		10.846	80606	1.41
3		11.350	248652	4.36
4		12.574	585778	10.27
5	Diastereomers	13.910	3972502	69.65
6		14.235	590580	10.36
7		14.529	97281	1.71

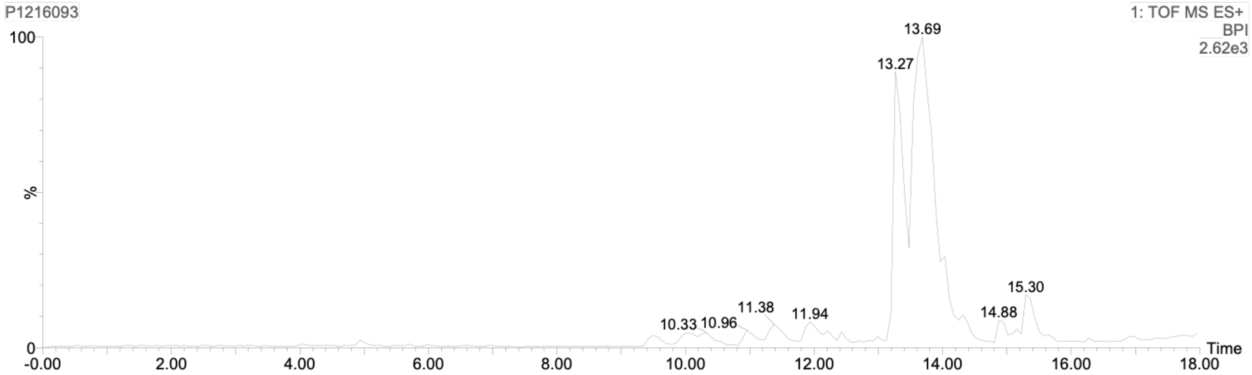

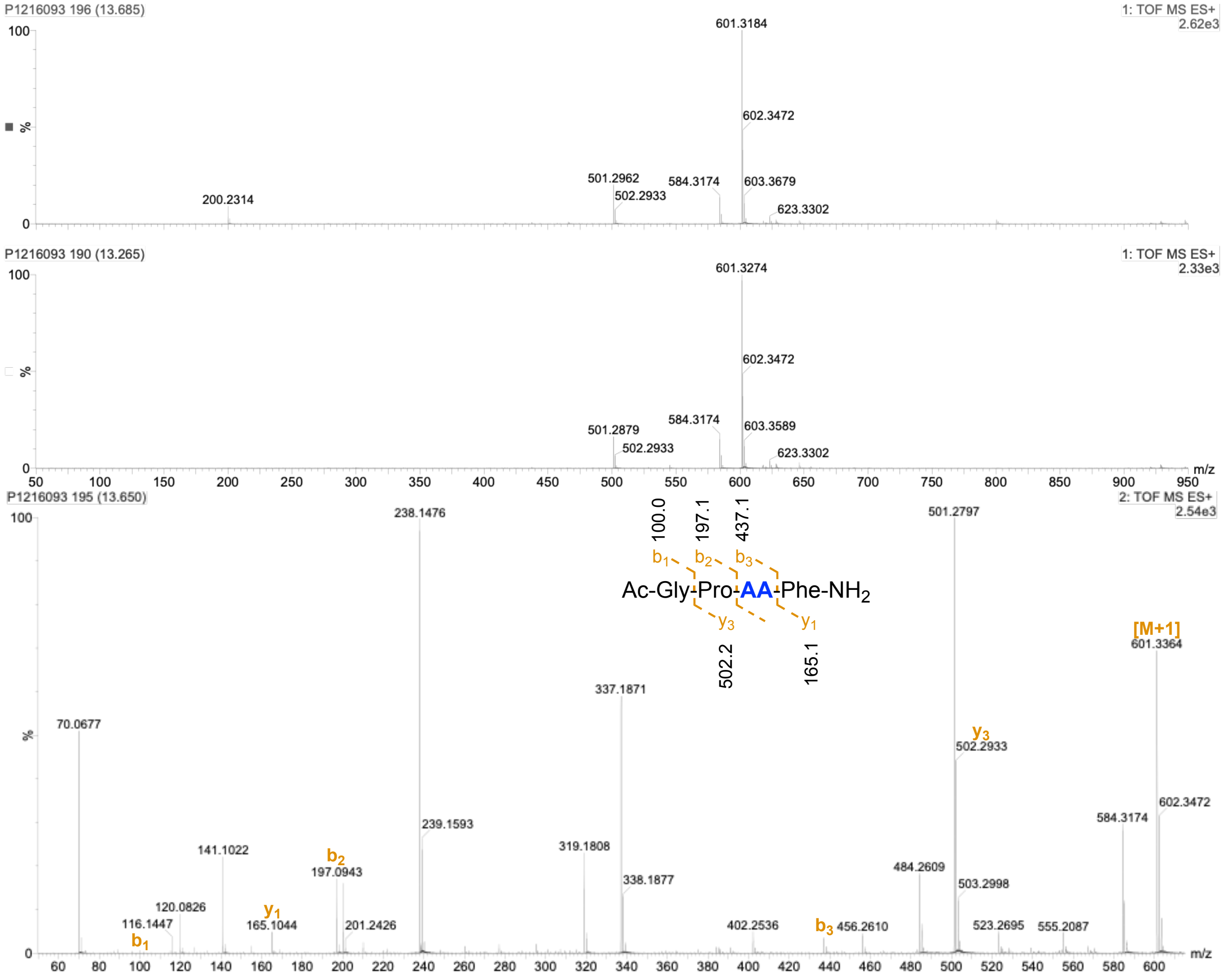
P1216093 196 (13.685)
1: TOF MS ES+
2.62e3
601.3184
602.3472
501.2962
502.2933
584.3174
603.3679
200.2314
623.3302
P1216093 190 (13.265)
1: TOF MS ES+
2.33e3
601.3274
602.3472
501.2879
502.2933
584.3174
603.3589
623.3302
m/z
P1216093 195 (13.650)
2: TOF MS ES+
2.54e3
238.1476
501.2797
Ac-Gly-Pro-AA-Phe-NH2
[M+1]
601.3364
337.1871
70.0677
502.2933
602.3472
584.3174
239.1593
141.1022
319.1808
197.0943
484.2609
503.2998
338.1877
120.0826
116.1447
165.1044
201.2426
402.2536
456.2610
523.2695
555.2087

5H’: MW = 543.7, Purity = 12.4%, Yield = 2.3% [0.029 mg]

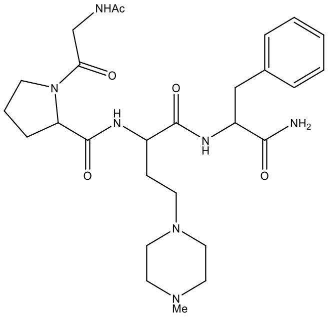

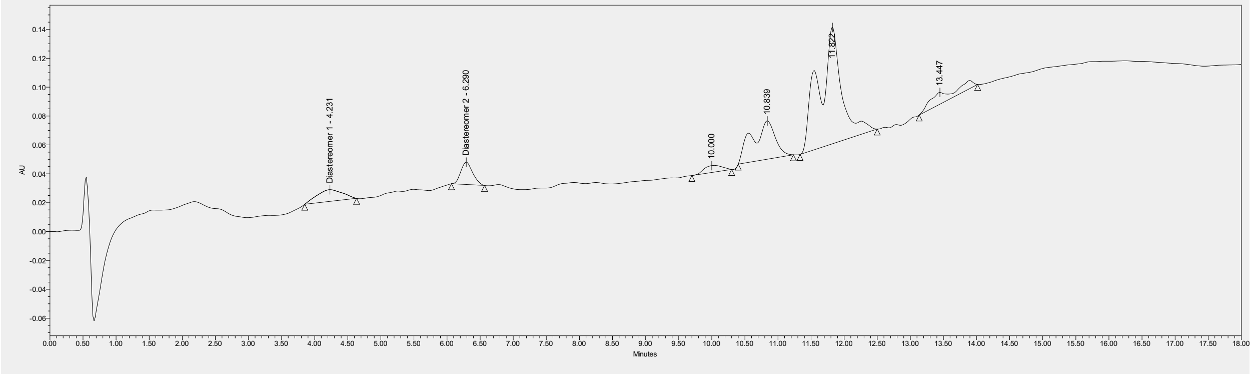

	Name	Retention Time	Area	% Area
1	Diastereomer 1	4.231	218721	6.61
2	Diastereomer 2	6.290	192620	5.82
3		10.000	90059	2.72
4		10.839	644331	19.48
5		11.822	1923578	58.14
6		13.447	239013	7.22

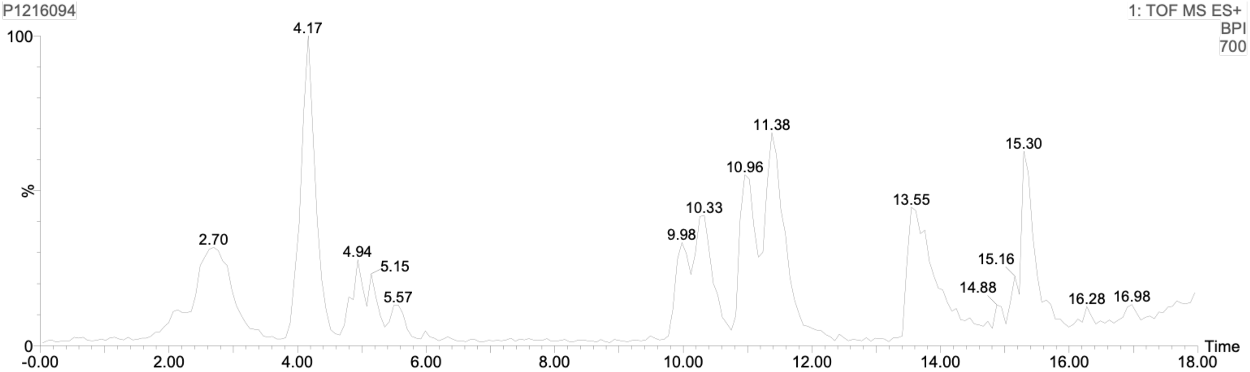

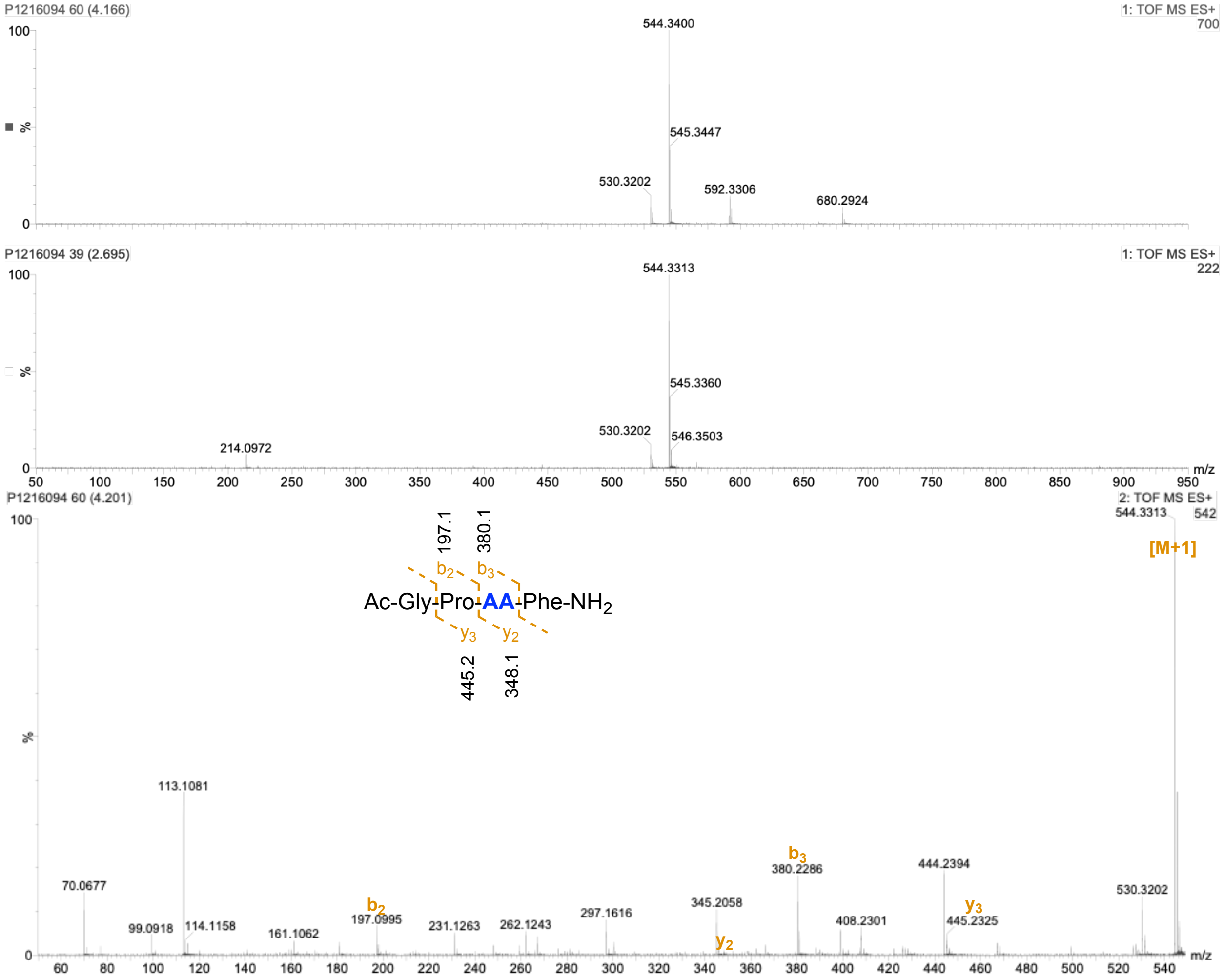
P1216094 60 (4.166)
1: TOF MS ES+
700
544.3400
545.3447
530.3202
592.3306
680.2924
P1216094 39 (2.695)
1: TOF MS ES+
222
544.3313
545.3360
530.3202
546.3503
214.0972
m/z
P1216094 60 (4.201)
2: TOF MS ES+
542
544.3313
[M+1]
Ac-Gly-Pro-AA-Phe-NH2
b2
b3
y3
y2
197.1
380.1
445.2
348.1
113.1081
70.0677
99.0918
114.1158
161.1062
197.0995
231.1263
262.1243
297.1616
345.2058
380.2286
408.2301
444.2394
445.2325
530.3202
m/z

6H’: MW = 491.6, Purity = 51.1%, Yield = 16.8% [0.19 mg]

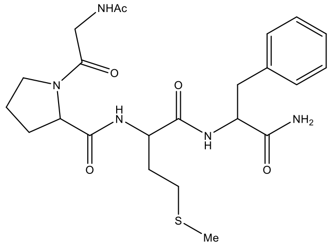

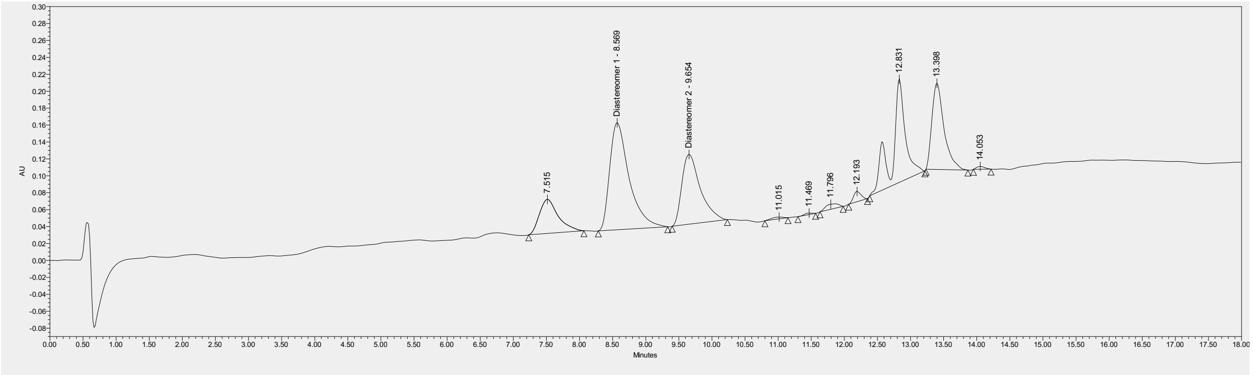

	Name	Retention Time	Area	% Area
1		7.515	796380	9.71
2	Diastereomer 1	8.569	2587021	31.56
3	Diastereomer 2	9.654	1604621	19.57
4		11.015	27217	0.33
5		11.469	14936	0.18
6		11.796	79494	0.97
7		12.193	95854	1.17
8		12.831	1663683	20.29
9		13.398	1300215	15.86
10		14.053	28795	0.35

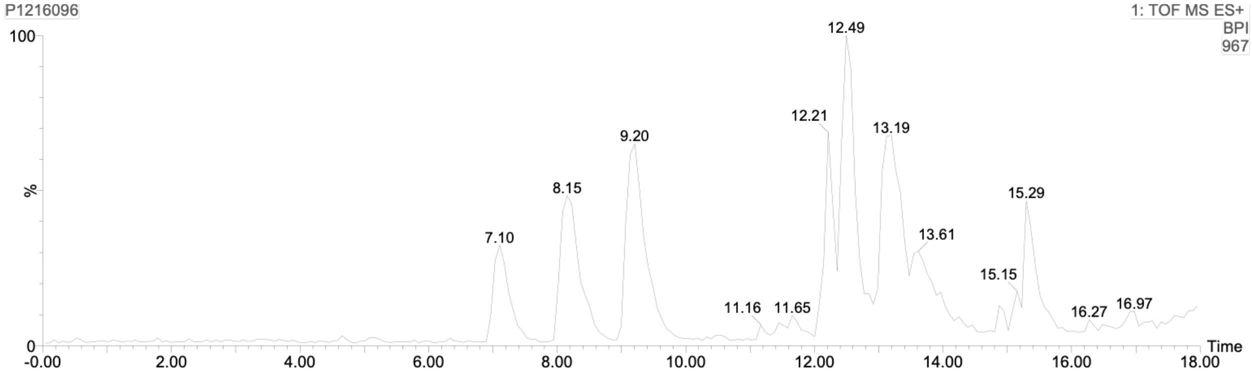

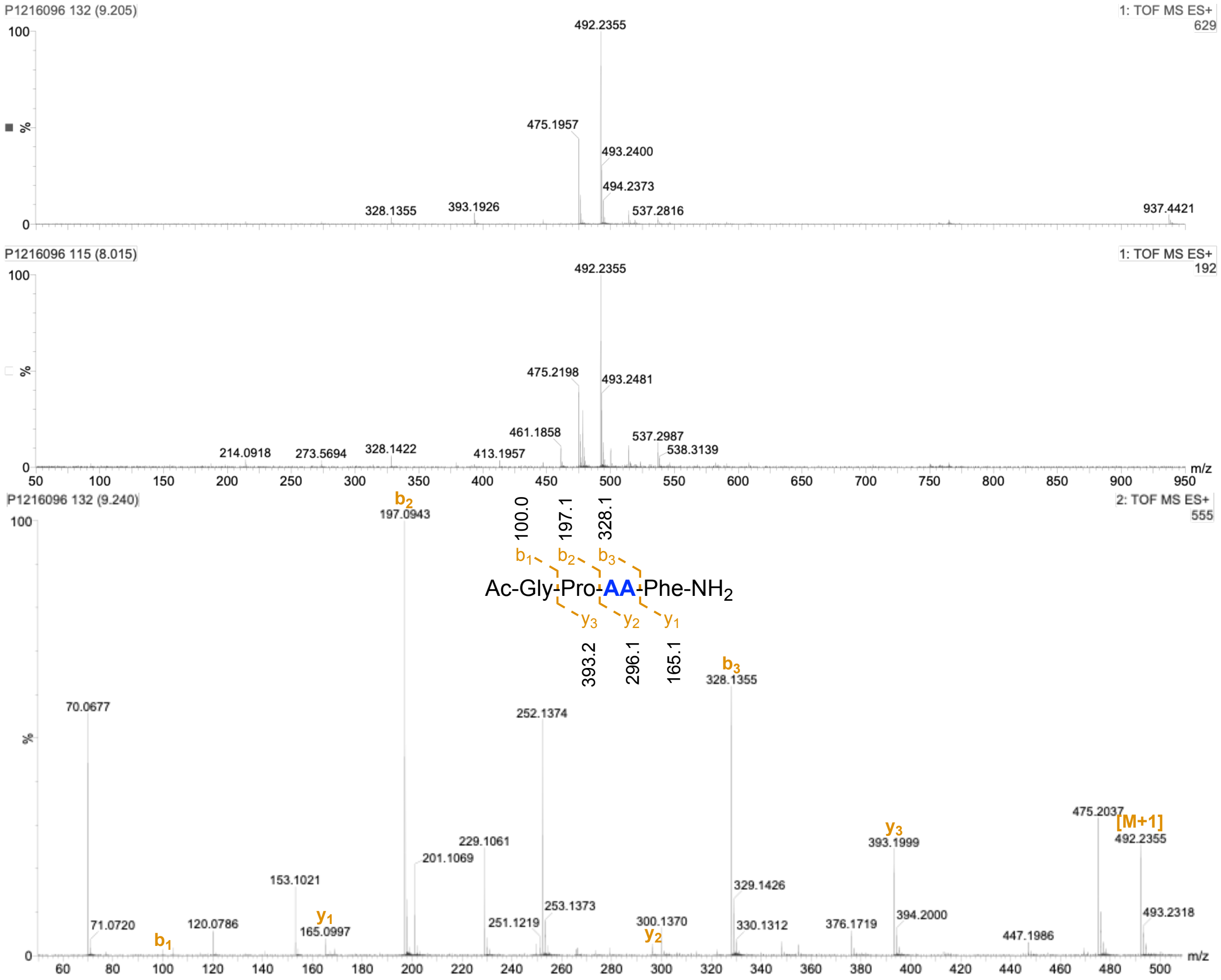
P1216096 132 (9.205)
1: TOF MS ES+
629
492.2355
475.1957
493.2400
494.2373
328.1355
393.1926
537.2816
937.4421
P1216096 115 (8.015)
1: TOF MS ES+
192
492.2355
475.2198
493.2481
461.1858
537.2987
538.3139
214.0918
273.5694
328.1422
413.1957
m/z
P1216096 132 (9.240)
2: TOF MS ES+
555
b2
197.0943
Ac-Gly-Pro-AA-Phe-NH2
100.0
197.1
328.1
393.2
296.1
165.1
b3
328.1355
70.0677
252.1374
475.2037
[M+1]
492.2355
y3
393.1999
229.1061
201.1069
153.1021
329.1426
253.1373
y1
165.0997
b1
71.0720
120.0786
251.1219
300.1370
y2
330.1312
376.1719
394.2000
447.1986
493.2318
%
m/z

7H’: MW = 600.7, Purity = 35.2%, Yield = 3.0% [0.042 mg]

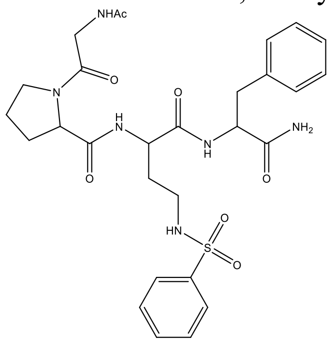

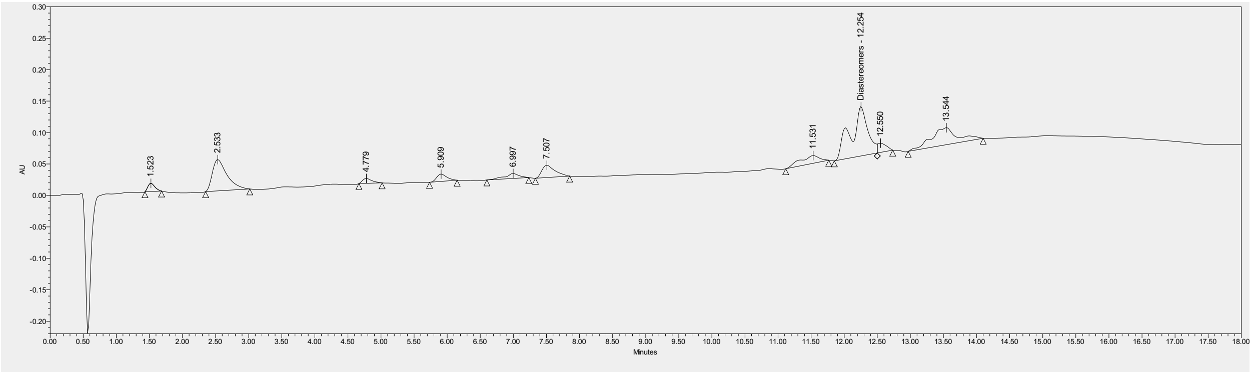

	Name	Retention Time	Area	% Area
1		1.523	95469	2.40
2		2.533	763594	19.18
3		4.779	76113	1.91
4		5.909	124079	3.12
5		6.997	116795	2.93
6		7.507	268668	6.75
7		11.531	262732	6.60
8	Diastereomers	12.254	1400654	35.19
9		12.550	130642	3.28
10		13.544	742011	18.64

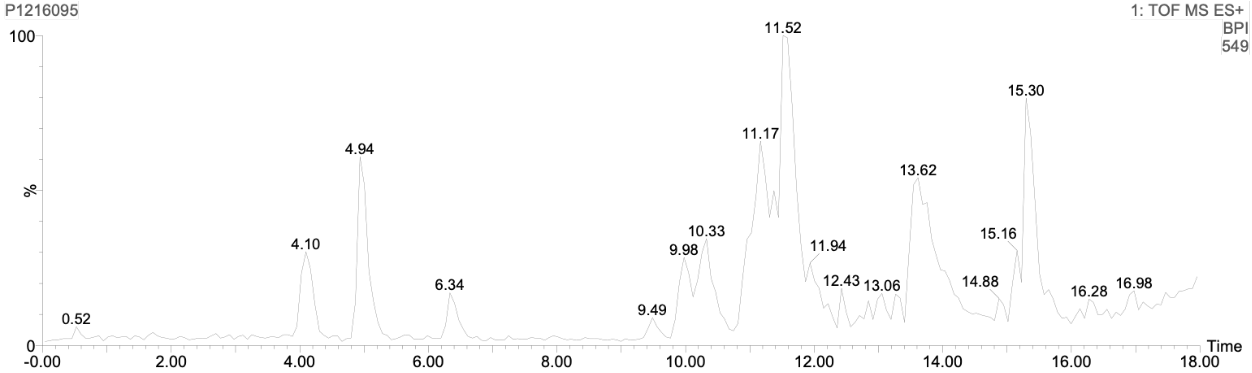

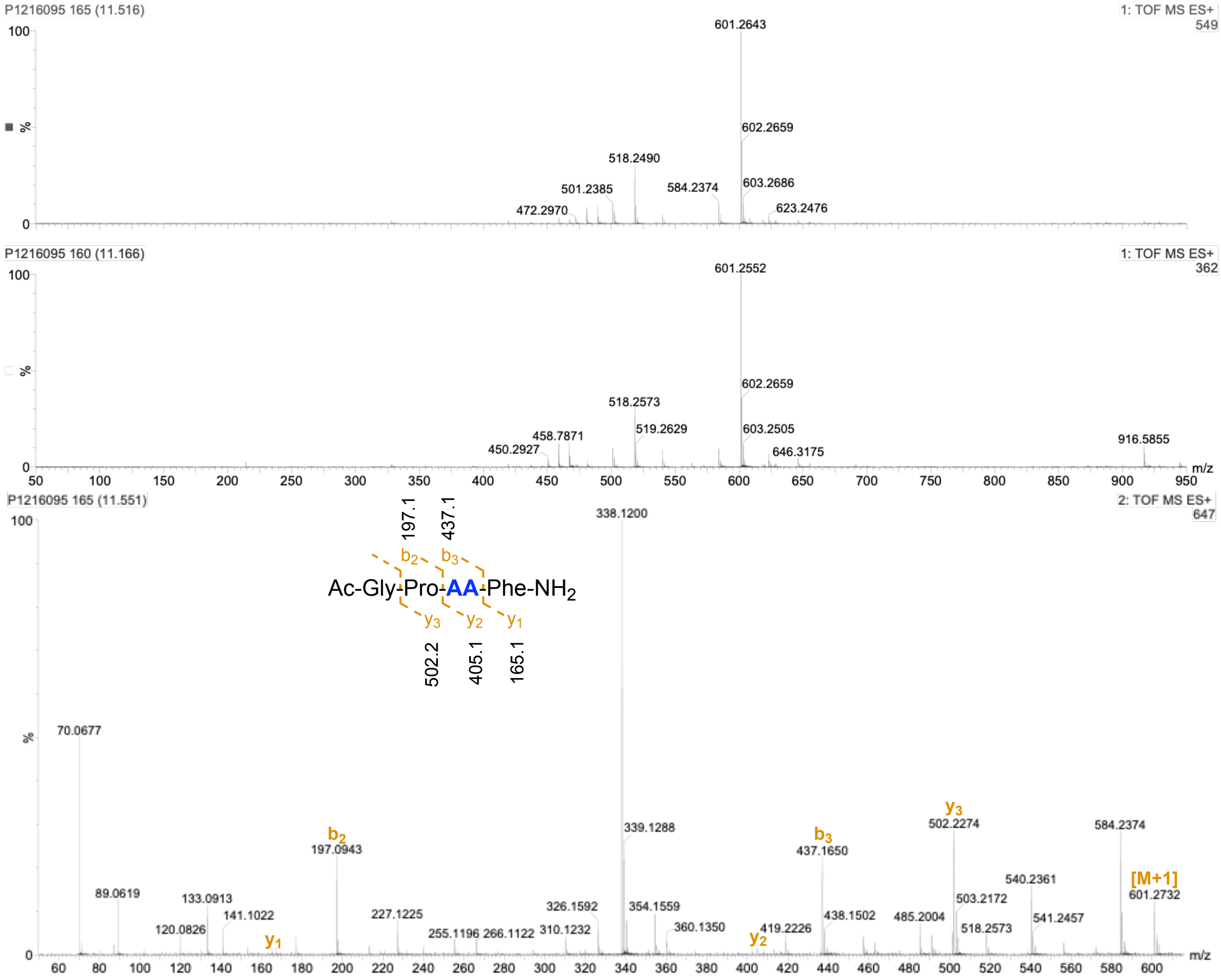
P1216095 165 (11.516)
1: TOF MS ES+
549
601.2643
602.2659
518.2490
584.2374
501.2385
603.2686
472.2970
623.2476
P1216095 160 (11.166)
1: TOF MS ES+
362
601.2552
602.2659
518.2573
519.2629
603.2505
458.7871
450.2927
646.3175
916.5855
m/z
P1216095 165 (11.551)
2: TOF MS ES+
647
338.1200
Ac-Gly-Pro-AA-Phe-NH2
b2 197.1
b3 437.1
y3 502.2
y2 405.1
y1 165.1
70.0677
89.0619
133.0913
141.1022
120.0826
b2
197.0943
227.1225
255.1196
266.1122
310.1232
326.1592
339.1288
354.1559
360.1350
y1
y2
419.2226
b3
437.1650
438.1502
485.2004
y3
502.2274
503.2172
518.2573
540.2361
541.2457
584.2374
[M+1]
601.2732
m/z

8H': MW = 485.6, Purity = 50.9%, Yield = 14.2% [0.16 mg]

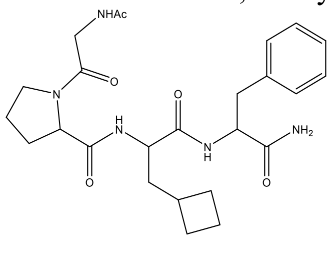

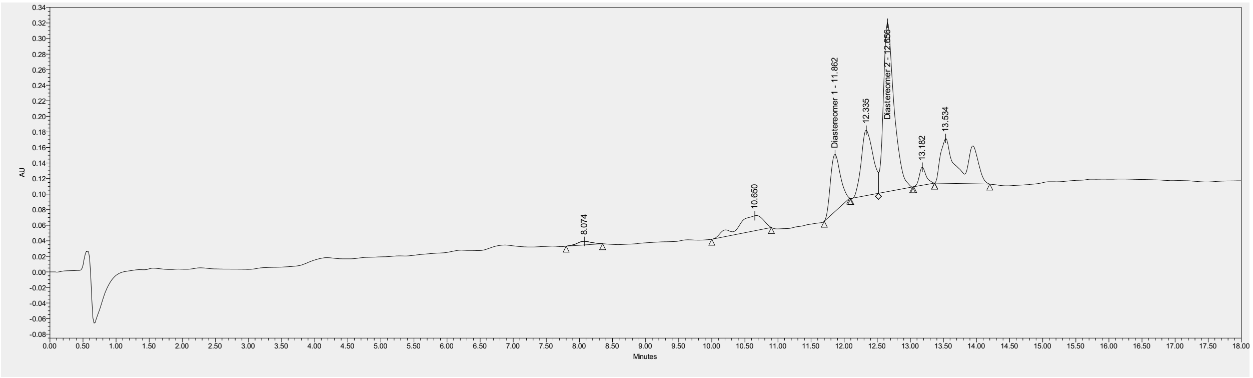

	Name	Retention Time	Area	% Area
1		8.074	72196	1.14
2		10.650	542601	8.53
3	Diastereomer 1	11.862	771342	12.13
4		12.335	1045789	16.45
5	Diastereomer 2	12.656	2462397	38.73
6		13.182	166563	2.62
7		13.534	1297561	20.41

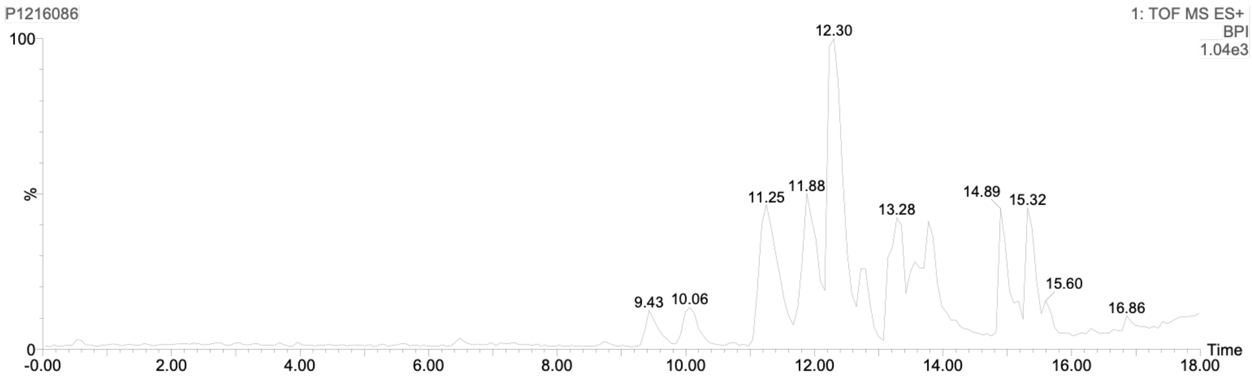

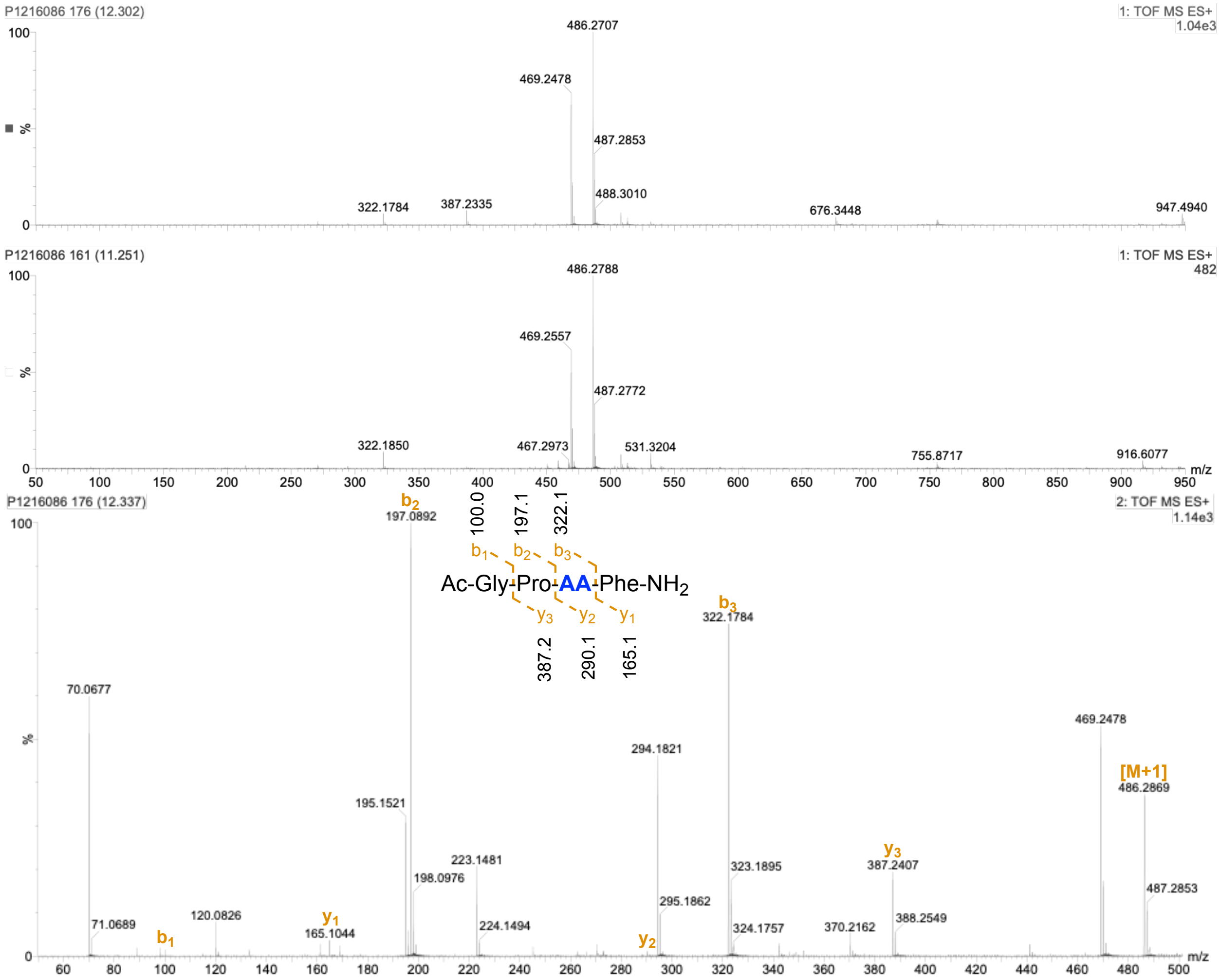
P1216086 176 (12.302)
1: TOF MS ES+
1.04e3
486.2707
469.2478
487.2853
488.3010
322.1784
387.2335
676.3448
947.4940
P1216086 161 (11.251)
1: TOF MS ES+
482
486.2788
469.2557
487.2772
322.1850
467.2973
531.3204
755.8717
916.6077
m/z
P1216086 176 (12.337)
2: TOF MS ES+
1.14e3
Ac-Gly-Pro-AA-Phe-NH2
b1 b2 b3
y3 y2 y1
100.0
197.1
322.1
387.2
290.1
165.1
b2
197.0892
b3
322.1784
70.0677
71.0689
b1
120.0826
y1
165.1044
195.1521
198.0976
223.1481
224.1494
294.1821
295.1862
y2
323.1895
324.1757
370.2162
y3
387.2407
388.2549
469.2478
[M+1]
486.2869
487.2853
m/z
%

9H': MW = 586.7, Purity = 28.5%, Yield = 8.2% [0.11 mg]

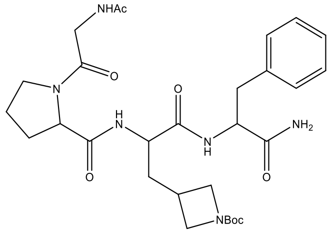

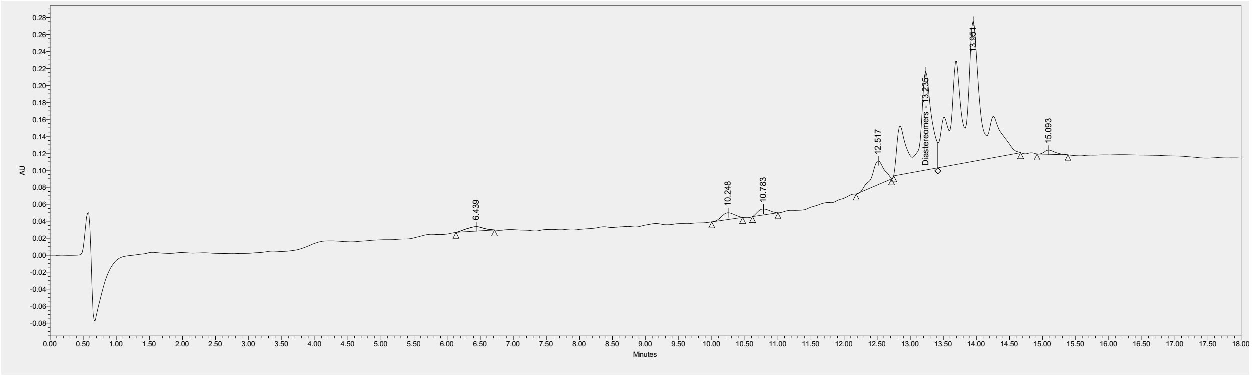

	Name	Retention Time	Area	% Area
1		6.439	95558	1.46
2		10.248	105831	1.62
3		10.783	87893	1.35
4		12.517	365325	5.59
5	Diastereomers	13.235	1863748	28.54
6		13.951	3955380	60.57
7		15.093	56305	0.86

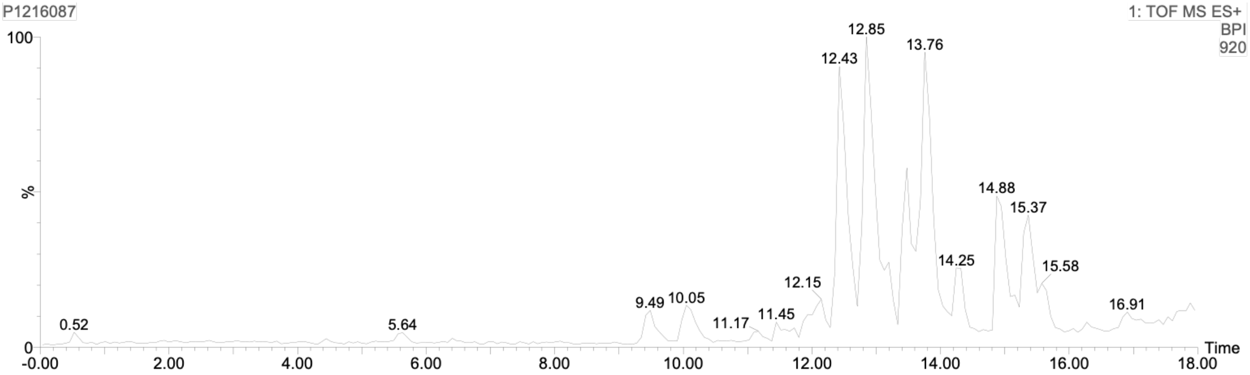

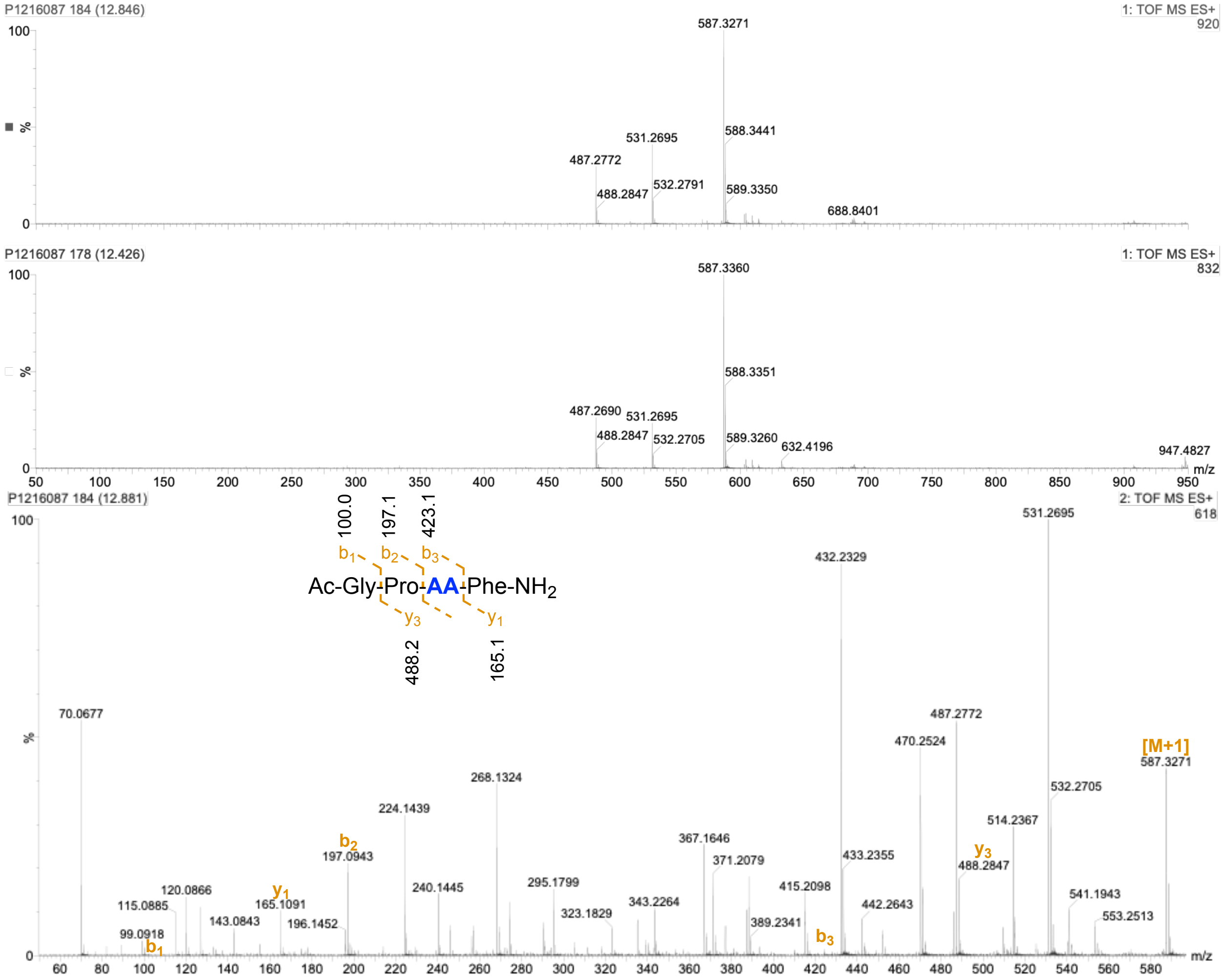

P1216087 184 (12.846)
1: TOF MS ES+
920
587.3271
531.2695
588.3441
487.2772
488.2847
532.2791
589.3350
688.8401
P1216087 178 (12.426)
1: TOF MS ES+
832
587.3360
588.3351
487.2690
531.2695
488.2847
532.2705
589.3260
632.4196
947.4827
m/z
P1216087 184 (12.881)
2: TOF MS ES+
618
Ac-Gly-Pro-AA-Phe-NH2
100.0
197.1
423.1
488.2
165.1
531.2695
432.2329
487.2772
470.2524
[M+1]
587.3271
532.2705
514.2367
488.2847
70.0677
268.1324
224.1439
197.0943
367.1646
371.2079
433.2355
415.2098
240.1445
295.1799
120.0866
115.0885
165.1091
343.2264
323.1829
442.2643
541.1943
553.2513
143.0843
196.1452
389.2341
99.0918

10H’: MW = 473.6, Purity = 68.9%, Yield = 28.7% [0.32 mg]

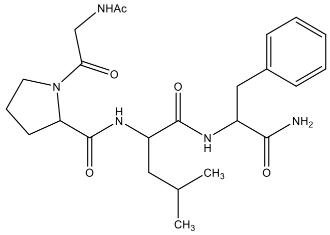

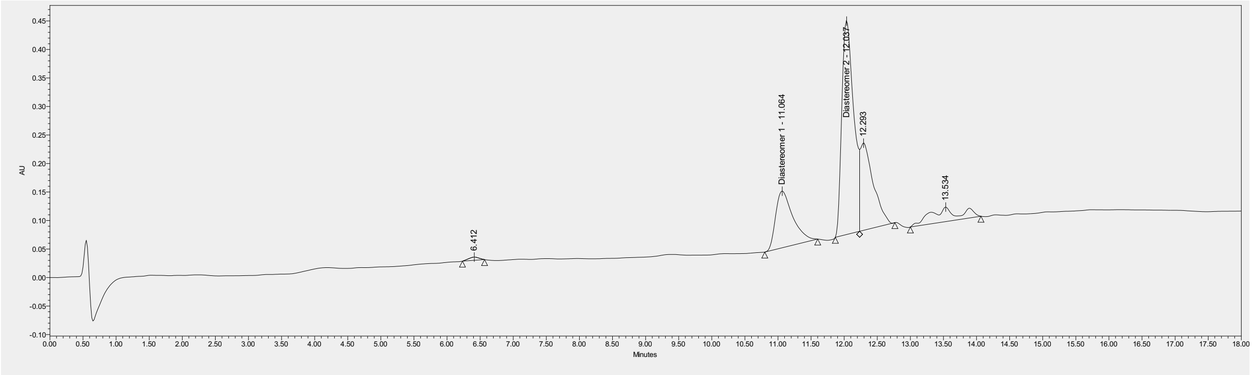

	Name	Retention Time	Area	% Area
1		6.412	62010	0.66
2	Diastereomer 1	11.064	1801359	19.06
3	Diastereomer 2	12.037	4712137	49.87
4		12.293	2113029	22.36
5		13.534	760558	8.05

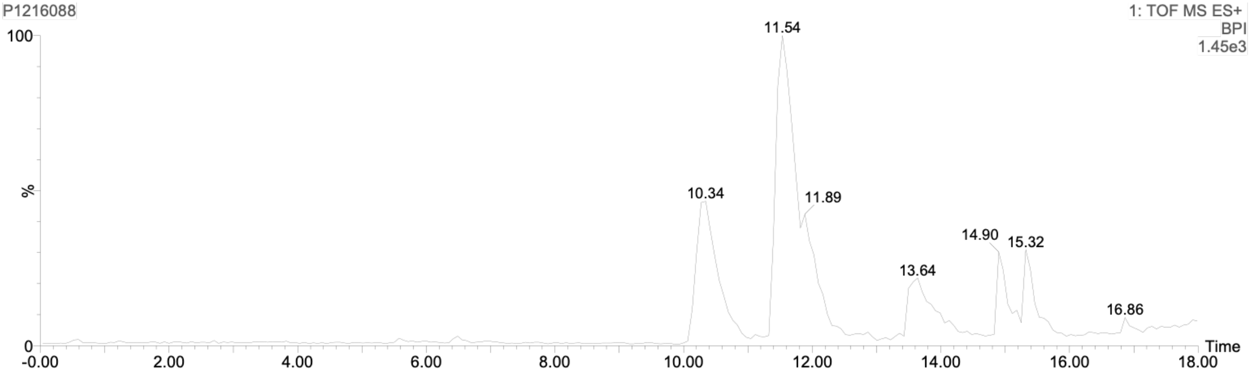

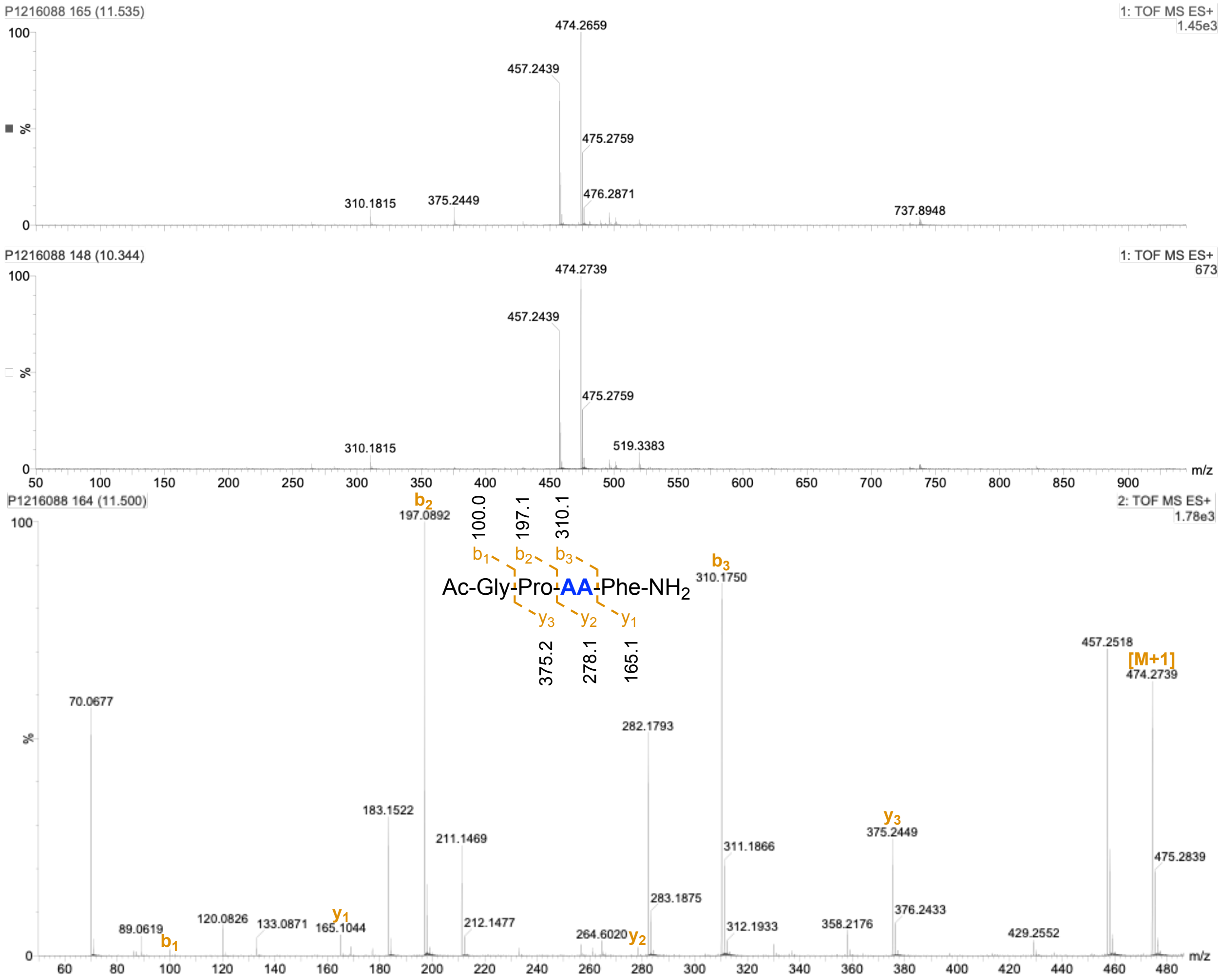

P1216088 165 (11.535)
1: TOF MS ES+
1.45e3
474.2659
457.2439
475.2759
476.2871
310.1815
375.2449
737.8948
P1216088 148 (10.344)
1: TOF MS ES+
673
474.2739
457.2439
475.2759
310.1815
519.3383
m/z
P1216088 164 (11.500)
2: TOF MS ES+
1.78e3
Ac-Gly-Pro-AA-Phe-NH2
b1 b2 b3
y3 y2 y1
100.0 197.1 310.1
375.2 278.1 165.1
b2 197.0892
b3 310.1750
457.2518
[M+1] 474.2739
70.0677
282.1793
183.1522
211.1469
y3 375.2449
311.1866
475.2839
283.1875
376.2433
120.0826
133.0871
y1 165.1044
212.1477
89.0619
b1
264.6020
y2
312.1933
358.2176
429.2552
m/z

11H’: MW = 513.6, Purity = 75.1%, Yield = 13.1% [0.16 mg]

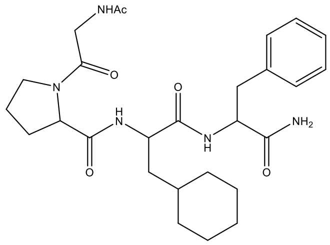

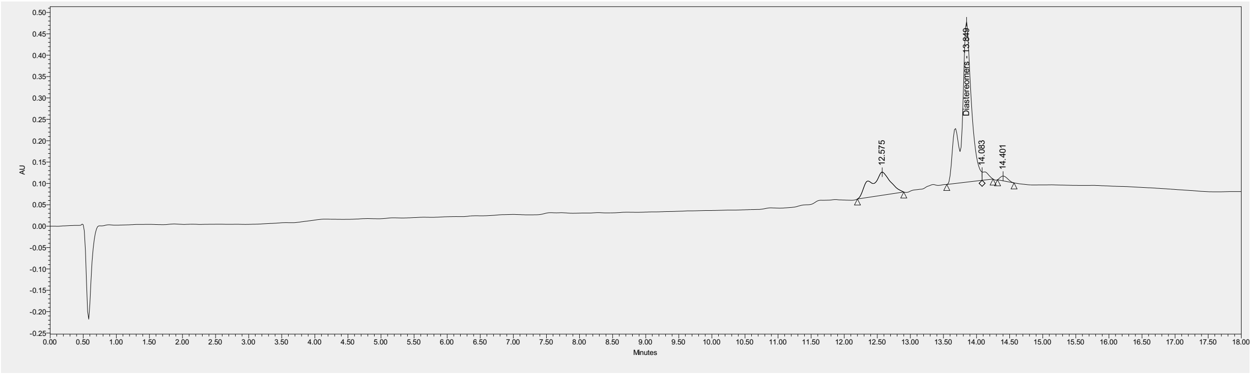

	Name	Retention Time	Area	% Area
1		12.575	1147553	21.10
2	Diastereomers	13.849	4083102	75.07
3		14.083	121884	2.24
4		14.401	86797	1.60

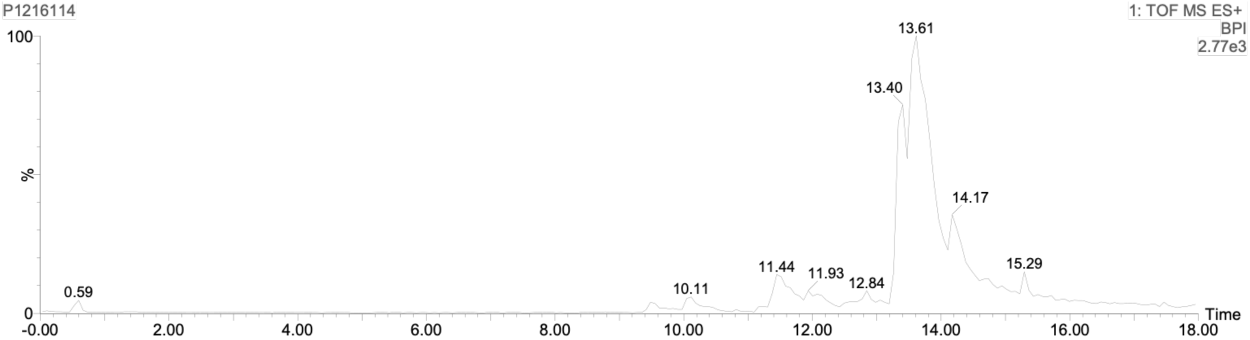

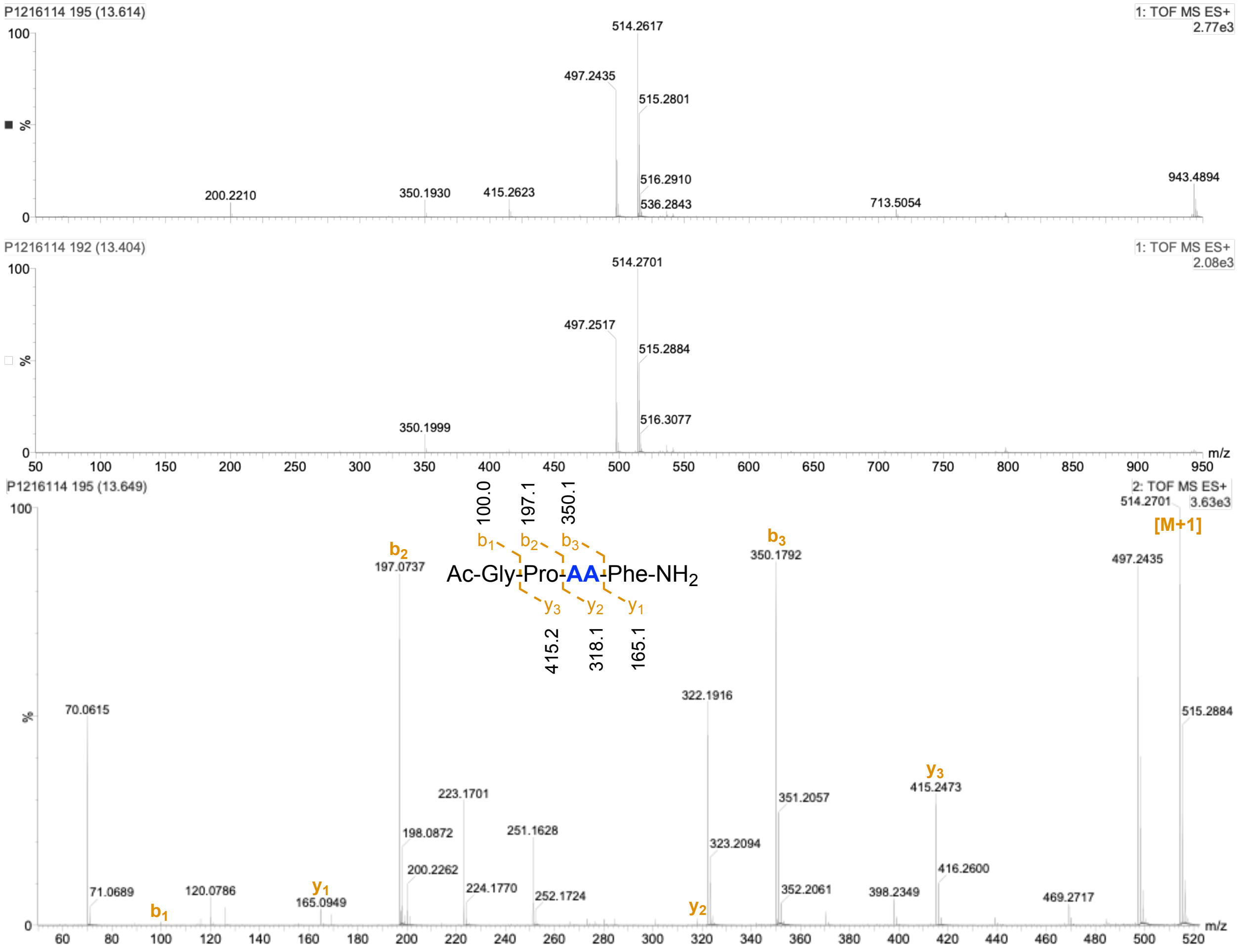
P1216114 195 (13.614)
1: TOF MS ES+
2.77e3
514.2617
497.2435
515.2801
200.2210
350.1930
415.2623
516.2910
536.2843
713.5054
943.4894
P1216114 192 (13.404)
1: TOF MS ES+
2.08e3
514.2701
497.2517
515.2884
350.1999
516.3077
m/z
P1216114 195 (13.649)
2: TOF MS ES+
514.2701
3.63e3
[M+1]
Ac-Gly-Pro-AA-Phe-NH2
100.0
197.1
350.1
415.2
318.1
165.1
b2
197.0737
b3
350.1792
497.2435
70.0615
322.1916
515.2884
223.1701
351.2057
y3
415.2473
198.0872
251.1628
323.2094
200.2262
416.2600
71.0689
120.0786
y1
165.0949
224.1770
252.1724
352.2061
398.2349
469.2717
b1
y2
m/z

12H': MW = 487.6, Purity = 53.6%, Yield = 7.9% [0.090 mg]

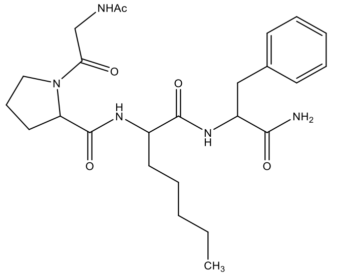

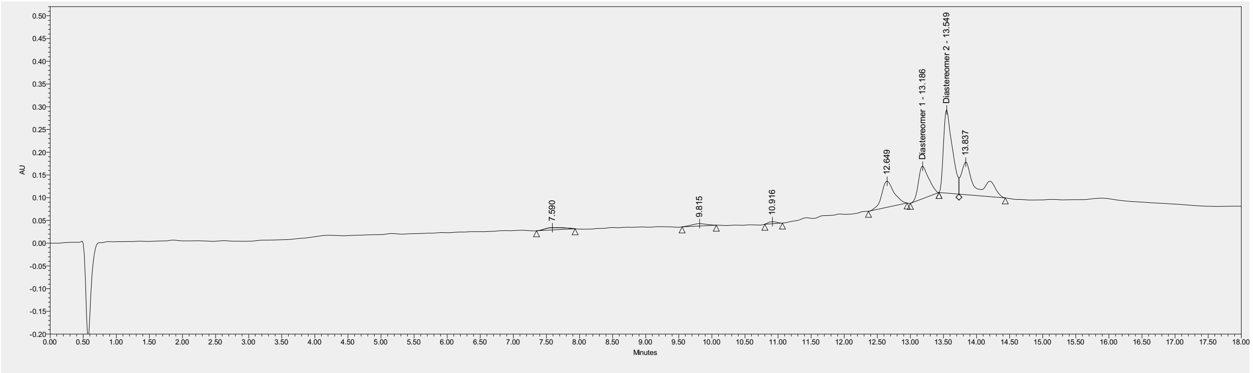

	Name	Retention Time	Area	% Area
1		7.590	102030	2.21
2		9.815	82593	1.79
3		10.916	40026	0.87
4		12.649	747634	16.22
5	Diastereomer 1	13.186	773407	16.78
6	Diastereomer 2	13.549	1697330	36.83
7		13.837	1165701	25.29

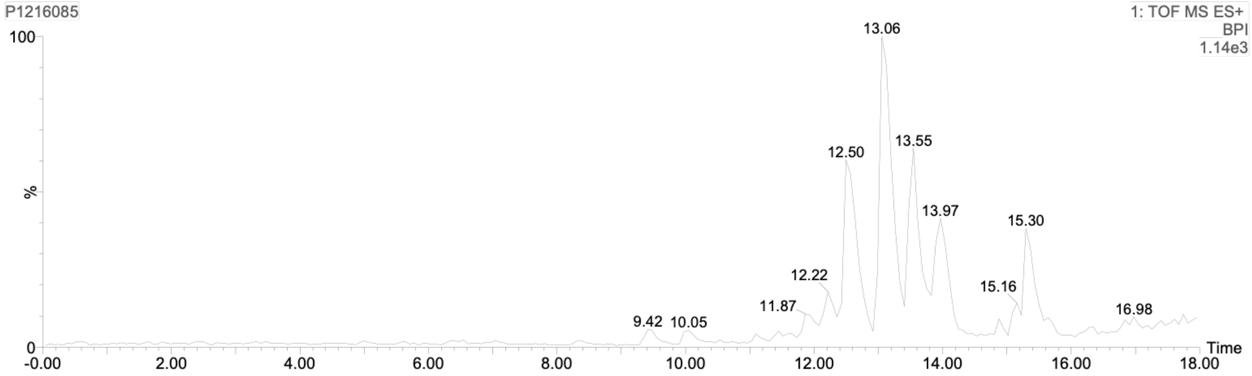

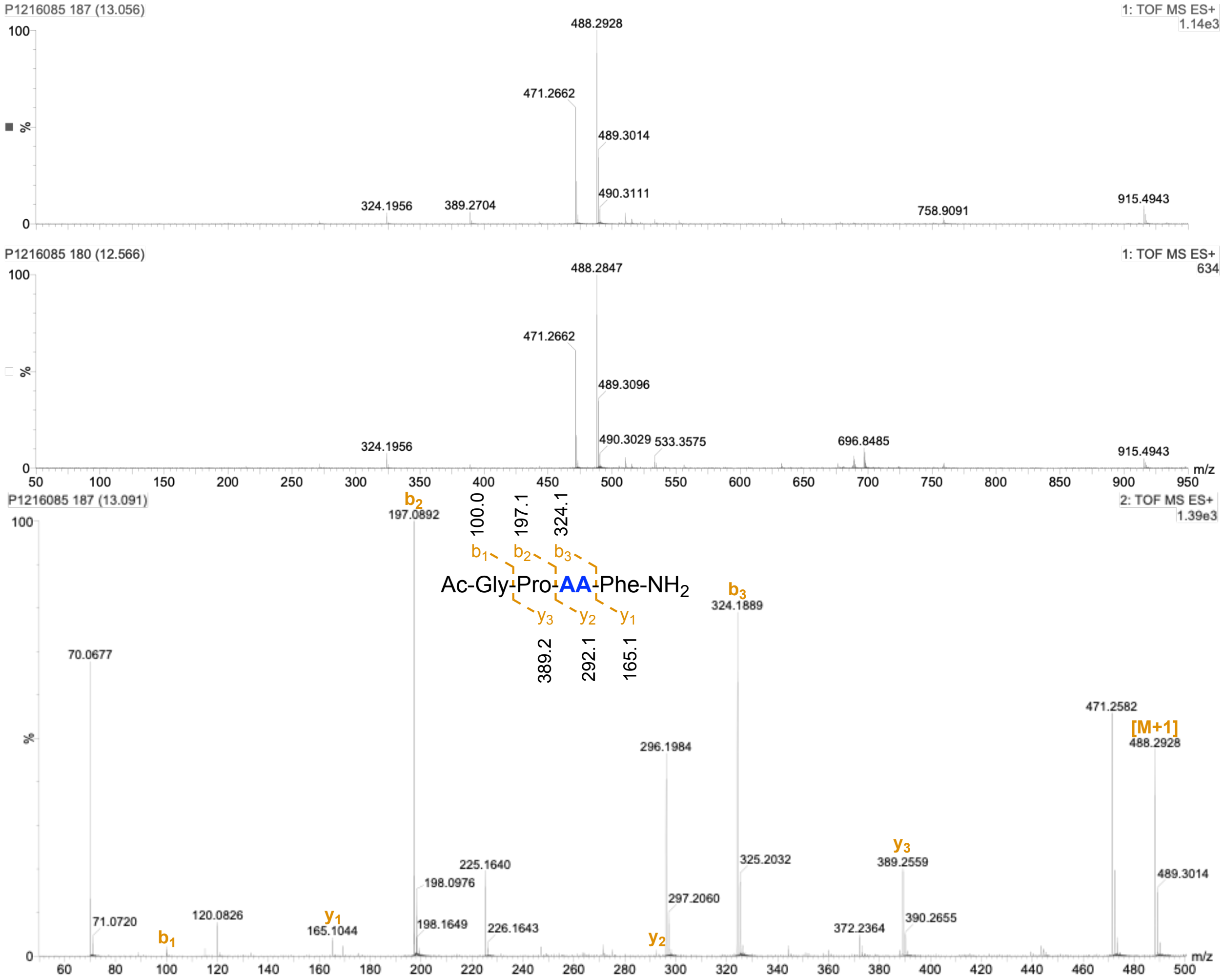

P1216085 187 (13.056)
1: TOF MS ES+
1.14e3
488.2928
471.2662
489.3014
490.3111
324.1956
389.2704
758.9091
915.4943
P1216085 180 (12.566)
1: TOF MS ES+
634
488.2847
471.2662
489.3096
490.3029
533.3575
696.8485
324.1956
915.4943
m/z
P1216085 187 (13.091)
2: TOF MS ES+
1.39e3
b2
197.0892
100.0
197.1
324.1
Ac-Gly-Pro-AA-Phe-NH2
389.2
292.1
165.1
b3
324.1889
70.0677
471.2582
[M+1]
488.2928
296.1984
225.1640
198.0976
325.2032
y3
389.2559
489.3014
297.2060
120.0826
71.0720
b1
y1
165.1044
198.1649
226.1643
y2
372.2364
390.2655
m/z

References

1 I. Jhulki, P. K. Chanani, S. H. Abdelwahed, T. P. Begley, *J. Am. Chem. Soc.* 2016, **138**, 8324–8327.
2 P. M. Morrison, P. J. Foley, S. L. Warriner, M. E. Webb, *Chem. Commun.* 2015, **51**, 13470–13473.
3 B. J. H. Kuipers, H. Gruppen, *J. Agric. Food Chem.* 2007, **55**, 5445–5451.
4 G. Torsi, G. Chiavari, C. Laghi, A. M. Asmundsdottir, F. Fagioli, R. Vecchietti, *J. Chromatogr. A* 1989, **482**, 207–214.
